# Enantioselective synthesis of *N*-alkylindoles enabled by nickel-catalyzed C-C coupling

**DOI:** 10.1038/s41467-022-34615-9

**Published:** 2022-11-11

**Authors:** Lun Li, Jiangtao Ren, Jingjie Zhou, Xiaomei Wu, Zhihui Shao, Xiaodong Yang, Deyun Qian

**Affiliations:** 1 Key Laboratory of Medicinal Chemistry for Natural Resource, Ministry of Education, School of Chemical Science and Technology, Yunnan Provincial Center for Research & Development of Natural Products, and State Key Laboratory for Conservation and Utilization of Bio-Resources in Yunnan, Yunnan University, Kunming, China; 2 Southwest United Graduate School, Kunming, China

**Keywords:** Synthetic chemistry methodology, Asymmetric synthesis, Stereochemistry

## Abstract

Enantioenriched *N*-alkylindole compounds, in which nitrogen is bound to a stereogenic sp^3^ carbon, are an important entity of target molecules in the fields of biological, medicinal, and organic chemistry. Despite considerable efforts aimed at inventing methods for stereoselective indole functionalization, straightforward access to a diverse range of chiral *N*-alkylindoles in an intermolecular catalytic fashion from readily available indole substrates remains an ongoing challenge. In sharp contrast to existing C–N bond-forming strategies, here, we describe a modular nickel-catalyzed C–C coupling protocol that couples a broad array of *N*-indolyl-substituted alkenes with aryl/alkenyl/alkynyl bromides to produce chiral *N*-alkylindole adducts in single regioisomeric form, in up to 91% yield and 97% ee. The process is amenable to proceed under mild conditions and exhibit broad scope and high functional group compatibility. Utility is highlighted through late-stage functionalization of natural products and drug molecules, preparation of chiral building blocks.

## Abstract (web summary)

Enantioenriched *N*-alkylindole compounds, in which nitrogen is bound to a stereogenic sp^3^ carbon, are target molecules in the fields of biological, medicinal, and organic chemistry. Here the authors show a nickel-catalyzed C-C coupling protocol to synthesize these molecules from *N*-indolylsubstituted alkenes.

## Introduction

**Fig. 1:**
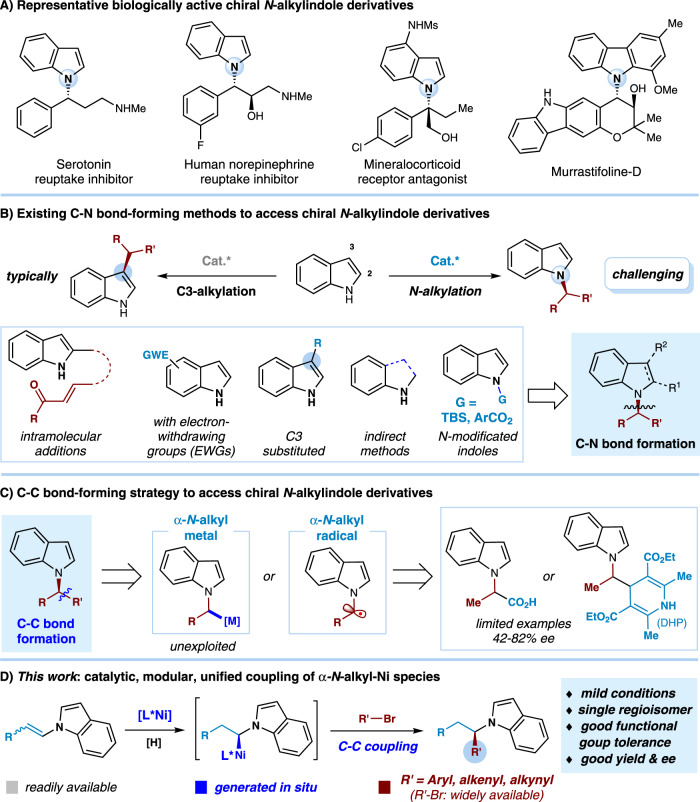
Representative chiral *N*-alkylindole derivatives and strategies for catalytic enantioselective synthesis of *N*-alkylindoles. **A** Representative biologically active chiral *N*-alkylindole derivatives; **B** Existing C–N bond-forming methods to access chiral *N*-alkylindole derivatives; **C** C–C bond-forming strategy to access chiral *N*-alkylindole derivatives; **D** This work: catalytic, modular, unified coupling of *α*-*N*-alkyl-Ni species.

Enantioenriched indole derivatives are of great interest in pharmaceutical science and organic chemistry^1–4^. Particularly, the indole core is one of the most frequent *N*-heterocyclic fragment featured in FDA-approved drugs^5^. Therefore, different methods have been designed for the construction of chiral indole scaffolds^6–9^. The most typical functionalizations of indoles take place at the C3 positions, due to their innate nucleophilicity^10,11^. In contrast, the development of techniques involving a stereocenter adjacent to the nitrogen, an essential structural motif imbedded in many biologically active molecules (Fig. 1A)^12–16^, remains a great challenge, presumably owing to the attenuated nucleophilicity of the nitrogen atom (Fig. 1B). To this end, a few powerful C-N bond-forming approaches have been developed to access chiral *N*-alkylindoles (Fig. 1B). However, these transformations often rely on the enantioselective intramolecular addition of prefunctionalized indole substrates^17–19^, or intermolecular *N*-alkylation (mostly *N*-allylation) of indoles with C3-blocking substituents^20–25^ or electron-withdrawing groups^26–33^. Moreover, indirect methods using indole precursors such as indolines or aryl hydrazines were also developed to obtain high regio- and enantioselectivity^34–36^. Recently, the Vilotijevic and Buchwald groups demonstrated elegant works using *N*-modificated strategy to engage *N*-silyl indoles and *N*-(benzoyloxy)indoles in C-N bond-forming reactions, respectively^37,38^. Despite these remarkable advances, a general, modular and selective synthesis of enantioenriched *N*-alkylindoles is in crucial demand, particularly if the substrates and catalysts are readily available^39,40^.

New strategic bond-forming reactions would offer a complementary protocol to existing C-N bond-forming process and an opportunity to explore currently inaccessible chemical space. In this regard, the enantioselective coupling of an *α-N*-alkyl metal species or an *α-N*-alkyl radical species represents a straightforward strategy to the synthesis of chiral alkylindoles (Fig. 1C)^41,42^. However, forging a C–C bond asymmetrically at the position *α* to the indole nitrogen remains elusive^43–45^. Recently, the Melchiorre^43^ and Davidson^44^ groups independently reported impressive works using photoredox chemistry to engage indole-derived *α-N*-alkyl radical intermediates in *N*-alkylindole synthesis, despite limited substrate scope and enantioselectivity (Fig. 1C, right). Moreover, in the the case, a leaving group (-CO_2_H or DHP) is necessary for the generation of an *α-N*-alkyl radical. In contrast, there have been no reports on generation and coupling of indole-derived *α-N*-alkyl metal species. The enantioselective Ni-catalyzed reductive coupling of olefins with electrophiles represents an attractive utilization of in-situ generated alkyl-Ni species^46–52^. We wondered whether this reductive coupling strategy could be harness to access chiral *N*-alkylindoles. However, catalytic enantioselective reductive coupling of *N*-alkenyl indoles faces several challenges. First, current asymmetric reductive coupling is largely limited to the use of liner alkyl–Ni intermediates^46–58^. Catalytic enantioselective coupling of branched alkyl–Ni intermediates generated from hydronickellation of olefins remains elusive^59–63^. Moreover, modulation of the site-selectivity pattern across differently substituted *N*-alkenyl indoles is unknown. In addition, the propensity of *N*-alkenyl polymerization and reduction is possible^64–67^.

As a part of our interest in chiral alkylamine-bearing molecules^25,68^, here, we show a catalytic enantioselective coupling of in-situ generated *α-N*-alkyl nickel species with aryl/alkenyl/alkynyl bromides, analogous to the C(sp^3^)–C(sp^2^)/C(sp) cross-coupling reaction, enabling a unified method toward structually diverse chiral *N*-alkylindoles in high yields and ee’s (Fig. 1D). By employing mild conditions, this modular, unified fragment coupling provides practical advantages in reaction efficiency, functional group compatibility, as well as substrate availability and scope, which would be broadly useful yet mechanistically orthogonal to established *N*-alkylation processes. In particular, application in late-stage diversifcation of many natural products and drug molecules demonstrates its utility in accelerating access to *N*-alkylated drug-like complexity.

## Results

### Reaction discovery and investigations

By the use of 4-bromophenylacetone as the coupling partner, we firstly examined the reaction of *N*-vinylindole **1a**, representing a class of nucleophiles that has previously been unexploited in asymmetric nickel-catalyzed reactions although has been widely utilized as monomers for the synthesis of polymeric materials^64,69^. Upon investigating a series of reaction parameters (Table 1 and Supplementary Tables 1–6), we discovered that running of the cross-coupling partners at 40 °C for 20 h in the presence of NiCl_2_•DME, commercially available (*S*,*S*)-diphenyl-Box ligand (L*1), a hydride source (diethoxy-methylsilane), and a base (KF) provides the desired product in 36% yield and 79% enantiomeric excess (ee) as a single isomer (entry 1). Compared to L*1, employing bis-oxazoline analogs L*2-L*4 bearing alkyl substitutents (*R*) almost did not produce **3a**. Moreover, the isopropylidene bridge of L*1 proved to be essential, as L*5 with *gem*-H could not afford the product. Inferior results were found with other chiral nitrogen-based ligands, such as pyridine-oxazoline (Pybox) and 2,2-bis(2-oxazoline) (Bi-Ox) ligands (entries 3–4). Further solvent screening indicated 1,2-dimethoxyethane was superior to other solvents, producing **3a** in good yield and ee under room temperature (entry 5). Interestingly, the mixed solvent (DME/DCE) turned out as the best solvent to obtain both high yield and high enantioselectivity (entries 5–7), thus indicating the subtle interplay of reagents and solvents in this case. In addition, use of other nickel salts, NiBr_2_•DME resulted in higher ee (entry 8), while NiI_2_•xH_2_O gave the best result (entry 9).

**Table 1:** Summary of the effects of crucial reaction parameters^a^


Entry	Variant	Yield (%)	ee (%)^b^
1	L* = L*1	36	79
2	L* = L*2, L*3, L*4, or L*5	trace	n.d.
3	L* = L*6	34	16
4	L* = L*7	62	58
5	L* = L*1, DME as solvent, rt	80	81
6	L* = L*1, DCE as solvent, rt	16	88
7	L* = L*1, DME/DCE (3:1) as solvent, rt	82	86
8	NiBr_2_•DME as Ni-Cat. *vs*. entry 7	78	88
9	NiI_2_•xH_2_O as Ni-Cat. *vs*. entry 7	81 (77)^c^	92

^a^See the SI for experimental details; all reactions were carried out in 0.2 mmol scale with respect to **1a**; corrected ^1^H NMR yields using CH_2_Br_2_ as an internal standard were reported. ^b^The enantiomeric excesses (ee’s) were determined by HPLC analysis. ^c^Isolated yield is shown in the parenthesis. L* chiral ligand; *DME* 1,2-dimethoxyethane; *DCE* 1,2-dichloroethane, *rt* room temperature; *h* hour; *n.d.* not detected.

### Reaction scope

**Fig. 2:**
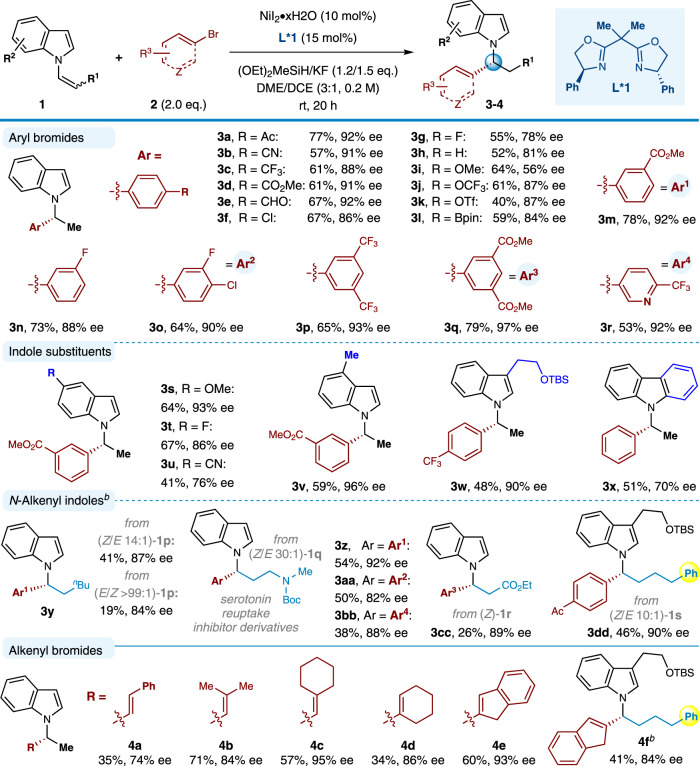
Scope of enantioselective synthesis of *N*-benzyl and *N*-allylic indoles enabled by Ni-catalyzed C(sp^3^)-C(sp^2^) coupling reactions. Conditions: ^*a*^All reactions were carried out with NiI_2_•xH_2_O (10 mol%), ligand L*1 (15 mol%), **1** (0.40 mmol), **2** (0.80 mmol), (OEt)_2_MeSiH (0.48 mmol), KF (0.60 mmol) and DME/DCE (3:1, 2.0 mL) at room temperature for 20 h; ^*b*^DME as solvent, (OEt)_2_MeSiH (0.72 mmol), KF (0.88 mmol), 48 h.

The generality of this catalytic enantioselective method is broad (Fig. 2). Concerning the aryl bromide, the reaction proceeded smoothly with a wide array of substrates to provide the corresponding products in moderate to high yields with universally high enantioselectivities. Notably, electron-deficient or electron-rich arenes were amenable coupling partners, in which the substituent could be placed at *para* and *meta* position. A variety of functionalities such as a nitrile (**2b**), trifluoromethyls (**2c, 2p, 2r**), esters (**2d, 2m, 2q**), halides (**2f, 2** **g, 2n, 2o**), ethers (**2i, 2j**) were all readily accommodated. Despite the ability of nickel complex to activated aryl chlorides, our approach could tolerate Ar-Cl groups (**2f, 2o**). In particular, sensitive functional groups including easily reduced ketone (**2a**) and aldehyde (**2e**), and triflate (**2k**) and boronic acid pinacol ester (**2l**) commonly used for cross-coupling, all remained intact under the standard reaction conditions. Furthermore, the pharmaceutically important heterocycle–pyridine (**2r**) was compatible as well.

Next, we sought to survey the influence of the *N*-vinylindole variants that could be used in the catalytic hydroarylation event. Delightfully, a diverse array of functional groups were suitable at different positions on the benzene ring of the indole, including a 6-methoxy (**3s**), 6-fluoro (**3t**), 6-cyano (**3u**), and 5-methyl (**3v**) substituent. It is worth noting that alkyl group at the C3-position of the indole scaffold was accommodated, delivering the corresponding product **3w** in moderate yield but with excellent enantioselectivity, which is difficult to obtain employing previous CuH catalysis^38^. In addition, carbazole-derived substrate afforded the desired *N*-alkylated product in moderate yield and enantioselectivity (**3x**). Gratifyingly, a diverse set of more sterically hindered (*Z*)-*N*-alkenyl indoles were also successfully transformed utilizing this method, and the corresponding *N*-alkylindoles were readily prepared in useful yields with good levels of enantioselectivity (**3y-3dd**). However, (*E*)-*N*-alkenyl indoles performed lower reactivity and slightly lower enantioselectivity than their *Z* isomers (e.g., **3y**: 19%, 84% ee *vs*. 41%, 87% ee). The isomerization of *Z-N*-alkenyl substrate to its *E* isomer was observed during the reaction process, and over 40% of the *E*/*Z* mixture could be recovered after the reaction (Supplementary Method 1.6). Of note, the C–C bond-forming event occurs regioselectively at the carbon α to the nitrogen of indoles, even in the presence of other directing groups such as amide (**3z-3bb**), ester (**3cc**), and aryl (**3dd**). Especially, this modular reaction could be applied to prepare important serotonin reuptake inhibitor derivatives with good efficiency and enantioselectivities (**3z-3bb**, 81–92% ee’s).

In addition to aryl bromides, vinyl bromides were incorporated as well in this reaction, leading to structually diverse chiral *N*-allyl indoles **4a-4f** in 35–60% yields with 73–95% ee values. Remarkably, this modular alkenylation complements previously established metal-catalyzed indole *N*-allylations in that di- and trisubstituted allylic products bearing aryl and alkyl groups are readily accessed^27,29,31,70,71^.

**Fig. 3:**
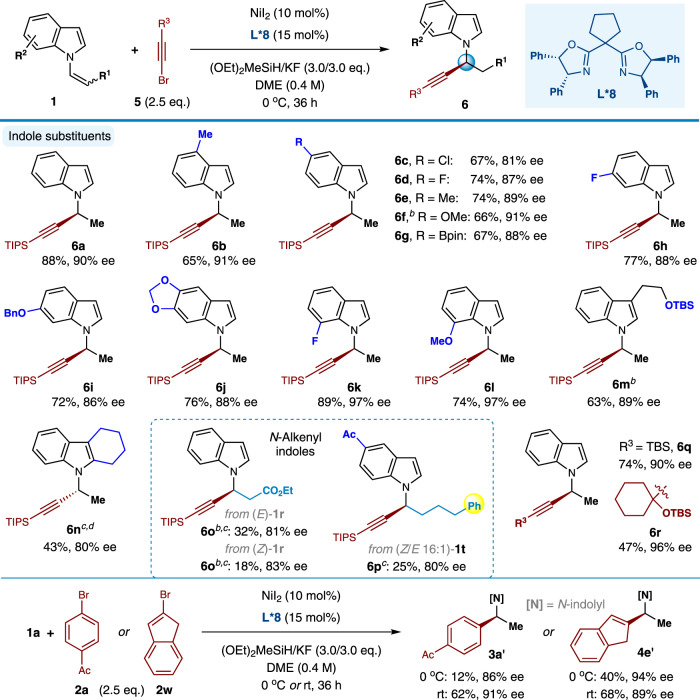
Scope of enantioselective synthesis of *N*-propargyl indoles enabled by Ni-catalyzed C(sp^3^)-C(sp) coupling reactions. Conditions: ^*a*^ All reactions were carried out with NiI_2_ (10 mol%), ligand L*8 (15 mol%), **1** (0.10 mmol), **5** (0.25 mmol), (OEt)_2_MeSiH (0.30 mmol), KF (0.30 mmol) and DME (0.25 mL) at 0 °C for 36 h; ^*b*^ NiI_2_**·**xH_2_O instead of NiI_2_; ^*c*^ MeCN instead of DME; ^*d*^ Pybox ligand (*S*,*S*)-L*17 instead of L*8, K_3_PO_4_ instead of KF, 40 °C, 24 h (see SI for details). TIPS = triisopropylsilyl; TBS = *t*-butyldimethylsilyl.

Besides the C(sp^2^) bromides, this catalytic C-C bond-forming reaction was also viable for C(sp) bromides–bromoalkynes **5**. While the above standard conditions with ligand L*1 resulted in poor enantiocontrol for the C(sp^3^)-C(sp) coupling (e.g., **6a**: 28% yield, 55% ee), delightfully, the reaction selectivity could be significantly improved by further optimization efforts (see Supplementary Table 7). As shown in Fig. 3, the treatment of bromoalkynes **5** and *N*-alkenylindoles** 1** with 10 mol% NiI_2_ as the catalyst, 15 mol% L*8 as the ligand at 0 °C could yield the corresponding chiral *N*-propargyl indoles **6** in mostly good yields (25–89%) and high levels of enantioselectivity (ee values of 80–97%). A variety of *N*-alkenyl indoles substituted at the 4-position (**6b**), 5-position (**6c-6g**), 6-position (**6h-6j**), and 7-position (**6k, 6l**) each underwent efficient hydroalkynylation to provide the corresponding products with uniformly high enantioselectivities. Of note, alkyl group at the C3-position of the indole scaffold was demonstrated being tolerated again (**6m**). With regard to medicinal chemistry applications, the generation of product **6g** demonstrates tolerance of a pinacol boronate subunit under the conditions of catalytic enantioselective alkynylation. Additionally, more sterically hindered *cis-β*-substituted *N*-alkenyl indoles also successfully underwent C(sp^3^)-C(sp) bond-formation to deliever compounds **6o** and **6p**, respectively, as single regioisomers with reasonable yields and good enantioselectivities. Similar to the C(sp^3^)-C(sp^2^) coupling, the reaction reactivity and stereoselectivity was influenced by the *Z* and *E* configuration of *N*-alkenyl indoles (e.g., **6o**).

Beyond the TIPS-substituted ethynyl bromide **5a**, TBS- and 3° alkyl-substituted ethynyl bromides **5b-5c** proved to be viable coupling partners in this system, affording the desired indole adducts (**6q-6r**) in 47–74% yields with excellent enantioselectivities. However, ethynyl bromides with less steric hindered alkyl and aryl substitutents delivered inferior results (e.g., R^3^ = *n-*Bu: no reaction; R^3^ = Ph: 26% yield, 50% ee). In addition, it should be noted that the current L*8-ligated nickel catalysis could be expanded to enable the synthesis of chiral *N*-benzyl and *N*-allylic indoles in high enantioselectivities (e.g., **3a’** and **4e’**).

### Synthetic applications

**Fig. 4:**
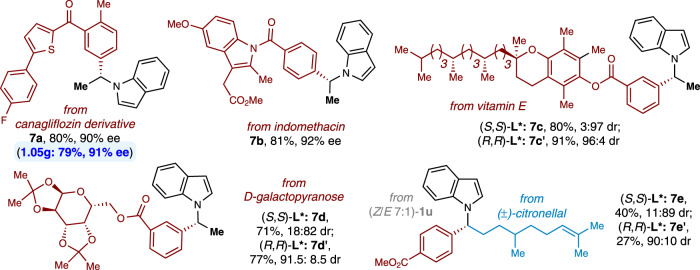
Late-stage diversification of drug molecules and natural products. Conditions: All reactions were carried out with NiI_2_•xH_2_O (10 mol%), ligand L*1 (15 mol%), **1a** or citronellal-derived **1u** (0.40 mmol), drugs- or ntural products-derived **2** (0.80 mmol), (OEt)_2_MeSiH (0.48 or 0.72 mmol), KF (0.60 or 0.88 mmol) and DME/DCE (3:1, 2.0 mL) at room temperature for 20 or 48 h.

More importantly, the present method could be applied in late-stage diversification of complex drug molecules and natural products^72^. As depicted in Fig. 4, aryl bromides derived from complexe bioactive molecules canagliflozin derivative, an antidiabetic drug (**7a**), indomethacin, a nonsteroidal anti-inflammatory drug (**7b**), and vitamin E, an antioxidant (**7c**), coupled with *N*-vinylindole **1a** in good yields and stereoselectivities, thus revealing the appeal that our approach might have for lead generation protocols in drug discovery. Furthermore, aryl bromides or *N*-alkenyl indoles bearing multiple stereocenters originated from vitamin E (**7c**), D-galactopyanose (**7d**), and citronellal (**7e**) were all viable substrates, affording potentially valuable *N*-alkyl adducts in synthetically useful yields and high diastereoselectivity. To further showcase the robustness and synthetic utility of the method, the catalytic enantioselective synthesis of *N*-benzyl indole **7a** on a gram scale was carried out with similar efficiency (Supplementary Method 1.7).

**Fig. 5:**
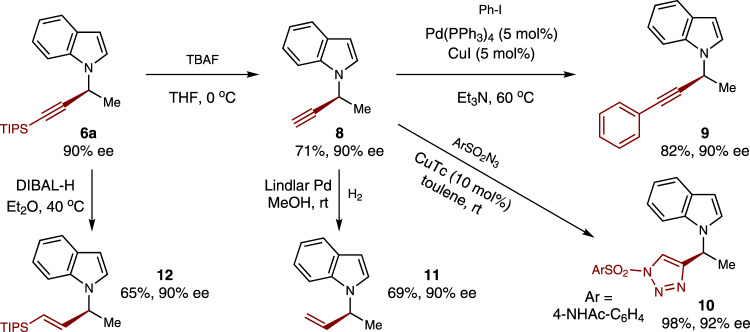
Derivatizations of chiral *N*-propargyl indoles. Desilylation and alkynyl reduction of compound **6a**; Sonogashira coupling, [3 + 2] cycloaddition, and hydrogenation of compound **8**. DIBAL-H = diisobutylaluminum hydride; Lindlar Pd = Pd/BaSO_4_; CuTc = copper(I) thiophene-2-carboxylate.

Notably, the alkynyl group on the chiral *N*-propargyl indoles prodvided a useful and versatile handle for derivatizations (Fig. 5). For eaxmple, desilylation of **6a** provided the enantioenriched terminal alkyne **8**, which subsequently underwent the Sonogashira coupling to afford the aryl-substituted alkynye product **9**. Terminal alkyne **8** underwent a click reaction to give chiral triazole **10**. Reduction of **8** with Lindlar Pd and H_2_ afforded chiral *N*-allylic indole **11**, while reduction of **6a** with DIBAL-H produced chiral vinylsilyl compound **12**.

### Mechanistic studies

**Fig. 6:**
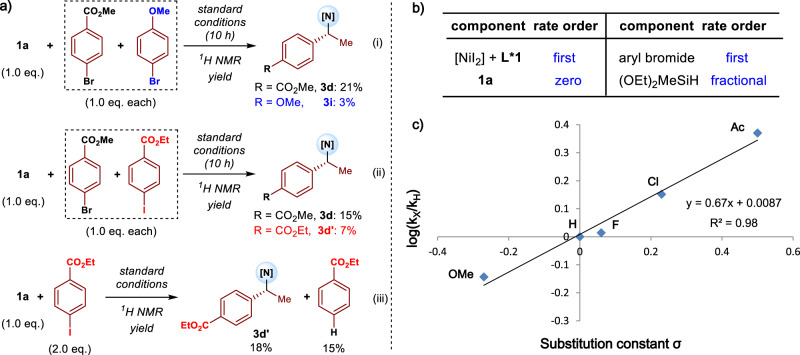
Mechanistic experiments. **a** Competition experiments ([N] = *N*-indolyl); **b** Initial rate experiments; **c** Hammett study for the formation of **3** versus the corresponding *σ* value (k = reaction rate).

To gain insight into the mechanism and origin of selectivity, a series of experiments well conducted. Generally, the reductive coupling process consists of *π*-bond insertion into L*Ni−H species, oxidative addition of the resulting L*Ni-alkyl intermediate, and reductive elimination to form product and the L*Ni-H catalyst^46–63^. Take the reductive coupling of *N*-alkenyl indoles with aryl halides as an example, competition experiments were performed to compare the reactivity between different aryl halides, indicating that (i) electron-deficent aryl bromide is more reactive than an electron-rich one (Fig. 6a–i), and (ii) aryl bromide is more reactive than aryl iodide (Fig. 6a–ii). In fact, a competing reductive hydrodehalogenation event was observed when an aryl iodide was uesd as the electrophile (Fig. 6a–iii)^73,74^, suggesting it is disadvantaged for the final product formation that oxidative addition of L*Ni complex prior to generation of L*Ni-alkyl intermediate. Next, the reductive coupling of **1a** was chosen for kinetic studies, and the reaction progress was monitored by ^19^F and ^1^H NMR. Initial rate experiments disclosed that the reaction was zero-order in *N*-alkenyl indole, first-order in catalyst and aryl bromide, and fractional-order in diethoxymethylsilane (Fig. 6b, see also Supplementary Method 1.9.1). Moreover, Hammett studies were also performed to evaluate the influence that electronic variation of the aryl electrophiles had on the rate of hydroarylation (Fig. 6c)^75,76^. As a result, a variety of *para*-substituted aryl bromides reacted with *N*-vinylindole **1a** at different rates, indicating that electronic variation of the aryl electrophile had a remarkable impact on the rate of *N*-vinylindole hydroarylation. A linear relationship was further observed through a Hammett plot. The positive slope (*ρ* = 0.67) suggests negative charge accumulation in the turnover-determining transition state, which is stabilized by electron-withdrawing substituents. Taken together, the above results reveal that oxidative addition is most likely the turnover-limiting step.

**Fig. 7:**
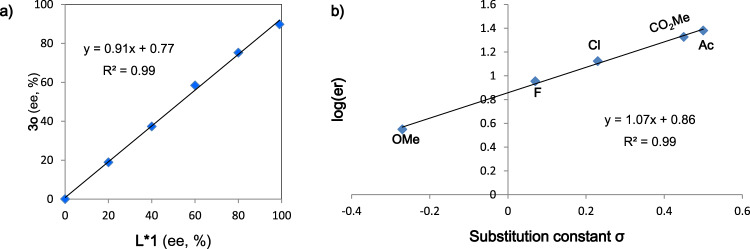
Further mechanistic experiments. **a** Nonlinear effect study; **b** Hammett plot for the enantiomeric ratio (er) of hydroarylation products using *para*-substituted aryl bromides.

Furthermore, a linear correlation was observed by nonlinear effect studies on the enantiomeric composition of chiral ligand L*1 and *N*-alkylindole product **3o** (Fig. 7a), which is consistent with a ligated nickel catalyst being of a monomeric nature. To identify the enantiodetermining step of the *N*-vinylindole hydroarylation reaction, we next investigated linear free energy relationships (LFERs) between the Hammett electronic parameters of various *para*-substituted aryl bromides and the enantioselectivities of the corresponding products (Fig. 7b)^77,78^. A linear correlation was observed with *para*-substituted aryl bromides as enantioselectivity increased with the introduction of electron-withdrawing groups (*ρ* = 1.07): 56% ee and 92% ee were observed for 4-methoxyphenyl bromide (*σ* = −0.27) and 4-acetylphenyl bromide (*σ* = 0.50), respectively, implying that the enantioselectivity of the process is not solely under catalyst control. In addition, the silane did essentially not affect the enantioselectivity of the reaction. On the basis of these results, oxidative addition is most likely the enantiodetermining step.

**Fig. 8:**
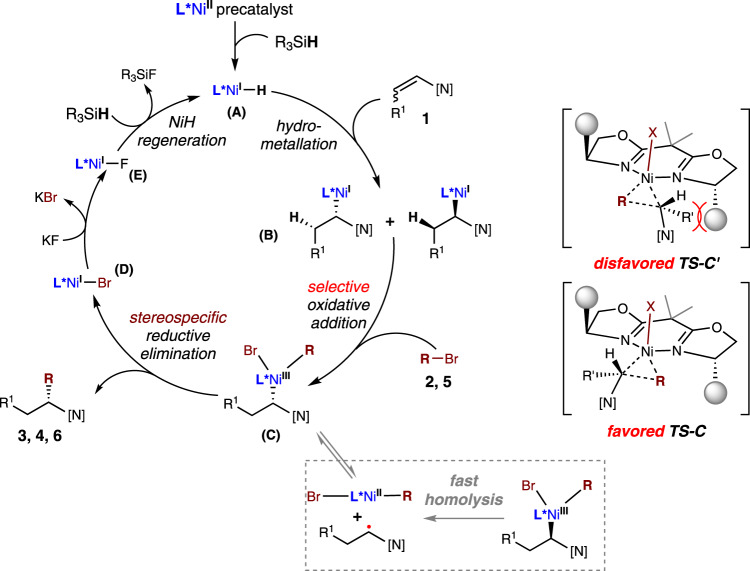
Possible reaction pathway. The selective oxidative addition is proposed to be both the turnover-determining step and the enantio-determining step. [N] = *N*-indolyl; TS = transition state.

As shown in Fig. 8, a more complete description of the proposed mechanism is outlined. The *syn*-hydrometallation of an L*Ni-H species into an *N*-alkenyl indole would form alkyl-Ni(I) species (**B**). Subsequently, the selective oxidative addition between a particular isomer of the alkyl-Ni(I) species and the bromide (**2**, **5**) would ultimately generate a single alkyl-Ni(III)-R enantiomer (**C**), because this step would be both the turnover-determining step and the enantio-determining step in the presence of a chiral ligand (L*1, L*8). In particular, the favored enantioselective transition state **TS-C** would lead to the major enantiomeric product, owing to steric interations with the ligand phenyl substituents (**TS-C’**). Then, stereospecific reductive elimination would afford the desired product (**3**, **4**, **6**, **7**) and regenerate the active nickel hydride species (**A**). Alternatively, the competitive alkyl-Ni homolysis process was also possible^53,55,62,79^.

In summary, we have developed a nickel-catalyzed enantioselective, modular coupling of indole-based *N*-alkyl-Ni fragments with C(sp^2^)/C(sp) bromides. By the use of easily accessible and stable indole-derived alkenes as nucleophiles, this protocol enables streamlined preparation of enantioenriched *N*-alkylindole molecules under mild conditions, with previously inaccessible functional group tolerance and chemical space. Application in late-stage diversification of several complex drug molecules and natural products as well as chiral syntheses demonstrates its potential utility in the synthesis of valuable chiral *N*-alkylated bioactive compounds.

## Methods

### General procedure for the enantioselective synthesis of *N*-benzyl and *N*-allylic indoles

To an oven-dried 8.0 mL Teflon-screw cap test tube containing a magnetic stir was charged with NiI_2_•xH_2_O (16.8 mg, 10 mol%) and ligand L*1 (20.2 mg, 15 mol%) under an N_2_ atmosphere using glove-box techniques. Subsequently, anhydrous DME (1.5 mL) was added, and the mixture was stirred for 15 min at room temperature. Next, KF (35.0 mg, 0.60 mmol, 1.5 equiv.), *N*-alkenyl indole **1** (0.40 mmol, 1.0 equiv), aryl/alkenyl bromide **2** (0.80 mmol, 2.0 equiv.), DCE (0.5 mL), and (OEt)_2_MeSiH (78.0 uL, 0.48 mmol, 1.2 equiv.) were sequentially added. Then the tube was sealed with airtight electrical tapes and removed from the glove box and stirred at room temperature for 20–48 h. After that, the reaction mixture was diluted with saturated NH_4_Cl (aq., 1.0 mL) and EtOAc (5.0 mL). The aqueous phase was extracted with EtOAc (2 × 5.0 mL) and the combined organic phases were concentrated. The crude mixture was purified by flash column chromatography on silica gel using a mixture of PE/EtOAc as eluent to obtain the desired product **3, 4, 7**.

### General procedure for the enantioselective synthesis of *N*-propargyl indoles

To an oven-dried 12 mL Teflon-screw cap test tube containing a magnetic stir was charged with NiI_2_ (3.1 mg, 10 mol%) and ligand L*8 (7.7 mg, 15 mol%) under a nitrogen N_2_ atmosphere using glove-box techniques. Subsequently, anhydrous DME (0.25 mL) was added, and the mixture was stirred for 30 min at room temperature. Next, KF (17.4 mg, 0.30 mmol, 3.0 equiv), *N*-alkenyl indole **1** (0.10 mmol, 1.0 equiv), alkynyl bromide **5** (0.25 mmol, 2.5 equiv), and (OEt)_2_MeSiH (43.0 µL, 0.30 mmol, 3.0 equiv.) were sequentially added. Then the tube was sealed with airtight electrical tapes and removed from the glove box and stirred at 0 °C for 36 h. After that, the reaction mixture was diluted with saturated NH_4_Cl (aq., 1.0 mL) and EtOAc (5.0 mL). The aqueous phase was extracted with EtOAc (2 × 5.0 mL) and the combined organic phases were concentrated. The crude mixture was purified by flash column chromatography on silica gel using a mixture of PE/EtOAc as eluent to obtain the desired product **6**.

## Supplementary information

Supplementary Information

## Data Availability

The data relating to the materials and methods, experimental procedures, mechanism research, NMR spectra, and HPLC spectra are available in the Supplementary Information. All other data are available from the authors upon request.
