# Supplementary material for: Enantioselective synthesis of N-alkylindoles enabled by nickel-catalyzed C-C coupling
Source: Nat Commun. 2022 Nov 11;13:6861. doi: 10.1038/s41467-022-34615-9 (PMC9652415; doi:10.1038/s41467-022-34615-9)
Supplement: Supplementary file 1 — Supplementary Information [file 41467_2022_34615_MOESM1_ESM.pdf]

Supplementary Information

Enantioselective Synthesis of *N*-Alkylindoles Enabled by Nickel-Catalyzed C-C Coupling

Lun Li[1,#], Jiangtao Ren[1,2,#], Jingjie Zhou[1], Xiaomei Wu[1], Zhihui Shao[1,2,*], Xiaodong Yang[1,*] & Deyun Qian[1,*]

[1] Key Laboratory of Medicinal Chemistry for Natural Resource, Ministry of Education, School of Chemical Science and Technology, Yunnan Provincial Center for Research & Development of Natural Products, and State Key Laboratory for Conservation and Utilization of Bio-Resources in Yunnan, Yunnan University, Kunming 650500, China.
[2] Southwest United Graduate School, Kunming 650500, China.
[#] These authors contributed equally: Lun Li & Jiangtao Ren
E-mail: dyqian@ynu.edu.cn

Table of Contents

1. Supplementary Methods S2
1.1 General Information S2
1.2 Optimization of the Reaction Conditions S3
1.3 Synthesis of *N*-Vinylindole S11
1.4 Preparation of Aryl Halides, Alkenyl Halides, and Alkynyl Bromides S21
1.5 Ni-Catalyzed Enantioselective Synthesis of *N*-Alkyl Indoles S23
1.6 Competition Experiment of (*Z*)- and (*E*)-*N*-Alkenyl Indoles S65
1.7 Gram-Scale Experiment S65
1.8 Derivatization of Coupling Products S66
1.9 Mechanistic Study S70
1.10 Determination of the Absolute Configuration S79
1.11 NMR and HPLC Spectra S80
2. Supplementary References S332

1. Supplementary Methods

1.1 General Information

All reactions were set up in a 8 mL Teflon-screw capped test tubes (unless otherwise noted) under an inert nitrogen (N_2) atmosphere. Solvents were purified under nitrogen using a solvent purification system.

Analytical thin layer chromatography (TLC) was performed using silica gel plates. Visualisation was by ultraviolet fluorescence, and/or phosphomolybdic acid, and/or $KMnO_4$.

Flash column chromatography (FC) was performed using *Qingdao* (200-300 mesh) silica gel.

NMR: ^{1}H and ^{13}CNMR spectra were recorded on a Bruker Avance 500 Spectrometer. ^{1}H and ^{13}C chemical shifts were referenced internally to residual solvent peaks relative to TMS (δ = 0 ppm) at 299 K. Chemical shifts (δ (ppm)) are reported relative to TMS (δ (^{1}H) 0.0 ppm, δ (^{13}C) 0.0 ppm). The solvents' residual proton resonance and the respective carbon resonance (for $CHCl_3$; δ (^{1}H) 7.26 ppm, δ (^{13}C) 77.0 ppm were used for calibration.

HPLC spectra were recorded on an *Agilent* HPLC. Column, eluent and retention times for HPLC analysis used for the determination of enantiomeric ratios are given below in the details of the relevant experiments.

Optical rotations were measured on a *JASCO* DIP-370 polarimeter.

High-resolution mass spectra (HRMS) by electrospray ionization (ESI) method were performed at the *Agilent* 1100 LC/MSD TOF mass spectrometer.

All reagents were either prepared according to known literatures or purchased from *Sigma-Aldrich*, *TCI*, *Acros Organics*, *Alfa Aesar*, *Bide-pharmatech* and *Tansoole*.

NiI_2•xH_2O (CAS: 7790-34-3) was purchased from *Bide-pharmatech*;

KF (white powder, CAS: 7789-23-3) was purchased from *Acros Organics*;

$(EtO)_2MeSiH$ (CAS: 2031-62-1) was purchased from *Alfa Aesar*;

DME (1,2-Dimethoxyethane, SafeDry, over molecular sieve, CAS: 110-71-4) was purchased from *Adamas-beta*.

1.2 Optimization of the Reaction Conditions

General Procedure: To an oven-dried 8.0 mL Teflon-screw cap test tube containing a magnetic stir was charged with a **Ni-salt** (10 mol%) and the corresponding ligand (15 mol%) under an inert nitrogen (N_2) atmosphere using glove-box techniques. Subsequently, anhydrous solvent (1.0 mL) was added, and the mixture was stirred for 1.0 hour at room temperature. Then, a base (1.5 equiv), *N*-vinylindole **1a** (27.0 uL, 0.20 mmol, 1.0 equiv), 4′-bromoacetophenone **2a** (60.0 mg , 0.30 mmol, 1.5 equiv), $(OEt)_2MeSiH$ (38.6 uL, 1.2 equiv.) were sequentially added. Afterwards, the tube was sealed with airtight electrical tapes and removed from the glove box and stirred at 40 ^{o}C for 24 hours at 650 rpm unless otherwise noted. After the reaction was completed, the reaction mixture was diluted with saturated NH_4Cl (aq., 0.5 mL) and EtOAc (3.0 mL). Then, the aqueous phase was extracted with EtOAc (2 × 3.0 mL). The combined organic phases were dried over Na_2SO_4, and the volatiles were removed to afford the crude product. CH_2Br_2 (14.0 μL, 0.20 mmol) was added as an internal standard and a small aliquot of the organic phase was removed for ^{1}H NMR analysis. Finally, the mixture was purified by PTLC and the enantiomeric excess was determined by HPLC analysis.

Supplementary Table 1. Screening of chiral ligands[a]

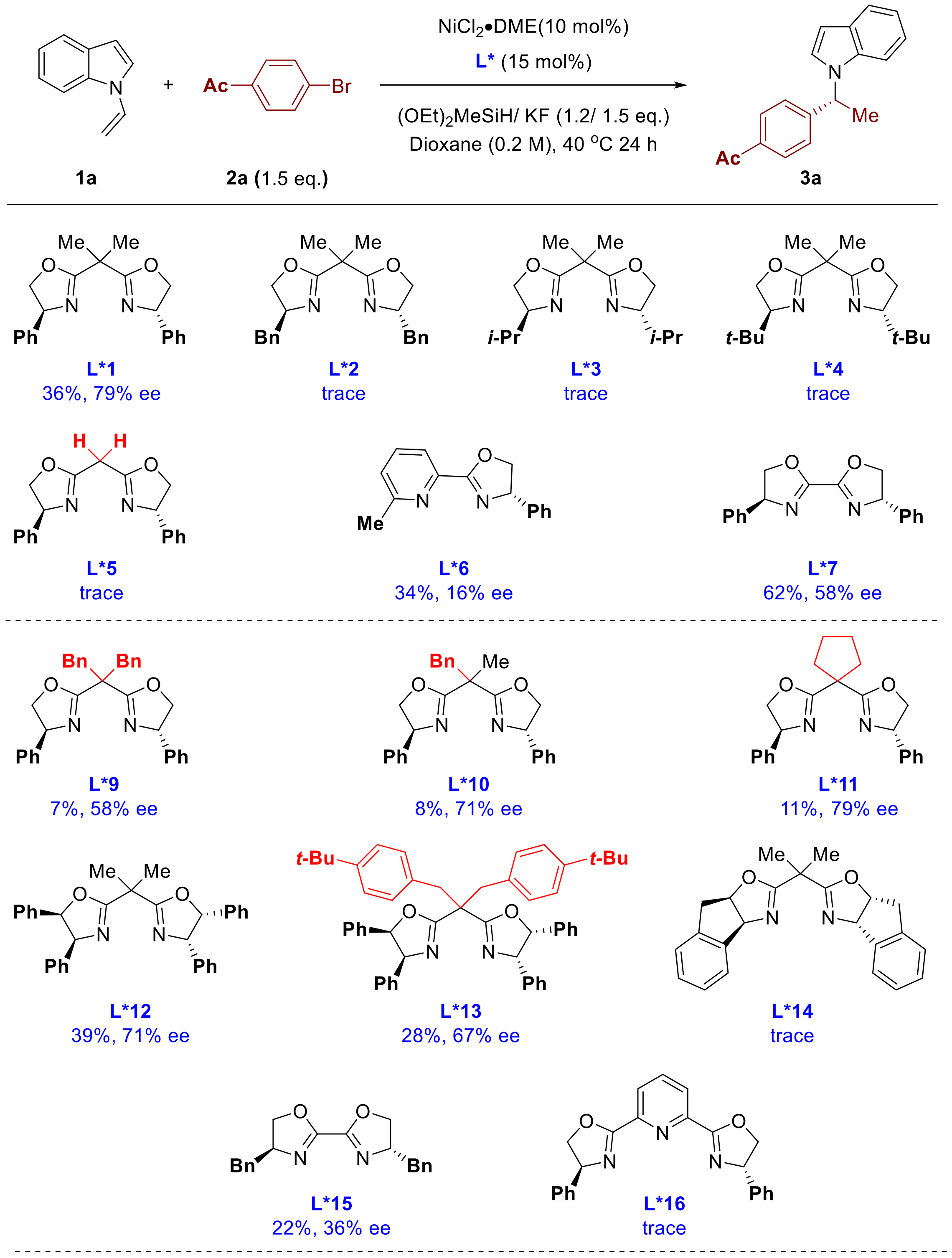

[a] Conditions: **1a** (0.20 mmol, 1.0 equiv), **2a** (0.30 mmol, 1.5 equiv), $NiCl_2$•DME (10 mol%), **L*** (15 mol%), $(EtO)_2MeSiH$ (0.24 mmol), KF (0.30 mmol), Dioxane (1.0 mL), 40 ℃, 20 h. [b] Yields were determined by ^1H NMR, using CH_2Br_2 as the internal standard. Ees were determined by HPLC.

Supplementary Table 2. Screening of solvents[a]

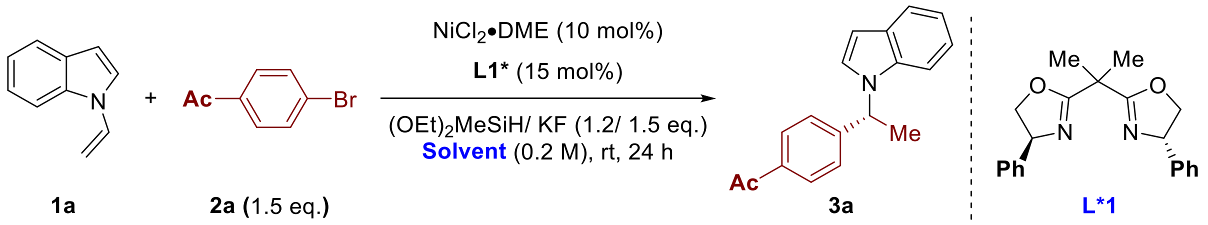

Entry	Solvents	Yield (%)[b]	ee (%)[c]
1	Dioxane	25	85
2	THF	23	83
3	2-Me-THF	69	81
4	DME	80	81
5	Et_2O	trace	N.D.
6	MTBE	trace	N.D.
7	DMF	41	59
8	DMA	39	81
9	NMP	67	63
10	DMSO	53	62
11	CF_3Ph	7	3
13	CyH	trace	N.D.
14	DCE	16	88
15	2-Me-THF/DCE (1:1)	79.3	82
16	2-Me-THF/Dioxane (1:1)	33	86
17	DME/DCE (1:1)	72	87
17	DME/DCE (3:1)	82	86

[a] Conditions: **1a** (0.20 mmol, 1.0 equiv), **2a** (0.30 mmol, 1.5 equiv), $NiCl_2$•DME (10 mol%), **L*1** (15 mol%), $(EtO)_2MeSiH$ (0.24 mmol), KF (0.30 mmol), **Solvent** (1.0 mL), rt, 24 h. [b] Yields were determined by ^1H NMR, using CH_2Br_2 as the internal standard. Ees were determined by HPLC. DME = 1,2-Ethanediol dimethyl ether, MTBE = tert-Butyl methyl ether, DMF = *N,N*-Dimethylformamide, DMA = *N,N*-Dimethylacetamide, NMP = 1-Methyl-2-pyrrolidinone, DMSO = Dimethyl sulfoxide, DCE = 1,2-Dichloroethane.

Supplementary Table 3. Screening of Ni-sources[a]

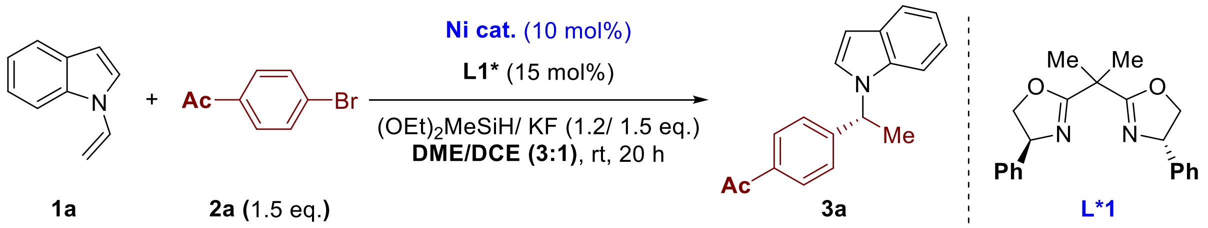

Entry	Ni cat.	Solvents	Yield (%)[b]	ee (%)[c]
1	$NiCl_2$•dme	DME/DCE (3:1)	82	86
2	$NiBr_2$•dme	"	78	88
3	$NiBr_2$•diglyme	"	70	89
4	$NiBr_2$	"	trace	N.D.
5	NiI_2	"	59	92
6	NiI_2•xH_2O	"	69	92
7	$Ni(ClO_4)_2$•6H_2O	"	63	89
8	$Ni(BF_4)_2$•6H_2O	"	86	90
9	$Ni(COD)_2$	"	91	88
10	NiI_2•xH_2O	2-Me-THF/DCE (3:1)	83	90
11	"	DME/DCE (1:3)	46	91
12	"	DME/DCE (1:1)	61	92
13	"	DME/DCM (1:1)	35	94
14	"	DME/TTCE (1:1)	trace	N.D
15	"	Dioxane/DCE (1:1)	63	91
16	"	Diglyme/DCE (1:1)	45	91
17	"	2-Me-THF/DCE (1:1)	79	90

[a] Conditions: **1a** (0.20 mmol, 1.0 equiv), **2a** (0.30 mmol, 1.5 equiv), **Ni cat.** (10 mol%), **L*1** (15 mol%), $(EtO)_2MeSiH$ (0.24 mmol), KF (0.30 mmol), solvent (1.0 mL), rt, 20 h. [b] Yields were determined by ^1H NMR, using CH_2Br_2 as the internal standard. Ees were determined by HPLC.

Supplementary Table 4. Screening of [H] source[a]

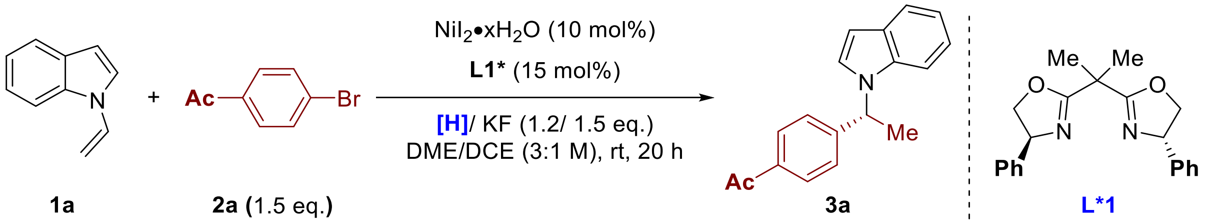

Entry	[H]	Yield (%)[b]	ee (%)[c]
1	$(EtO)_2MeSiH$	69	92
2	$(EtO)_3SiH$	29	91
3	$(MeO)_3SiH$	13	92
4	$(Me_3SiH)_2O$	19	88
5	PMHS	65	91
6	HBPin	47	78

[a] Conditions: **1a** (0.20 mmol, 1.0 equiv), **2a** (0.30 mmol, 1.5 equiv), NiI_2•xH_2O (10 mol%), **L*1** (15 mol%), **[H]** (0.24 mmol), KF (0.30 mmol), DME/DCE (3:1, 1.0 mL), rt, 20 h. [b] Yields were determined by ^1H NMR, using CH_2Br_2 as the internal standard. Ees were determined by HPLC.

Supplementary Table 5. Screening of bases[a]

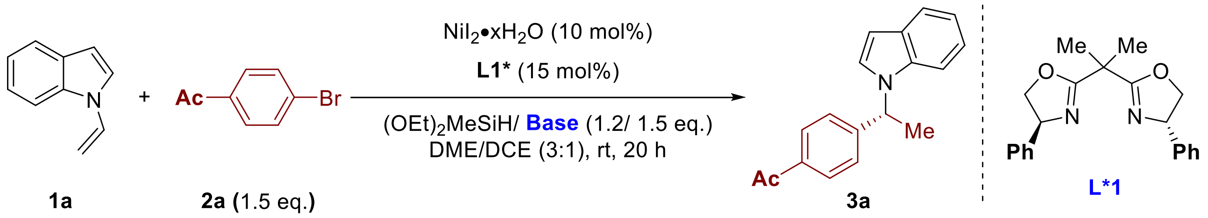

Entry	Bases	Yield (%)[b]	ee (%)[c]
1	KF	69	92
2	CsF	12	91
3	Na_2CO_3	32	92
4	Cs_2CO_3	10	84
5	K_3PO_4	8	87
6	K_3PO_4•H_2O	trace	N.D.

[a] Conditions: **1a** (0.20 mmol, 1.0 equiv), **2a** (0.30 mmol, 1.5 equiv), NiI_2•xH_2O (10 mol%), **L*1** (15 mol%), $(EtO)_2MeSiH$ (0.24 mmol), base (0.30 mmol), DME/DCE (3:1, 1.0 mL), rt, 20 h. [b] Yields were determined by ^1H NMR, using CH_2Br_2 as the internal standard. Ees were determined by HPLC.

Supplementary Table 6. Control experiments[a]

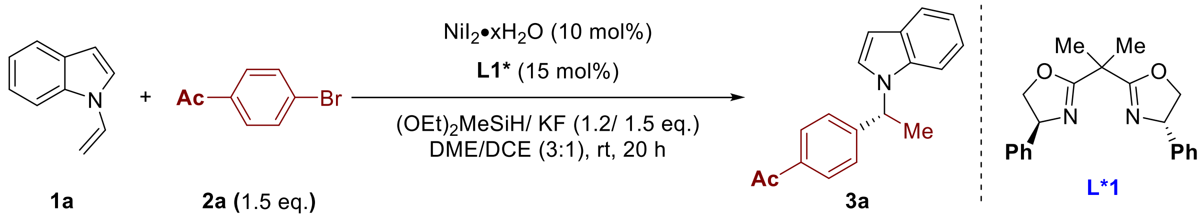

Entry	Deviation	Yield (%)[b]	ee (%)[c]
1	none	69	92
2	no Ni Cat.	0	N.D.
3	no **L*1**	0	N.D.
4	no $(EtO)_2MeSiH$	0	N.D.
5	no KF	0	N.D.
6	at air	65	91
7	**2a** (1.2 eq.)	62	92
8	**2a** (1.8 eq.)	72	92
9	**2a** (2.0 eq.)	81	92
10	$(EtO)_2MeSiH$/ KF (1.5/ 1.8 eq.)	80	92
11	$(EtO)_2MeSiH$/ KF (2.0/ 2.0 eq.)	80	92
12	$(EtO)_2MeSiH$/ KF (2.0/ 2.5 eq.)	80	92
13	DME/DCE (3:1, 0.6 mL)	54	92
14	DME/DCE (3:1, 2.0 mL)	77	90
15	DME/DCE (2:1, 1.0 mL)	72	92
16	DME/DCE (4:1, 1.0 mL)	80	92
17	DME/DCE (1:2, 1.0 mL)	71	92
18	DME/DCE (1:3, 1.0 mL)	58	93

[a] Conditions: **1a** (0.20 mmol, 1.0 equiv), **2a** (0.30 mmol, 1.5 equiv), $NiI_2 \cdot xH_2O$ (10 mol%), **L*1** (15 mol%), $(EtO)_2MeSiH$ (0.24 mmol), KF (0.30 mmol), DME/DCE (3:1, 1.0 mL), rt, 20 h. [b] Yields were determined by ^1H NMR, using CH_2Br_2 as the internal standard. Ees were determined by HPLC.

Supplementary Table 7. Screening of reaction parameters for the synthesis of *N*-propargyl indoles[a]

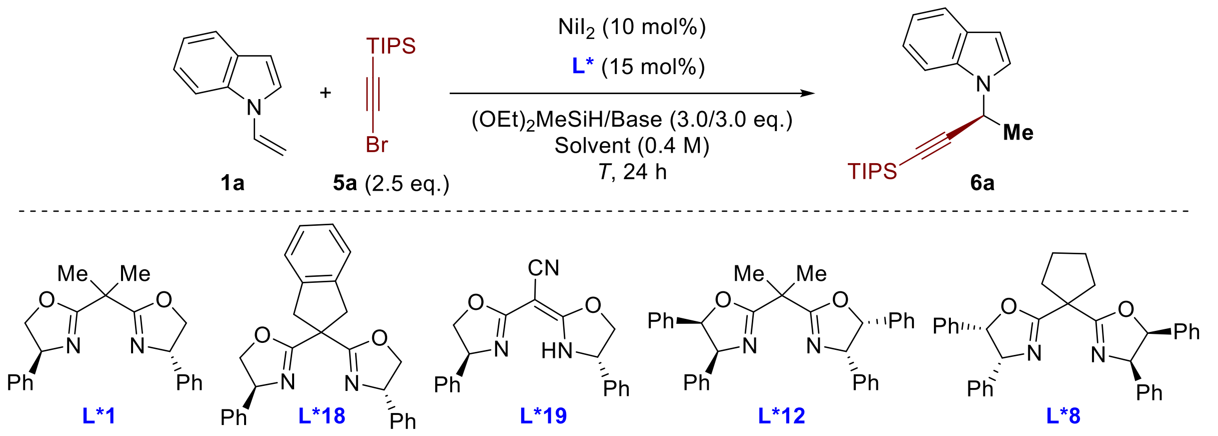

Entry	L	Base	Solvent	*T*	Yield (%)[b]	ee (%)[c]
1	**L*1**	KF	DME	rt	47	-64
2	**L*18**	KF	DME	rt	10	-73
3	**L*19**	KF	DME	rt	trace	N.D.
4	**L*12**	KF	DME	rt	76	-66
5	**L*8**	KF	DME	rt	60	83
6	**L*8**	CsF	DME	rt	19	70
7	**L*8**	Na_2CO_3	DME	rt	0	N.D.
8	**L*8**	K_2CO_3	DME	rt	0	N.D.
9	**L*8**	K_3PO_4	DME	rt	0	N.D.
10	**L*8**	KF	THF	rt	56	73
11	**L*8**	KF	DMF	rt	15	80
12	**L*8**	KF	MeCN	rt	51	82
13	**L*8**	KF	DCE	rt	0	N.D.
14	**L*8**	KF	DME	40 °C	42	75
15	**L*8**	KF	DME	0 °C (36 h)	91	90

[a] Conditions: All reactions were carried out with NiI_2 (10 mol%), ligand **L*** (15 mol%), **1a** (0.10 mmol), **5a** (0.25 mmol), $(OEt)_2MeSiH$ (0.30 mmol), KF (0.30 mmol) and solvent (0.25 mL) at the corresponding temperature (*T*) for 24 hours. [b] ee was determined by HPLC.

1.3 Synthesis of *N*-Vinylindole

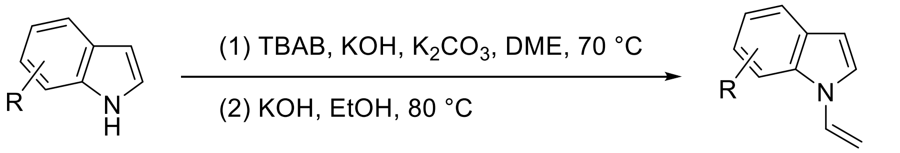

1a-1n

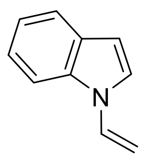

1a

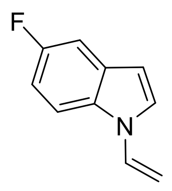

1b

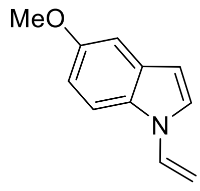

1c

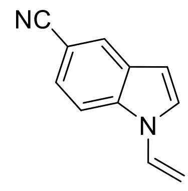

1d

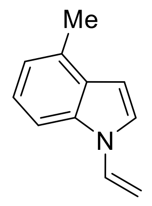

1e

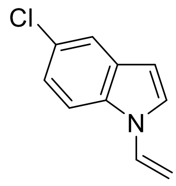

1f

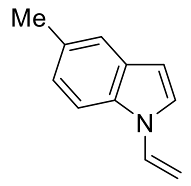

1g

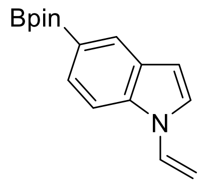

1h

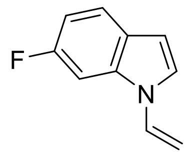

1i

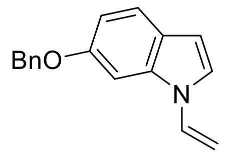

1j

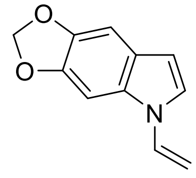

1k

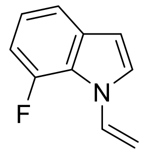

1l

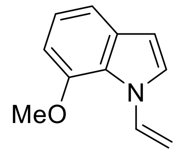

1m

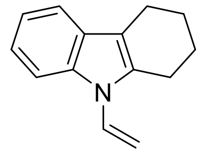

1n

In a round-bottom flask, substrate indole (1.0 equiv.), tetrabutylammonium bromide (TBAB) (0.1 equiv.), KOH (10.0 equiv.), K_2CO_3 (4.0 equiv.) and the solvent DCE (0.27 M) were added. Then, the mixture was stirred at 70 °C for 12 h. After the reaction completion monitored by TLC analysis, the solvent was evaporated under reduced pressure. The mixture of the residue, KOH (4.0 equiv.), and EtOH (0.20 M) were stirred in a preheated oil at 80 °C for 3 h. After the reaction completion monitored by TLC analysis, the reaction mixture was filtered and evaporated under vacuum. The residue was purified by a silica-gel column chromatography using petroleum ether/ethyl acetate as an eluent to obtain the product **1a-1n,** all spectral data of **1a**[1], **1c**[1], **1e**[1], **1i**[1], **1b**[2] were in accordance with the literature.

1-Vinyl-1*H*-indole-5-carbonitrile

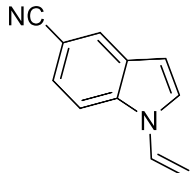

1d

Yellow solid, 68% yield, flash column chromatography (SiO_2, 5:1 PE/EtOAc).

^{1}H NMR (400 MHz, CDCl$_3$) δ 7.92 (s, 1H), 7.54-7.49 (m, 1H), 7.49-7.42 (m, 2H), 7.25 - 7.09 (m, 1H), 6.67 (s, 1H), 5.28 (d, *J* = 15.6 Hz, 1H), 4.92 (d, *J* = 9.2 Hz, 1H).

^{13}C NMR (101 MHz, CDCl$_3$) δ 136.7, 128.8, 128.7, 126.3, 125.6, 125.5, 120.2, 110.2, 105.2, 103.7, 99.3.

HRMS (ESI) m/z: $[M + H]^+$ Calcd for $C_{11}H_9N_2^+$ 169.0760; Found 169.0763.

5-Chloro-1-vinyl-1*H*-indole

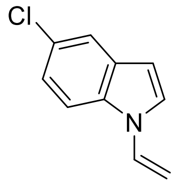

1f

Yellow solid, 62% yield, flash column chromatography (SiO_2, 100:0 PE/EtOAc).

^{1}H NMR (500 MHz, Acetone-*d$_6$*) δ 7.77 (d, *J* = 3.5 Hz, 1H), 7.66 – 7.62 (m, 2H), 7.44 (dd, *J* = 15.5, 9.0 Hz, 1H), 7.23 (d, *J* = 9.0, 1H), 6.65 (d, *J* = 3.5 Hz, 1H), 5.39 (d, *J* = 15.5 Hz, 1H), 4.82 (d, *J* = 9.0 Hz, 1H).

^{13}C NMR (126 MHz, Acetone-*d$_6$*) δ 134.1, 130.4, 129.7, 125.9, 125.3, 122.6, 120.3, 111.2, 104.4, 96.9.

HRMS (ESI) m/z: $[M + H]^+$ Calcd for $C_{10}H_9ClN^+$ 178.0418; Found 178.0416.

5-Methyl-1-vinyl-1*H*-indole

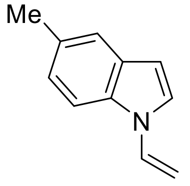

1g

White solid, 59% yield, flash column chromatography (SiO_2, 100:0 PE/EtOAc).

^{1}H NMR (400 MHz, Acetone-d_6) δ 7.63 (d, J = 3.2 Hz, 1H), 7.51 (d, J = 8.4 Hz, 1H), 7.44 – 7.37 (m, 2H), 7.07 (dd, J = 8.4, 1.0 Hz, 1H), 6.56 (d, J = 3.2 Hz, 1H), 5.29 (d, J = 15.6, 1H), 4.72 (d, J = 9.2, 1H), 2.39 (s, 3H).

^{13}C NMR (101 MHz, Acetone-d_6) δ 134.1, 130.0, 129.7, 129.6, 124.1, 123.6, 120.7, 109.4, 104.4, 95.1, 28.4.

HRMS (ESI) m/z: $[M + H]^+$ Calcd for $C_{11}H_{12}N^+$ 158.0964; Found 158.0967.

5-(4,4,5,5-Tetramethyl-1,3,2-dioxaborolan-2-yl)-1-vinyl-1*H*-indole

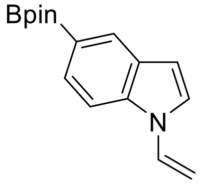

1h

White solid, 57% yield, flash column chromatography (SiO_2, 50:1 PE/EtOAc).

^{1}H NMR (400 MHz, Acetone-d_6) δ 8.05 (s, 1H), 7.72 (d, J = 3.2 Hz, 1H), 7.63 (s, 2H), 7.49 (dd, J = 15.6, 9.2 Hz, 1H), 6.71 (d, J = 3.2 Hz, 1H), 5.37 (d, J = 15.6 Hz, 1H), 4.80 (d, J = 9.2 Hz, 1H), 1.34 (s, 12H).

^{13}C NMR (101 MHz,Acetone-d_6) δ 137.5, 129.8, 128.9, 128.7, 128.6, 123.8, 109.0, 105.3, 96.2, 83.3, 24.4.

HRMS (ESI) m/z: $[M + H]^+$ Calcd for $C_{16}H_{21}BNO_2^+$ 269.1696; Found 269.1696.

6-(Benzyloxy)-1-vinyl-1*H*-indole

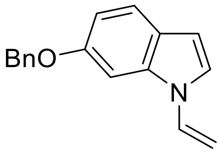

1j

White solid, 73% yield, flash column chromatography (SiO_2, 100:1 PE/EtOAc).

^{1}H NMR (400 MHz, Acetone-d_6) δ 7.57 – 7.46 (m, 4H), 7.44 – 7.31 (m, 5H), 6.90 – 6.86 (m, 1H), 6.58 – 6.56 (m, 1H), 5.28 (d, J = 15.6 Hz, 1H), 5.18 (s, 2H), 4.72 (d, J = 9.2 Hz, 1H).

^{13}C NMR (101 MHz, Acetone-d_6) δ 156.3, 137.8, 136.5, 129.9, 128.4, 127.7, 127.7, 123.4, 122.4, 121.5, 111.1, 104.9, 95.3, 94.9, 70.1.

HRMS (ESI) m/z: $[M + H]^+$ Calcd for $C_{17}H_{16}NO^+$ 250.1226; Found 250.1224.

5-Vinyl-5*H*-[1,3]dioxolo[4,5-f]indole

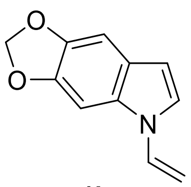

1k

White solid, 45% yield, flash column chromatography (SiO_2, 100:1 PE/EtOAc).

^{1}H NMR (400 MHz, Acetone-*d$_6$*) δ 7.53 (d, *J* = 3.2 Hz, 1H), 7.35 (dd, *J* = 15.6, 9.2 Hz, 1H), 7.18 (s, 1H), 6.99 (s, 1H), 6.51 (d, *J* = 3.2 Hz, 1H), 5.96 (s, 2H), 5.27 (d, *J* = 15.6, 1H), 4.71 (d, *J* = 9.2, 1H).

^{13}C NMR (101 MHz, Acetone-*d$_6$*) δ 145.5, 143.6, 130.8, 130.1, 123.0, 122.0, 105.1, 100.8, 99.3, 95.4, 91.0.

HRMS (ESI) m/z: $[M + H]^+$ Calcd for $C_{11}H_{10}NO_2^+$ 188.0706; Found 188.0708.

7-Fluoro-1-vinyl-1*H*-indole

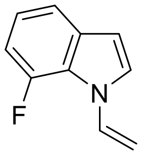

1l

Colorless oil, 60% yield, flash column chromatography (SiO_2, 100:0 PE/EtOAc).

^{1}H NMR (400 MHz, $CDCl_3$) δ 7.63 (dd, *J* = 15.6, 9.2 Hz, 1H), 7.49 (d, *J* = 3.2 Hz, 1H), 7.38 – 7.36 (m, 1H), 7.06 – 7.01 (m, 1H), 6.98 – 6.92 (m, 1H), 6.66-6.62 (m, 1H), 5.13 (d, *J* = 15.6, 1H), 4.74 (d, *J* = 9.2 Hz, 1H).

^{13}C NMR (101 MHz, $CDCl_3$) δ 150.4 (d, J_{C-F} = 244.4 Hz), 132.6 (d, J_{C-F} = 5.1 Hz), 132.0 (d, J_{C-F} = 10.5 Hz), 123.7, 123.1 (d, J_{C-F} = 8.7 Hz), 120.7 (d, *J* = 6.8 Hz), 116.9 (d, J_{C-F} = 3.5 Hz), 108.8, 105.61, 96.75.

^{19}F NMR (376 MHz, $CDCl_3$) δ -133.3.

HRMS (ESI) m/z: $[M + H]^+$ Calcd for $C_{10}H_9FN^+$ 162.0714; Found 162.0718.

7-Methoxy-1-vinyl-1*H*-indole

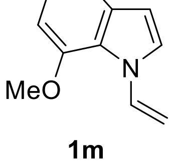

1m

White solid, 53% yield, flash column chromatography (SiO_2, 100:0 PE/EtOAc).

^{1}H NMR (400 MHz, CDCl$_3$) δ 8.09 (dd, J = 16.0, 9.2 Hz, 1H), 7.68 (d, J = 3.2 Hz, 1H), 7.18 – 7.16 (m, 1H), 7.00 (t, J = 7.6 Hz, 1H), 6.80 – 6.78 (m, 1H), 6.60 (d, J = 3.2 Hz, 1H), 6.19 (d, J = 16.0, 1H), 4.61 (d, J = 9.2, 1H), 3.96 (s, 3H).
^{13}C NMR (101 MHz, CDCl$_3$) δ 147.9, 133.4, 131.3, 124.7, 123.0, 121.0, 113.8, 105.3, 104.4, 94.6, 55.0.
HRMS (ESI) m/z: $[M + H]^+$ Calcd for $C_{11}H_{12}NO^+$ 174.0913; Found 174.0910.

9-Vinyl-2,3,4,9-tetrahydro-1*H*-carbazole

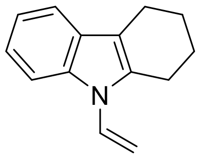

1n

Colorless oil, 21% yield, flash column chromatography (SiO_2, 100:0 PE/EtOAc).
^{1}H NMR (400 MHz, Acetone-*d*$_6$) δ 7.61 (d, J = 8.0 Hz, 1H), 7.42 (d, J = 7.6 Hz, 1H), 7.20 – 7.07 (m, 3H), 5.27 (d, J = 16.0, 1H), 4.87 (d, J = 9.6 Hz, 1H), 2.81 – 2.78 (m, 2H), 2.68 – 2.64 (m, 2H), 1.94 – 1.80 (m, 4H).
^{13}C NMR (101 MHz, Acetone-*d*$_6$) δ 135.6, 134.9, 129.9, 128.8, 121.8, 120.2, 117.7, 112.1, 110.6, 99.0, 23.2, 23.1, 22.5, 20.7.
HRMS (ESI) m/z: $[M + H]^+$ Calcd for $C_{14}H_{16}N^+$ 198.1277; Found 198.1283.

3-(2-((*tert*-Butyldimethylsilyl)oxy)ethyl)-1-vinyl-1*H*-indole

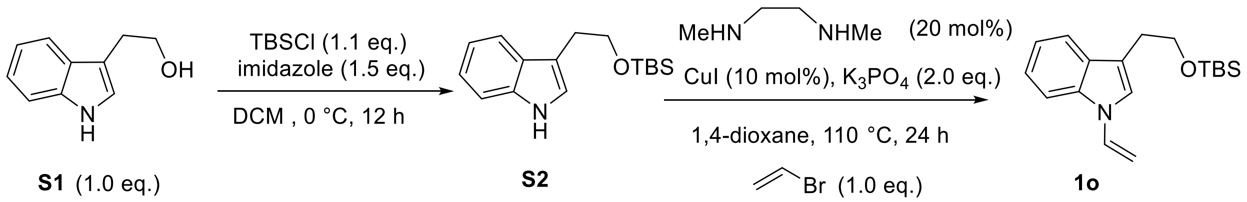

To a stirred solution of 2-(1*H*-indol-3-yl)ethanol (5.02 g, 31.00 mmol, 1.0 equiv.) and imidazole (3.15 g, 46.50 mmol, 1.5 equiv) in CH_2Cl_2 (0.31 M) at 0 °C was added *t*-butyl-dimethylsilyl chloride (5.01 g, 39.00 mmol, 1.1 equiv.). The resulting solution was stirred at room temperature overnight. Once completion of the reaction, the mixture was diluted with water (125.0 mL) and extracted with DCM (3 times). The combined organic phases were washed with brine (100.0 mL), then dried (Na_2SO_4) and concentrated. The residue was subjected to flash chromatography on silica gel (EtOAc/PE = 1/10) to afford **S2**[3] (94% yield). Then, CuI (10 mol%), **S2** (18.00 mmol, 1.2 equiv.) and K_3PO_4 (15.00 mmol, 2.0 equiv.) were

added to pre-dried a flask with a Teflon-lined septum. The flask was then evacuated and backfilled with N_2 (3 cycles). Vinyl bromide (15.00 mmol, 1.0 equiv), *N,N*-dimethylethane-1,2-diamine (20 mol%), and 1,4-dioxane (0.50 M) were added by syringe at room temperature. The flaske was then sealed and the reaction mixture was stirred at 110 °C for 24 h. The reaction was cooled to room temperature. Ethyl acetate (10.0 mL) was added and stirred for 10 min. The deposition was separated and washed with ethyl acetate (20.0 mL × 3). The organic phase was combined. The solvent was removed under vacuum. The crude product was purified by colum chromatography on silica gel to give corresponding *N*-alkenyl indol product **1o**.[4]

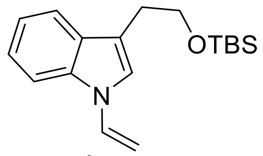

1o

Yellow oil, 75% yield, flash column chromatography (SiO_2, 10:1 PE/EtOAc).

^{1}H NMR (500 MHz, Acetone-*d*$_6$) δ 7.54 – 7.50 (m, 2H), 7.36 (dd, *J* = 15.5, 9.0 Hz, 1H), 7.20 – 7.14 (m, 1H), 7.07 (dd, *J* = 7.0, 1.0 Hz, 1H), 5.17 (d, *J* = 15.6 Hz, 1H), 4.63 (d, *J* = 9.6 Hz, 1H), 3.87 (t, *J* = 7.0 Hz, 2H), 2.92 (t, *J* = 7.5 Hz, 2H), 0.84 (s, 9H), -0.03 (s, 6H).

^{13}C NMR (126 MHz, Acetone-*d*$_6$) δ 136.6, 130.4, 129.9, 123.4, 122.1, 121.0, 119.9, 116.7, 110.3, 95.1, 64.0, 29.4, 26.2, 18.7, -5.3.

HRMS (ESI) m/z: $[M + H]^+$ Calcd for $C_{18}H_{27}NOSi^+$ 301.1935; Found 301.1932.

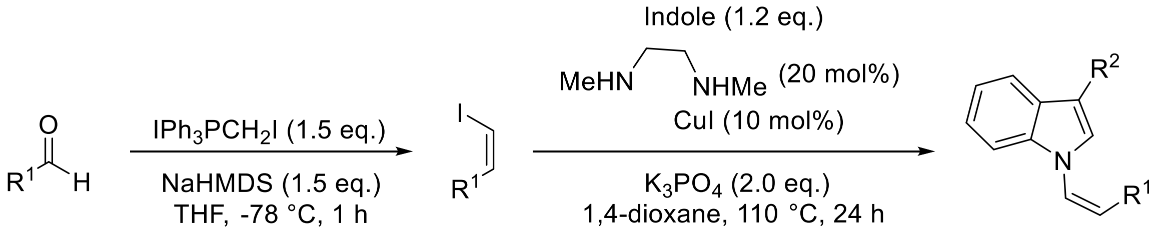

S3 **S4** **1p-1q, 1s-1u**

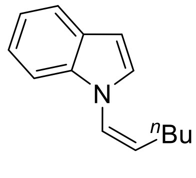

1p

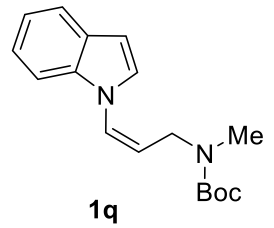

1q

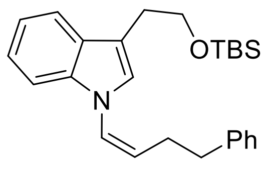

1s

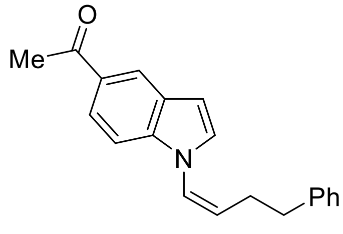

1t

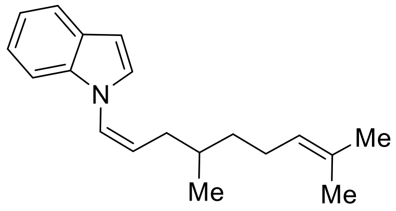

1u

Following a reported procedure,[5] under nitrogen atmosphere, iodomethyl triphenylphosphonium iodide (10.32 g, 18.50 mmol, 1.5 equiv) was suspended in THF (26.0 mL). At room temperature, sodium bis(trimethylsilyl)amide (2.00 M in THF, 9.2 mL, 18.50 mmol, 1.5 equiv) was added dropwise over 5 min. The reaction was stirred for 30 min then cooled to -78 °C. A solution of **S3** (12.32 mmol, 1.0 equiv) in THF (26.0 mL) was added dropwise over 15 min. The reaction was then stirred at -78 °C for 1 h then quenched with Saturated ammonium chloride solution(10.0 mL) then warmed to RT. The crude was diluted with Et_2O (20.0 mL) and saturated salt solution (20.0 mL). The layers were separated, the aqueous layers were extracted with Et_2O (5 x 30.0 mL). The organic layer was washed with sat. $NaHCO_3$ (2 x 20.0 mL), then brine (20.0 mL). The organic layers were combined and concentrated under reduced pressure. The compound was purified by column chromatography: SiO_2 using pentane affording **S3.** Following a reported procedure,[4] CuI (10 mol%), **S4** (1.2 equiv.) and K_3PO_4 (2.0 equiv.) were added to pre-dried a flask with a Teflon-lined septum. The flask was then evacuated and backfilled with N_2 (3 cycles). Indole (1.0 equiv.), *N,N*-dimethylethane-1,2-diamine (20 mol%), and 1,4-dioxane (0.50 M) were added by syringe at room temperature. The flask was then sealed and the reaction mixture was stirred at 110 °C for 24 h. The reaction was cooled to room temperature. Ethyl acetate (10.0 mL) was added and stirred for 10 min. The deposition was separated and washed with ethyl

acetate (20.0 mL x3). The organic phase was combined. The solvent was removed under vacuum. The crude product was purified by colum chromatography on silica gel to give corresponding *N*-alkenyl indol products **1p, 1q, 1s, 1t, 1u.**

1-(*Hex*-1-en-1-yl)-1*H*-indole

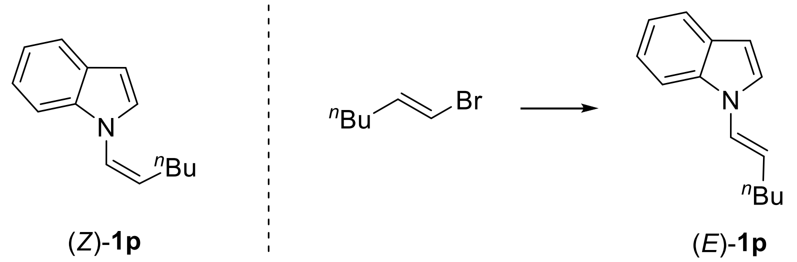

(*Z*)-**1p**: Colorless oil, 63% yield (*Z/E* = 14:1), flash column chromatography (SiO_2, 10:1 PE/EtOAc).

^{1}H NMR (500 MHz, $CDCl_3$) δ 7.57-7.52 (m, 1H), 7.27-7.22 (m, 1H), 7.20 -7.12 (m, 2H), 7.09-7.04 (m, 1H), 6.71-6.65 (m, 1H), 6.50 (d, *J* = 6.0 Hz, 1H), 5.41 – 5.28 (m, 1H), 2.24 - 2.15 (m, 2H), 1.38 – 1.31 (m, 2H), 1.28 -1.15 (m, 2H), 0.83 -0.76 (m, 3H).

^{13}C NMR (126 MHz, $CDCl_3$) δ 136.3, 128.1, 127.6, 124.1, 123.2, 122.0, 120.8, 120.2, 110.0, 102.7, 31.8, 26.9, 22.3, 13.9.

HRMS (ESI) m/z: $[M + H]^+$ Calcd for $C_{14}H_{18}N^+$ 200.1434; Found 200.1437.

(*E*)-**1p**: Following the same procedure as the synthesis of (*Z*)-**1p,** (*E*)-1-bromohex-1-ene as the alkenyl reagent; colorless oil, 75% yield (*E/Z* = >99:1), flash column chromatography (SiO_2, PE).

^{1}H NMR (500 MHz, $CDCl_3$) δ 7.50 (d, *J* = 7.6 Hz, 1H), 7.31 (d, *J* = 8.0 Hz, 1H), 7.19 (d, *J* = 3.2 Hz, 1H), 7.16 - 7.09 (m, 1H), 7.08 – 6.97 (m, 1H), 6.89 - 6.73 (m, 1H), 6.45 (d, *J* = 3.2 Hz, 1H), 5.67 - 5.51 (m, 1H), 2.12 - 2.02 (m, 2H), 1.40 – 1.24 (m, 4H), 0.83 (t, *J* = 7.2 Hz, 3H).

^{13}C NMR (126 MHz, $CDCl_3$) δ 135.3, 128.8, 124.2, 123.8, 122.2, 121.0, 120.2, 116.0, 109.5, 103.7, 32.0, 29.8, 22.1, 13.9.

***tert*-Butyl -(3-(1*H*-indol-1-yl)allyl)(methyl)carbamate**

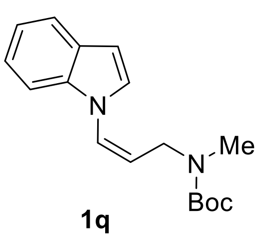

1q

Yellow oil, 31% yield (Z/E = 30:1), flash column chromatography (SiO_2, 10:1 PE/EtOAc).

^{1}H NMR (500 MHz, $CDCl_3$) δ 7.63 (d, J = 8.0 Hz, 1H), 7.33 (d, J = 8.0 Hz, 1H), 7.27 – 7.22 (m, 1H), 7.17 (t, J = 7.5 Hz, 2H), 6.96 (d, J = 8.5 Hz, 1H), 6.61 (d, J = 3.0 Hz, 1H), 5.44 – 5.38 (m, 1H), 4.18 – 4.01 (m, 2H), 2.77 (s, 3H), 1.43 (s, 9H).

^{13}C NMR (126 MHz, $CDCl_3$) δ 155.6, 136.3, 128.3, 127.5, 125.5, 122.4, 120.9, 120.6, 119.0, 109.9, 103.7, 79.8, 45.3, 33.8, 28.4.

HRMS (ESI) m/z: $[M + H]^+$ Calcd for $C_{17}H_{22}N_2NaO_2^+$ 309.1573; Found 309.1574.

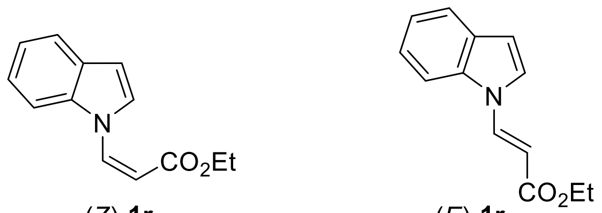

(*Z*)-**1r** (*E*)-**1r**

Following a reported procedure,[6] to give corresponding products (*Z*)- and (*E*)-**1r** and all spectral datas were in accordance with the literature.

3-(2-((*tert*-Butyldimethylsilyl)oxy)ethyl)-1-(4-phenylbut-1-en-1-yl)-1*H*-indole

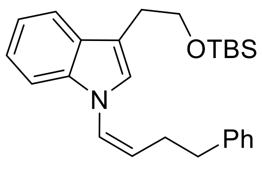

1s

White solid, 71% yield (Z/E = 10:1), flash column chromatography (SiO_2, 10:1 PE/EtOAc).

^{1}H NMR (500 MHz, $CDCl_3$) δ 7.55 - 7.51 (m, 1H), 7.24 - 7.06 (m, 8H), 6.89 (d, J = 6.5 Hz, 1H), 6.69 (t, J = 6.5 Hz, 1H), 5.29 (m, 1H), 3.84 – 3.75 (m, 2H), 2.91 (t, J = 7.0 Hz, 2H), 2.69 (t, J = 7.0 Hz, 2H), 2.54 (t, J = 7.0 Hz, 2H), 0.83 (s, 9H), -0.04 (s, 6H).

^{13}C NMR (126 MHz, $CDCl_3$) δ 141.1, 136.5, 128.5, 128.4, 128.0, 126.1, 125.3, 123.8, 122.1, 121.3, 119.8, 119.0, 113.7, 110.0, 63.7, 35.8, 29.1, 29.0, 26.0, 18.4, -5.3.

HRMS (ESI) m/z: $[M + H]^+$ Calcd for $C_{26}H_{36}NOSi^+$ 406.2561; Found 406.2561.

1-(1-(4-Phenylbut-1-en-1-yl)-1*H*-indol-5-yl)ethan-1-one

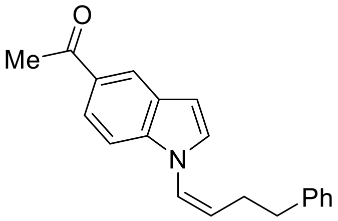

1t

White solid, 43% yield (*Z/E* = 16:1), flash column chromatography (SiO_2, 20:1 PE/EtOAc).

^{1}H NMR (500 MHz, Acetone-d_6) δ 8.32 (s, 1H), 7.87 (d, *J* = 8.5 Hz, 1H), 7.39 – 7.34 (m, 2H), 7.26 – 7.23 (m, 2H), 7.19 – 7.16 (m, 3H), 6.94 (d, *J* = 8.5 Hz, 1H), 6.72 (d, *J* = 3.0 Hz, 1H), 5.63 (q, *J* = 7.5 Hz, 1H), 2.77 (t, *J* = 7.5 Hz, 2H), 2.60 (s, 3H), 2.58-2.52 (m, 2H).

^{13}C NMR (126 MHz, Acetone-d_6) δ 196.6, 141.3, 138.6, 130.5, 129.4, 128.4, 128.3, 127.9, 126.0, 124.7, 123.6, 122.7, 122.1, 110.0, 104.3, 35.1, 29.0, 25.8.

HRMS (ESI) m/z: $[M + H]^+$ Calcd for $C_{20}H_{20}NO^+$ 290.1539; Found 290.1541.

1-(4,8-Dimethylnona-1,7-dien-1-yl)-1*H*-indole

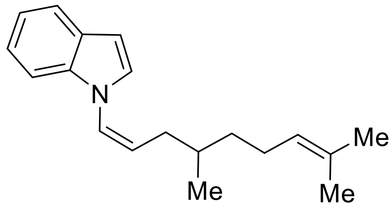

1u

Colorless oil, 54% yield (*Z/E* = 7:1), flash column chromatography (SiO_2, 50:1 PE/EtOAc).

^{1}H NMR (500 MHz, $CDCl_3$) δ 7.56 (d, *J* = 8.0 Hz, 1H), 7.25 (d, *J* = 8.0 Hz, 1H), 7.18 – 7.15 (m, 2H), 7.08 (d, *J* = 7.5 Hz, 1H), 6.76 (d, *J* = 8.5 Hz, 1H), 6.55 – 6.46 (m, 1H), 5.38 (q, *J* = 7.5 Hz, 1H), 4.99 (d, *J* = 7.5 Hz, 1H), 2.27 – 2.14 (m, 1H), 2.11 – 1.99 (m, 1H), 1.94 – 1.79 (m, 2H), 1.60 (s, 3H), 1.49 (s, 3H), 1.32 - 1.25 (m, 1H), 1.20 (s, 1H), 1.11 -1.07 (m, 1H), 0.83 (d, *J* = 7.0 Hz, 3H).

^{13}C NMR (126 MHz, $CDCl_3$) δ 136.4, 131.3, 128.1, 127.6, 124.5, 124.0, 123.0, 122.0, 120.8, 120.2, 110.1, 102.7, 36.6, 34.3, 32.9, 25.5, 19.5, 17.6.

HRMS (ESI) m/z: $[M + H]^+$ Calcd for $C_{19}H_{26}N^+$ 268.2060; Found 268.2060.

1.4 Preparation of Aryl Halides, Alkenyl Halides, and Alkynyl Bromides

Aryl halides and alkenyl halides were purchased from commercial sources (**2a-2t**) or prepared according to known literature procedures (**2y**[7], **2z-2aa**[8], **5a-5c**[9,10]).

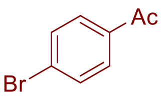

2a

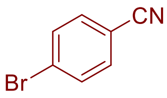

2b

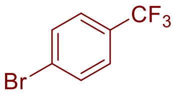

2c

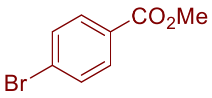

2d

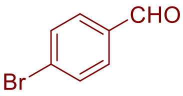

2e

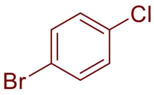

2f

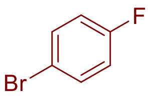

2g

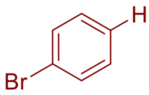

2h

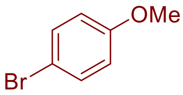

2i

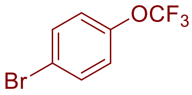

2j

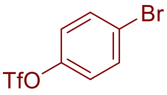

2k

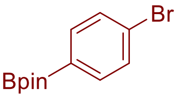

2l

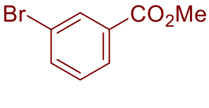

2m

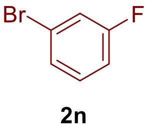

2n

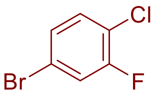

2o

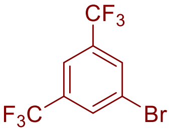

2p

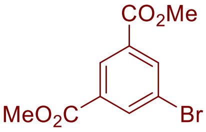

2q

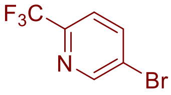

2r

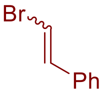

2s
(*E*/*Z* 1:1)

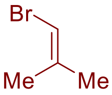

2t

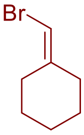

2u

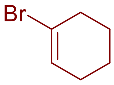

2v

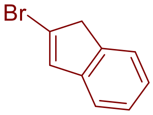

2w

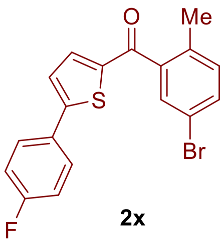

2x

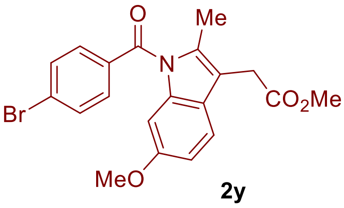

2y

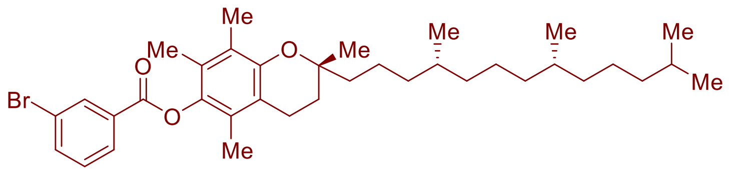

2z

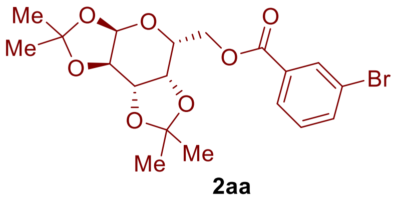

2aa

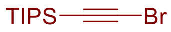

5a

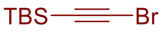

5b

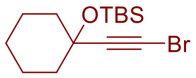

5c

(*R*)-3,5,7,8-Tetramethyl-3-((4*R*,8*R*)-4,8,12-trimethyltridecyl)chroman-6-yl 3-bromobenzoate

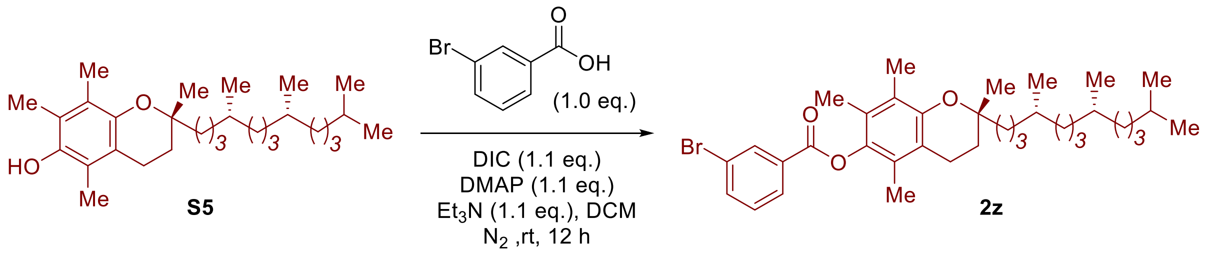

To a stirred solution of 3-iodobenzoic acid (4.96 g, 20.00 mmol, 1.0 equiv.) in dry DCM (60.0 mL) at 0 °C under N_2 atmosphere was added *N,N'*-diisopropylcarbodiimide (3.4 mL, 22.00 mmol, 1.1 equiv.) and DMAP (488.2 mg, 4.00 mmol, 0.2 equiv). After 10 minutes, Vitamin E **S5** (8.62 g, 20.00 mmol, 1.0 equiv) and Et_3N (3.1 mL, 22.00 mmol 1.1 equiv.) were added to it. The resulting reaction mixture was allowed to warm up to room temperature and stirring was continued for overnight. The solution was diluted with DCM and filtered through a plug of silica gel. The solvent was removed in vacuo. The crude product was purified by column chromatography on silica gel with 10:1 PE:EtOAc as eluent to obtain the desired product **2z** (9.88 g, 81%) as a yellow oil.

^{1}H NMR (600 MHz, $CDCl_3$) δ 8.30 (s, 1H), 8.09 (d, *J* = 7.8 Hz, 1H), 7.67 (d, *J* = 8.4 Hz, 1H), 7.31 (t, *J* = 7.8 Hz, 1H), 2.53 (t, *J* = 7.2 Hz, 2H), 2.04 (s, 3H), 1.96 (s, 3H), 1.92 (s, 3H), 1.78 – 1.66 (m, 2H), 1.44 – 1.03 (m, 23H), 0.82 - 0.74 (m, 13H).

^{13}C NMR (151 MHz, $CDCl_3$) δ 167.7, 163.8, 149.6, 140.5, 136.3, 133.0, 130.1, 128.7, 126.7, 125.0, 123.2, 122.7, 117.5, 75.1, 68.1, 39.4, 37.4, 37.3, 32.8, 30.4, 28.9, 28.0, 24.8, 24.4, 22.7, 22.6, 21.0, 20.6, 19.8, 19.7, 14.0, 13.0, 12.2, 11.8, 11.0.

HRMS (ESI) m/z: $[M + H]^+$ Calcd for $C_{36}H_{54}BrO_3^+$ 613.3251; Found 613.3246.

1.5 Ni-Catalyzed Enantioselective Synthesis of *N*-Alkyl Indoles

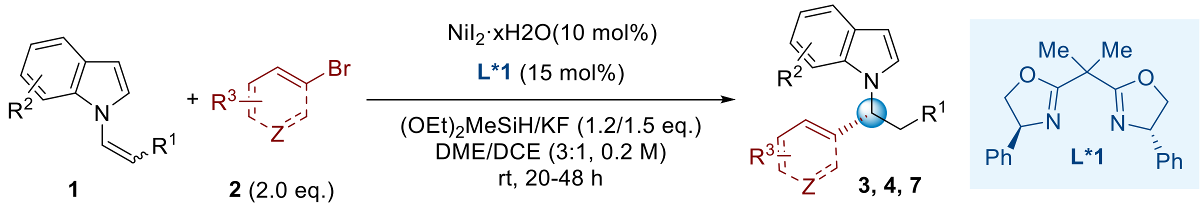

General Procedure 1 (GP1): To an oven-dried 8.0 mL Teflon-screw cap test tube containing a magnetic stir was charged with NiI_2•xH_2O (16.8 mg, 10 mol%) and ligand **L*1** (20.2 mg, 15 mol%) under a nitrogen (N_2) atmosphere using glove-box techniques. Subsequently, anhydrous DME (1.5 mL) was added, and the mixture was stirred for 15 minutes at room temperature. Then, KF (35.0 mg, 0.60 mmol, 1.5 equiv.), N-alkenyl indole **1** (0.40 mmol, 1.0 equiv), alryl/alkenyl bromide **2** (0.80 mmol, 2.0 equiv.), DCE (0.5 mL), and $(OEt)_2MeSiH$ (78.0 uL, 0.48 mmol, 1.2 equiv.) were sequentially added. Afterwards, the tube was sealed with airtight electrical tapes and removed from the glove box and stirred at room temperature for 20-48 hours at 650 rpm. After the reaction was completed, the reaction mixture was diluted with saturated NH_4Cl (aq., 1.0 mL) and EtOAc (5.0 mL). The aqueous phase was extracted with EtOAc (2 x 5.0 mL) and the combined organic phases were concentrated in vacuo. The crude mixture was purified by flash column chromatography on silica gel using a mixture of PE/EtOAc as eluent to obtain the desired product **3**, **4, 7**.

(*R*)-1-(4-(1-(1*H*-Indol-1-yl)ethyl)phenyl)ethan-1-one (3a)

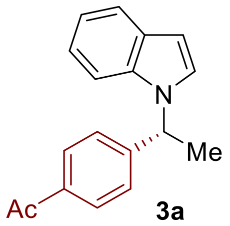

Prepared according to **GP1** with *N*-vinylindole **1a** (56.0 uL, 0.40 mmol, 1.0 equiv.) and **2a** (80.0 mg, 0.80 mmol, 2.0 equiv.). Flash column chromatography (SiO_2, 10:1 PE/EtOAc) afforded the desired product **3a** as a yellow oil (81.1 mg, 77%).

^{1}H NMR (500 MHz, $CDCl_3$) δ 7.76 (d, J = 8.0 Hz, 2H), 7.62 – 7.43 (m, 1H), 7.22 (d, J = 3.0 Hz, 1H), 7.12 – 6.86 (m, 5H), 6.51 (d, J = 3.0 Hz, 1H), 5.60 (q, J = 7.0 Hz, 1H), 2.44 (s, 3H), 1.84 (d, J = 7.0 Hz, 3H).

^{13}C NMR (126 MHz, CDCl$_3$) δ 197.4, 148.1, 136.3, 136.0, 128.8, 126.0, 124.6, 121.6, 121.0, 119.7, 109.9, 101.9, 54.6, 26.5, 21.5.

HRMS (ESI) m/z: $[M + H]^+$ Calcd for $C_{18}H_{18}NO^+$ 264.1383; Found 264.1385.

HPLC: 4:96 er determined by analytical HPLC, Daicel CHIRALCEL® OJ-H column, 25 °C, Hexane:*i*-PrOH = 80:20, 1.0 mL/min, 254 nm, t_{minor} = 31.3 min, t_{major} = 40.8 min.

$[\alpha]_D^{20}$ = +35.3 (*c* = 0.31, CHCl$_3$).

(*R*)-4-(1-(1*H*-Indol-1-yl)ethyl)benzonitrile (3b)

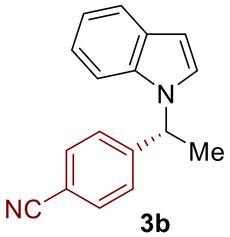

3b

Prepared according to **GP1** with *N*-vinylindole **1a** (56.0 uL, 0.40 mmol, 1.0 equiv.) and **2b** (145.6 mg, 0.80 mmol, 2.0 equiv.). Flash column chromatography (SiO$_2$, 15:1 PE/EtOAc) afforded the desired product **3b** as a colorless oil (56.4 mg, 57%).

^{1}H NMR (500 MHz, CDCl$_3$) δ 7.69 – 7.60 (m, 1H), 7.54 (d, *J* = 8.0 Hz, 2H), 7.30 (s, 1H), 7.19 – 6.94 (m, 5H), 6.61 (d, *J* = 3.0 Hz, 1H), 5.68 (q, *J* = 7.5 Hz, 1H), 1.93 (d, *J* = 6.5 Hz, 3H).

^{13}C NMR (126 MHz, CDCl$_3$) δ 148.2, 135.9, 132.6, 128.8, 126.5, 124.4, 121.8, 121.1, 119.9, 118.5, 111.4, 109.7, 102.2, 54.5, 21.5.

HRMS (ESI) m/z: $[M + H]^+$ Calcd for $C_{17}H_{15}N_2^+$ 247.1230; Found 247.1227.

HPLC: 4.5:95.5 er determined by analytical HPLC, Daicel CHIRALCEL® OJ-H column,, 25 °C, Hexane:*i*-PrOH = 80:20, 1.0 mL/min, 254 nm, t_{minor} = 27.0 min, t_{major} = 53.2 min.

$[\alpha]_D^{20}$ = +135.2 (*c* = 0.92, CHCl$_3$).

(*R*)-1-(1-(4-(Trifluoromethyl)phenyl)ethyl)-1*H*-indole (3c)

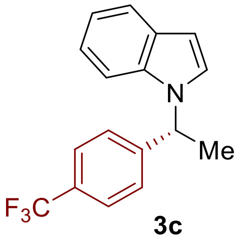

3c

Prepared according to **GP1** with *N*-vinylindole **1a** (56.0 uL, 0.40 mmol, 1.0 equiv.) and **2c** (112.0 µL, 0.80 mmol, 2.0 equiv.). Flash column chromatography (SiO_2, 20:1 PE/EtOAc) afforded the desired product **3c** as a yellow oil (70.6 mg, 61%).

^{1}H NMR (500 MHz, $CDCl_3$) δ 7.69 – 7.61 (m, 1H), 7.53 (d, J = 8.0 Hz, 2H), 7.31 (d, J = 3.0 Hz, 1H), 7.19 (d, J = 8.0 Hz, 2H), 7.17 – 7.04 (m, 3H), 6.61 (d, J = 3.0 Hz, 1H), 5.71 (q, J = 7.0 Hz, 1H), 1.95 (d, J = 7.0 Hz, 3H).

^{13}C NMR (126 MHz, $CDCl_3$) δ 146.9, 136.0, 129.7 (d, $J_{C\text{-}F}$ = 32.5 Hz), 128.9, 126.1, 125.7 (q, $J_{C\text{-}F}$ = 3.8 , 7.6 Hz), 124.5, 124.0 (d, $J_{C\text{-}F}$ = 272.5 Hz), 121.7, 121.1, 119.8, 109.8, 102.0, 54.5, 21.7.

^{19}F NMR (471 MHz, $CDCl_3$) δ -62.5.

HRMS (ESI) m/z: $[M + H]^+$ Calcd for $C_{17}H_{15}F_3N^+$ 290.1151; Found 290.1152.

HPLC: 6:94 er determined by analytical HPLC, Daicel CHIRALCEL® OJ-H column, 20 °C, Hexane:*i*-PrOH = 90:10, 1.0 mL/min, 254 nm, t_{minor} = 9.4 min, t_{major} = 12.2 min.

$[\alpha]_D^{20}$ = +67.4 (c = 0.42, $CHCl_3$).

Methyl (*R*)-4-(1-(1*H*-indol-1-yl)ethyl)benzoate (3d)

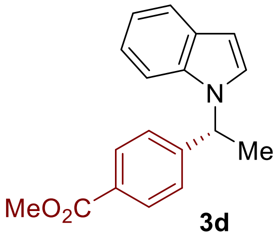

3d

Prepared according to **GP1** with *N*-vinylindole **1a** (56.0 uL, 0.40 mmol, 1.0 equiv.) and **2d** (172.0 mg, 0.80 mmol, 2.0 equiv.). Flash column chromatography (SiO_2, 15:1 PE/EtOAc) afforded the desired product **3d** as a yellow oil (68.2 mg, 61%).

^{1}H NMR (500 MHz, $CDCl_3$) δ 7.84 (d, J = 8.0 Hz, 2H), 7.55 (d, J = 7.0 Hz, 1H), 7.20 (s, 1H), 7.09 - 6.96 (m, 5H), 6.50 (d, J = 3.5 Hz, 1H), 5.58 (q, J = 7.0 Hz, 1H), 3.77 (s, 3H), 1.82 (d, J = 7.0 Hz, 3H) .

^{13}C NMR (126 MHz, $CDCl_3$) δ 166.6, 147.9, 136.0, 130.0, 129.3, 128.8, 125.8, 124.6, 121.6, 121.0, 119.7, 109.9, 101.8, 54.7, 52.0, 21.5.

HRMS (ESI) m/z: $[M + H]^+$ Calcd for $C_{18}H_{18}NO_2^+$ 280.1332; Found 280.1329.

HPLC: 4.5:95.5 er determined by analytical HPLC, Daicel CHIRALPAK® AD-H column, 20 °C, Hexane:*i*-PrOH = 80:20, 1.0 mL/min, 254 nm, t_{minor} = 17.5 min, t_{minor} = 22.0 min.

$[\alpha]_D^{20}$ = +38.2 (c = 1.3, $CHCl_3$).

(*R*)-4-(1-(1*H*-Indol-1-yl)ethyl)benzaldehyde (3e)

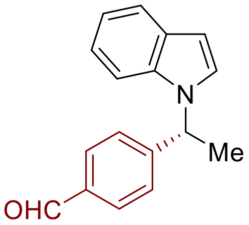

3e

Prepared according to **GP1** with *N*-vinylindole **1a** (56.0 uL, 0.40 mmol, 1.0 equiv.) and **2e** (148.1 mg, 0.80 mmol, 2.0 equiv.). Flash column chromatography (SiO_2, 15:1 PE/EtOAc) afforded the desired product **3e** as a pink oil (66.6 mg, 67%).

^{1}H NMR (500 MHz, $CDCl_3$) δ 9.85 (s, 1H), 7.69 (d, *J* = 8.0 Hz, 2H), 7.57 (d, *J* = 7.0 Hz, 1H), 7.24 (d, *J* = 3.0 Hz, 1H), 7.14 (d, *J* = 8.0 Hz, 2H), 7.09 – 6.97 (m, 3H), 6.53 (d, *J* = 3.0 Hz, 1H), 5.62 (q, *J* = 7.5 Hz, 1H), 1.86 (d, *J* = 7.0 Hz, 3H) .

^{13}C NMR (126 MHz, $CDCl_3$) δ 190.6, 148.7, 134.9, 134.6, 129.2, 127.8, 125.4, 123.5, 120.7, 120.0, 118.8, 108.8, 101.0, 53.7, 20.6 .

HRMS (ESI) m/z: $[M + H]^+$ Calcd for $C_{17}H_{16}NO^+$ 250.1226; Found 250.1225.

HPLC: 4:96 er determined by analytical HPLC, Daicel CHIRALCEL® OD-3 column, 25 °C, Hexane:*i*-PrOH = 90:10, 1.0 mL/min, 254 nm, t_{minor} = 31.7 min, t_{mjor} = 40.0 min.

$[\alpha]_D^{20}$ = +129.7 (*c* = 0.66, $CHCl_3$).

***(R)*-1-(1-(4-Chlorophenyl)ethyl)-1*H*-indole (3f)**

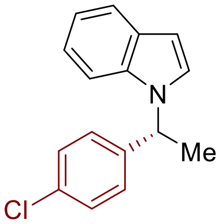

3f

Prepared according to **GP1** with *N*-vinylindole **1a** (56.0 uL, 0.40 mmol, 1.0 equiv.) and **2f** (152.8 mg, 0.80 mmol, 2.0 equiv.). Flash column chromatography (SiO_2, 30:1 PE/EtOAc) afforded the desired product **3f** as a yellow oil (68.7 mg, 67%).

^{1}H NMR (400 MHz, $CDCl_3$) δ 7.64 (d, *J* = 6.4 Hz, 1H), 7.26 (d, *J* = 3.2 Hz, 1H), 7.23 (d, *J* = 8.4 Hz, 2H), 7.18 – 7.06 (m, 3H), 7.02 (d, *J* = 8.4 Hz, 2H), 6.57 (d, *J* = 3.2 Hz, 1H), 5.62 (q, *J* = 7.2 Hz, 1H), 1.89 (d, *J* = 7.2 Hz, 3H).

^{13}C NMR (101 MHz, CDCl$_3$) δ 141.3, 135.9, 133.1, 128.8, 128.8, 127.2, 124.6, 121.6, 121.0, 119.7, 109.9, 101.7, 54.2, 21.7.

HRMS (ESI) m/z: $[M + H]^+$ Calcd for $C_{16}H_{15}ClN^+$ 256.0888; Found 256.0886.

HPLC: 7:93 er determined by analytical HPLC, Daicel CHIRALCEL® OD-H column, 25 °C, Hexane:*i*-PrOH = 90:10, 1.0 mL/min, 254 nm, t_{minor} = 8.4 min, t_{major} = 13.6 min.

$[\alpha]_D^{20}$ = +103.8 (*c* = 0.47, CHCl$_3$).

(*R*)-1-(1-(4-Fluorophenyl)ethyl)-1*H*-indole (3g)

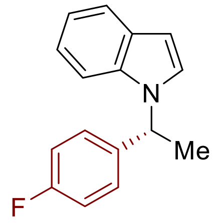

3g

Prepared according to **GP1** with *N*-vinylindole **1a** (56.0 uL, 0.40 mmol, 1.0 equiv.) and **2g** (172.0 mg, 0.80 mmol, 2.0 equiv.). Flash column chromatography (SiO$_2$, 4:1 PE/EtOAc) afforded the desired product **3g** as a colorless oil (52.2 mg, 55%).

^{1}H NMR (400 MHz, CDCl$_3$) δ 7.62 – 7.48 (m, 1H), 7.18 (d, *J* = 3.2 Hz, 1H), 7.15 – 7.11 (m, 1H), 7.08 – 6.95 (m, 4H), 6.87 (t, *J* = 8.8 Hz, 2H), 6.56 (s, 1H), 5.56 (q, *J* = 7.2 Hz, 1H), 1.81 (d, *J* = 7.2 Hz, 3H).

^{13}C NMR (101 MHz, CDCl$_3$) δ 162.0 (d, J_{C-F} = 246.4 Hz), 138.5 (d, J_{C-F} = 3.0 Hz), 135.9, 128.8, 127.5 (d, J_{C-F} = 7.1 Hz), 124.6, 121.5, 121.0, 119.6, 115.5 (d, J_{C-F} = 21.2 Hz), 109.9, 101.7, 54.2, 21.8.

^{19}F NMR (376 MHz, CDCl$_3$) δ -115.2

HRMS (ESI) m/z: $[M + H]^+$ Calcd for $C_{16}H_{15}FN^+$ 240.1183; Found 240.1182.

HPLC: 11:89 er determined by analytical HPLC, Daicel CHIRALCEL® OD-H column, 25 °C, Hexane:*i*-PrOH = 90:10, 1.0 mL/min, 254 nm, t_{minor} = 8.1 min, t_{mjor} = 12.1 min.

$[\alpha]_D^{20}$ = +58.3 (*c* = 0.55, CHCl$_3$).

(*R*)-1-(1-Phenylethyl)-1*H*-indole (3h)

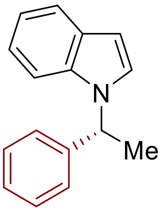

3h

Prepared according to **GP1** with *N*-vinylindole **1a** (56.0 uL, 0.40 mmol, 1.0 equiv.) and **2h** (84.7 uL, 0.80 mmol, 2.0 equiv.). Flash column chromatography (SiO_2, 49:1 PE/EtOAc) afforded the desired product **3h** as a white solid (40.2 mg, 52%).

^{1}H NMR (400 MHz, $CDCl_3$) δ 7.69 – 7.58 (m, 1H), 7.30 – 7.20 (m, 5H), 7.15 – 7.04 (m, 4H), 6.56 (d, *J* = 3.2 Hz, 1H), 5.65 (q, *J* = 7.2 Hz, 1H), 1.90 (d, *J* = 7.2 Hz, 3H).
^{13}C NMR (101 MHz, $CDCl_3$) δ 142.7, 136.1, 128.8, 128.7, 127.4, 125.9, 124.8, 121.4, 120.9, 119.5, 110.0, 101.5, 54.8, 21.7.
HRMS (ESI) m/z: $[M + H]^+$ Calcd for $C_{16}H_{16}N^+$ 222.1277; Found 222.1276.
HPLC: 9.5:90.5 er determined by analytical HPLC, Daicel CHIRALCEL® OD-3 column, 20 °C, Hexane:*i*-PrOH = 98:2, 1.0 mL/min, 280 nm, t_{minor} = 9.4 min, t_{major} = 14.8 min.
$[\alpha]_D^{25}$ = +75.2 (*c* = 0.8, $CHCl_3$).

(*R*)-1-(1-(4-Methoxyphenyl)ethyl)-1*H*-indole (3i)

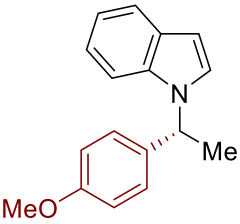

3i

Prepared according to **GP1** with *N*-vinylindole **1a** (56.0 uL, 0.40 mmol, 1.0 equiv.) and **2i** (76.0 ul, 0.80 mmol, 2.0 equiv.). Flash column chromatography (SiO_2, 20:1 PE/EtOAc) afforded the desired product **3i** as a colorless oil (64.2 mg, 64%,).
^{1}H NMR (500 MHz, $CDCl_3$) δ 7.63 (d, *J* = 7.5 Hz, 1H), 7.27 – 7.21 (m, 2H), 7.12 (t, *J* = 7.5 Hz, 1H), 7.10 - 7.05 (m, 3H), 6.80 (d, *J* = 8.0 Hz, 2H), 6.54 (d, *J* = 3.0 Hz, 1H), 5.62 (q, *J* = 7.0 Hz, 1H), 3.74 (s, 3H), 1.87 (d, *J* = 7.0 Hz, 3H).
^{13}C NMR (126 MHz, $CDCl_3$) δ 158.8, 136.0, 134.6, 128.8, 127.1, 124.8, 121.3, 120.9, 119.4, 114.0, 110.0, 101.3, 55.2, 54.2, 21.6.
HPLC: 22:78 er determined by analytical HPLC, Daicel CHIRALCEL® OD-H column, 20 °C, Hexane:*i*-PrOH = 90:10, 1.0 mL/min, 254 nm, t_{minor} = 8.5 min, t_{major} = 10.8 min.

HRMS (ESI) m/z: $[M + H]^+$ Calcd for $C_{17}H_{18}NO^+$ 252.1383; Found 252.1381.
$[\alpha]_D^{20}$ = +68.1 (c = 0.50, $CHCl_3$).

(*R*)-1-(1-(4-(Trifluoromethoxy)phenyl)ethyl)-1*H*-indole (3j)

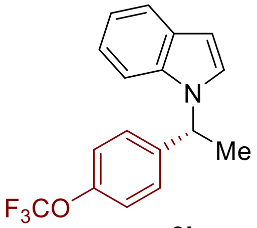

3j

Prepared according to **GP1** with *N*-vinylindole **1a** (56.0 uL, 0.40 mmol, 1.0 equiv.) and **2j** (118.9 mg, 0.80 mmol, 2.0 equiv.). Flash column chromatography (SiO_2, 20:1 PE/EtOAc) afforded the desired product **3j** as a yellow oil (73.9 mg, 61%).

^{1}H NMR (500 MHz, $CDCl_3$) δ 7.56 (d, J = 7.5 Hz, 1H), 7.19 (d, J = 3.5 Hz, 1H), 7.10 (d, J = 8.0 Hz, 1H), 7.10 - 6.95 (m, 6H), 6.50 (d, J = 3.5 Hz, 1H), 5.57 (q, J = 7.0 Hz, 1H), 1.81 (d, J = 7.0 Hz, 3H).
^{13}C NMR (126 MHz, $CDCl_3$) δ 148.4, 141.5, 136.0, 128.8, 127.3, 124.6, 121.6, 120.4, 121.1, 121.0 (d, J_{C-F} = 257.0 Hz), 119.7, 109.9, 101.9, 54.1, 21.7.
^{19}F NMR (471 MHz, $CDCl_3$) δ -57.9.
HRMS (ESI) m/z: $[M + H]^+$ Calcd for $C_{17}H_{15}F_3NO^+$ 306.1100; Found 306.1099.
HPLC: 6.5:93.5 er determined by analytical HPLC, Daicel CHIRALCEL® OD-H column, 20 °C, Hexane:*i*-PrOH = 90:10, 1.0 mL/min, 254 nm, t_{minor} = 7.4 min, t_{major} = 13.3 min.
$[\alpha]_D^{20}$ = +65.8 (c = 1.1, $CHCl_3$).

(*R*)-4-(1-(1*H*-Indol-1-yl)ethyl)phenyl trifluoromethanesulfonate (3k)

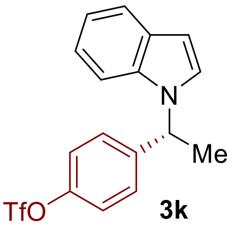

3k

Prepared according to **GP1** with *N*-vinylindole **1a** (56.0 uL, 0.40 mmol, 1.0 equiv.) and **2j** (133.7 ul, 0.80 mmol, 2.0 equiv.). Flash column chromatography (SiO_2, 20:1 Hexane/EtOAc) afforded the desired product **3k** as a yellow oil (61.0 mg, 40%).

^{1}H NMR (500 MHz, CDCl$_3$) δ 7.71 – 7.59 (m, 1H), 7.28 (d, *J* = 3.0 Hz, 1H), 7.22 – 7.05 (m, 7H), 6.60 (d, *J* = 3.0 Hz, 1H), 5.69 (q, *J* = 7.0 Hz, 1H), 1.94 (d, *J* = 7.0 Hz, 3H).

^{13}C NMR (126 MHz, CDCl$_3$) δ 148.6, 143.4, 135.9, 128.8, 127.7, 124.5, 121.8, 121.6, 121.1, 119.9, 118.7 (d , $J_{C\text{-}F}$ = 321.3 Hz), 109.8, 102.1, 54.1, 21.7.

^{19}F NMR (471 MHz, CDCl$_3$) δ -73.0 .

HRMS (ESI) m/z: $[M + H]^+$ Calcd for$C_{17}H_{15}F_3NO_3S^+$ 370.0719; Found 370.0716.

HPLC: 6.5:93.5 er determined by analytical HPLC, Daicel CHIRALCEL® OD-H column, 20 °C, Hexane:*i*-PrOH = 90:10, 1.0 mL/min, 254 nm, t_{minor} = 9.8 min, t_{major} = 19.3 min.

$[\alpha]_D^{20}$ = +64.3 (*c* = 0.63, CHCl$_3$).

(*R*)-1-(1-(4-(4,4,5,5-Tetramethyl-1,3,2-dioxaborolan-2-yl)phenyl)ethyl)-1*H*-indole (3l)

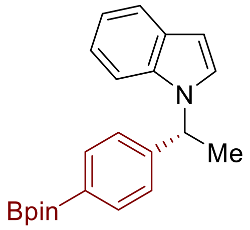

3l

Prepared according to **GP1** with *N*-vinylindole **1a** (56.0 uL, 0.40 mmol, 1.0 equiv.) and **2l** (289.5 mg, 0.80 mmol, 2.0 equiv.). Flash column chromatography (SiO$_2$, 20:1 PE/EtOAc) afforded the desired product **3l** as a pink solid (81.8 mg, 59%).

^{1}H NMR (500 MHz, Acetone-*d*$_6$) δ 7.67 (d, *J* = 8.0 Hz, 2H), 7.61 – 7.49 (m, 2H), 7.30 (d, *J* = 8.0 Hz, 1H), 7.22 (d, *J* = 7.5 Hz, 2H), 7.09 -6.95 (m, 2H), 6.54 (d, *J* = 3.0 Hz, 1H), 5.83 (q, *J* = 7.0 Hz, 1H), 1.93 (d, *J* = 7.0 Hz, 3H), 1.30 (s, 12H).

^{13}C NMR (126 MHz, Acetone-*d*$_6$) δ 147.4, 137.0, 135.7 (2C), 129.8, 126.1, 125.8, 121.9, 121.3, 120.0, 111.0, 102.0, 84.4, 55.5, 25.1, 21.8 .

HRMS (ESI) m/z: $[M + H]^+$ Calcd for $C_{22}H_{27}BNO_2^+$ 348.2129; Found 348.2124.

HPLC: 8:92 er determined by analytical HPLC, Daicel CHIRALCEL® OD-3 column, 20 °C, Hexane:*i*-PrOH = 95:5, 1.0 mL/min, 254 nm, t_{minor} = 10.3 min, t_{major} = 14.6 min.

$[\alpha]_D^{20}$ = +88.3 (*c* = 0.67, CHCl$_3$).

Methyl (*R*)-3-(1-(1*H*-indol-1-yl)ethyl)benzoate (3m)

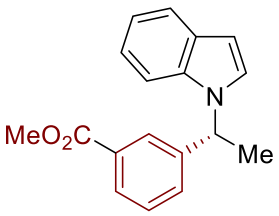

3m

Prepared according to **GP1** with *N*-vinylindole **1a** (56.0 uL, 0.40 mmol, 1.0 equiv.) and **2m** (172.0 mg, 0.80 mmol, 2.0 equiv.). Flash column chromatography (SiO_2, 15:1 Hexane/EtOAc) afforded the desired product **3m** as a colorless oil (84.7 mg, 78%).

^{1}H NMR (500 MHz, $CDCl_3$) δ 7.87 (s, 1H), 7.82 (d, *J* = 8.0 Hz, 1H), 7.55 (d, *J* = 7.5 Hz, 1H), 7.26 – 7.15 (m, 2H), 7.11 (d, *J* = 6.5 Hz, 2H), 7.01 (t, *J* = 8.5 Hz, 2H), 6.49 (d, *J* = 3.5 Hz, 1H), 5.60 (q, *J* = 7.0 Hz, 1H), 3.79 (s, 3H), 1.83 (d, *J* = 7.0 Hz, 3H) .
^{13}C NMR (126 MHz, $CDCl_3$) δ 166.8, 143.2, 136.0, 130.6, 130.4, 128.9, 128.8, 128.7, 127.1, 124.6, 121.6, 121.0, 119.6, 109.9, 101.8, 54.6, 52.1, 21.6.
HRMS (ESI) m/z: $[M + H]^+$ Calcd for $C_{18}H_{18}NO_2^+$ 280.1332; Found 280.1329.
HPLC: 4:96 er determined by analytical HPLC, Daicel CHIRALCEL® OJ-H column, 20 °C, Hexane:*i*-PrOH = 80:20, 1.0 mL/min, 254 nm, t_{minor} = 30.9 min, t_{major} = 54.0 min.
$[\alpha]_D^{20}$ = +62.6 (*c* = 0.93, $CHCl_3$).

(*R*)-1-(1-(3-Fluorophenyl)ethyl)-1*H*-indole (3n)

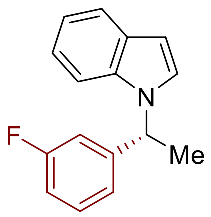

3n

Prepared according to **GP1** with *N*-vinylindole **1a** (56.0 uL, 0.40 mmol, 1.0 equiv.) and **2n** (88.0 ul, 0.80 mmol, 2.0 equiv.). Flash column chromatography (SiO_2, 20:1 PE/EtOAc) afforded the desired product **3n** as a yellow oil (69.5 mg, 73%).

^{1}H NMR (500 MHz, $CDCl_3$) δ 7.64 (d, *J* = 7.0 Hz, 1H), 7.29 (d, *J* = 3.0 Hz, 1H), 7.25 – 7.18 (m, 2H), 7.16 – 7.06 (m, 2H), 7.00 – 6.85 (m, 2H), 6.84 -6.74 (m, 1H), 6.58 (d, *J* = 3.0 Hz, 1H), 5.65 (q, *J* = 7.0 Hz, 1H), 1.91 (d, *J* = 7.0 Hz, 3H).

^{13}**C NMR (126 MHz, CDCl$_3$)** δ 163.1 (d, $J_{C\text{-}F}$= 246.7), 145.5 (d, $J_{C\text{-}F}$= 6.7 Hz), 136.0, 130.2 (d, $J_{C\text{-}F}$=8.2 Hz), 128.8, 124.6, 121.6, 121.5 (d, $J_{C\text{-}F}$= 4.8 Hz), 121.0, 119.7, 114.4 (d, $J_{C\text{-}F}$ =21.2 Hz), 112.9 (d, $J_{C\text{-}F}$=29.2 Hz), 110.0, 101.8, 54.4, 21.7.
^{19}**F NMR (471 MHz, CDCl$_3$)** δ -112.4 .
HRMS (ESI) m/z: $[M + H]^+$ Calcd for $C_{16}H_{15}FN^+$ 240.1183; Found 240.1185.
HPLC: 6:94 er determined by analytical HPLC, Daicel CHIRALCEL® OJ-H column, 20 °C, Hexane:*i*-PrOH = 90:10, 1.0 mL/min, 254 nm, t_{minor} = 14.3 min, t_{major} = 15.4 min.
$[\alpha]_D^{20}$ = +76.7 (*c* = 0.32, CHCl$_3$).

(*R*)-1-(1-(4-Chloro-3-fluorophenyl)ethyl)-1*H*-indole (3o)

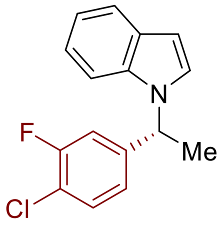

3o

Prepared according to **GP1** with *N*-vinylindole **1a** (56.0 uL, 0.40 mmol, 1.0 equiv.) and **2o** (98.0 mg, 0.80 mmol, 2.0 equiv.). Flash column chromatography (SiO$_2$, 20:1 PE/EtOAc) afforded the desired product **3o** as a yellow oil (71.3 mg, 64%).

^{1}**H NMR (500 MHz, CDCl$_3$)** δ 7.56 (d, *J* = 7.5 Hz, 1H), 7.26 - 7.15 (m, 2H), 7.11 – 6.93 (m, 3H), 6.76 (d, *J* = 10.0 Hz, 1H), 6.72 (d, *J* = 8.5 Hz, 1H), 6.50 (d, *J* = 3.0 Hz, 1H), 5.51 (q, *J* = 7.0 Hz, 1H), 1.79 (d, *J* = 7.0 Hz, 3H).
^{13}**C NMR (126 MHz, CDCl$_3$)** δ 158.2 (d, $J_{C\text{-}F}$= 250.1 Hz), 143.9 (d, $J_{C\text{-}F}$= 5.0 Hz), 135.9, 130.8, 128.9, 124.4, 122.2 ($J_{C\text{-}F}$= 3.8 Hz), 121.8, 121.1, 119.87 (d, $J_{C\text{-}F}$= 17.6), 119.86, 114.2 (d, $J_{C\text{-}F}$ = 21.8 Hz) , 109.8, 102.1, 54.0, 21.5.
^{19}**F NMR (471 MHz, CDCl$_3$)** δ -114.4 .
HRMS (ESI) m/z: $[M + H]^+$ Calcd for $C_{16}H_{14}ClFN^+$ 274.0793; Found 274.0798 .
HPLC: 5:95 er determined by analytical HPLC, Daicel CHIRALCEL® OD-3 column, 20 °C, Hexane:*i*-PrOH = 90:10, 1.0 mL/min, 254 nm, t_{minor} = 10.0 min, t_{major} = 19.7 min.
$[\alpha]_D^{20}$ = +84.6 (*c* = 0.35, CHCl$_3$).

(*R*)-1-(1-(3,5-Bis(trifluoromethyl)phenyl)ethyl)-1*H*-indole (3p)

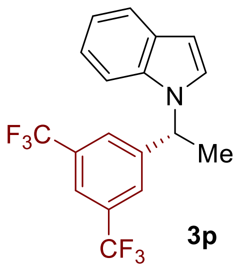

3p

Prepared according to **GP1** with *N*-vinylindole **1a** (56.0 uL, 0.40 mmol, 1.0 equiv.) and **2p** (137.9 uL, 0.80 mmol, 2.0 equiv.). Flash column chromatography (SiO_2, 15:1 PE/EtOAc) afforded the desired product **3p** as a scarlet oil (92.2 mg, 65%).

^{1}H NMR (500 MHz, $CDCl_3$) δ 7.68 (s, 1H), 7.57 (d, J = 7.5 Hz, 1H), 7.46 (s, 2H), 7.25 - 7.11 (m,, 1H), 7.05 (d, J = 14.5 Hz, 3H), 6.55 (d, J = 3.0 Hz, 1H), 5.65 (q, J = 7.0 Hz, 1H), 1.85 (d, J = 7.0 Hz, 3H) .

^{13}C NMR (126 MHz, $CDCl_3$) δ 145.6, 135.9, 132.2 (q , $J_{C\text{-}F}$ = 33.4 Hz), 129.0, 126.0, 124.2 (d , $J_{C\text{-}F}$ = 6.7 HZ), 124.1, 122.0, 121.7 (d, $J_{C\text{-}F}$ = 4.2 Hz), 121.3, 120.1, 109.5, 102.8, 54.2, 21.6 .

^{19}F NMR (471 MHz, $CDCl_3$) δ -62.9 .

HRMS (ESI) m/z: $[M + H]^+$ Calcd for $C_{18}H_{14}F_6N^+$ 358.1025; Found 358.1024.

HPLC: 3.5:96.5 er determined by analytical HPLC, Daicel CHIRALCEL® OD-3 column, 20 °C, Hexane:*i*-PrOH = 90:10, 1.0 mL/min, 254 nm, t_{minor} = 11.1 min, t_{major} = 19.0 min.

$[\alpha]_D^{20}$ = +46.6 (c = 0.35, $CHCl_3$).

Dimethyl (*R*)-5-(1-(1*H*-indol-1-yl)ethyl)isophthalate (3q)

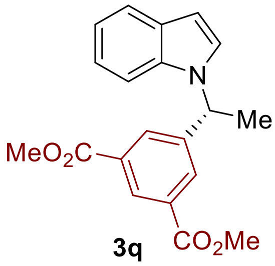

3q

Prepared according to **GP1** with *N*-vinylindole **1a** (56.0 uL, 0.40 mmol, 1.0 equiv.) and **2q** (218.4 mg, 0.80 mmol, 2.0 equiv.). Flash column chromatography (SiO_2, 15:1 PE/EtOAc) afforded the desired product **3q** as a white oil (106.1 mg, 79%).

^{1}H NMR (500 MHz, $CDCl_3$) δ 8.47 (s, 1H), 7.95 (s, 2H), 7.54 (d, J = 7.5 Hz, 1H), 7.22 (d, J = 3.0 Hz, 1H), 7.10 (d, J = 8.0 Hz, 1H), 7.06 -6.94 (m, 2H), 6.51 (d, J = 3.0 Hz, 1H), 5.64 (q, J = 7.0 Hz, 1H), 3.80 (s, 6H), 1.85 (d, J = 7.0 Hz, 3H) .

^{13}**C NMR (126 MHz, CDCl$_3$)** δ 165.8, 143.9, 135.9, 131.22, 131.20, 129.86, 128.93, 124.4, 121.7, 121.1, 119.8, 109.7, 102.2, 54.4, 52.3, 21.6.

HRMS (ESI) m/z: $[M + H]^+$ Calcd for $C_{20}H_{20}NO_4^+$ 338.1387 ; Found 338.1384.

HPLC: 1.5:98.5 er determined by analytical HPLC, Daicel CHIRALCEL® OD-3 column, 20 °C, Hexane:*i*-PrOH = 90:10, 1.0 mL/min, 254 nm, t_{minor} = 20.7 min, t_{major} = 22.3 min.

$[\alpha]_D^{20}$ = +35.3 (*c* = 0.31, $CHCl_3$).

(*R*)-1-(1-(6-(Trifluoromethyl)pyridin-3-yl)ethyl)-1*H*-indole (3r)

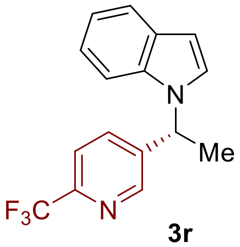

3r

Prepared according to **GP1** with *N*-vinylindole **1a** (56.0 uL, 0.40 mmol, 1.0 equiv.) and **2o** (180.8 mg, 0.80 mmol, 2.0 equiv.). Flash column chromatography (SiO_2, 20:1 PE/EtOAc) afforded the desired product **3r** as a yellow oil (61.0 mg, 53%).

^{1}**H NMR (600 MHz, CDCl$_3$)** δ 8.61 (d, *J* = 2.4 Hz, 1H), 7.70 – 7.62 (m, 1H), 7.55 (d, *J* = 7.8 Hz, 1H), 7.41 - 7.34 (m, 1H), 7.32 (d, *J* = 3.0 Hz, 1H), 7.19 - 7.08 (m, 3H), 6.64 (d, *J* = 3.0 Hz, 1H), 5.78 (q, *J* = 7.2 Hz, 1H), 2.01 (d, *J* = 7.2 Hz, 3H).

^{13}**C NMR (151 MHz, CDCl$_3$)** δ 147.9, 147.4 (d, J_{C-F}= 36.2 Hz), 141.6, 135.8, 134.6, 129.0, 124.1, 122.1, 121.4 (d, J_{C-F}= 273.3 Hz), 121.3, 120.5 (d, J_{C-F}= 4.5 Hz), 120.2, 110.0, 102.8, 52.4, 21.4.

^{19}**F NMR (565 MHz, CDCl$_3$)** δ -67.9.

HRMS (ESI) m/z: $[M + H]^+$ Calcd for $C_{16}H_{14}F_3N_2^+$ 291.1104; Found 291.1104.

HPLC: 4:96 er determined by analytical HPLC, Daicel CHIRALCEL® OD-3 column, 20 °C, Hexane:*i*-PrOH = 90:10, 1.0 mL/min, 254 nm, t_{minor} = 14.6 min, t_{major} = 28.9 min.

$[\alpha]_D^{20}$ = +62.7 (*c* = 0.17., $CHCl_3$).

Methyl (*R*)-3-(1-(5-methoxy-1*H*-indol-1-yl)ethyl)benzoate (3s)

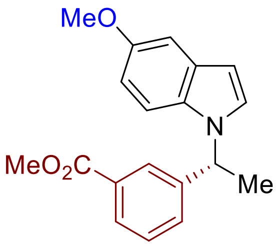

3s

Prepared according to **GP1** with 5-methoxy-1-vinyl-1*H*-indole-**1c** (68.9 mg, 0.20 mmol, 1.0 equiv.) and **2m** (172 mg, 0.80 mmol, 2.0 equiv.). Flash column chromatography (SiO_2, 15:1 PE/EtOAc) afforded the desired product **3s** as a colorless oil (79.0 mg, 64%).

^{1}H NMR (400 MHz, $CDCl_3$) δ 8.01 – 7.81 (m, 2H), 7.35-7.29 (m, 1H), 7.28 (d, *J* = 3.2 Hz, 1H), 7.19-7.15 (m, 1H), 7.09 (d, *J* = 2.4 Hz, 1H), 7.06 (d, *J* = 8.8 Hz, 1H), 6.84 - 6.72 (m,, 1H), 6.50 (d, *J* = 3.2 Hz, 1H), 5.64 (q, *J* = 7.2 Hz, 1H), 3.89 (s, 3H), 3.82 (s, 3H), 1.92 (d, *J* = 7.2 Hz, 3H).

^{13}C NMR (101 MHz, $CDCl_3$) δ 166.8, 154.1, 143.3, 131.3, 130.6, 130.3, 129.2, 128.9, 128.7, 127.0, 125.2, 111.9, 110.6, 102.6, 101.4, 55.8, 54.8, 52.1, 21.7.

HRMS (ESI) m/z: $[M + H]^+$ Calcd for $C_{19}H_{20}NO_3^+$ 310.1438; Found 310.1434.

HPLC: 3.5:96.5 er determined by analytical HPLC, Daicel CHIRALCEL® OD-3 column, 20 °C, Hexane:*i*-PrOH = 90:10, 1.0 mL/min, 254 nm, t_{minor} = 27.0 min, t_{major} = 30.7 min.

$[\alpha]_D^{20}$ = +40.8 (*c* = 0.52, $CHCl_3$).

Methyl (*R*)-3-(1-(5-fluoro-1*H*-indol-1-yl)ethyl)benzoate (3t)

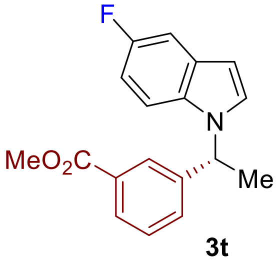

3t

Prepared according to **GP1** with 5-fluoro-1-vinyl-1*H*-indole **1b** (64.5 mg, 0.40 mmol, 1.0 equiv.) and **2m** (172.0 mg, 0.80 mmol, 2.0 equiv.). Flash column chromatography (SiO_2, 10:1 PE/EtOAc) afforded the desired product **3t** as a colorless oil (80.0 mg, 67%).

^{1}H NMR (400 MHz, Acetone-d_6) δ 7.95 – 7.76 (m, 2H), 7.68 (d, *J* = 3.2 Hz, 1H), 7.49 – 7.42 (m, 2H), 7.35 -7.30 (m, 1H), 7.30 - 7.24 (m, 1H), 6.92 -6.76 (m, 1H), 6.56 (d, *J* = 3.2 Hz, 1H), 5.93 (q, *J* = 7.2 Hz, 1H), 3.84 (s, 3H), 1.97 (d, *J* = 7.2 Hz, 3H).

^{13}C NMR (101 MHz, Acetone-d_6) δ 166.8, 158.5 (d, $J_{C\text{-}F}$ = 233.3 Hz), 144.7, 133.6, 131.4, 131.2, 130.1 (d, $J_{C\text{-}F}$ = 10.2 Hz), 129.7, 129.1, 127.7, 127.5, 111.8 (d, $J_{C\text{-}F}$ = 9.8 Hz), 110.0 (d, $J_{C\text{-}F}$ = 26.5 Hz), 105.9 (d, $J_{C\text{-}F}$ = 23.4 Hz), 102.3 (d, $J_{C\text{-}F}$ = 4.7 Hz), 55.4, 52.3, 21.8.

^{19}F NMR (376 MHz, Acetone-d_6) δ -126.6 .

HRMS (ESI) m/z: $[M + H]^+$ Calcd for $C_{18}H_{17}FNO_2^+$ 298.1238; Found 298.1236.

HPLC: 7:93 er determined by analytical HPLC, Daicel CHIRALCEL® OJ-H column, 20 °C, Hexane:*i*-PrOH = 80:20, 1.0 mL/min, 254 nm, t_{minor} = 21.7 min, t_{major} = 41.2 min.

$[\alpha]_D^{20}$ = +42.1 (c = 0.25, $CHCl_3$).

Methyl (*R*)-3-(1-(5-cyano-1*H*-indol-1-yl)ethyl)benzoate (3u)

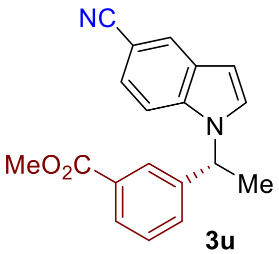

3u

Prepared according to **GP1** with 1-vinyl-1*H*-indole-5-carbonitrile **1d** (67.3 mg, 0.40 mmol, 1.0 equiv.) and **2m** (172.0 mg, 0.80 mmol, 2.0 equiv.). Flash column chromatography (SiO_2, 5:1 PE/EtOAc) afforded the desired product **3u** as a colorless oil (39.8 mg, 41%).

^{1}H NMR (400 MHz, $CDCl_3$) δ 8.05 – 7.84 (m, 3H), 7.42 (d, J = 3.2 Hz, 1H), 7.40 - 7.31 (m, 2H), 7.26 – 7.18 (m, 2H), 6.66 (d, J = 3.2 Hz, 1H), 5.72 (q, J = 7.2 Hz, 1H), 3.90 (s, 3H), 1.96 (d, J = 7.2 Hz, 3H).

^{13}C NMR (101 MHz, $CDCl_3$) δ 166.6, 142.1, 137.4, 130.9, 130.2, 129.11, 129.08, 128.6, 127.8, 126.9, 126.6, 124.5, 120.6, 110.7, 102.9, 102.8, 55.1, 52.2, 21.6.

HRMS (ESI) m/z: $[M + H]^+$ Calcd for $C_{19}H_{17}N_2O_2^+$ 305.1285; Found 305.1281.

HPLC: 12:88 er determined by analytical HPLC, Daicel CHIRALCEL® OD-H column, 20 °C, Hexane:*i*-PrOH = 80:20, 1.0 mL/min, 254 nm, t_{minor} = 27.0 min, t_{major} = 36.8 min.

$[\alpha]_D^{20}$ = +66.4 (c = 0.73, $CHCl_3$).

Methyl (*R*)-3-(1-(4-methyl-1*H*-indol-1-yl)ethyl)benzoate (3v)

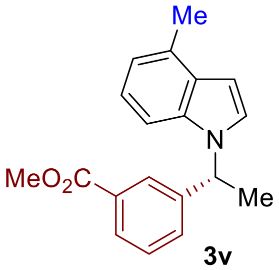

3v

Prepared according to **GP1** with 4-methyl-1-vinyl-1*H*-indole **1e** (62.9 mg, 0.40 mmol, 1.0 equiv.) and **2m** (172 mg, 0.80 mmol, 2.0 equiv.). Flash column chromatography (SiO_2, 10:1 PE/EtOAc) afforded the desired product **3v** as a white solid (69.2 mg, 59%).

^{1}H NMR (400 MHz, $CDCl_3$) δ 7.96 (s, 1H), 7.90 (d, *J* = 8.0 Hz, 1H), 7.35 – 7.26 (m, 2H), 7.19 (d, *J* = 8.0 Hz, 1H), 7.04 (d, *J* = 6.0 Hz, 2H), 6.93 - 6.84 (m, 1H), 6.59 (d, *J* = 3.2 Hz, 1H), 5.68 (q, *J* = 7.2 Hz, 1H), 3.89 (s, 3H), 2.56 (s, 3H), 1.93 (d, *J* = 7.2 Hz, 3H).
^{13}C NMR (101 MHz, $CDCl_3$) δ 166.8, 143.2, 135.7, 130.5, 130.44, 130.40, 128.9, 128.7, 128.6, 127.1, 124.0, 121.7, 119.9, 107.5, 100.3, 54.7, 52.1, 21.6, 18.7.
HRMS (ESI) m/z: $[M + H]^+$ Calcd for $C_{19}H_{20}NO_2^+$ 294.1489; Found 294.1490.
HPLC: 2:98 er determined by analytical HPLC, Daicel CHIRALCEL® OD-H column, 20 °C, Hexane:*i*-PrOH = 80:20, 1.0 mL/min, 254 nm, t_{minor} = 10.8 min, t_{major} = 16.1 min.
$[\alpha]_D^{20}$ = +24.7 (*c* = 0.87, $CHCl_3$).

Methyl (*R*)-3-(1-(3-(2-((*tert*-butyldimethylsilyl)oxy)ethyl)-1*H*-indol-1-yl)ethyl)benzoate (3w)

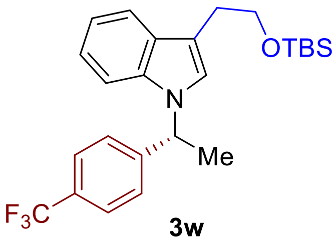

3w

Prepared according to **GP1** with 3-(2-((*tert*-butyldimethylsilyl)oxy)ethyl)-1-vinyl-1*H*-indole **1o** (109.8 mg, 0.40 mmol, 1.0 equiv.) and **2c** (112.1 uL, 0.80 mmol, 2.0 equiv.). Flash column chromatography (SiO_2, 30:1 PE/EtOAc) afforded the desired product **3w** as a yellow oil (85.0 mg, 48%).

^{1}H NMR (500 MHz, Acetone-d_6) δ 7.75 -7.52 (m, 3H), 7.45 (s, 1H), 7.41 (d, *J* = 8.0 Hz, 2H), 7.28 (d, *J* = 8.0 Hz, 1H), 7.10 - 6.98 (m, 2H), 5.90 (q, *J* = 7.0 Hz, 1H), 3.93 (t, *J* = 7.0 Hz, 2H), 3.00 (t, *J* = 7.0 Hz, 2H), 1.96 (d, *J* = 7.0 Hz, 3H), 0.89 (s, 9H), 0.02 (s, 6H).

^{13}C NMR (126 MHz, Acetone-*d$_6$*) δ 149.0, 137.3, 129.8, 129.5, 127.5, 126.3(q, $J_{C\text{-}F}$ = 3.8 Hz), 124.1, 123.8, 122.2, 119.8, 119.7, 113.3, 110.7, 64.4, 54.9, 29.7, 26.3, 21.7, 18.8, -5.2.
^{19}F NMR (471 MHz, Acetone-*d$_6$*) δ -62.9.
HRMS (ESI) m/z: $[M + H]^+$ Calcd for $C_{25}H_{33}F_3NOSi^+$ 448.2278; Found 448.2273.
HPLC: 5:95 er determined by analytical HPLC, Daicel Daicel CHIRALCEL® OD-3 column, 20 °C, Hexane:*i*-PrOH = 90:10, 1.0 mL/min, 254 nm, t_{minor} = 4.3 min, t_{major} = 5.1 min.
$[\alpha]_D^{20}$ = +42.0 (*c* = 0.25, $CHCl_3$).

Methyl (*R*)-3-(1-(9*H*-carbazol-9-yl)ethyl)benzoate (3x)

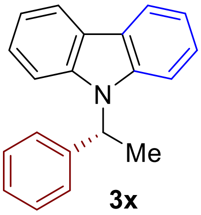

3x

Prepared according to **GP1** with 9-vinyl-9*H*-carbazole (27.0 uL, 0.20 mmol, 1.0 equiv.) and **2h** (42.1 uL, 0.40 mmol, 2.0 equiv.). Flash column chromatography (SiO_2, 30:1 PE/EtOAc) afforded the desired product **3x** as a colorless oil (27.6 mg, 51%).

^{1}H NMR (500 MHz, $CDCl_3$) δ 8.12 (d, *J* = 7.5 Hz, 2H), 7.38 – 7.15 (m, 11H), 6.07 (q, *J* = 7.5 Hz, 1H), 1.99 (d, *J* = 7.0 Hz, 3H).
^{13}C NMR (126 MHz, $CDCl_3$) δ 140.7, 139.8, 128.6, 127.3, 126.5, 125.4, 123.4, 120.3, 118.9, 110.1, 52.3, 17.4.
HRMS (ESI) m/z: $[M + H]^+$ Calcd for $C_{20}H_{18}N^+$ 272.1434; Found 272.1435.
HPLC: 85:15 er determined by analytical HPLC, Daicel CHIRALCEL® OJ-H column, 20 °C, Hexane:*i*-PrOH = 98:2, 1.0 mL/min, 254 nm, t_{major} = 15.5 min, t_{minor} = 18.0 min.
$[\alpha]_D^{20}$ = +66.4 (*c* = 0.38, $CHCl_3$).

Methyl (*R*)-3-(1-(1*H*-indol-1-yl)hexyl)benzoate (3y)

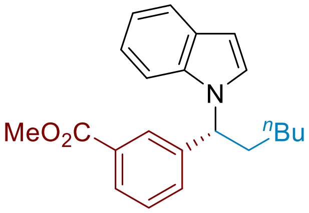

3y

Prepared according to **GP1** with **1p** (79.7 mg, 0.40 mmol, 1.0 equiv.) and **2m** (172.0 mg, 0.80 mmol, 2.0 equiv.), $(OEt)_2MeSiH$ (115.3 uL, 0.72 mmol, 1.8 equiv.), and KF (51.4 mg, 0.88 mmol, 2.2 equiv.); DME as solvent, stirred at room temperature for 48 hours.. Flash column chromatography (SiO_2, 10:1 PE/EtOAc) afforded the desired product **3y** as a yellow oil (57.1 mg, 41%).

^{1}H NMR (500 MHz, $CDCl_3$) δ 7.98 (d, *J* = 2.0 Hz, 1H), 7.89 (d, *J* = 7.5 Hz, 1H), 7.62 (d, *J* = 8.0 Hz, 1H), 7.38 – 7.25 (m, 4H), 7.13 (t, *J* = 7.0 Hz, 1H), 7.11 – 7.03 (m, 1H), 6.59 (d, *J* = 3.0 Hz, 1H), 5.48 (t, *J* = 9.0, 6.5 Hz, 1H), 3.89 (s, 3H), 2.44 - 2.30 (m, 1H), 2.30 - 2.16 (m, 1H), 1.35 – 1.24 (m, 6H), 0.85 (t, *J* = 7.0 Hz, 3H).

^{13}C NMR (126 MHz, $CDCl_3$) δ 166.8, 142.4, 136.3, 130.8, 130.5, 128.8, 128.7, 128.6, 127.4, 124.6, 121.5, 120.9, 119.6, 109.7, 102.0, 59.4, 52.2, 35.4, 31.5, 26.3, 22.4, 13.9 .

HRMS (ESI) m/z: $[M + H]^+$ Calcd for $C_{22}H_{26}NO_2^+$ 336.1958; Found 336.1960.

HPLC: 6.5:93.5 er determined by analytical HPLC, Daicel CHIRALCEL® OD-3 column, 20 °C, Hexane:*i*-PrOH = 90:10, 1.0 mL/min, 254 nm, t_{minor} = 9.3 min, t_{major} = 11.0 min.

$[\alpha]_D^{25}$ = +50.0 (*c* = 0.8, $CHCl_3$).

Methyl (*R*)-3-(3-((*tert*-butoxycarbonyl)(methyl)amino)-1-(1*H*-indol-1-yl)propyl)benzoate (3z)

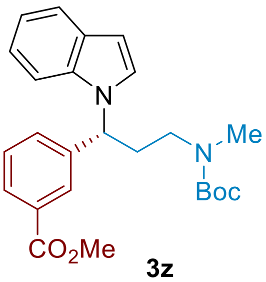

3z

Prepared according to **GP1** with **1q** (57.7 mg, 0.20 mmol, 1.0 equiv.) and **2m** (86.0 mg, 0.40 mmol, 2.0 equiv.), $(OEt)_2MeSiH$ (57.7 uL, 0.36 mmol, 1.8 equiv.), and KF (25.7 mg, 0.44 mmol, 2.2 equiv.); DME as solvent, stirred at room temperature for 48 hours. Flash column chromatography (SiO_2, 10:1 PE/EtOAc) afforded the desired product **3z** a colorless oil (45.4 mg, 54%).

^{1}H NMR (600 MHz, Acetone-*d*$_6$) δ 7.99 (s, 1H), 7.88 (d, *J* = 7.8 Hz, 1H), 7.74 (s, 1H), 7.64 – 7.51 (m, 2H), 7.45 (d, *J* = 7.8 Hz, 2H), 7.09 (t, *J* = 7.8 Hz, 1H), 7.01 (t, *J* = 7.8 Hz, 1H), 6.60 (s, 1H), 5.80 (s, 1H), 3.84 (s, 3H), 3.39 – 3.23 (m, 2H), 2.83 (s, 3H), 2.76 - 2.67 (m, 1H), 2.65 -2.52 (m, 1H), 1.43 - 1.29 (m, 9H).

^{13}C NMR (151 MHz, Acetone-d_6) δ 166.9, 155.7, 143.6, 137.3, 131.7, 131.5, 129.8, 129.7, 129.2, 128.1, 125.8, 122.2, 121.5, 120.3, 110.8, 102.8, 79.4, 57.6, 52.3, 47.1, 34.2, 31.1, 28.4.
HRMS (ESI) m/z: $[M + H]^+$ Calcd for $C_{25}H_{31}N_2O_4^+$ 423.2278; Found 423.2281.
HPLC: 96:4 er determined by analytical HPLC, Daicel CHIRALCEL® OD column, 20 °C, Hexane:*i*-PrOH = 80:20, 1.0 mL/min, 254 nm, t_{major} = 12.8 min, t_{minor} = 17.9 min.
$[\alpha]_D^{25}$ = +31.7 (c = 0.7, $CHCl_3$)

***tert*-Butyl (*R*)-(3-(4-chloro-3-fluorophenyl)-3-(1H-indol-1-yl)propyl)(methyl)carbamate (3aa)**

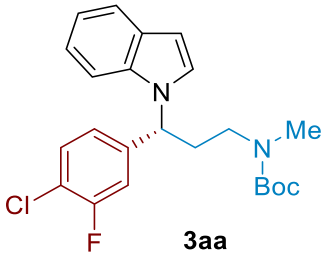

3aa

Prepared according to **GP1** with **1q** (57.7 mg, 0.20 mmol, 1.0 equiv.) and **2o** (49.1 uL, 0.40 mmol, 2.0 equiv.), $(OEt)_2MeSiH$ (57.7 uL, 0.36 mmol, 1.8 equiv.), and KF (25.7 mg, 0.44 mmol, 2.2 equiv.); DME as solvent, stirred at room temperature for 48 hours. Flash column chromatography (SiO_2, 15:1 PE/EtOAc) afforded the desired product **3aa** as a yellow oil (41.1 mg, 50%).

^{1}H NMR (600 MHz, Acetone-d_6) δ 7.72 (s, 1H), 7.58 (d, J = 8.4 Hz, 1H), 7.50- 7.40 (m, 2H), 7.28 (d, J = 10.2 Hz, 1H), 7.16 (d, J = 8.4 Hz, 1H), 7.10 (t, J = 7.2 Hz, 1H), 7.07 - 7.00 (m, 1H), 6.60 (s, 1H), 5.74 (t, J = 7.8 Hz, 1H), 3.39 – 3.24 (m, 2H), 2.83 (s, 3H), 2.73 - 2.64 (m, 1H), 2.61 - 2.53 (m, 1H), 1.43 - 1.29 (m, 9H).
^{13}C NMR (151 MHz, Acetone-d_6) δ 158.7 (d, J_{C-F} = 247.6 Hz), 155.7, 157.8, 144.6 (d, J_{C-F} = 6.0 Hz), 137.2, 131.6, 129.8, 125.6, 124.3 (d, J_{C-F} = 4.5 Hz), 122.3, 121.5, 120.4, 119.9 (d, J_{C-F} = 18.1 Hz), 115.6 (d, J_{C-F} = 21.1 Hz), 110.8, 103.0, 79.4, 56.9, 46.9, 33.8, 32.6, 28.4.
^{19}F NMR (565 MHz, Acetone-d_6) δ -116.8.
HRMS (ESI) m/z: $[M + H]^+$ Calcd for $C_{23}H_{27}ClFN_2O_2^+$ 417.1740; Found 417.1737.
HPLC: 91:9 er determined by analytical HPLC, Daicel CHIRALPAK® AD-H column, 20 °C, Hexane:*i*-PrOH = 98:2, 0.8 mL/min, 254 nm, t_{major} = 12.5 min, t_{minor} = 14.0 min.
$[\alpha]_D^{25}$ = +34.3 (c = 0.4, $CHCl_3$)

tert-Butyl (_R_)-(3-(1_H_-indol-1-yl)-3-(2-(trifluoromethyl)pyridin-4-yl)propyl)(methyl)carbamate (3bb)

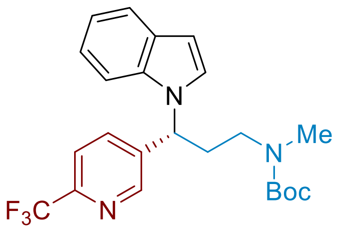

3bb

Prepared according to **GP1** with **1q** (57.7 mg, 0.20 mmol, 1.0 equiv.) and **2r** (90.4 uL, 0.40 mmol, 2.0 equiv.), $(OEt)_2MeSiH$ (57.7 uL, 0.36 mmol, 1.8 equiv.), and KF (25.7 mg, 0.44 mmol, 2.2 equiv.); DME as solvent, stirred at room temperature for 48 hours. Flash column chromatography (SiO_2, 1:1 PE/DCM) afforded the desired product **3bb** as a buff oil (33.3 mg, 38%).

^{1}H NMR (600 MHz, Acetone-d_6) δ 8.77 (s, 1H), 7.92 (s, 1H), 7.84 – 7.74 (m, 2H), 7.59 (d, J = 7.8 Hz, 1H), 7.49 (d, J = 8.4 Hz, 1H), 7.11 (t, J = 7.8 Hz, 1H), 7.04 (t, J = 7.8 Hz, 1H), 6.64 (s, 1H), 5.94 (t, J = 7.2 Hz, 1H), 3.46 - 3.27 (m, 2H), 2.85 - 3.72 (m, 1H), 2.84 (s, 3H), 2.71 - 2.62 (m, 1H), 1.43 - 1.26 (m, 9H).

^{13}C NMR (151 MHz, Acetone-d_6) δ 155.7, 149.5, 147.5 (q, $J_{C\text{-}F}$ = 34.7 Hz), 142.0, 137.2, 136.4, 131.9, 129.9, 129.6, 125.6, 121.9 (d, $J_{C\text{-}F}$ = 191.8 Hz), 121.7, 120.6, 110.7, 103.5, 79.5, 55.4, 46.8, 39.6, 33.5, 28.4.

^{19}F NMR (565 MHz, Acetone-d_6) δ -68.4.

HRMS (ESI) m/z: $[M + Na]^+$ Calcd for $C_{23}H_{26}F_3N_3NaO_2^+$ 456.1869; Found 456.1866.

HPLC: 6:94 er determined by analytical HPLC, Daicel CHIRALCEL® OD-H column, 20 °C, Hexane:*i*-PrOH = 90:10, 1.0 mL/min, 254 nm, t_{minor} = 10.4 min, t_{major} = 16.0 min.

$[\alpha]_D^{25}$ = +34.1 (c = 0.7, $CHCl_3$)

Dimethyl (_R_)-5-(1-(1_H_-indol-1-yl)-3-methoxy-3-oxopropyl)isophthalate (3cc)

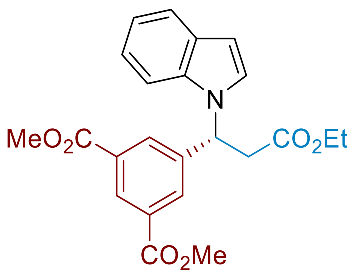

3cc

Prepared according to **GP1** with methyl ethyl (*Z*)-3-(1*H*-indol-1-yl)acrylate (*Z*)-**1r** (86.0 mg, 0.40 mmol, 1.0 equiv.) and **2q** (271.6 mg, 0.80 mmol, 2.0 equiv.), $(OEt)_2MeSiH$ (115.3 uL, 0.72 mmol, 1.8 equiv.), and KF (51.4 mg, 0.88mmol, 2.2equiv.); DME as solvent, stirred at room temperature for 48 hours. Flash column chromatography (SiO_2, 5:1 PE/EtOAc) afforded the desired product **3cc** as a white solid (40.4 mg, 26%).

^{1}H NMR (400 MHz, $CDCl_3$) δ 8.58 (s, 1H), 8.12 (s, 2H), 7.61 (d, *J* = 7.6 Hz, 1H), 7.33 (d, *J* = 8.4 Hz, 1H), 7.26 (s, 1H), 7.20 – 7.14 (m, 1H), 7.10 (t, *J* = 7.2 Hz, 1H), 6.59 (d, *J* = 3.2 Hz, 1H), 6.16 (t, *J* = 7.6 Hz, 1H), 4.05 (q, *J* = 7.2 Hz, 2H), 3.91 (s, 6H), 3.40 - 3.29 (m, 2H), 1.11 (t, *J* = 7.2 Hz, 3H).
^{13}C NMR (101 MHz, $CDCl_3$) δ 169.6, 165.7, 141.1, 136.0, 131.7, 131.4, 130.4, 128.9, 124.5, 122.0, 121.1, 120.0, 109.7, 103.1, 61.2, 55.6, 52.5, 40.2, 13.9.
HRMS (ESI) m/z: $[M + H]^+$ Calcd for $C_{23}H_{24}NO_6^+$ 410.1598; Found 410.1596.
HPLC: 5.5:94.5 er determined by analytical HPLC, Daicel CHIRALPAK® AD-H column, 20 °C, Hexane:*i*-PrOH = 90:10, 1.0 mL/min, 280 nm, t_{minor} = 17.4 min, t_{major} = 19.5 min.
$[\alpha]_D^{20}$ = +46.0 (*c* = 0.28, $CHCl_3$).

(*R*)-1-(4-(1-(3-(2-((*tert*-Butyldimethylsilyl)oxy)ethyl)-1*H*-indol-1-yl)-4-phenylbutyl)-phenyl)ethan-1-one (3dd)

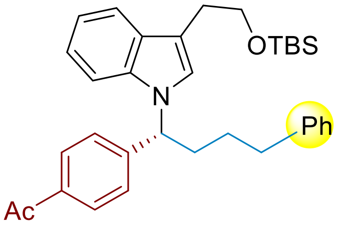

3dd

Prepared according to **GP1** with **1s** (81.1 mg, 0.20 mmol, 1.0 equiv.) and **2a** (79.6 mg, 0.40 mmol, 2.0 equiv.), $(OEt)_2MeSiH$ (57.7 uL, 0.36 mmol, 1.8 equiv.), and KF (25.7 mg, 0.44 mmol, 2.2 equiv.); DME as solvent, stirred at room temperature for 48 hours. Flash column chromatography (SiO_2, 10:1 PE/EtOAc) afforded the desired product **3dd** as a yellow oil (48.0 mg, 46%).

^{1}H NMR (500 MHz, Acetone-*d_6*) δ 7.86 - 7.74 (m, 2H), 7.51 (d, *J* = 8.0 Hz, 1H), 7.43 – 7.26 (m, 4H), 7.19 (t, *J* = 7.0 Hz, 2H), 7.11 (d, *J* = 7.5 Hz, 3H), 7.01 (t, *J* = 7.5 Hz, 1H), 6.95 (t, *J* = 7.5 Hz, 1H), 5.67 (t , 7.5 Hz, 1H), 3.86 (t, *J* = 7.0 Hz, 2H), 2.92 (t, *J* = 7.0 Hz, 2H), 2.67 (m, 2H), 2.46 (s, 3H), 2.40 (m, 1H), 2.29 (m, 1H), 1.65 (m, 2H), 0.83 (s, 9H), -0.04 (s, 6H) .

^{13}C NMR (126 MHz, Acetone-d_6) δ 197.2, 148.3, 142.7, 137.7, 137.2, 129.3, 129.2, 129.13, 129.06, 127.4, 126.6, 123.8, 122.1, 119.6, 113.5, 110.6, 100.8, 64.4, 59.5, 35.8, 35.1, 29.2, 26.5, 26.3, 18.7, 1.3, -5.2 .

HRMS (ESI) m/z: $[M + H]^+$ Calcd for $C_{34}H_{44}NO_2Si^+$ 526.3136; Found 526.3132.

HPLC: 95:5 er determined by analytical HPLC, Daicel CHIRALPAK® AD-H column, 20 °C, Hexane:*i*-PrOH = 98:2, 1.0 mL/min, 254 nm, t_{major} = 16.8 min, t_{minor} = 19.4 min.

$[\alpha]_D^{20}$ = +21.6 (*c* = 0.47, $CHCl_3$).

(*R*,*E*)-1-(4-Phenylbut-3-en-2-yl)-1*H*-indole (4a)

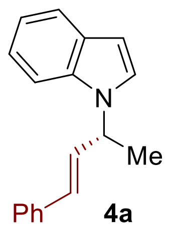

Prepared according to **GP1** with *N*-vinylindole **1a** (56.0 uL, 0.40 mmol, 1.0 equiv.) and **2s** (102.6 uL, 0.80 mmol, 2.0 equiv.). Flash column chromatography (SiO_2, 50:1 PE/EtOAc) afforded the desired product **4a** as a colorless oil (34.6 mg, 35%).

^{1}H NMR (500 MHz, $CDCl_3$) δ 7.65 (d, *J* = 8.0 Hz, 1H), 7.41 (d, *J* = 8.5 Hz, 1H), 7.37 – 7.18 (m, 7H), 7.11 (t, *J* = 7.5 Hz, 1H), 6.55 (d, *J* = 3.0 Hz, 1H), 6.41 (d, *J* = 5.0 Hz, 2H), 5.31 – 5.18 (m, 1H), 1.74 (d, *J* = 7.0 Hz, 3H).

^{13}C NMR (126 MHz, $CDCl_3$) δ 136.3, 135.7, 130.5, 130.3, 128.7, 128.6, 127.8, 126.5, 124.6, 121.4, 121.0, 119.4, 109.8, 101.5, 52.8, 20.3 .

HRMS (ESI) m/z: $[M + H]^+$ Calcd for $C_{18}H_{18}N^+$ 248.1434; Found 248.1438.

HPLC: 13:87 er determined by analytical HPLC, Daicel CHIRALPAK® AD-H column, 20 °C, Hexane:*i*-PrOH = 95:5, 1.0 mL/min, 214 nm, t_{minor} = 6.2 min, t_{major} = 6.9 min.

$[\alpha]_D^{20}$ = -138.2 (*c* = 0.47, $CHCl_3$).

(*R*)-1-(4-Methylpent-3-en-2-yl)-1*H*-indole (4b)

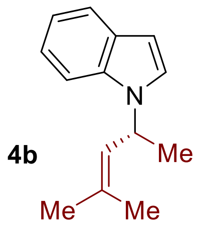

Prepared according to **GP1** with *N*-vinylindole **1a** (56.0 uL, 0.40 mmol, 1.0 equiv.) and **2t** (82.0 uL, 0.80 mmol, 2.0 equiv.). Flash column chromatography (SiO_2, 49:1 PE/DCM) afforded the desired product **4b** as a colorless oil (54.3 mg, 71%).

^{1}H NMR (500 MHz, Acetone-*d*$_6$) δ 7.59 - 7.51 (m, 1H), 7.46 (d, *J* = 8.5 Hz, 1H), 7.37 (d, *J* = 3.0 Hz, 1H), 7.16 - 7.07 (m, 1H), 7.05 - 6.94 (m, 1H), 6.43 (d, *J* = 2.5 Hz, 1H), 5.52 - 5.43 (m, 1H), 5.39 – 5.32 (m, 1H), 1.73 (s, 3H), 1.70 (s, 3H), 1.54 (d, *J* = 7.0 Hz, 3H).

^{13}C NMR (126 MHz, Acetone-*d*$_6$) δ 136.4, 135.2, 129.6, 127.1, 125.3, 121.6, 121.3, 119.7, 110.5, 101.7, 50.0, 25.5, 21.9, 18.1.

HRMS (ESI) m/z: $[M + H]^+$ Calcd for $C_{14}H_{18}N^+$ 200.1434; Found 200.1436.

HPLC: 92:8 er determined by analytical HPLC,Daicel CHIRALCEL® OJ-H column, 20 °C, Hexane:*i*-PrOH = 98:2, 1.0 mL/min, 254 nm, t_{major} = 9.3 min, t_{minor} = 11.6 min.

$[\alpha]_D^{25}$ = +23.6 (*c* = 1.0, $CHCl_3$).

(*R*)-1-(1-Cyclohexylidenepropan-2-yl)-1*H*-indole (4c)

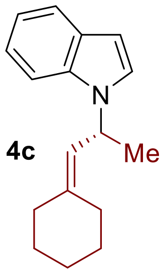

4c

Prepared according to **GP1** with *N*-vinylindole **1a** (56.0 uL, 0.40 mmol, 1.0 equiv.) and **2u** (105.6 uL, 0.80 mmol, 2.0 equiv.). Flash column chromatography (SiO_2, 49:1 PE/DCE) afforded the desired product **4c** as a green oil (54.4 mg, 57%).

^{1}H NMR (600 MHz, Acetone-*d*$_6$) δ 7.54 (d, *J* = 7.8 Hz, 1H), 7.45 (d, *J* = 8.4 Hz, 1H), 7.41-7.25 (m, 1H), 7.12 (t, *J* = 7.8 Hz, 1H), 7.00 (t, *J* = 7.2 Hz, 1H), 6.45-6.39 (m, 1H), 5.46 – 5.40 (m, 1H), 5.38 (d, *J* = 8.4 Hz, 1H), 2.31 – 2.24 (m, 2H), 2.08 (q, *J* = 6.0 Hz, 2H), 1.57 – 1.47 (m, 9H).

^{13}C NMR (151 MHz, Acetone-*d*$_6$) δ 143.1, 136.5, 129.7, 125.3, 123.8, 121.6, 121.4, 119.8, 110.6, 101.7, 49.2, 37.4, 29.2, 28.4, 27.2, 22.3.

HRMS (ESI) m/z: $[M + H]^+$ Calcd for $C_{17}H_{22}N^+$ 240.1747; Found 240.1752.

HPLC: 97.5:2.5 er determined by analytical HPLC, Daicel CHIRALCEL® OJ-H column, 20 °C, Hexane:*i*-PrOH = 98:2, 0.8 mL/min, 280 nm, t_{major} = 9.0 min, t_{minor} = 20.0 min.

$[\alpha]_D^{25}$ = +17.1 (*c* = 0.8, $CHCl_3$).

(*R*)-1-(1-(Cyclohex-1-en-1-yl)ethyl)-1*H*-indole (4d)

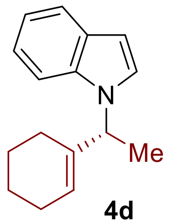

4d

Prepared according to **GP1** with *N*-vinylindole **1a** (56.0 uL, 0.40 mmol, 1.0 equiv.) and **2v** (92.7 uL, 0.80 mmol, 2.0 equiv.). Flash column chromatography (SiO_2, 49:1 PE/DCM) afforded the desired product **4d** as a yellow oil (31.2 mg, 34%). .

^{1}H NMR (600 MHz, Acetone-*d*$_6$) δ 7.54 (d, *J* = 7.8 Hz, 1H), 7.42 (d, *J* = 8.4 Hz, 1H), 7.31-7.25 (m, 1H), 7.10 (t, *J* = 7.8 Hz, 1H), 7.00 (t, *J* = 7.8 Hz, 1H), 6.44 (d, *J* = 3.0 Hz, 1H), 5.70 (s, 1H), 5.00 (d, *J* = 7.2 Hz, 1H), 2.05-1.99 (m, 2H), 1.79 (d, *J* = 17.4 Hz, 1H), 1.68 (d, *J* = 16.2 Hz, 1H), 1.63 (d, *J* = 7.2 Hz, 3H), 1.54 – 1.46 (m, 4H).

^{13}C NMR (151 MHz, Acetone-*d*$_6$) δ 139.0, 137.1, 129.8, 126.0, 123.2, 121.8, 121.3, 119.8, 110.8, 101.6, 57.0, 25.9, 25.6, 23.3, 23.0, 19.0.

HRMS (ESI) m/z: $[M + H]^+$ Calcd for $C_{16}H_{20}N^+$ 226.1590; Found 226.1593.

HPLC: 7:93 er determined by analytical HPLC, Daicel CHIRALCEL® OD-3 column, 20 °C, Hexane:*i*-PrOH = 98:2, 0.8 mL/min, 254 nm, t_{minor} = 7.2 min, t_{major} = 8.3 min.

$[\alpha]_D^{25}$ = +46.1 (*c* = 0.9, $CHCl_3$).

***(R)*-1-(1-*(1H*-Inden-2-yl*)*ethyl*)-1H*-indole (4e)**

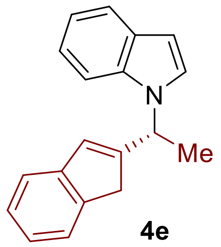

4e

Prepared according to **GP1** with *N*-vinylindole **1a** (56.0 uL, 0.40 mmol, 1.0 equiv.) and **2w** (153.7 mg, 0.80 mmol, 2.0 equiv.). Flash column chromatography (SiO_2, PE) afforded the desired product **4e** as a green oil (62.3 mg, 60%).

^{1}H NMR (600 MHz, $CDCl_3$) δ 7.56 (d, *J* = 8.4 Hz, 1H), 7.30 (d, *J* = 8.4 Hz, 1H), 7.20 (q, *J* = 9.6 Hz, 2H), 7.14 (t, *J* = 7.8 Hz, 1H), 7.12 – 7.06 (m, 2H), 7.06 - 6.99 (m, 2H), 6.59 (d, *J* =

10.8 Hz, 1H), 6.48 – 6.37 (m, 1H), 5.42 (q, *J* = 7.2 Hz, 1H), 3.09 (d, *J* = 7.2 Hz, 2H), 1.77 (d, *J* = 7.2 Hz, 3H).

^{13}C NMR (151 MHz, CDCl$_3$) δ 149.6, 144.1, 143.0, 135.8, 128.7, 127.9, 126.4, 125.0, 124.7, 123.7, 121.5, 121.0, 120.9, 119.5, 109.6, 101.7, 51.6, 39.1, 20.0.

HRMS (ESI) m/z: $[M + H]^+$ Calcd for $C_{19}H_{18}N^+$ 260.1434; Found 260.1435.

HPLC: 96.5:3.5 er determined by analytical HPLC, Daicel CHIRALPAK® AD-H column, 20 °C, Hexane:*i*-PrOH = 98:2, 1.0 mL/min, 254 nm, t_{major} = 5.9 min, t_{minor} = 6.5 min.

$[\alpha]_D^{25}$ = +140.5 (*c* = 0.4, CHCl$_3$).

(*R*)-1-(1-(1*H*-Inden-2-yl)-4-phenylbutyl)-3-(2-((*tert*-butyldimethylsilyl)oxy)ethyl)-1*H*-indole (4f)

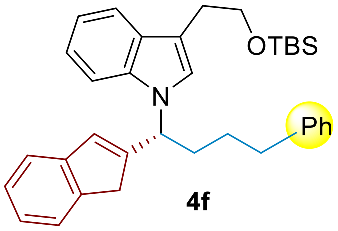

4f

Prepared according to **GP1** with **1s** (81.1 mg, 0.20 mmol, 1.0 equiv.) and **2w** (76.9 mg, 0.40 mmol, 2.0 equiv.), (OEt)$_2$MeSiH (57.7 uL, 0.36 mmol, 1.8 equiv.), and KF (25.7 mg, 0.44 mmol, 2.2 equiv.); DME as solvent, stirred at room temperature for 48 hours. Flash column chromatography (SiO$_2$, 49:1 PE/DCM) afforded the desired product **4f** as a colorless oil (46.3 mg, 41%).

^{1}H NMR (400 MHz, Acetone-*d*$_6$) δ 7.59 - 7.54 (m, 1H), 7.50 (d, *J* = 8.4 Hz, 1H), 7.32 - 7.26 (m, 2H), 7.25 – 7.17 (m, 4H), 7.15 – 7.06 (m, 5H), 7.03 – 6.96 (m, 1H), 6.75 (t, *J* = 1.6 Hz, 1H), 5.53 (t, *J* = 7.6 Hz, 1H), 3.88 (t, *J* = 6.8 Hz, 2H), 3.24 – 3.07 (m, 2H), 2.94 (t, *J* = 6.4 Hz, 2H), 2.72 – 2.60 (m, 2H), 2.35 (q, 8.0 Hz, 2H), 1.71 – 1.63 (m, 1H), 1.61 – 1.54 (m, 1H), 0.85 (s, 9H), -0.01 (s, 3H), -0.02 (s, 3H).

^{13}C NMR (101 MHz, Acetone-*d*$_6$) δ 150.4, 145.2, 143.8, 142.9, 137.6, 129.2, 129.1, 129.0, 128.3, 127.0, 126.5, 125.2, 124.3, 124.2, 122.0, 121.5, 119.6, 119.4, 113.2, 110.6, 64.3, 56.4, 39.5, 35.9, 33.8, 29.7, 29.1, 26.2, 18.7, -5.2.

HRMS (ESI) m/z: $[M + H]^+$ Calcd for $C_{35}H_{44}NOSi^+$ 522.3187; Found 522.3184.

HPLC: 92:8 er determined by analytical HPLC, Daicel CHIRALCEL® OD column, 20 °C, Hexane:*i*-PrOH = 98:2, 1.0 mL/min, 254 nm, t_{major} = 7.5 min, t_{minor} = 14.0 min.

$[\alpha]_D^{25}$ = +53.6 (*c* = 0.2, CHCl$_3$).

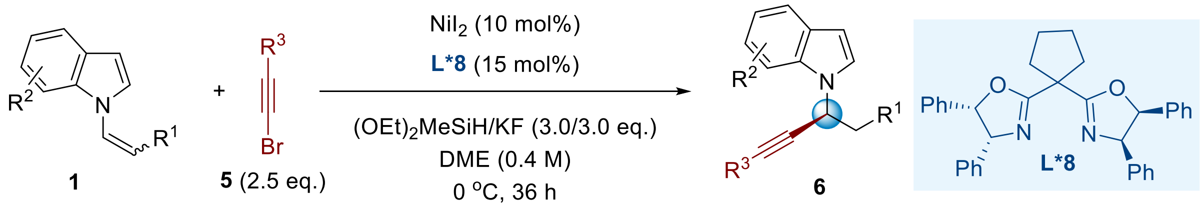

General Procedure 2 (GP2): To an oven-dried 12 mL Teflon-screw cap test tube containing a magnetic stir was charged with NiI_2 (3.1 mg, 10 mol%) and ligand **L*8** (7.7 mg, 15 mol%) under a nitrogen (N_2) atmosphere using glove-box techniques. Subsequently, anhydrous DME (0.25 mL) was added, and the mixture was stirred for 30 minutes at room temperature. Then, KF (17.4 mg, 0.30 mmol, 3.0 equiv), *N*-alkenyl indole **1** (0.10 mmol, 1.0 equiv), alkynyl bromide **5** (0.25 mmol, 2.5 equiv), and $(OEt)_2MeSiH$ (43.0 uL, 0.30 mmol, 3.0 equiv.) were sequentially added. Afterwards, the tube was sealed with airtight electrical tapes and removed from the glove box and stirred at 0 °C for 36 hours at 500 rpm. After the reaction was completed, the reaction mixture was diluted with saturated NH_4Cl (aq., 1.0 mL) and EtOAc (5.0 mL). The aqueous phase was extracted with EtOAc (2 x 5.0 mL) and the combined organic phases were concentrated in vacuo. The crude mixture was purified by flash column chromatography on silica gel using a mixture of PE/EtOAc as eluent to obtain the desired product **6**.

(*S*)-1-(4-(Triisopropylsilyl)but-3-yn-2-yl)-1*H*-indole (6a)

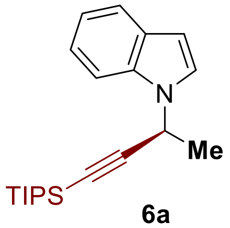

6a

Prepared according to **GP2** with 1-vinyl-1*H*-indole **1a** (14.3 mg, 0.10 mmol, 1.0 equiv.) and **5a** (66.0 uL, 0.25 mmol, 2.5 equiv.). Flash column chromatography (SiO_2, 100:0 petroleum ether/EtOAc) afforded the desired product **6a** as a colorless oil (28.7 mg, 88%).

^{1}H NMR (400 MHz, $CDCl_3$) δ 7.63 (d, *J* = 8.0 Hz, 1H), 7.44 – 7.42 (m, 2H), 7.23 – 7.19 (m, 1H), 7.13 – 7.09 (m, 1H), 6.53 (d, *J* = 3.2 Hz, 1H), 5.36 (q, *J* = 6.8 Hz, 1H), 1.72 (d, *J* = 6.8 Hz, 3H), 1.08 (s, 21H).

^{13}C NMR (101 MHz, $CDCl_3$) δ 134.9, 129.1, 125.3, 121.4, 121.1, 119.6, 109.5, 105.7, 101.6, 86.1, 44.4, 23.4, 18.6, 11.1.

HRMS (ESI) m/z: $[M + H]^+$ Calcd for $C_{21}H_{32}NSi^+$ 326.2299; Found 326.2298.

HPLC: 5:95 er determined by analytical HPLC, Daicel CHIRALPAK® IB-3 column, 25 °C, Hexane:*i*-PrOH = 100:0, 1.0 mL/min, 254 nm, t_{minor} = 9.0 min, t_{major} = 12.8 min.

$[\alpha]_D^{20}$ = -7.2 (c = 0.42, $CHCl_3$).

(*S*)-4-Methyl-1-(4-(triisopropylsilyl)but-3-yn-2-yl)-1*H*-indole (6b)

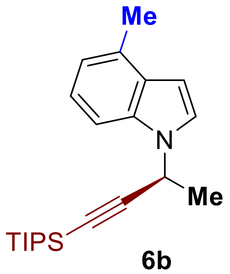

6b

Prepared according to **GP2** with 4-methyl-1-vinyl-1*H*-indole **1e** (15.7 mg, 0.10 mmol, 1.0 equiv.) and **5a** (66.0 uL, 0.25 mmol, 2.5 equiv.). Flash column chromatography (SiO_2, 100:0 petroleum ether/EtOAc) afforded the desired product **6b** as a colorless oil (22.1 mg, 65%).

^{1}H NMR (500 MHz, $CDCl_3$) δ 7.44 (d, J = 3.5 Hz, 1H), 7.29 – 7.27 (m, 1H), 7.15 – 7.12 (m, 1H), 6.94 – 6.93 (m, 1H), 6.56 (d, J = 3.0 Hz, 1H), 5.36 (q, J = 7.0 Hz, 1H), 2.57 (s, 3H), 1.73 (d, J = 7.0 Hz, 3H), 1.10 (s, 21H).

^{13}C NMR (125 MHz, $CDCl_3$) δ 134.6, 130.5, 128.9, 124.7, 121.6, 119.8, 107.1, 105.8, 100.1, 86.0, 44.5, 23.5, 18.7, 18.6, 11.1.

HRMS ((ESI)) m/z: $[M + H]^+$ Calcd for $C_{22}H_{34}NSi^+$ 340.2455; Found 340.2452.

HPLC: 4.5:95.5 er determined by analytical HPLC, Daicel CHIRALPAK® IB-3 column, 25 °C, Hexane:*i*-PrOH = 100:0, 1.0 mL/min, 254 nm, t_{minor} = 7.9 min, t_{major} = 11.4 min.

$[\alpha]_D^{20}$ = -16.5 (c = 0.42, $CHCl_3$).

(*S*)-5-Chloro-1-(4-(triisopropylsilyl)but-3-yn-2-yl)-1*H*-indole (6c)

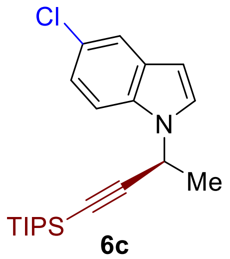

6c

Prepared according to **GP2** with 5-chloro-1-vinyl-1*H*-indole **1f** (17.8 mg, 0.10 mmol, 1.0 equiv.) and **5a** (66.0 uL, 0.25 mmol, 2.5 equiv.). Flash column chromatography (SiO_2, 100:0 petroleum ether/EtOAc) afforded the desired product **6c** as a colorless oil (24.1 mg, 67%).

^{1}H NMR (600 MHz, CDCl$_3$) δ 7.59 (s, 1H), 7.42 – 7.35 (m, 2H), 7.16 – 7.15 (m, 1H), 6.46 (s, 1H), 5.31 (q, J = 6.6 Hz, 1H), 1.72 (d, J = 6.6 Hz, 3H), 1.08 (s, 21H).

^{13}C NMR (126 MHz, CDCl$_3$) δ 133.3, 130.1, 126.7, 125.3, 121.7, 120.4, 110.6, 105.2, 101.3, 86.6, 44.8, 23.2, 18.6, 11.1.

HRMS ((ESI)) m/z: $[M + H]^+$ Calcd for $C_{21}H_{31}ClNSi^+$ 360.1909; Found 360.1907.

HPLC: 9.5:90.5 er determined by analytical HPLC, Daicel CHIRALPAK® IB-3 column, 25 °C, Hexane:*i*-PrOH = 100:0, 1.0 mL/min, 254 nm, t_{minor} = 8.1 min, t_{major} = 20.4 min.

$[\alpha]_D^{20}$ = -9.86 (c = 1.0, CHCl$_3$).

(*S*)-5-Fluoro-1-(4-(triisopropylsilyl)but-3-yn-2-yl)-1*H*-indole (6d)

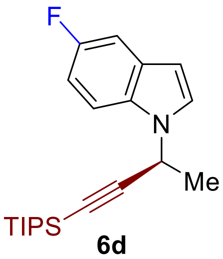

6d

Prepared according to **GP2** with 5-fluoro-1-vinyl-1*H*-indole **1b** (16.1 mg, 0.10 mmol, 1.0 equiv.) and **5a** (66.0 uL, 0.25 mmol, 2.5 equiv.). Flash column chromatography (SiO$_2$, 100:0 petroleum ether/EtOAc) afforded the desired product **6d** as a colorless oil (25.5 mg, 74%).

^{1}H NMR (400 MHz, CDCl$_3$) δ 7.44 (d, J = 3.2 Hz, 1H), 7.36 (dd, J = 9.2, 4.4 Hz, 1H), 7.29 – 7.27 (m, 1H), 6.99 – 6.93 (m, 1H), 6.49 (d, J = 2.8 Hz, 1H), 5.31 (q, J = 6.8 Hz, 1H), 1.72 (d, J = 6.8 Hz, 3H), 1.09 (s, 21H).

^{13}C NMR (101 MHz, CDCl$_3$) δ 157.9 (d, $J_{c\text{-}F}$ = 234.2 Hz), 131.5, 129.3 (d, $J_{c\text{-}F}$ = 10.1 Hz), 126.9, 110.2 (d, $J_{c\text{-}F}$ = 9.8 Hz), 109.8 (d, $J_{c\text{-}F}$ = 26.2 Hz), 105.8 (d, $J_{c\text{-}F}$ = 23.2 Hz), 105.4, 101.5 (d, $J_{c\text{-}F}$ = 4.7 Hz), 86.4, 44.8, 23.2, 18.6, 11.1.

^{19}F NMR (376 MHz, CDCl$_3$) δ -125.2.

HRMS ((ESI)) m/z: $[M + H]^+$ Calcd for $C_{21}H_{31}FNSi^+$ 344.2204; Found 344.2204.

HPLC: 6.5:93.5 er determined by analytical HPLC, Daicel CHIRALPAK® IB-3 column, 25 °C, Hexane:*i*-PrOH = 100:0, 1.0 mL/min, 254 nm, t_{minor} = 4.5 min, t_{major} = 5.6 min.

$[\alpha]_D^{20}$ = -4.5 (c = 0.72, CHCl$_3$).

(*S*)-5-Methyl-1-(4-(triisopropylsilyl)but-3-yn-2-yl)-1*H*-indole (6e)

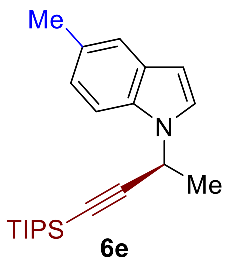

6e

Prepared according to **GP2** with 5-methyl-1-vinyl-1*H*-indole **1g** (15.7 mg, 0.10 mmol, 1.0 equiv.) and **5a** (66.0 uL, 0.25 mmol, 2.5 equiv.). Flash column chromatography (SiO_2, 100:0 petroleum ether/EtOAc) afforded the desired product **6e** as a colorless oil (25.2 mg, 74%).

^{1}H NMR (400 MHz, $CDCl_3$) δ 7.43 (s, 1H), 7.39 (d, *J* = 3.2 Hz, 1H), 7.33 – 7.31 (m, 1H), 7.05 (d, *J* = 8.4, 1H), 6.45 (d, *J* = 3.2 Hz, 1H), 5.33 (q, *J* = 6.8 Hz, 1H), 2.46 (s, 3H), 1.71 (d, *J* = 6.8 Hz, 3H), 1.10 (s, 21H).

^{13}C NMR (101 MHz, $CDCl_3$) δ 133.3, 129.3, 128.7, 125.4, 123.0, 120.7, 109.1, 105.8, 101.0, 85.9, 44.5, 23.4, 21.3, 18.6, 11.1.

HRMS ((ESI)) m/z: $[M + H]^+$ Calcd for $C_{22}H_{34}NSi^+$ 340.2455; Found 340.2451.

HPLC: 5.5:94.5 er determined by analytical HPLC, Daicel CHIRALPAK® IB-3 column, 25 °C, Hexane:*i*-PrOH = 100:0, 1.0 mL/min, 254 nm, t_{minor} = 7.7 min, t_{major} = 16.7 min.

$[\alpha]_D^{20}$ = -8.2 (*c* = 0.46, $CHCl_3$).

(*S*)-5-Methoxy-1-(4-(triisopropylsilyl)but-3-yn-2-yl)-1*H*-indole (6f)

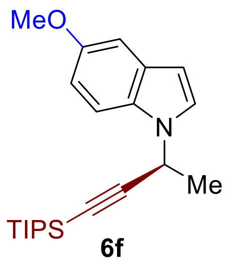

6f

Prepared according to **GP2** with 5-methoxy-1-vinyl-1*H*-indole **1c** (17.3 mg, 0.10 mmol, 1.0 equiv.) and **5a** (66.0 uL, 0.25 mmol, 2.5 equiv.); $NiI_2 \cdot xH_2O$ instead of NiI_2. Flash column chromatography (SiO_2, 100:1 petroleum ether/EtOAc) afforded the desired product **6f** as a white solid (23.5 mg, 66%).

^{1}H NMR (500 MHz, $CDCl_3$) δ 7.38 (d, *J* = 3.0 Hz, 1H), 7.33 (d, *J* = 9.0 Hz, 1H), 7.11 (d, *J* = 2.0 Hz, 1H), 6.88 (d, *J* = 9.0, 1H), 6.45 (d, *J* = 3.0 Hz, 1H), 5.30 (q, *J* = 7.0 Hz, 1H), 3.86 (s, 3H), 1.71 (d, *J* = 7.0 Hz, 3H), 1.09 (s, 21H).

^{13}C NMR (126 MHz, CDCl$_3$) δ 154.1, 130.2, 129.5, 126.0, 111.8, 110.3, 105.7, 102.7, 101.2, 86.1, 55.9, 44.7, 23.4, 18.6, 11.1.

HRMS (ESI) m/z: [M + H]$^+$ Calcd for $C_{22}H_{34}NOSi^+$ 356.2404; Found 356.2409.

HPLC: 4.5:95.5 er determined by analytical HPLC, Daicel CHIRALPAK® IB-3 column, 25 °C, Hexane:*i*-PrOH = 99:1, 1.0 mL/min, 254 nm, t_{minor} = 4.8 min, t_{major} = 6.5 min.

[α]$_D^{20}$ = -7.7 (*c* = 0.31, CHCl$_3$).

(*S*)-5-(4,4,5,5-Tetramethyl-1,3,2-dioxaborolan-2-yl)-1-(4-(triisopropylsilyl)but-3-yn-2-yl)-1*H*-indole (6g)

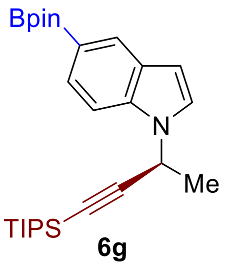

6g

Prepared according to GP2 with 5-(4,4,5,5-tetramethyl-1,3,2-dioxaborolan-2-yl)-1-vinyl-1*H*-indole **1h** (26.9 mg, 0.10 mmol, 1.0 equiv.) and **5a** (66.0 uL, 0.25 mmol, 2.5 equiv.). Flash column chromatography (SiO$_2$, 50:1 petroleum ether/EtOAc) afforded the desired product **6g** as a colorless oil (30.3 mg, 67%).

^{1}H NMR (400 MHz, CDCl$_3$) δ 8.17 (s, 1H), 7.66 (d, *J* = 8.4, 1H), 7.44 – 7.41 (m, 2H), 6.55 (d, *J* = 3.2 Hz, 1H), 5.37 (q, *J* = 6.8 Hz, 1H), 1.71 (d, *J* = 6.8 Hz, 3H), 1.37 (s, 12H), 1.09 (s, 21H).

^{13}C NMR (101 MHz, CDCl$_3$) δ 136.8, 129.0, 128.8, 127.5, 125.5, 108.9, 105.5, 102.3, 86.2, 83.4, 44.5, 24.9, 24.9, 23.4, 18.6, 11.1.

HRMS (ESI) m/z: [M + H]$^+$ Calcd for $C_{27}H_{43}BNO_2Si^+$ 451.3187; Found 451.3182.

HPLC: 6:94 er determined by analytical HPLC, Daicel CHIRALPAK® IB-3 column, 25 °C, Hexane:*i*-PrOH = 99:1, 1.0 mL/min, 254 nm, t_{minor} = 3.7 min, t_{major} = 4.4 min.

[α]$_D^{20}$ = -16.2 (*c* = 0.72, CHCl$_3$).

(*S*)-6-Fluoro-1-(4-(triisopropylsilyl)but-3-yn-2-yl)-1*H*-indole (6h)

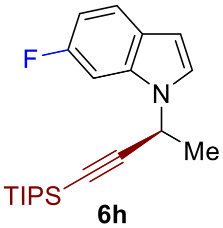

6h

Prepared according to GP2 with 6-fluoro-1-vinyl-1*H*-indole **1i** (16.1 mg, 0.10 mmol, 1.0 equiv.) and **5a** (66.0 uL, 0.25 mmol, 2.5 equiv.). Flash column chromatography (SiO_2, 100:0 petroleum ether/EtOAc) afforded the desired product **6h** as a colorless oil (26.5 mg, 77%).

^{1}H NMR (400 MHz, $CDCl_3$) δ 7.55 – 7.51 (m, 1H), 7.37 (d, *J* = 3.2 Hz, 1H), 7.15 (dd, *J* = 10.0, 2.0 Hz, 1H), 6.91 – 6.86 (m, 1H), 6.50 (d, *J* = 3.2 Hz, 1H), 5.25 (q, *J* = 6.8 Hz, 1H), 1.72 (d, *J* = 6.8 Hz, 3H), 1.09 (s, 21H).

^{13}C NMR (101 MHz, $CDCl_3$) δ 159.5 (d, $J_{c\text{-}F}$ = 237.4 Hz), 134.8 (d, $J_{c\text{-}F}$ = 12.0 Hz), 125.8 (d, $J_{c\text{-}F}$ = 3.6 Hz), 125.5, 121.7 (d, $J_{c\text{-}F}$ =10.2 Hz), 108.3 (d, $J_{c\text{-}F}$ = 24.6 Hz), 105.2, 101.7, 96.2 (d, $J_{c\text{-}F}$ = 26.8 Hz), 86.5, 44.8, 23.1, 18.6, 11.1.

^{19}F NMR (376 MHz, $CDCl_3$) δ -121.1.

HRMS (ESI) m/z: $[M + H]^+$ Calcd for $C_{21}H_{31}FNSi^+$ 344.2204; Found 344.2209.

HPLC: 6:94 er determined by analytical HPLC, Daicel CHIRALPAK® IB-3 column, 25 °C, Hexane:*i*-PrOH = 100:0, 1.0 mL/min, 254 nm, t_{minor} = 6.4 min, t_{major} = 9.7 min.

$[\alpha]_D^{20}$ = -17.2 (*c* = 1.0, $CHCl_3$).

(*S*)-6-(Benzyloxy)-1-(4-(triisopropylsilyl)but-3-yn-2-yl)-1*H*-indole (6i)

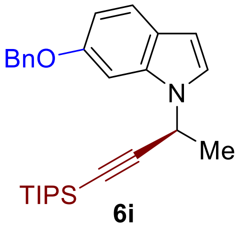

6i

Prepared according to GP2 with 6-(benzyloxy)-1-vinyl-1*H*-indole **1g** (24.9 mg, 0.10 mmol, 1.0 equiv.) and **5a** (66.0 uL, 0.25 mmol, 2.5 equiv.). Flash column chromatography (SiO_2, 100:1 petroleum ether/EtOAc) afforded the desired product **6i** as a colorless oil (31.1 mg, 76%).

^{1}H NMR (400 MHz, $CDCl_3$) δ 7.54 – 7.49 (m, 3H), 7.43 – 7.40 (m, 2H), 7.36 – 7.33 (m, 2H), 7.01 (d, *J* = 2.0 Hz, 1H), 6.90 (dd, *J* = 8.4, 2.4 Hz, 1H), 6.48 (d, *J* = 3.2 Hz, 1H), 5.27 (q, *J* = 6.8 Hz, 1H), 5.14 (s, 2H), 1.70 (d, *J* = 6.8 Hz, 3H), 1.11 (s, 21H).

^{13}C NMR (101 MHz, CDCl$_3$) δ 155.2, 137.4, 135.5, 128.5, 127.8, 127.5, 124.4, 123.6, 121.6, 110.0, 105.6, 101.5, 94.9, 86.0, 70.7, 44.4, 23.0, 18.6, 11.1.

HRMS (ESI) m/z: [M + H]$^+$ Calcd for $C_{28}H_{38}NOSi^+$ 432.2717; Found 432.2718.

HPLC: 7:93 er determined by analytical HPLC, Daicel CHIRALPAK® IB-3 column, 25 °C, Hexane:*i*-PrOH = 99:1, 1.0 mL/min, 254 nm, t_{minor} = 4.7 min, t_{major} = 5.1 min.

[α]$_D^{20}$ = -30.1 (*c* = 1.1, CHCl$_3$).

(*S*)-5-(4-(Triisopropylsilyl)but-3-yn-2-yl)-5*H*-[1,3]dioxolo[4,5-*f*]indole (6j)

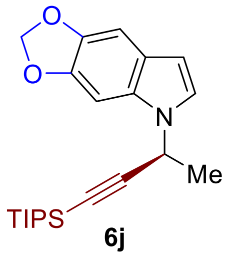

6j

Prepared according to GP2 with 5-vinyl-5*H*-[1,3]dioxolo[4,5-f]indole **1k** (18.7 mg, 0.10 mmol, 1.0 equiv.) and **5a** (66.0 uL, 0.25 mmol, 2.5 equiv.). Flash column chromatography (SiO$_2$, 100:1 petroleum ether/EtOAc) afforded the desired product **6j** as a colorless oil (28.1 mg, 76%).

^{1}H NMR (400 MHz, CDCl$_3$) δ 7.30-7.25 (m, 1H), 7.01 (s, 1H), 6.93 (s, 1H), 6.40 (d, *J* = 3.2 Hz, 1H), 5.94 (s, 2H), 5.21 (q, *J* = 6.8 Hz, 1H), 1.70 (d, *J* = 6.8 Hz, 3H), 1.09 (s, 21H).

^{13}C NMR (101 MHz, CDCl$_3$) δ 144.6, 142.8, 129.9, 123.9, 122.8, 105.5, 101.7, 100.5, 99.5, 90.8, 86.2, 44.8, 23.1, 18.6, 11.1.

HRMS (ESI) m/z: [M + H]$^+$ Calcd for $C_{22}H_{32}NO_2Si^+$ 370.2197; Found 370.2196.

HPLC: 6:94 er determined by analytical HPLC, Daicel CHIRALPAK® IB-3 column, 25 °C, Hexane:*i*-PrOH = 99:1, 1.0 mL/min, 254 nm, t_{minor} = 4.7 min, t_{major} = 6.3 min.

[α]$_D^{20}$ = -19.7 (*c* = 0.41, CHCl$_3$).

(*S*)-7-Fluoro-1-(4-(triisopropylsilyl)but-3-yn-2-yl)-1*H*-indole (6k)

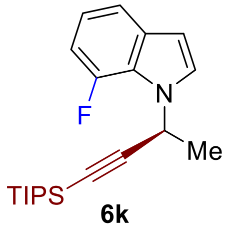

6k

Prepared according to GP2 with 7-fluoro-1-vinyl-1*H*-indole **1l** (16.1 mg, 0.10 mmol, 1.0 equiv.) and **5a** (66.0 uL, 0.25 mmol, 2.5 equiv.). Flash column chromatography (SiO_2, 100:0 petroleum ether/EtOAc) afforded the desired product **6k** as a colorless oil (30.6 mg, 89%).

^{1}H NMR (400 MHz, $CDCl_3$) δ 7.55 (d, J = 3.2 Hz, 1H), 7.37 (d, J = 8.0 Hz, 1H), 7.02 – 6.97 (m, 1H), 6.91 – 6.85 (m, 1H), 6.55 – 6.54 (m, 1H), 5.73 (q, J = 6.8 Hz, 1H), 1.71 (d, J = 6.8 Hz, 3H), 1.11 (s, 21H).

^{13}C NMR (101 MHz, $CDCl_3$) δ 150.0 (d, $J_{c\text{-}F}$ = 243.4 Hz), 133.0 (d, $J_{c\text{-}F}$ = 6.1 Hz), 126.5, 122.9 (d, $J_{c\text{-}F}$ = 10.1 Hz), 119.7 (d, $J_{c\text{-}F}$ =7.1 Hz), 116.8 (d, $J_{c\text{-}F}$ = 3.0 Hz),107.3 (d, $J_{c\text{-}F}$ = 18.2 Hz), 105.8, 102.5 (d, $J_{c\text{-}F}$ = 2.0 Hz), 86.6, 46.5, 46.4, 24.9, 18.6, 11.1.

^{19}F NMR (376 MHz, $CDCl_3$) δ -135.5.

HRMS (ESI) m/z: $[M + H]^+$ Calcd for $C_{21}H_{31}FNSi^+$ 344.2204; Found 344.2200.

HPLC: 1.5:98.5 er determined by analytical HPLC, Daicel CHIRALPAK® IB-3 column, 25 °C, Hexane:*i*-PrOH = 100:0, 1.0 mL/min, 254 nm, t_{minor} = 6.0 min, t_{major} = 6.3 min.

$[\alpha]_D^{20}$ = -19.8 (c = 0.97, $CHCl_3$).

(*S*)-7-Methoxy-1-(4-(triisopropylsilyl)but-3-yn-2-yl)-1*H*-indole (6l)

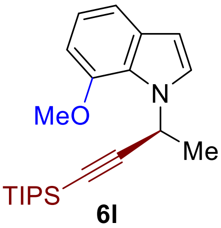

6l

Prepared according to GP2 with 7-methoxy-1-vinyl-1*H*-indole **1m** (17.3 mg, 0.10 mmol, 1.0 equiv.) and **5a** (66.0 uL, 0.25 mmol, 2.5 equiv.). Flash column chromatography (SiO_2, 100:0 petroleum ether/EtOAc) afforded the desired product **6l** as a colorless oil (26.3 mg, 74%).

^{1}H NMR (400 MHz, $CDCl_3$) δ 7.54 (d, J = 3.2 Hz, 1H), 7.24 – 7.22 (m, 1H), 7.01 (t, J = 7.8 Hz, 1H), 6.64 (d, J = 7.6 Hz, 1H), 6.50 (d, J = 3.2 Hz, 1H), 6.17 (q, J = 6.8 Hz, 1H), 3.96 (s, 3H), 1.66 (d, J = 6.8 Hz, 3H), 1.12 (s, 21H).

^{13}C NMR (101 MHz, $CDCl_3$) δ 147.3, 131.1, 125.8, 124.7, 119.9, 113.9, 107.0, 102.4, 101.9, 85.7, 55.3, 46.3, 25.4, 18.6, 11.2.

HRMS (ESI) m/z: $[M + H]^+$ Calcd for $C_{22}H_{34}NOSi^+$ 356.2404; Found 356.2403.

HPLC: 1.5:98.5 er determined by analytical HPLC, Daicel CHIRALPAK® IB-3 column, 25 °C, Hexane:*i*-PrOH = 100:0, 0.7 mL/min, 254 nm, t_{minor} = 11.9 min, t_{major} = 12.2 min.

$[\alpha]_D^{20}$ = -15.7 (c = 0.69, $CHCl_3$).

(*S*)-3-(2-((*tert*-Butyldimethylsilyl)oxy)ethyl)-1-(4-(triisopropylsilyl)but-3-yn-2-yl)-1*H*-indole (6m)

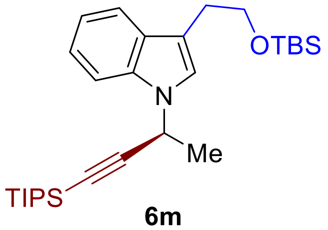

6m

Prepared according to GP2 with 3-(2-((*tert*-butyldimethylsilyl)oxy)ethyl)-1-vinyl-1*H*-indole **1o** (30.2 mg, 0.10 mmol, 1.0 equiv.) and **5a** (66.0 uL, 0.25 mmol, 2.5 equiv.); $NiI_2 \cdot xH_2O$ instead of NiI_2. Flash column chromatography (SiO_2, 100:1 petroleum ether/EtOAc) afforded the desired product **6m** as a colorless oil (30.5 mg, 63%).

^{1}H NMR (600 MHz, $CDCl_3$) δ 7.61 (d, J = 7.8 Hz, 1H), 7.40 – 7.38 (m, 1H), 7.24 (s, 1H), 7.20 (t, J = 7.2 Hz, 1H), 7.12 (t, J = 7.2 Hz, 1H), 5.31 (q, J = 6.6 Hz, 1H), 3.88 (t, J = 7.2 Hz, 2H), 3.00 (t, J = 7.2 Hz, 2H), 1.70 (d, J = 6.6 Hz, 3H), 1.09 (s, 21H), 0.91 (s, 9H), 0.04 (s, 6H).

^{13}C NMR (126 MHz, $CDCl_3$) δ 135.1, 128.8, 123.3, 121.4, 119.2, 119.0, 112.1, 109.4, 106.0, 85.8, 63.9, 44.2, 29.1, 26.0, 23.3, 18.6, 18.4, 11.1, -5.3.

HRMS (ESI) m/z: $[M + H]^+$ Calcd for $C_{29}H_{50}NOSi_2^+$ 484.3425; Found 484.3428.

HPLC: 5.5:94.5 er determined by analytical HPLC, Daicel CHIRALPAK® IB-3 column, 25 °C, Hexane:*i*-PrOH = 100:0, 1.0 mL/min, 254 nm, t_{minor} = 10.6 min, t_{major} = 14.8 min.

$[\alpha]_D^{20}$ = -10.71 (c = 0.85, $CHCl_3$).

(*R*)-9-(4-(Triisopropylsilyl)but-3-yn-2-yl)-2,3,4,9-tetrahydro-1*H*-carbazole (6n)

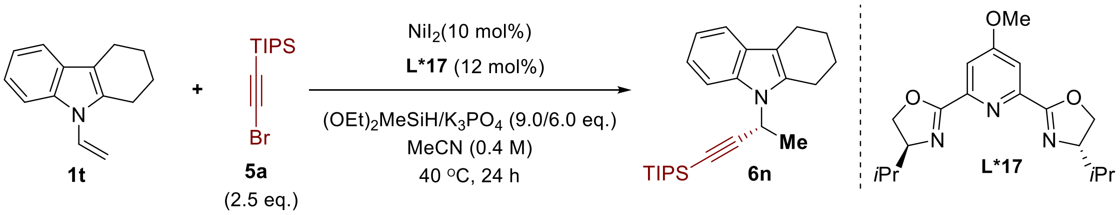

Prepared according to **GP2** with NiI_2 (10 mol%) and ligand **L*17** (4.3mg, 12 mol%), 9-vinyl-2,3,4,9-tetrahydro-1*H*-carbazole **1n** (19.7 mg, 0.10 mmol, 1.0 equiv.), **5a** (66.0 uL, 0.25 mmol, 2.5 equiv.), $(OEt)_2MeSiH$ (129.0 uL, 0.90 mmol, 9.0 equiv.) and K_3PO_4 (127.4 mg, 0.60 mmol, 6.0 equiv.); MeCN as solvent and stirred at 40 ℃ for 24 hours. Flash column

chromatography (SiO_2, 100:0 petroleeum ether/EtOAc) afforded the desired product **6n** as a colorless oil (16.3 mg, 43%).

^{1}H NMR (400 MHz, CDCl$_3$) δ 7.66 (d, *J* = 8.0 Hz, 1H), 7.47 – 7.46 (m, 1H), 7.14 – 7.05 (m, 2H), 5.32 (q, *J* = 7.2 Hz, 1H), 2.91 – 2.72 (m, 4H), 1.97 – 1.85 (m, 4H), 1.69 (d, *J* = 7.2 Hz, 3H), 1.07 (s, 21H).

^{13}C NMR (101 MHz, CDCl$_3$) δ 134.9, 134.5, 127.9, 120.4, 118.7, 117.7, 110.6, 110.2, 106.2, 85.4, 42.2, 23.4, 23.0, 22.9, 22.7, 21.1, 18.6, 11.2.

HRMS (ESI) m/z: $[M + H]^+$ Calcd for $C_{25}H_{38}NSi^+$ 380.2768; Found 380.2764.

HPLC: 10:90 er determined by analytical HPLC, Daicel CHIRALPAK® IB-3 column, 25 °C, Hexane:*i*-PrOH = 100:0, 1.0 mL/min, 254 nm, t_{minor} = 12.2 min, t_{major} = 13.4 min.

$[\alpha]_D^{20}$ = -6.8 (*c* = 0.4, CHCl$_3$).

Ethyl (*S*)-3-(1H-indol-1-yl)-5-(triisopropylsilyl)pent-4-ynoate (6o)

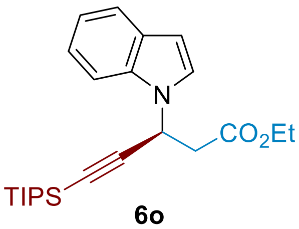

6o

Prepared according to GP2 with ethyl (*E*)-3-(1*H*-indol-1-yl) acrylate **1q** (21.5 mg, 0.10 mmol, 1.0 equiv.) and **5a** (66.0 uL, 0.25 mmol, 2.5 equiv.); $NiI_2 \cdot xH_2O$ instead of NiI_2, MeCN instead of DME as solvent and stirred at 0 ℃ for 48 hours. Flash column chromatography (SiO_2, 20:1 petroleum ether/EtOAc) afforded the desired product **6o** as a colorless oil (12.7 mg, 32%).

^{1}H NMR (400 MHz, CDCl$_3$) δ 7.62 (d, *J* = 7.6 Hz, 1H), 7.51 (d, *J* = 8.0 Hz, 1H), 7.37 – 7.36 (m, 1H), 7.23 – 7.19 (m, 1H), 7.14 – 7.10 (m, 1H), 6.53 (d, *J* = 2.8 Hz, 1H), 5.73 (t, *J* = 7.2 Hz, 1H), 4.11 – 4.06 (m, 2H), 3.07 – 2.96 (m, 2H), 1.18 (t, *J* = 7.2 Hz, 3H), 1.07 (s, 21H).

^{13}C NMR (101 MHz, CDCl$_3$) δ 169.4, 134.8, 129.1, 126.0, 121.7, 121.1, 119.8, 109.8, 103.1, 102.2, 87.7, 61.1, 45.9, 42.2, 18.5, 14.0, 11.1.

HRMS (ESI) m/z: $[M + H]^+$ Calcd for $C_{24}H_{36}NO_2Si^+$ 398.2510; Found 398.2507.

HPLC: 90.5:9.5 er determined by analytical HPLC, Daicel CHIRALPAK® IB-3 column, 25 °C, Hexane:*i*-PrOH = 100:0, 1.0 mL/min, 254 nm, t_{major} = 4.6 min, t_{minor} = 7.1 min.

$[\alpha]_D^{20}$ = +4.2 (*c* = 0.21, CHCl$_3$).

(*S*)-1-(1-(6-Phenyl-1-(triisopropylsilyl)hex-1-yn-3-yl)-1*H*-indol-5-yl)ethan-1-one (6p)

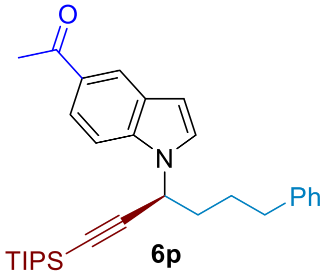

6p

Prepared according to GP2 with (*Z*)-1-(1-(4-phenylbut-1-en-1-yl)-1*H*-indol-5-yl)ethan-1-one **1t** (28.9 mg, 0.10 mmol, 1.0 equiv.) and **5a** (66.0 uL, 0.25 mmol, 2.5 equiv.); MeCN instead of DME as solvent and stirred at 0 ℃ for 48 hours. Flash column chromatography (SiO_2, 10:1 petroleum ether/EtOAc) afforded the desired product **6p** as a colorless oil (11.8 mg, 25%).

^{1}H NMR (500 MHz, $CDCl_3$) δ 8.28 (s, 1H), 7.86 (d, *J* = 8.5 Hz, 1H), 7.42 – 7.37 (m, 2H), 7.26 – 7.23 (m, 2H), 7.18 – 7.16 (m, 1H), 7.10 – 7.09 (m, 2H), 6.62 (d, *J* = 6.5 Hz, 1H), 5.23 (t, *J* = 7.0 Hz, 1H), 2.65 (s, 3H), 2.61 (t, *J* = 7.5 Hz, 2H), 2.05 – 2.02 (m, 2H), 1.77 – 1.74 (m, 2H), 1.08 (s, 21H).
^{13}C NMR (126 MHz, $CDCl_3$) δ 198.2, 141.4, 137.5, 129.8, 128.5, 128.4, 128.3, 127.6, 126.0, 123.4, 121.7, 109.5, 103.8, 103.4, 87.8, 49.5, 36.2, 35.0, 27.4, 26.6, 18.6, 11.1.
HRMS (ESI) m/z: $[M + H]^+$ Calcd for $C_{31}H_{42}NOSi^+$ 472.3030; Found 472.3033.
HPLC: 90:10 er determined by analytical HPLC, Daicel CHIRALPAK® OD-H column, 25 °C, Hexane:*i*-PrOH = 90:10, 1.0 mL/min, 254 nm, t_{major} = 6.8 min, t_{minor} = 9.7 min.
$[\alpha]_D^{20}$ = +5.7 (*c* = 0.64, $CHCl_3$).

(*S*)-1-(4-(*tert*-Butyldimethylsilyl)but-3-yn-2-yl)-1*H*-indole (6q)

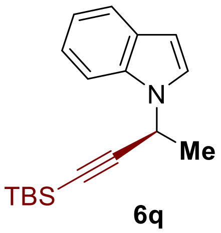

6q

Prepared according to **GP2** with 1-vinyl-1*H*-indole **1a** (14.3 mg, 0.10 mmol, 1.0 equiv.) and **5b** (55.0 uL, 0.25 mmol, 2.5 equiv.). Flash column chromatography (SiO_2, 100:0 petroleum ether/EtOAc) afforded the desired product **6r** as a colorless oil (20.9 mg, 74%).

^{1}H NMR (500 MHz, CDCl$_3$) δ 7.65 (d, *J* = 8.0 Hz, 1H), 7.44 – 7.39 (m, 2H), 7.24 – 7.21 (m, 1H), 7.15 – 7.12 (m, 1H), 6.54 (d, *J* = 2.0 Hz, 1H), 5.34 (q, *J* = 7.0 Hz, 1H), 1.73 (d, *J* = 7.0 Hz, 3H), 0.97 (s, 9H), 0.14 (s, 6H).

^{13}C NMR (126 MHz, CDCl$_3$) δ 134.9, 129.1, 125.3, 121.5, 121.1, 119.6, 109.5, 104.5, 101.7, 88.0, 44.4, 26.0, 23.1, 16.5, -4.7.

HRMS (ESI) m/z: [M + H]$^+$ Calcd for $C_{18}H_{26}NSi^+$ 284.1829; Found 284.1826.

HPLC: 5:95 er determined by analytical HPLC, Daicel CHIRALPAK® IB-3 column, 25 °C, Hexane:*i*-PrOH = 100:0, 1.0 mL/min, 254 nm, t_{minor} = 10.0 min, t_{major} = 12.2 min.

$[\alpha]_D^{20}$ = -16.2 (*c* = 0.36, CHCl$_3$).

(*S*)-1-(4-(1-((*tert*-Butyldimethylsilyl)oxy)cyclohexyl)but-3-yn-2-yl)-1*H*-indole (6r)

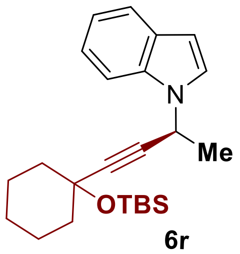

6r

Prepared according to **GP2** with 1-vinyl-1*H*-indole **1a** (14.3 mg, 0.10 mmol, 1.0 equiv.) and **5c** (79.0 uL, 0.25 mmol, 2.5 equiv.). Flash column chromatography (SiO_2, 100:0 petroleum ether/EtOAc) afforded the desired product **6t** as a colorless oil (18.0 mg, 47%).

^{1}H NMR (500 MHz, CDCl$_3$) δ 7.65 (d, *J* = 8.0 Hz, 1H), 7.43 – 7.38 (m, 1H), 7.24 – 7.21 (m, 1H), 7.14 – 7.11 (m, 1H), 6.54 (d, *J* = 3.0 Hz, 1H), 5.38 (q, *J* = 7.0 Hz, 1H), 1.80 – 1.77 (m, 2H), 1.73 (d, *J* = 7.0 Hz, 3H), 1.65 – 1.56 (m, 4H), 1.49 – 1.26 (m, 5H), 0.88 (s, 9H), -0.12 (s, 3H), -0.13 (s, 3H).

^{13}C NMR (126 MHz, CDCl$_3$) δ 134.9, 129.0, 125.1, 121.4, 121.1, 119.6, 109.4, 101.6, 89.3, 82.8, 69.3, 43.7, 41.1, 41.0, 25.8, 25.2, 22.8, 18.1, -2.78, -2.82 .

HRMS (ESI) m/z: [M + H]$^+$ Calcd for $C_{24}H_{36}NOSi^+$ 382.2561; Found 382.2565.

HPLC: 2:98 er determined by analytical HPLC, Daicel CHIRALPAK® IB-3 column, 25 °C, Hexane:*i*-PrOH = 100:0, 1.0 mL/min, 254 nm, t_{minor} = 11.0 min, t_{major} = 15.5 min.

$[\alpha]_D^{20}$ = -11.34 (*c* = 0.62, CHCl$_3$).

(*R*)-(5-(1-(1*H*-Indol-1-yl)ethyl)-2-methylphenyl)(5-(4-fluorophenyl)thiophen-2-yl)methanone (7a)

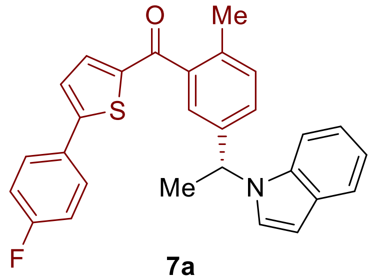

7a

Prepared according to **GP1** with *N*-vinylindole **1a** (56.0 uL, 0.40 mmol, 1.0 equiv.) and **2x** (300.2 mg, 0.80 mmol, 2.0 equiv.). Flash column chromatography (SiO_2, 15:1 PE/EtOAc) afforded the desired product 7**a** as a yellow solid (141.2 mg, 80%).

^{1}H NMR (600 MHz, $CDCl_3$) δ 7.63 (d, *J* = 7.2 Hz, 1H), 7.60 - 7.50 (m, 2H), 7.29 (d, *J* = 3.6 Hz, 1H), 7.24 – 7.17 (m, 3H), 7.15-7.08 (m, 5H), 7.05 (d, *J* = 3.6 Hz, 1H), 6.91 (d, *J* = 4.2 Hz, 1H), 6.54 (d, *J* = 3.6 Hz, 1H), 5.66 (q, *J* = 7.2 Hz, 1H), 2.34 (s, 3H), 1.91 (d, *J* = 7.2 Hz, 3H).
^{13}C NMR (151 MHz, $CDCl_3$) δ 189.6, 163.2 (d, $J_{C\text{-}F}$ = 250.1), 152.6, 143.1, 139.9, 138.2, 136.4, 135.9, 135.9, 131.5, 129.6, 129.6, 129.0, 128.2, 128.1, 127.8, 125.7, 124.8, 124.0, 121.5, 121.0, 119.6, 116.2 (d, $J_{C\text{-}F}$ = 21.9), 110.2, 101.7, 54.5, 21.5, 19.3.
^{19}F NMR (565 MHz, $CDCl_3$) δ -111.6.
HRMS (ESI) m/z: $[M + H]^+$ Calcd for $C_{28}H_{23}FNOS^+$ 440.1479; Found 440.1476.
HPLC: 5:95 er determined by analytical HPLC, Daicel CHIRALCEL® OD-H column, 35 °C, Hexane:*i*-PrOH = 90:10, 1.0 mL/min, 254 nm, t_{minor} = 29.9 min, t_{major} = 33.3 min.
$[\alpha]_D^{20}$ = +38.1 (*c* = 0.99, $CHCl_3$).

Methyl (*R*)-2-(1-(4-(1-(1*H*-indol-1-yl)ethyl)benzoyl)-5-methoxy-2-methyl-1*H*-indol-3-yl)acetate (7b)

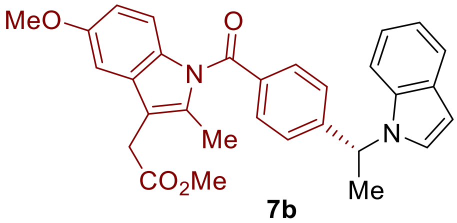

7b

Prepared according to **GP1** with *N*-vinylindole **1a** (56.0 uL, 0.40 mmol, 1.0 equiv.) and **2y** (281.1 mg, 0.80 mmol, 2.0 equiv.). Flash column chromatography (SiO_2, 2:1 PE/EtOAc) afforded the desired product **7b** as a green oil (155.2 mg, 81%).

^{1}H NMR (500 MHz, CDCl$_3$) δ 7.64 (d, *J* = 7.5 Hz, 1H), 7.60 (d, *J* = 8.0 Hz, 2H), 7.31 (d, *J* = 3.0 Hz, 1H), 7.21 - 7.08 (m, 5H), 6.93 (s, 1H), 6.82 (d, *J* = 9.0 Hz, 1H), 6.66 – 6.55 (m, 2H), 5.71 (q, *J* = 7.0 Hz, 1H), 3.82 (s, 3H), 3.68 (s, 3H), 3.64 (s, 2H), 2.32 (s, 3H), 1.96 (d, *J* = 7.0 Hz, 3H).

^{13}C NMR (126 MHz, CDCl$_3$) δ 171.3, 168.9, 155.9, 147.8, 135.91, 135.88, 134.7, 130.9, 130.5, 130.1, 128.9, 126.2, 124.6, 121.6, 121.0, 119.8, 114.9, 112.2, 111.5, 109.9, 102.0, 101.2, 55.6, 54.7, 52.1, 30.1, 21.5, 13.3 .

HRMS (ESI) m/z: $[M + H]^+$ Calcd for $C_{30}H_{29}N_2O_4^+$ 481.2122; Found 481.2121.

HPLC: 4:96 er determined by analytical HPLC, Daicel CHIRALCEL® OD-H column, 35 °C, Hexane:*i*-PrOH = 65:35, 1.0 mL/min, 254 nm, t_{minor} = 33.0 min, t_{major} = 37.6 min.

$[\alpha]_D^{20}$ = +73.7 (*c* = 1.62, CHCl$_3$).

(*R*)-2,5,7,8-tetramethyl-2-((4*R*,8*R*)-4,8,12-trimethyltridecyl)chroman-6-yl 3-((*R*)-1-(1*H*-indol-1-yl)ethyl)benzoate (7c)

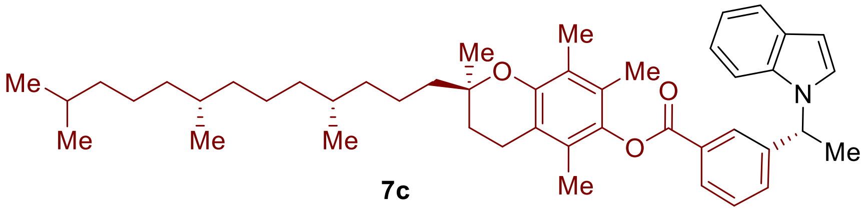

7c

Prepared according to **GP1** with *N*-vinylindole **1a** (27.0 uL, 0.20 mmol, 1.0 equiv.) and **2z** (245.2 mg, 0.40 mmol, 2.0 equiv.), (*S*,*S*)-**L*1** as ligand. Flash column chromatography (SiO$_2$, 10:1 PE/EtOAc) afforded the desired product **7c** as a colorless oil (107.7 mg, 80%).

^{1}H NMR (500 MHz, CDCl$_3$) δ 8.20 – 8.07 (m, 2H), 7.65 (d, *J* = 7.5 Hz, 1H), 7.39 (t, *J* = 8.0 Hz, 1H), 7.34 (d, *J* = 3.0 Hz, 1H), 7.28-7.23 (m, 3H), 7.17 - 7.06 (m, 2H), 6.60 (d, *J* = 3.0 Hz, 1H), 5.76 (q, *J* = 7.0 Hz, 1H), 2.65-2.60 (m, 2H), 2.12 (s, 3H), 2.03 (d, *J* = 10.0 Hz, 3H), 1.99 (d, *J* = 7.5 Hz, 6H), 1.65 – 1.48 (m, 5H), 1.34 - 1.20 (m, 12H), 1.18 - 1.00 (m, 7H), 0.92 – 0.77 (m, 13H).

^{13}C NMR (126 MHz, CDCl$_3$) δ 164.9, 149.5, 143.5, 140.5, 136.0, 130.9, 130.1, 129.2, 129.1, 128.9, 127.7, 126.9, 125.1, 124.6, 123.2, 121.6, 121.0, 119.7, 117.5, 109.9, 101.9, 75.1, 54.6, 40.5, 39.6, 39.4, 37.5, 37.3, 32.8, 32.7, 28.0, 24.8, 24.5, 24.2, 23.7, 22.7, 22.6, 21.7, 21.0, 20.6, 19.8, 19.7, 13.1, 12.2, 11.9.

HRMS (ESI) m/z: $[M + H]^+$ Calcd for $C_{46}H_{64}NO_3^+$ 678.4881; Found 678.4882.

HPLC: 3:97 er determined by analytical HPLC, Daicel CHIRALCEL® OD-3 column, 30 °C, Hexane:*i*-PrOH = 90:10, 1.0 mL/min, 254 nm, t_{minor} = 5.9 min, t_{major} = 6.8 min.

$[\alpha]_D^{20}$ = +26.6 (c = 0.71, $CHCl_3$).

(*R*)-2,5,7,8-tetramethyl-2-((4*R*,8*R*)-4,8,12-trimethyltridecyl)chroman-6-yl 3-((*S*)-1-(1H-indol-1-yl)ethyl)benzoate (7c')

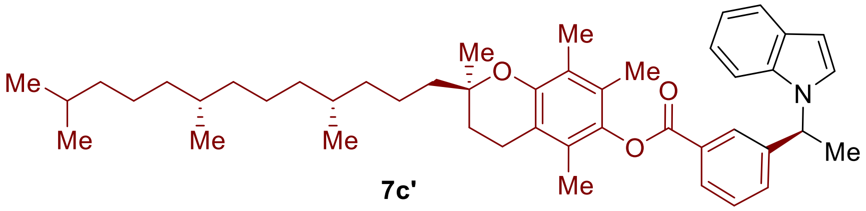

7c'

Prepared according to **GP1** with *N*-vinylindole **1a** (56.0 uL, 0.40 mmol, 1.0 equiv.) and **2z** (172 mg, 0.80 mmol, 2.0 equiv.), (*R*,*R*)-**L*1** as ligand. Flash column chromatography (SiO_2, 4:1 Hexane/EtOAc) afforded the desired product **7c'** as a colorless oil (123.4 mg, 91%).

^{1}H NMR (400 MHz, $CDCl_3$) δ 8.24 – 8.07 (m, 2H), 7.64 (d, J = 7.6 Hz, 1H), 7.39 (t, J = 7.6 Hz, 1H), 7.33 (d, J = 3.2 Hz, 1H), 7.26 – 7.22 (m, 2H), 7.18 - 7.04 (m, 2H), 6.59 (d, J = 3.2 Hz, 1H), 5.76 (q, J = 7.2 Hz, 1H), 2.61-2.55 (m, 2H), 2.12 (s, 3H), 2.06 - 1.92 (m, 9H), 1.87 - 1.74 (m, 2H), 1.63 – 1.47 (m, 4H), 1.35 - 1.18 (m, 13H), 1.17 – 1.04 (m, 6H), 0.96 - 0.80 (m, 13H).

^{13}C NMR (101 MHz, $CDCl_3$) δ 164.9, 149.6, 143.5, 140.6, 136.1, 130.9, 130.1, 129.3, 129.2, 128.9, 127.7, 126.9, 125.1, 124.7, 123.2, 121.7, 121.1, 119.8, 117.5, 110.0, 102.0, 75.1, 54.6, 40.5, 39.4, 37.5, 37.3, 32.9, 31.3, 31.1, 28.0, 24.9, 24.5, 24.3, 23.7, 22.8, 22.7, 21.8, 21.1, 20.7, 19.8, 19.7, 13.1, 12.3, 11.9.

HRMS (ESI) m/z: $[M + H]^+$ Calcd for $C_{46}H_{64}NO_3^+$ 678.4881; Found 678.4882.

HPLC: 96:4 dr determined by analytical HPLC, Daicel CHIRALCEL® OD-3 column, 30 °C, Hexane:*i*-PrOH = 90:10, 1.0 mL/min, 254 nm, t_{major} = 5.8 min, t_{minor} = 6.8 min.

$[\alpha]_D^{20}$ = -17.3 (c = 0.30, $CHCl_3$).

((*3aR,5R,5aS,8aS,8bR*)-2,2,7,7-Tetramethyltetrahydro-5*H*-bis([1,3]dioxolo)[4,5-b:4',5'-d]pyran-5-yl)methyl 4-((*R*)-1-(1*H*-indol-1-yl)ethyl)benzoate (7d)

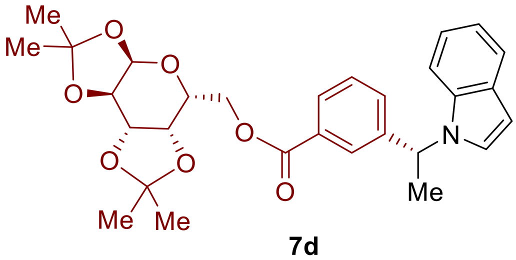

7d

Prepared according to **GP1** with *N*-vinylindole **1a** (27.0 uL, 0.20 mmol, 1.0 equiv.) and **2aa** (175.7 mg, 0.40 mmol, 2.0 equiv.), (*S*,*S*)-**L*1** as ligand. Flash column chromatography (SiO_2, 12:1 PE/EtOAc) afforded the desired product 7**d** as a colorless oil (72 mg, 71%).

^{1}H NMR (500 MHz, $CDCl_3$) δ 8.09 – 7.75 (m, 2H), 7.63 (d, *J* = 7.5 Hz, 1H), 7.30 (q, *J* = 7.0 Hz, 2H), 7.18 (d, *J* = 8.0 Hz, 2H), 7.17-7.06 (m, 2H), 6.57 (d, *J* = 3.5 Hz, 1H), 5.70 (q, *J* = 7.5 Hz, 1H), 5.56 (s, 1H), 4.71 - 4.59 (m, 1H), 4.53 - 4.45 (m, 1H), 4.45 - 4.38 (m, 1H), 4.38 - 4.31 (m, 1H), 4.25 (d, *J* = 8.0 Hz, 1H), 4.15 (d, *J* = 6.5 Hz, 1H), 1.94 (d, *J* = 7.0 Hz, 3H), 1.52 (s, 3H), 1.47 (s, 3H), 1.34 (s, 6H).

^{13}C NMR (126 MHz, $CDCl_3$) δ 166.1, 143.2, 135.9, 130.4, 128.9, 128.8, 127.2, 124.6, 121.6, 120.9, 119.6, 109.9, 109.6, 108.8, 101.8, 96.3, 71.04, 70.7, 70.5, 66.1, 63.9, 54.5, 26.0, 25.9, 25.0, 24.5, 21.6.

HRMS (ESI) m/z: $[M + H]^+$ Calcd for $C_{29}H_{34}NO_7^+$ 508.2330; Found 508.2328.

HPLC: 18:82 dr determined by analytical HPLC, Daicel CHIRALCEL® OD-H column, 30 °C, Hexane:*i*-PrOH = 90:10, 1.0 mL/min, 254 nm, t_{minor} = 26.6 min, t_{major} = 30.5 min.

$[\alpha]_D^{20}$ = -13.7 (*c* = 0.69, $CHCl_3$).

((*3aR,5R,5aS,8aS,8bR*)-2,2,7,7-Tetramethyltetrahydro-5*H*-bis([1,3]dioxolo)[4,5-b:4',5'-d]pyran-5-yl)methyl 4-((*S*)-1-(1*H*-indol-1-yl)ethyl)benzoate (7d')

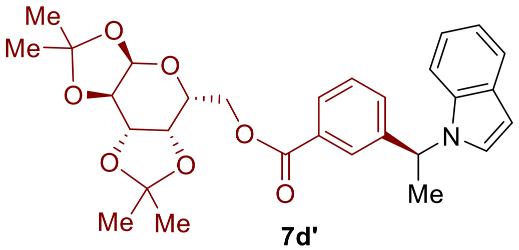

7d'

Prepared according to **GP1** with *N*-vinylindole **1a** (27.0 uL, 0.20 mmol, 1.0 equiv.) and **2aa** (175.7 mg, 0.40 mmol, 2.0 equiv.), (*R*,*R*)-**L*1** as ligand. Flash column chromatography (SiO_2, 12:1 PE/EtOAc) afforded the desired product 7**d'** as a colorless oil (77.1mg, 77%).

^{1}H NMR (400 MHz, $CDCl_3$) δ 8.03 – 7.83 (m, 2H), 7.63 (d, *J* = 7.2 Hz, 1H), 7.35 – 7.27 (m, 2H), 7.23 – 7.15 (m, 2H), 7.15 - 7.02 (m, 2H), 6.58 (d, *J* = 3.2 Hz, 1H), 5.70 (q, *J* = 7.2 Hz, 1H), 5.56 (d, *J* = 5.0 Hz, 1H), 4.72 -4.58 (m, 1H), 4.56 - 4.47 (m, 1H), 4.47 - 4.36 (m, 1H), 4.38 - 4.31 (m, 1H), 4.31 - 4.22 (m, 1H), 4.16 (t, *J* = 6.4 Hz, 1H), 1.94 (d, *J* = 7.2 Hz, 3H), 1.50 (s, 3H), 1.48 (s, 3H), 1.36 (s, 3H), 1.33 (s, 3H).

^{13}C NMR (101 MHz, CDCl$_3$) δ 166.1, 143.2, 135.9, 130.5, 128.9, 128.8, 127.2, 124.6, 121.6, 120.9, 119.6, 109.9, 109.7, 108.8, 101.8, 96.3, 71.1, 70.7, 70.5, 66.1, 64.0, 54.5, 26.00, 25.95, 25.0, 24.5, 21.6.

HRMS (ESI) m/z: $[M + H]^+$ Calcd for $C_{29}H_{34}NO_7^+$ 508.2330; Found 508.2328..

HPLC: 91.5:8.5 dr determined by analytical HPLC, Daicel CHIRALCEL® OD-H column, 30 °C, Hexane:*i*-PrOH = 90:10, 1.0 mL/min, 254 nm, t_{major} = 25.7 min, t_{minor} = 32.8 min.

$[\alpha]_D^{20}$ = −82.2 (*c* = 1.4, CHCl$_3$).

Methyl 4-((1*R*)-1-(1*H*-indol-1-yl)-4,8-dimethylnon-7-en-1-yl)benzoate (7e)

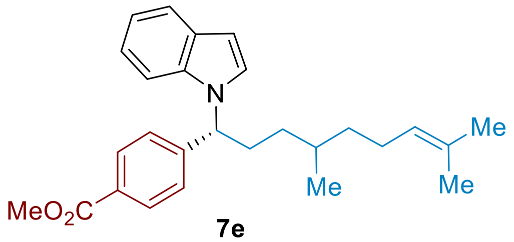

7e

Prepared according to **GP1** with **1u** (106.7 mg, 0.40 mmol, 1.0 equiv.) and **2d** (171.8 mg, 0.80 mmol, 2.0 equiv.), $(OEt)_2MeSiH$ (115.4 uL, 0.72 mmol, 1.8 equiv.), and KF (51.4 mg, 0.88 mmol, 2.2 equiv.), (*S*,*S*)-**L*1** as ligand. DME as solvent, stirred at room temperature for 48 hours. Flash column chromatography (SiO_2, 10:1 PE/EtOAc) afforded the desired product **7e** as a colorless oil (65.1 mg, 40%).

^{1}H NMR (600 MHz, CDCl$_3$) δ 7.93 (d, *J* = 7.8 Hz, 2H), 7.63 (d, *J* = 7.8 Hz, 1H), 7.32 (d, *J* = 3.0 Hz, 1H), 7.22 (t, *J* = 9.0 Hz, 3H), 7.13 (t, *J* = 7.8 Hz, 1H), 7.08 (t, *J* = 7.2 Hz, 1H), 6.60 (d, *J* = 3.0 Hz, 1H), 5.44 (q, *J* = 6.6 Hz, 1H), 5.09 - 4.99 (m, 1H), 3.87 (s, 3H), 2.40 – 2.19 (m, 2H), 1.99 - 1.84 (m, 2H), 1.66 (d, *J* = 11.4 Hz, 3H), 1.56 (d, *J* = 12.0 Hz, 3H), 1.52 – 1.43 (m, 1H), 1.40 - 1.28 (m, 2H), 1.24 – 1.05 (m, 2H), 0.92 - 0.85 (m, 3H).

^{13}C NMR (151 MHz, CDCl$_3$) δ 166.7, 147.0, 136.4, 131.3, 130.0, 129.4, 128.7, 126.3, 124.58, 124.55, 121.6, 121.0, 119.7, 109.7, 102.1, 59.8, 52.1, 36.9, 33.8, 32.8, 32.2, 25.7, 25.4, 19.6, 17.6.

HRMS (ESI) m/z: $[M + H]^+$ Calcd for $C_{27}H_{34}NO_2^+$ 404.2584; Found 404.2589.

HPLC: 11:89 dr determined by analytical HPLC, Daicel CHIRALCEL® IB-H column, 20 °C, Hexane:*i*-PrOH = 90:10, 1.0 mL/min, 254 nm, t_{minor} = (t_1 = 5.6 min, t_2 = 7.3 min), t_{major} =(t_3 = 8.5 min, t_4 = 10.3 min) .

$[\alpha]_D^{25}$ = +64.1 (*c* = 0.6, CHCl$_3$).

Methyl 4-((1*S*)-1-(1*H*-indol-1-yl)-4,8-dimethylnon-7-en-1-yl)benzoate (7e')

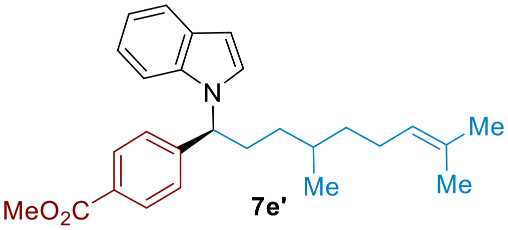

7e'

Prepared according to **GP1** with **1u** (36.0 uL, 0.20 mmol, 1.0 equiv.) and **2d** (172 mg, 0.80 mmol, 2.0 equiv.), $(OEt)_2MeSiH$ (74.8 uL, 0.72 mmol, 1.8 equiv.), and KF (51.4 mg, 0.88mmol, 2.2 equiv.); (*R*,*R*)-**L*1** as ligand, DME as solvent, stirred at room temperature for 48 hours. Flash column chromatography (SiO_2, 10:1 PE/EtOAc) afforded the desired product **7e'** as a colorless oil (42.8 mg, 27%).

^{1}H NMR (400 MHz, $CDCl_3$) δ 7.93 (d, *J* = 7.6 Hz, 2H), 7.63 (d, *J* = 7.6 Hz, 1H), 7.32 (d, *J* = 3.2 Hz, 1H), 7.25 – 7.19 (m, 3H), 7.17 – 7.02 (m, 2H), 6.60 (d, *J* = 2.4 Hz, 1H), 5.50 – 5.39 (m, 1H), 5.11 – 4.97 (m, 1H), 3.87 (s, 3H), 2.44 – 2.13 (m, 2H), 2.04 - 1.78 (m, 2H), 1.66 (d, *J* = 7.6 Hz, 3H), 1.56 (d, *J* = 7.6 Hz, 3H), 1.52 – 1.44 (m, 1H), 1.39 – 1.27 (m, 2H), 1.23 – 1.07 (m, 2H), 0.88 (t, *J* = 6.8 Hz, 3H).

^{13}C NMR (101 MHz, $CDCl_3$) δ 166.7, 147.1, 136.4, 131.3, 130.0, 129.4, 128.7, 126.3, 124.6, 124.5, 121.6, 121.0, 119.7, 109.7, 102.1, 59.9, 52.0, 36.6, 33.8, 32.8, 32.2, 25.7, 25.4, 19.6, 17.6.

HRMS (ESI) m/z: $[M + H]^+$ Calcd for $C_{27}H_{34}NO_2^+$ 404.2584; Found 404.2589.

HPLC: 90:10 dr determined by analytical HPLC, Daicel CHIRALCEL® IB-H column, 20 °C, Hexane:*i*-PrOH = 90:10, 1.0 mL/min, 254 nm, t_{major} = (t_1 = 5.7 min, t_2 = 7.3 min), t_{minor} =(t_3 = 8.7 min, t_4 = 10.5 min) .

$[\alpha]_D^{25}$ = -56.7 (*c* = 0.5, $CHCl_3$).

1.6 Competition Experiment of (*Z*)- and (*E*)-*N*-Alkenyl Indoles

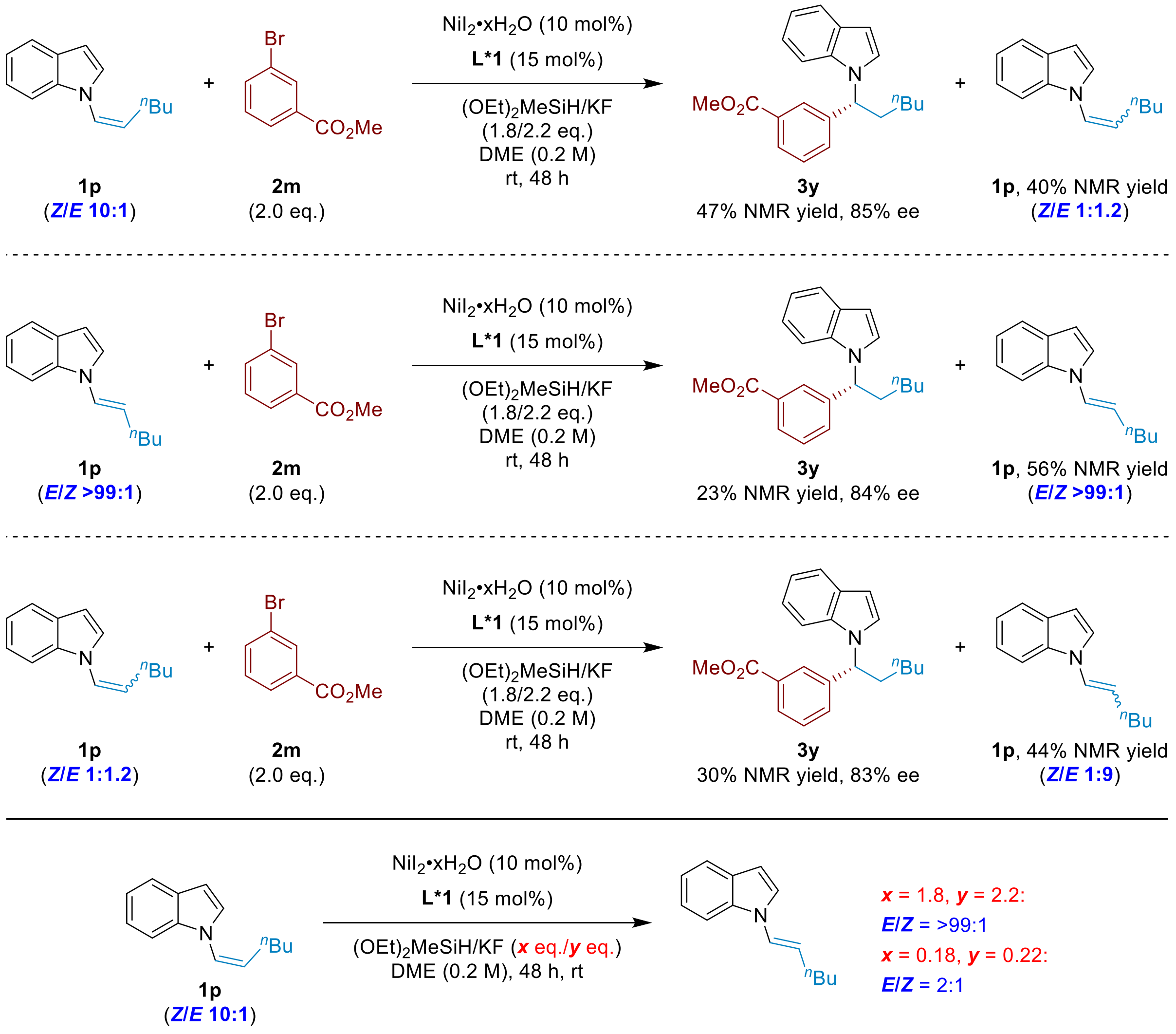

1.7 Gram-Scale Experiment

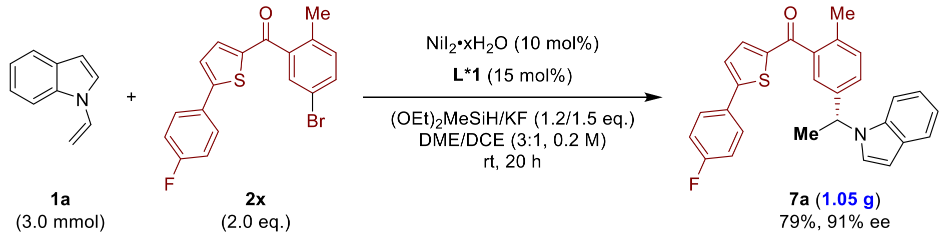

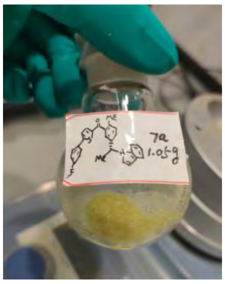

To an oven-dried 50 mL round bottom flask containing a magnetic stir was charged with NiI_2•xH_2O (126.0 mg, 10 mol%) and ligand **L*1** (151.5 mg, 15 mol%) under a nitrogen (N_2)

atmosphere using glove-box techniques. Subsequently, anhydrous DME (11.2 mL) was added, and the mixture was stirred for 15 minutes at room temperature. Then, KF (262.5 mg, 4.5 mmol, 1.5 equiv.), *N*-alkenyl indole **1a** (0.42 mL, 3.0 mmol, 1.0 equiv), alryl bromide **2x** (2.25 g, 6.0 mmol, 2.0 equiv.), DCE (3.8 mL), and $(OEt)_2MeSiH$ (0.59 mL, 3.6 mmol, 1.2 equiv.) were sequentially added. Afterwards, the flask was sealed with airtight electrical tapes and removed from the glove box and stirred at room temperature for 20 hours at 650 rpm. After the reaction was completed, the reaction mixture was diluted with saturated NH_4Cl (aq., 7.5 mL) and EtOAc (40.0 mL). The aqueous phase was extracted with EtOAc (2 x 40.0 mL) and the combined organic phases were concentrated in vacuo. The crude mixture was purified by flash column chromatography on silica gel using a mixture of PE/EtOAc (15:1) as eluent to provide **7a** as a yellow solid in 79% yield (1.05 g). The ee (91%) was determined via HPLC analysis (**General procedure 1**).

1.8 Derivatization of Coupling Products

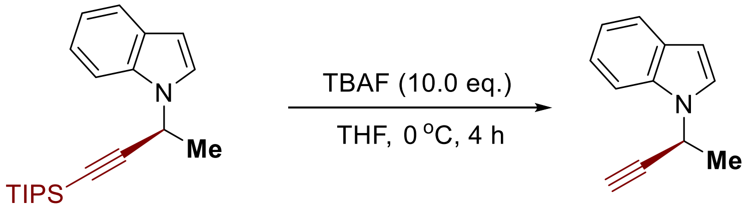

6a **8**

The compound **6a** (65.2 mg, 0.20 mmol) was dissolved in THF (2.0 mL) and the solution was cooled to 0 °C. Then, a solution of tetrabutylammonium fluoride (1.0 M in THF, 2.0 mL, 2.00 mmol, 10.0 equiv.) was added and stirred for 4 h. After a complete conversion (monitored by TLC), the mixture was poured into H_2O (4.0 mL) and extracted with EtOAc (3 x 4.0 mL). The combined organic phases were washed with brine (4.0 mL), dried (Na_2SO_4). After removal of the solvent, the resulting was purified by flash chromatography (SiO_2, 100:0 petroleum ether/EtOAc) to yield **8** (24.0 mg, 71%) as a colorless oil.

^{1}H NMR (400 MHz, $CDCl_3$) δ 7.67 (d, *J* = 8.0 Hz, 1H), 7.46 (d, *J* = 8.4 Hz, 1H), 7.39 (d, *J* = 3.2 Hz, 1H), 7.28 – 7.24 (m, 1H), 7.18 – 7.14 (m, 1H), 6.58 (d, *J* = 3.2 Hz, 1H), 5.34 (q, *J* = 6.8, 1H), 2.51 (d, *J* = 2.4 Hz, 1H), 1.78 (d, *J* = 7.2 Hz, 3H).

^{13}C NMR (101 MHz, $CDCl_3$) δ 135.3, 129.3, 125.3, 121.9, 121.5, 120.0, 109.7, 102.3, 82.5, 73.1, 43.7, 22.8.

HRMS (ESI) m/z: $[M + H]^+$ Calcd for $C_{12}H_{12}N^+$ 170.0964; Found 170.0969.

HPLC: 5:95 er determined by analytical HPLC, Daicel CHIRALPAK® IC-3 column, 25 °C, Hexane:*i*-PrOH = 99:1, 1.0 mL/min, 254 nm, t_{minor} = 4.9 min, t_{major} = 5.3 min.

$[\alpha]_D^{20}$ = -26.1 (*c* = 0.38, $CHCl_3$).

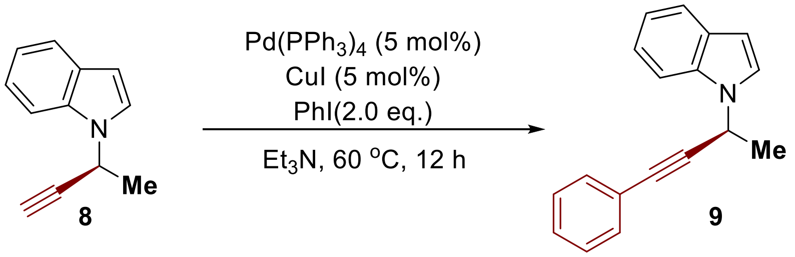

8 9

A mixture of **8** (33.8 mg, 0.20 mmol), iodobenzene (81.6 mg, 0.40 mmol, 2.0 equiv.), $Pd(PPh_3)_4$ (11.6 mg, 0.01 mmol), and CuI (1.9 mg, 0.01 mmol) in Et_3N (0.4 mL) was stirred at 60 ℃ for 12 h. The mixture was passed through a short column of silica gel with EtOAc and the mixture was concentrated on a rotary evaporator. The residue was subjected to a column chromatography on silica gel (100:1 petroleum ether/EtOAc) to give **9** (40.2 mg, 82%) as a colorless oil.

^{1}H NMR (500 MHz, $CDCl_3$) δ 7.66 (d, *J* = 8.0 Hz, 1H), 7.51 (d, *J* = 8.5 Hz, 1H), 7.45 – 7.44 (m, 3H), 7.32 – 7.29 (m, 3H), 7.25 – 7.23 (m, 1H), 7.15 – 7.12 (m, 1H), 6.55 (d, *J* = 3.0 Hz, 1H), 6.54 (q, *J* = 7.0 Hz, 1H), 1.83 (d, *J* = 7.0 Hz, 3H).

^{13}C NMR (126 MHz, $CDCl_3$) δ 135.0, 131.7, 129.0, 128.5, 128.3, 125.3, 122.4, 121.6, 121.1, 119.6, 109.6, 101.8, 87.6, 84.6, 44.2, 22.9.

HRMS (ESI) m/z: $[M + H]^+$ Calcd for $C_{18}H_{16}N^+$ 246.1277; Found 246.1279.

HPLC: 95:5 er determined by analytical HPLC, Daicel CHIRALPAK® IB-3 column, 25 °C, Hexane:*i*-PrOH = 99:1, 1.0 mL/min, 254 nm, t_{major} = 7.0 min, t_{minor} = 7.6 min.

$[\alpha]_D^{20}$ = -36.5 (*c* = 0.17, $CHCl_3$).

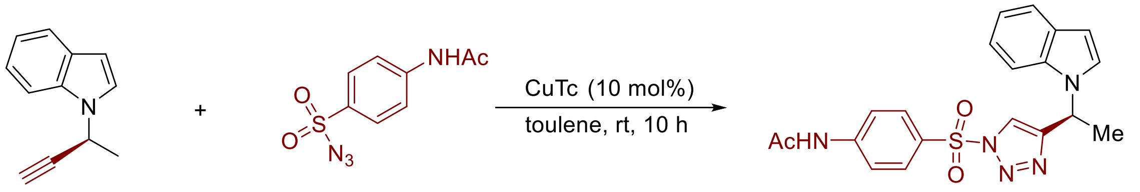

8 10

A solution of CuTc (3.8 mg, 0.02 mmol) and **8** (50.7 mg, 0.30 mmol) in toluene (2.0 mL), 4-acetamidobenzenesulfonyl azide (48.0 mg, 0.20 mmol) was added, and the mixture was stirred at room temperature for 10 h. When the reaction was complete, concentration and

gradient flash chromatography (SiO_2, 1:1 petroleum ether/EtOAc) afforded **10** (80.2 mg, 98%) as a white solid.

^{1}H NMR (400 MHz, $CDCl_3$) δ 7.94 – 7.89 (m, 3H), 7.68 – 7.62 (m, 4H), 7.27 – 7.26 (m, 1H), 7.26 - 7.20 (m, 1H), 7.18 – 7.09 (m, 2H), 6.57 (d, J = 3.2 Hz, 1H), 5.84 (q, J = 7.2 Hz, 1H), 2.16 (s, 3H), 1.99 (d, J = 7.2 Hz, 3H).

^{13}C NMR (101 MHz, $CDCl_3$) δ 169.0, 149.3, 144.9, 135.5, 130.3, 129.2, 128.7, 124.2, 121.9, 121.3, 120.8, 120.0, 119.5, 109.3, 102.8, 47.6, 24.7, 20.2.

HRMS (ESI) m/z: $[M + H]^+$ Calcd for $C_{20}H_{20}N_5O_3S^+$ 410.1281; Found 420.1281.

HPLC: 96:4 er determined by analytical HPLC, Daicel CHIRALPAK® IC column, 25 °C, Hexane:*i*-PrOH = 80:20, 1.0 mL/min, 254 nm, t_{major} = 16.8 min, t_{minor} = 19.5 min.

$[\alpha]_D^{20}$ = -75.7 (c = 0.15, $CHCl_3$).

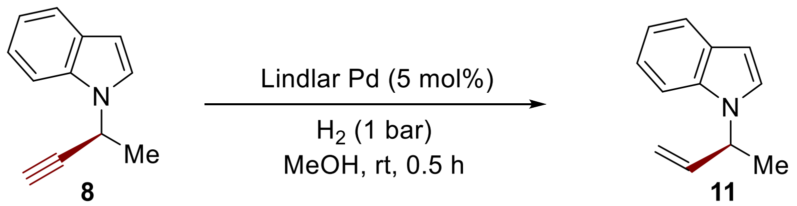

To a solution of **8** (33.8 mg, 0.2 mmol) in 2.0 mL of anhydrous MeOH, Lindlar catalyst ($Pd/BaSO_4$, 5% Pd; 0.01 mmol, 1.1 mg) were added. The mixture was submitted to a hydrogen atmosphere (1 bar) and stirred at room temperature for 0.5 h. The mixture was filtered on Celite and washed with ethyl acetate. The organic layer was washed with NH_4Cl (3.0 mL) and water, dried over Na_2SO_4, filtered and concentrated. The mixture was concentrated on a rotary evaporator and the residue was purified by column chromatography (100:1PE/EtOAc) to give product **11** (23.6 mg, 69%) as a colorless oil.

^{1}H NMR (500 MHz, $CDCl_3$) δ 7.65 (d, J = 8.0 Hz, 1H), 7.37 (d, J = 8.0 Hz, 1H), 7.22 – 7.10 (m, 3H), 6.54 (d, J = 3.0 Hz, 1H), 6.08-6.02 (m, 1H), 5.19 (d, J = 10.5 Hz,1H), 5.10 – 5.07 (m, 2H), 1.66 (d, J = 6.9 Hz, 3H).

^{13}C NMR (126 MHz, $CDCl_3$) δ 138.7, 135.7, 128.7, 124.6, 121.3, 120.9, 119.4, 115.5, 109.7, 101.4, 53.0, 19.7.

HRMS (ESI) m/z: $[M + H]^+$ Calcd for $C_{12}H_{14}N^+$ 172.1121; Found 172.1125.

HPLC: 95:5 er determined by analytical HPLC, Daicel CHIRALPAK® OD-H column, 25 °C, Hexane:*i*-PrOH = 99:1, 1.0 mL/min, 254 nm, t_{major} = 7.7 min, t_{minor} = 8.6 min.

$[\alpha]_D^{20}$ = -18.7 (c = 0.24, $CHCl_3$).

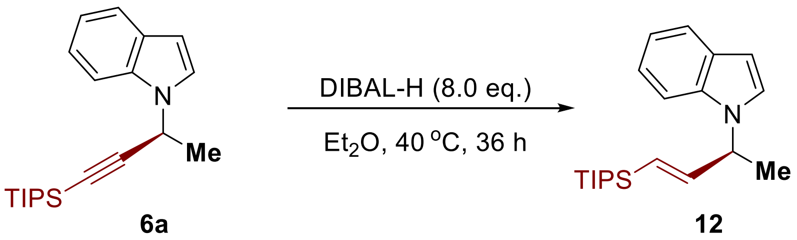

To a solution of **6a** (65.2 mg, 0.20 mmol) in Et_2O (2.0 mL), DIBAL-H (1.6 mL, 1.0 M solution in hexanes) was added, then the mixture was stirred at 40 °C for 36 h. After the reaction was completed, aqueous solution of NaOH was added to quench extra DIBAL-H, and the solution was extracted by Et_2O. The combined organic layer was dried over Na_2SO_4, and filtered. The mixture was concentrated on a rotary evaporator and the residue was purified by column chromatography (petroleum ether) to give product **12** (42.6 mg, 65%) as a colorless oil.

^{1}H NMR (400 MHz, $CDCl_3$) δ 7.69 (d, J = 8.0 Hz, 1H), 7.45 (d, J = 8.4 Hz, 1H), 7.31 – 7.24 (m, 2H), 7.18 – 7.14 (m, 1H), 7.02-6.97 (m, 1H), 6.57 (d, J = 3.2 Hz, 1H), 5.80 (d, J = 14.4 Hz, 1H), 5.14 – 5.07 (m, 1H), 1.62 (d, J = 6.8 Hz, 3H), 1.27 – 1.18 (m, 3H), 1.14 – 1.12 (m, 9H), 1.02 – 1.00 (m, 9H).

^{13}C NMR (101 MHz, $CDCl_3$) δ 147.6, 135.2, 128.6, 128.0, 124.1, 121.1, 120.9, 119.2, 109.7, 101.3, 54.3, 22.4, 18.8, 18.7, 12.1.

HRMS (ESI) m/z: $[M + H]^+$ Calcd for $C_{21}H_{34}NSi^+$ 328.2455; Found 328.2455.

HPLC: 95:5 er determined by analytical HPLC, Daicel CHIRALPAK® IB-3 column, 25 °C, Hexane:*i*-PrOH = 100:0, 1.0 mL/min, 254 nm, t_{major} = 9.1 min, t_{minor} = 9.7 min.

$[\alpha]_D^{20}$ = -12.8 (c = 0.48, $CHCl_3$).

1.9 Mechanistic Study

1.9.1 Initial-rate kinetic measurements

1) General procedure for kinetic experiments

The reactions used for kinetic analysis were set up in a nitrogen-filled glove box. A mixture of NiI_2•xH_2O and (*S,S*)-**L*1** in DME (0.75 mL) were stirred for 15 min in an oven-dried 8 mL Teflon-screw cap test tube at room temperature. Then, *N*-vinylindole **1a** (0.2 mmol), aryl bromide **2o** (2.0 eq.), and KF (1.5 eq.), DCE (0.25 mL) and $(OEt)_2MeSiH$ (1.2 eq.) were subsequently added to the reaction mixture. Reaction progress was monitored by NMR analysis of reaction aliquots taken at 30 min intervals for approximately 360 min. The initial reaction rate was determined by plotting product formation over time (up to approximately 20% conversion). This reaction progress plot used to determine d[P]/dt for this is provided below.

2) Rate order determination of NiI_2•xH_2O and (*S,S*)-L*1

Supplementary Table 8. Rates determined by varying [NiI_2•xH_2O] and [(*S,S*)-**L*1**] (1:1.5 ratio)

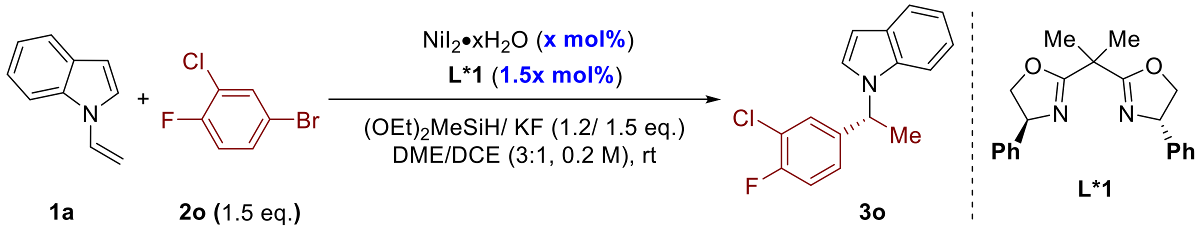

[L*1Ni] (M)	Average Rate (M/min)
0.0050	3.90×10^{-3}
0.010	4.33×10^{-3}
0.015	5.31×10^{-3}
0.020	6.06×10^{-3}

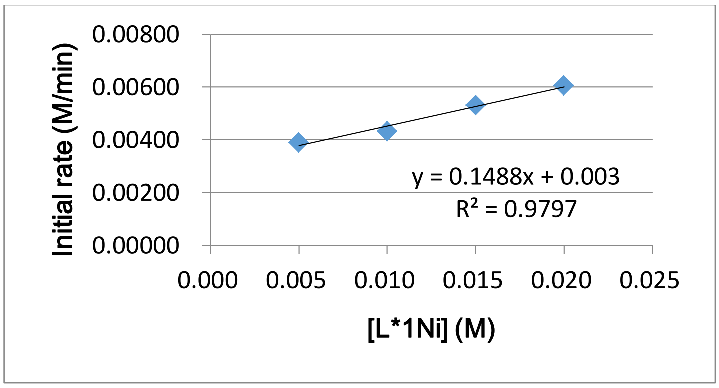

Supplementary Table 9. Rates dependence on [NiI_2•xH_2O] with constant [(*S*,*S*)-**L*1**]

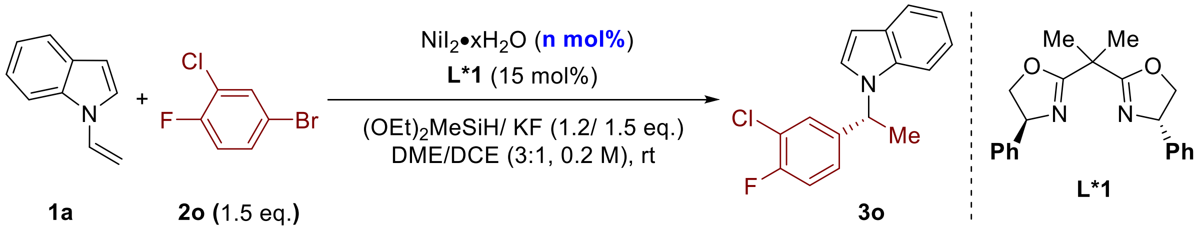

[NiI_2•xH_2O] (M)	Average Rate (M/min)
0.010	2.19×10^{-3}
0.020	6.20×10^{-3}
0.030	7.03×10^{-3}
0.040	9.08×10^{-3}

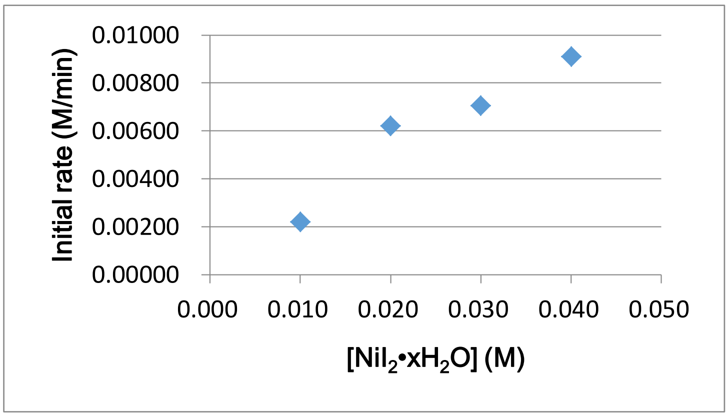

Supplementary Table 10. Rates dependence on [(*S,S*)-**L*1**] with constant [NiI_2•xH_2O]

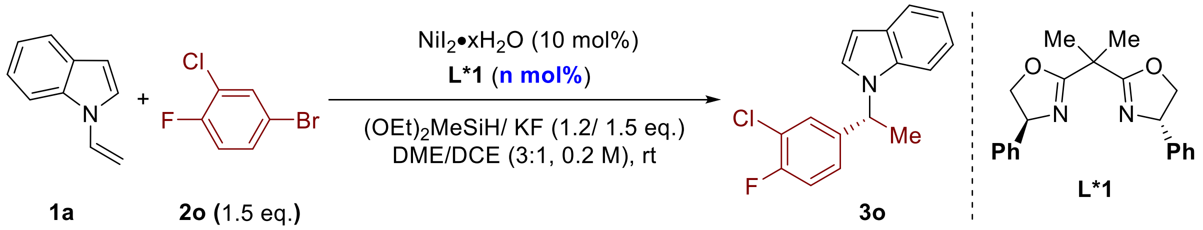

[L1*] (M)	Average Rate (M/min)
0.010	8.70 x 10^{-3}
0.020	4.06 x 10^{-3}
0.030	5.88 x 10^{-3}
0.040	8.44 x 10^{-3}

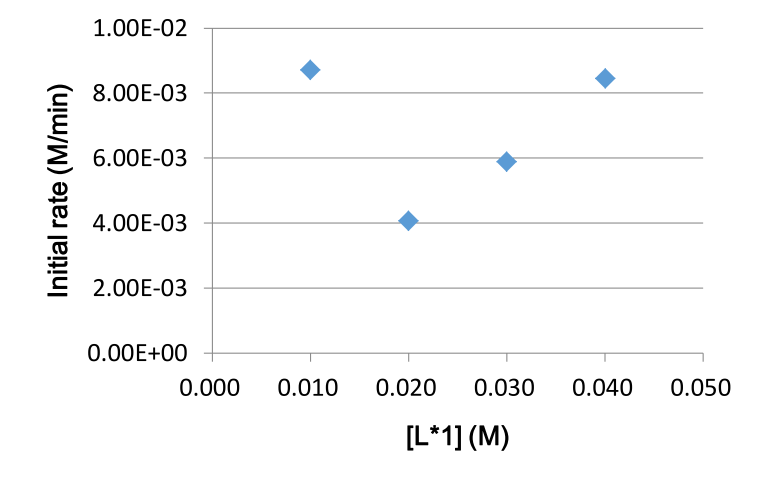

3) Rate order determination of *N*-vinylindole 1a

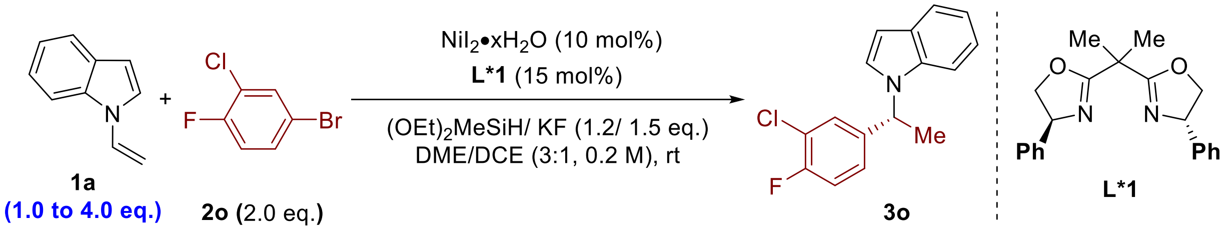

Supplementary Table 11. Rates determined by varying [*N*-vinylindole]

[*N*-vinylindole] (M)	Average Rate (M/min)
0.20	8.30×10^{-3}
0.40	8.32×10^{-3}
0.60	8.31×10^{-3}
0.80	8.29×10^{-3}

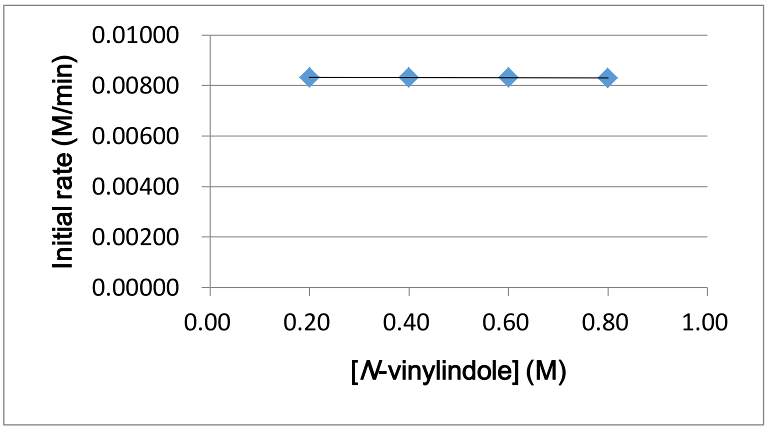

4) Rate order determination of aryl bromide 2n

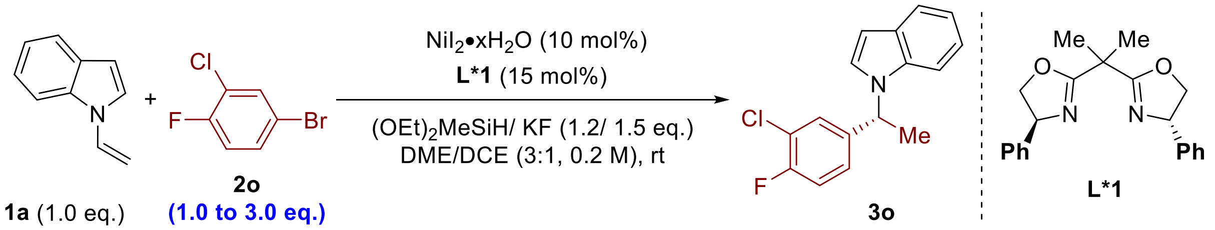

Supplementary Table 12. Rates determined by varying [Aryl Bromide]

[Aryl Bromide] (M)	Average Rate (M/min)
0.20	7.47×10^{-3}
0.30	8.71×10^{-3}
0.50	1.23×10^{-2}
0.60	1.49×10^{-2}

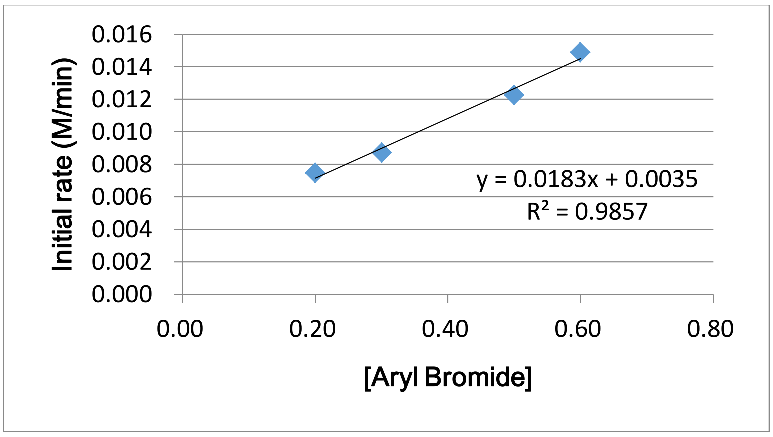

5) Rate order determination of $(OEt)_2MeSiH$

Supplementary Table 13. Rates determined by varying $[(OEt)_2MeSiH]$

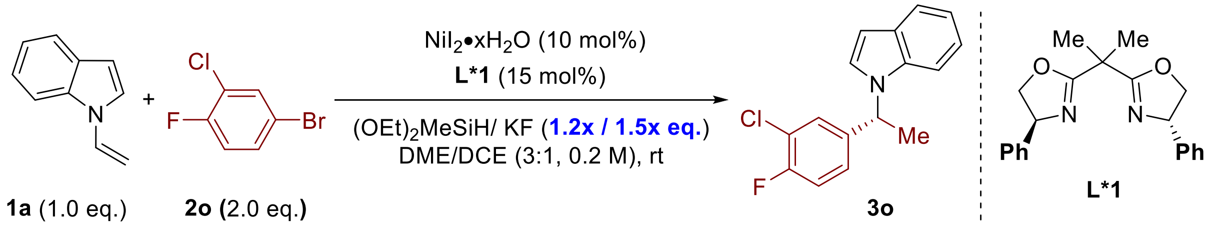

$[(OEt)_2MeSiH]$ (M)	Average Rate (M/min)
0.24	6.74×10^{-3}
0.30	1.15×10^{-2}
0.40	1.45×10^{-2}
0.50	1.39×10^{-2}
0.60	7.29×10^{-3}

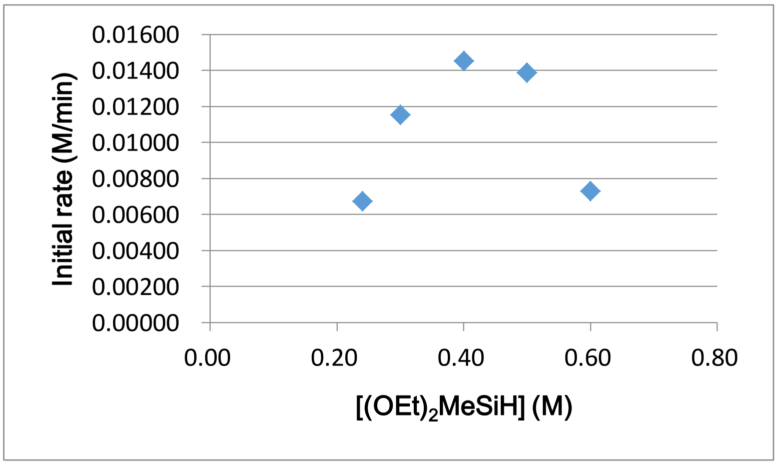

Supplementary Table 14. Rates dependence on [$(OEt)_2MeSiH$] with constant [KF]

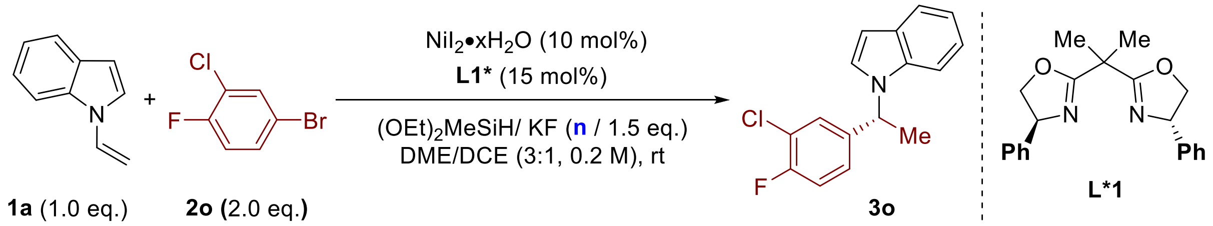

[$(OEt)_2MeSiH$] (M)	Average Rate (M/min)
0.10	6.28 x 10^{-3}
0.20	3.94 x 10^{-3}
0.30	8.46 x 10^{-3}
0.40	5.56 x 10^{-3}
0.50	8.31 x 10^{-3}

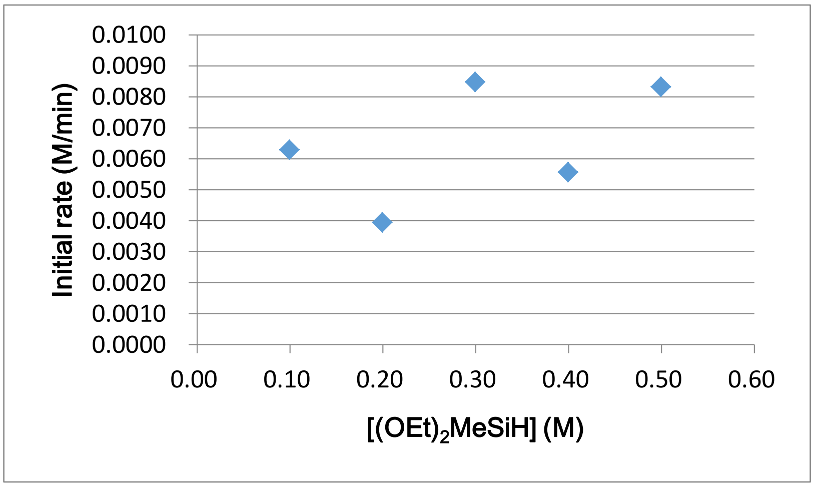

1.9.2 Hammett plots of aryl bromides

The general procedure for rate determination was followed using 4-methoxybromobenzene (50.1 uL, 0.40 mmol, 2.0 equiv), 4-fluorobromobenzene (44.0 uL, 0.40 mmol, 2.0 equiv), 4-chlorobromobenzene (76.6 mg, 0.40 mmol, 2.0 equiv), and 4-acetylbromobenzene (79.6 mg, 0.40 mmol, 2.0 equiv). Each experiment was repeated in duplicate. The quantitative rates of hydroaralation of these substrates are provided below with a corresponding Hammett plot.

Supplementary Table 15. Hammett plots of aryl bromides

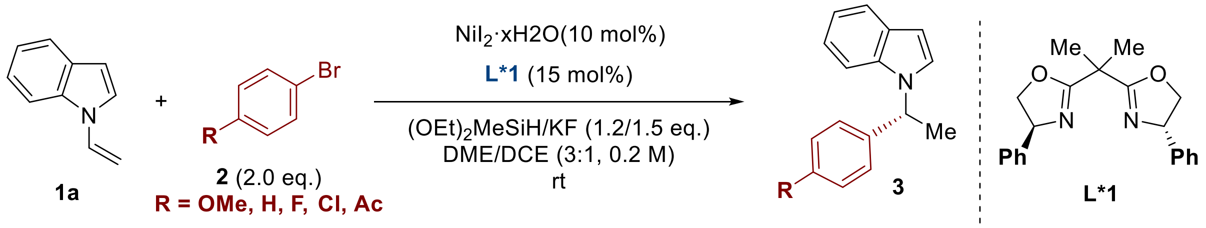

R	σ	Average Rate (M/min)
OMe	-0.27	6.70×10^{-3}
H	0	9.32×10^{-3}
F	0.06	9.64×10^{-3}
Cl	-0.23	1.32×10^{-2}
Ac	0.50	2.19×10^{-2}

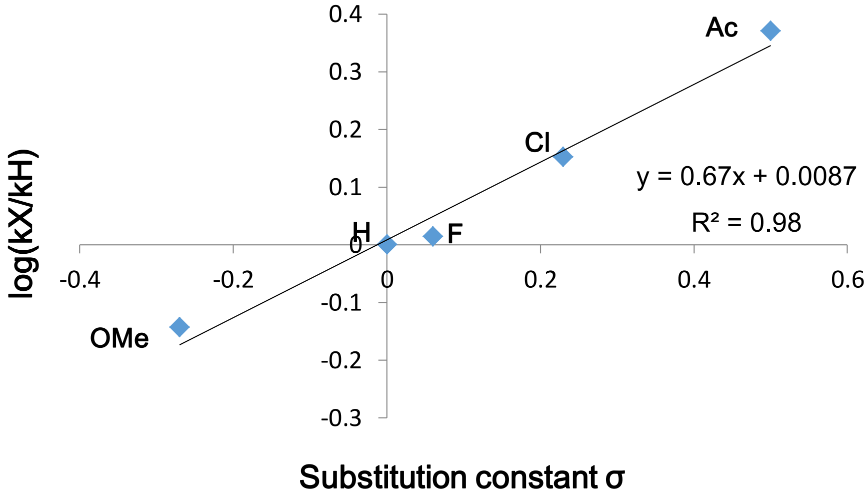

1.9.3 Nonlinear effect study

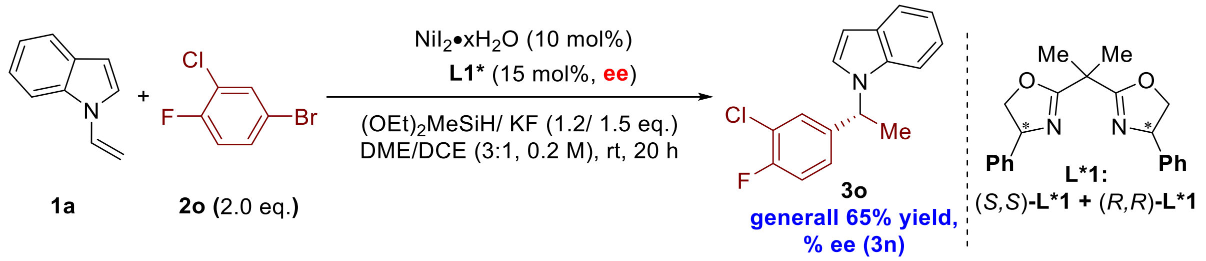

Supplementary Table 16. Nonlinear effect studies – setup of experiment and results

ee (%), L*1	(*S,S*)-L*1 (mg)	(*R,R*)-L*1 (mg)	ee (%), 3n
20	6.0	4.0	18.9
40	7.0	3.0	37.3
60	8.0	2.0	58.4
80	9.0	1.0	75.2
99	10.0	0	89.8

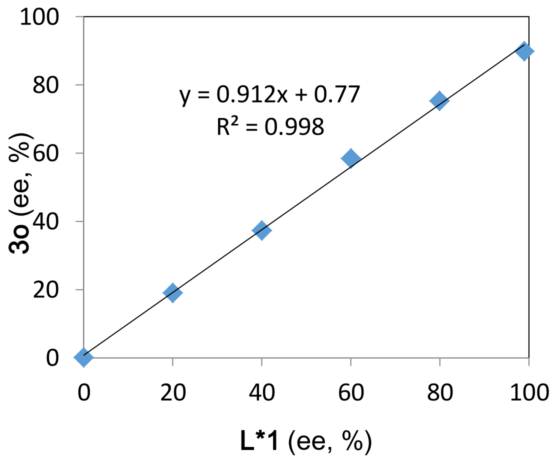

Figure S1 Nonlinear effect.

In a nitrogen-filled glove box, five 8-mL scintillation vials were charged with the specified amount of (*S,S*)-**L*1** and (*R,R*)- **L*1** to provide the enantiomeric composition of **L*1** as specified in Table S8-5. To each of these vials, NiI_2•xH_2O (8.4 mg, 10 mol%) and DME (0.75 mL) was added and these vials were stirred to homogeneity (30 min). Once homogeneity was reached, KF (17.5 mg, 0.30 mmol, 1.5 equiv), *N*-vinylindole **1a** (0.20 mmol, 1.0 equiv), aryl bromide **2o** (49.0 uL, 0.4 mmol, 2.0 equiv), DCE (0.25 mL), and $(OEt)_2MeSiH$ (38.6 uL, 0.24 mmol, 1.2 equiv.) were sequentially added. Caps were tightened on the reaction vial, the reaction vials were brought outside of the glove box, and stirred for 20 h. Afterwards, EtOAc (5.0 mL) and saturated NH_4Cl (aq., 1.0 mL) were added to the reaction mixture. The organic phase was separated and the aqueous phase was extracted with EtOAc (2x5.0 mL). The combined organic phases were dried over Na_2SO_4, and the volatiles were removed to afford the crude product. Then, the mixture was purified by PTLC and the enantiomeric excess (ee) was determined by HPLC analysis.

1.9.4 Linear free energy relationship studies

Enantiomeric ratios were compiled for the amine products shown above. The average er of two experiments for each substrate is shown below with the log(er), standard deviation and a Hammett plot.

Supplementary Table 17. Linear free energy relationship studies

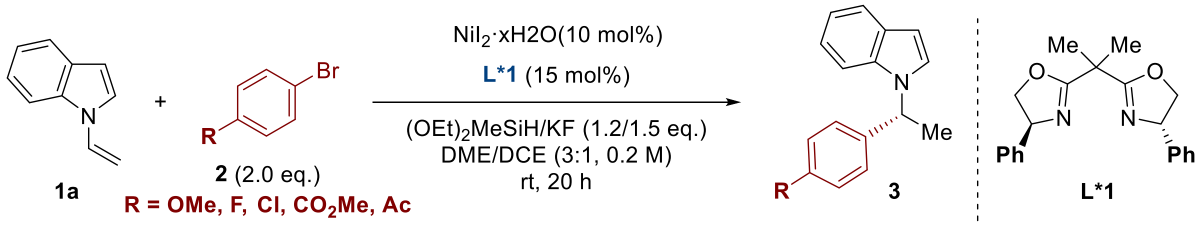

R	σ	Average er	log(er)
OMe	-0.27	78:22	0.55
F	0.06	90:10	0.95
Cl	0.23	93:7	1.12
CO_2Me	0.45	95.5:4.5	1.33
Ac	0.50	96:4	1.38

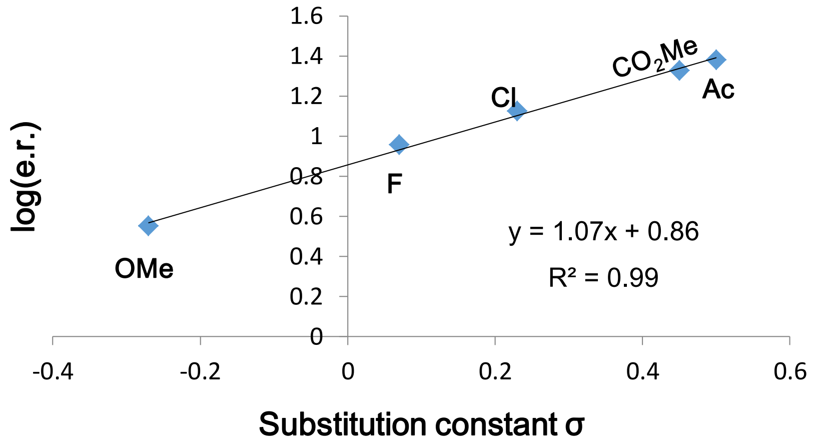

1.10 Determination of the Absolute Configuration

In Table 2 and Table 4, the absolution configuration of **3d** and **3g** were determined to be *R* by comparing their optical rotation with literature data, and the configurations of the other compounds described in this work are assigned in analogy to **3d** and **3g**.

In Table 3 and Figure 2, the absolution configuration of **11** was determined to be *S* by comparing its optical rotation with literature data, and the configurations of the other compounds described in this work are assigned in analogy to **11**.

**Our products**	**Known compounds**	**References**
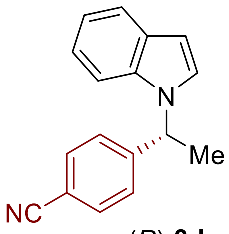 (*R*)-**3d**	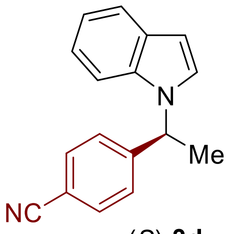 (*S*)-**3d**	*Org. Lett.* **2019**, *21*, 8957[11]
90% ee	42% ee	
$[\alpha]_D^{20}$ = +135.2 (c = 0.92, $CHCl_3$)	$[\alpha]_D^{20}$ = −34.6 (c = 2.9, $CHCl_3$)	
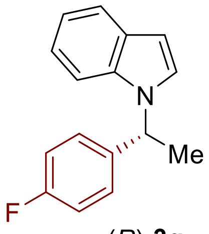 (*R*)-**3g**	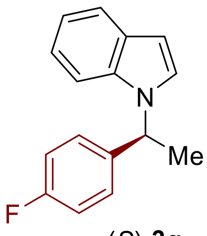 (*S*)-**3g**	*J. Am. Chem. Soc.* **2019**, *141*, 3901[12]
80% ee	93% ee	
$[\alpha]_D^{20}$ = +58.3 (c = 0.55, $CHCl_3$)	$[\alpha]_D^{22}$ = -80.6 (c = 1.0, $CHCl_3$)	

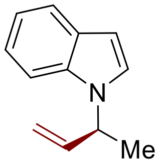 (*S*)-**11**	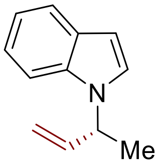 (*R*)-**11**	*Angew. Chem. Int. Ed.* **2012**, *51*, 5183[13]
90% ee	82% ee	
**[α]$_D$^{20}** = -18.7 (c = 0.2, $CHCl_3$)	**[α]$_D$^{20}** = +13.2 (c = 1.0, $CHCl_3$)	

1.11 NMR and HPLC Spectra

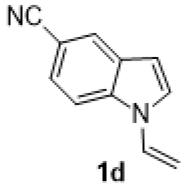

1d

^{1}H NMR, 400 MHz, $CDCl_3$

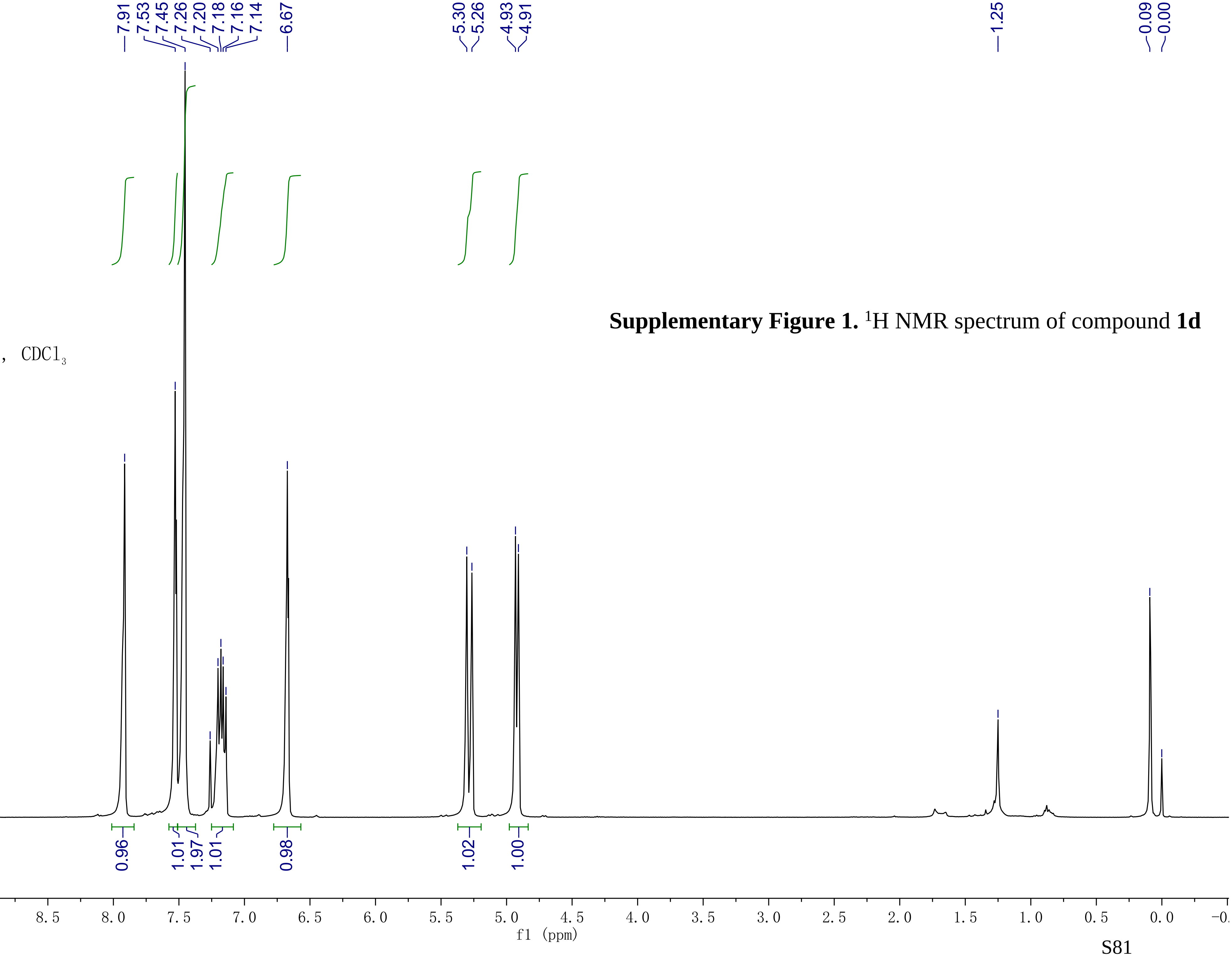

Supplementary Figure 1. ^{1}H NMR spectrum of compound **1d**

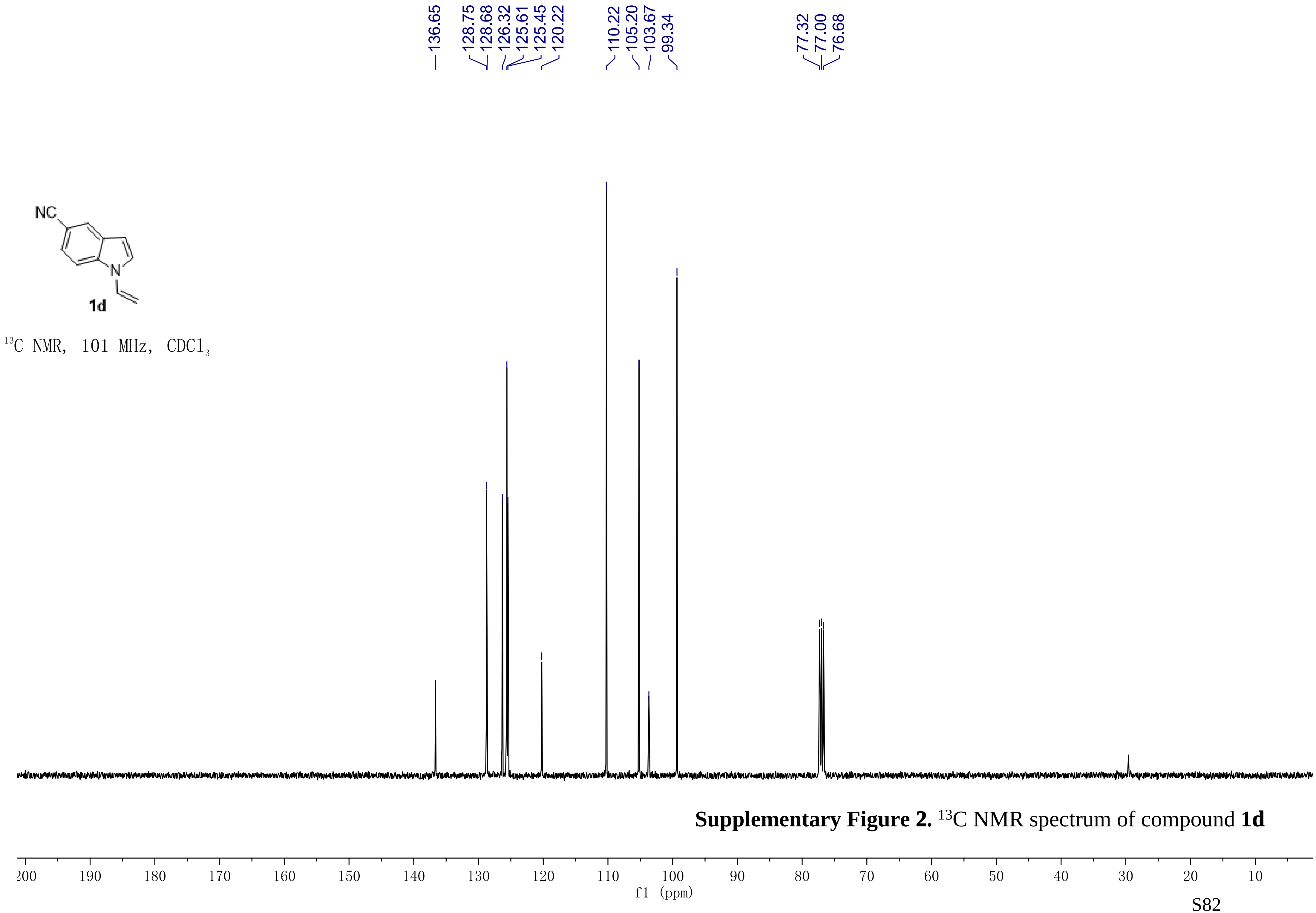

Supplementary Figure 2. ^{13}C NMR spectrum of compound **1d**

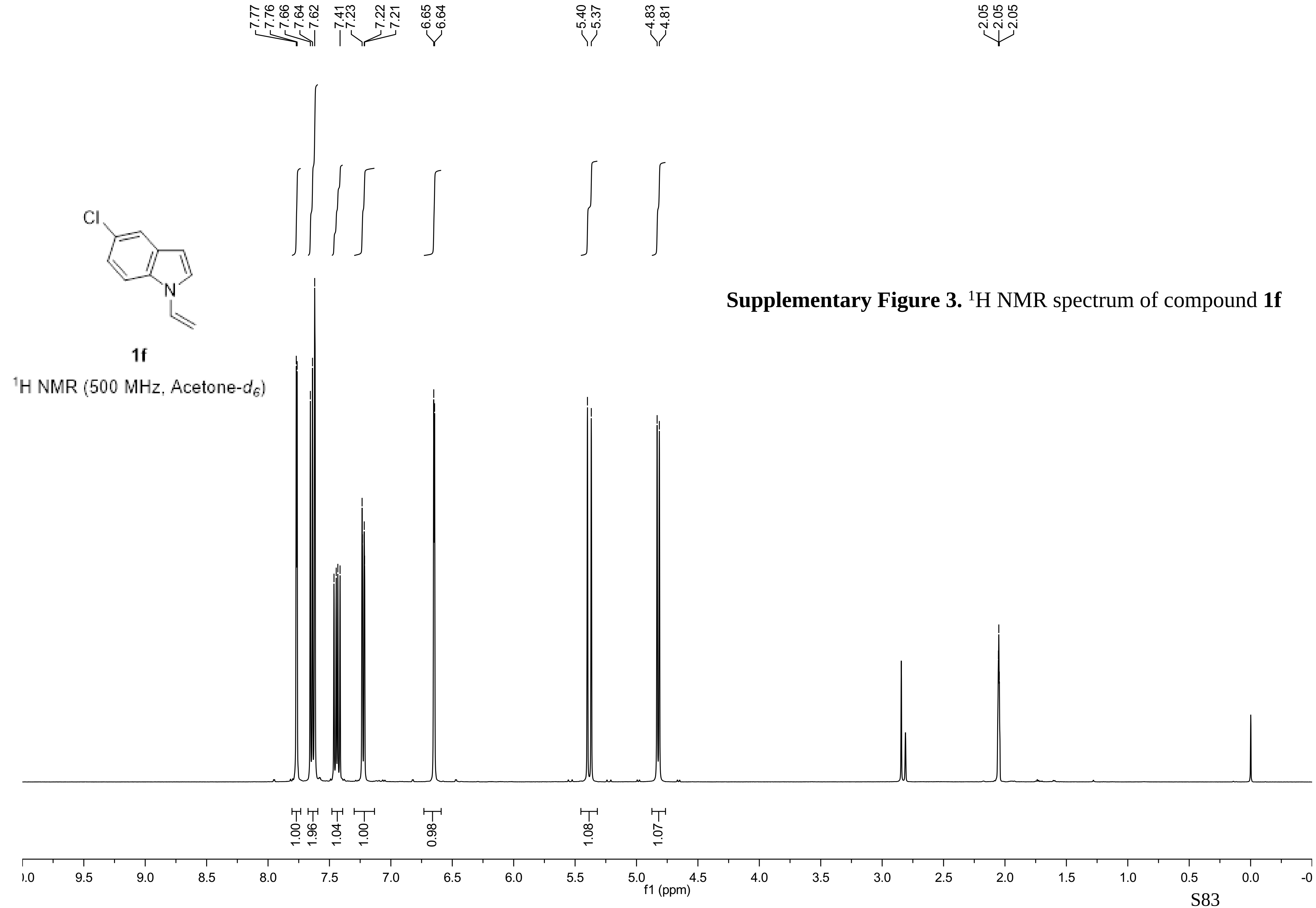

1f

^{1}H NMR (500 MHz, Acetone-d_6)

Supplementary Figure 3. ^{1}H NMR spectrum of compound **1f**

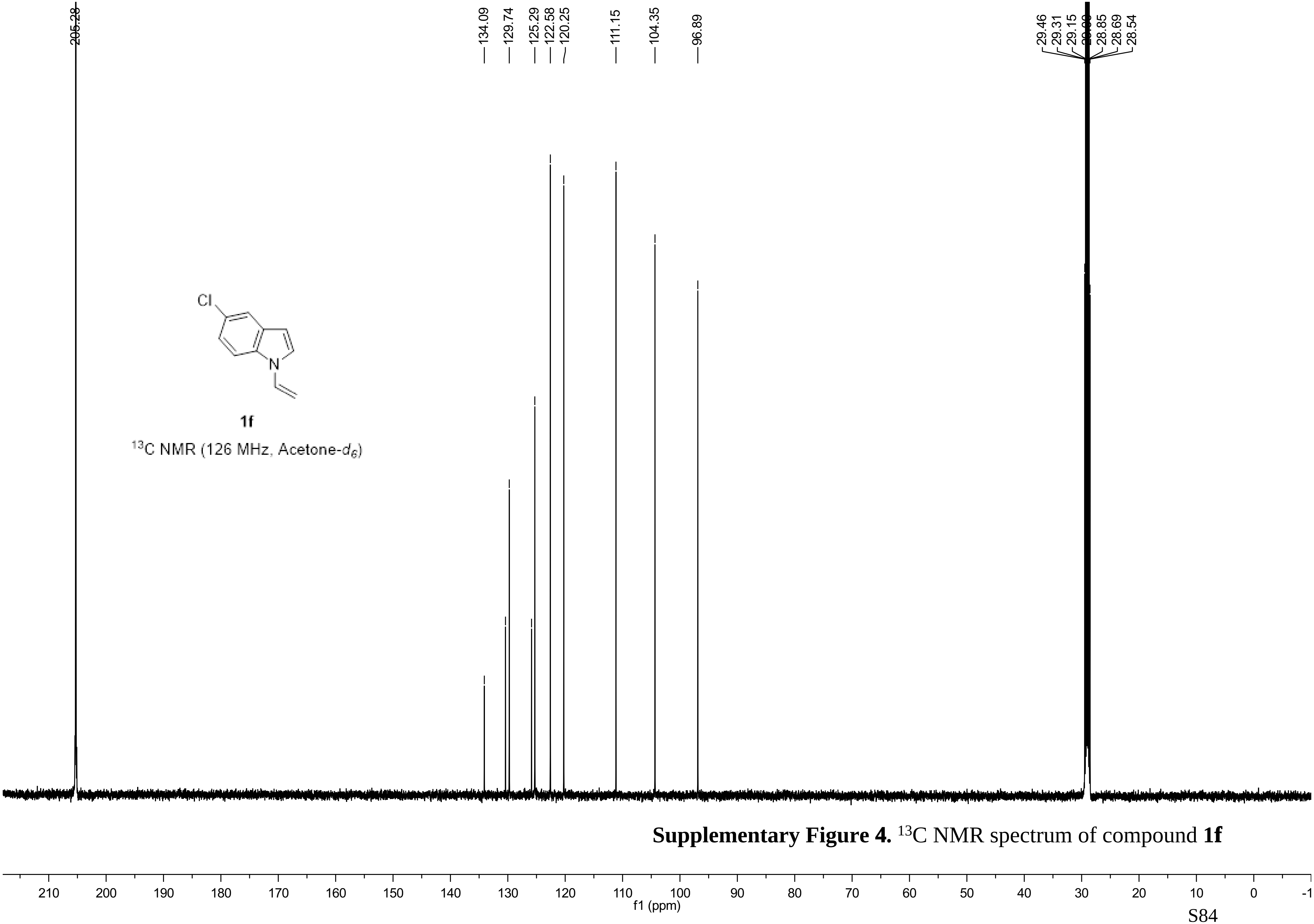

Supplementary Figure 4. ^{13}C NMR spectrum of compound **1f**

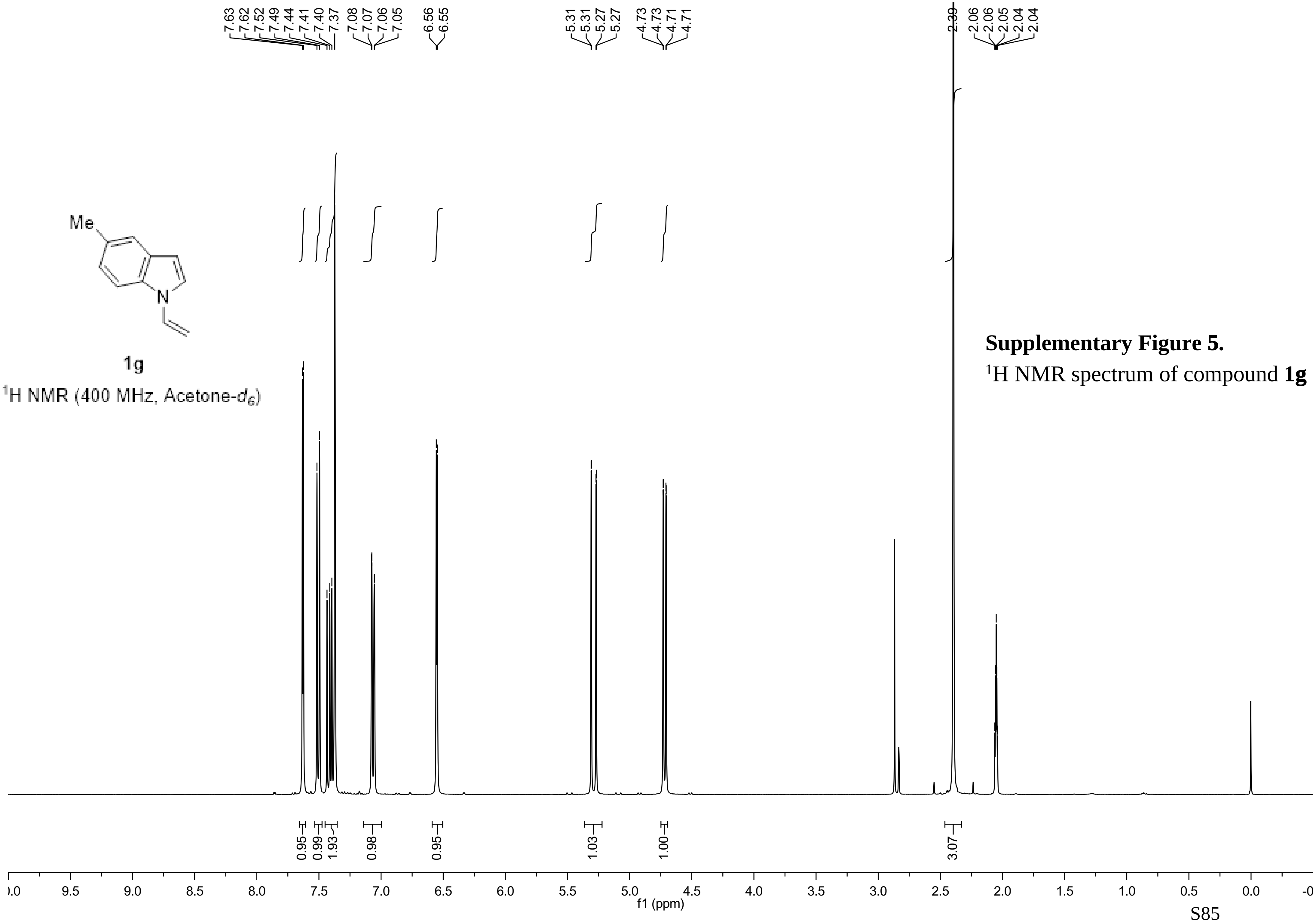

Supplementary Figure 5.
^{1}H NMR spectrum of compound **1g**

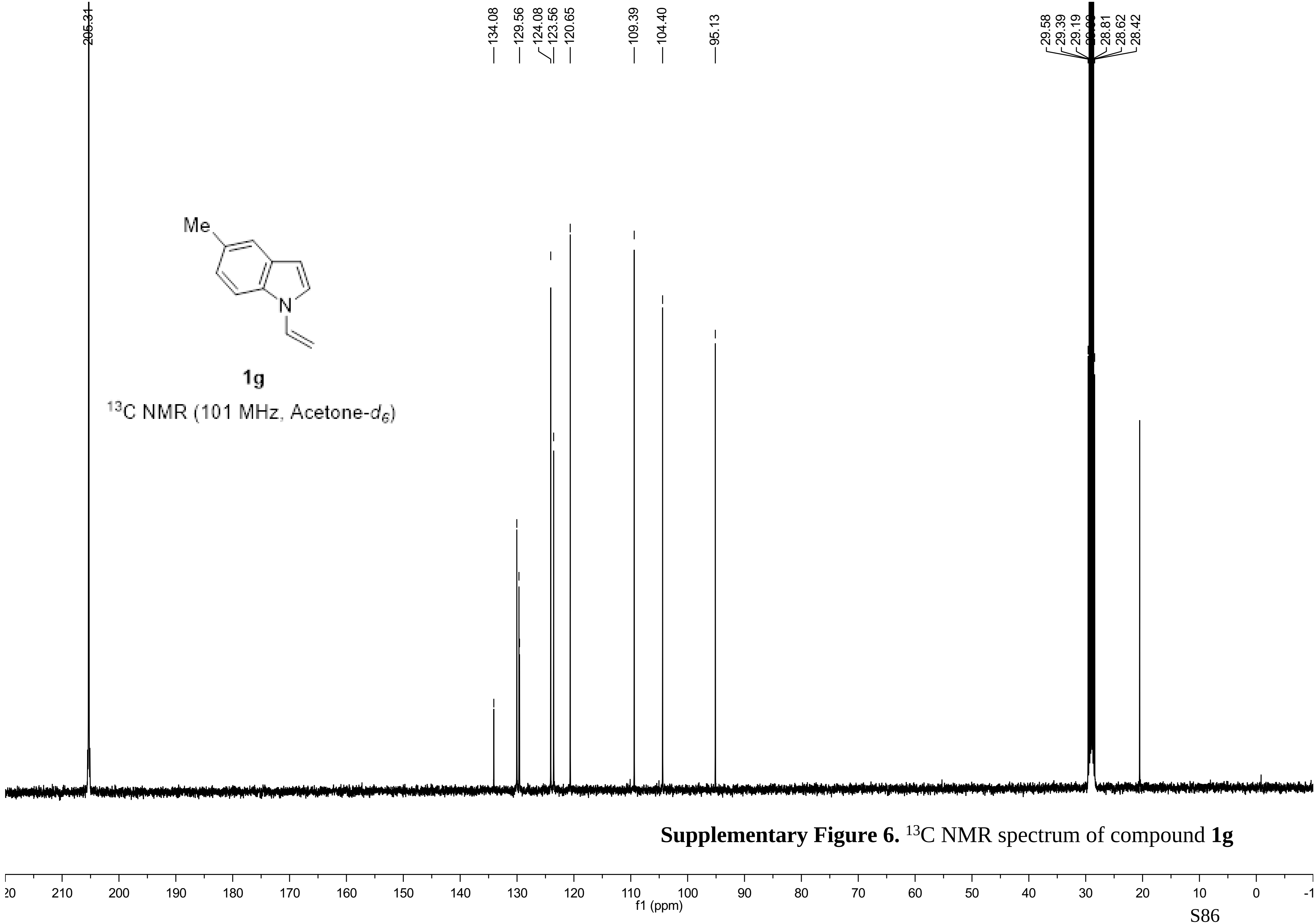

Supplementary Figure 6. ^{13}C NMR spectrum of compound **1g**

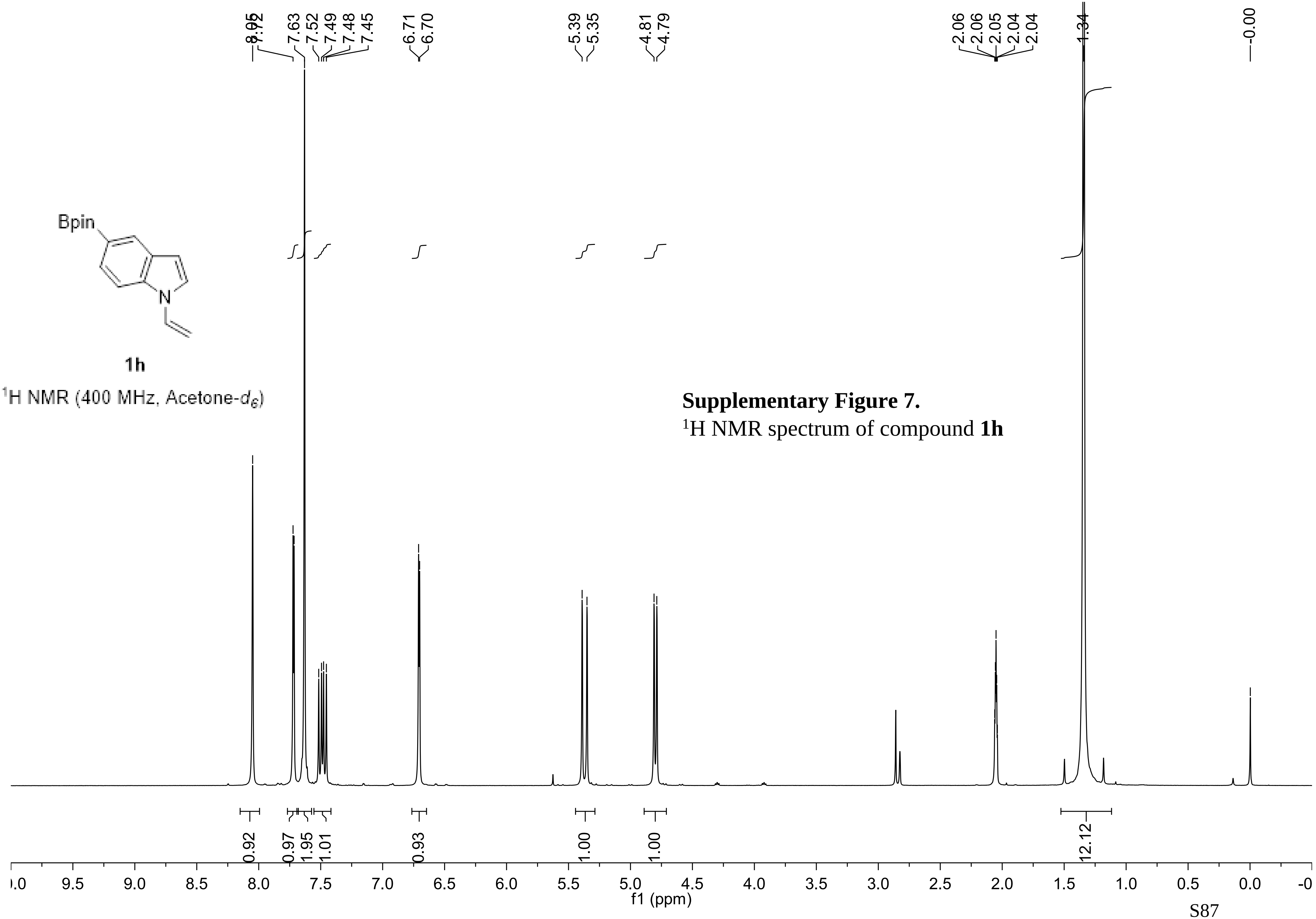

Supplementary Figure 7.
^{1}H NMR spectrum of compound **1h**

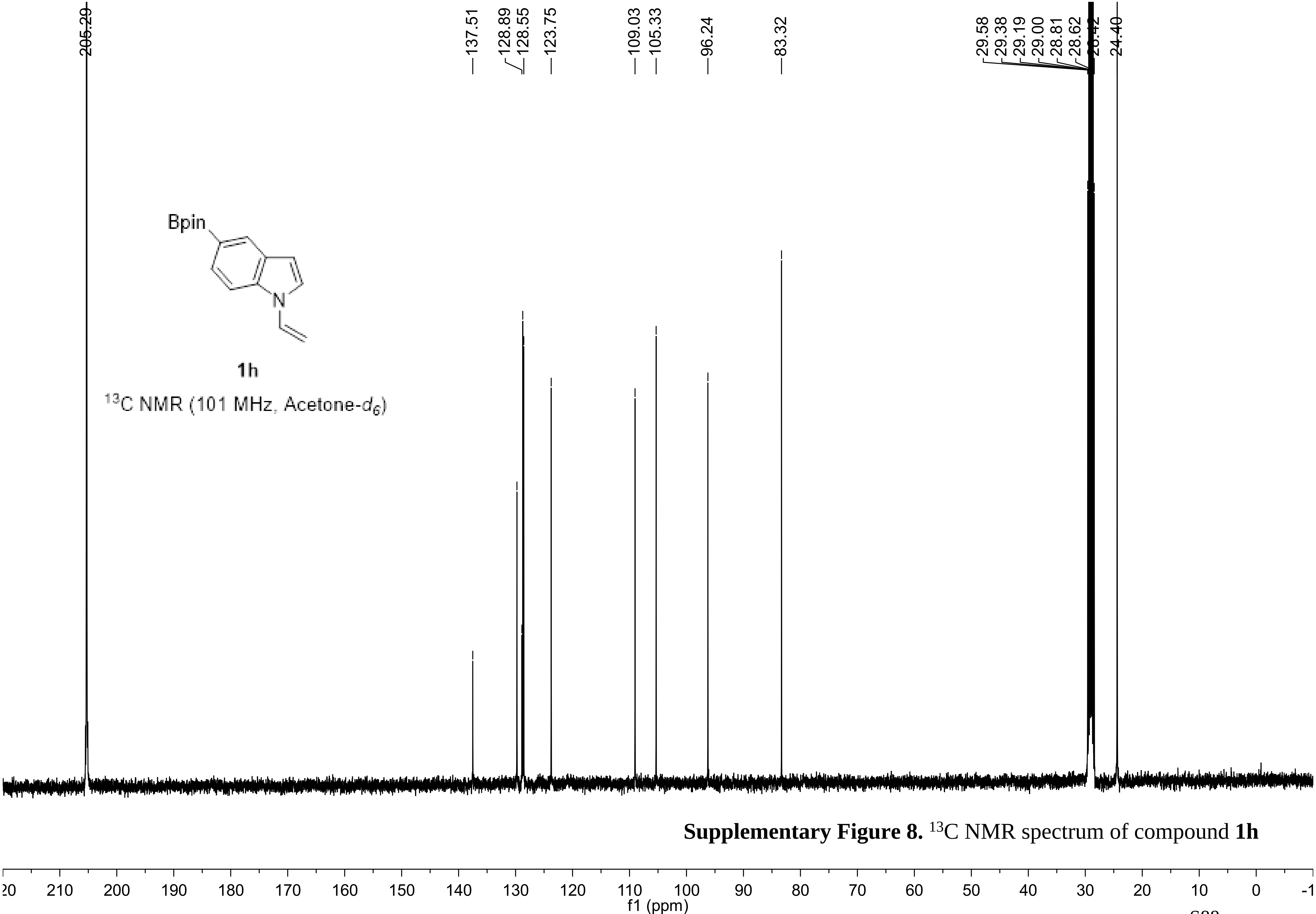

Supplementary Figure 8. ^{13}C NMR spectrum of compound **1h**

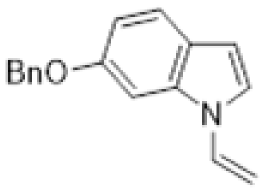

1j

^{1}H NMR (400 MHz, Acetone-d_6)

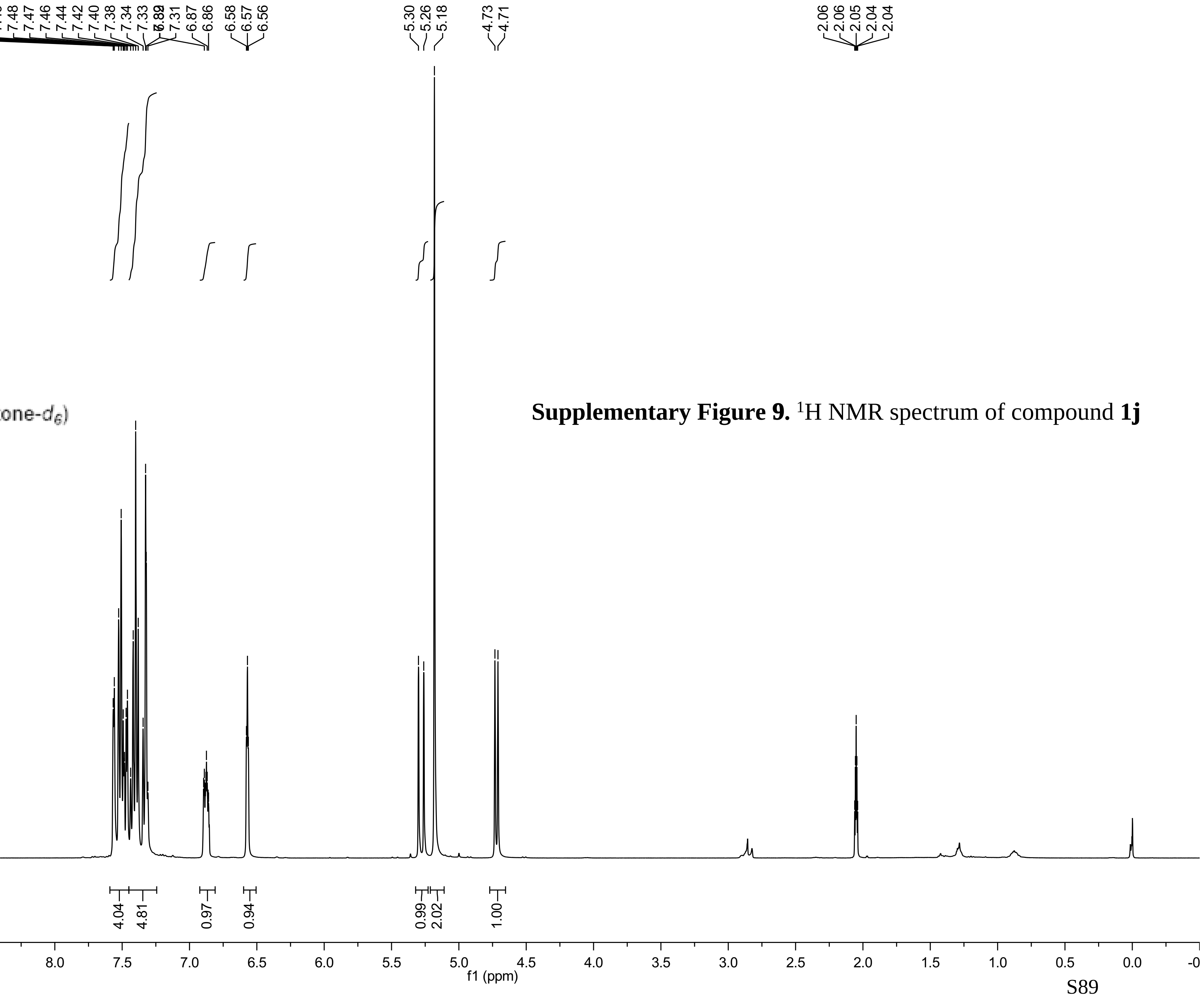

Supplementary Figure 9. ^{1}H NMR spectrum of compound **1j**

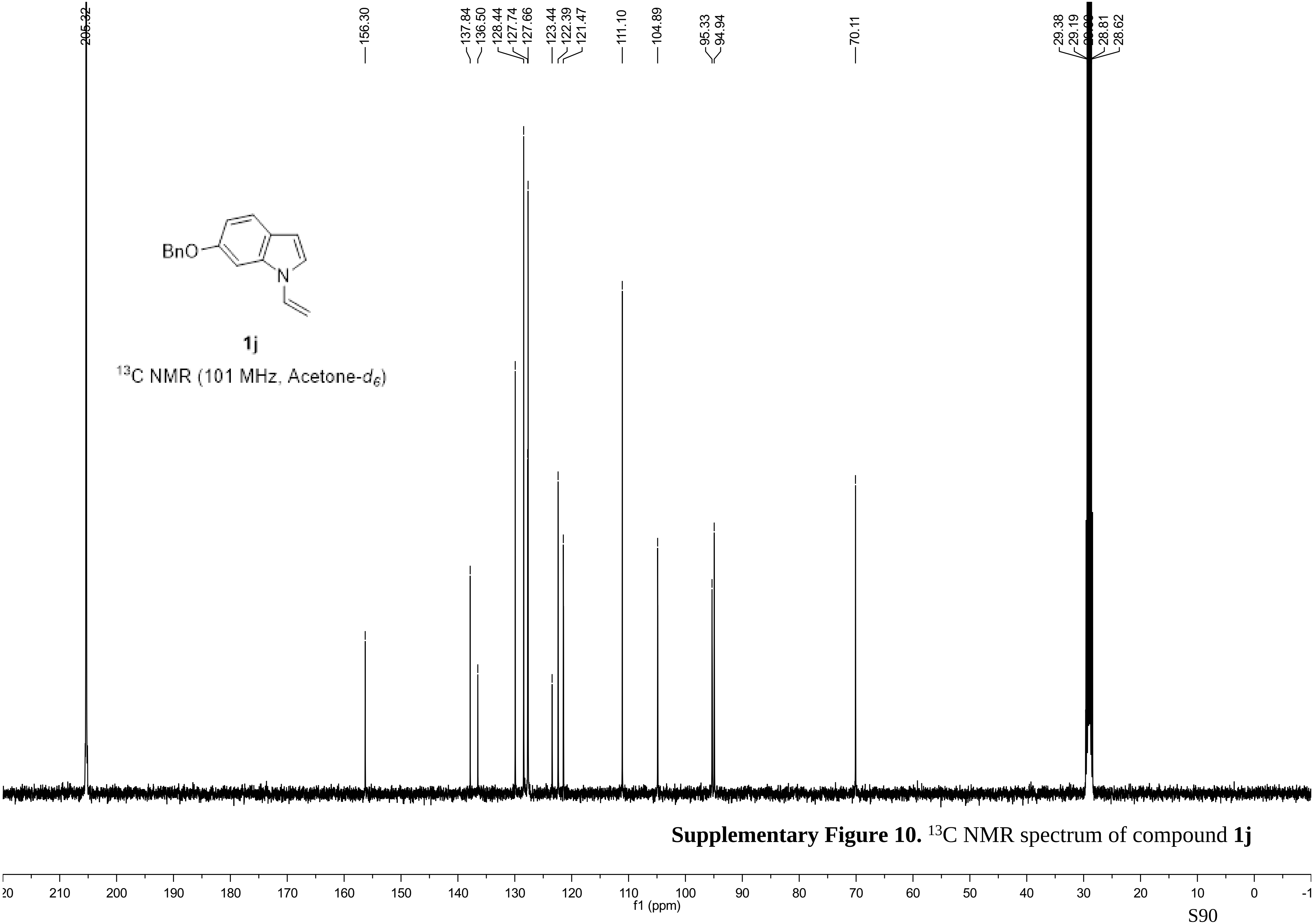

Supplementary Figure 10. ^{13}C NMR spectrum of compound **1j**

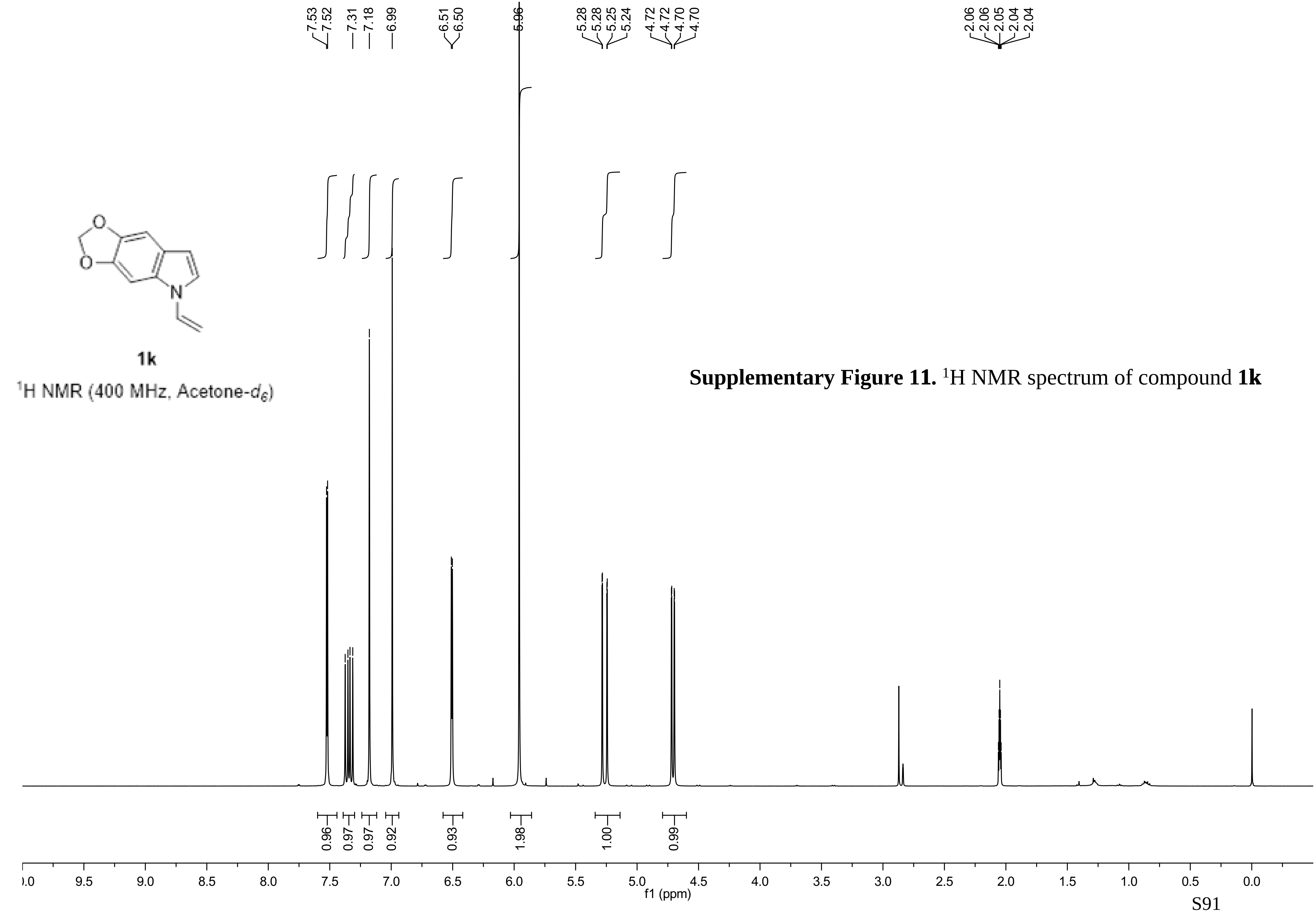

Supplementary Figure 11. ^{1}H NMR spectrum of compound **1k**

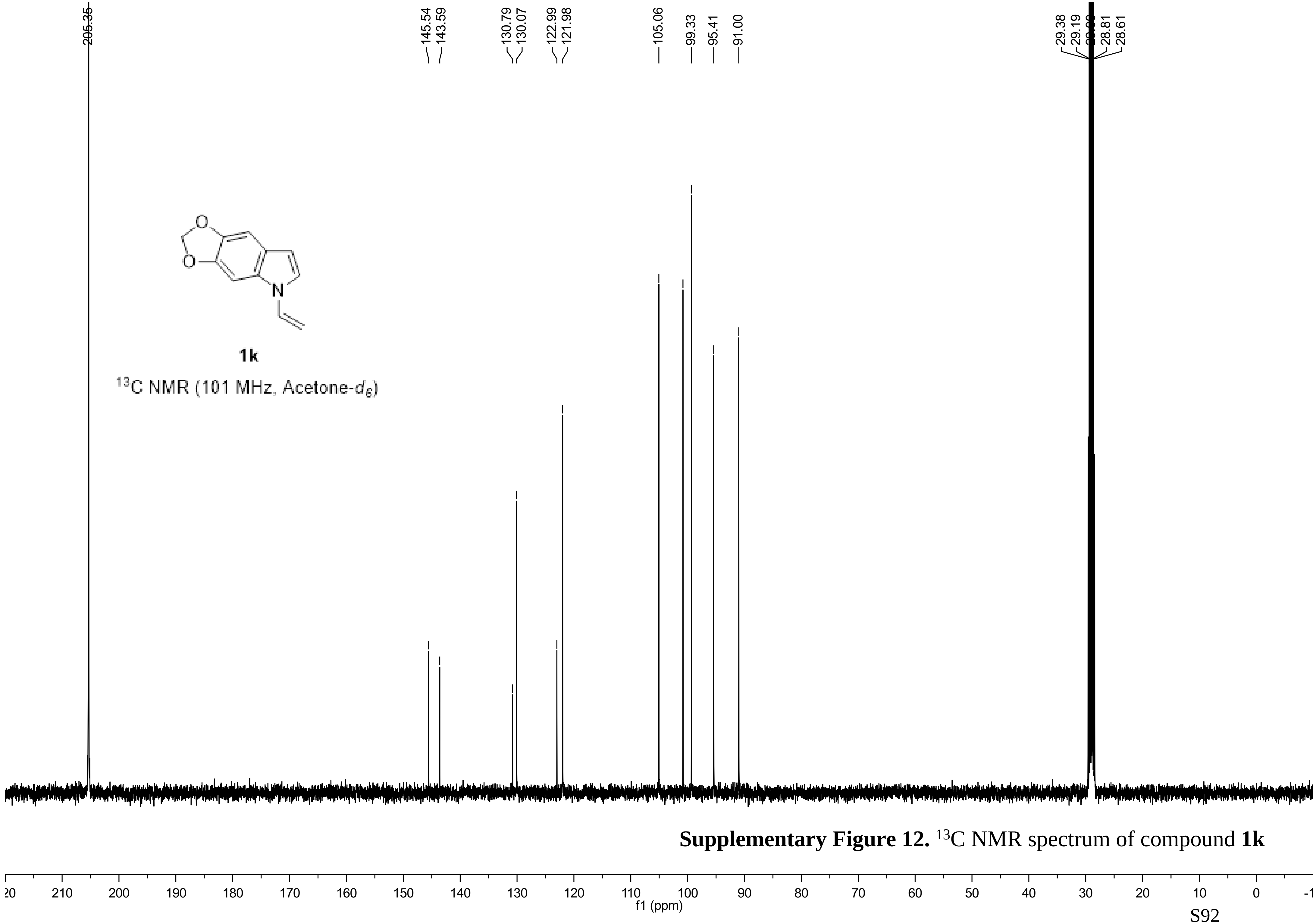

Supplementary Figure 12. ^{13}C NMR spectrum of compound **1k**

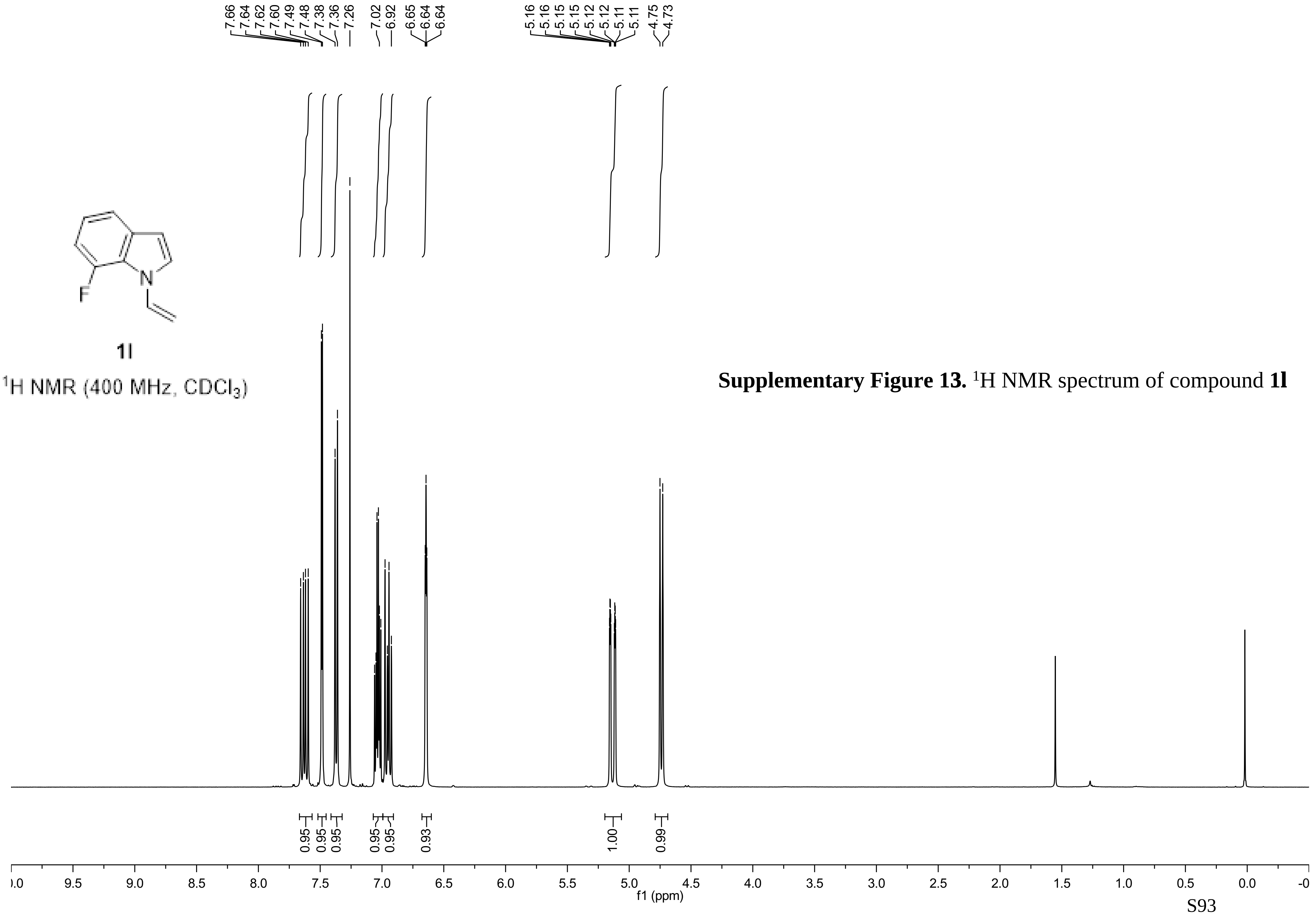

Supplementary Figure 13. ^{1}H NMR spectrum of compound **1l**

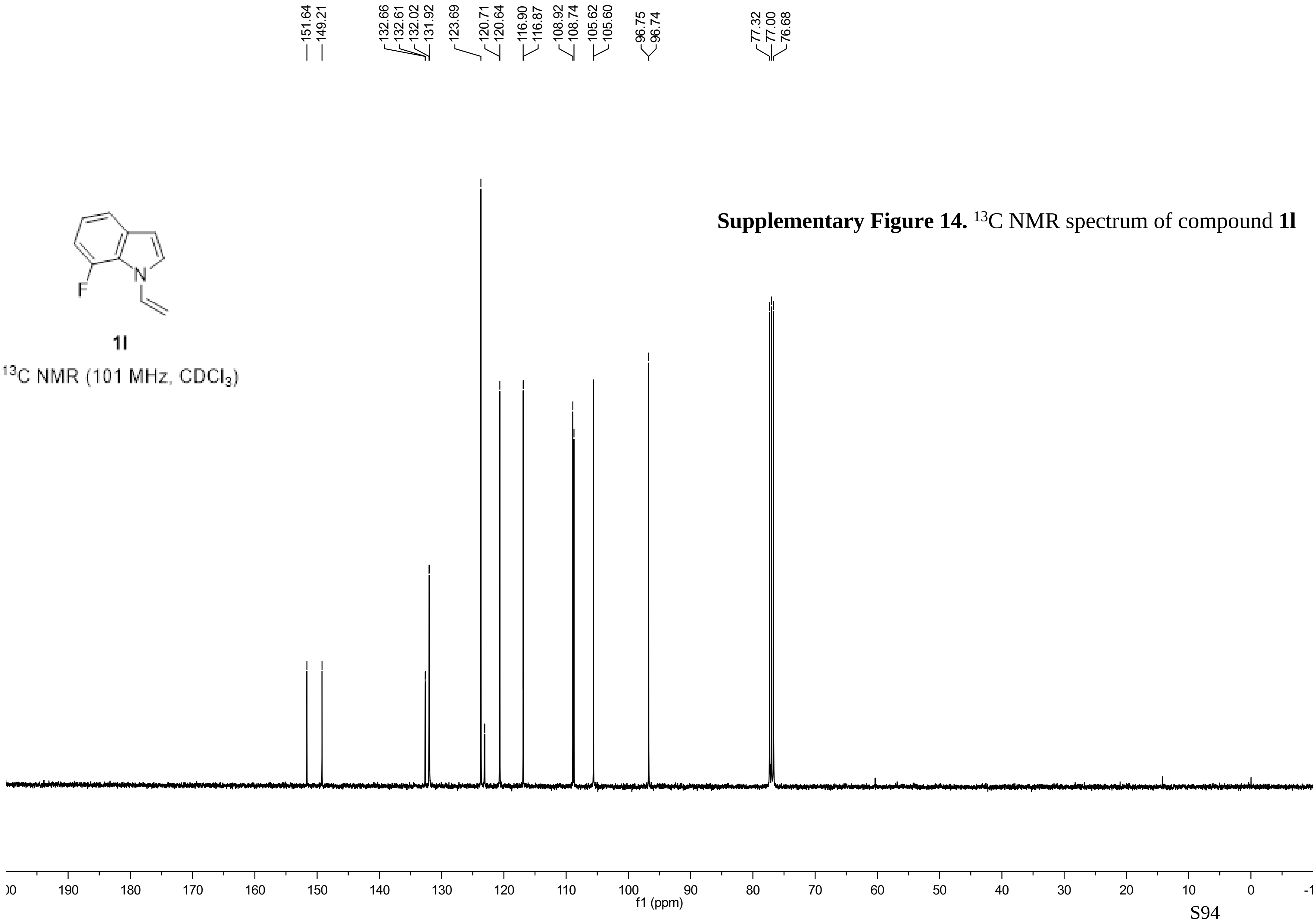

1l

^{13}C NMR (101 MHz, $CDCl_3$)

Supplementary Figure 14. ^{13}C NMR spectrum of compound **1l**

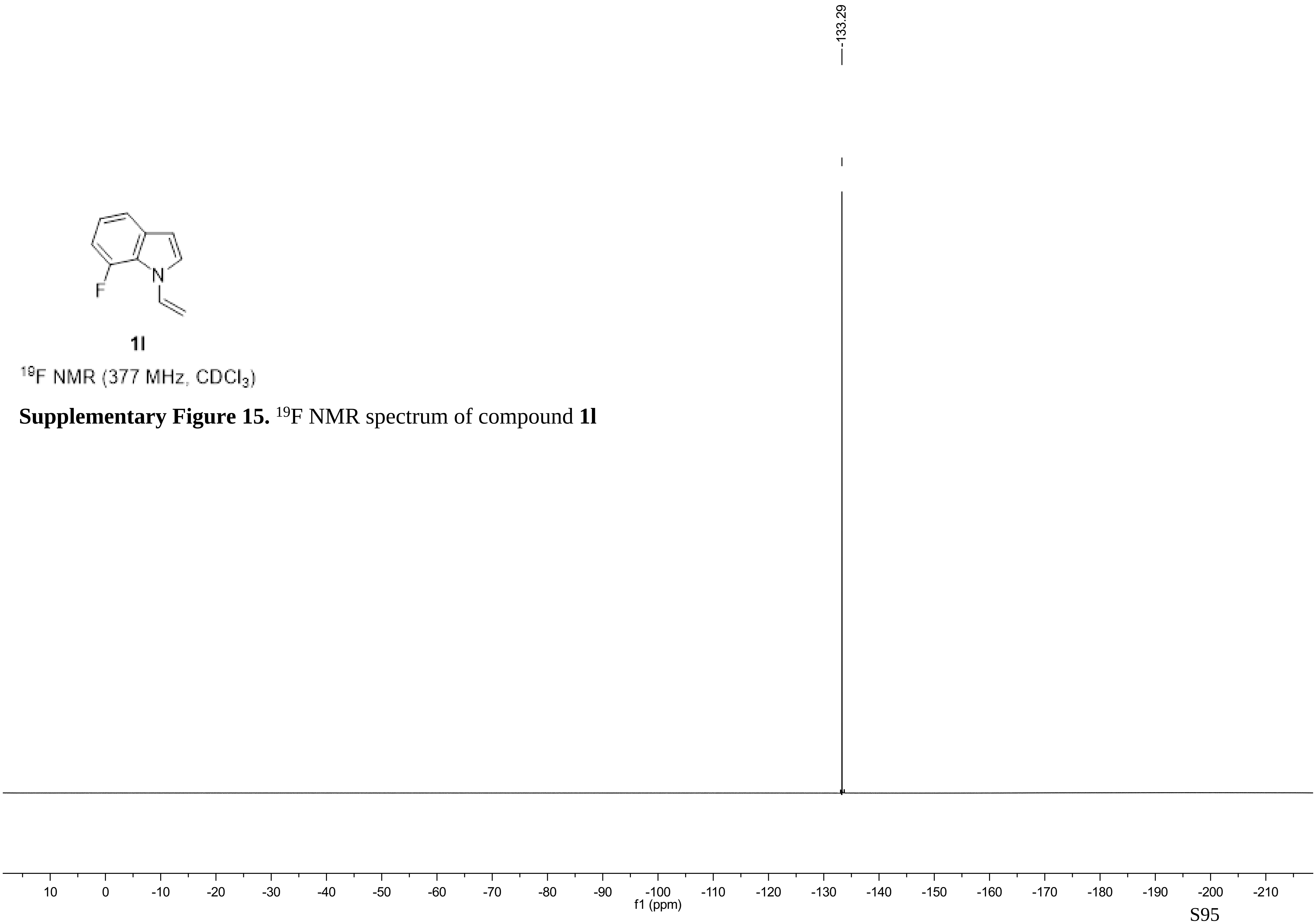

1l

^{19}F NMR (377 MHz, $CDCl_3$)

Supplementary Figure 15. ^{19}F NMR spectrum of compound **1l**

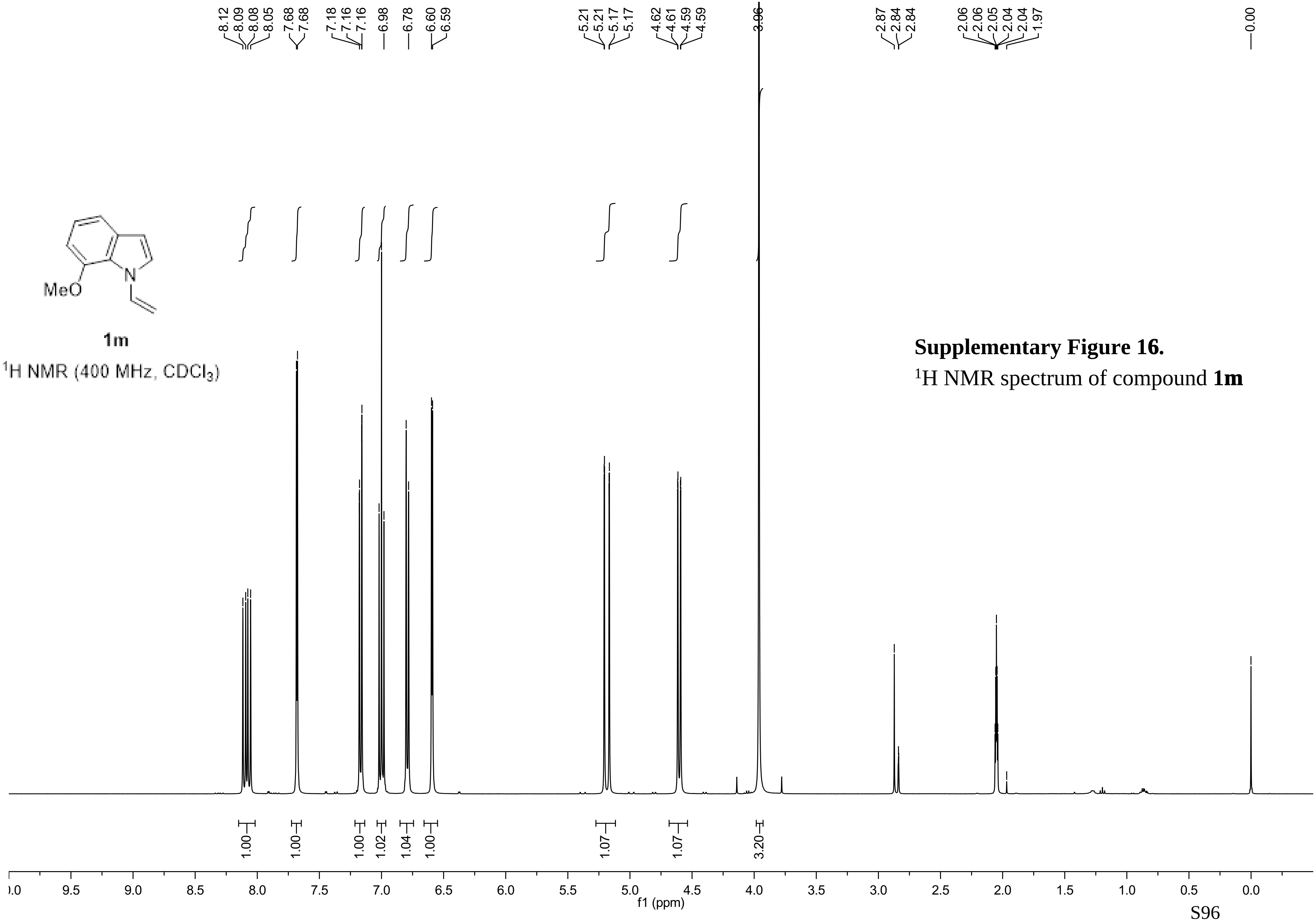

Supplementary Figure 16.
^{1}H NMR spectrum of compound **1m**

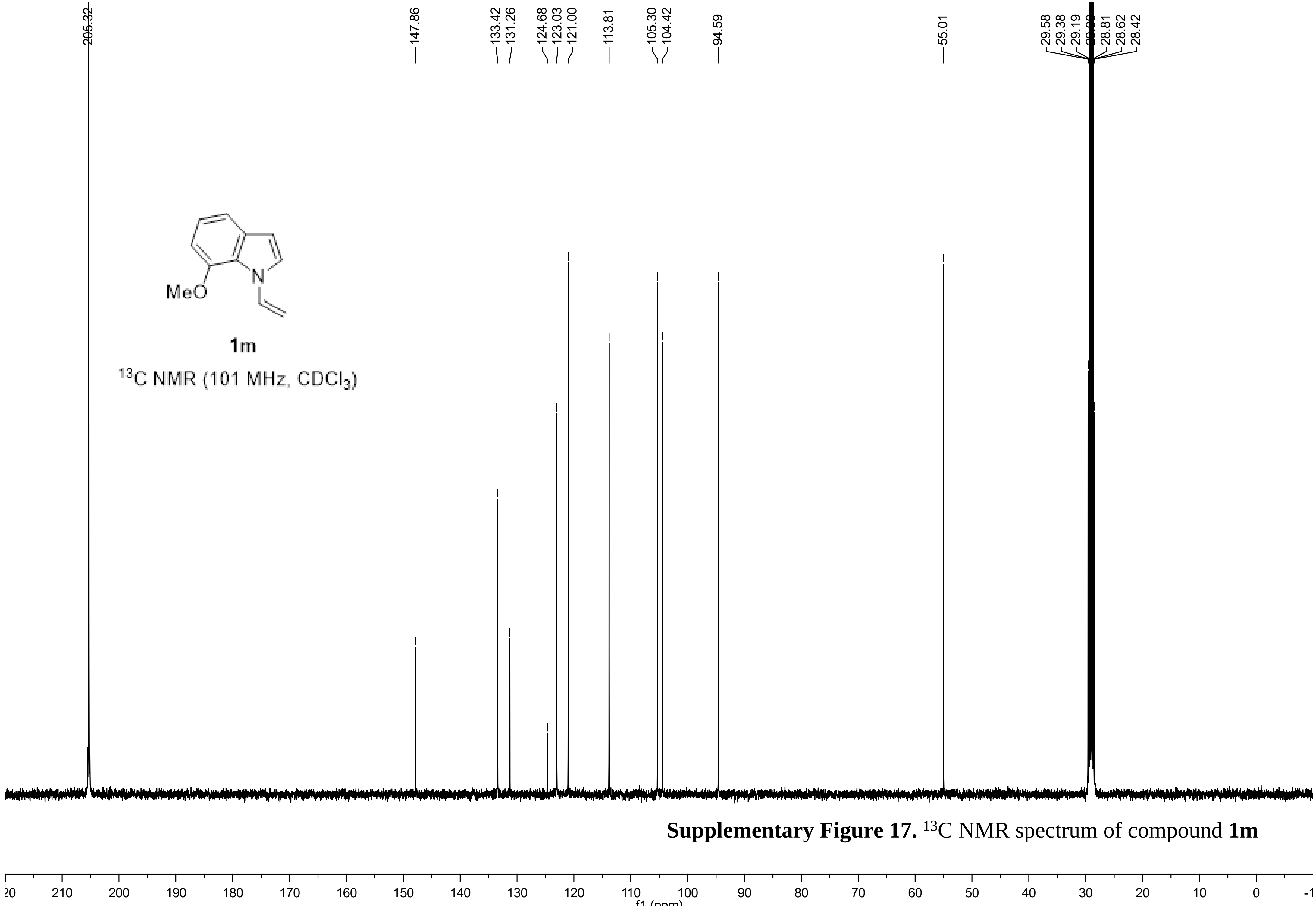

Supplementary Figure 17. ^{13}C NMR spectrum of compound **1m**

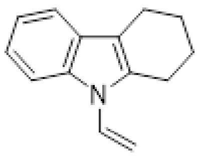

1n

^{1}H NMR (400 MHz, Acetone-d_6)

Supplementary Figure 18.

^{1}H NMR spectrum of compound **1n**

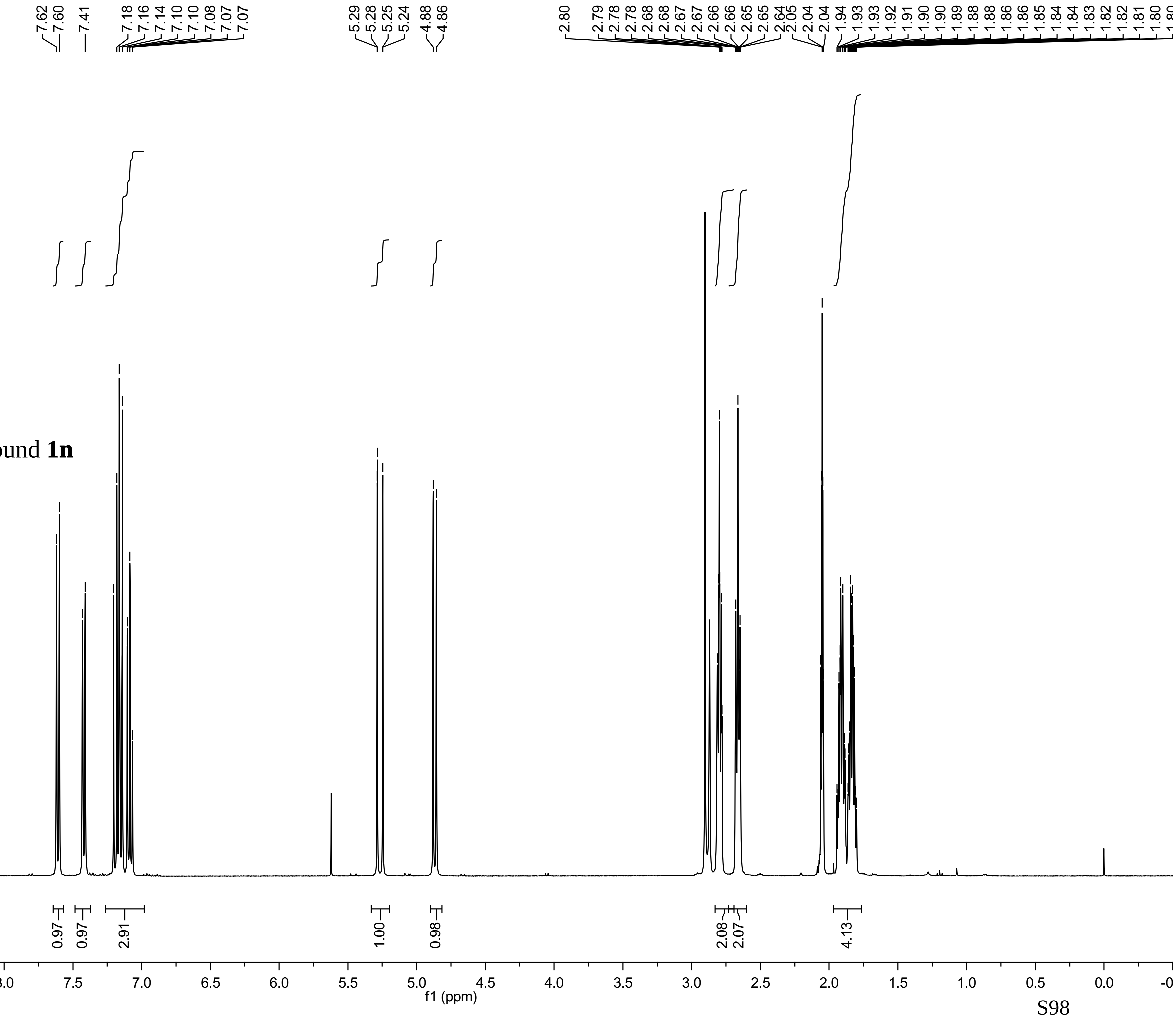

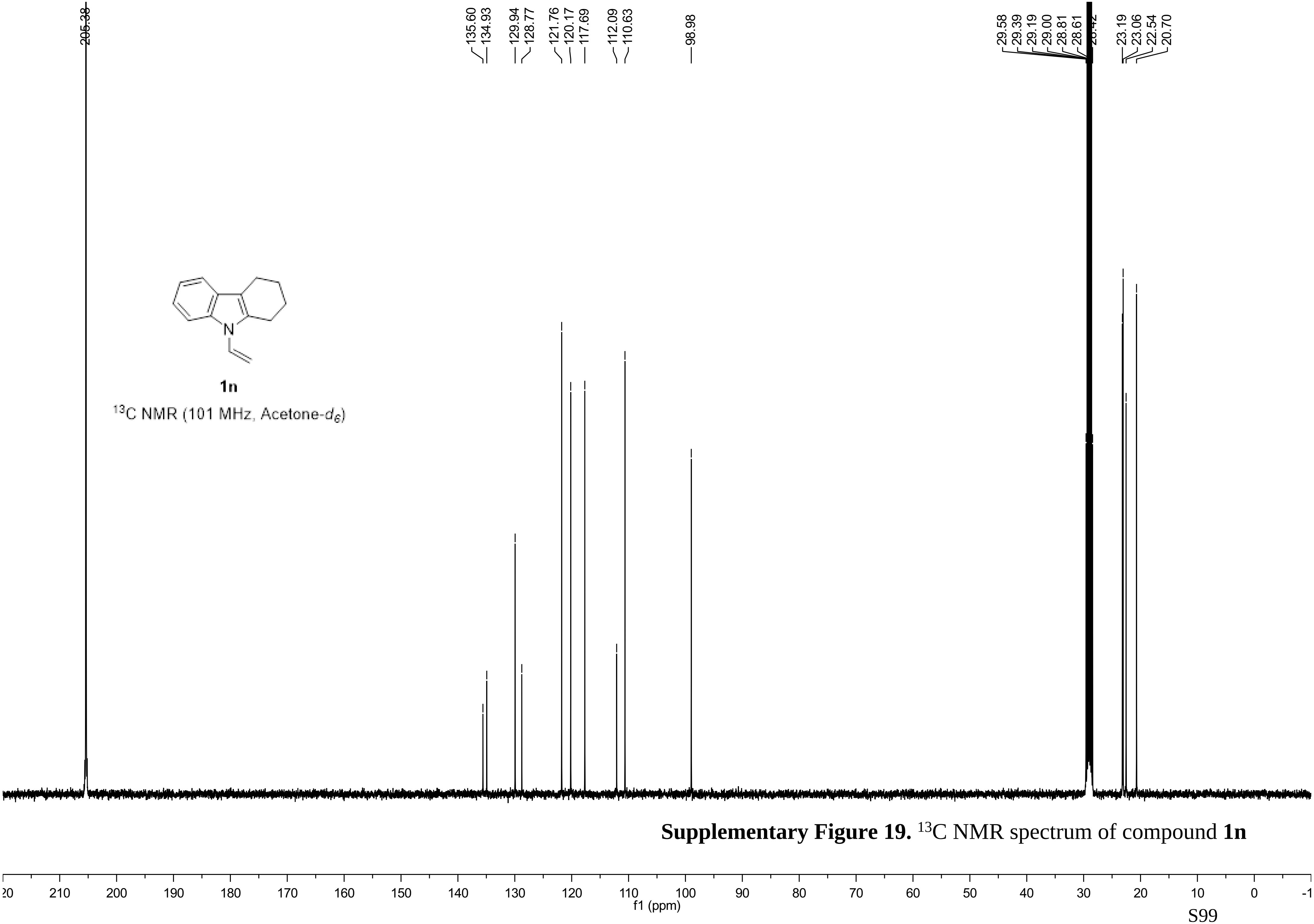

Supplementary Figure 19. ^{13}C NMR spectrum of compound **1n**

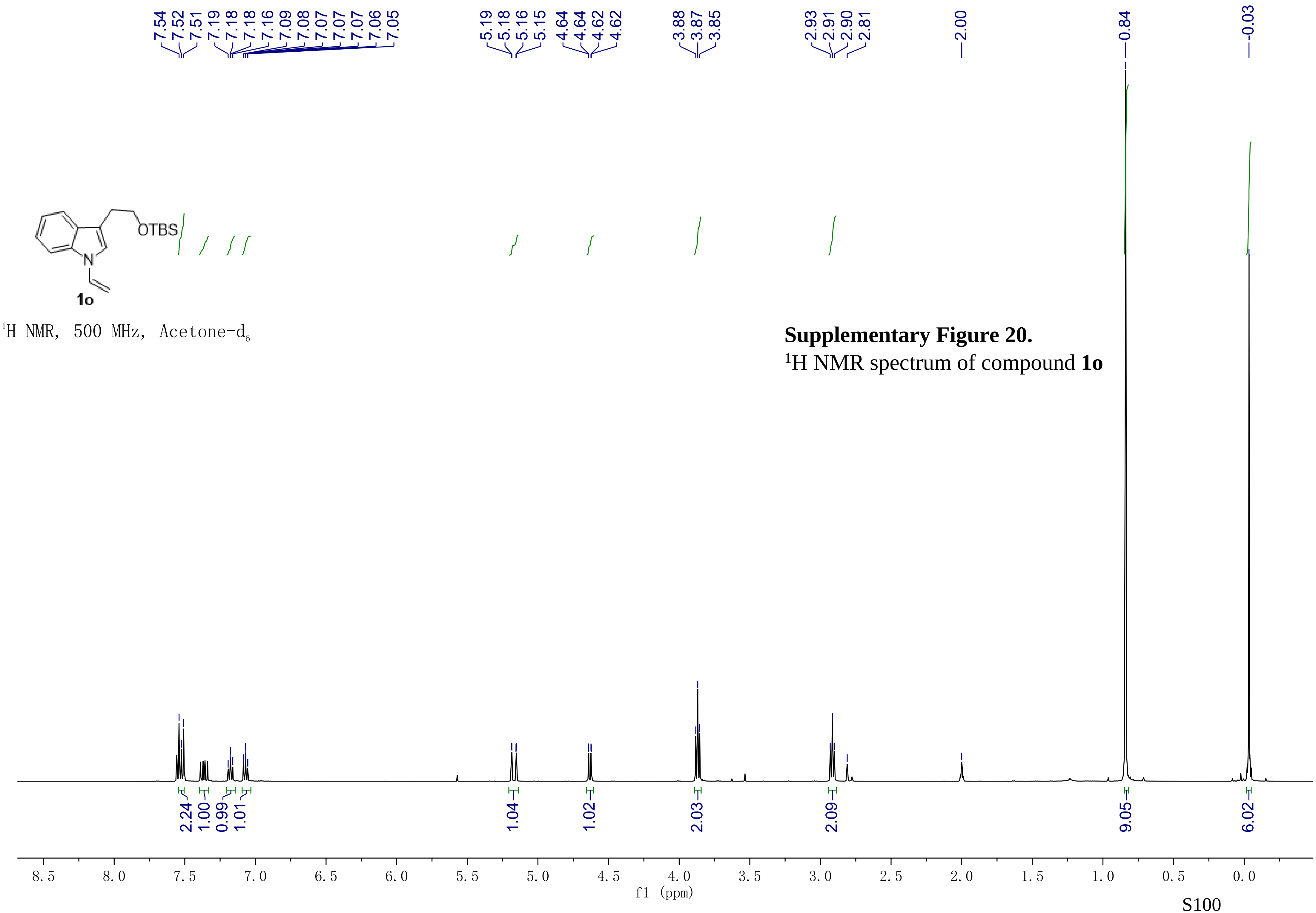

Supplementary Figure 20.
^{1}H NMR spectrum of compound **1o**

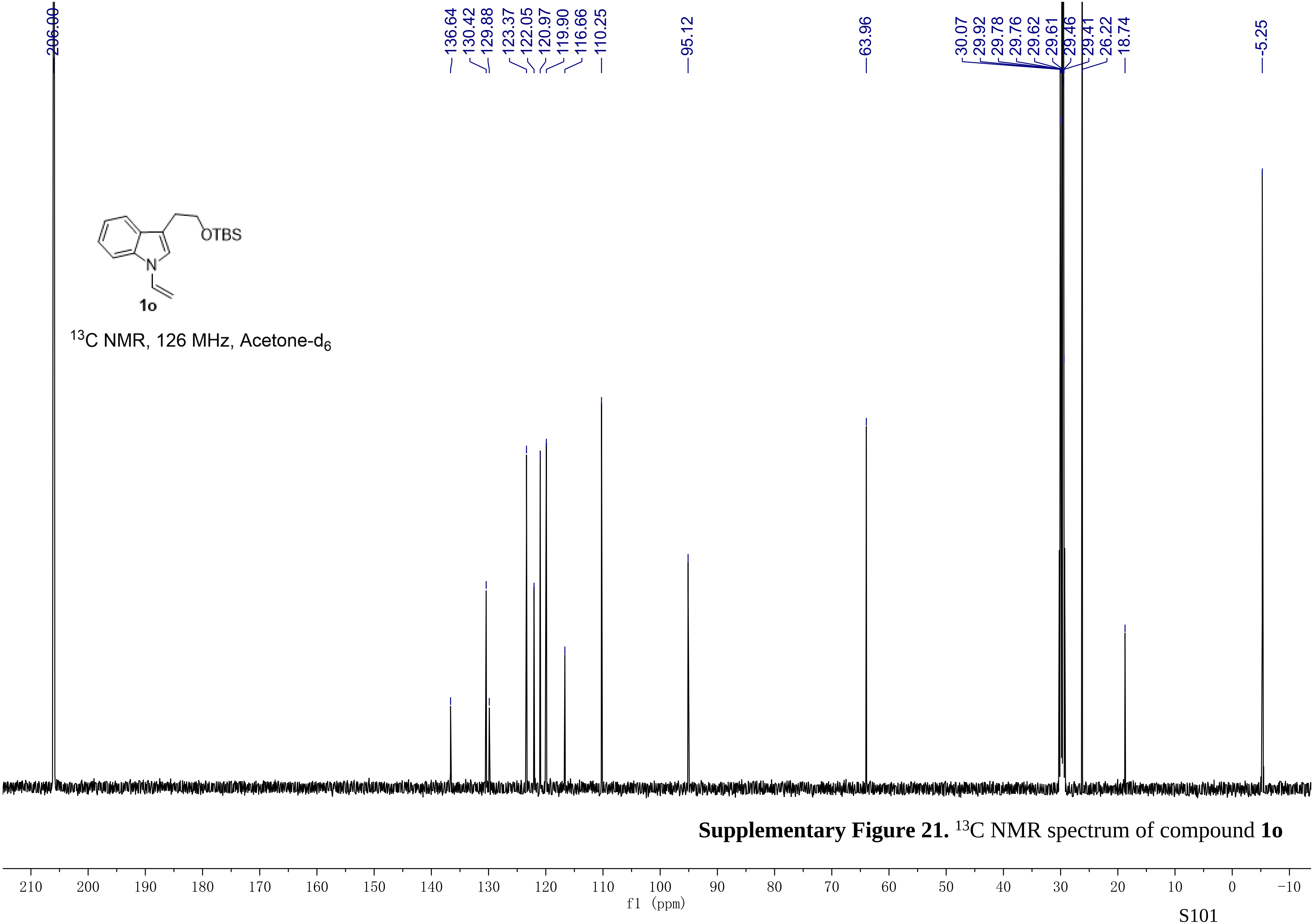

Supplementary Figure 21. ^{13}C NMR spectrum of compound **1o**

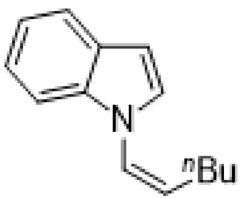

(*Z*)-**1p**

^{1}H NMR, 500 MHz, $CDCl_3$

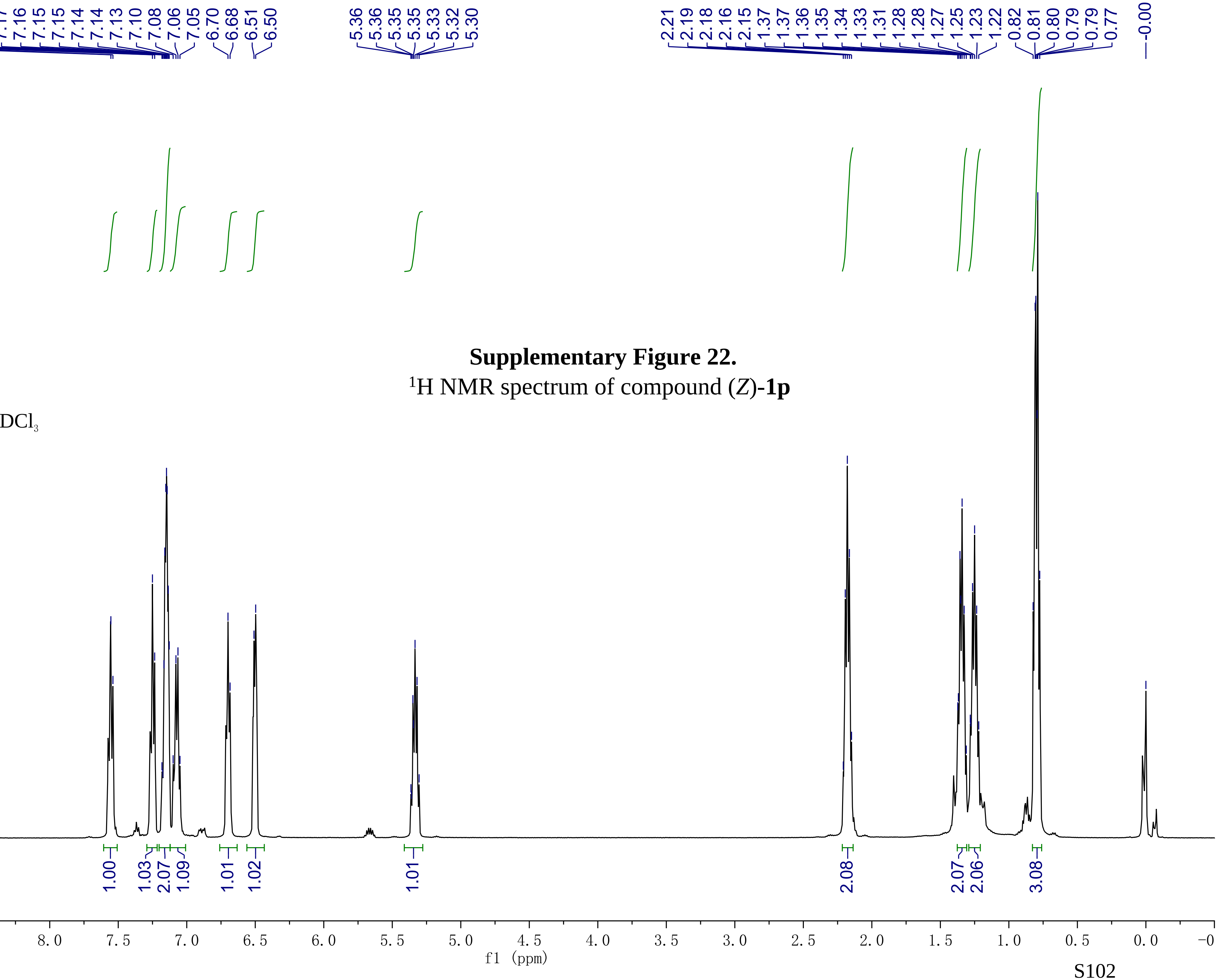

Supplementary Figure 22.
^{1}H NMR spectrum of compound (*Z*)-**1p**

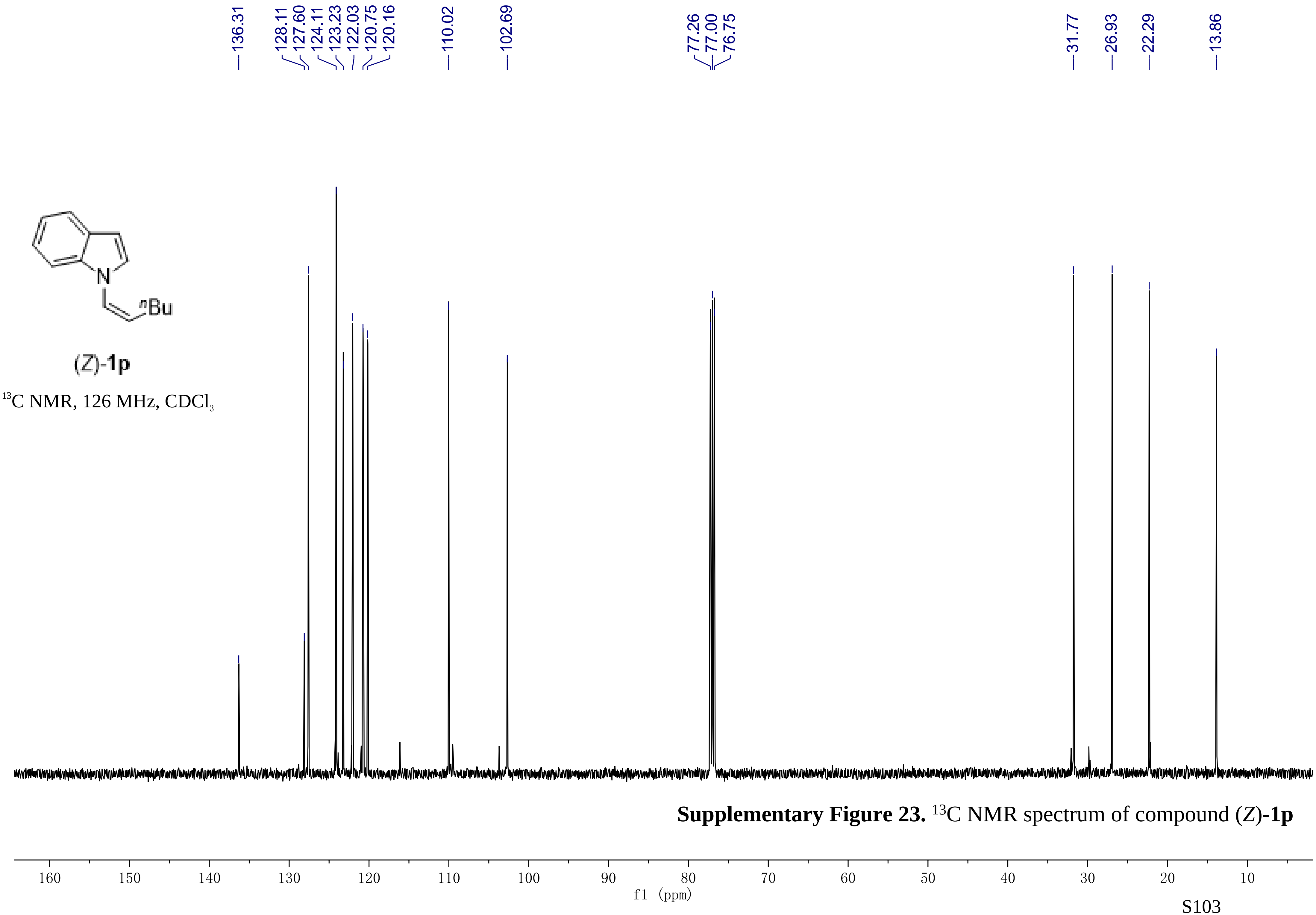

Supplementary Figure 23. ^{13}C NMR spectrum of compound (*Z*)-**1p**

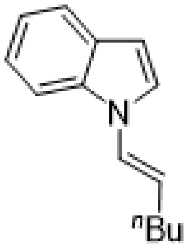

(*E*)-**1p**

^{1}H NMR, 400 MHz, $CDCl_3$

Supplementary Figure 24.
^{1}H NMR spectrum of compound (*E*)-**1p**

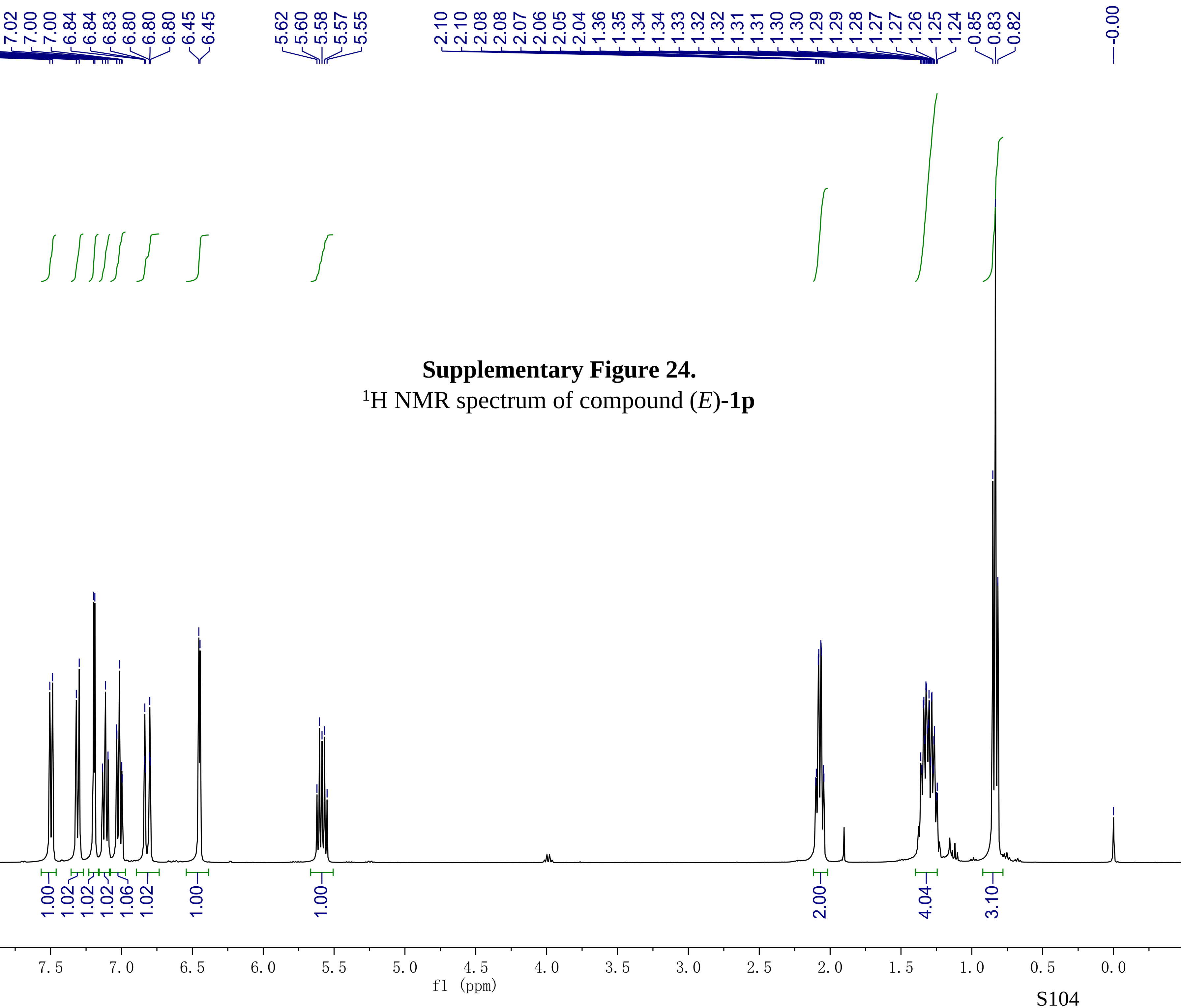

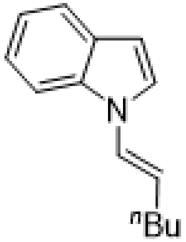

(*E*)-**1p**

^{13}C NMR, 101 MHz, $CDCl_3$

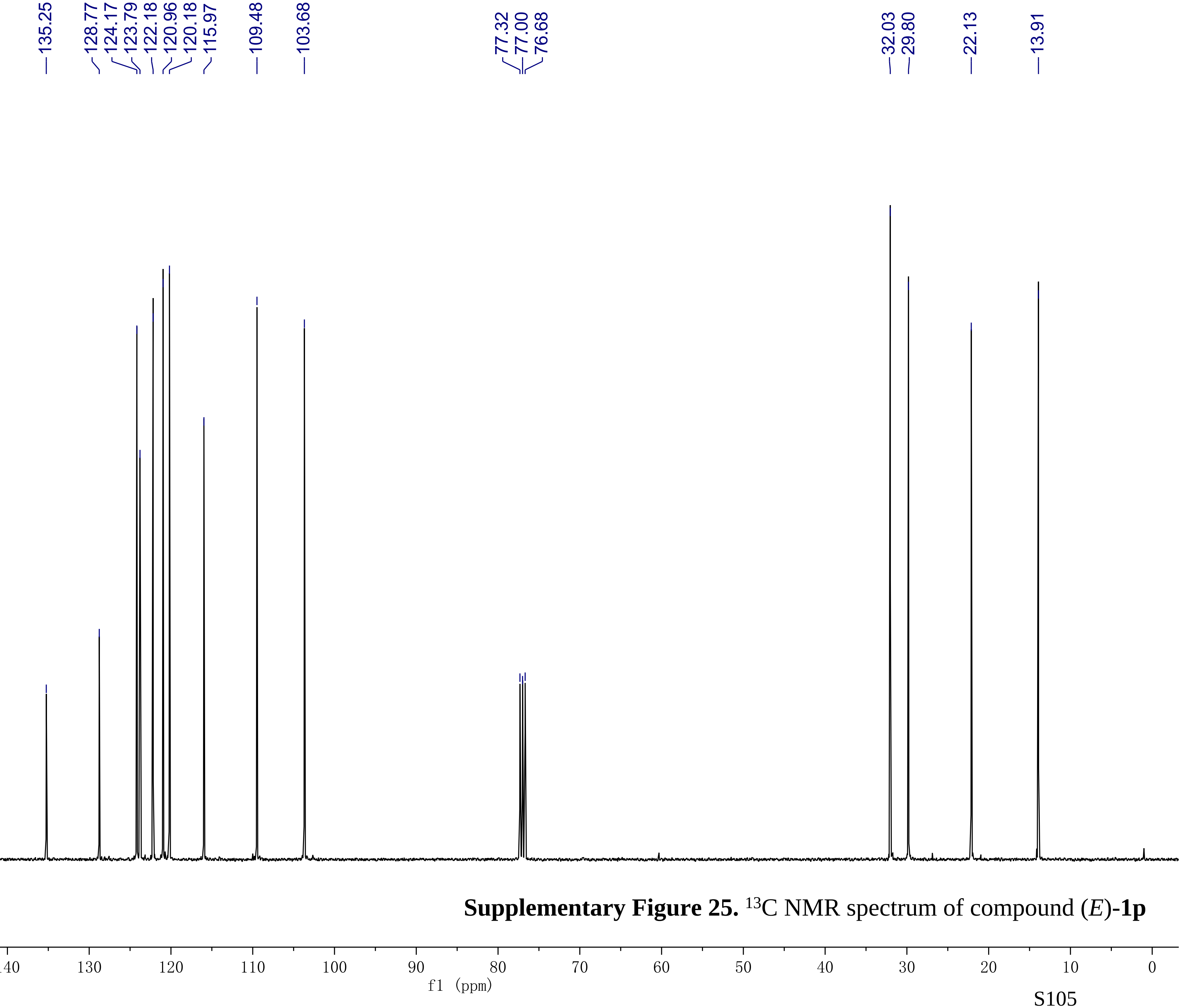

Supplementary Figure 25. ^{13}C NMR spectrum of compound (*E*)-**1p**

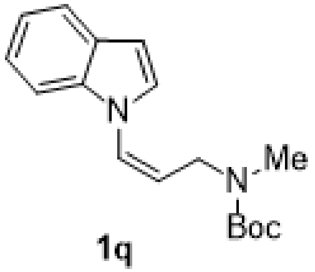

1q

^{1}H NMR, 500 MHz, $CDCl_3$

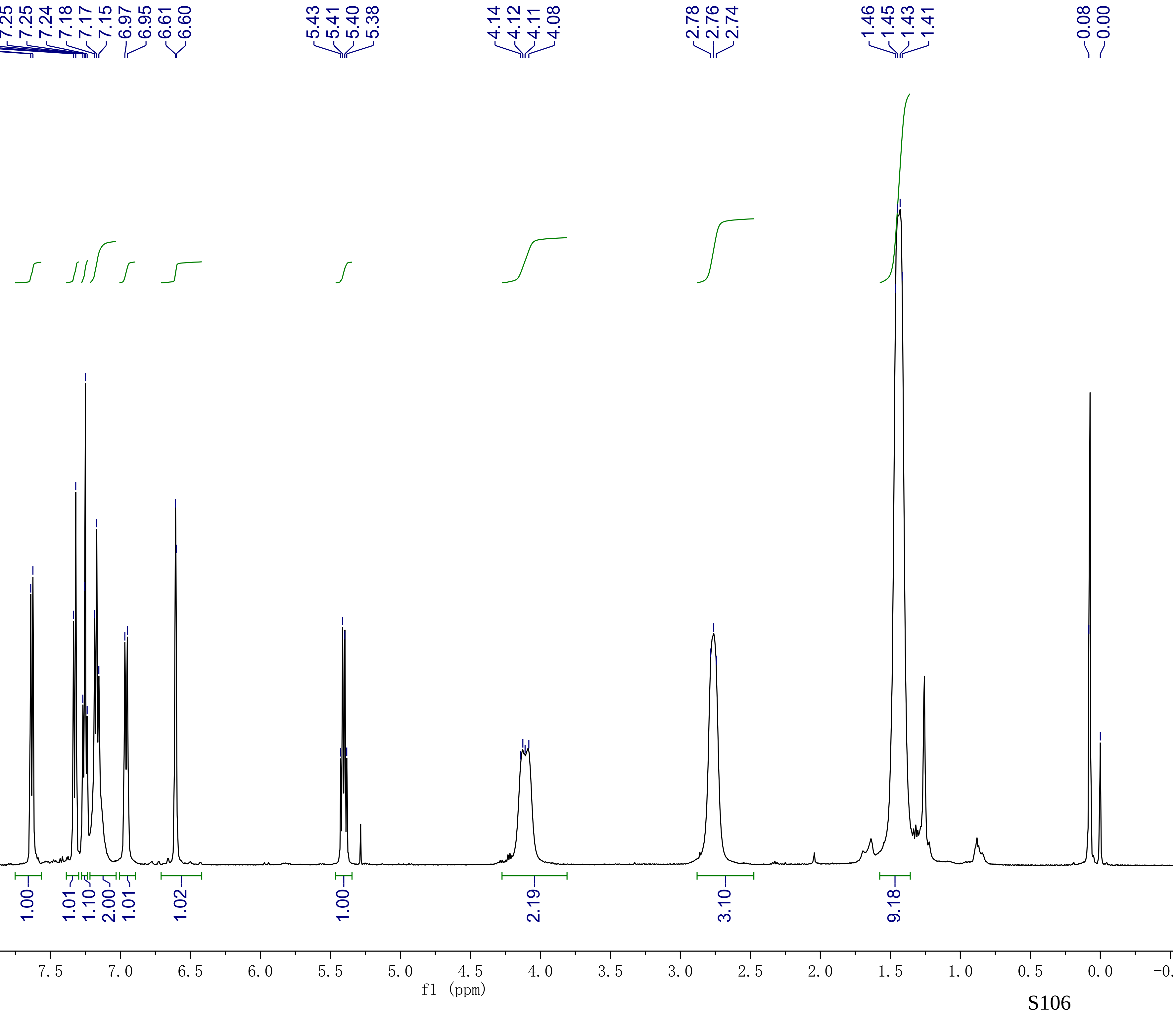

Supplementary Figure 26. ^{1}H NMR spectrum of compound **1q**

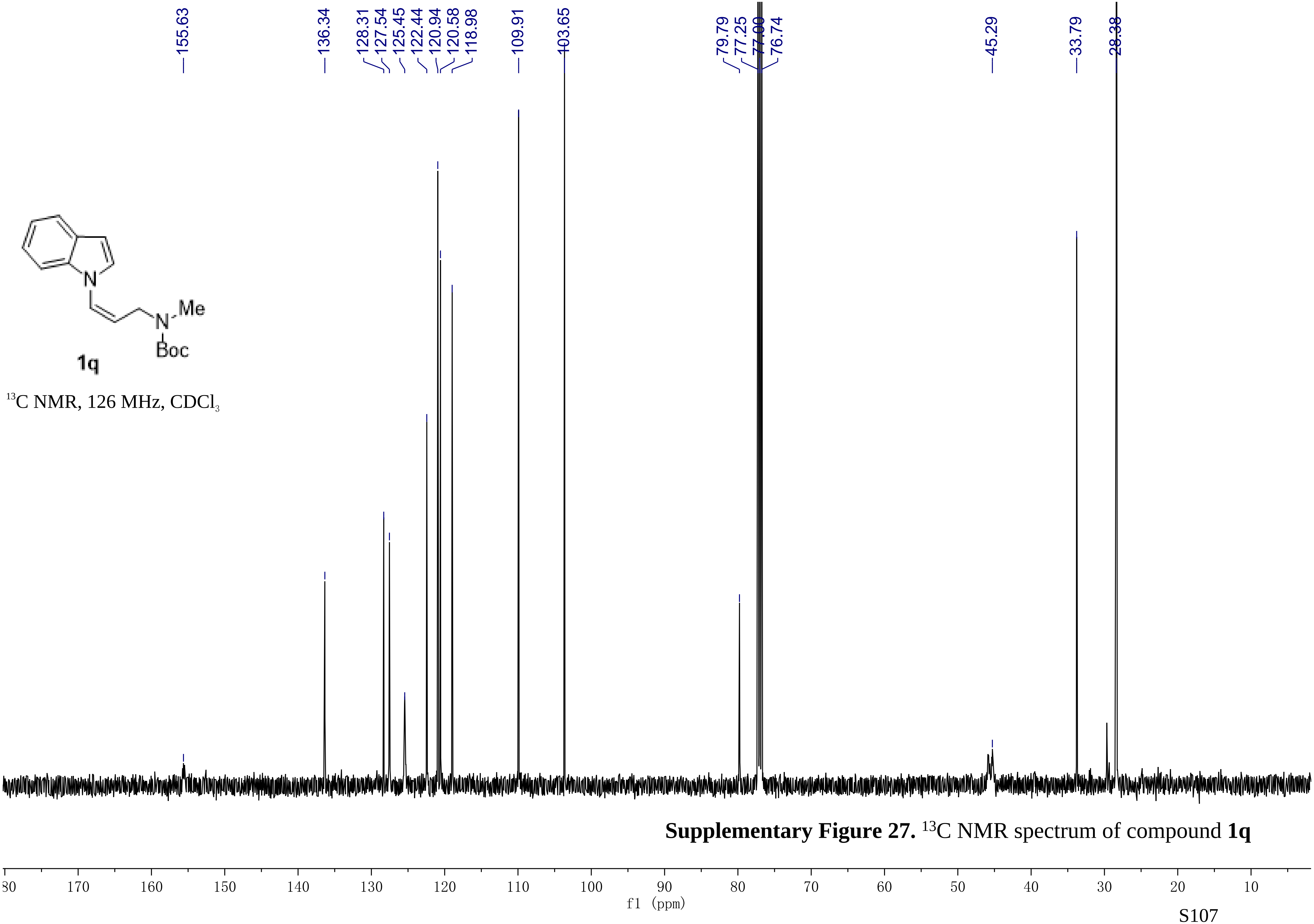

Supplementary Figure 27. ^{13}C NMR spectrum of compound **1q**

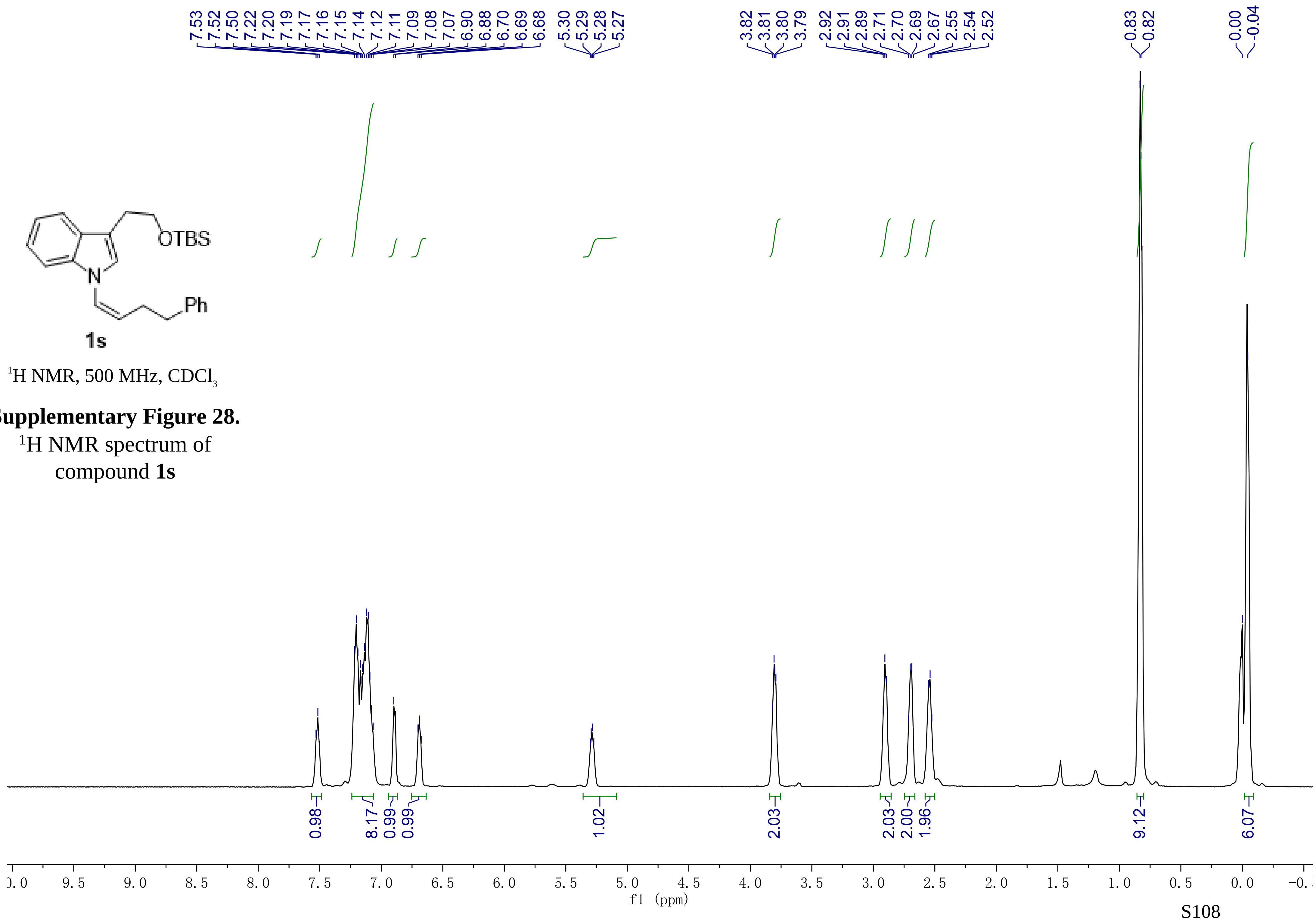

^{1}H NMR, 500 MHz, $CDCl_3$

Supplementary Figure 28. ^{1}H NMR spectrum of compound **1s**

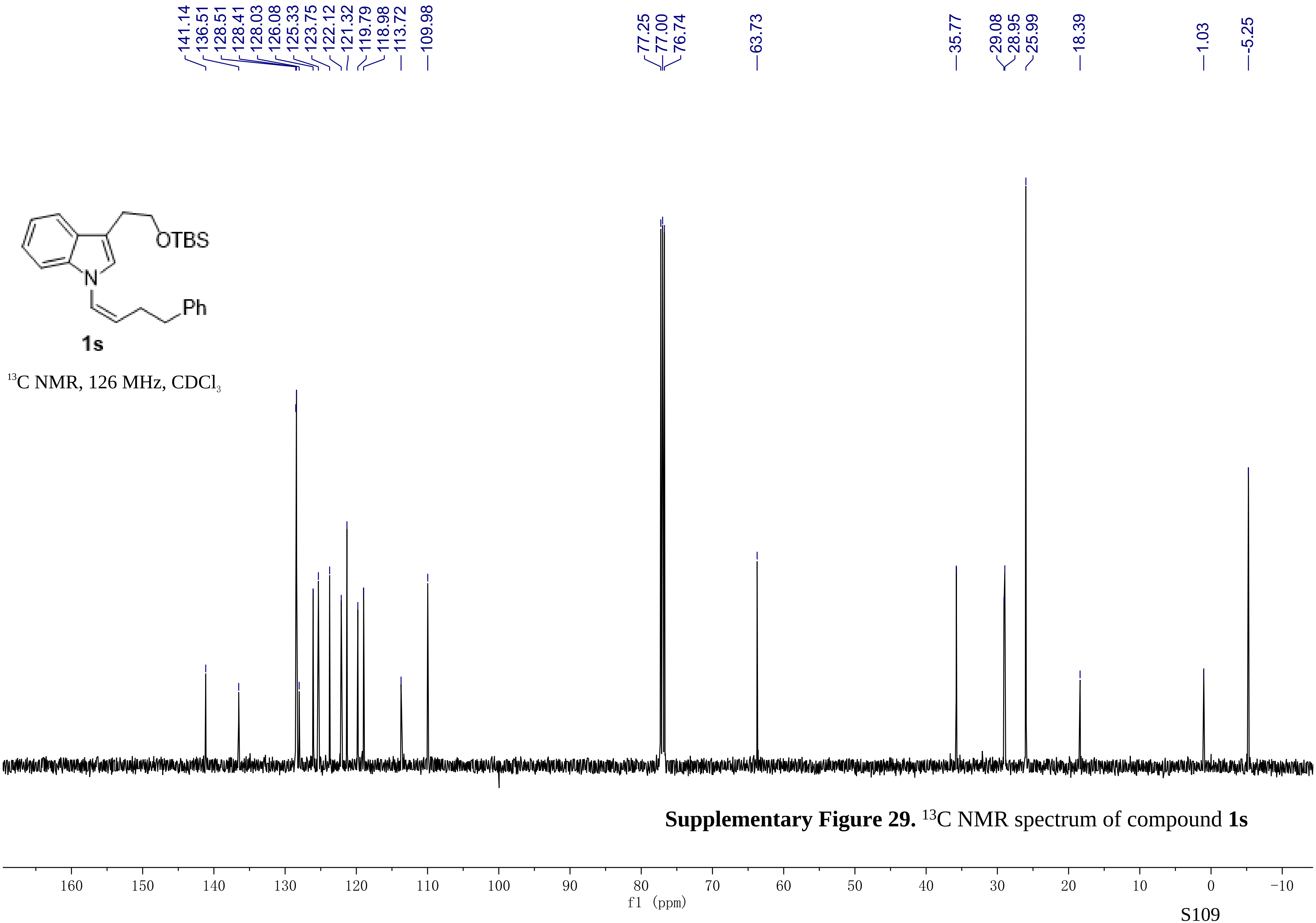

1s

^{13}C NMR, 126 MHz, $CDCl_3$

Supplementary Figure 29. ^{13}C NMR spectrum of compound **1s**

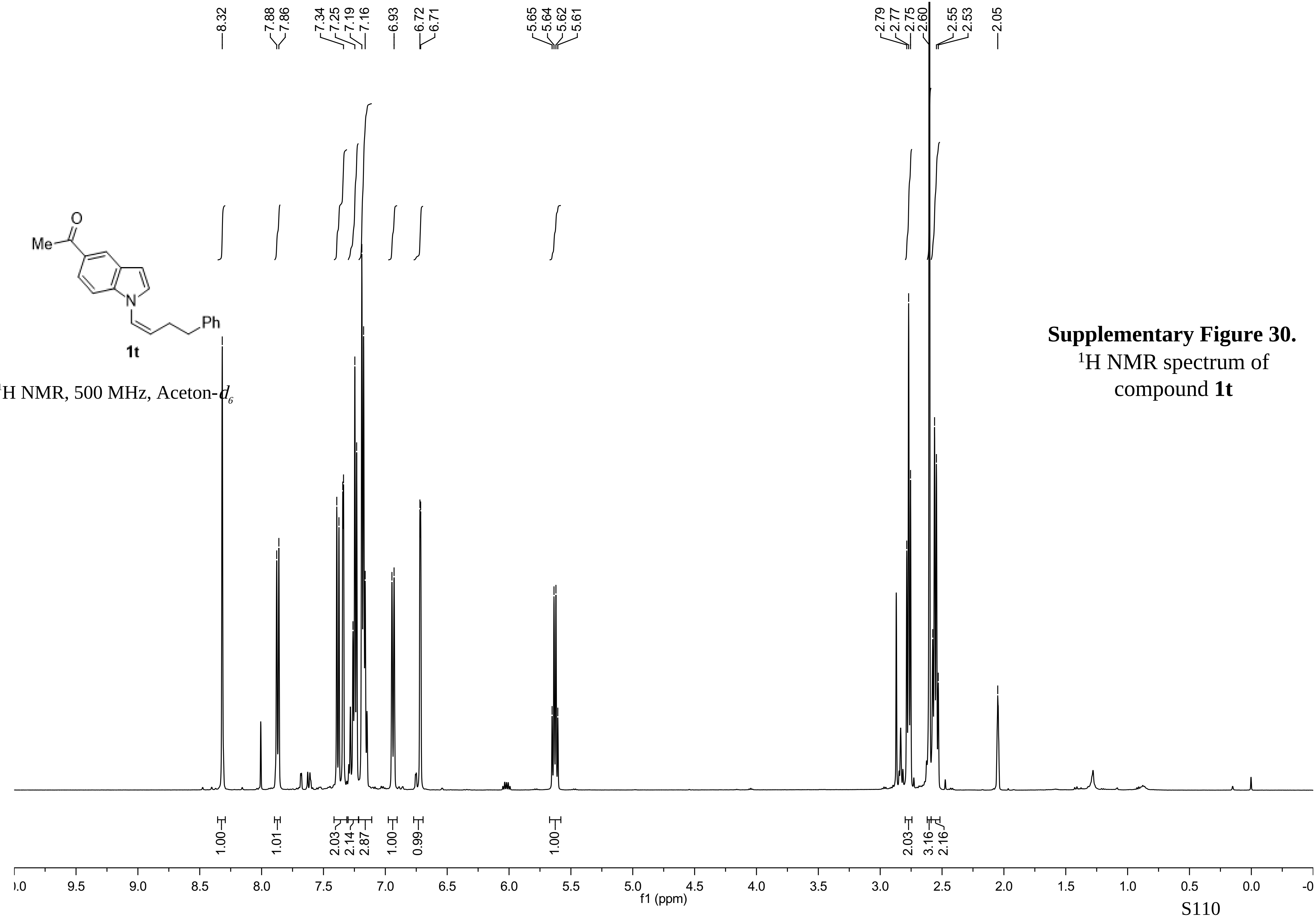

Supplementary Figure 30. ^{1}H NMR spectrum of compound **1t**

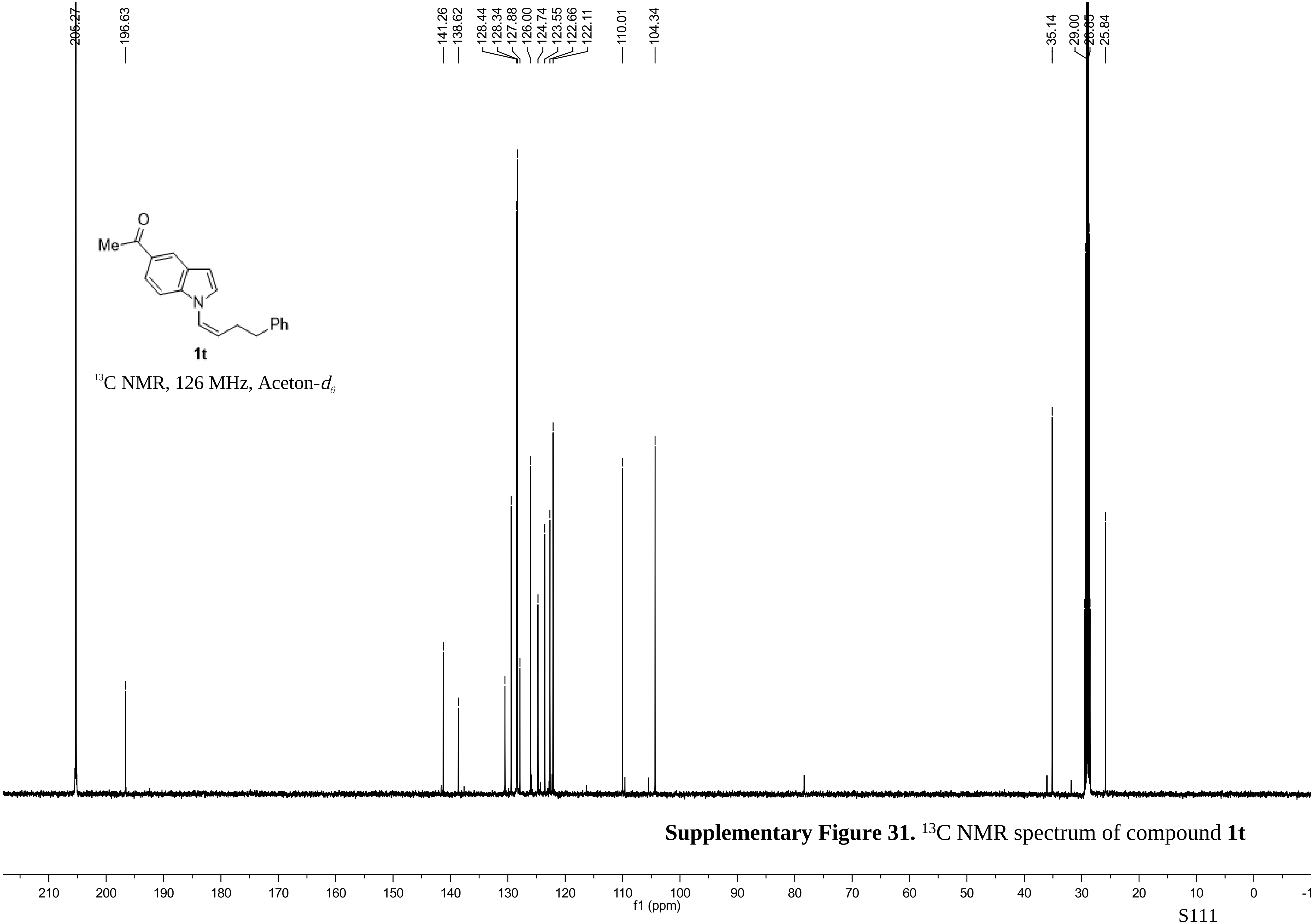

Supplementary Figure 31. ^{13}C NMR spectrum of compound **1t**

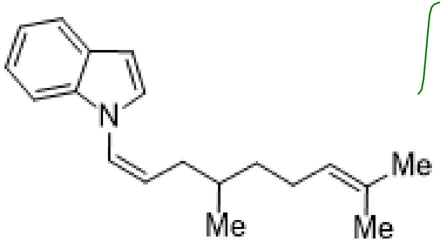

1u

^1H NMR, 500 MHz, $CDCl_3$

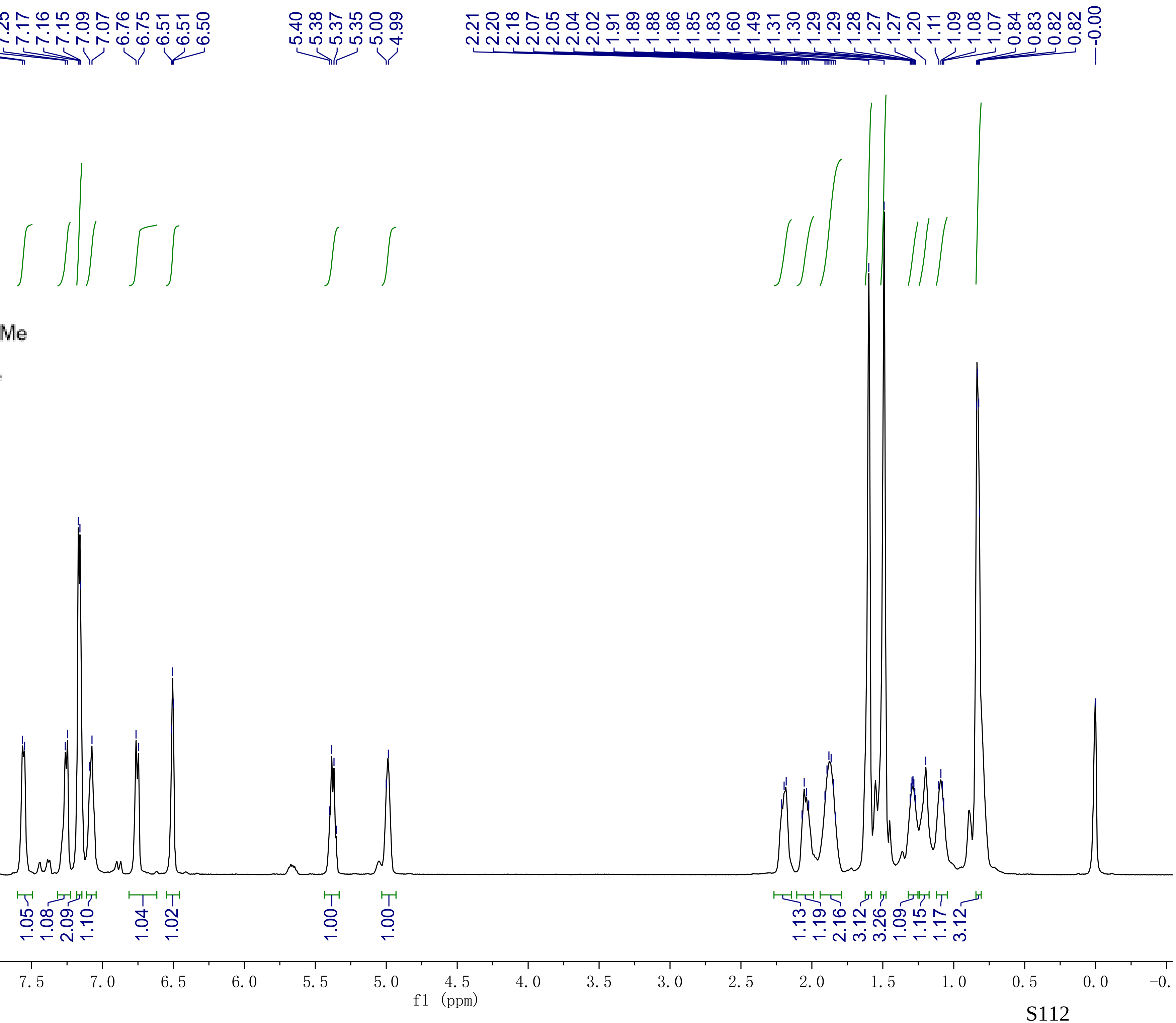

Supplementary Figure 32.
^1H NMR spectrum of compound **1u**

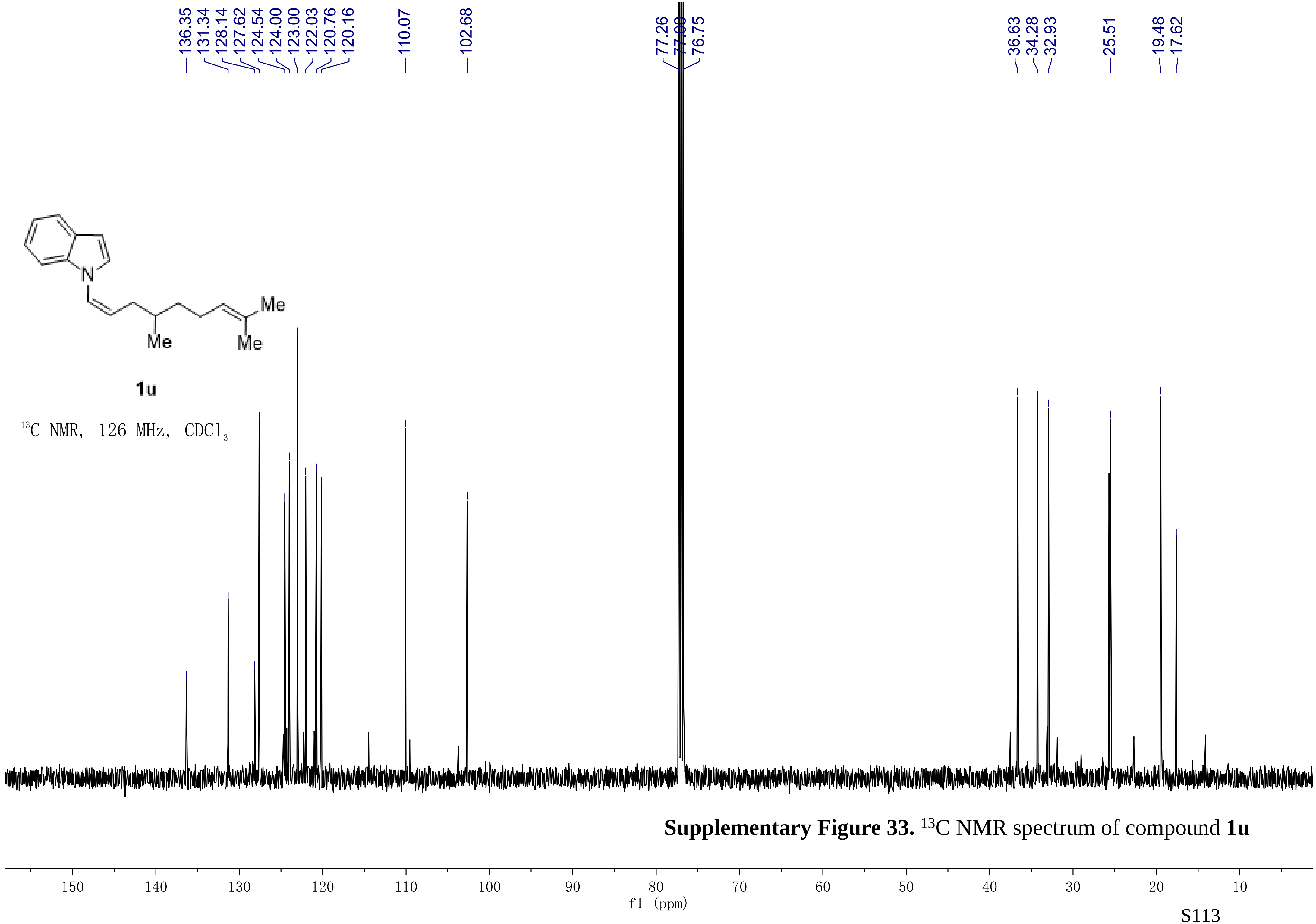

Supplementary Figure 33. ^{13}C NMR spectrum of compound **1u**

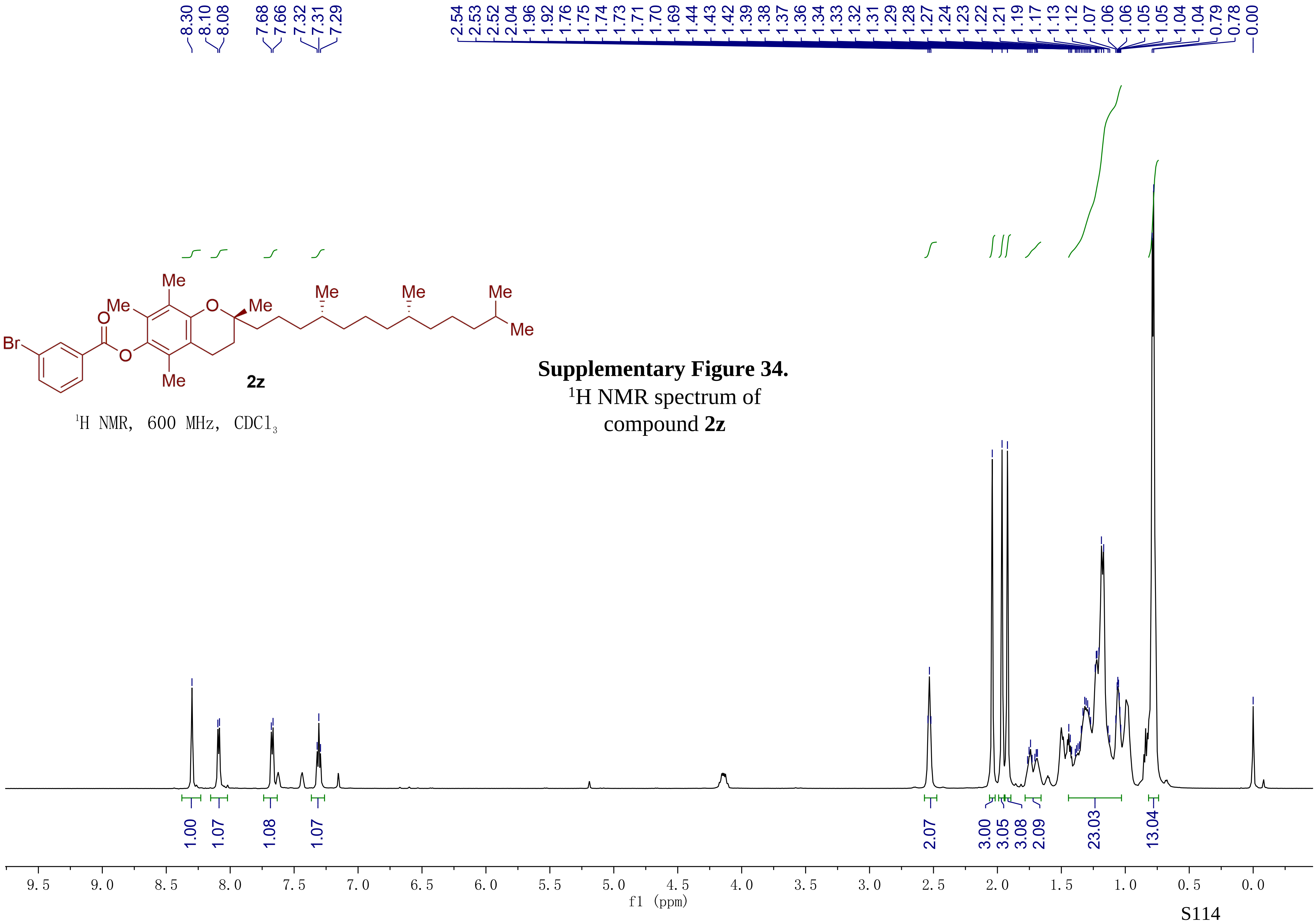

Supplementary Figure 34.
^{1}H NMR spectrum of compound **2z**

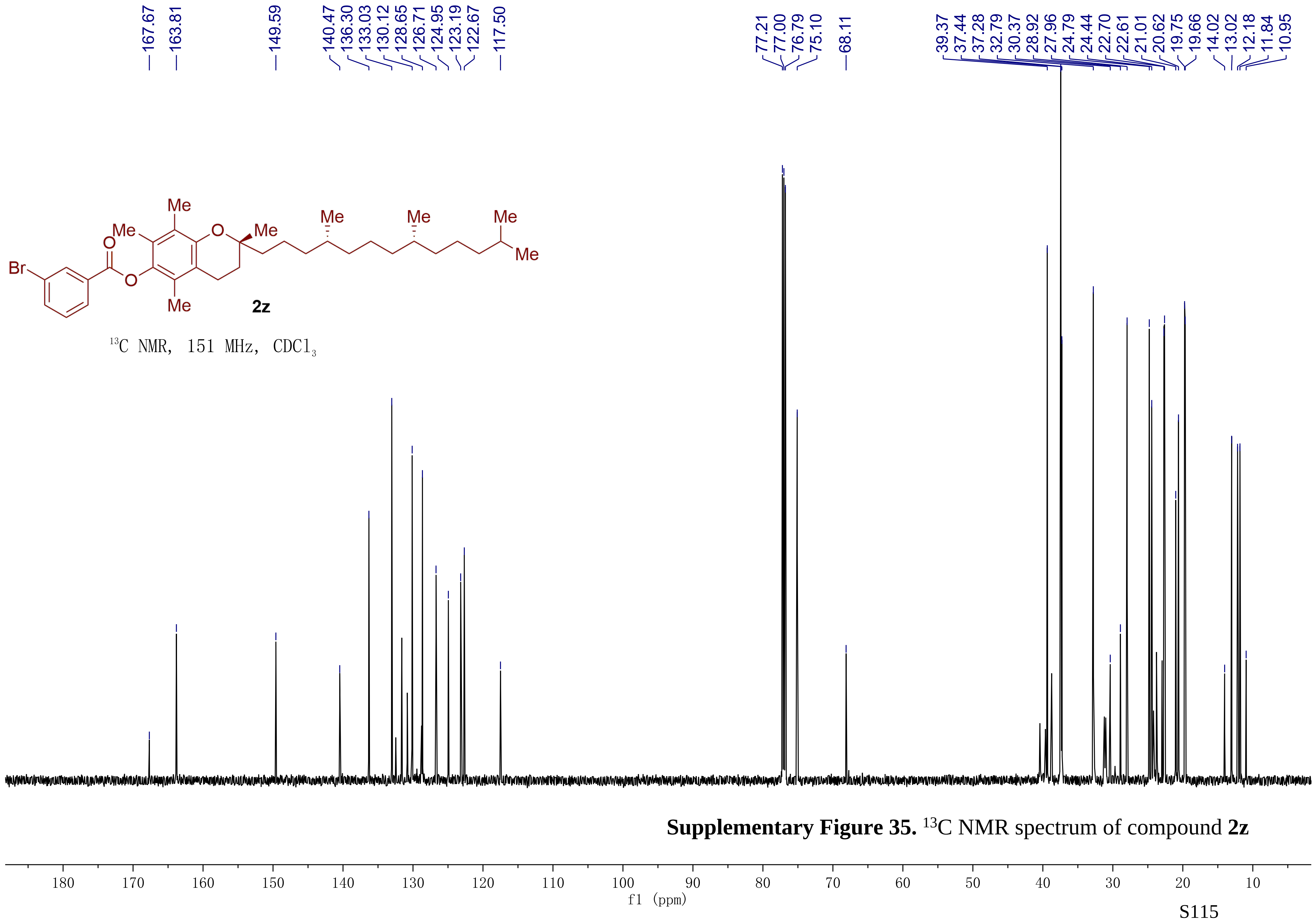

Supplementary Figure 35. ^{13}C NMR spectrum of compound **2z**

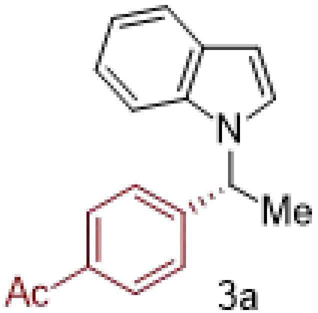

3a

^{1}H NMR, 500 MHz, $CDCl_3$

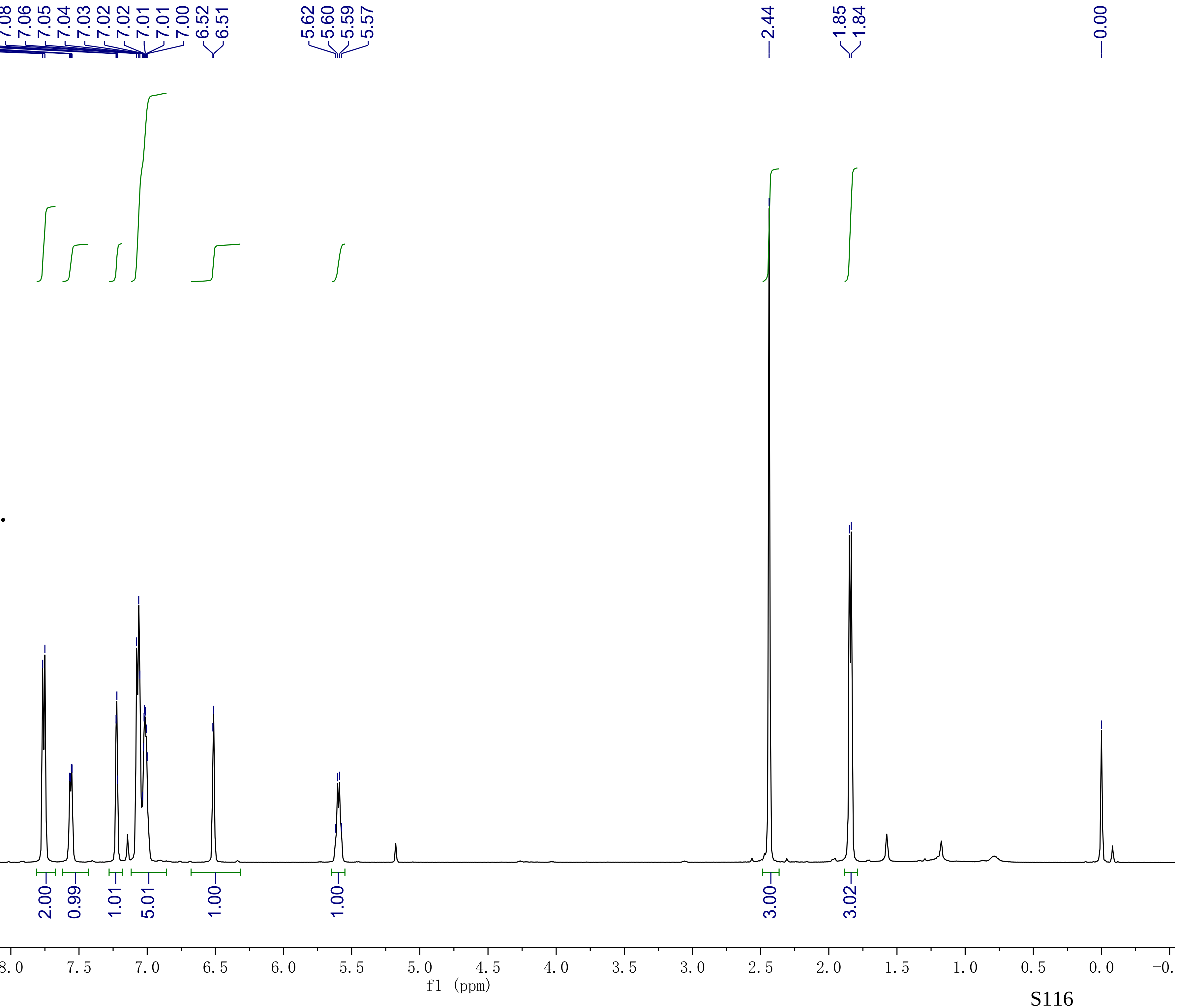

Supplementary Figure 36. ^{1}H NMR spectrum of compound **3a**

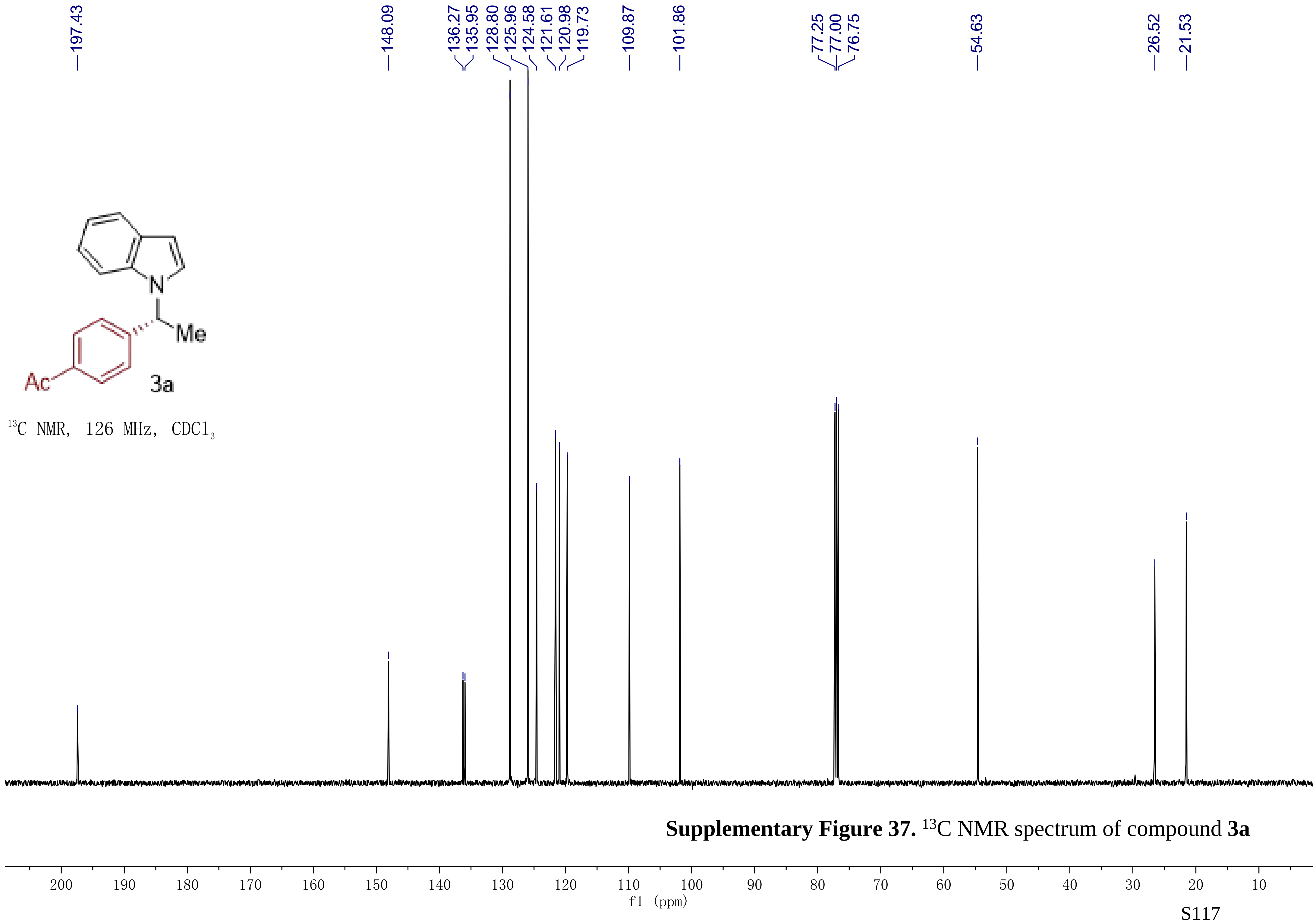

Supplementary Figure 37. ^{13}C NMR spectrum of compound **3a**

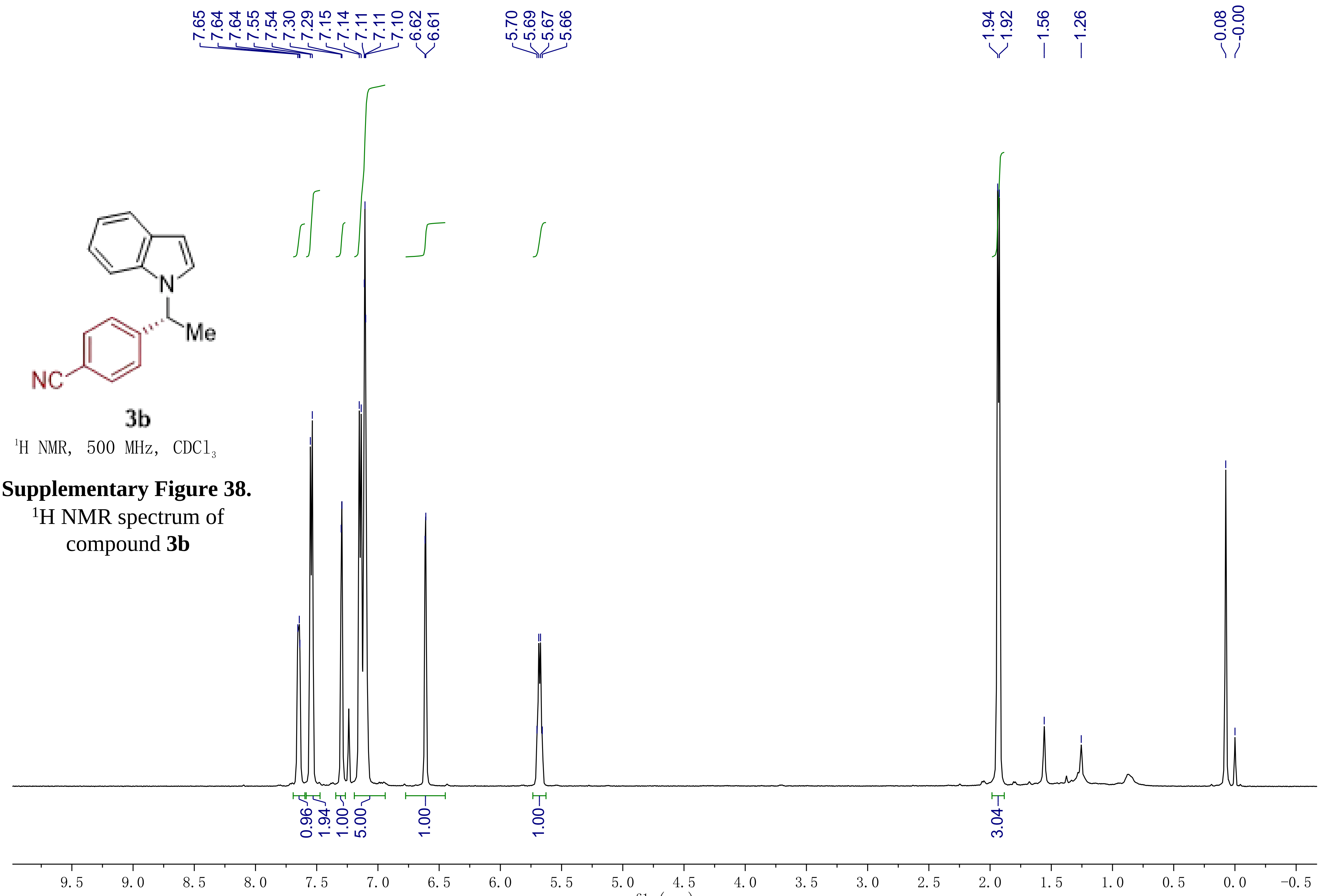

Supplementary Figure 38. ¹H NMR spectrum of compound **3b**

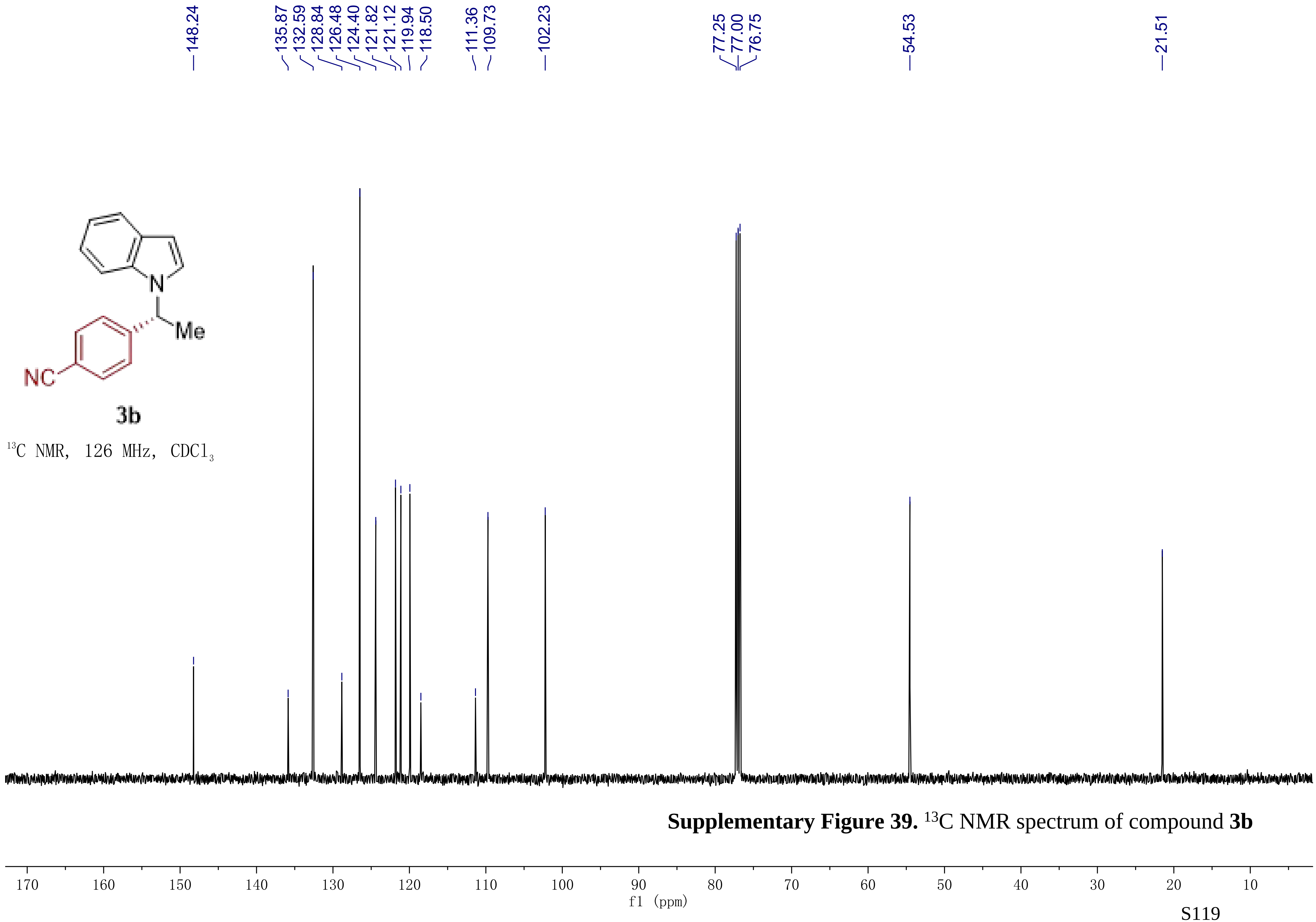

Supplementary Figure 39. ^{13}C NMR spectrum of compound **3b**

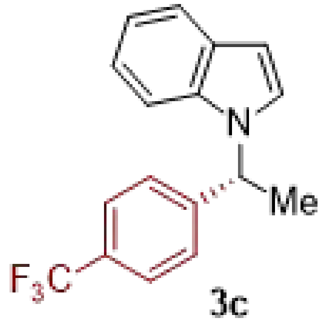

3c

^{1}H NMR, 500 MHz, $CDCl_3$

Supplementary Figure 40. ^{1}H NMR spectrum of compound **3c**

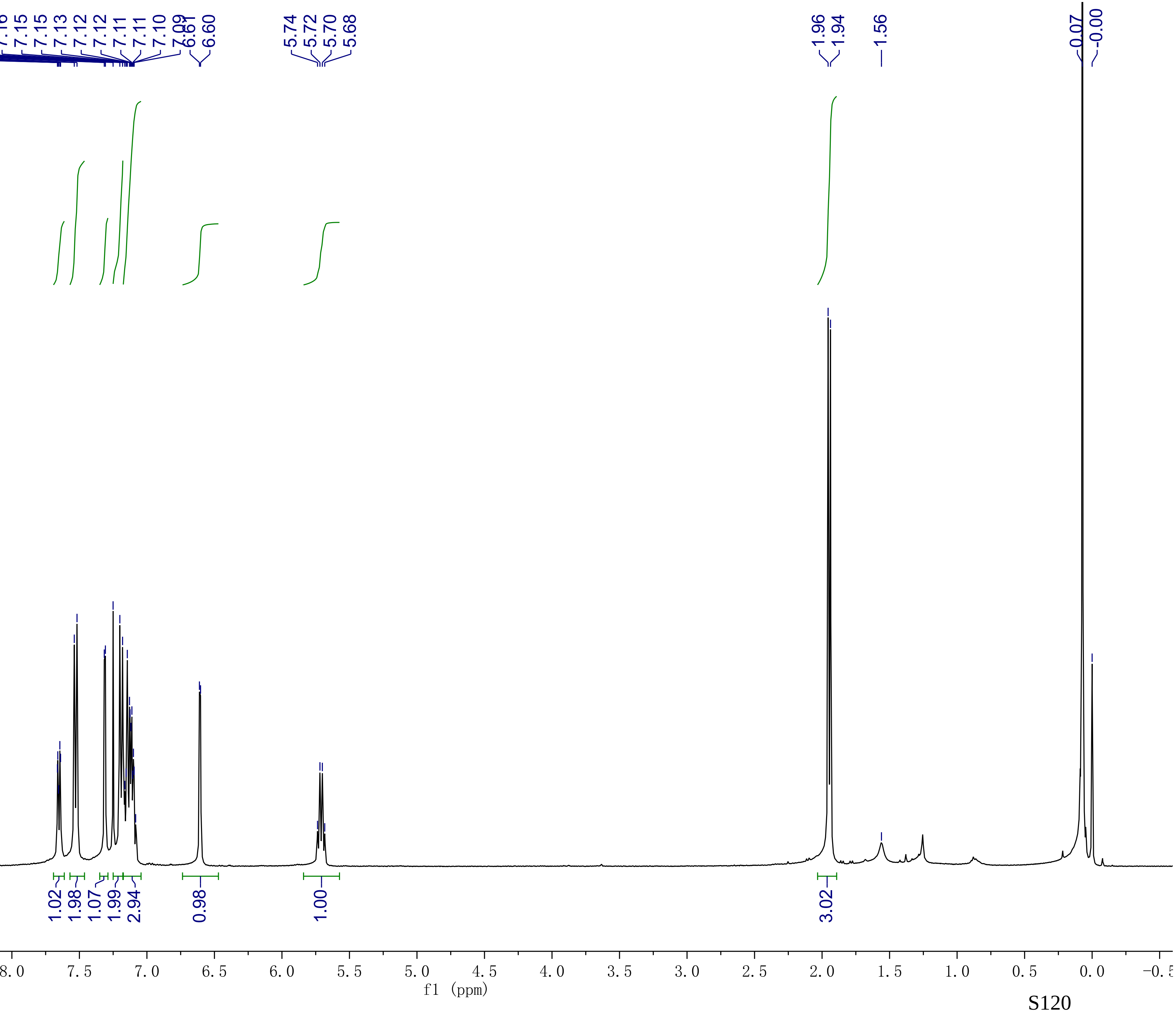

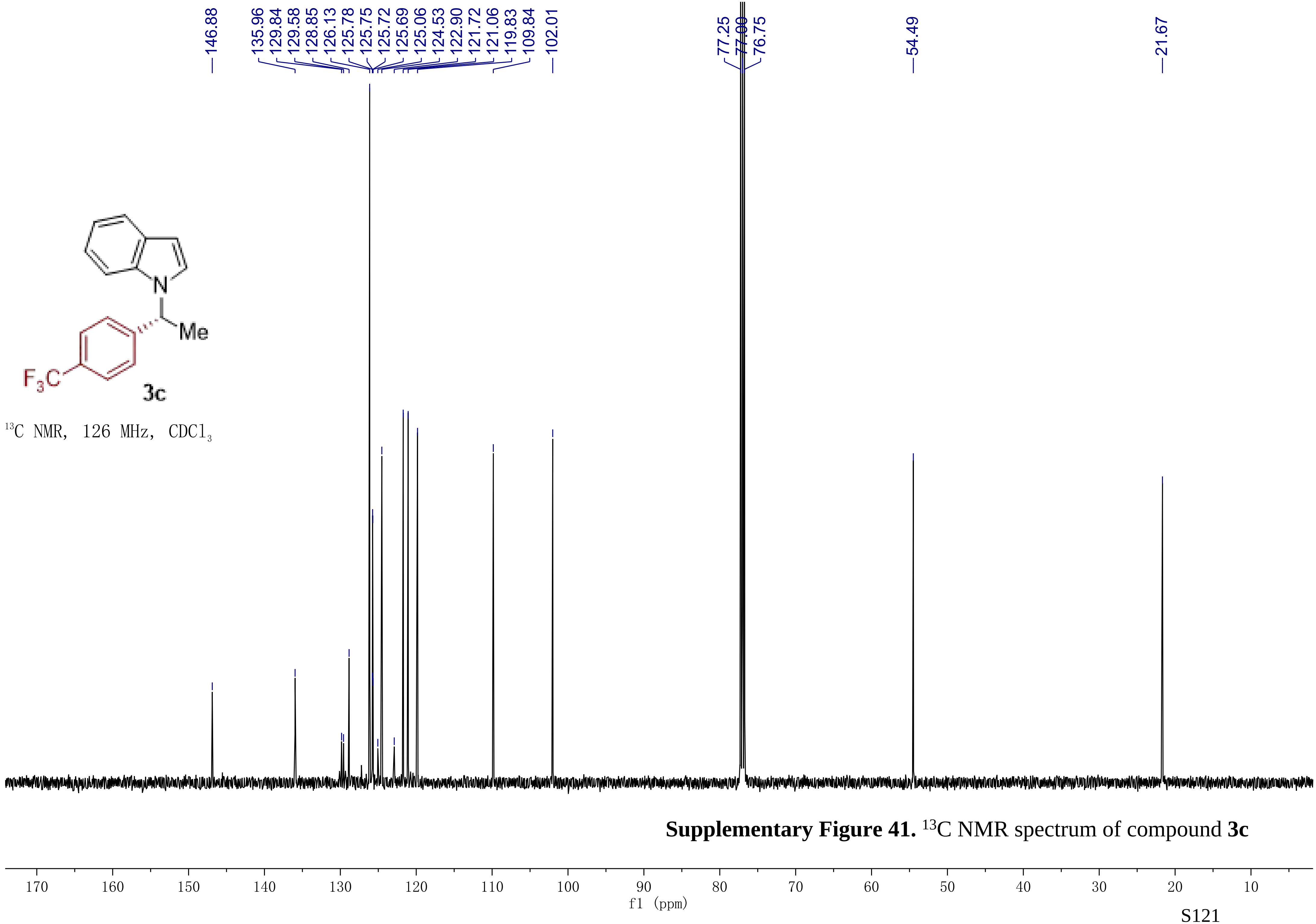

Supplementary Figure 41. ^{13}C NMR spectrum of compound **3c**

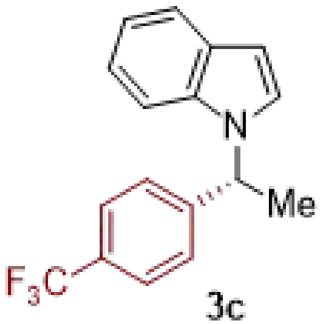

3c

^{19}F NMR, 471 MHz, $CDCl_3$

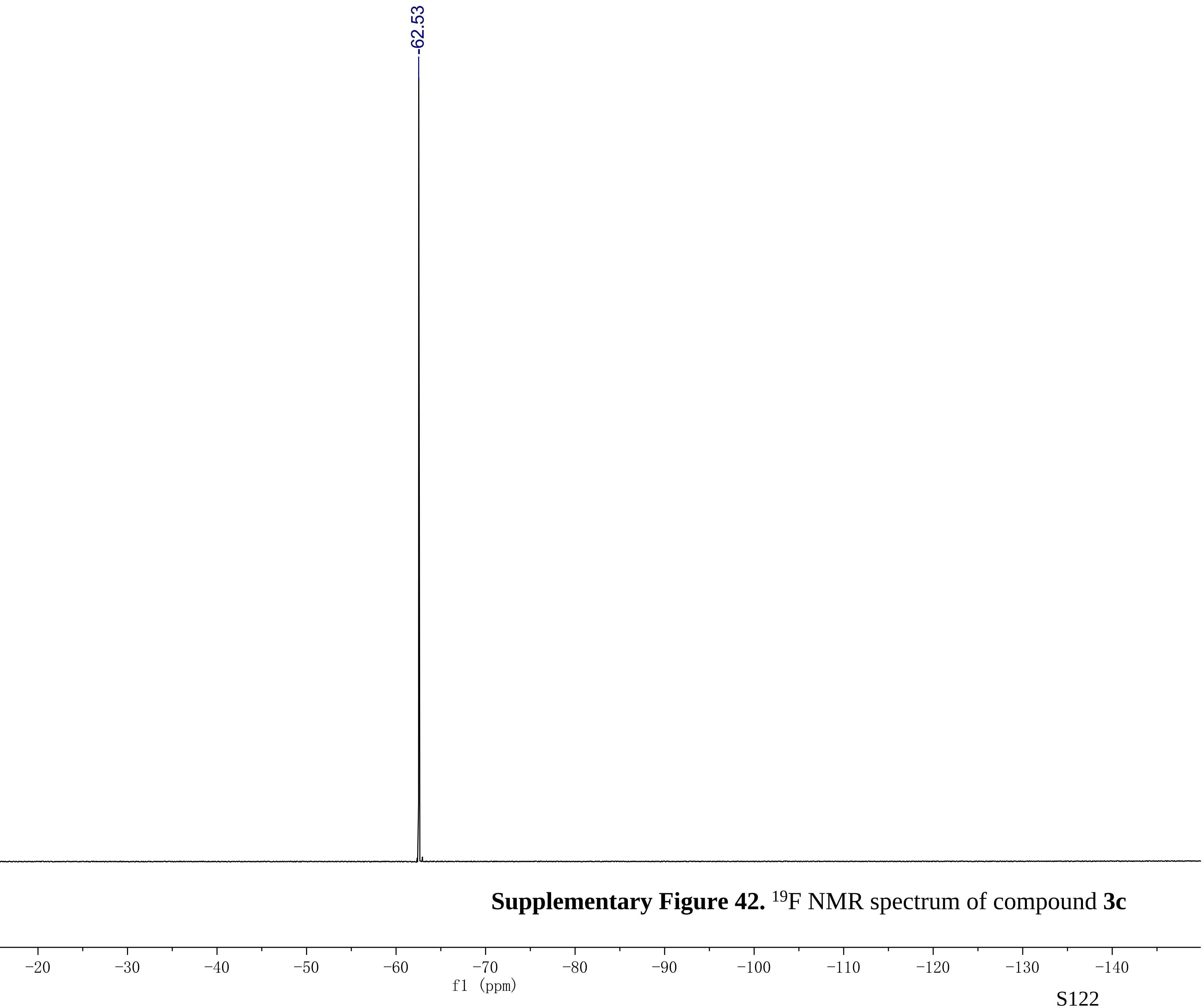

Supplementary Figure 42. ^{19}F NMR spectrum of compound **3c**

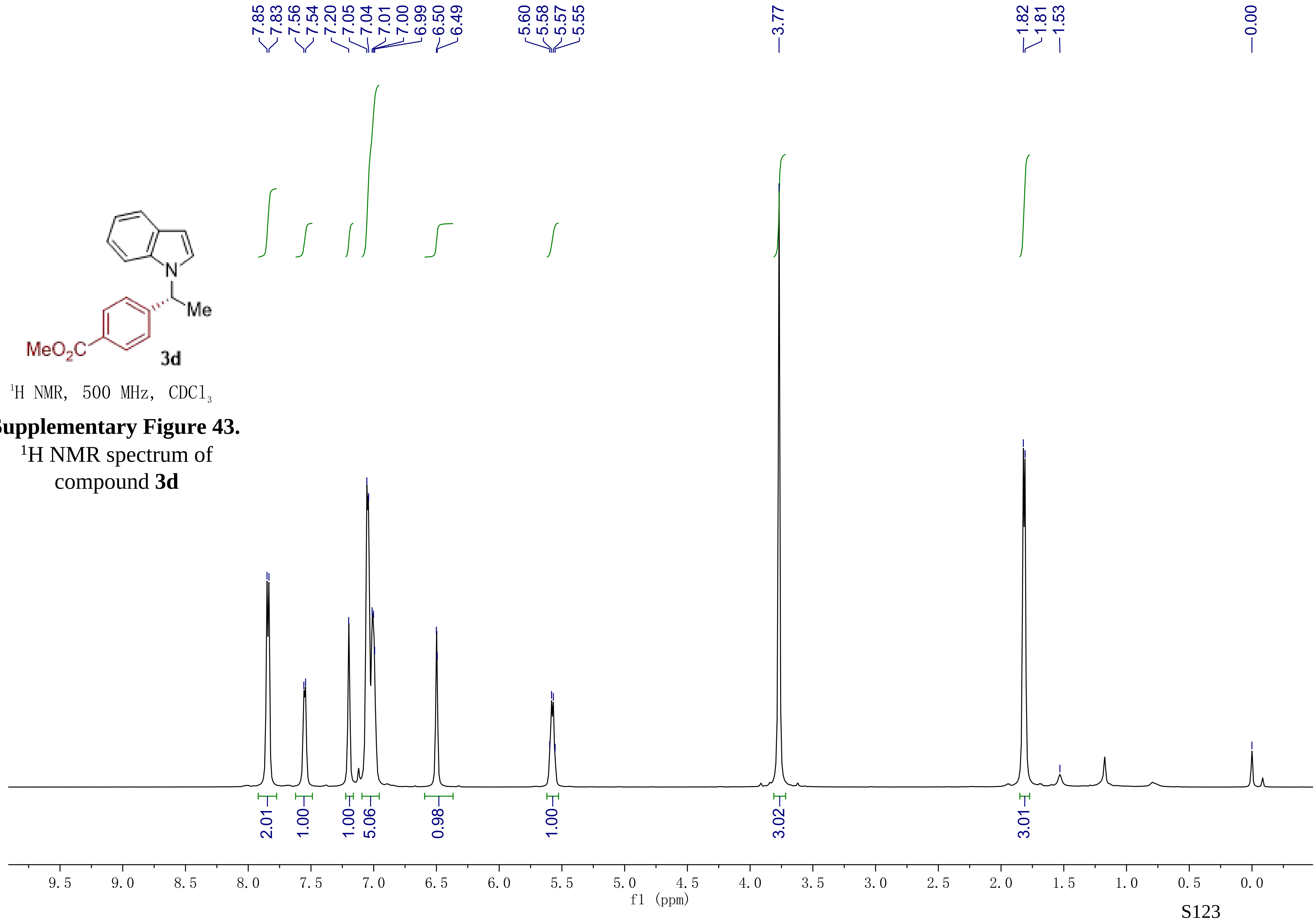

Supplementary Figure 43. ^{1}H NMR spectrum of compound **3d**

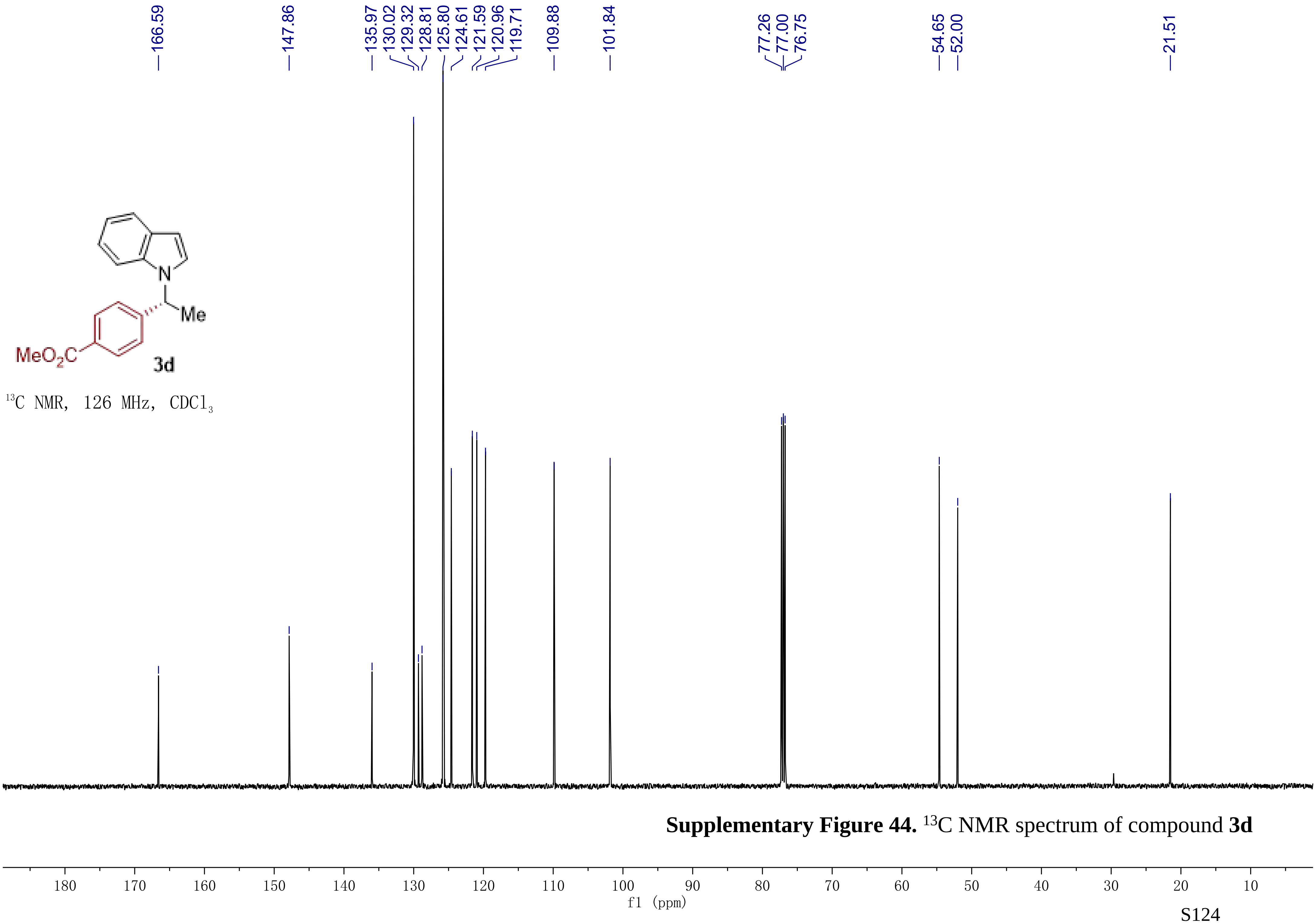

Supplementary Figure 44. ^{13}C NMR spectrum of compound **3d**

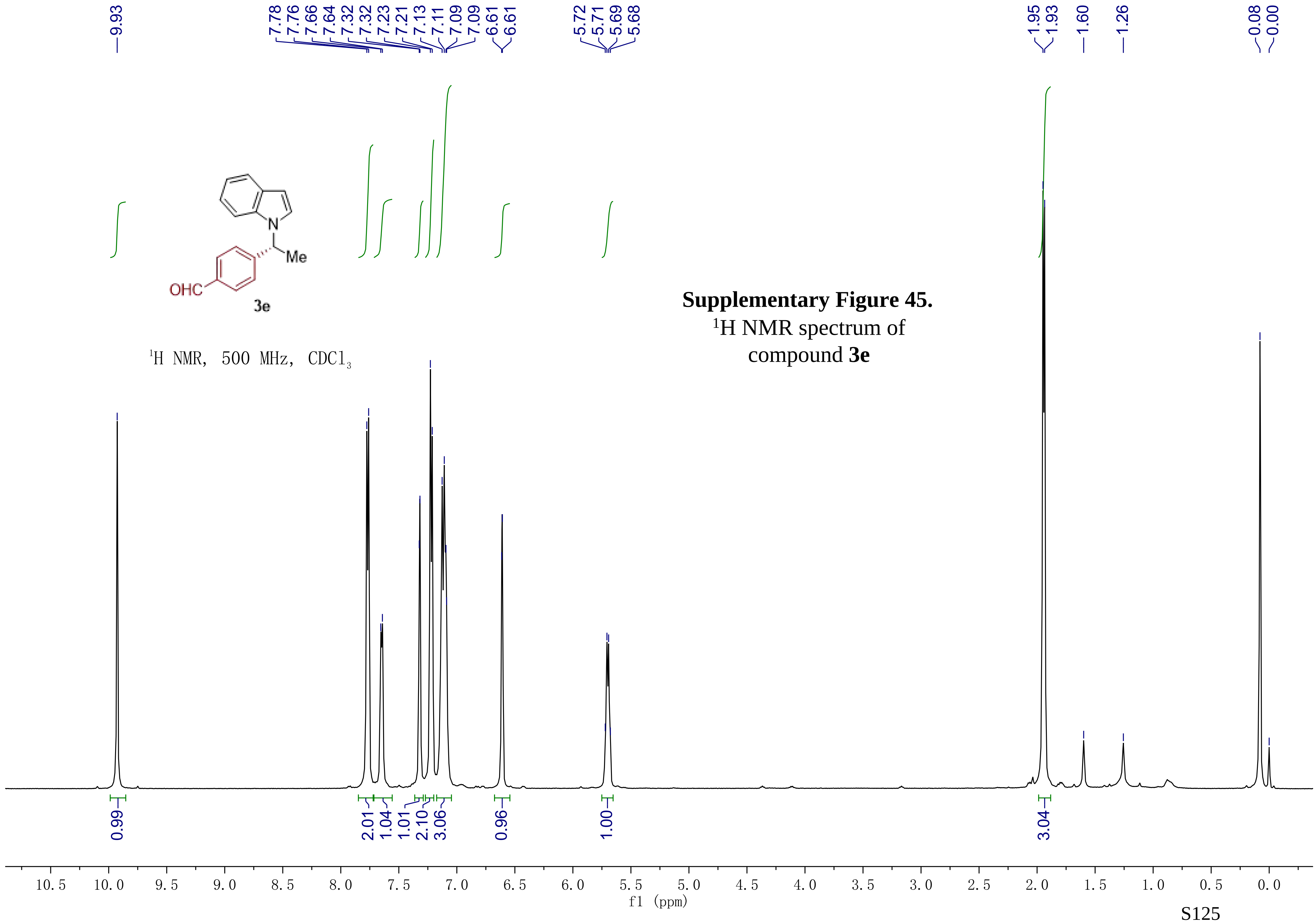

Supplementary Figure 45.
^{1}H NMR spectrum of compound **3e**

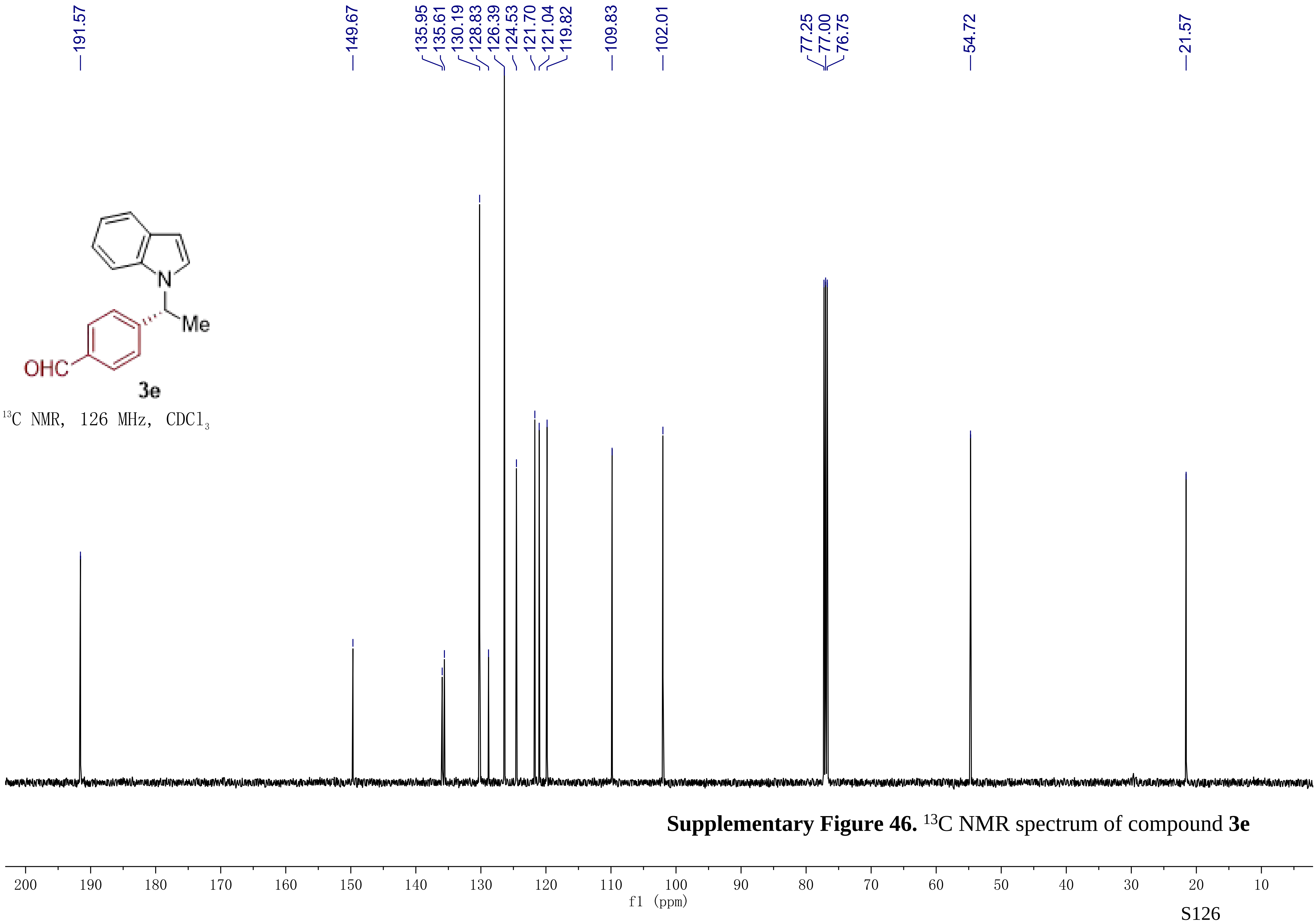

Supplementary Figure 46. ^{13}C NMR spectrum of compound **3e**

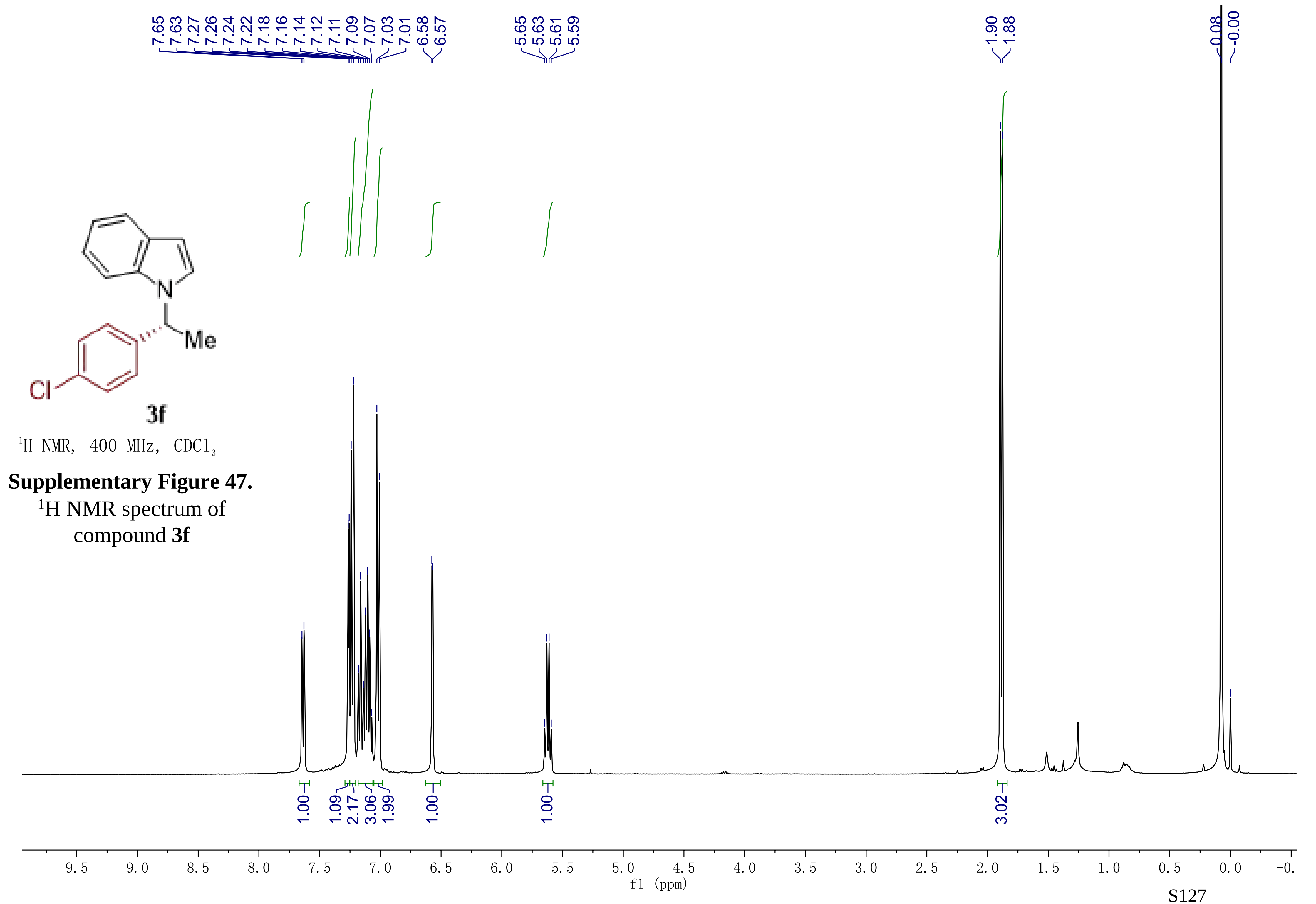

^{1}H NMR, 400 MHz, $CDCl_3$

Supplementary Figure 47. ^{1}H NMR spectrum of compound **3f**

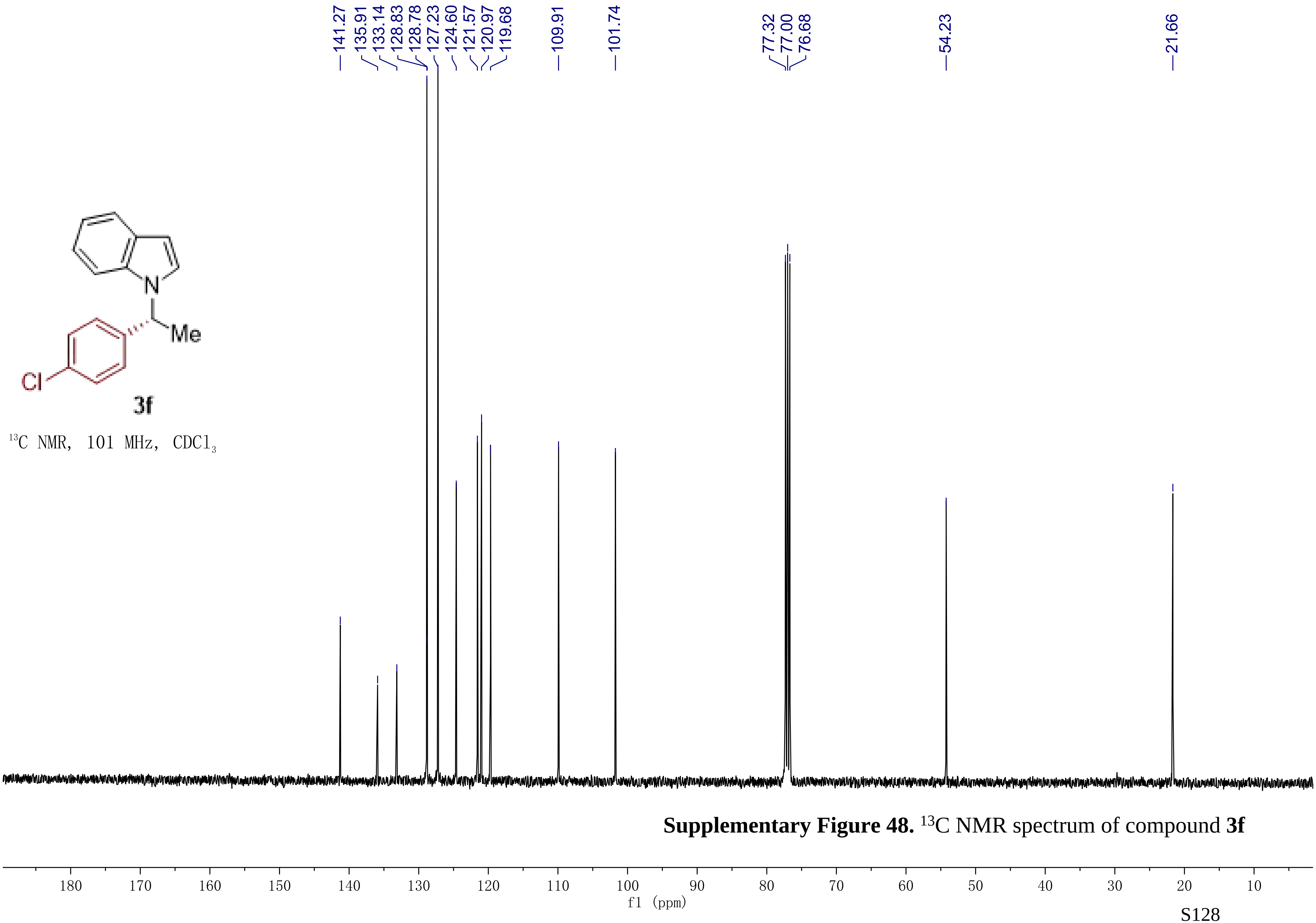

Supplementary Figure 48. ^{13}C NMR spectrum of compound **3f**

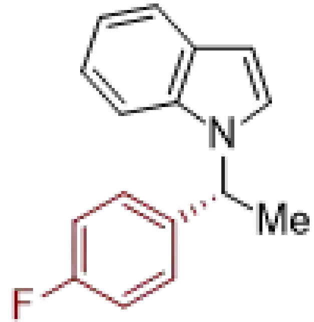

3g

1H NMR, 400 MHz, CDCl3

Supplementary Figure 49.
^{1}H NMR spectrum of compound **3g**

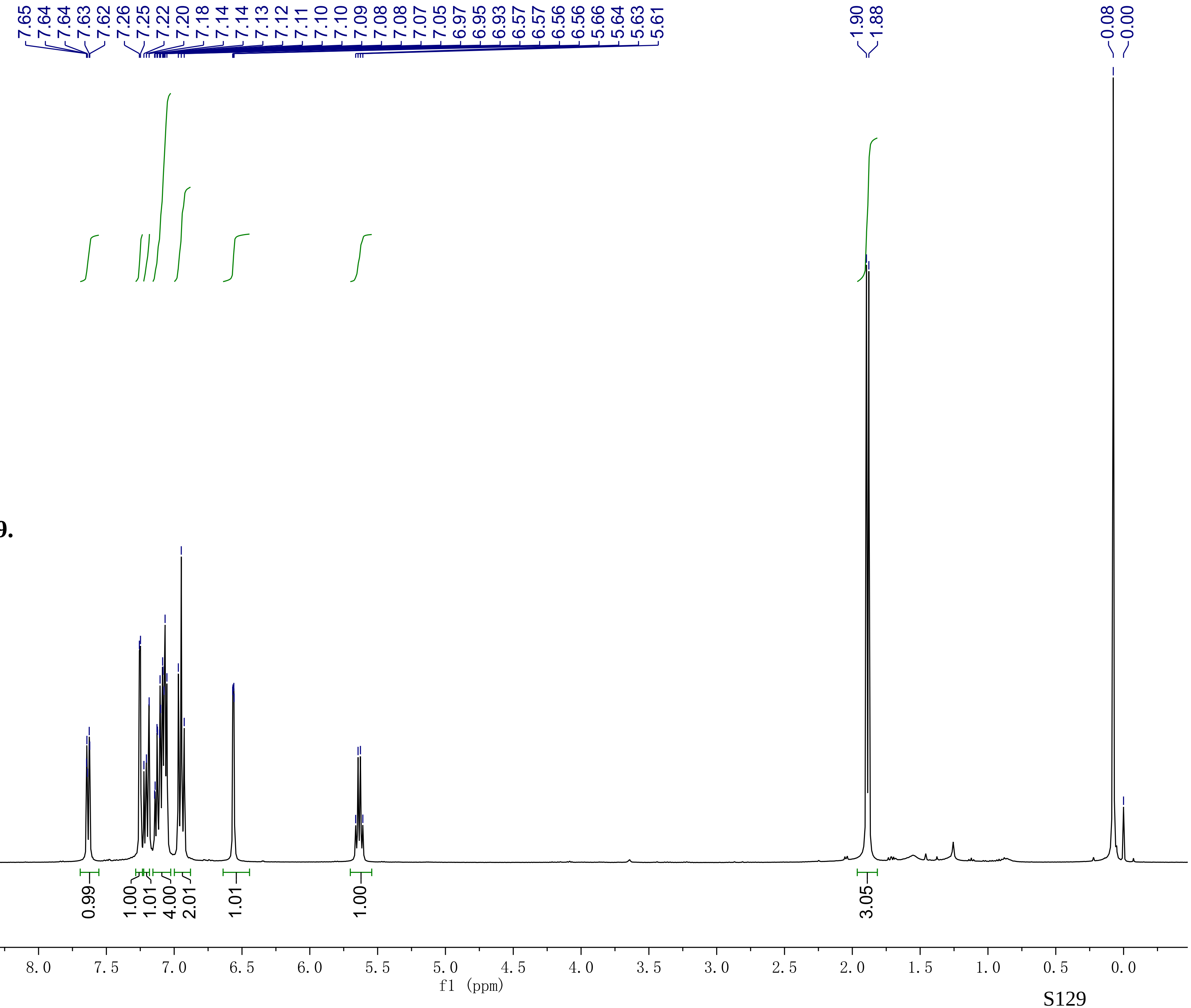

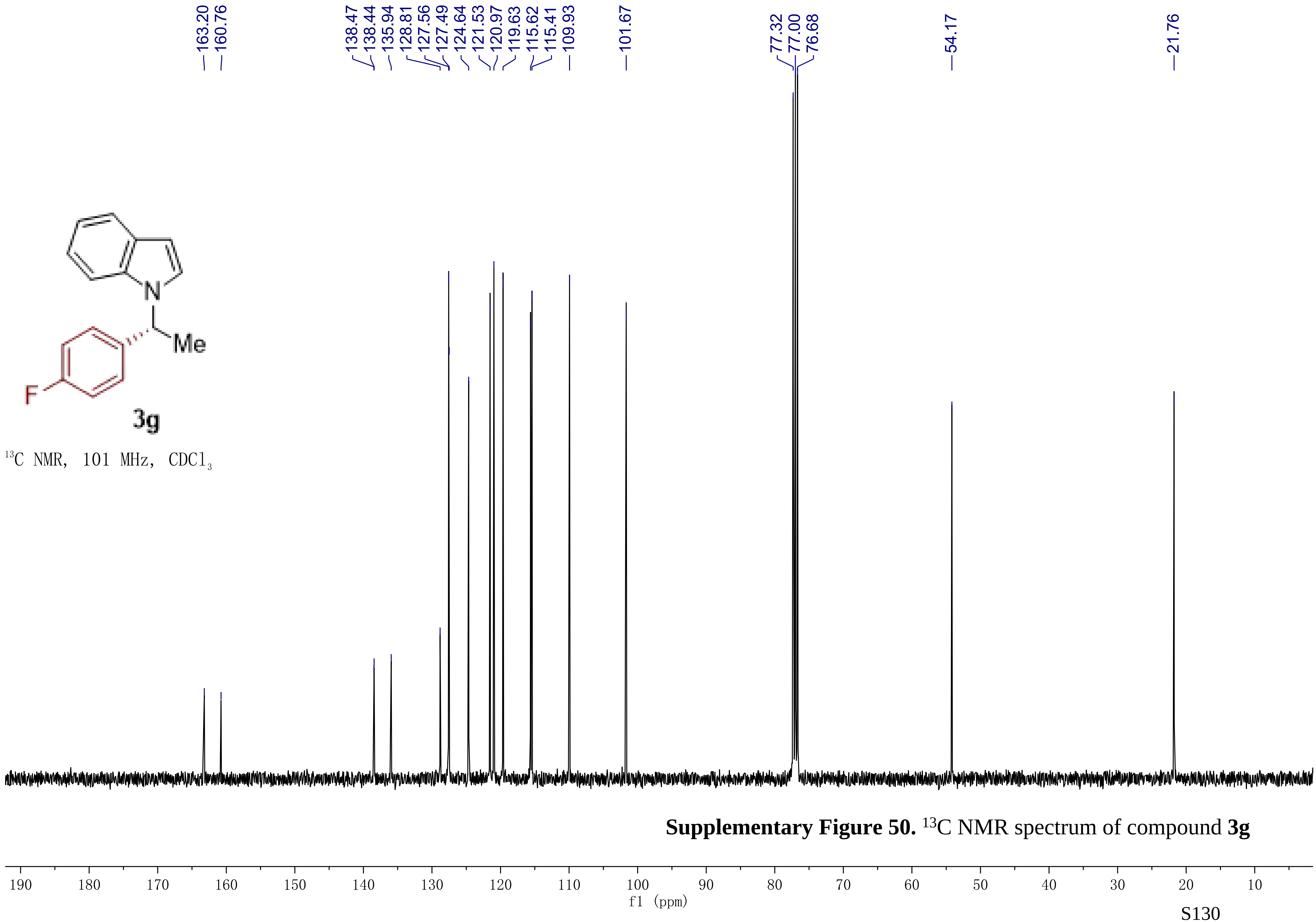

Supplementary Figure 50. ^{13}C NMR spectrum of compound **3g**

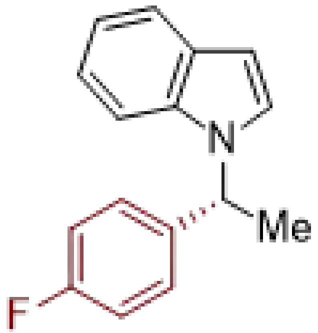

3g

^{19}F NMR, 376 MHz, $CDCl_3$

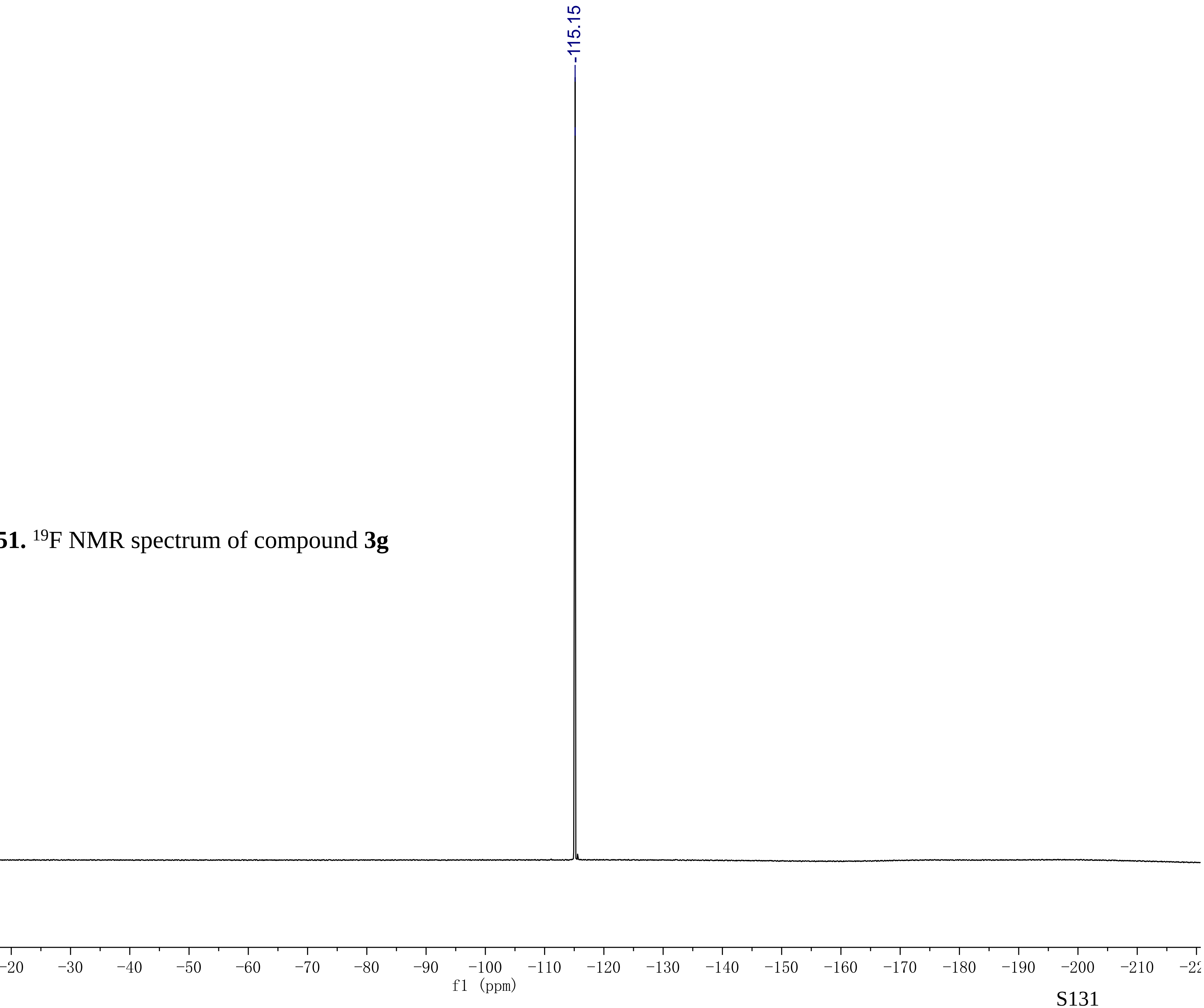

Supplementary Figure 51. ^{19}F NMR spectrum of compound **3g**

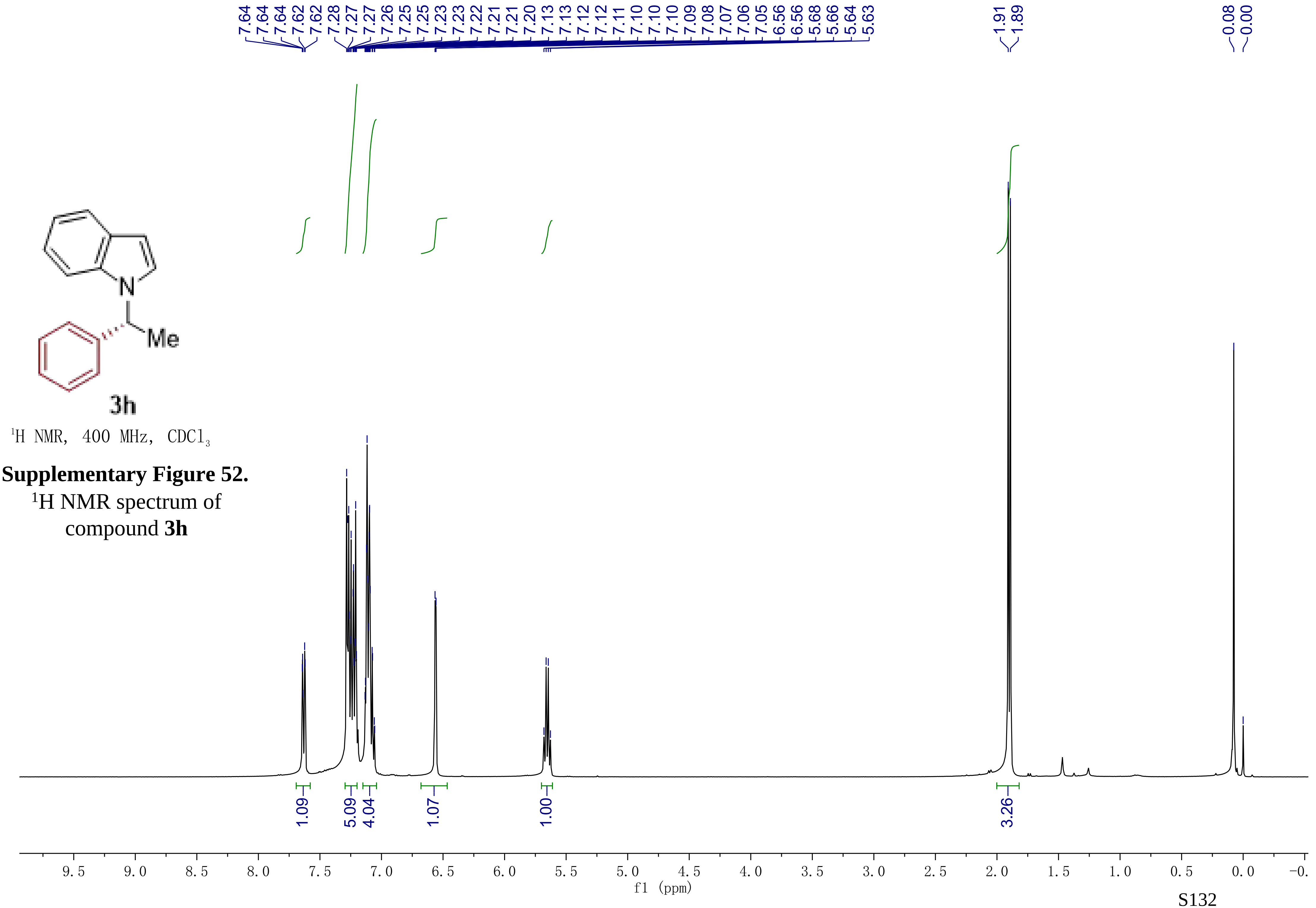

^{1}H NMR, 400 MHz, $CDCl_3$

Supplementary Figure 52. ^{1}H NMR spectrum of compound **3h**

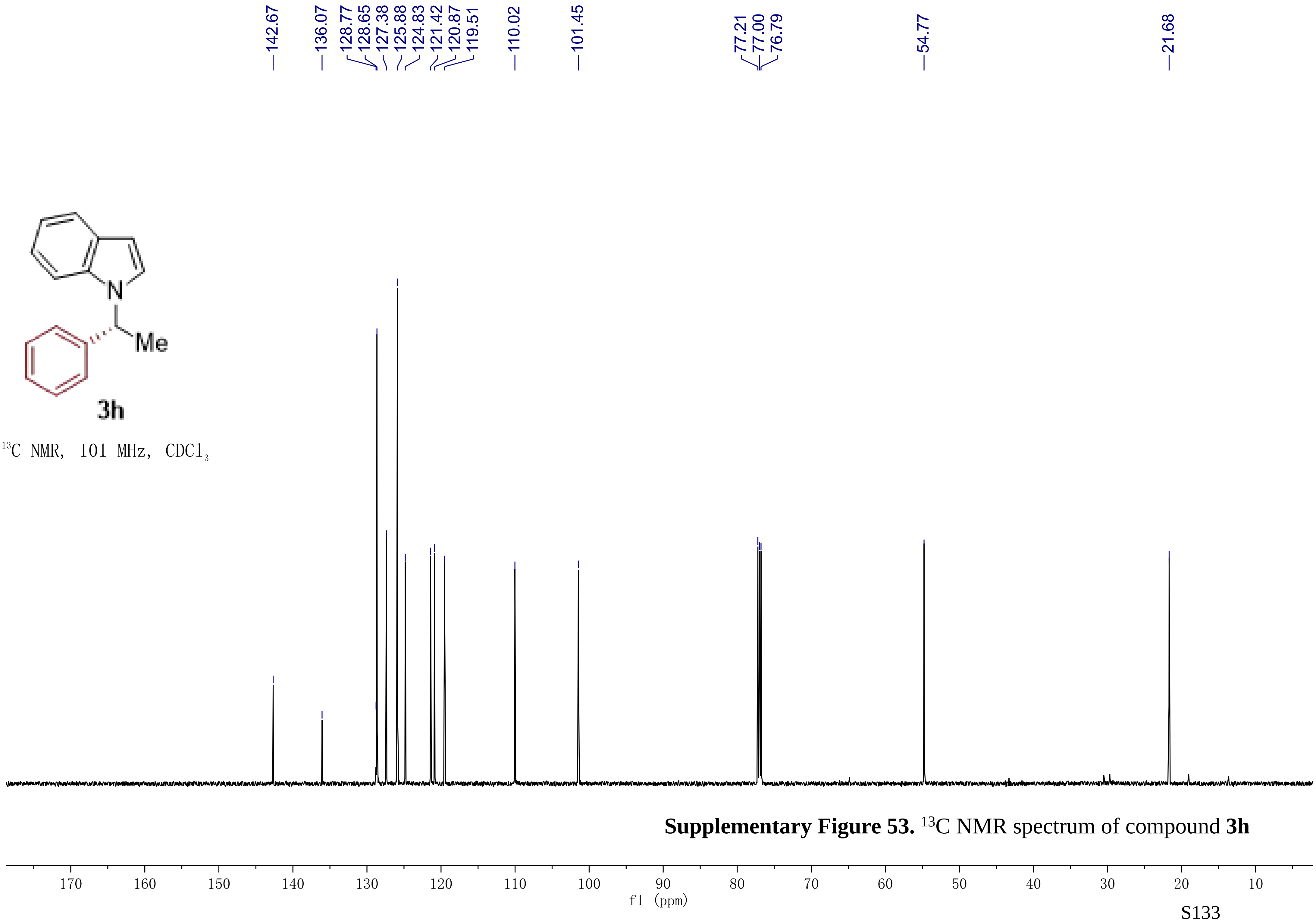

Supplementary Figure 53. ^{13}C NMR spectrum of compound **3h**

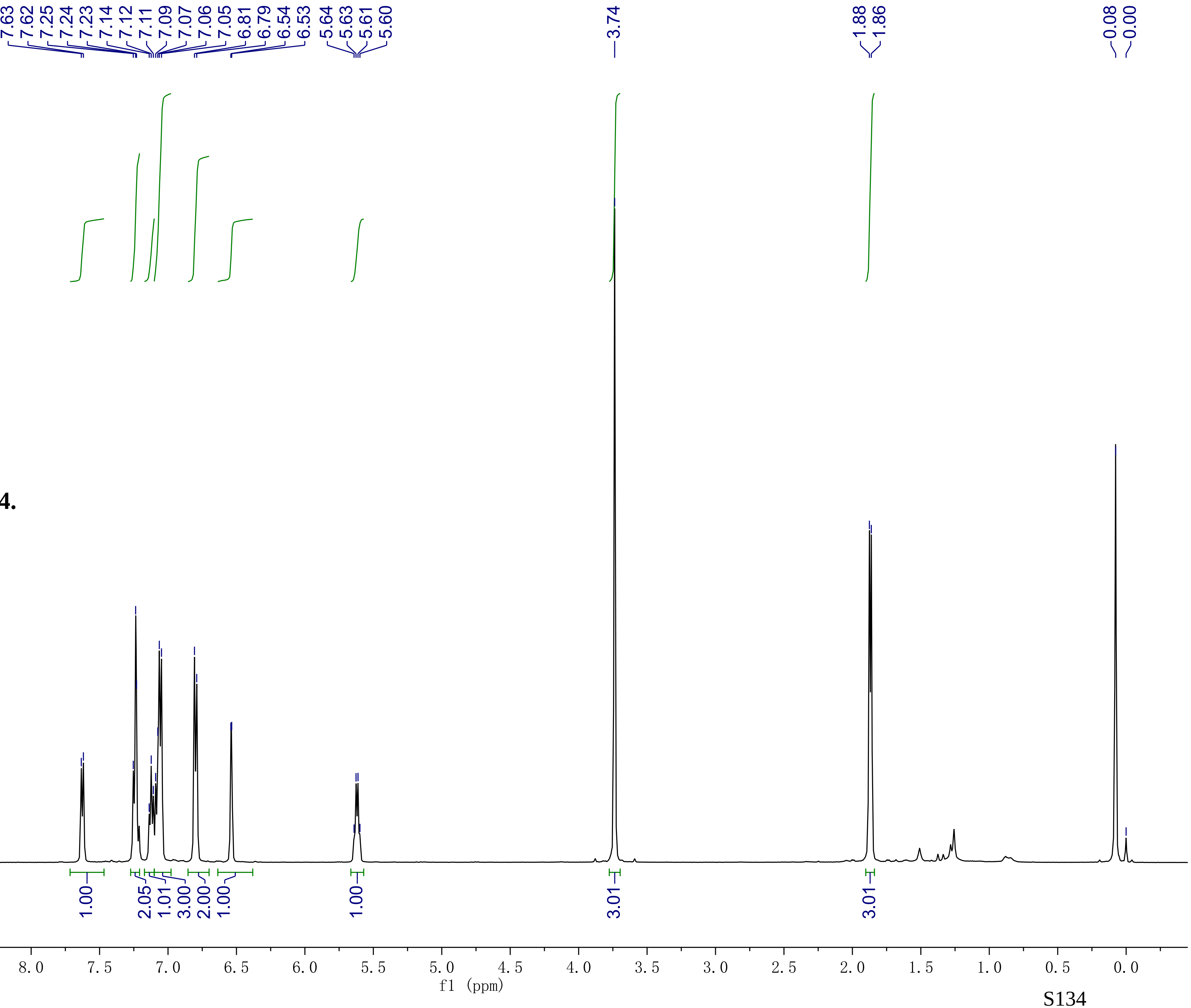

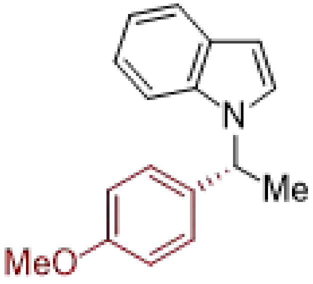

3i

^{1}H NMR, 500 MHz, $CDCl_3$

Supplementary Figure 54. ^{1}H NMR spectrum of compound **3i**

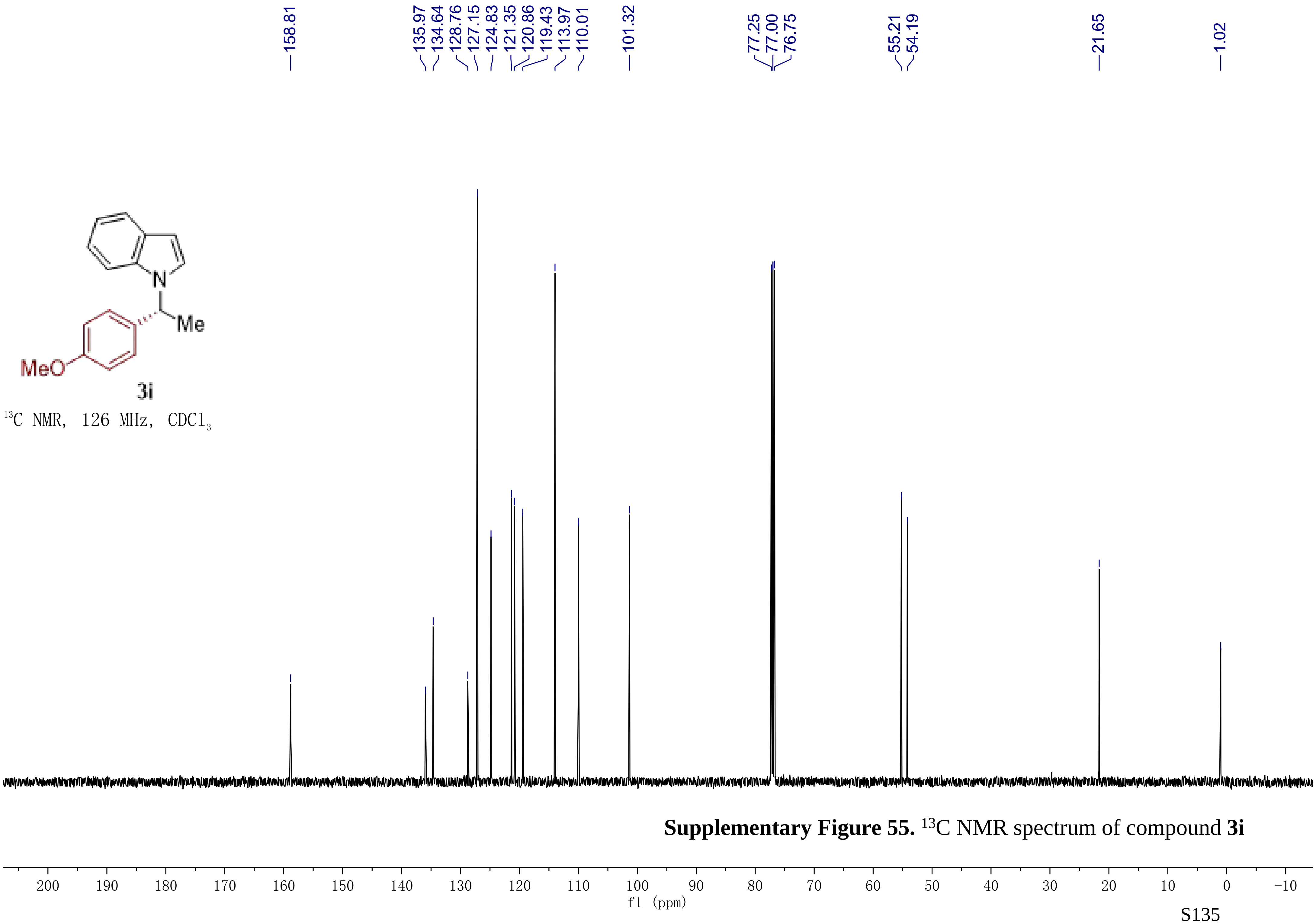

Supplementary Figure 55. ^{13}C NMR spectrum of compound **3i**

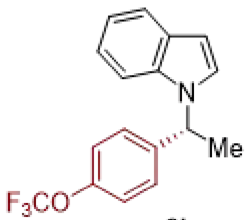

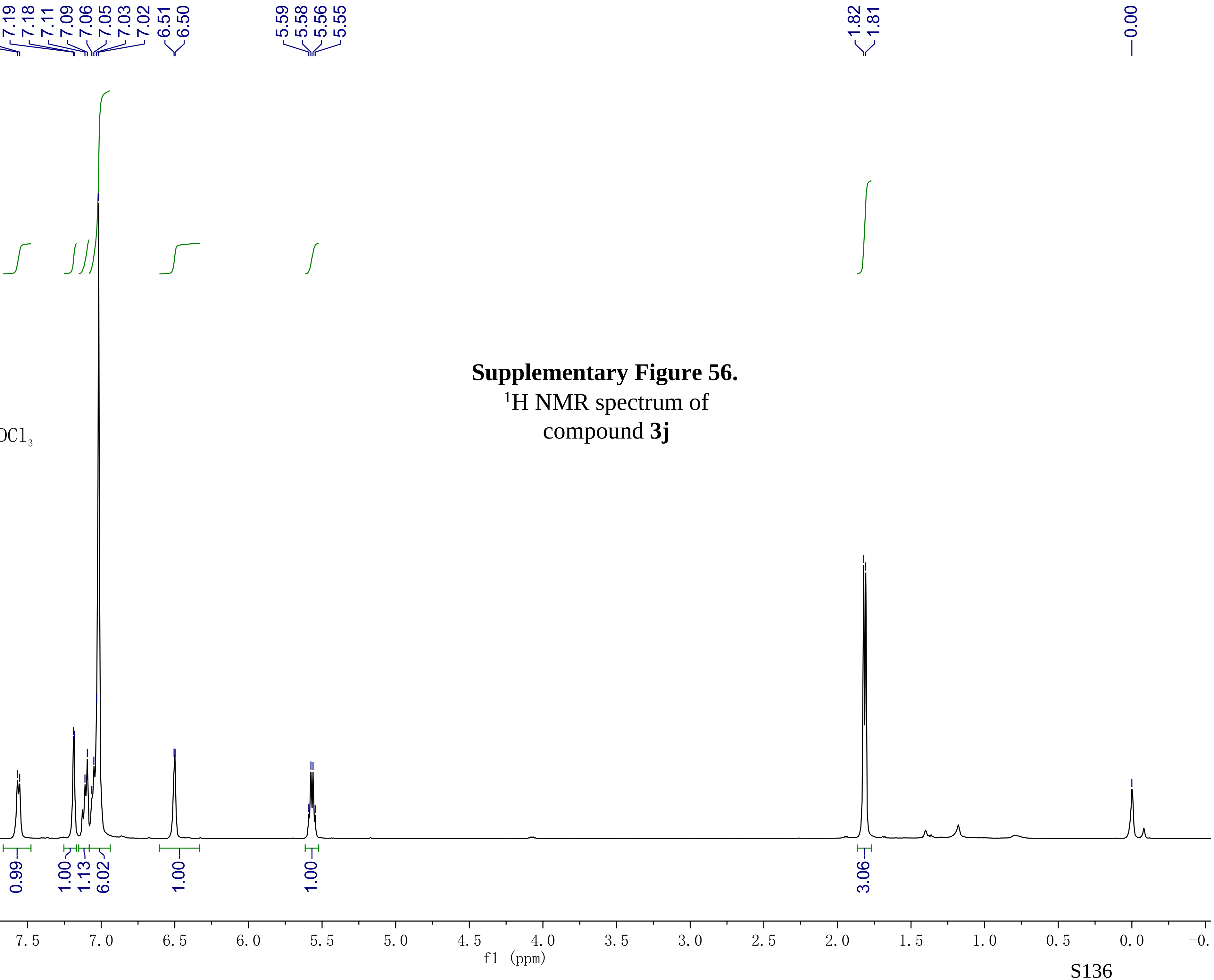

3j

^{1}H NMR, 500 MHz, $CDCl_3$

Supplementary Figure 56. ^{1}H NMR spectrum of compound **3j**

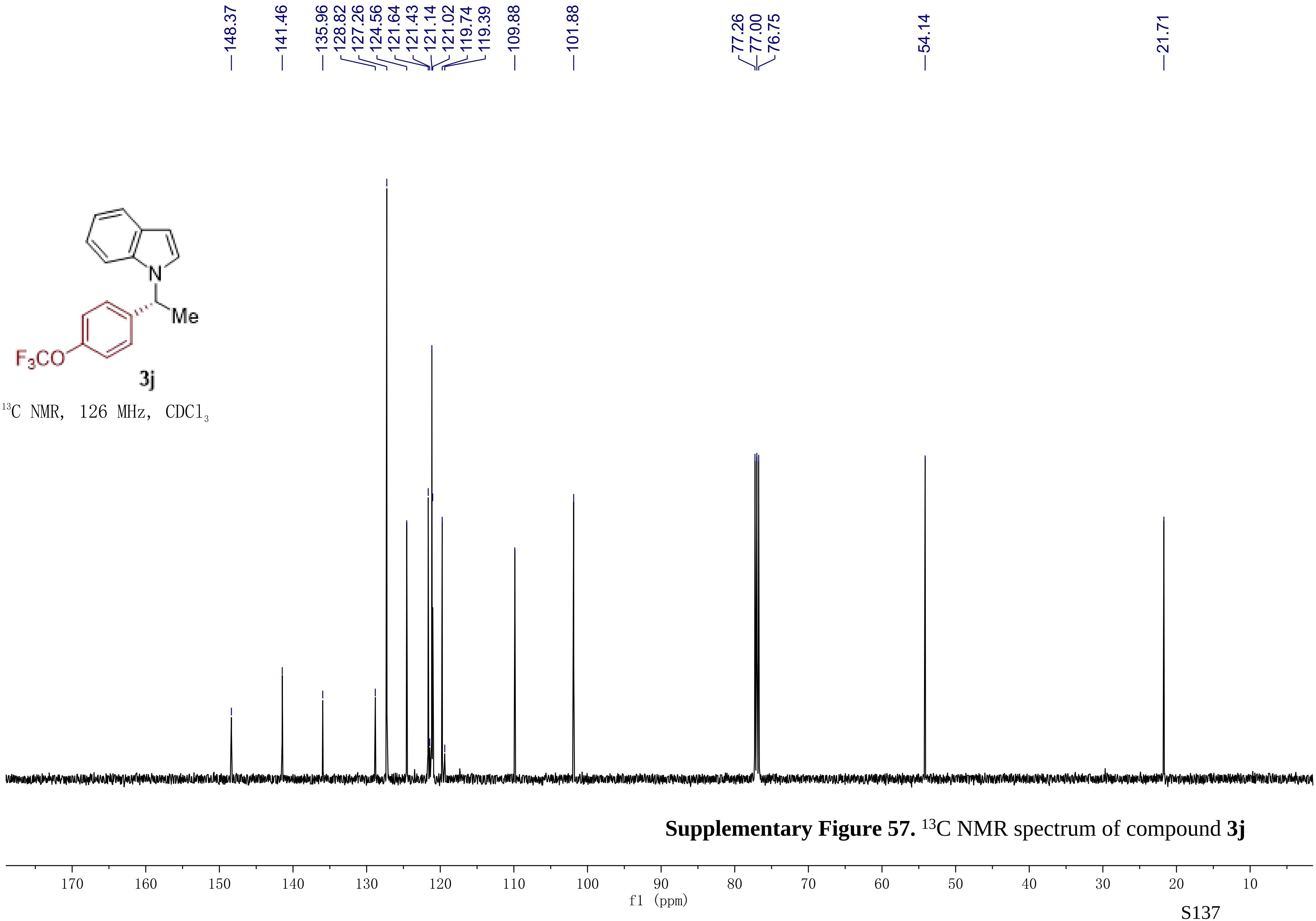

Supplementary Figure 57. ^{13}C NMR spectrum of compound **3j**

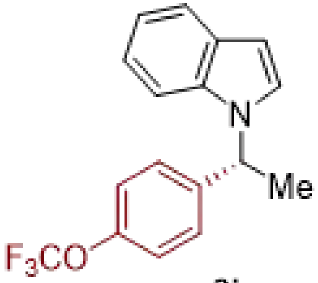

3j

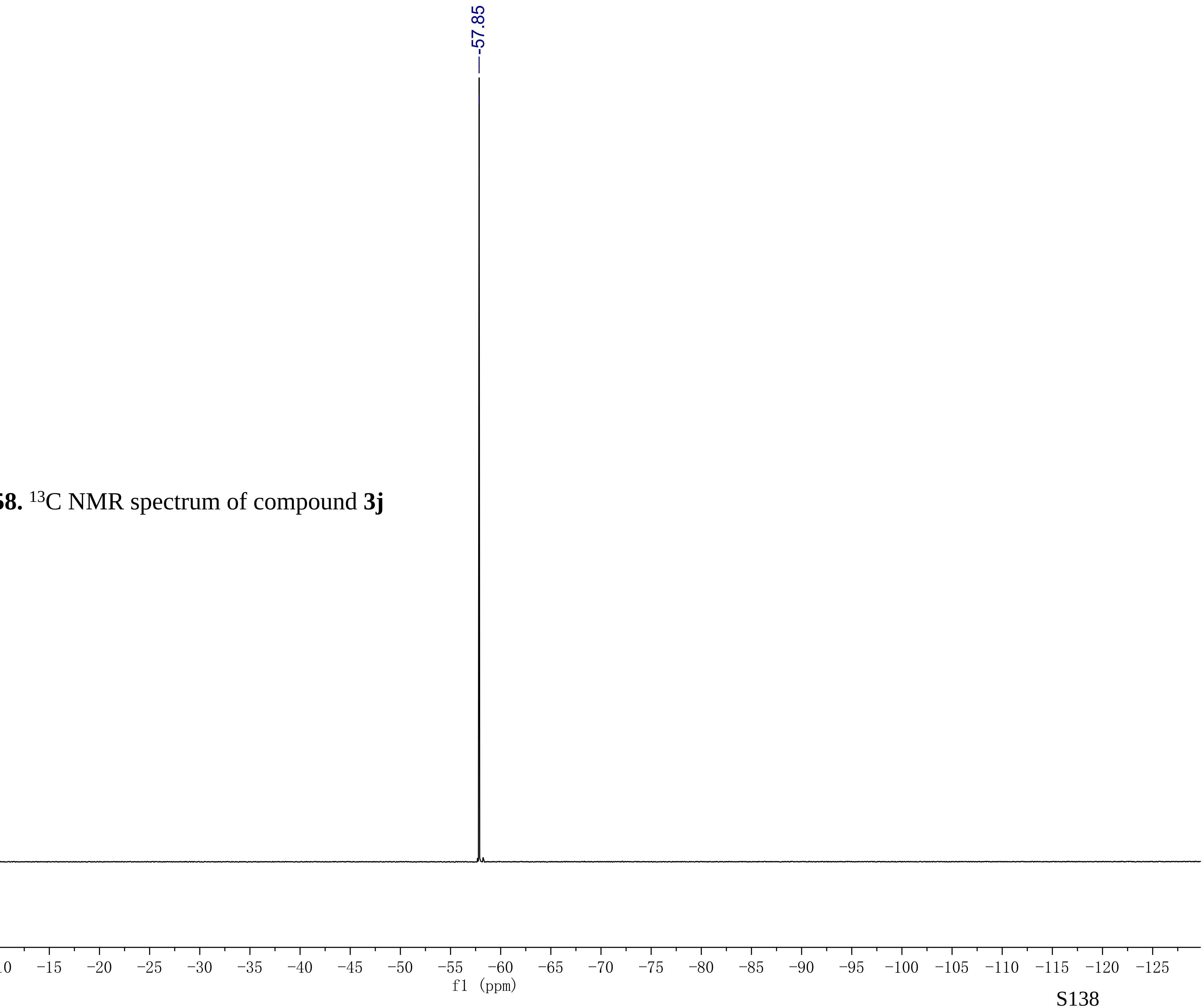

Supplementary Figure 58. ^{13}C NMR spectrum of compound **3j**

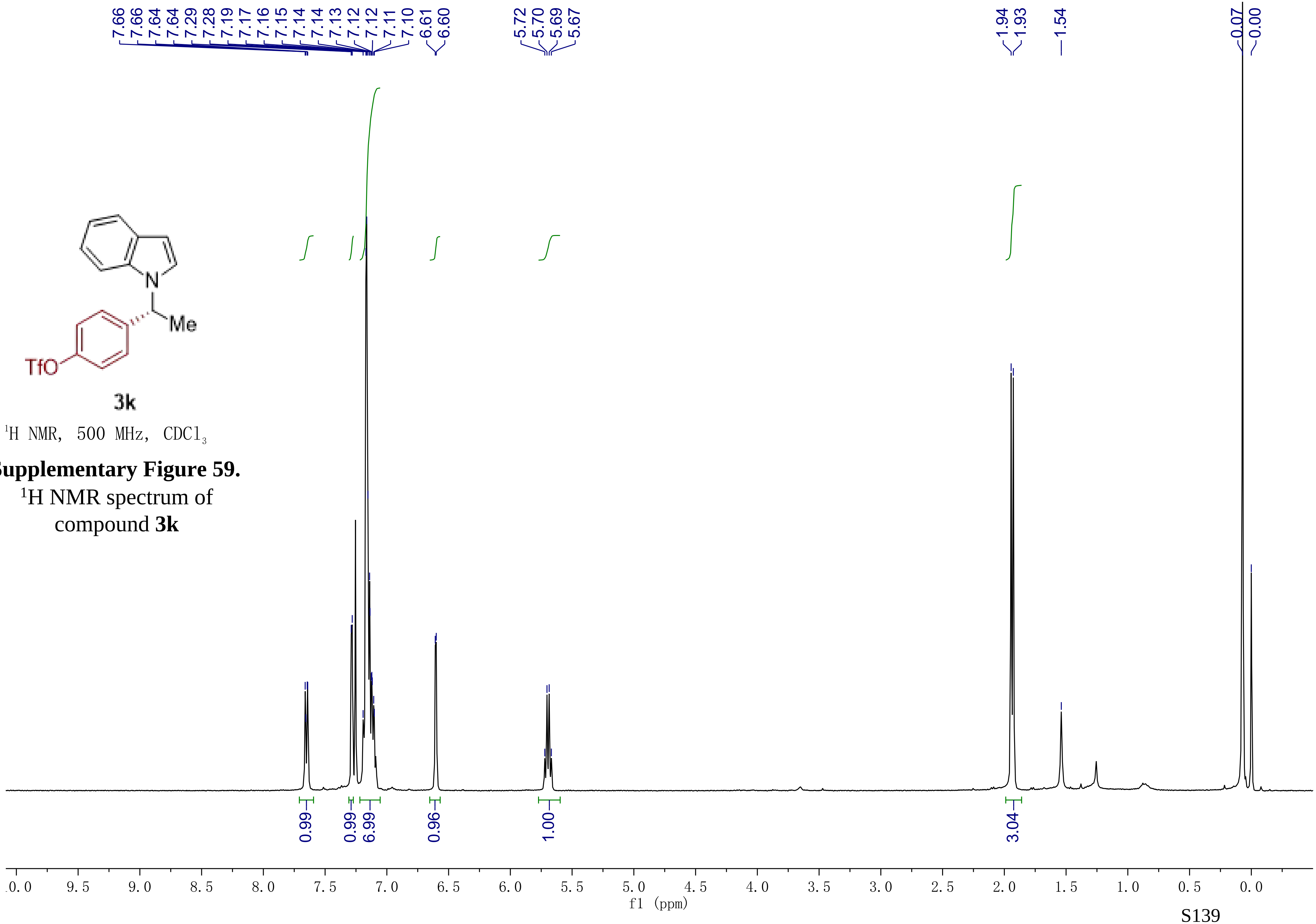

3k

^{1}H NMR, 500 MHz, $CDCl_3$

Supplementary Figure 59. ^{1}H NMR spectrum of compound **3k**

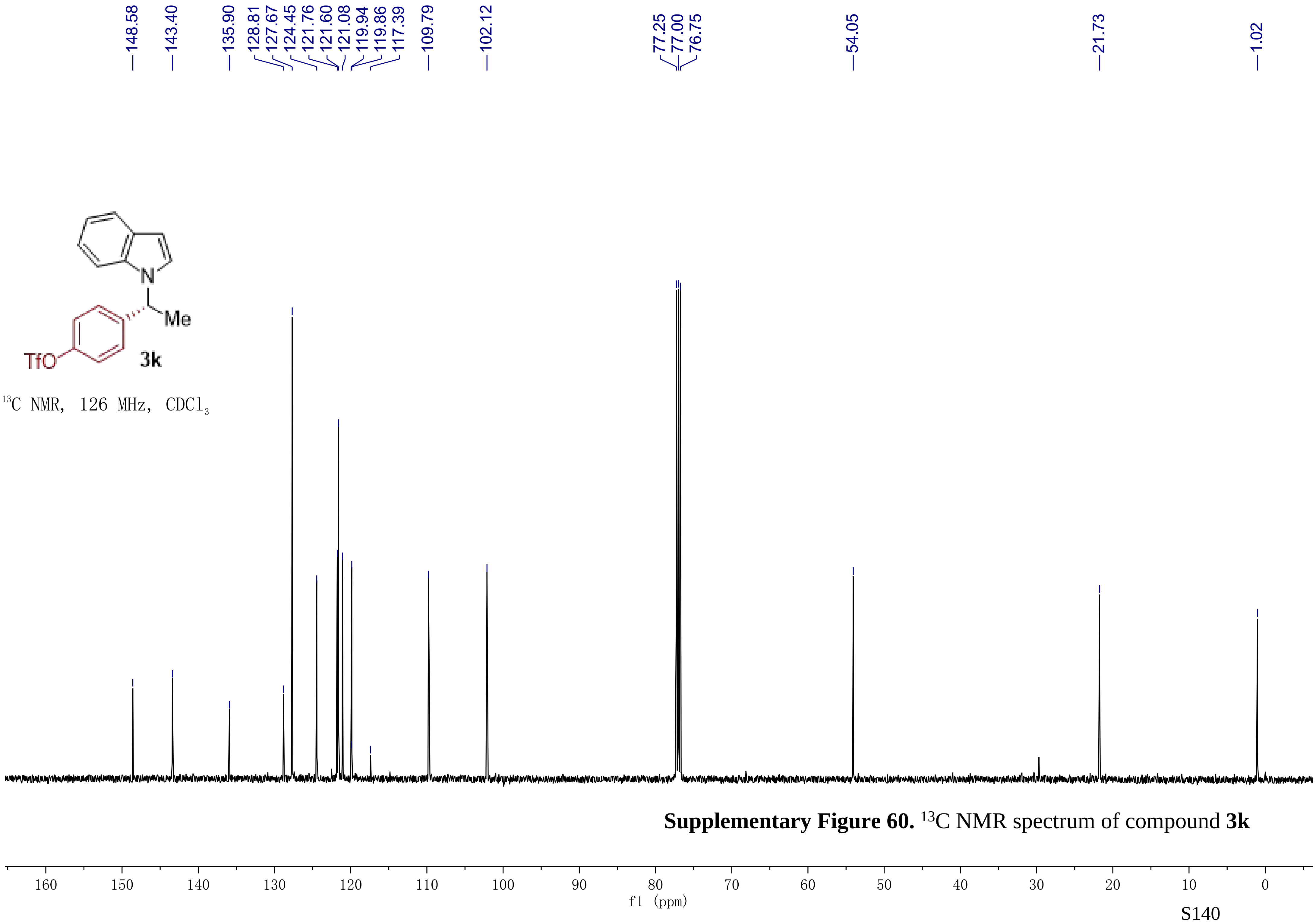

Supplementary Figure 60. ^{13}C NMR spectrum of compound **3k**

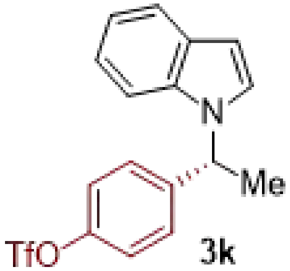

3k

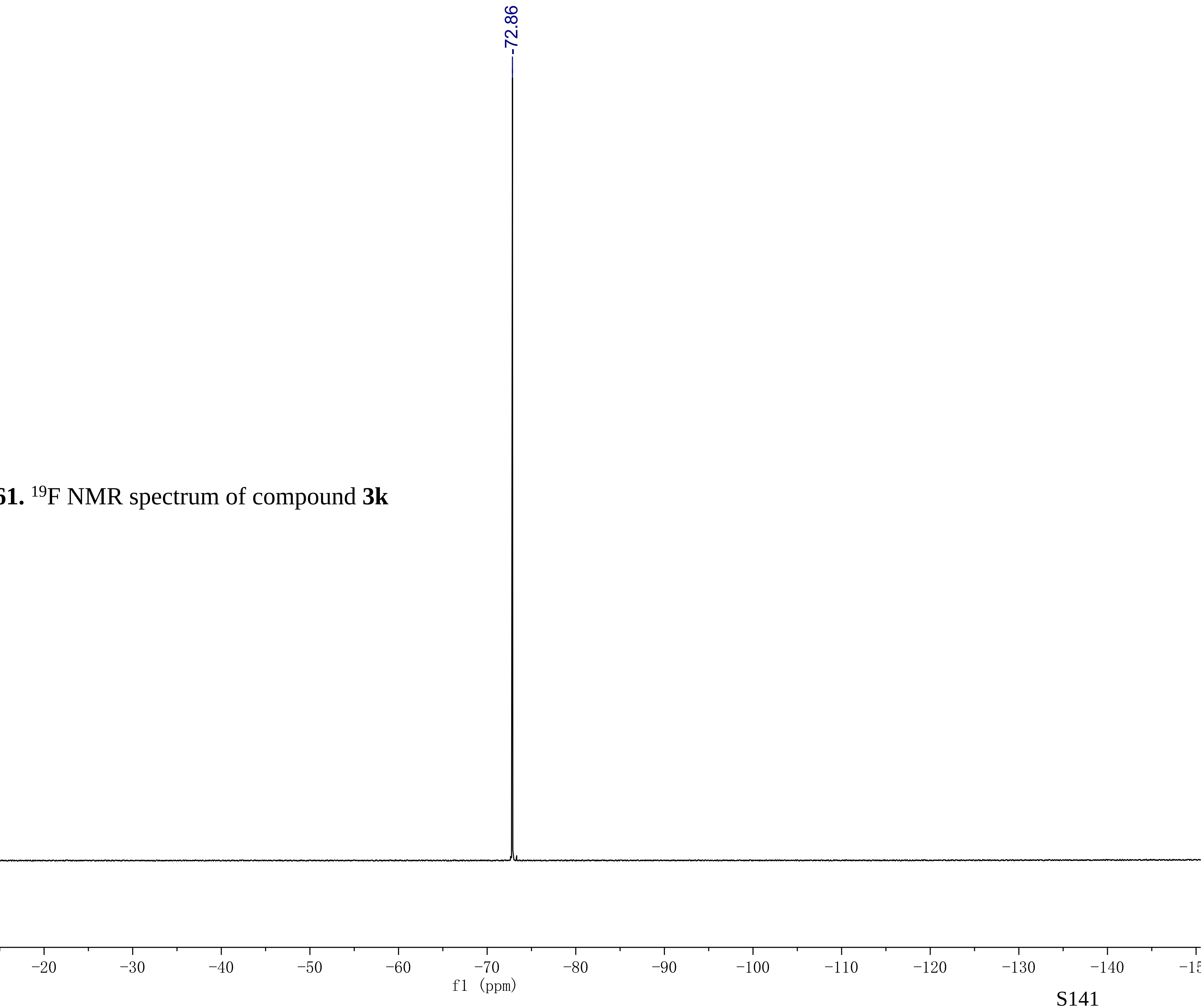

Supplementary Figure 61. ^{19}F NMR spectrum of compound **3k**

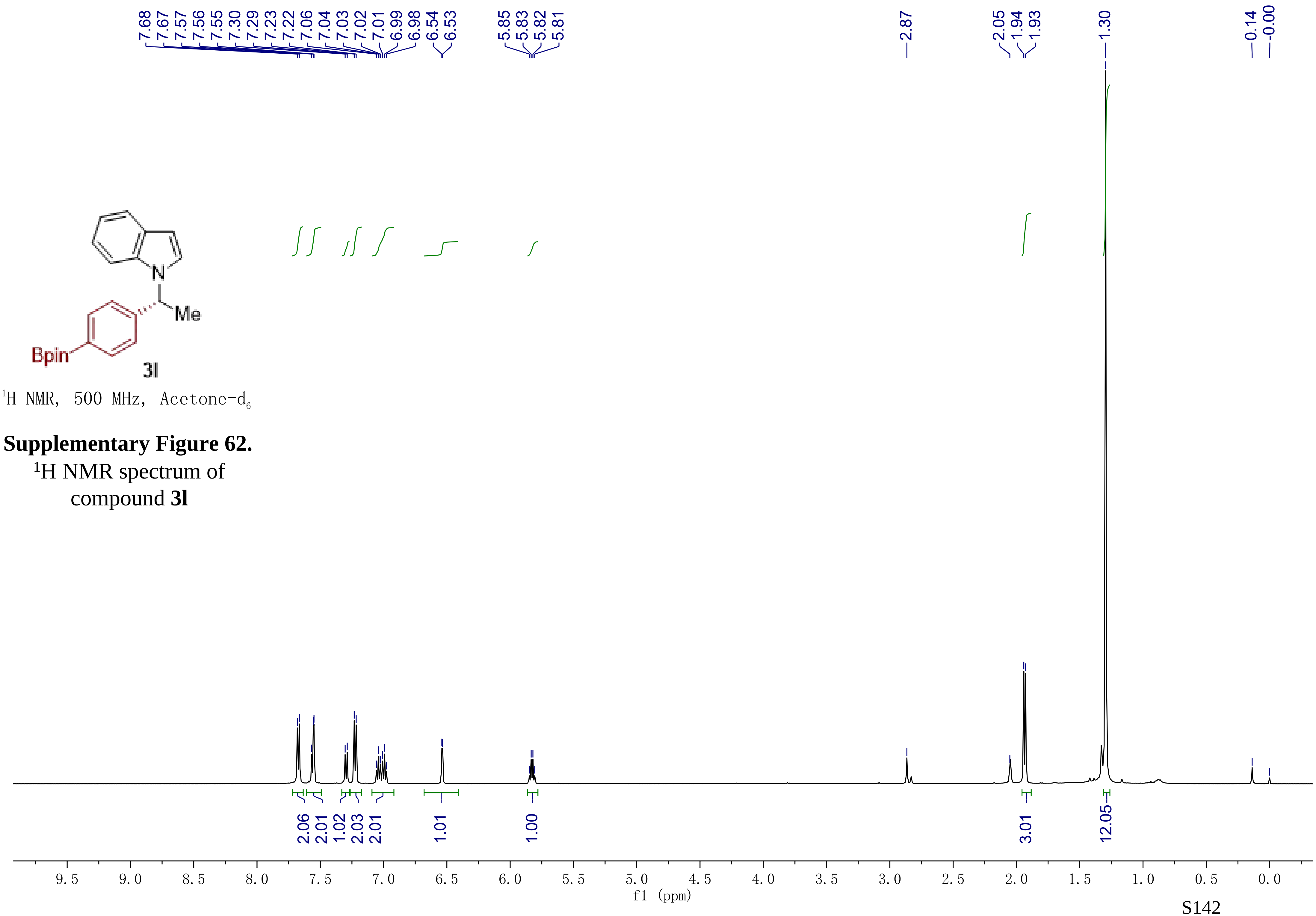

^{1}H NMR, 500 MHz, Acetone-d_6

Supplementary Figure 62. ^{1}H NMR spectrum of compound **3l**

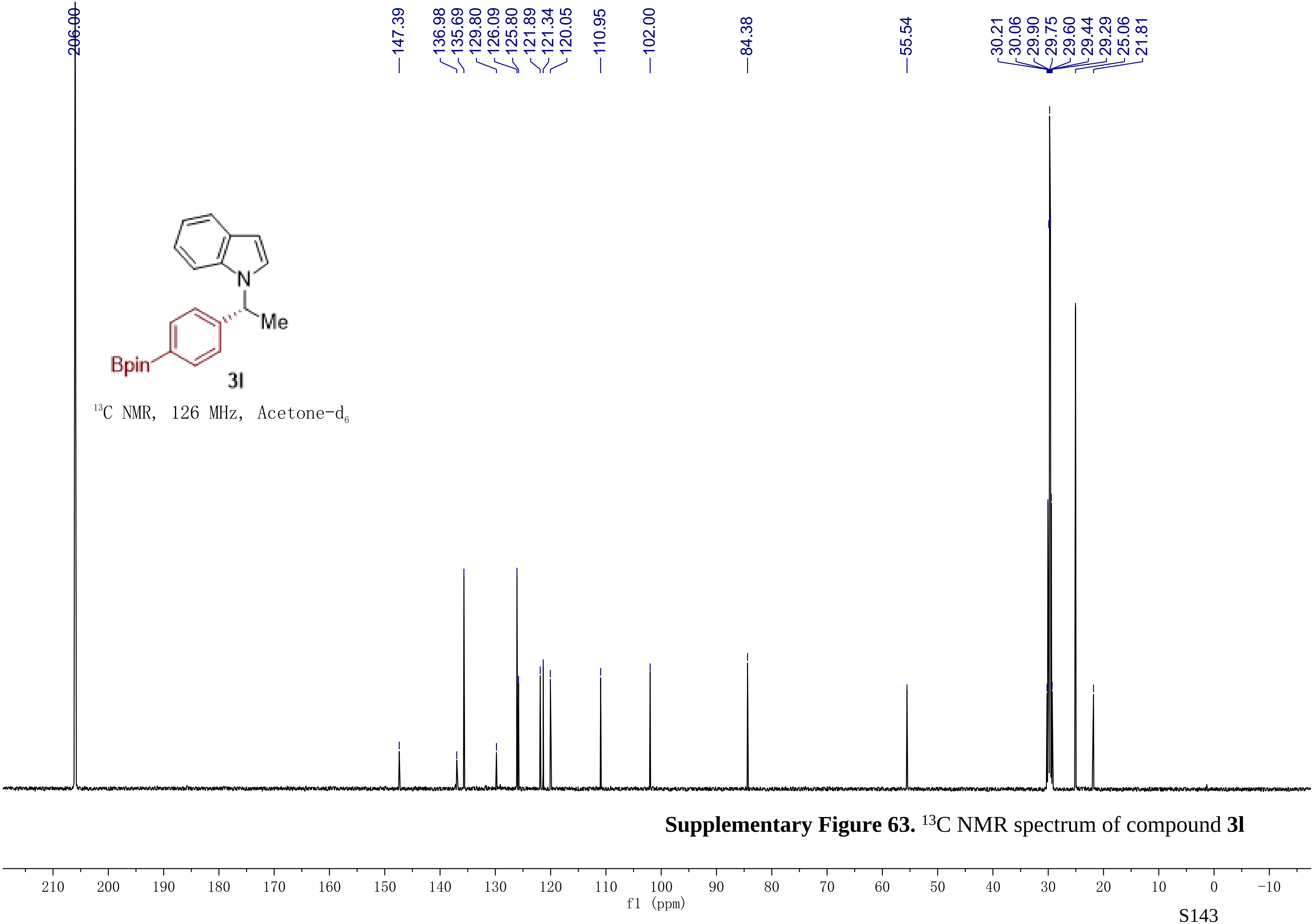

Supplementary Figure 63. ^{13}C NMR spectrum of compound **3l**

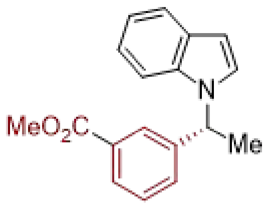

3m

^{1}H NMR, 500 MHz, $CDCl_3$

Supplementary Figure 64. ^{1}H NMR spectrum of compound **3m**

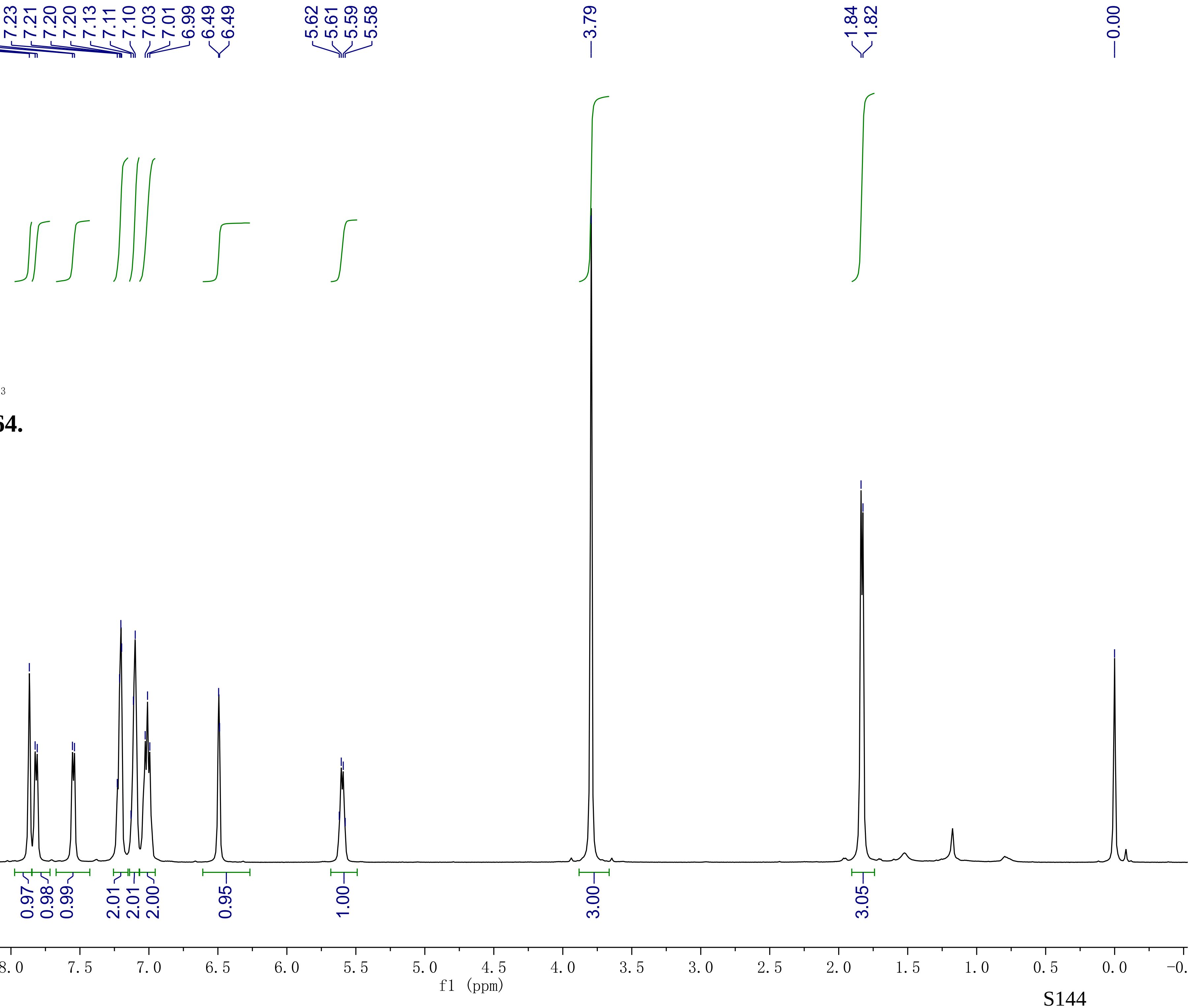

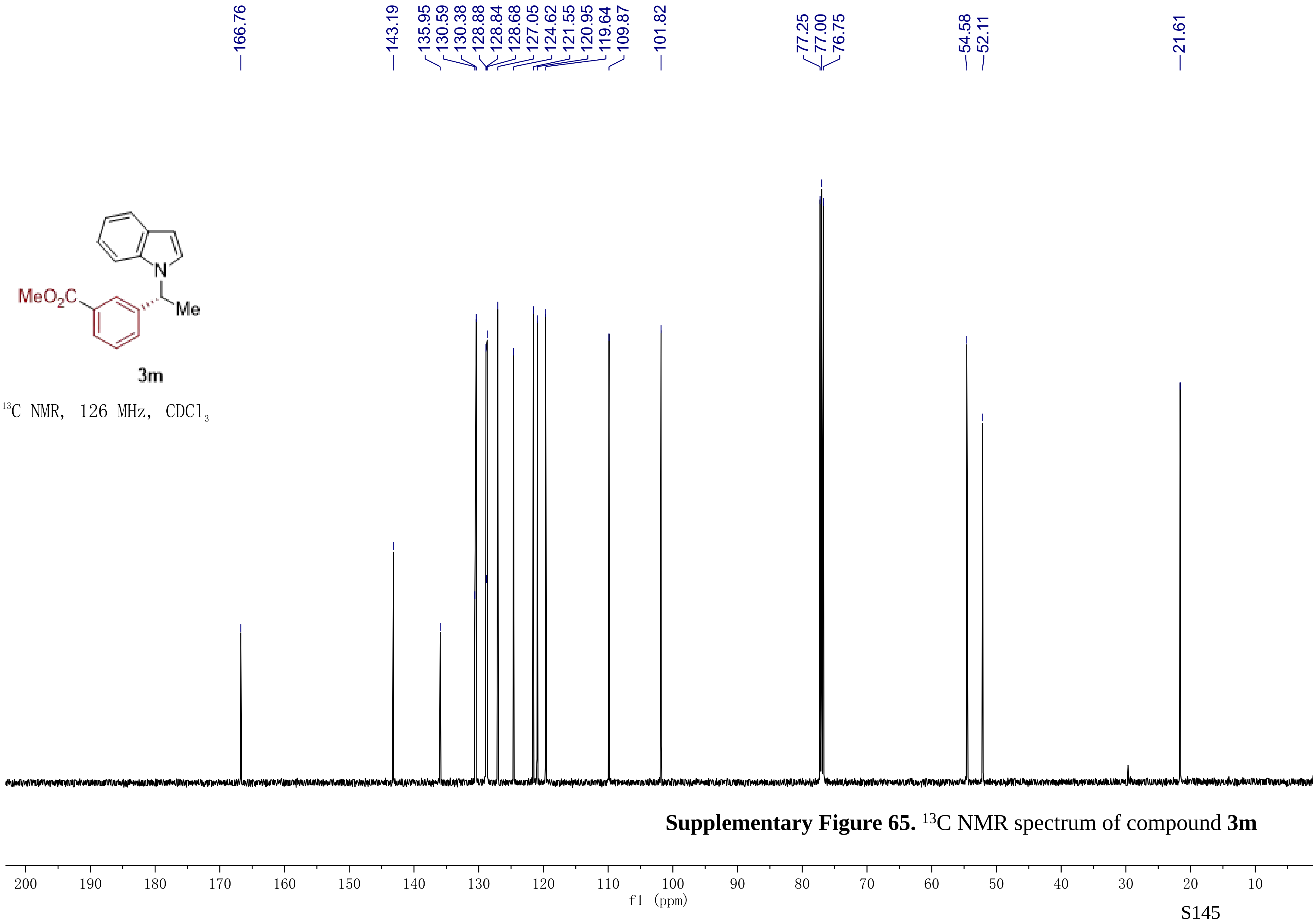

Supplementary Figure 65. ^{13}C NMR spectrum of compound **3m**

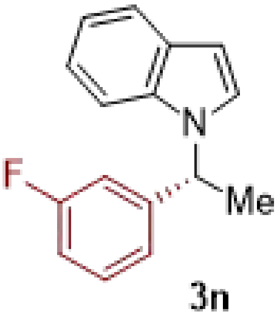

3n

^{1}H NMR, 500 MHz, $CDCl_3$

Supplementary Figure 66. ^{1}H NMR spectrum of compound **3n**

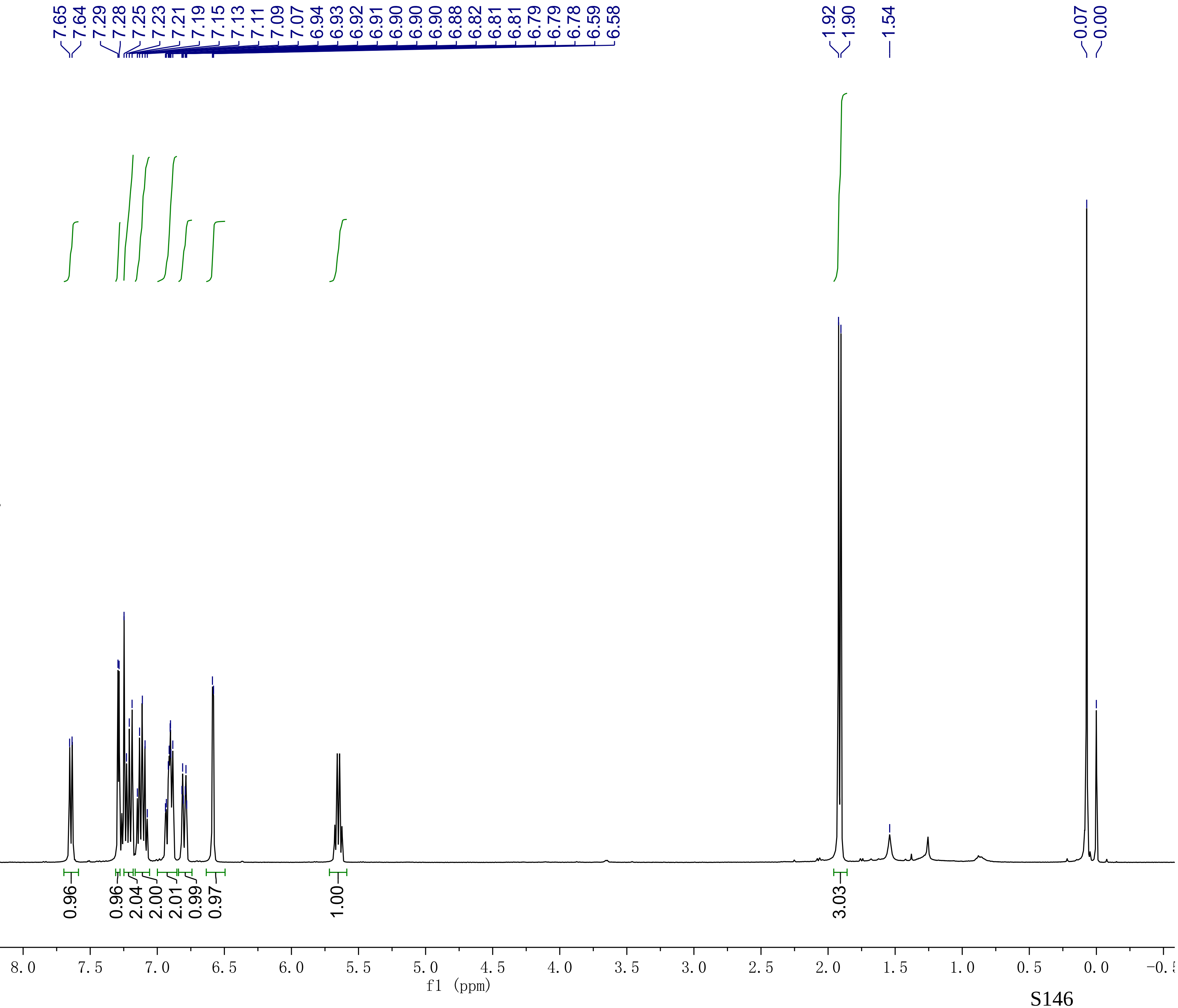

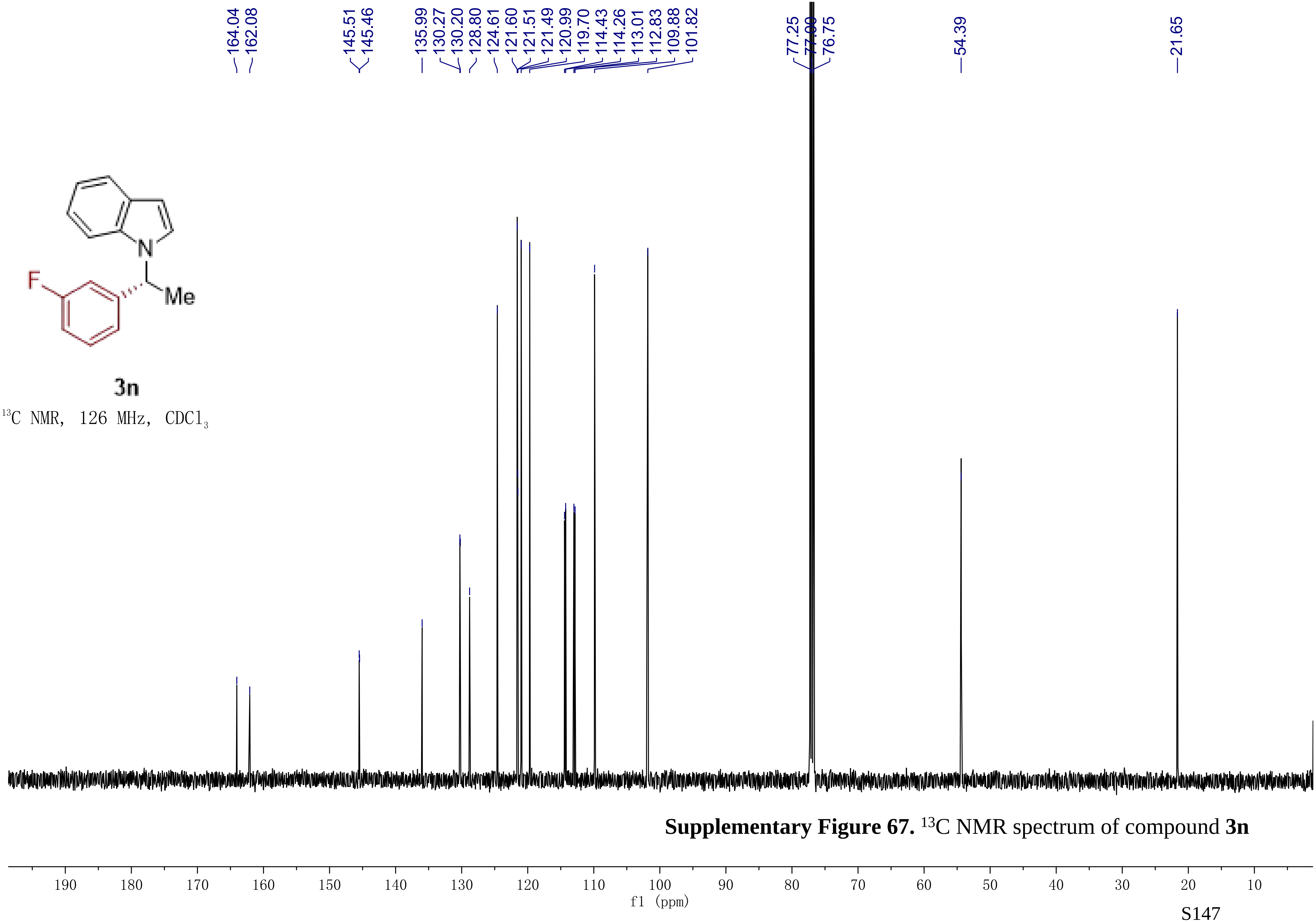

Supplementary Figure 67. ^{13}C NMR spectrum of compound **3n**

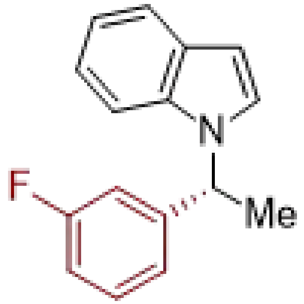

3n

^{19}F NMR, 471 MHz, $CDCl_3$

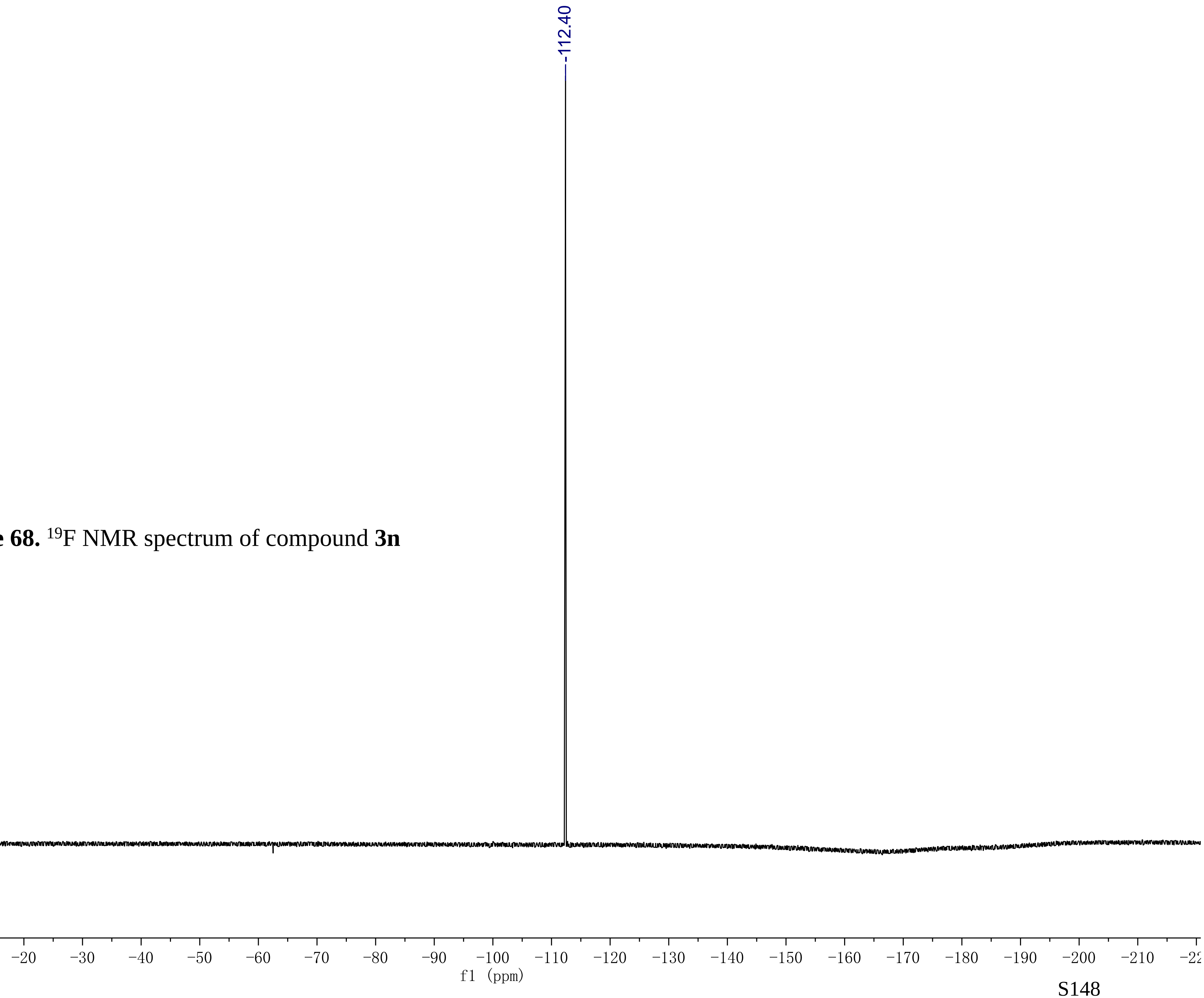

Supplementary Figure 68. ^{19}F NMR spectrum of compound **3n**

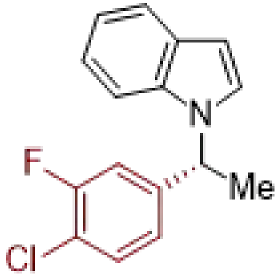

3o

^{1}H NMR, 500 MHz, $CDCl_3$

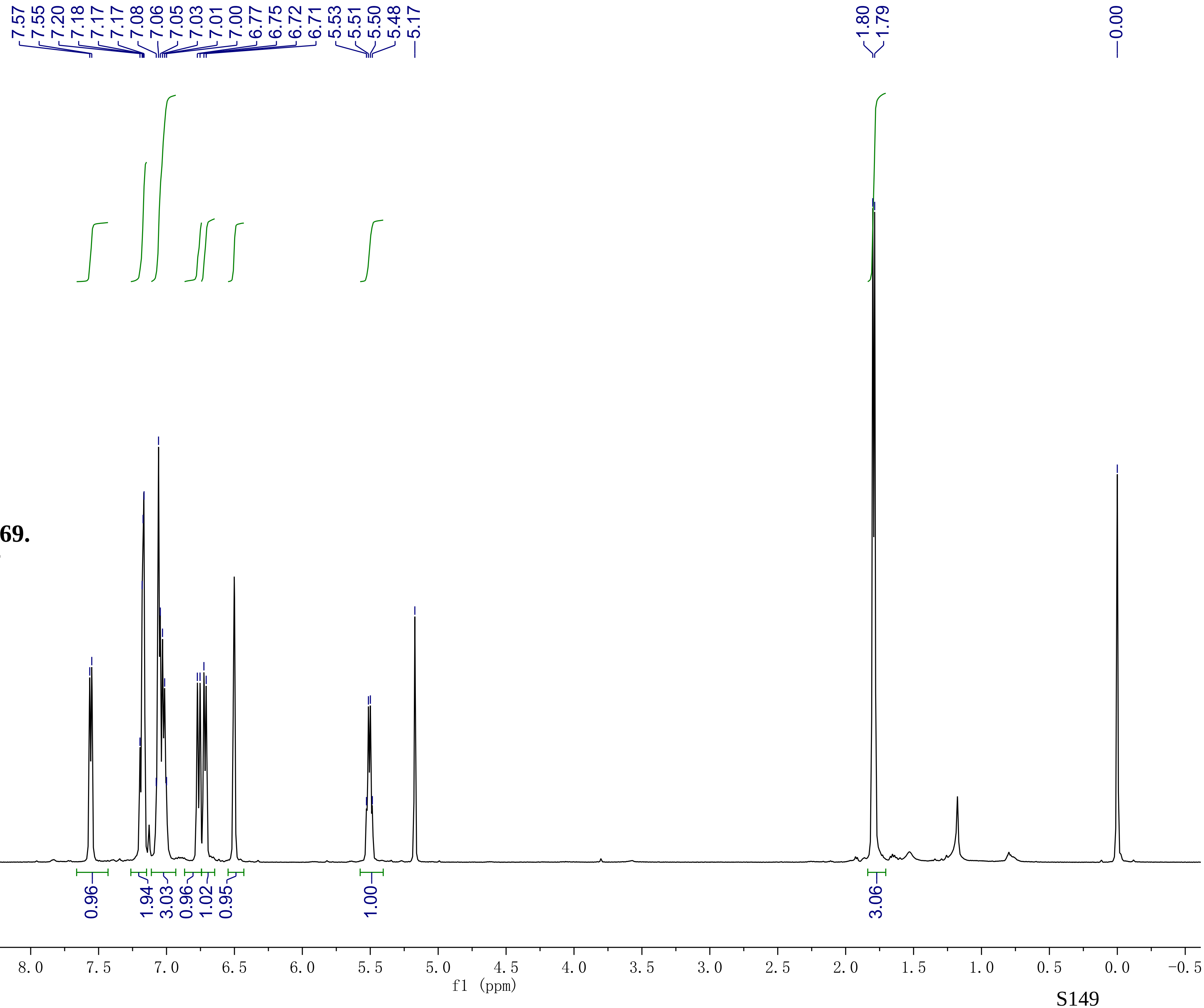

Supplementary Figure 69. ^{1}H NMR spectrum of compound **3o**

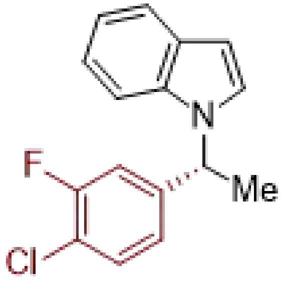

3o

^{13}C NMR, 126 MHz, $CDCl_3$

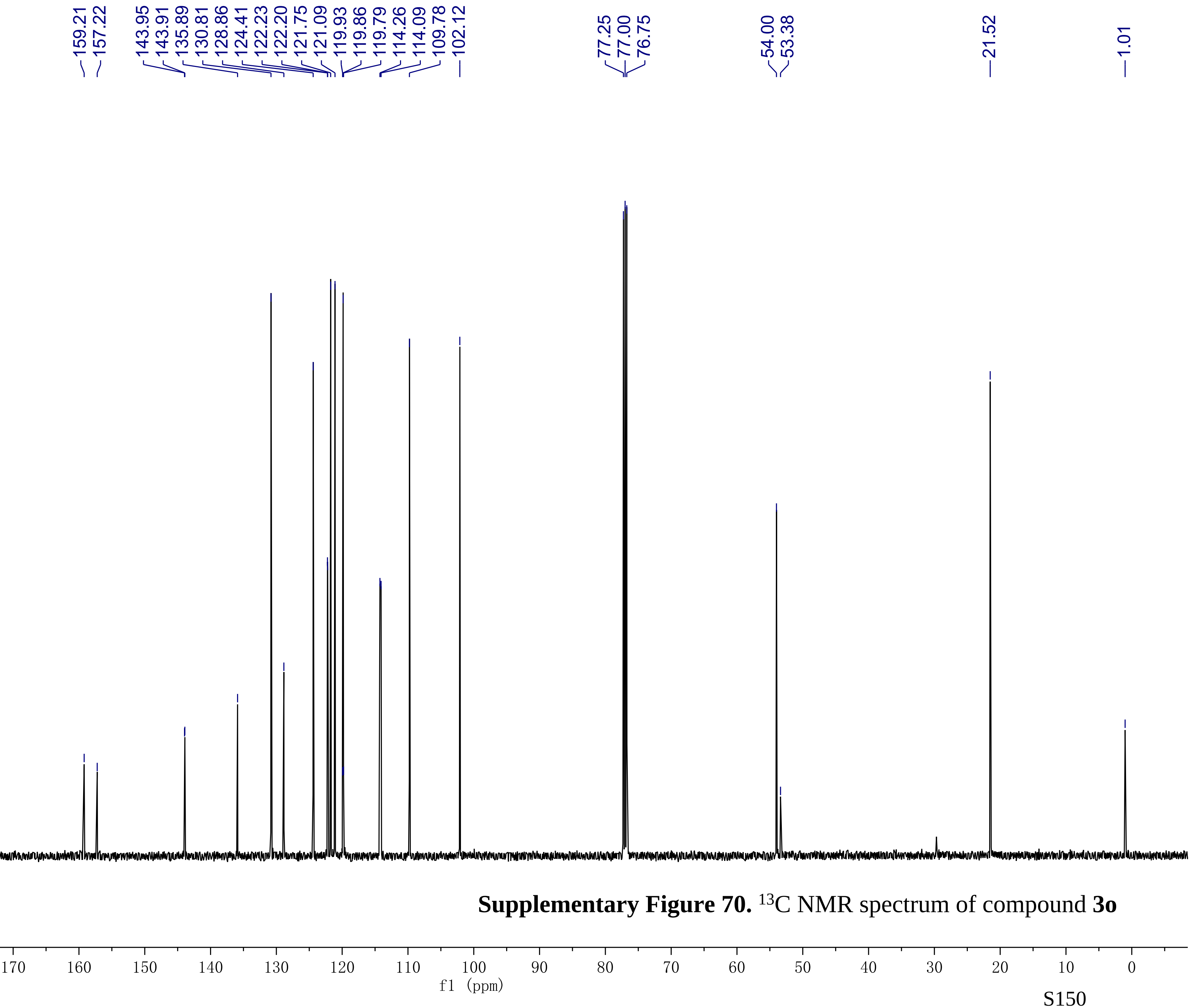

Supplementary Figure 70. ^{13}C NMR spectrum of compound **3o**

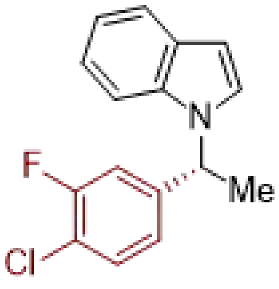

3o

^{19}F NMR, 471 MHz, $CDCl_3$

Supplementary Figure 71. ^{19}F NMR spectrum of compound **3o**

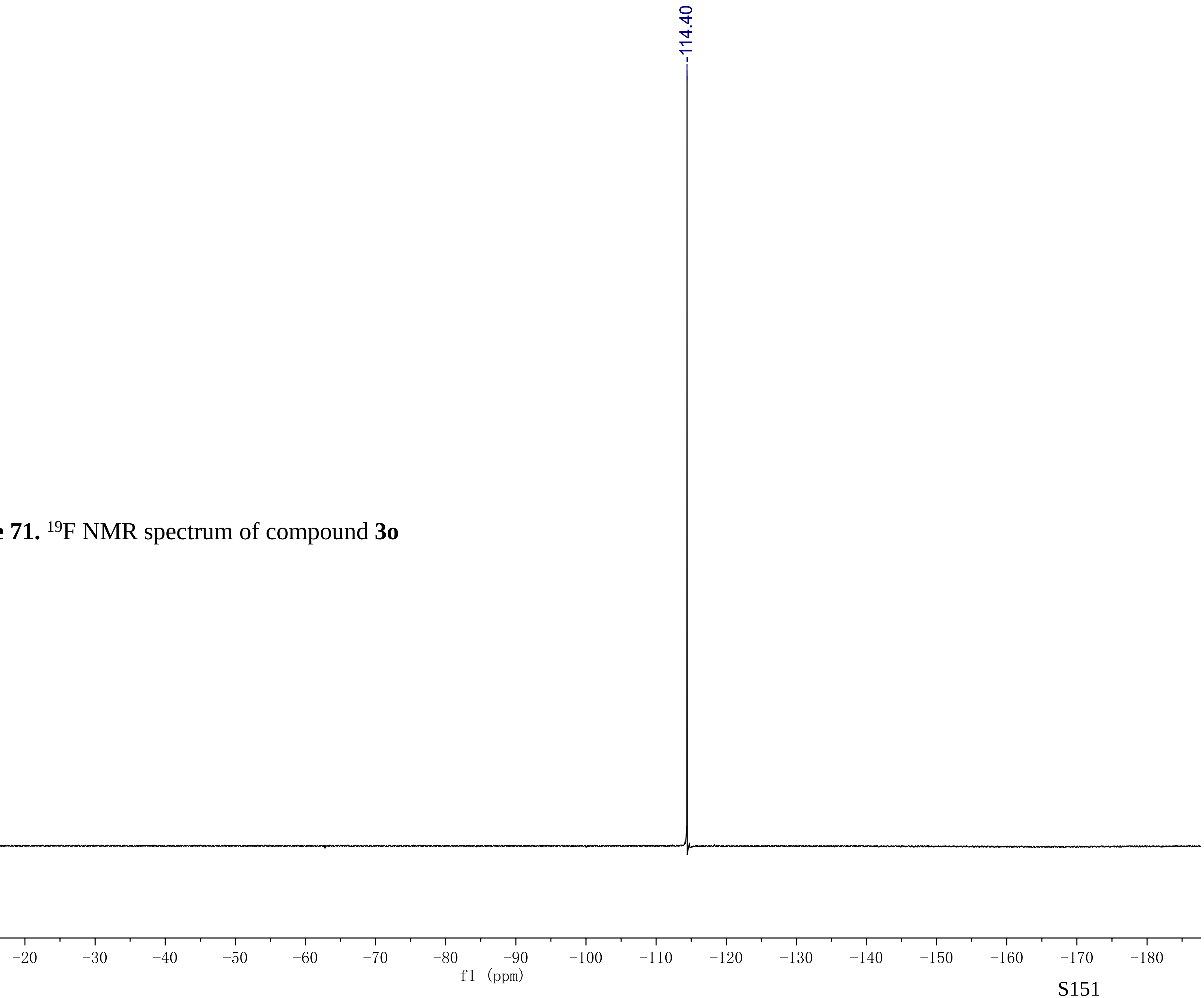

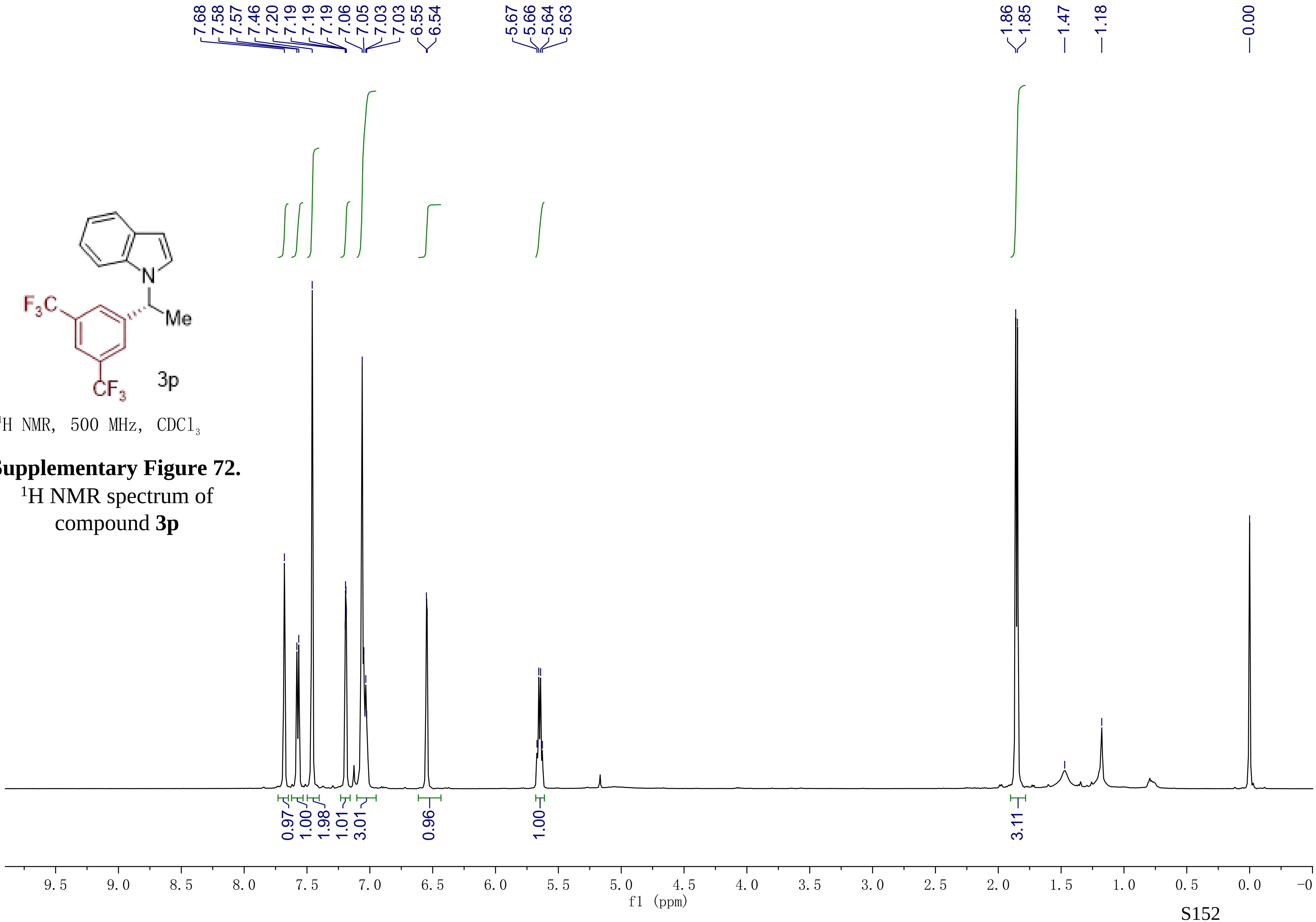

Supplementary Figure 72.
^{1}H NMR spectrum of compound **3p**

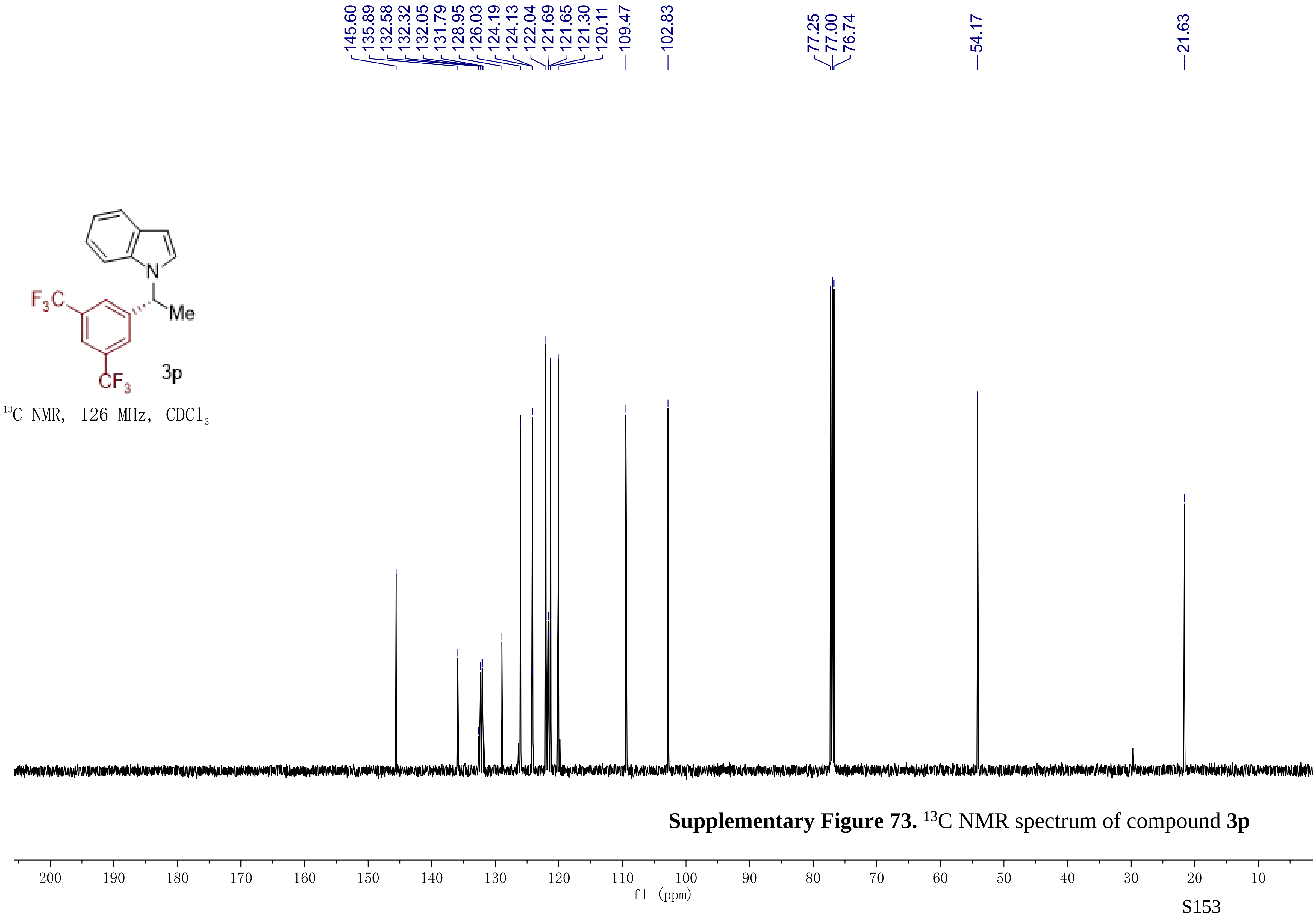

Supplementary Figure 73. ^{13}C NMR spectrum of compound **3p**

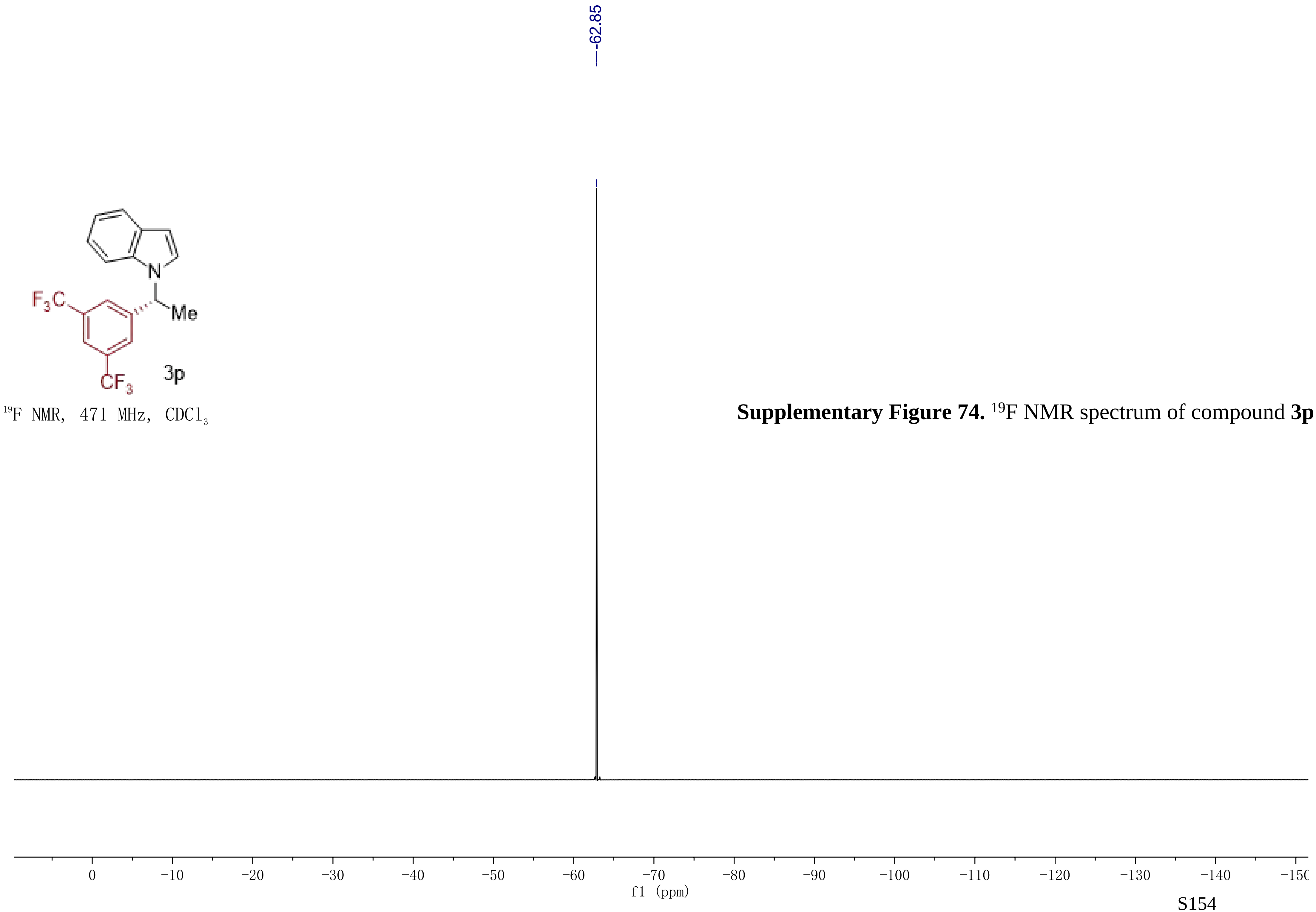

Supplementary Figure 74. ^{19}F NMR spectrum of compound **3p**

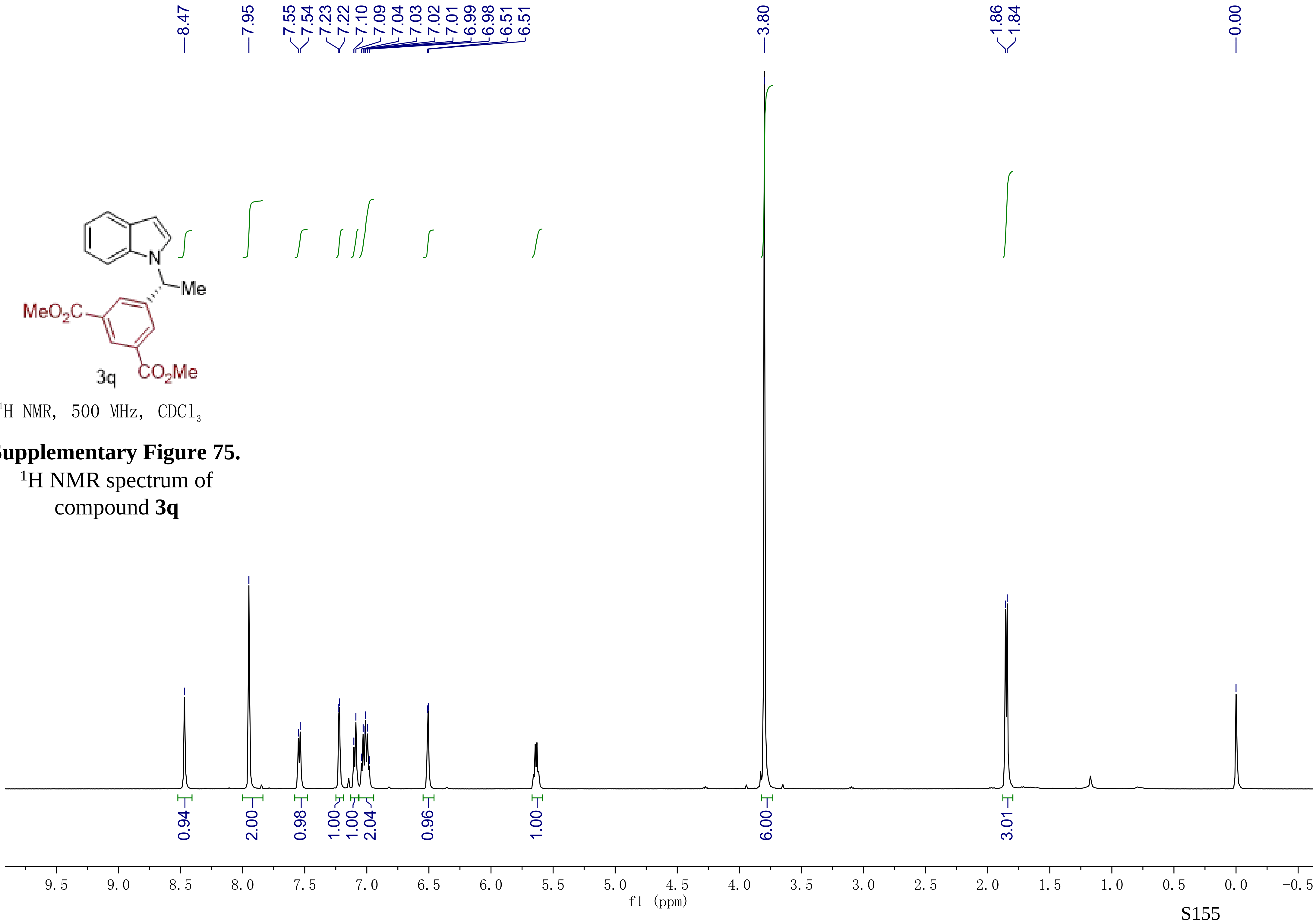

^{1}H NMR, 500 MHz, $CDCl_3$

Supplementary Figure 75. ^{1}H NMR spectrum of compound **3q**

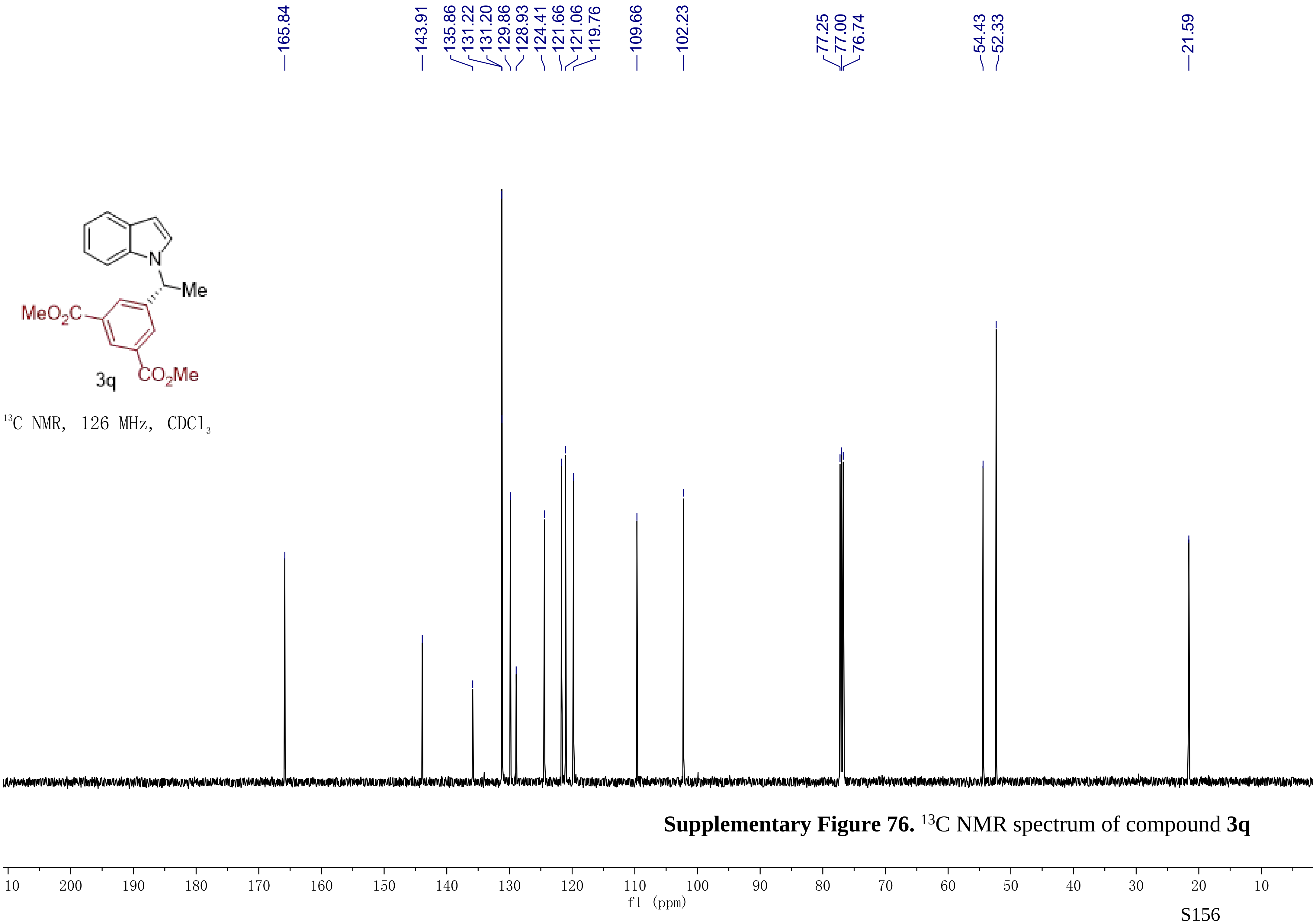

3q

^{13}C NMR, 126 MHz, $CDCl_3$

Supplementary Figure 76. ^{13}C NMR spectrum of compound **3q**

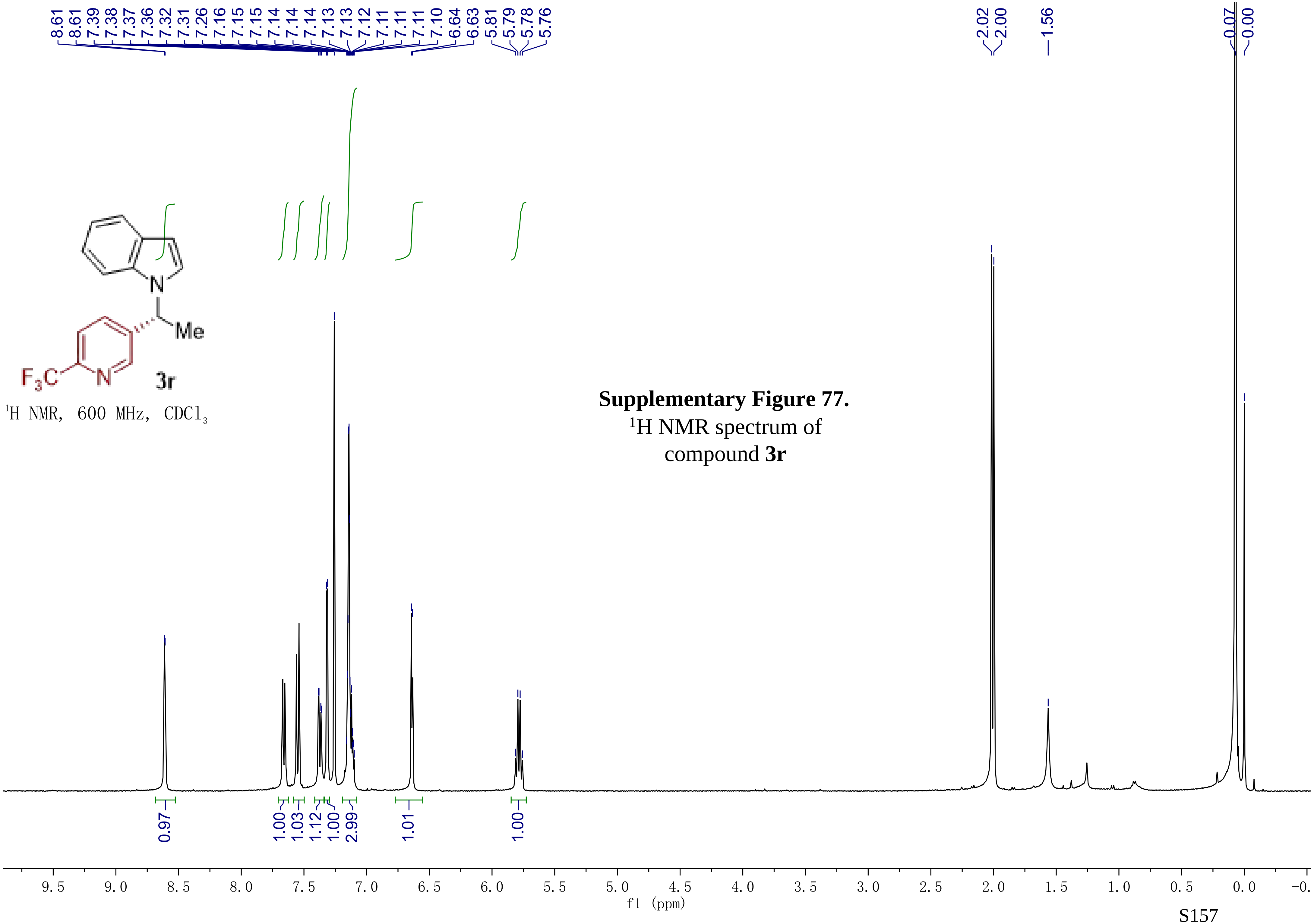

^{1}H NMR, 600 MHz, $CDCl_3$

Supplementary Figure 77.
^{1}H NMR spectrum of compound **3r**

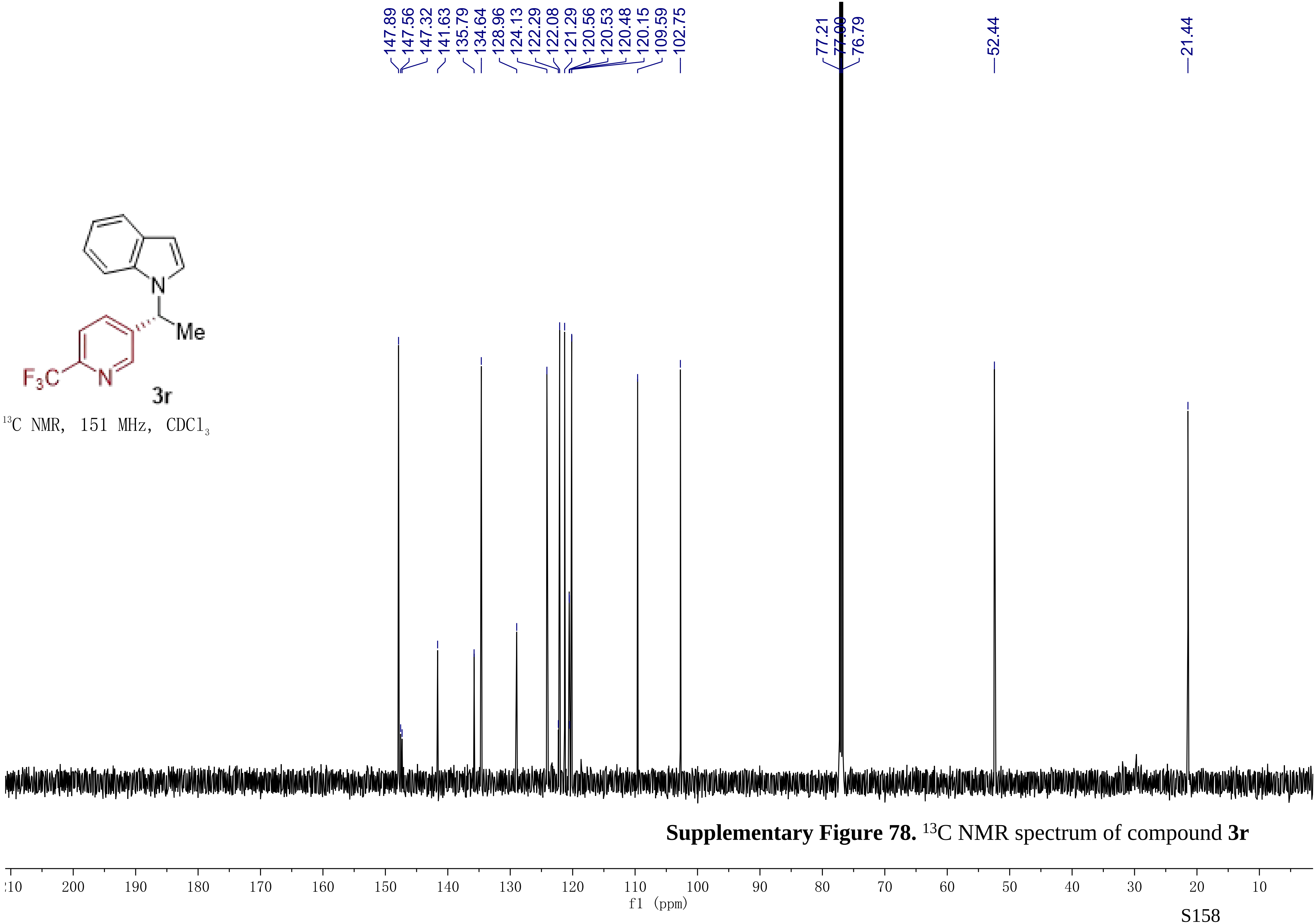

Supplementary Figure 78. ^{13}C NMR spectrum of compound **3r**

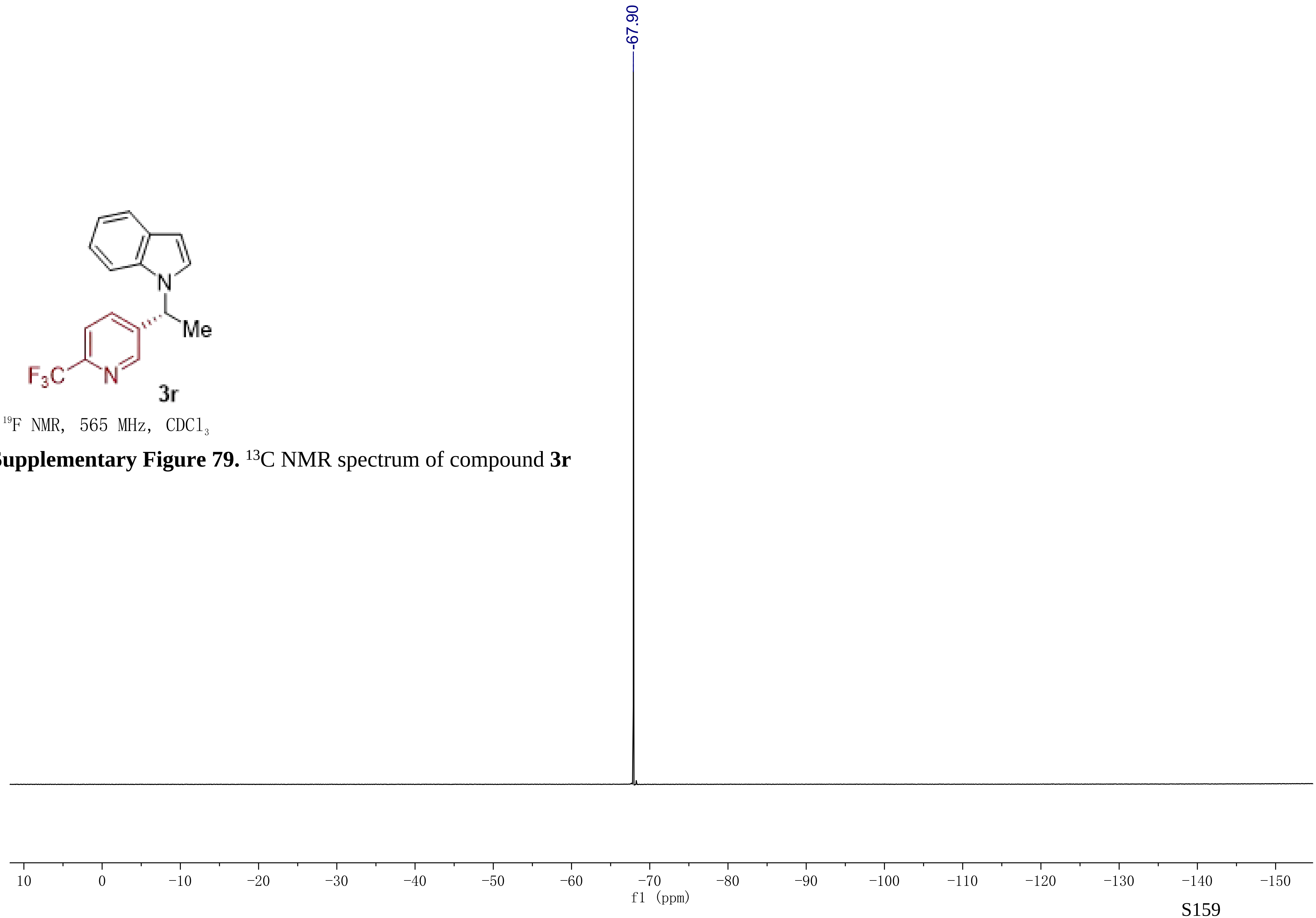

Supplementary Figure 79. ^{13}C NMR spectrum of compound **3r**

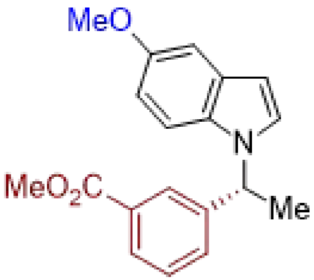

3s

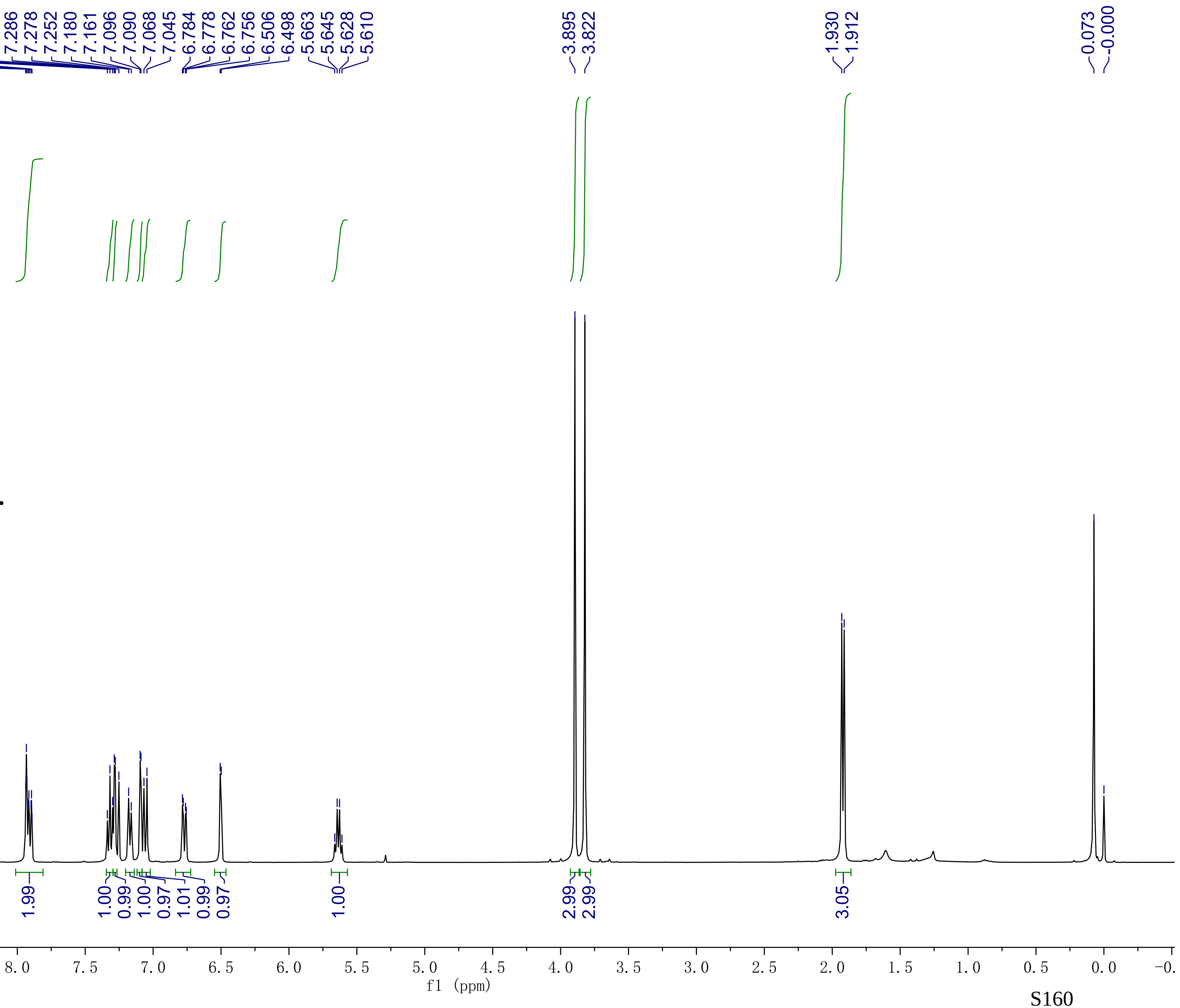

^{1}H NMR, 400 MHz, $CDCl_3$

Supplementary Figure 80. ^{1}H NMR spectrum of compound **3s**

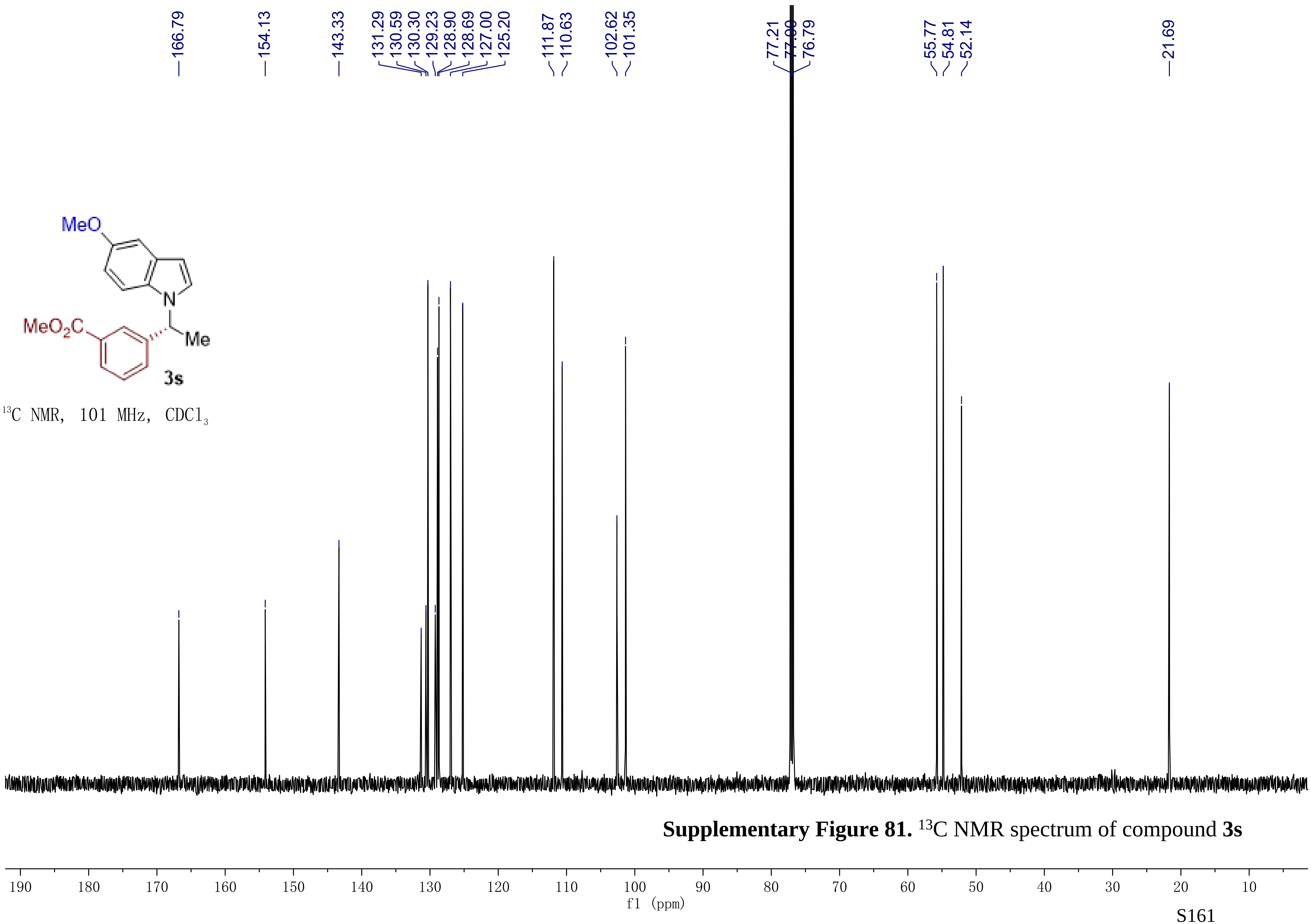

Supplementary Figure 81. ^{13}C NMR spectrum of compound **3s**

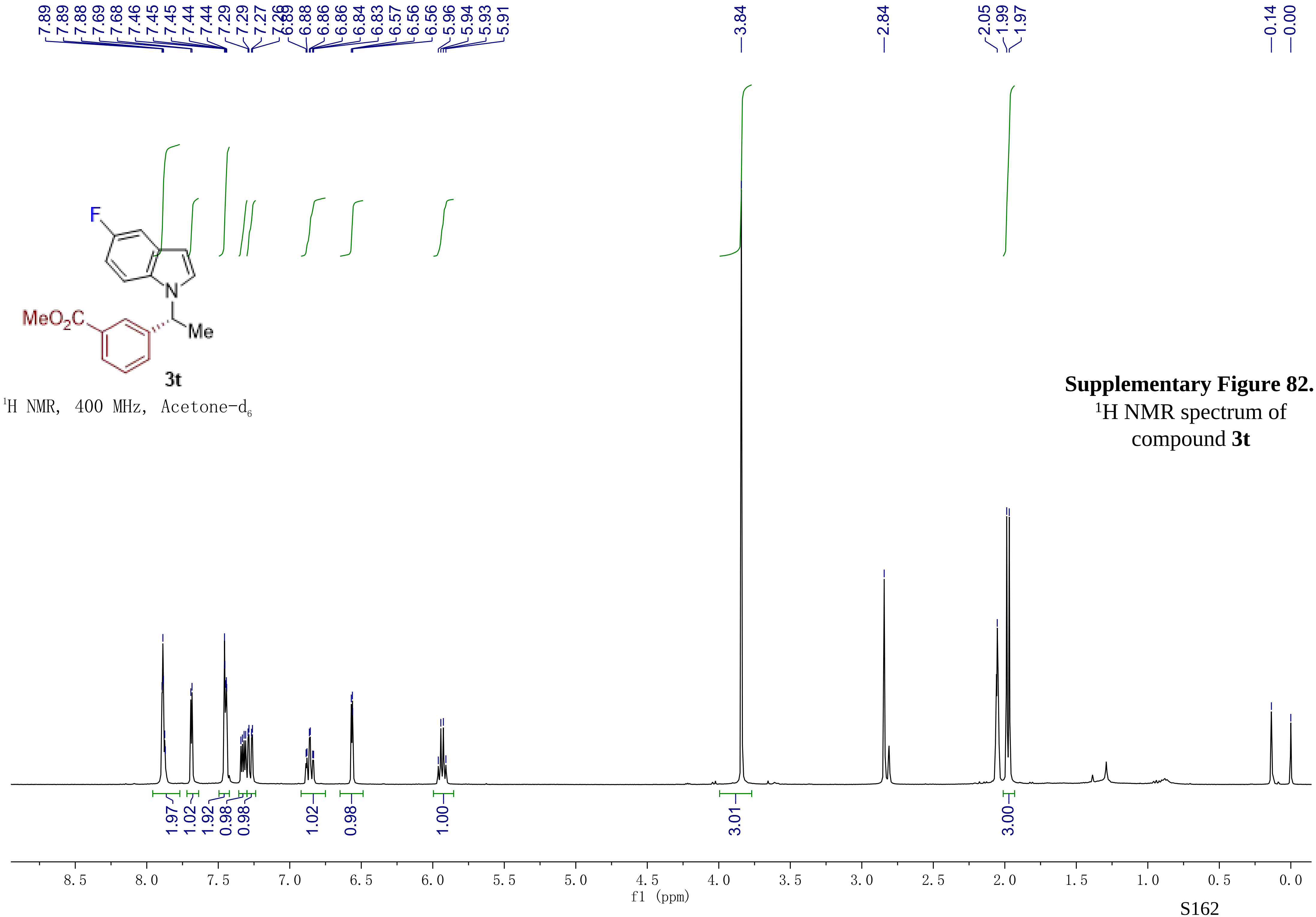

Supplementary Figure 82. ^{1}H NMR spectrum of compound **3t**

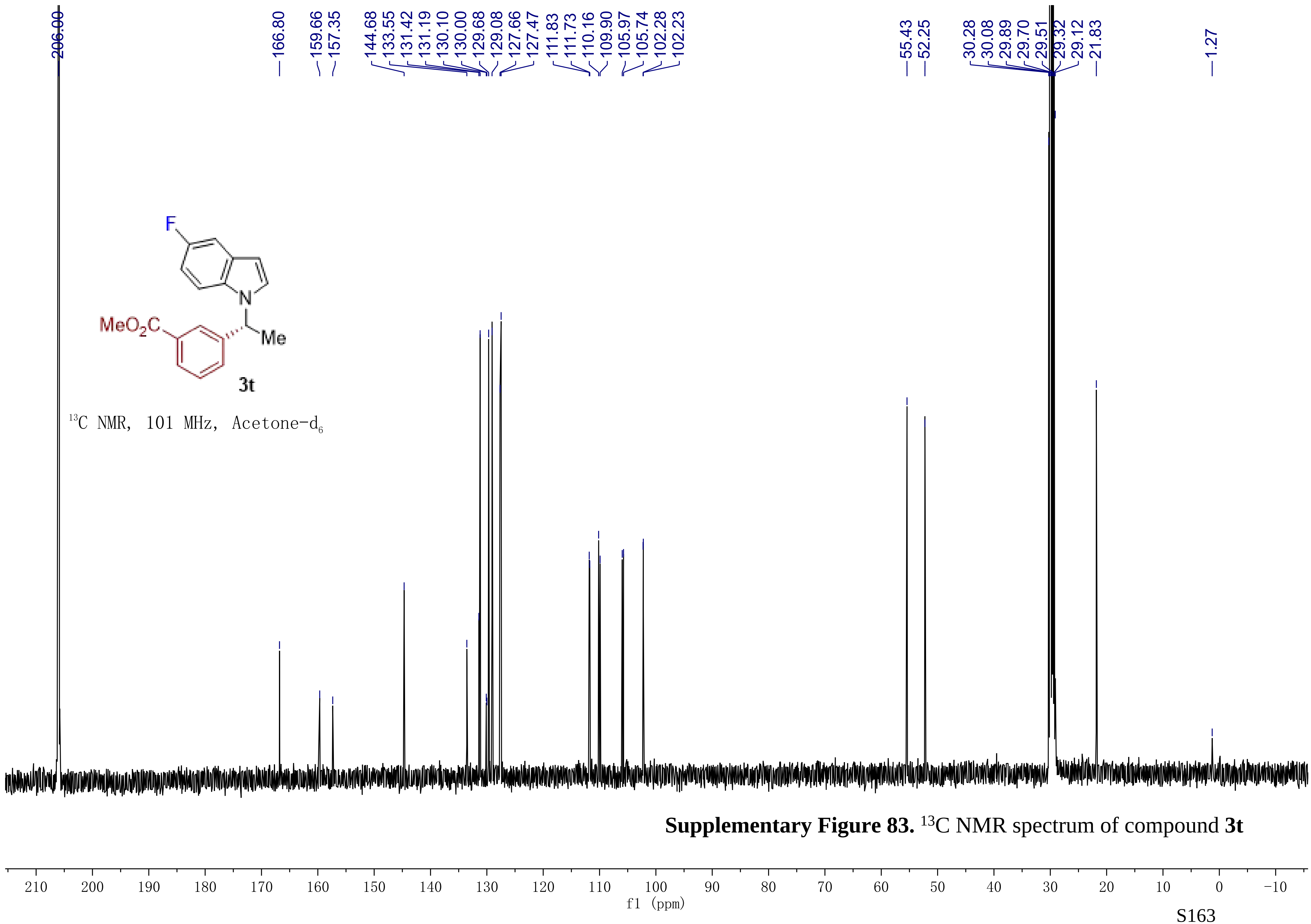

Supplementary Figure 83. ^{13}C NMR spectrum of compound **3t**

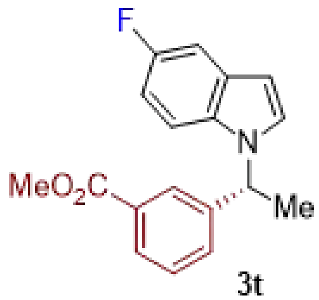

3t

^{19}F NMR, 376 MHz, Acetone-d_6

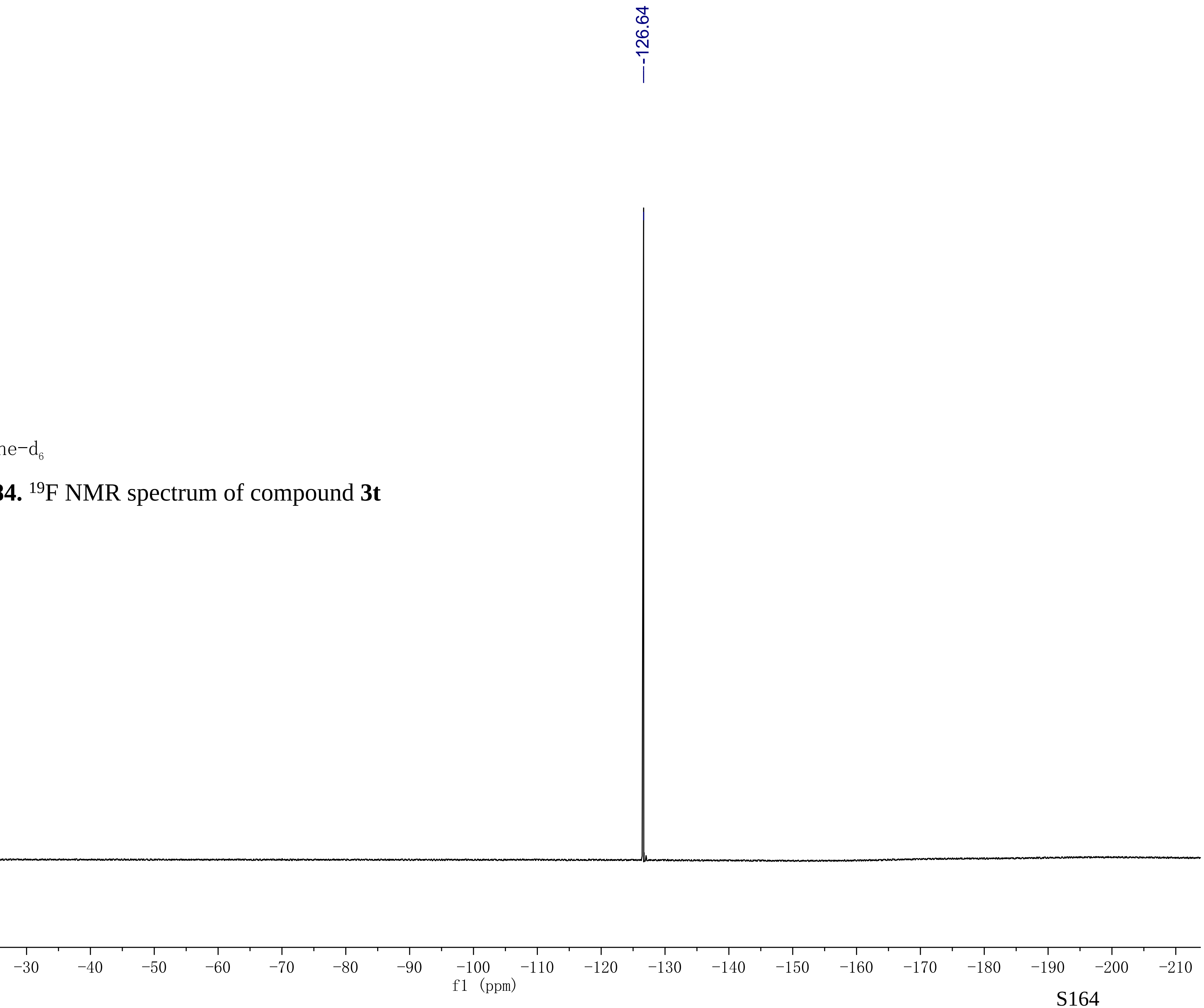

Supplementary Figure 84. ^{19}F NMR spectrum of compound **3t**

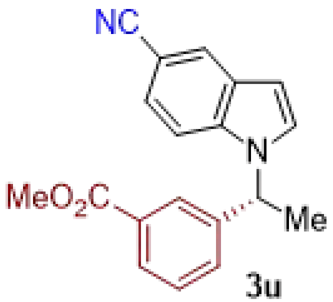

3u

^1H NMR, 400 MHz, $CDCl_3$

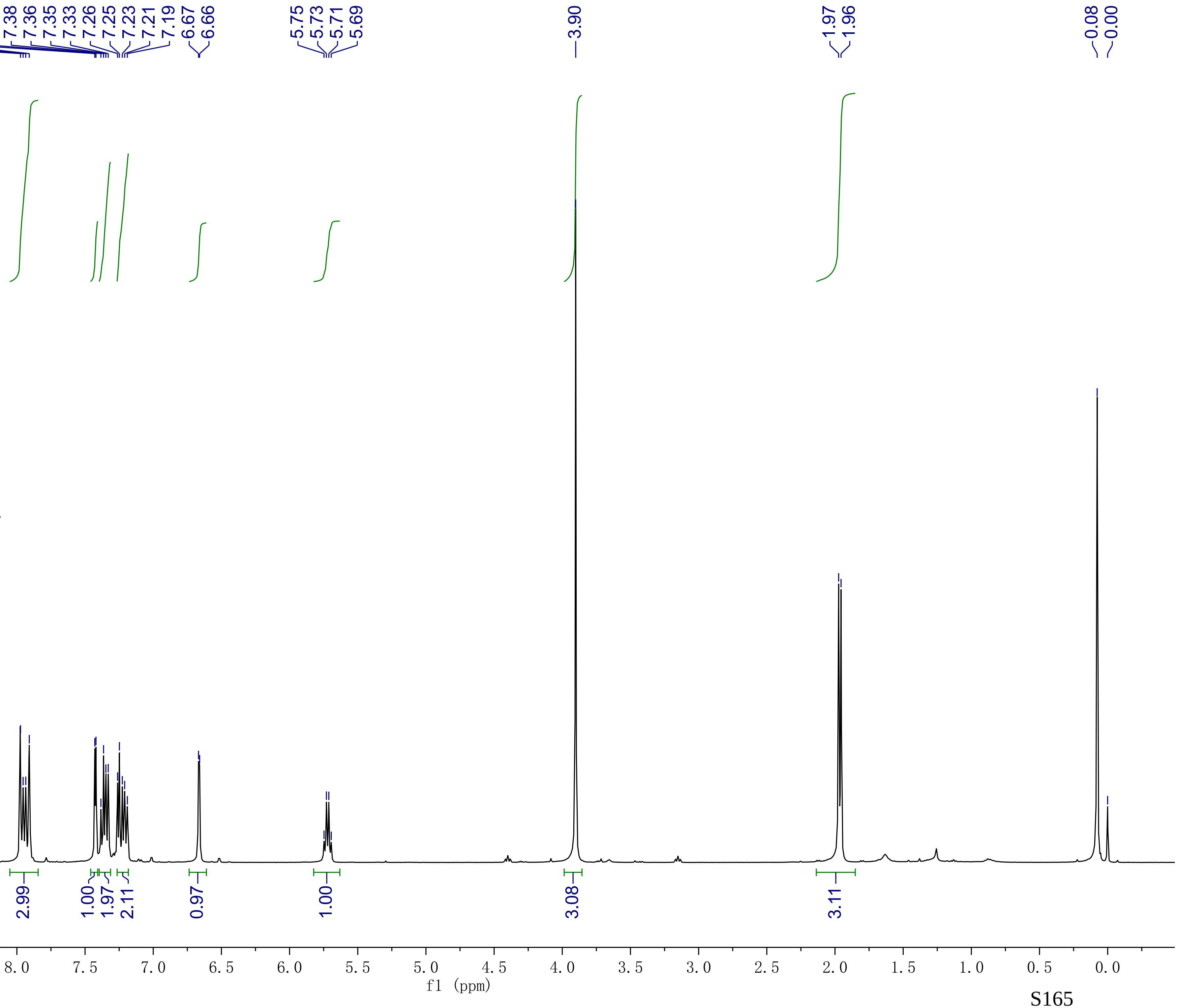

Supplementary Figure 85. ^1H NMR spectrum of compound **3u**

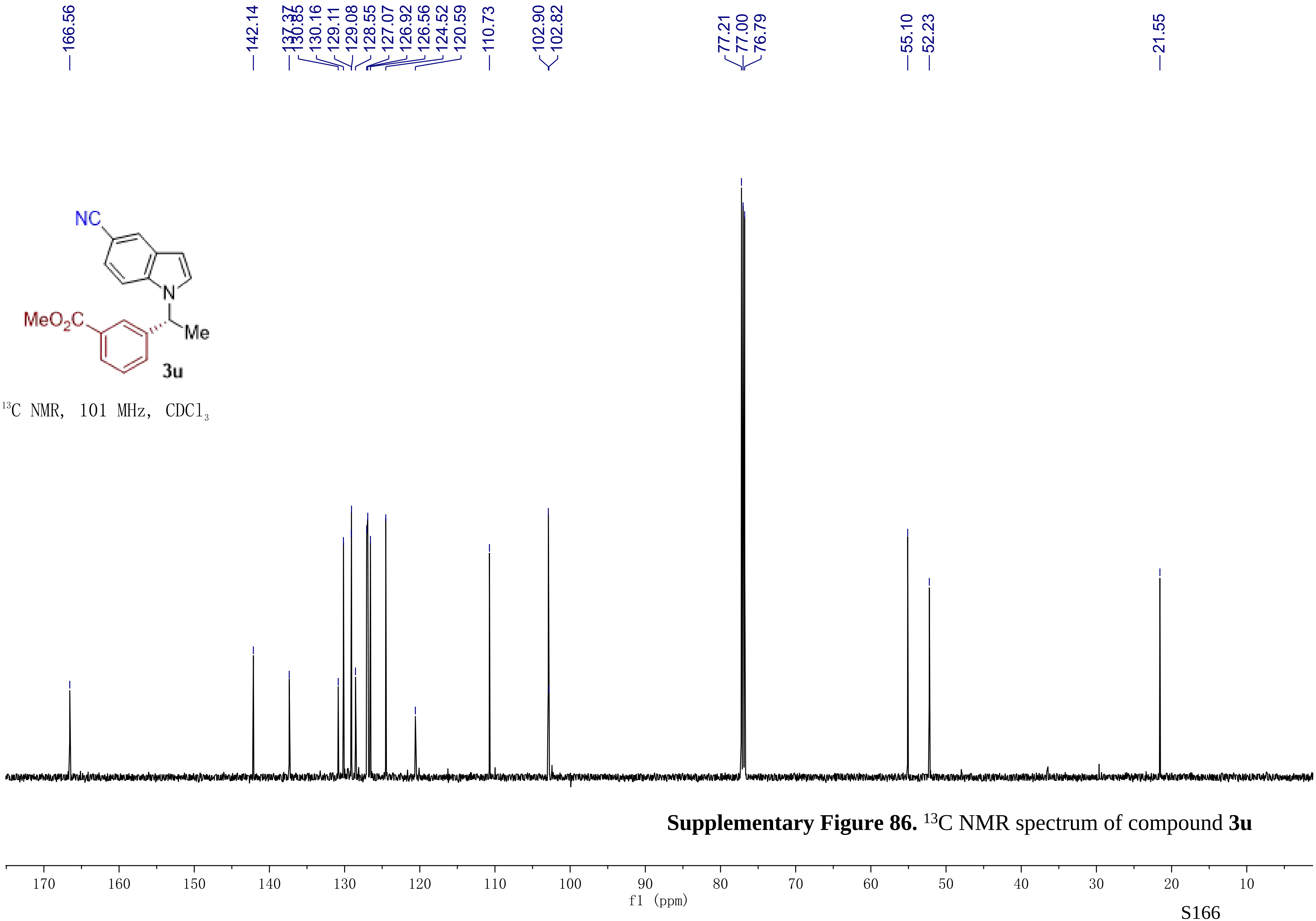

Supplementary Figure 86. ^{13}C NMR spectrum of compound **3u**

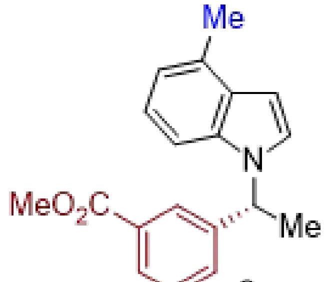

3v

^{1}H NMR, 400 MHz, $CDCl_3$

Supplementary Figure 87.
^{1}H NMR spectrum of compound **3v**

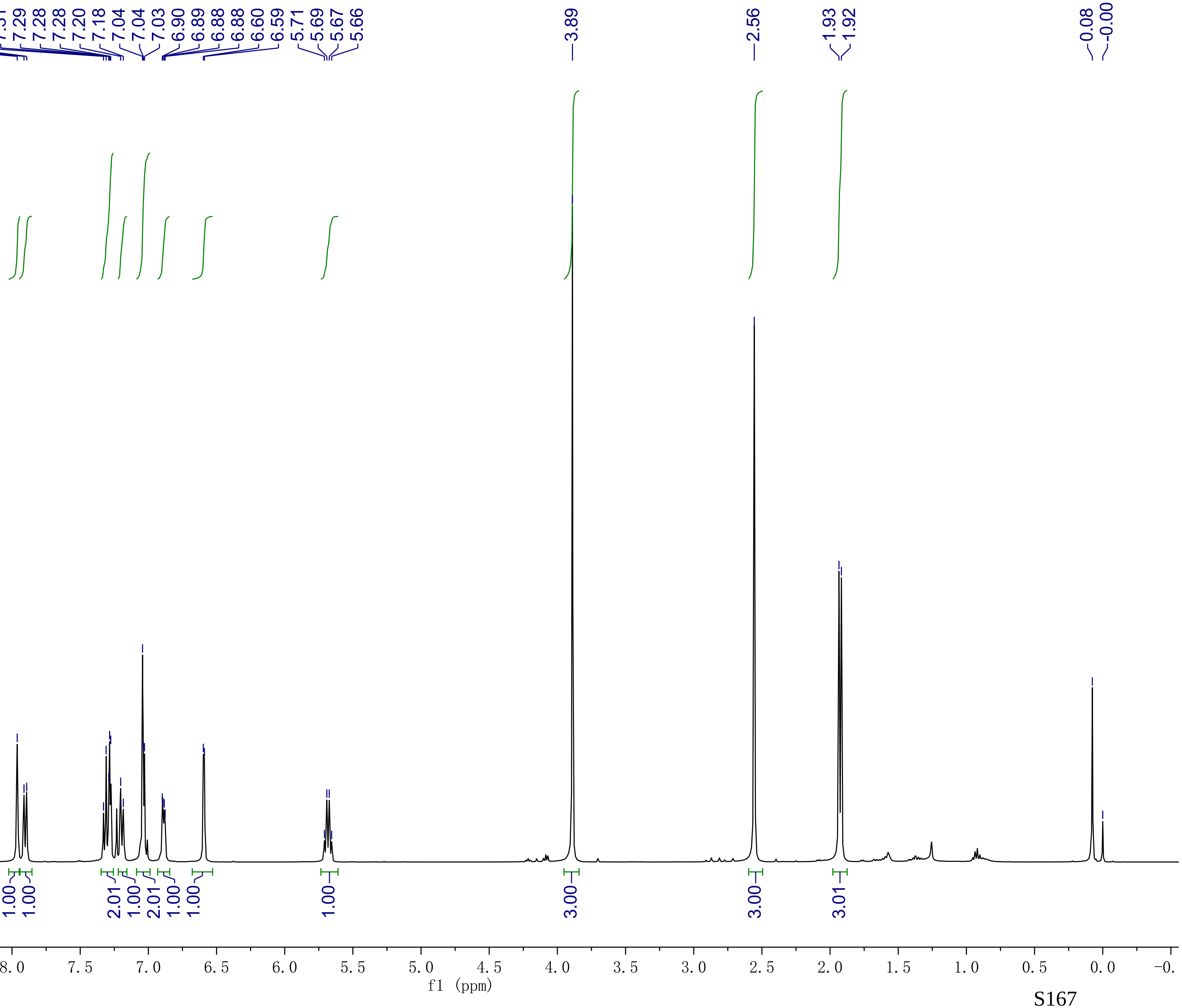

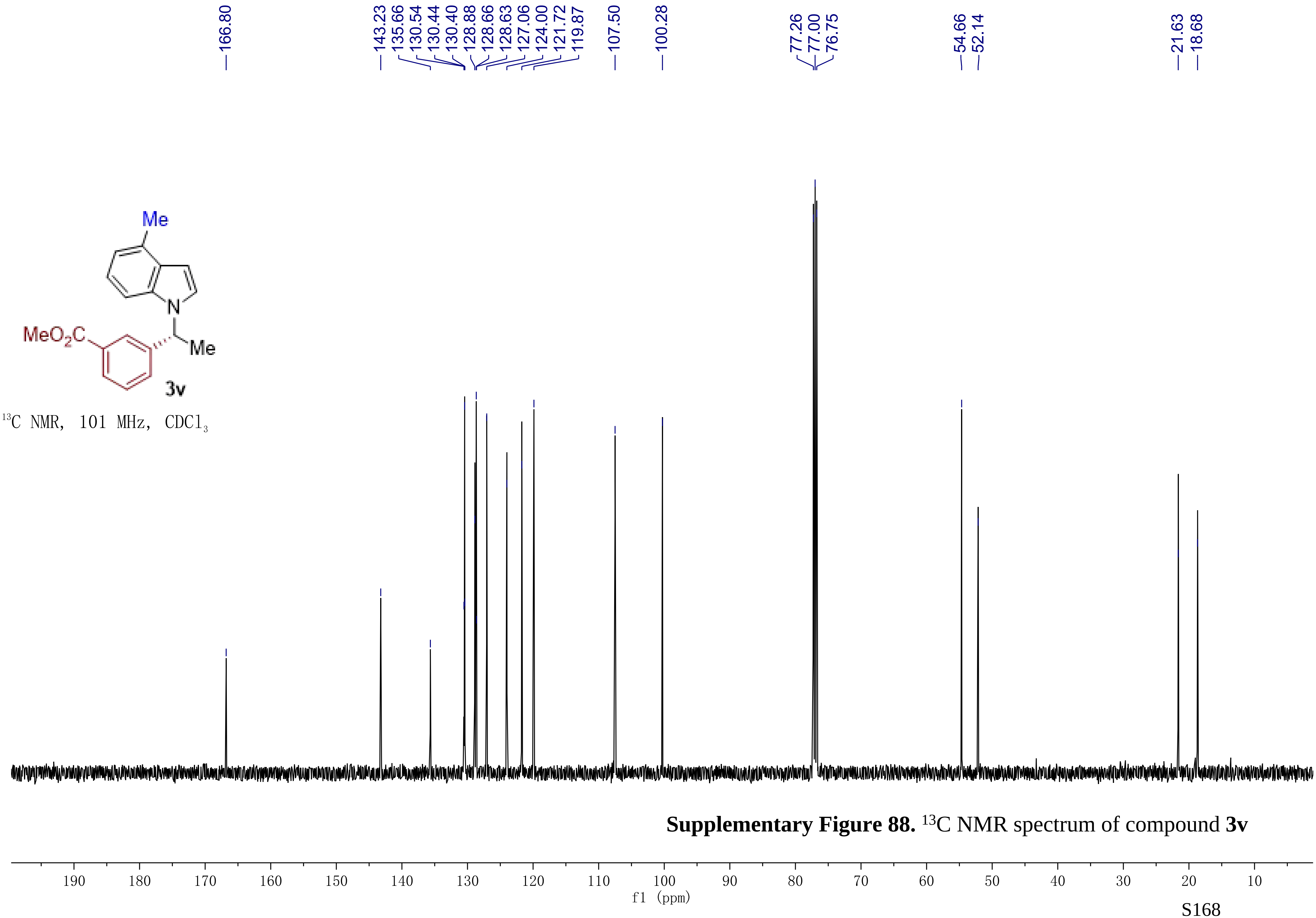

Supplementary Figure 88. ^{13}C NMR spectrum of compound **3v**

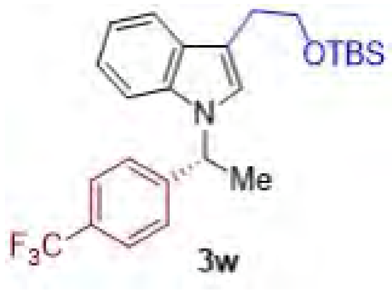

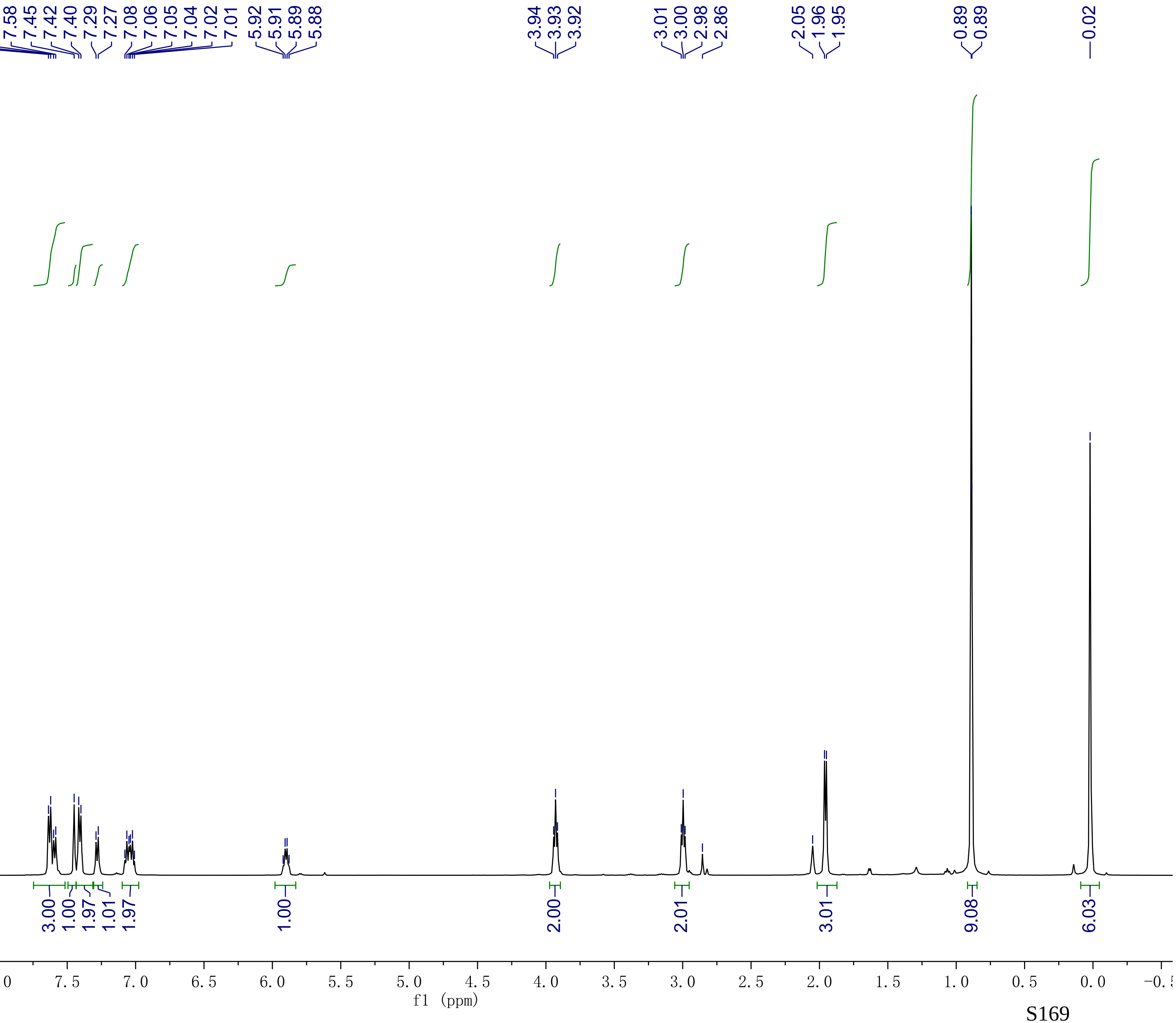

^{1}H NMR, 500 MHz, Acetone-d_6

Supplementary Figure 89. ^{1}H NMR spectrum of compound **3w**

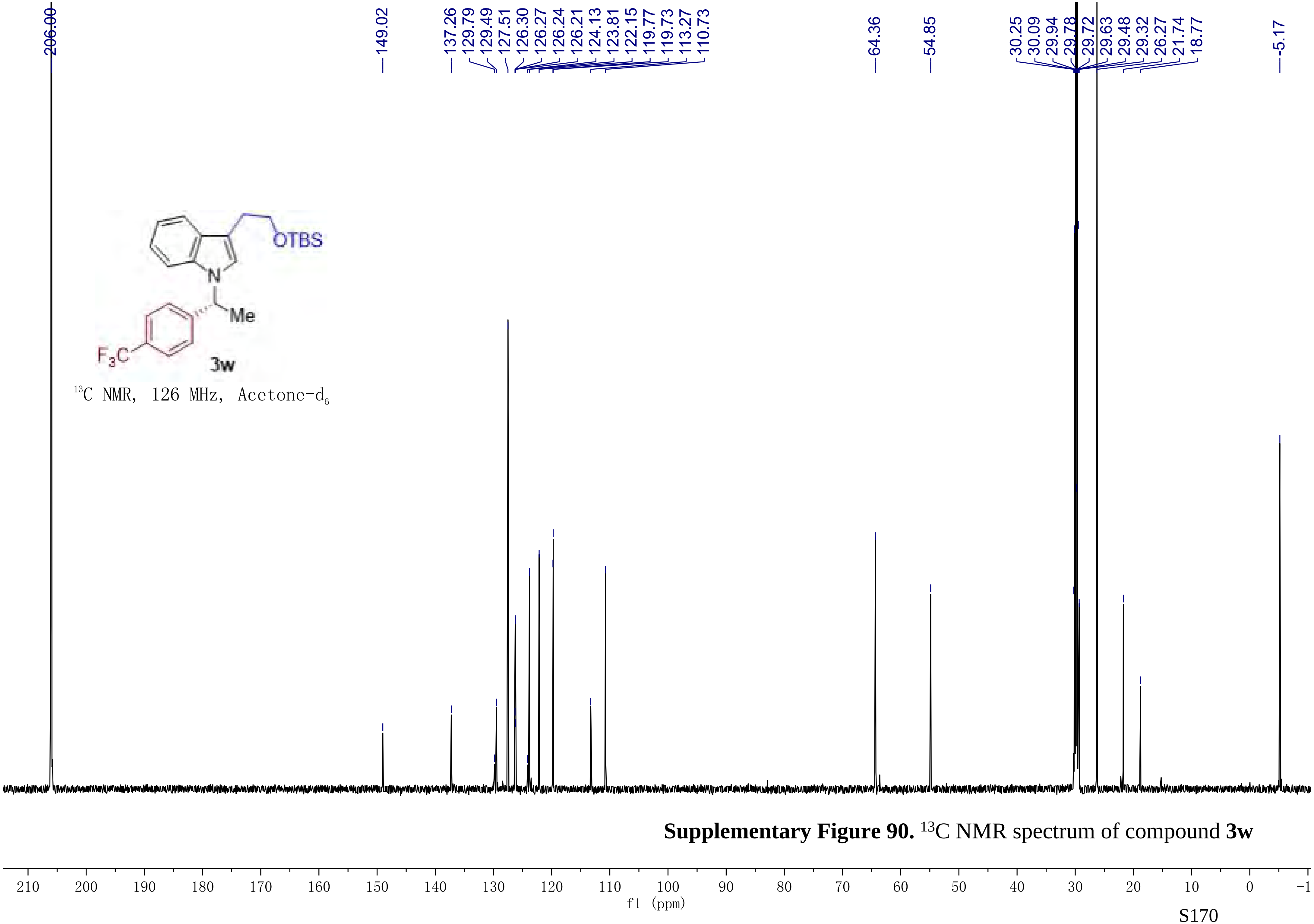

Supplementary Figure 90. ^{13}C NMR spectrum of compound **3w**

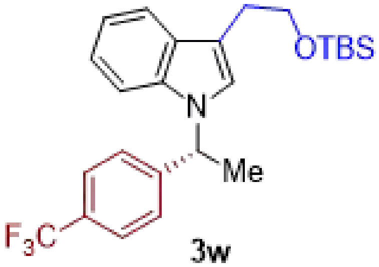

3w

^{19}F NMR, 471 MHz, Acetone-d_6

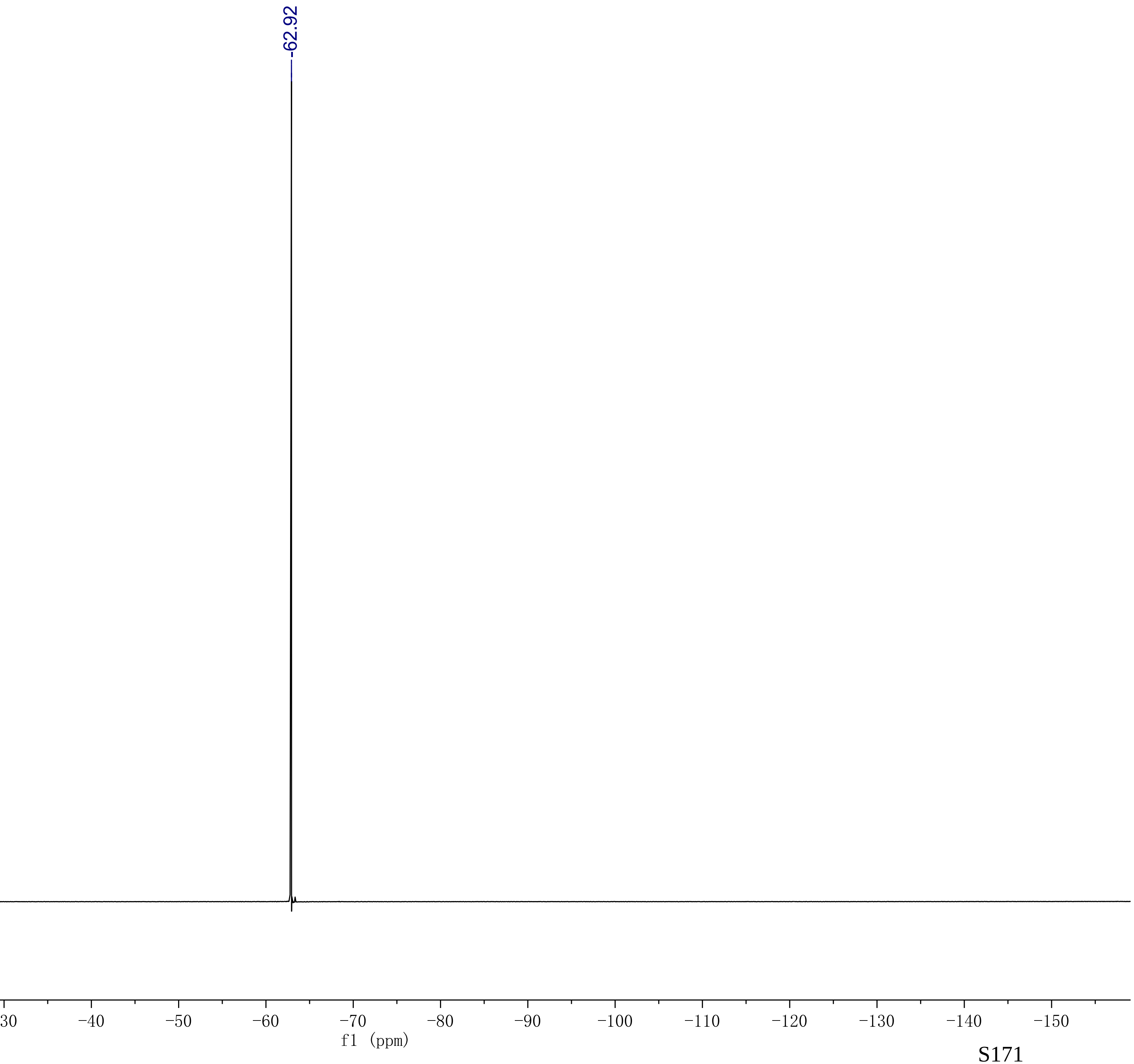

Supplementary Figure 91.
^{19}F NMR spectrum of compound **3w**

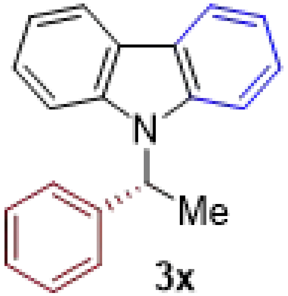

3x

^{1}H NMR, 500 MHz, $CDCl_3$

Supplementary Figure 92. ^{1}H NMR spectrum of compound **3x**

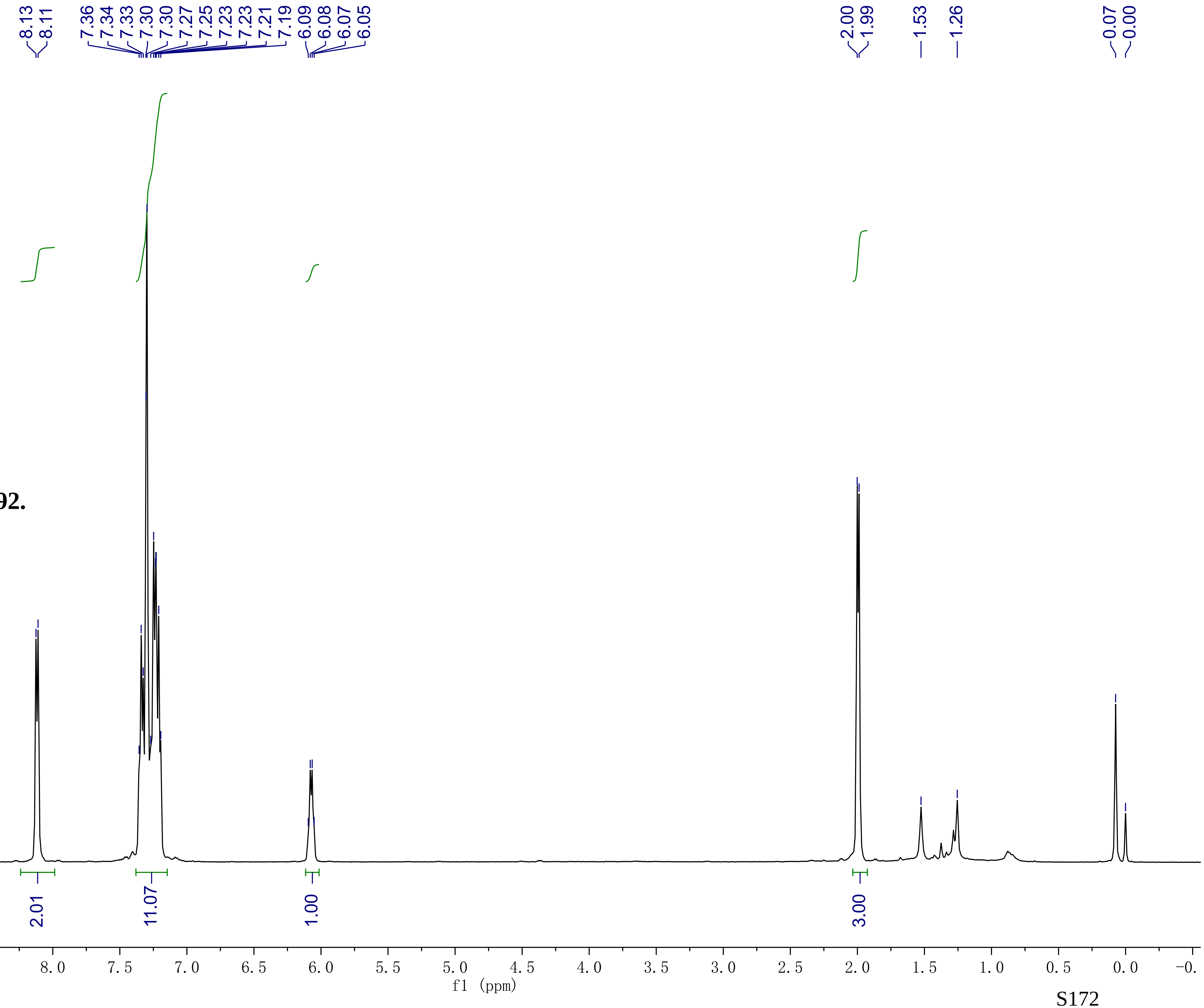

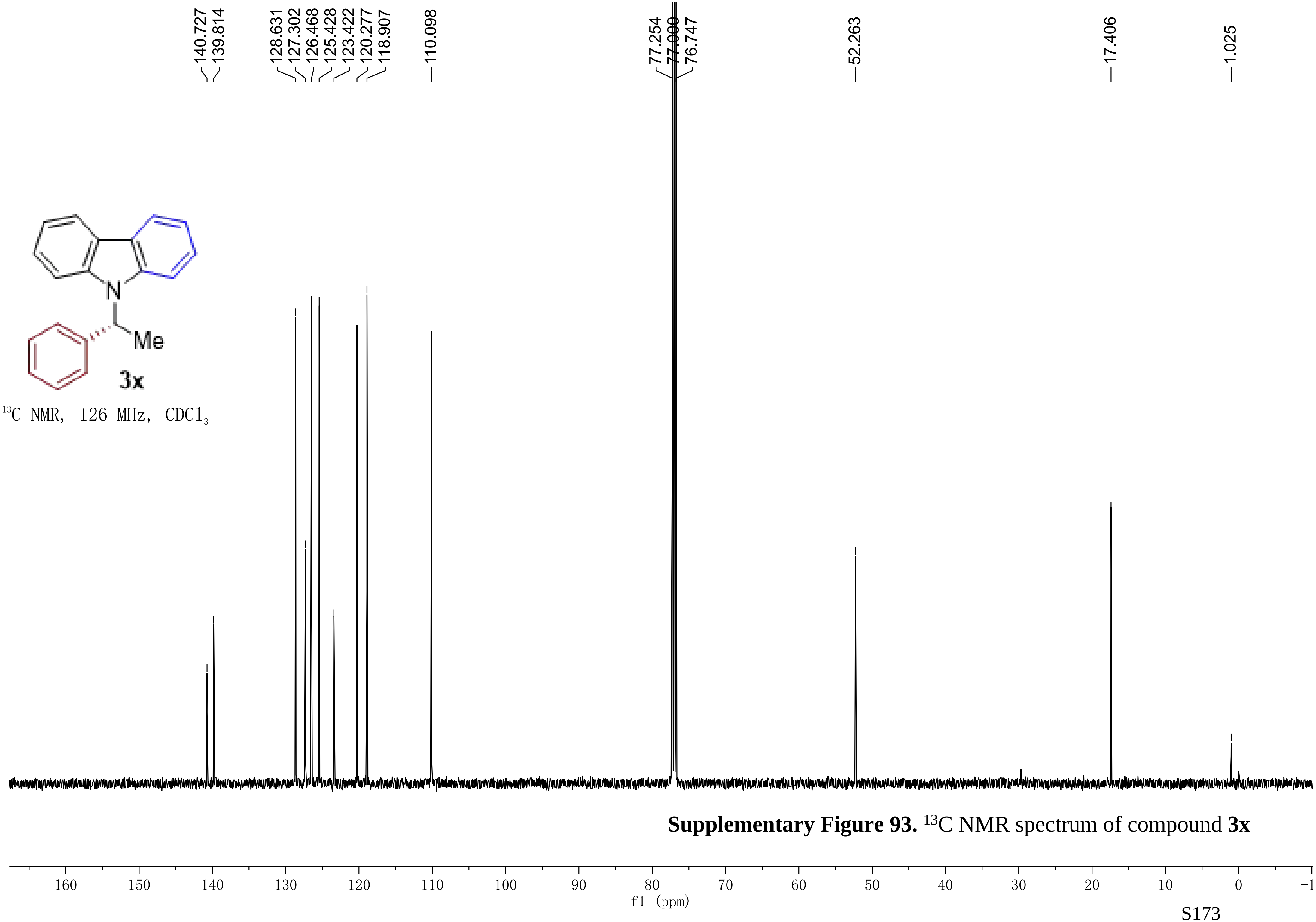

Supplementary Figure 93. ^{13}C NMR spectrum of compound **3x**

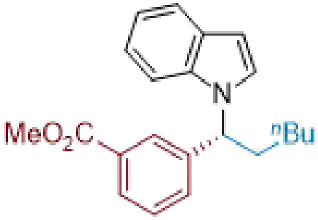

3y

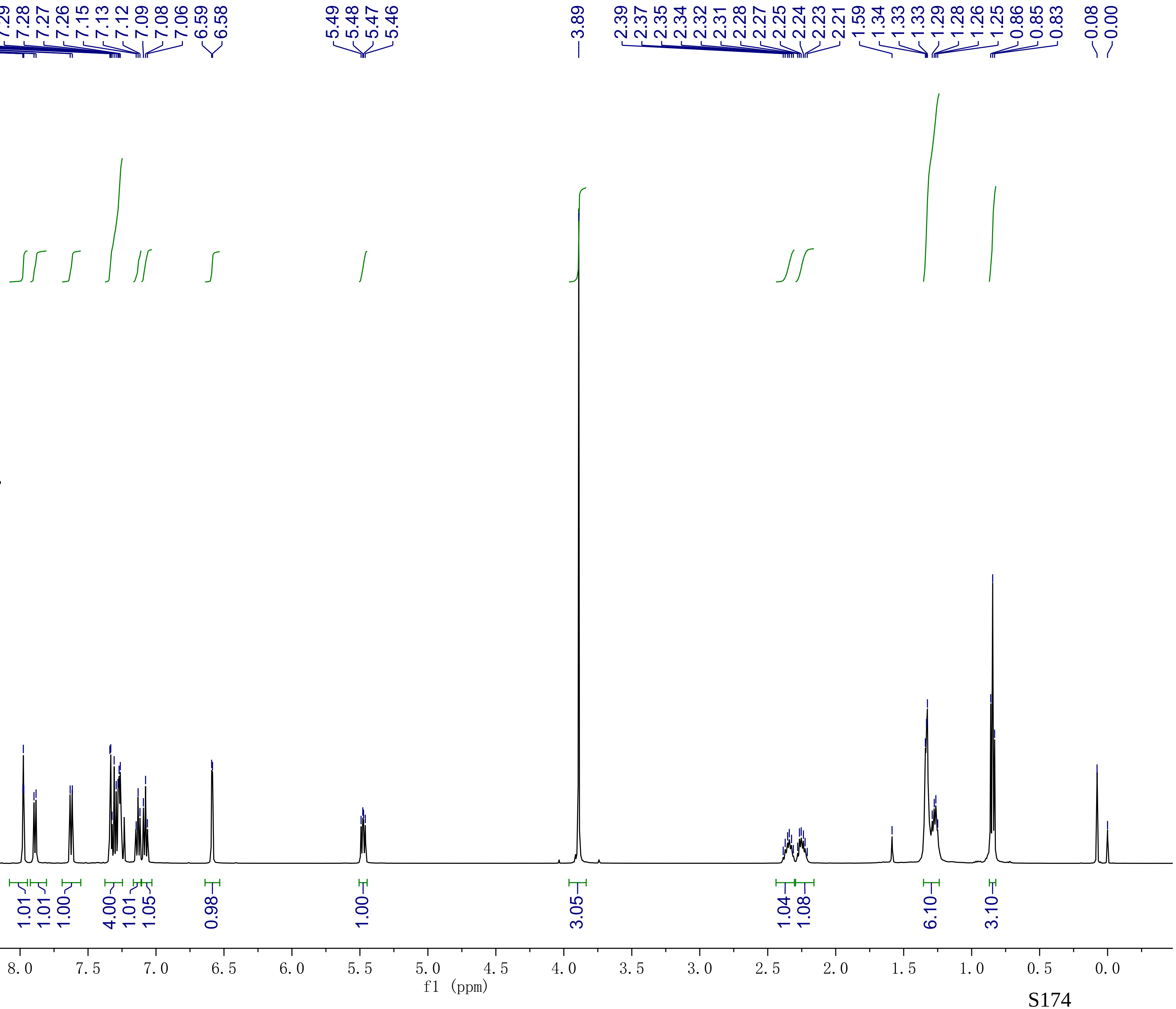

Supplementary Figure 94. ^{1}H NMR spectrum of compound **3y**

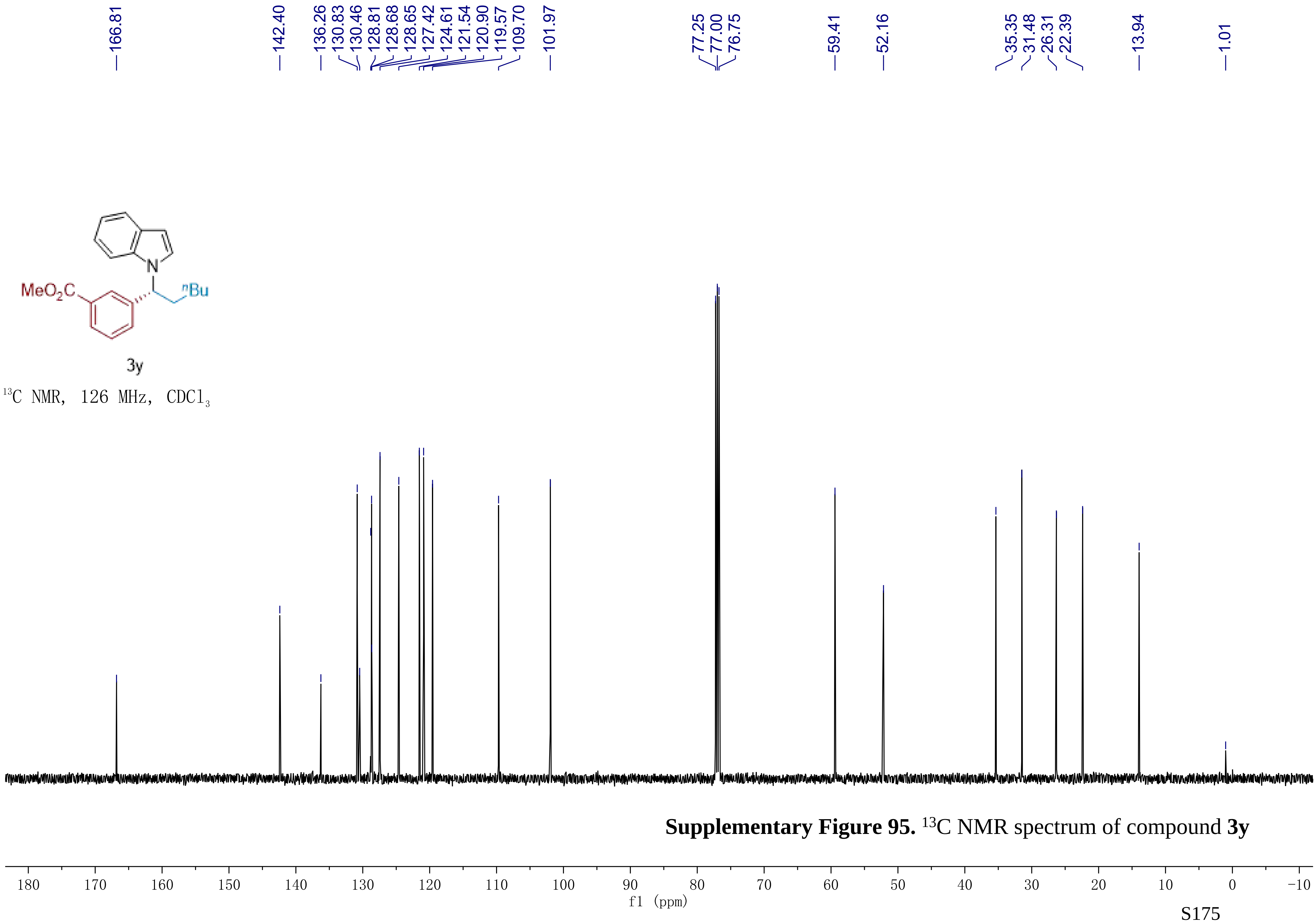

Supplementary Figure 95. ^{13}C NMR spectrum of compound **3y**

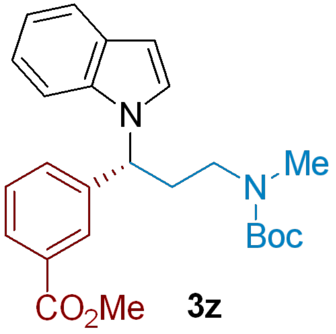

3z

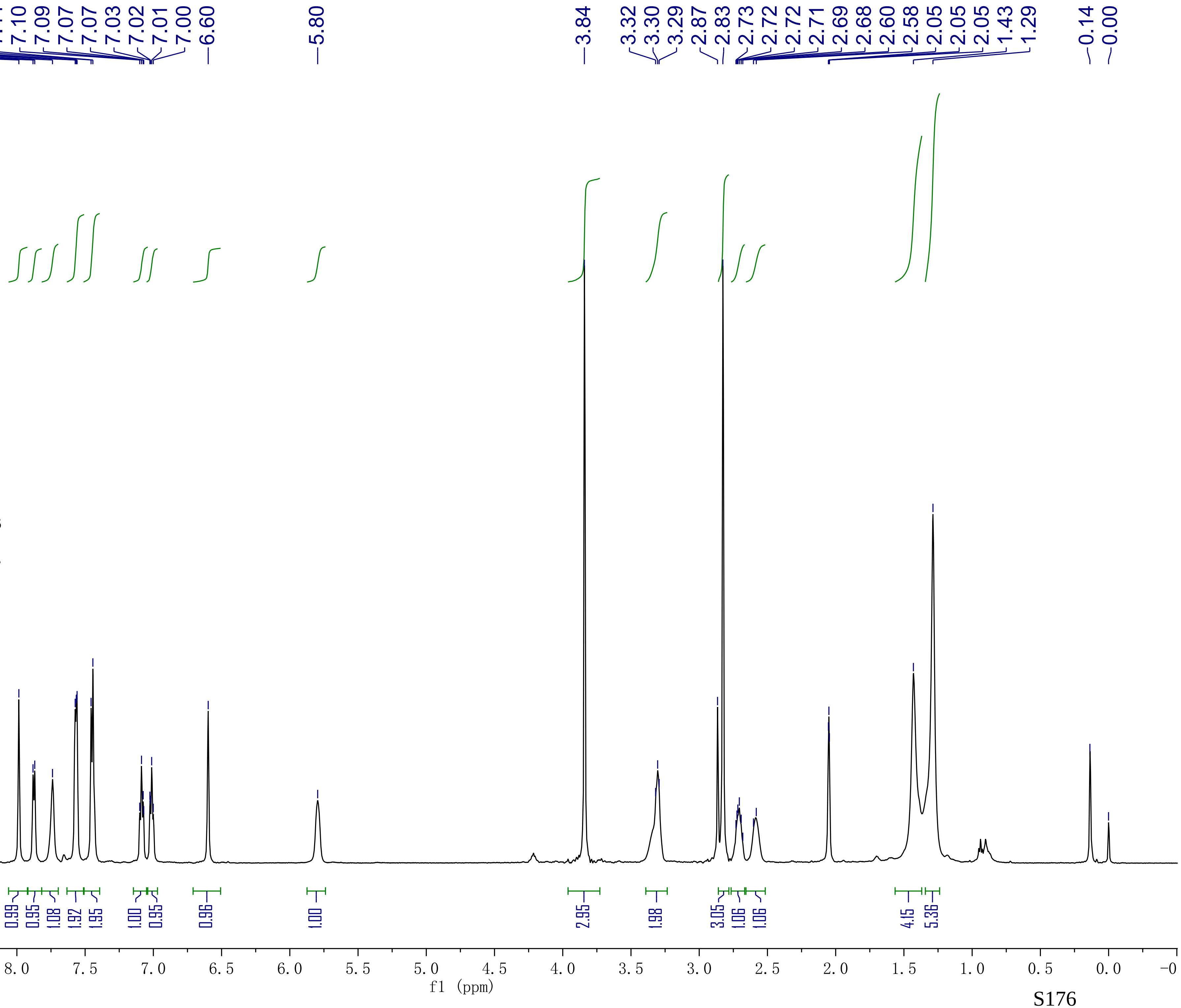

^{1}H NMR, 600 MHz, Acetone-d_6

Supplementary Figure 96. ^{1}H NMR spectrum of compound **3z**

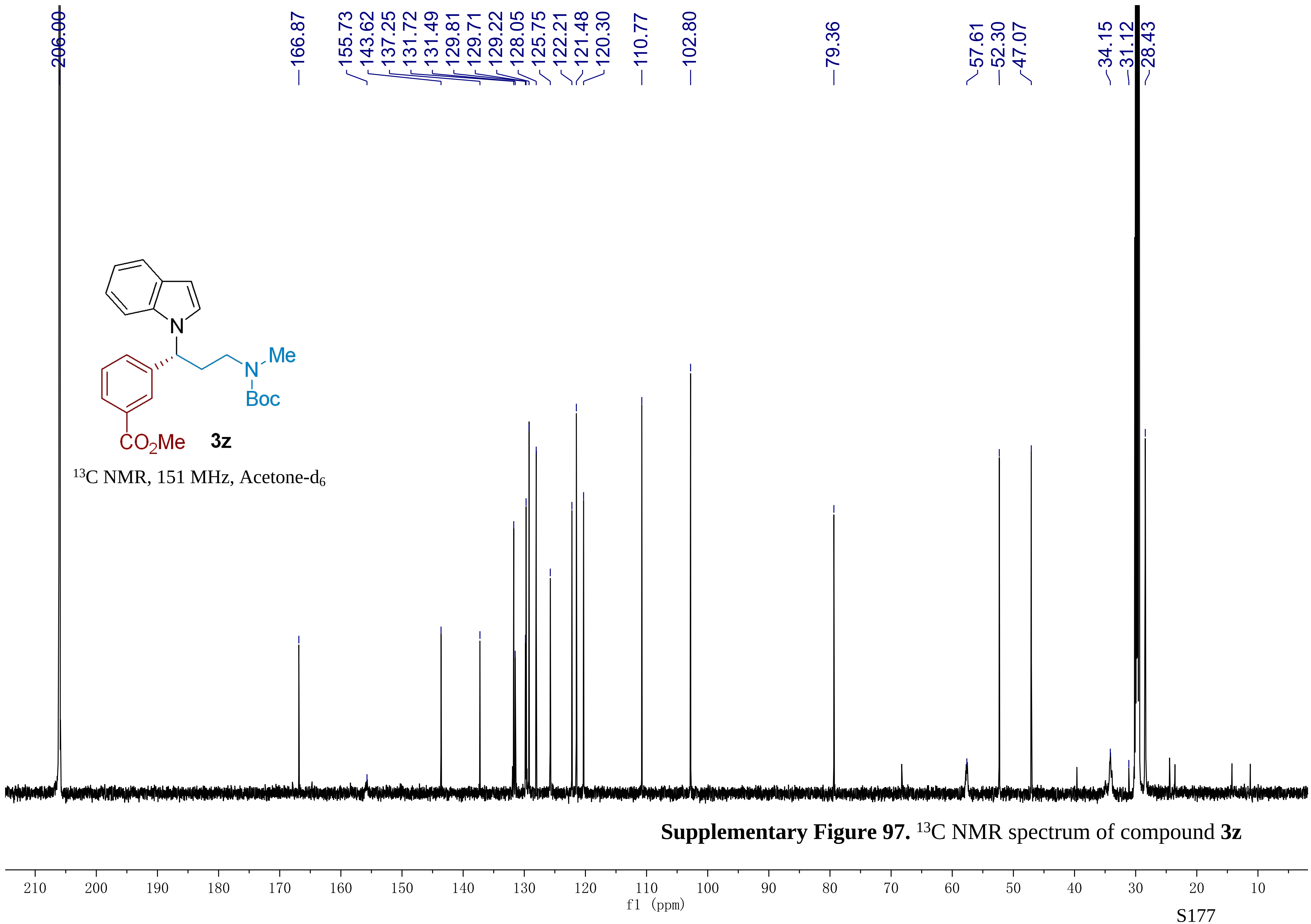

Supplementary Figure 97. ^{13}C NMR spectrum of compound **3z**

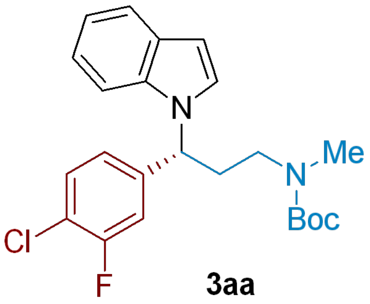

3aa

^{1}H NMR, 600 MHz, Acetone-d_6

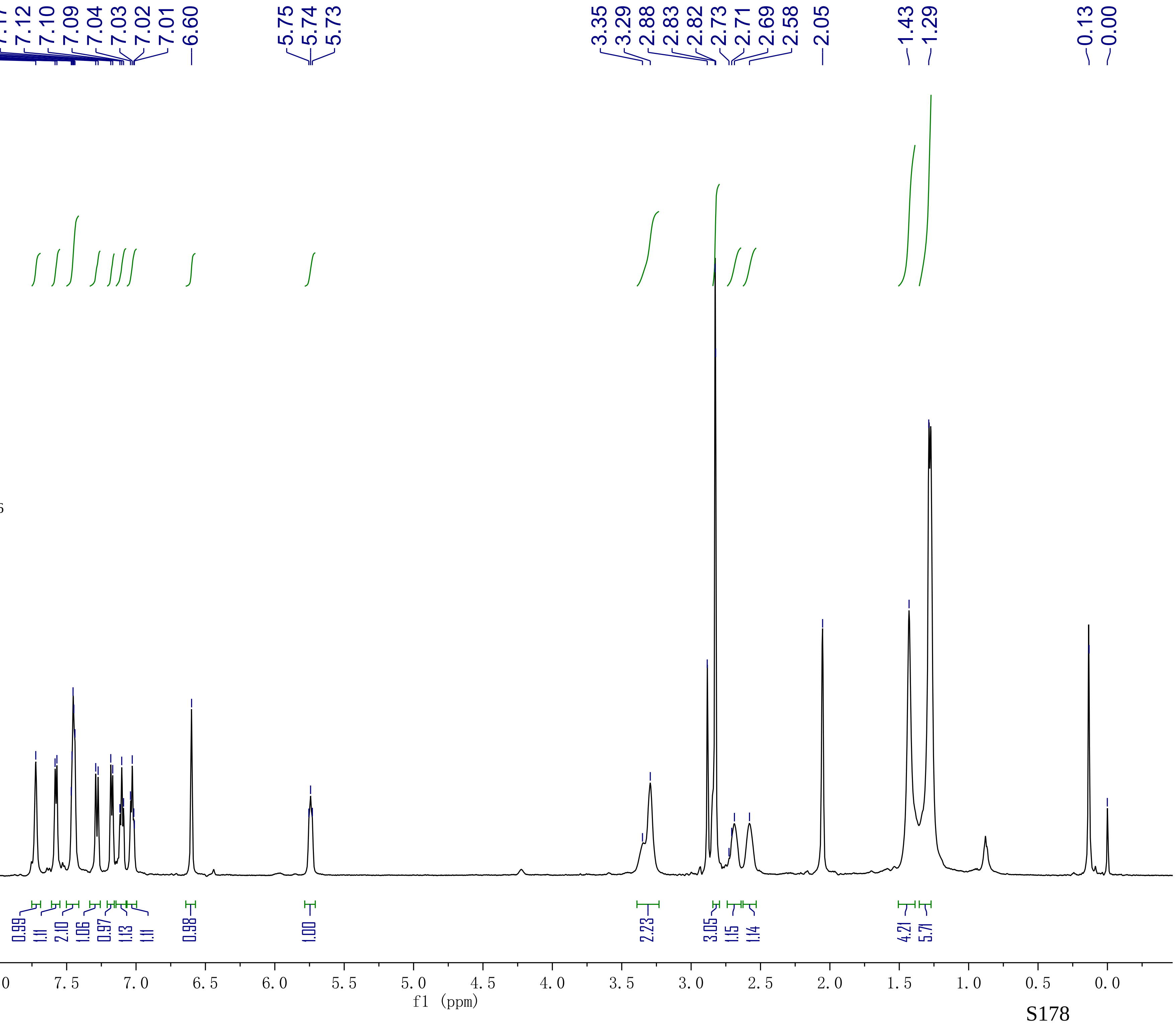

Supplementary Figure 98.
^{1}H NMR spectrum of compound **3aa**

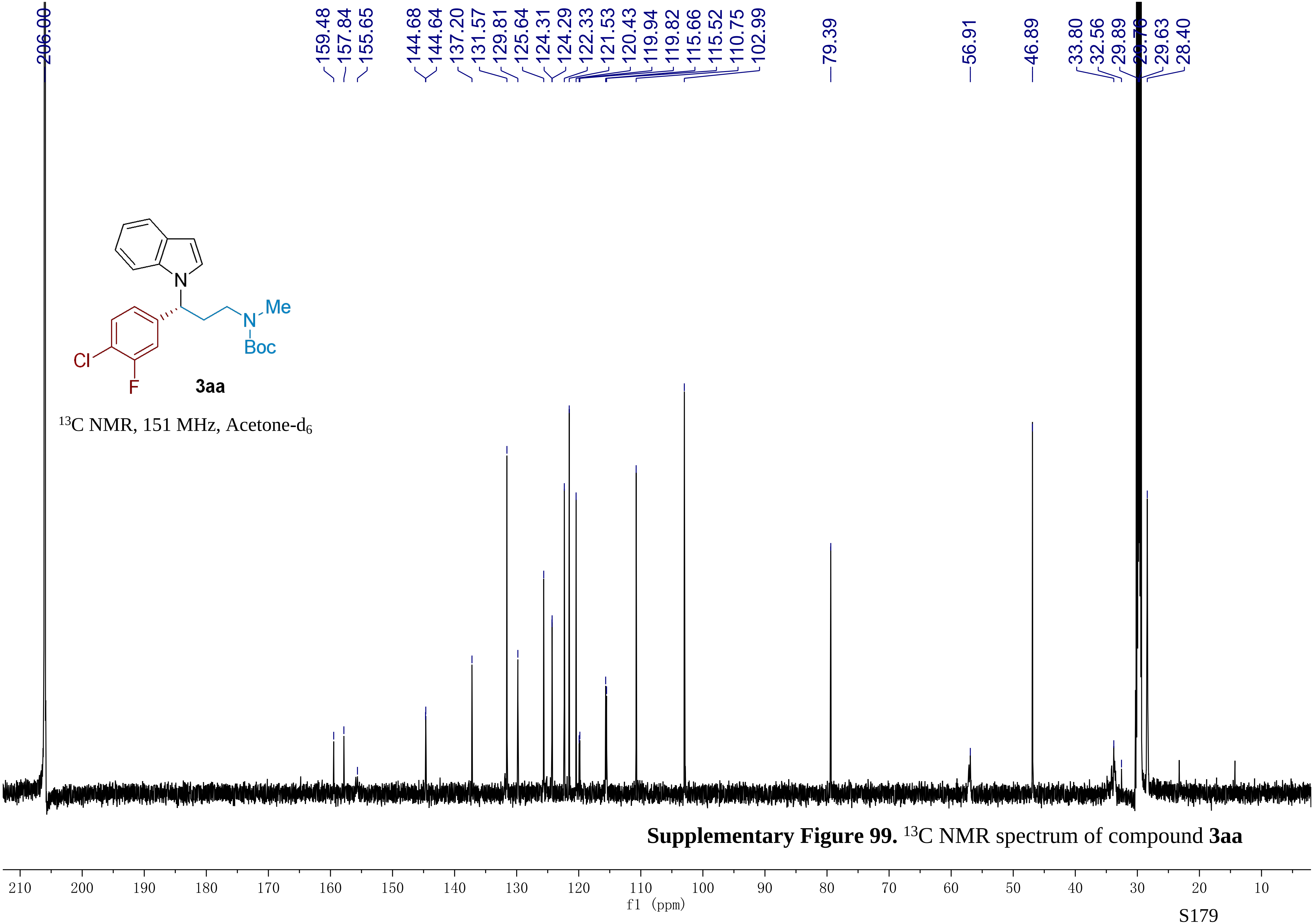

Supplementary Figure 99. ^{13}C NMR spectrum of compound **3aa**

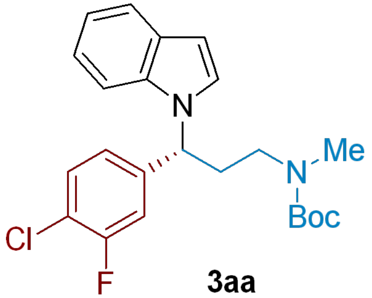

3aa

^{19}F NMR, 565 MHz, Acetone-d_6

Supplementary Figure 100. ^{19}F NMR spectrum of compound **3aa**

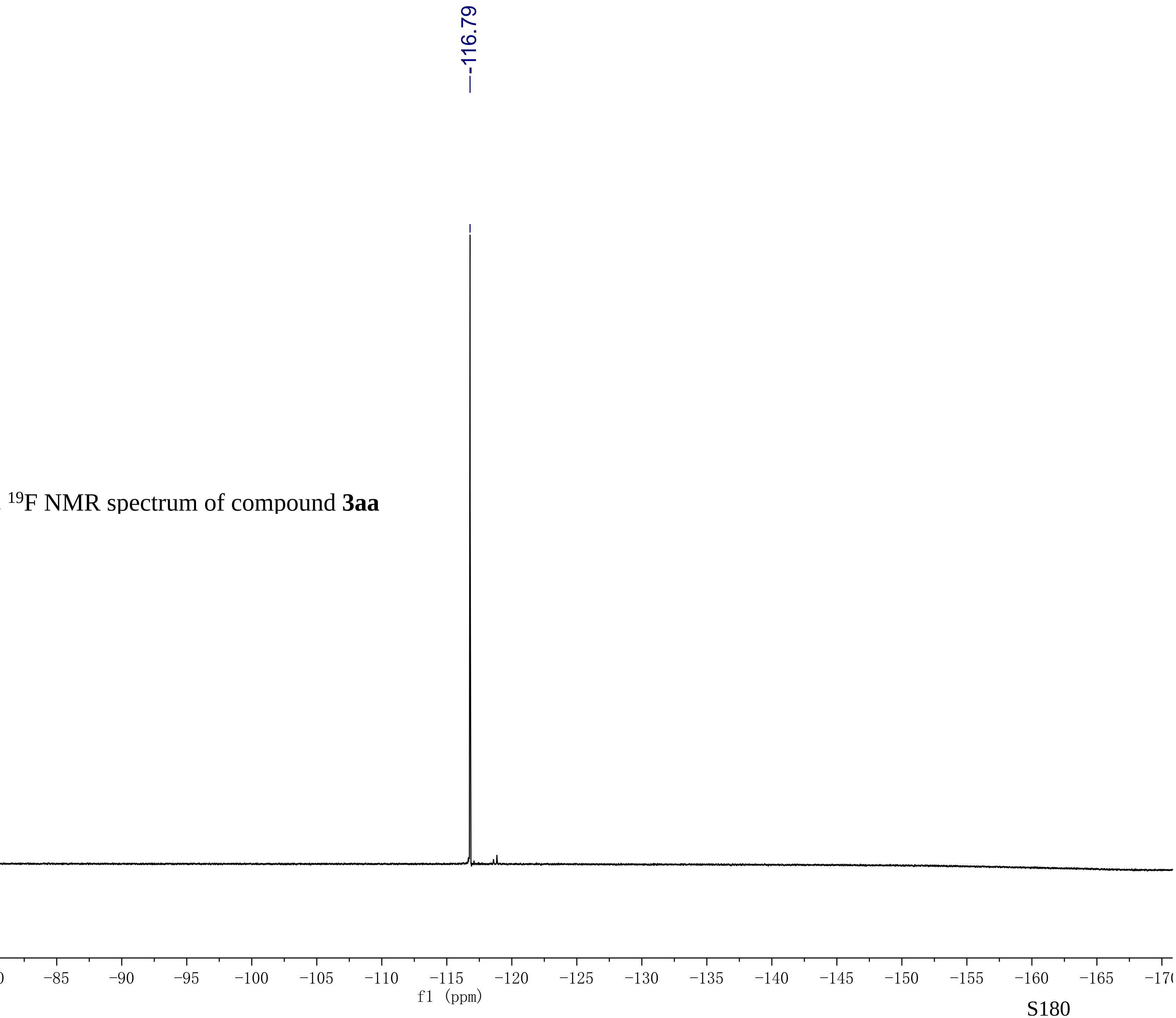

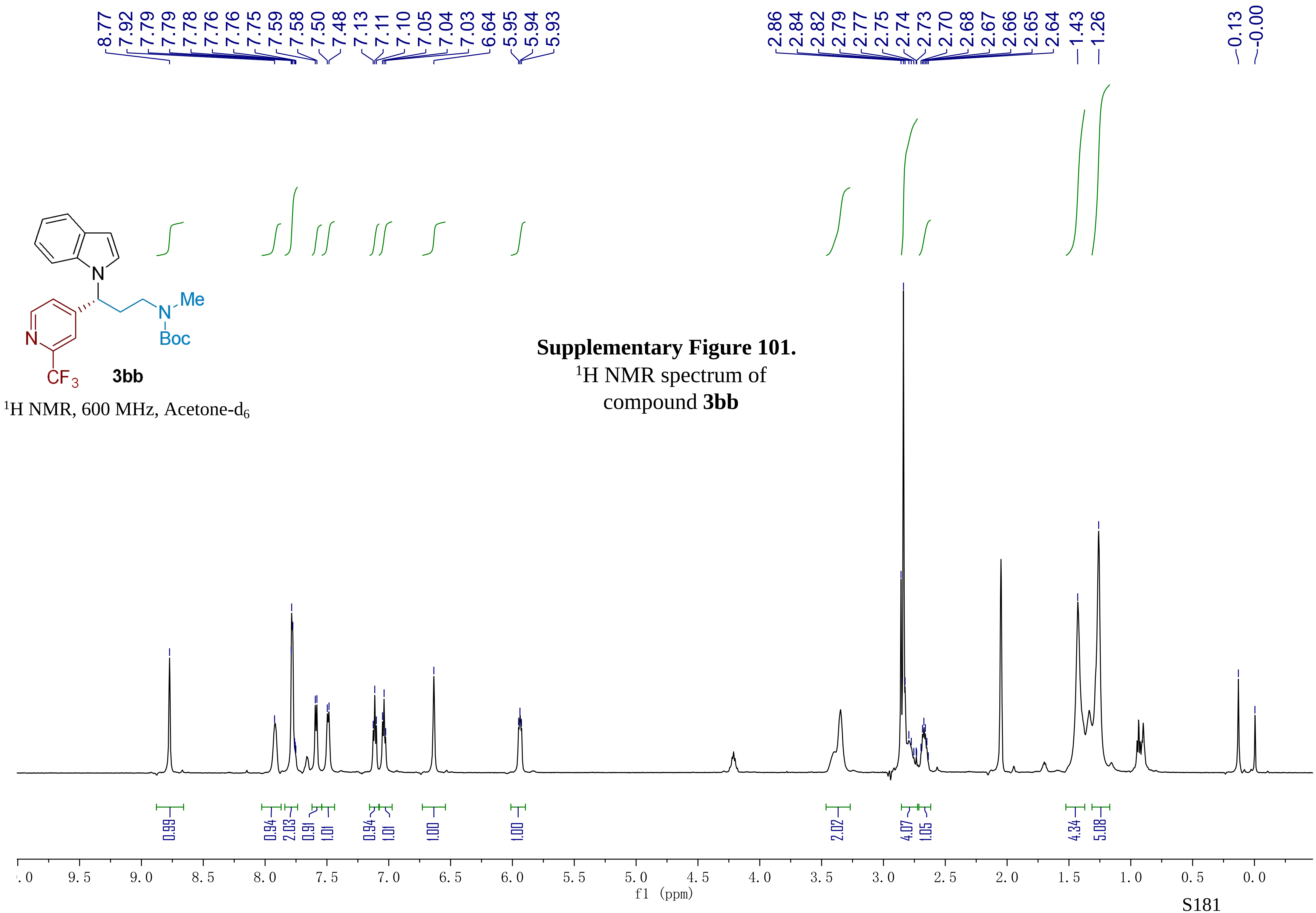

^{1}H NMR, 600 MHz, Acetone-d_6

Supplementary Figure 101.
^{1}H NMR spectrum of compound **3bb**

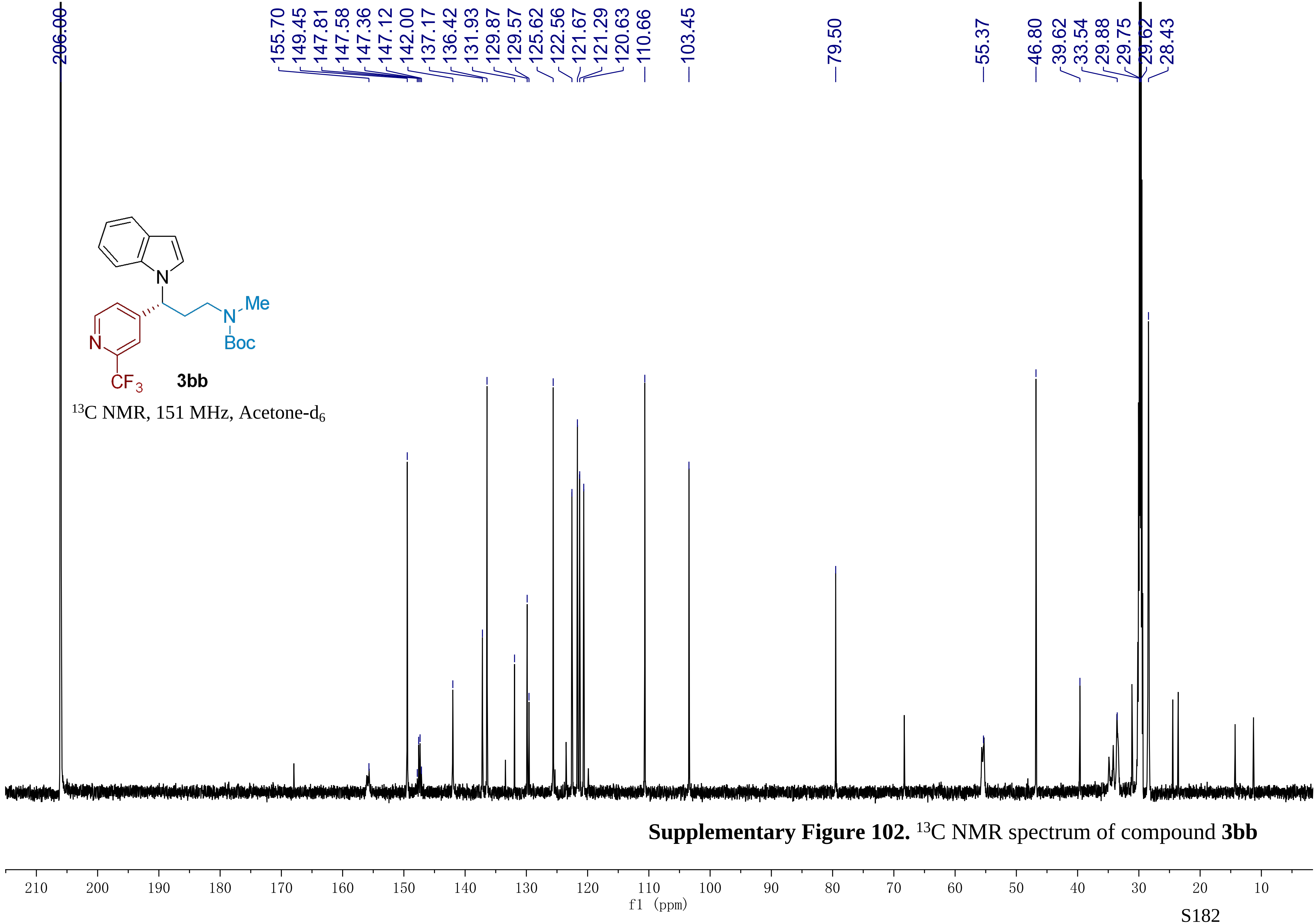

Supplementary Figure 102. ^{13}C NMR spectrum of compound **3bb**

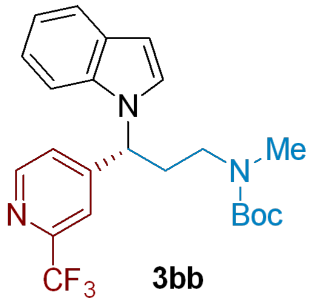

3bb

^{19}F NMR, 565 MHz, Acetone-d_6

Supplementary Figure 103.
^{19}F NMR spectrum of compound **3bb**

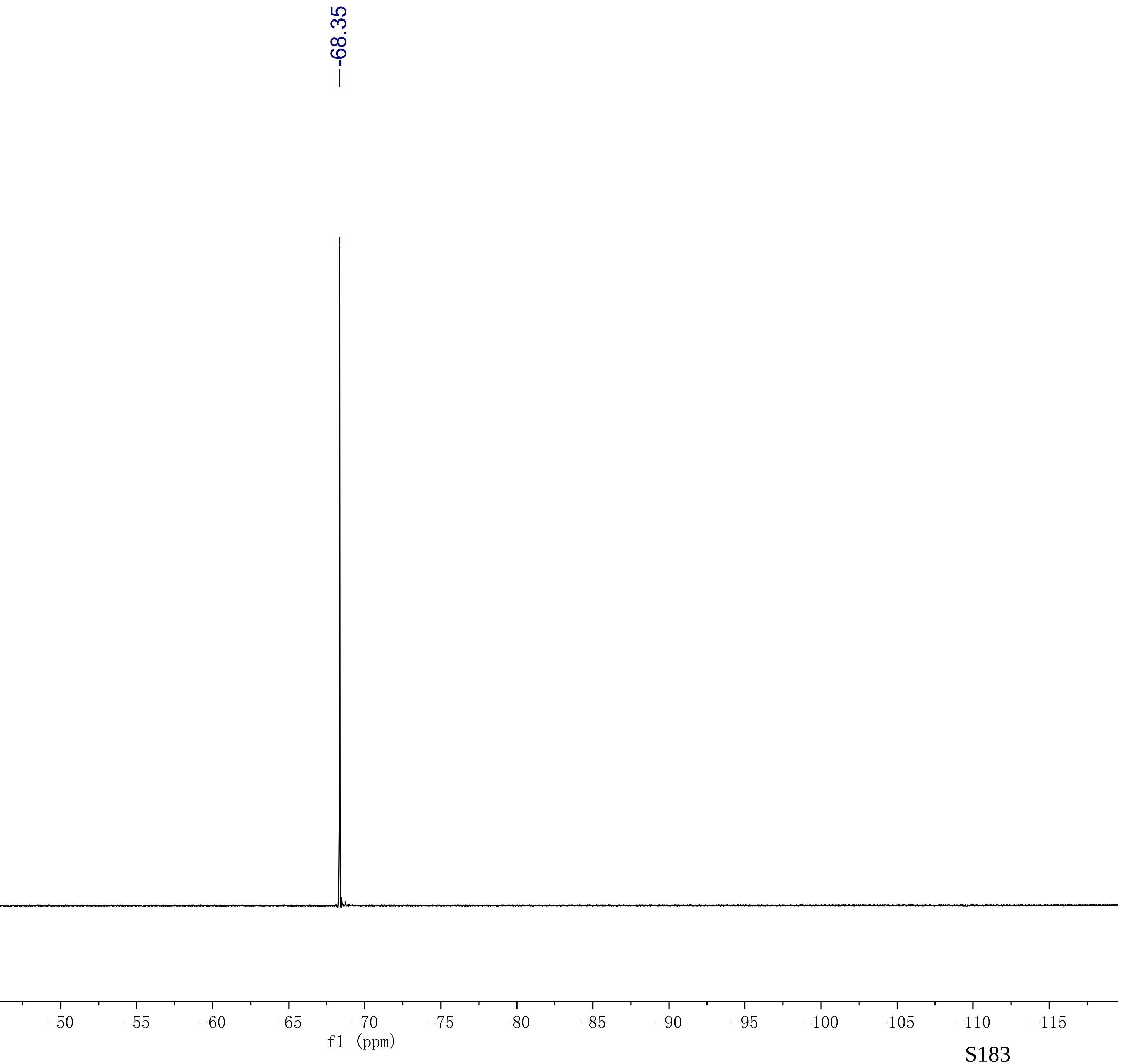

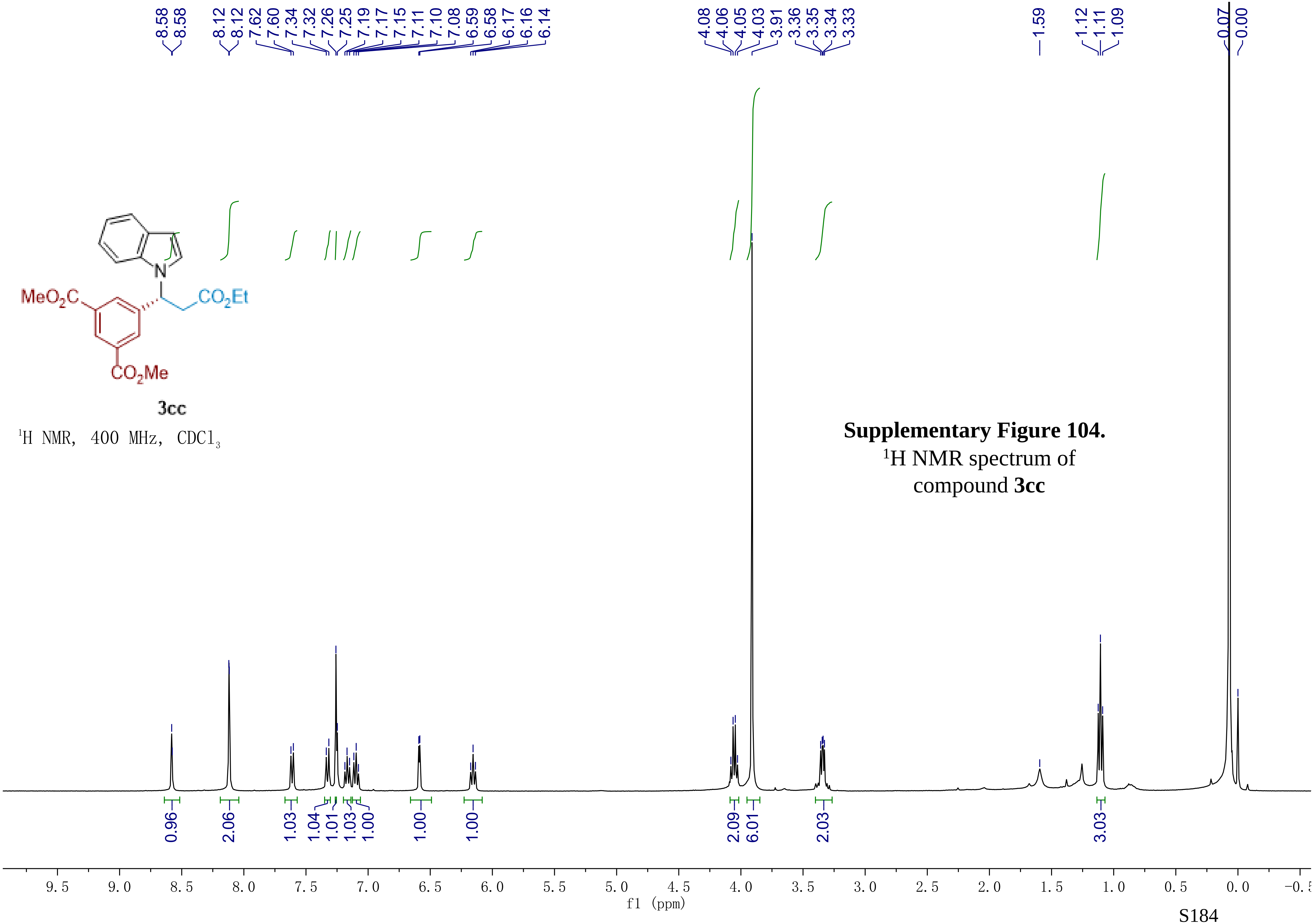

Supplementary Figure 104. ^{1}H NMR spectrum of compound **3cc**

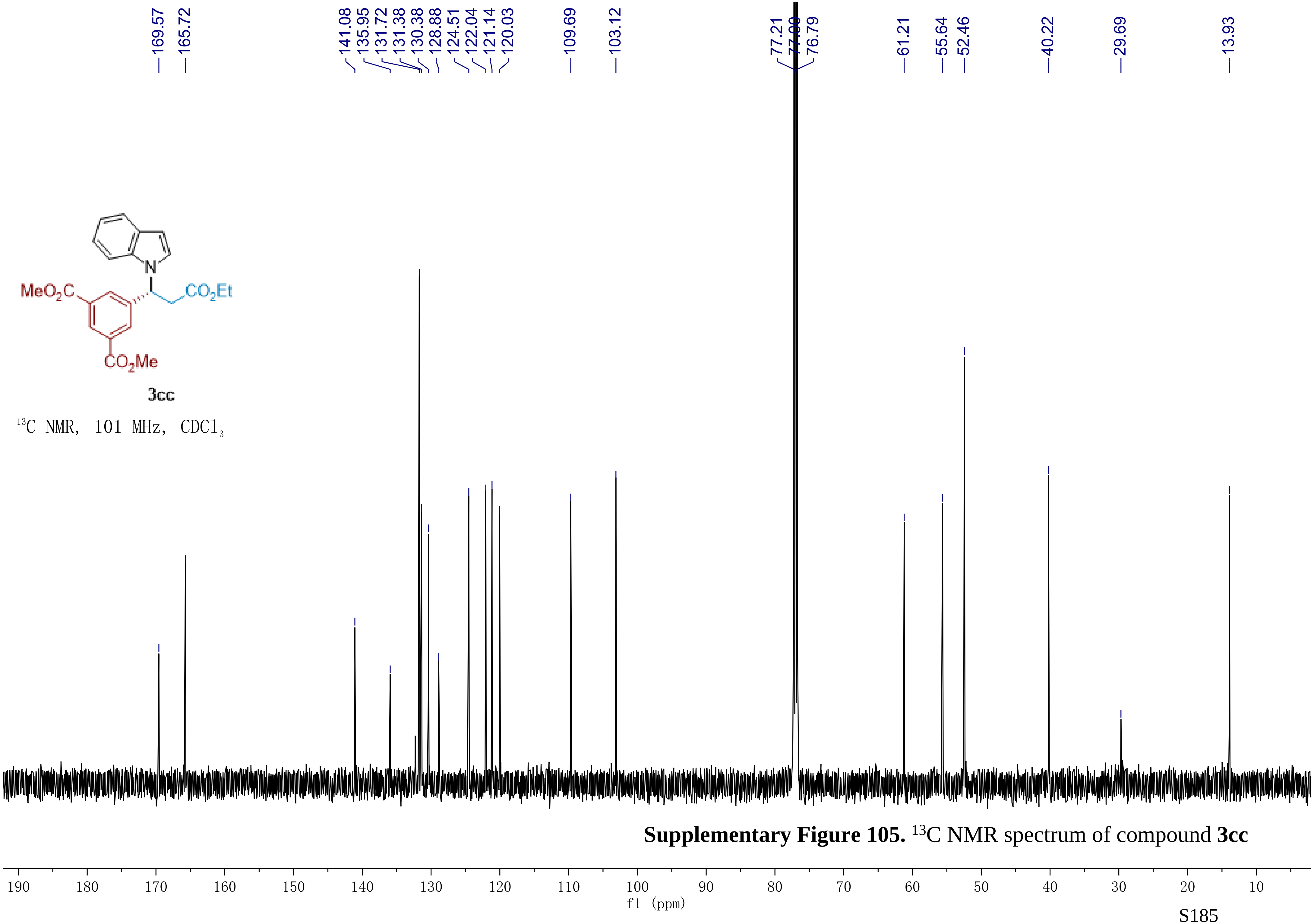

Supplementary Figure 105. ^{13}C NMR spectrum of compound **3cc**

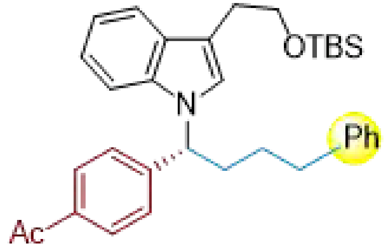

3dd

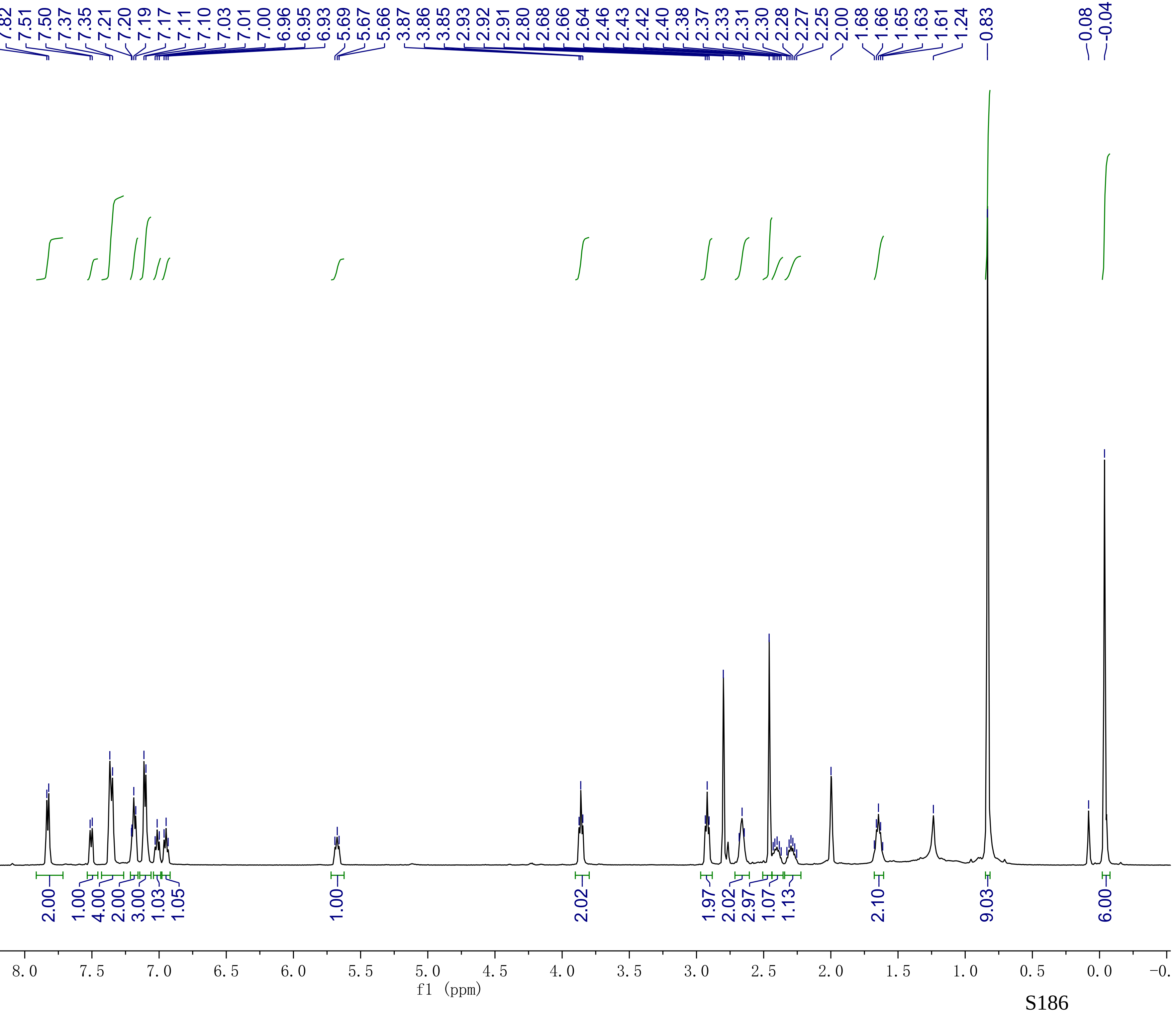

^{1}H NMR, 500 MHz, Acetone-d_6

Supplementary Figure 106. ^{1}H NMR spectrum of compound **3dd**

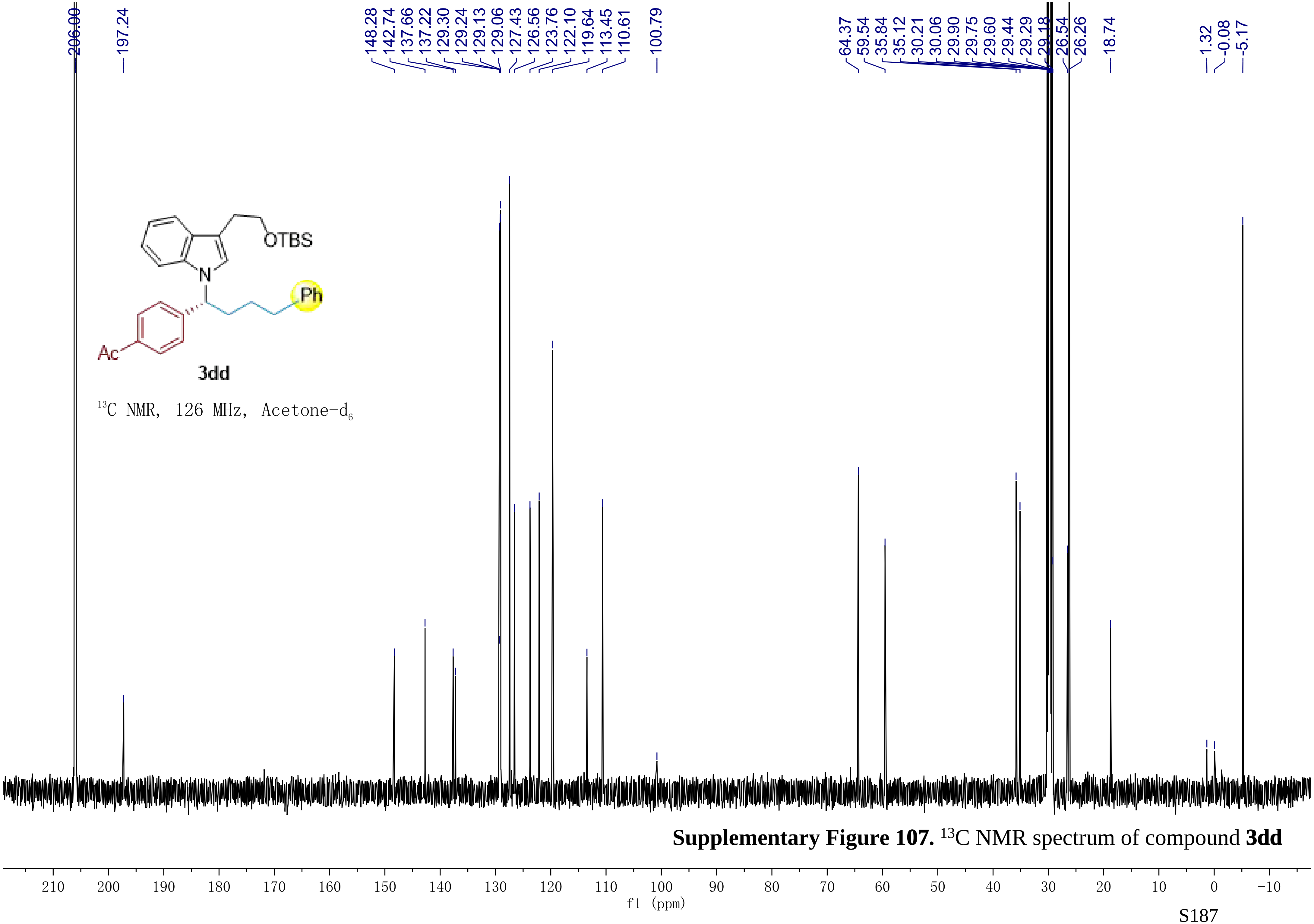

Supplementary Figure 107. ^{13}C NMR spectrum of compound **3dd**

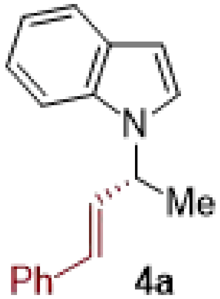

4a

^1H NMR, 500 MHz, $CDCl_3$

Supplementary Figure 108.
^1H NMR spectrum of compound **4a**

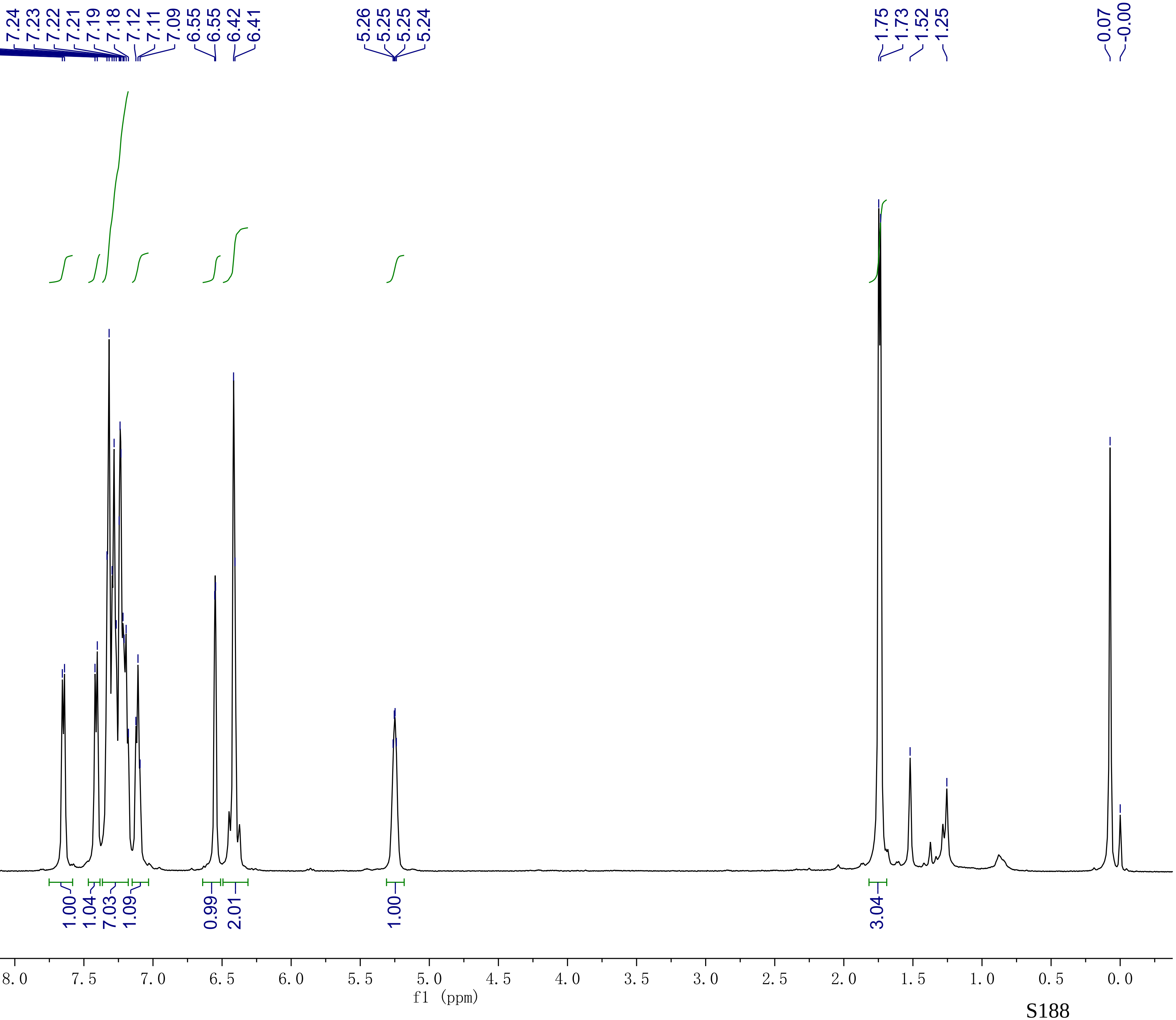

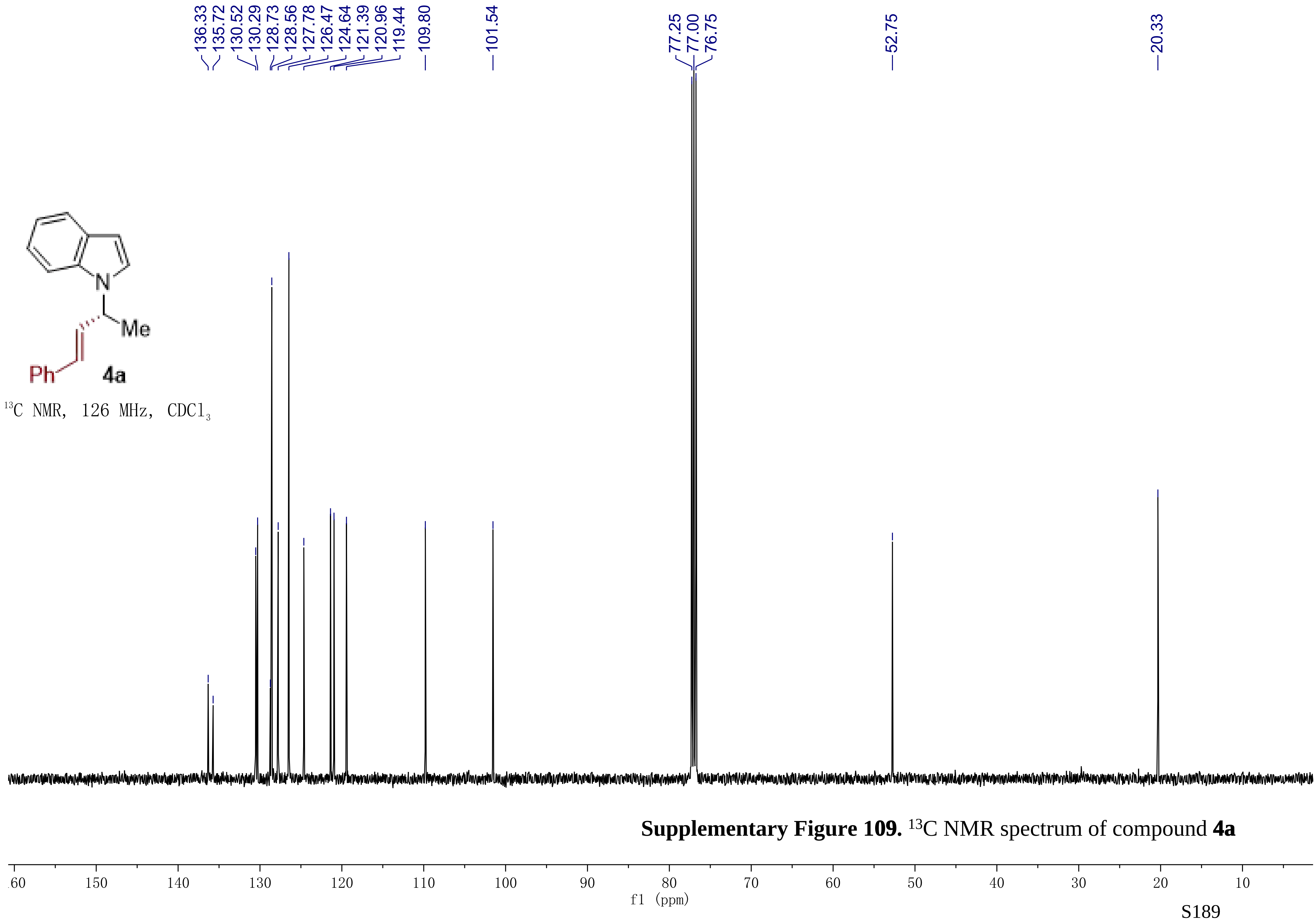

Supplementary Figure 109. ^{13}C NMR spectrum of compound **4a**

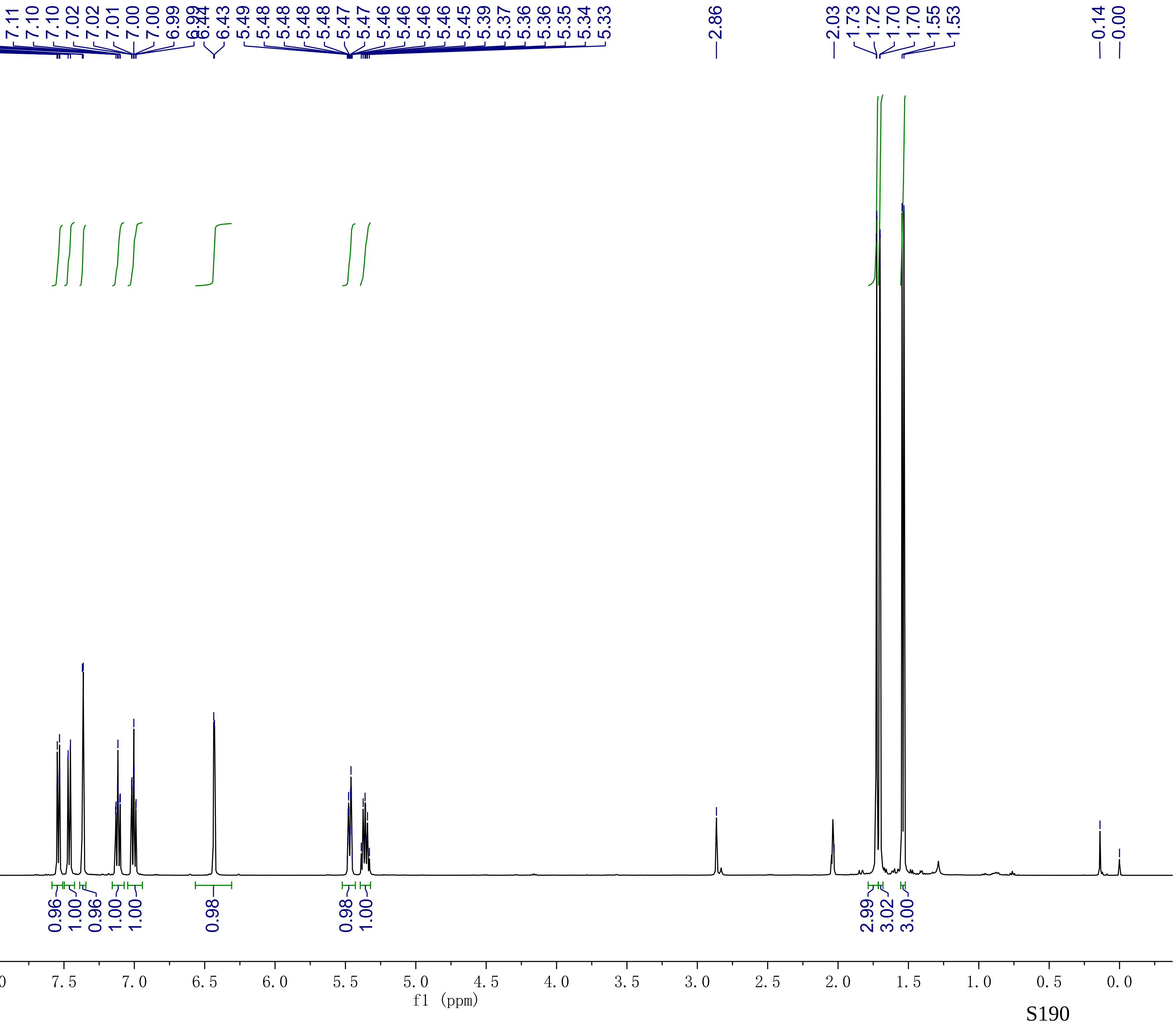

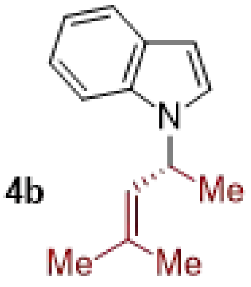

4b

^{1}H NMR, 500 MHz, Acetone-d_6

Supplementary Figure 110. ^{1}H NMR spectrum of compound **4b**

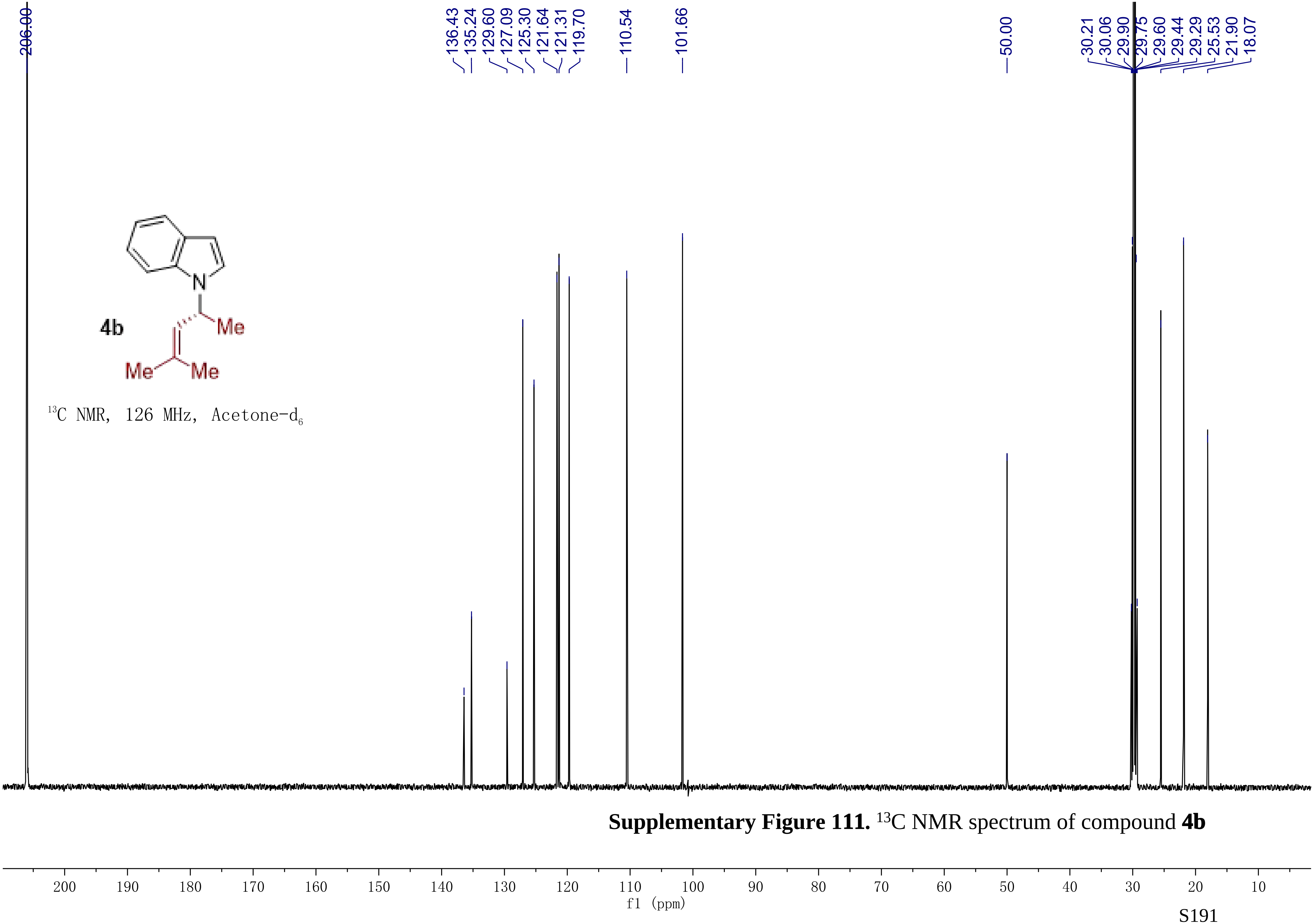

Supplementary Figure 111. ^{13}C NMR spectrum of compound **4b**

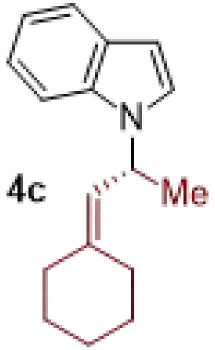

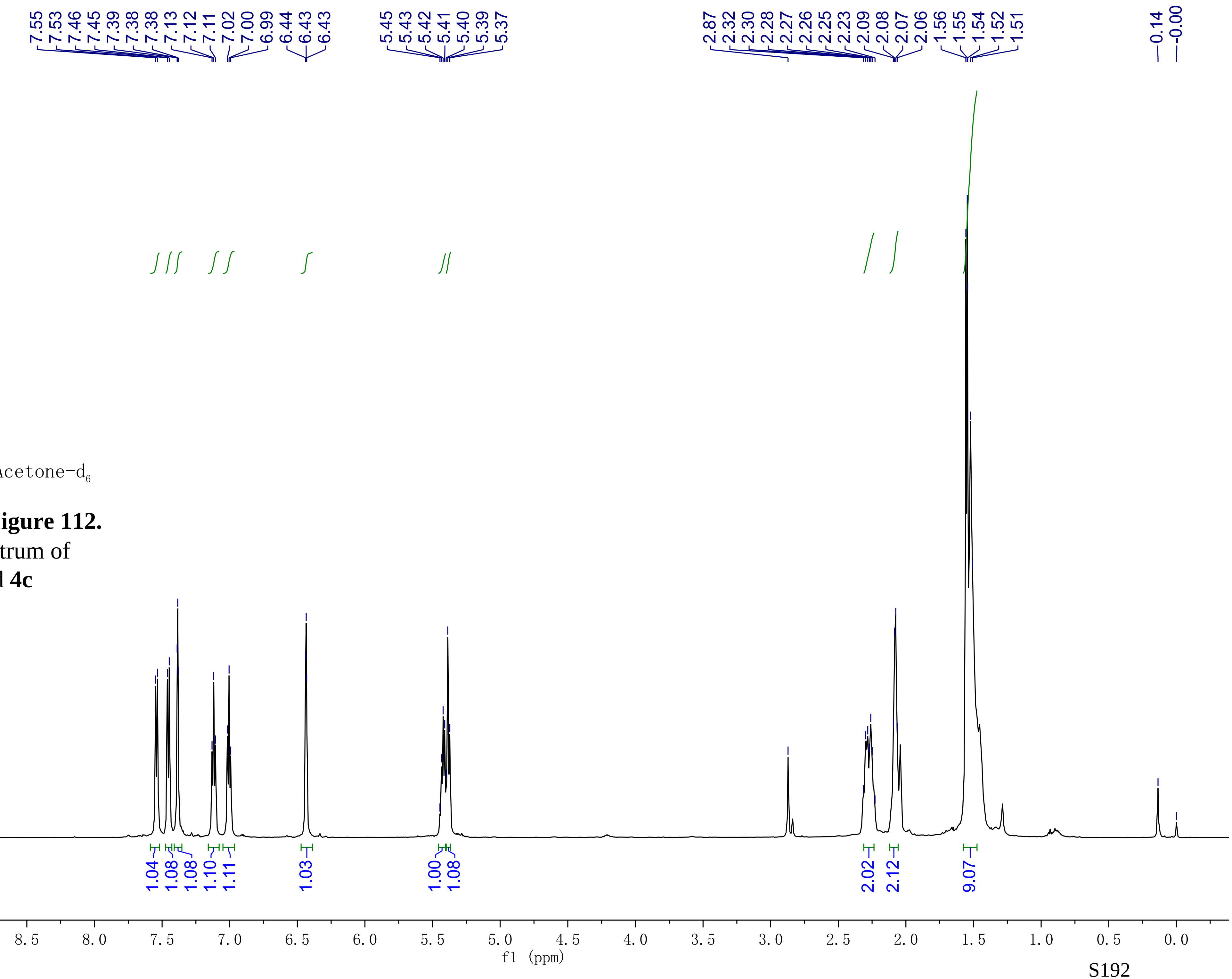

^{1}H NMR, 600 MHz, Acetone-d_6

Supplementary Figure 112. ^{1}H NMR spectrum of compound **4c**

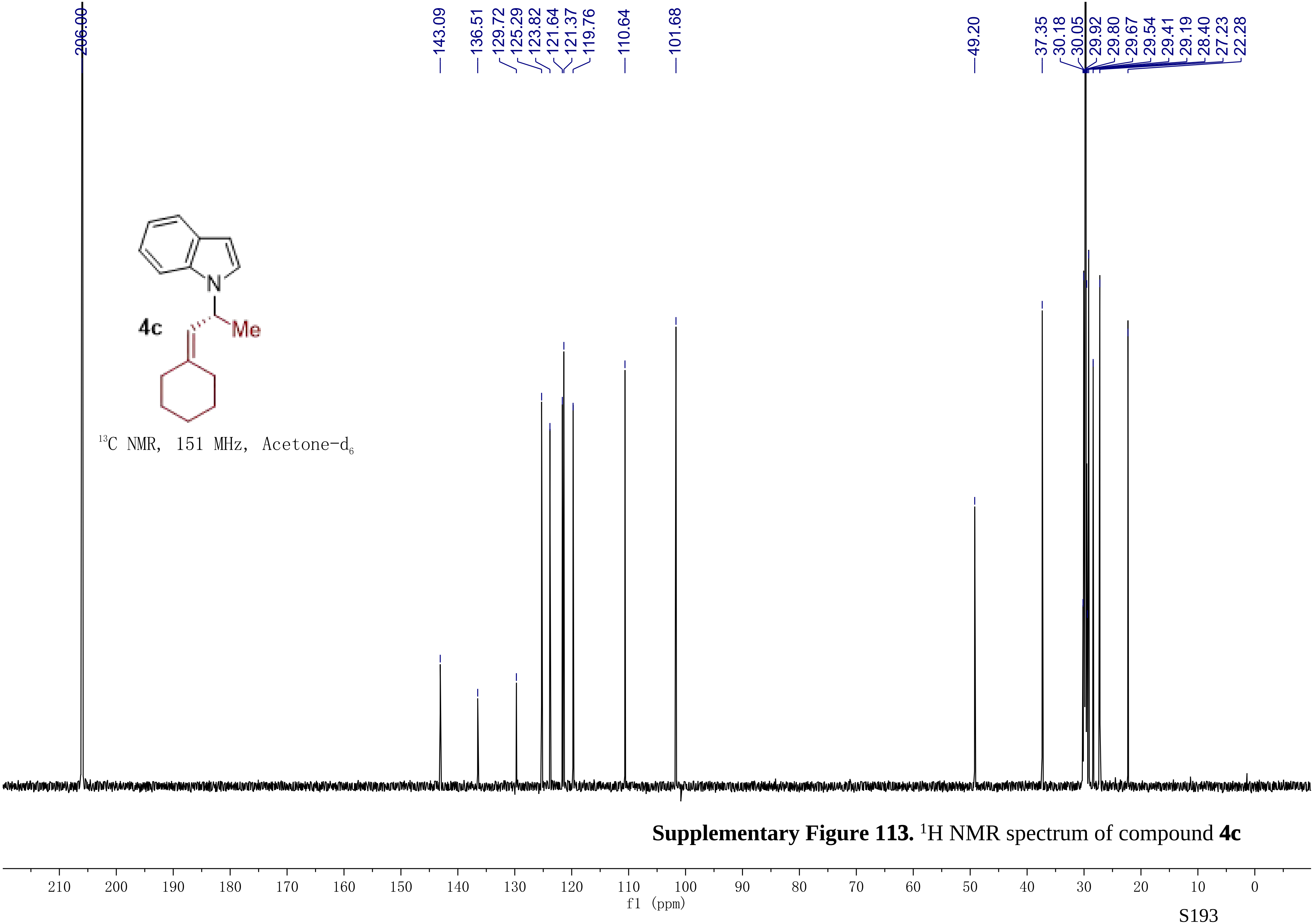

Supplementary Figure 113. ^{1}H NMR spectrum of compound **4c**

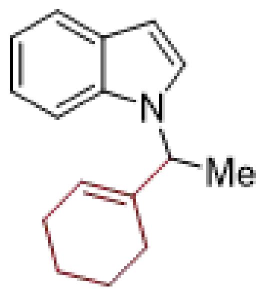

4d

^{1}H NMR, 600 MHz, Acetone-d_6

Supplementary Figure 114. ^{1}H NMR spectrum of compound **4d**

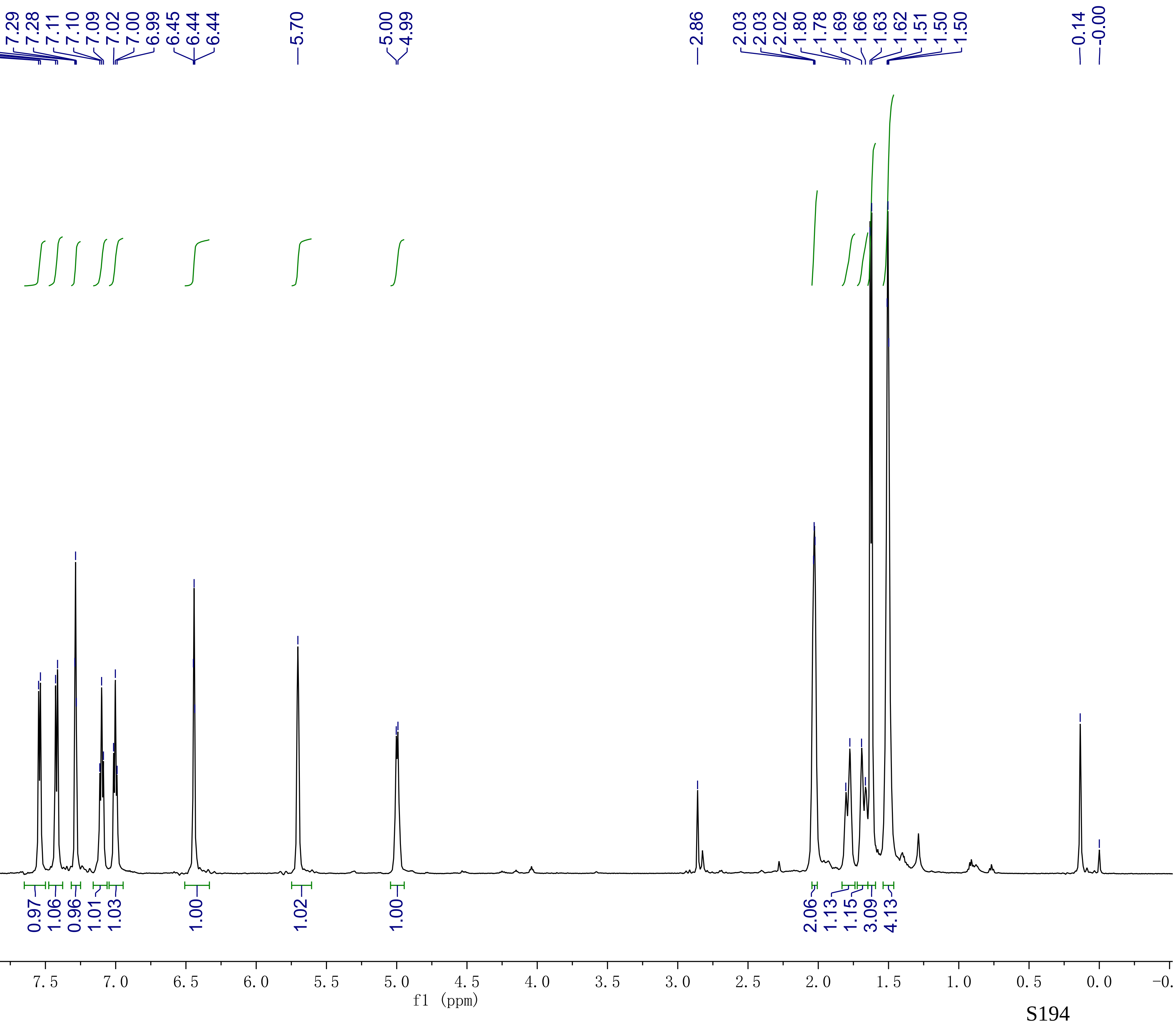

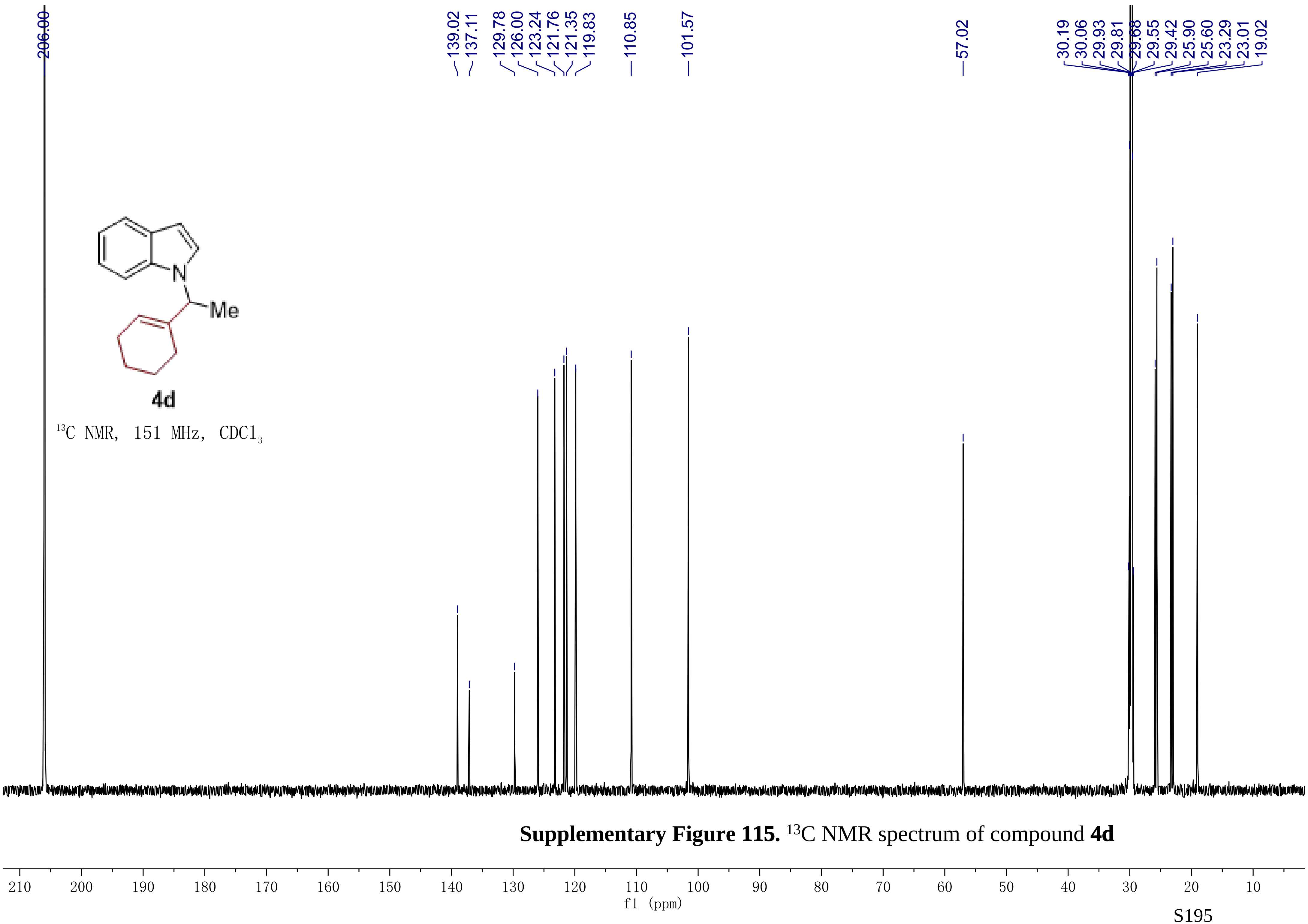

Supplementary Figure 115. ^{13}C NMR spectrum of compound **4d**

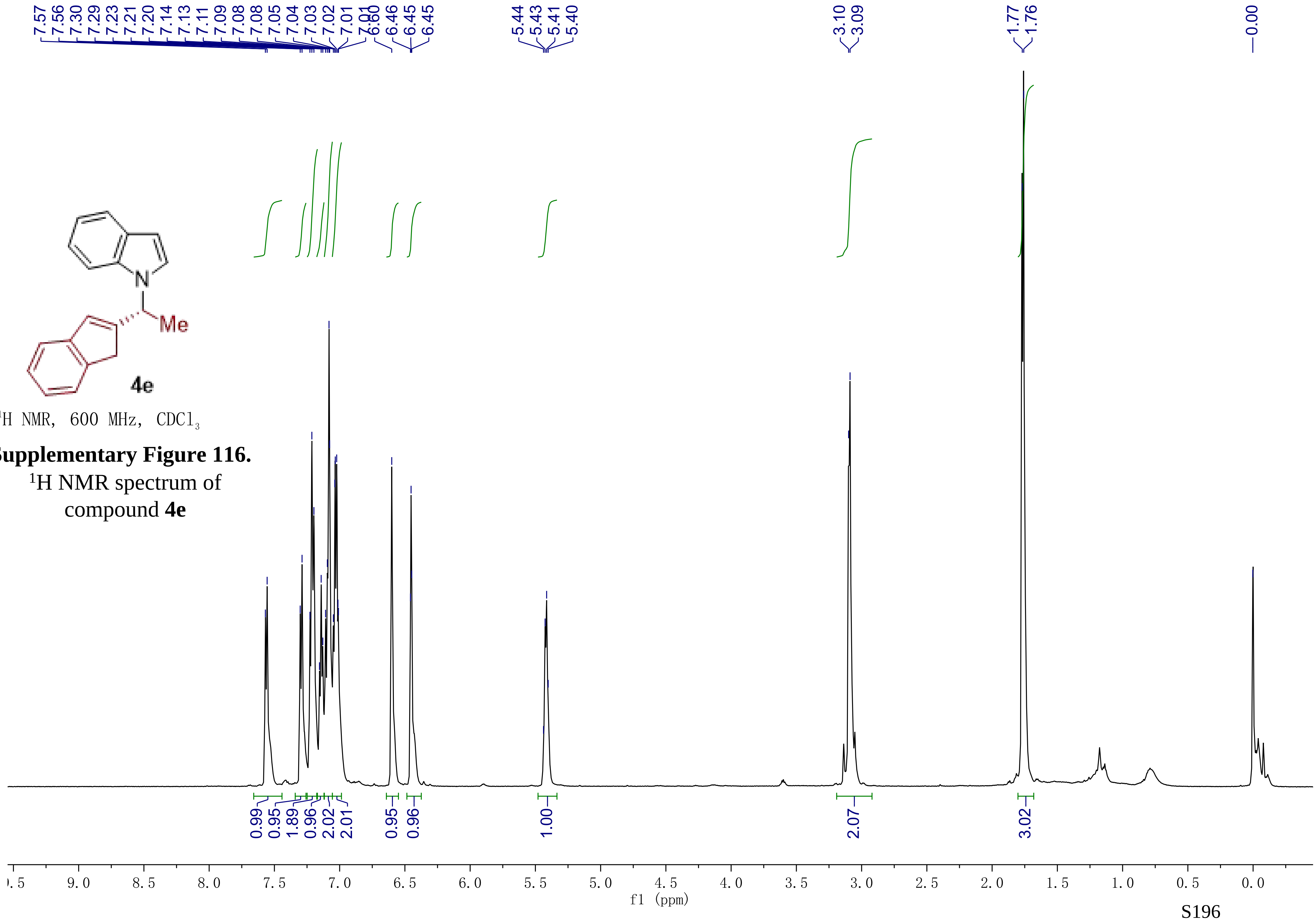

^{1}H NMR, 600 MHz, $CDCl_3$

Supplementary Figure 116. ^{1}H NMR spectrum of compound **4e**

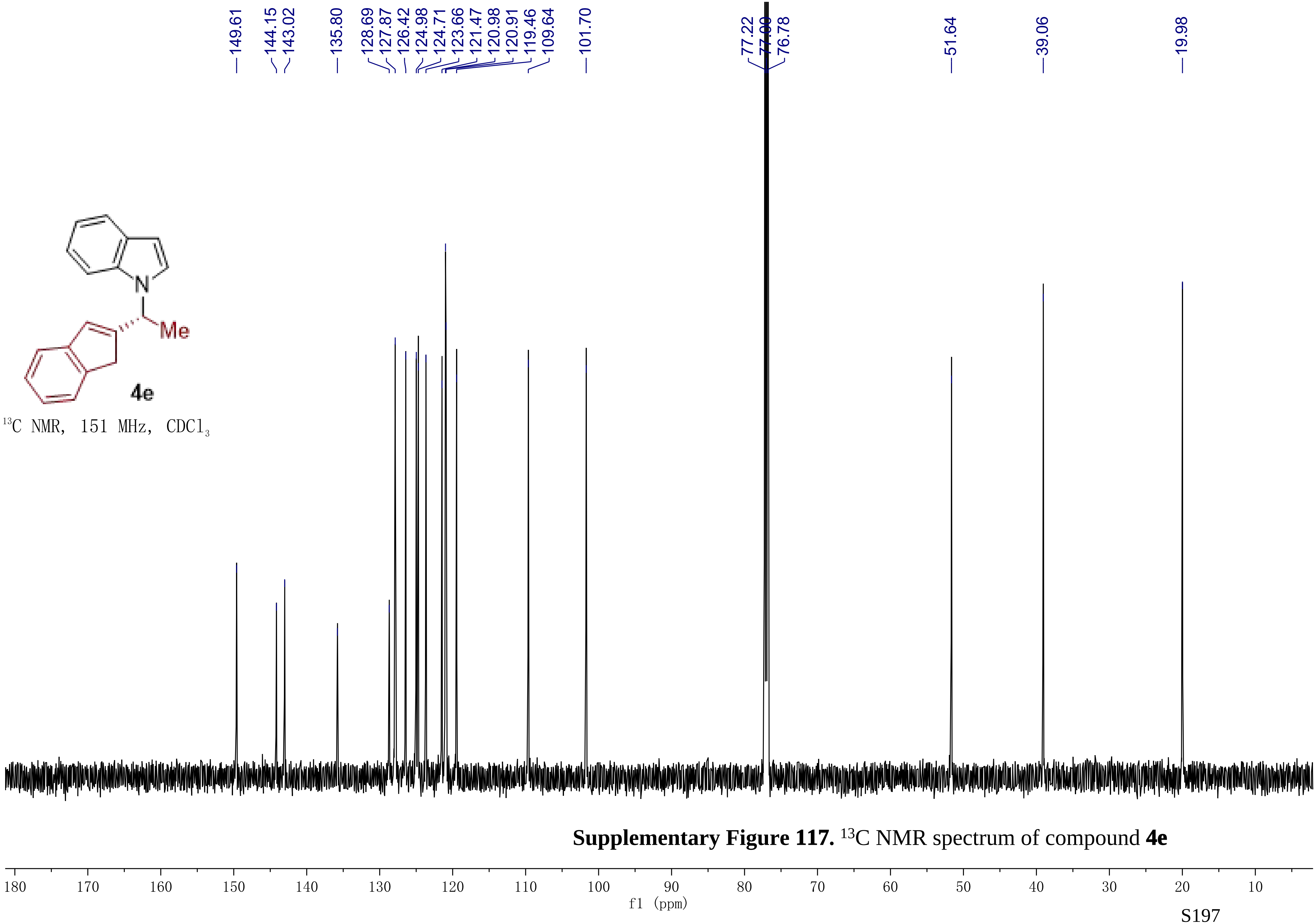

Supplementary Figure 117. ^{13}C NMR spectrum of compound **4e**

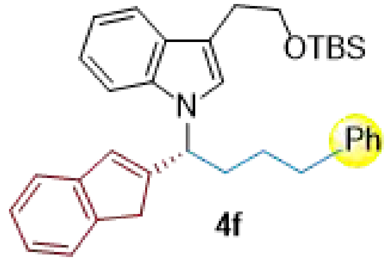

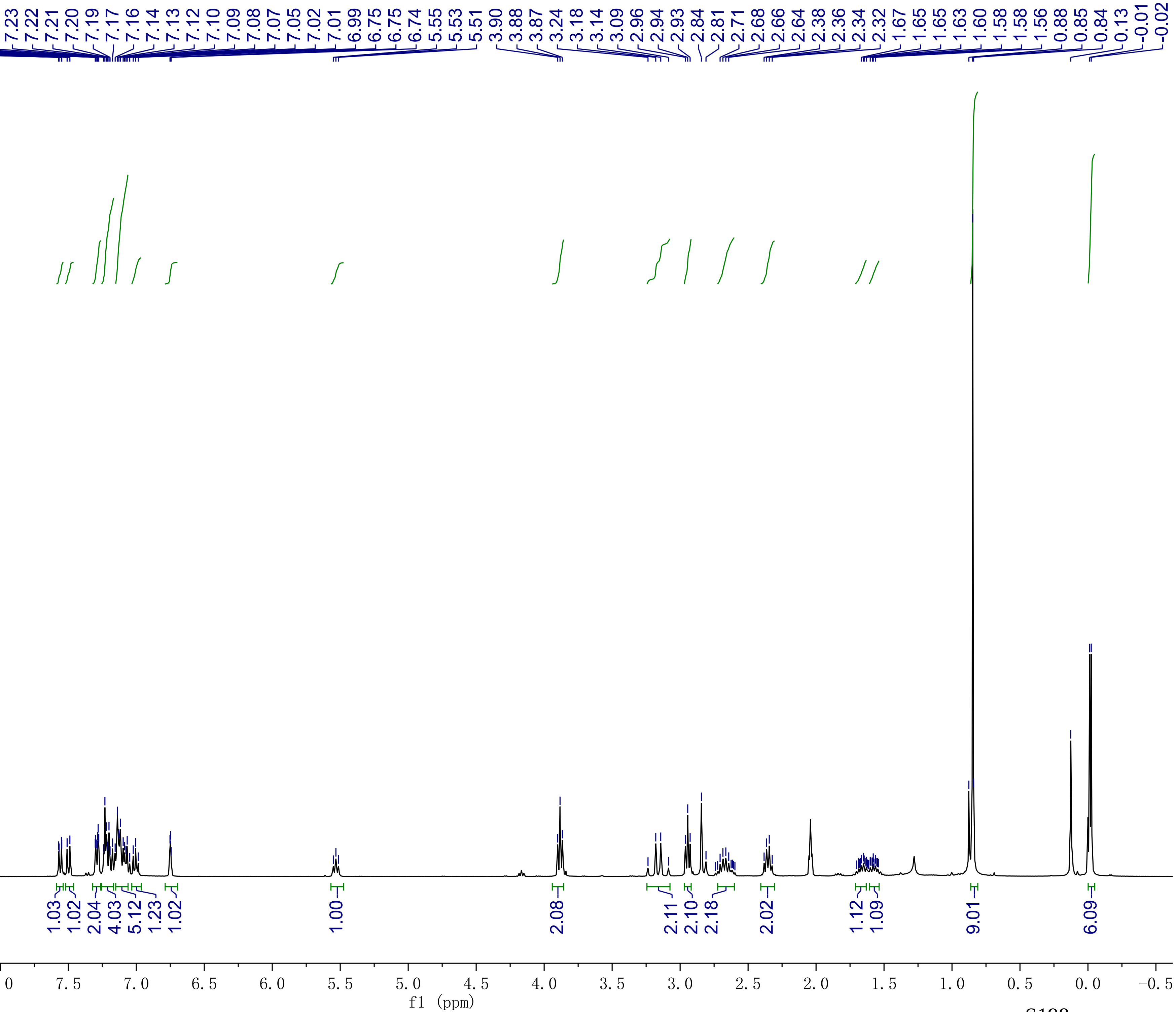

^{1}H NMR, 400 MHz, Acetone-d_6

Supplementary Figure 118. ^{1}H NMR spectrum of compound **4f**

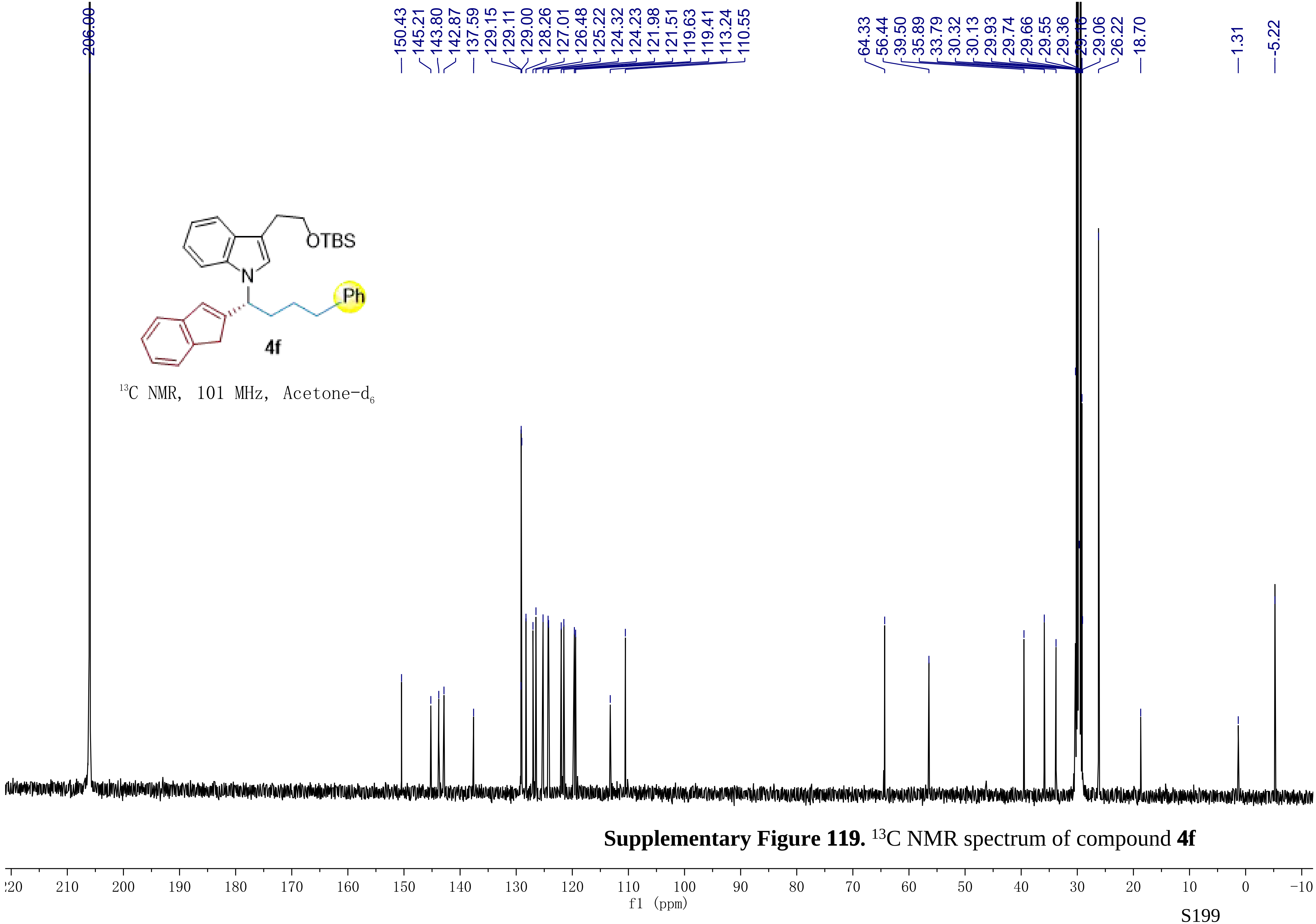

Supplementary Figure 119. ^{13}C NMR spectrum of compound **4f**

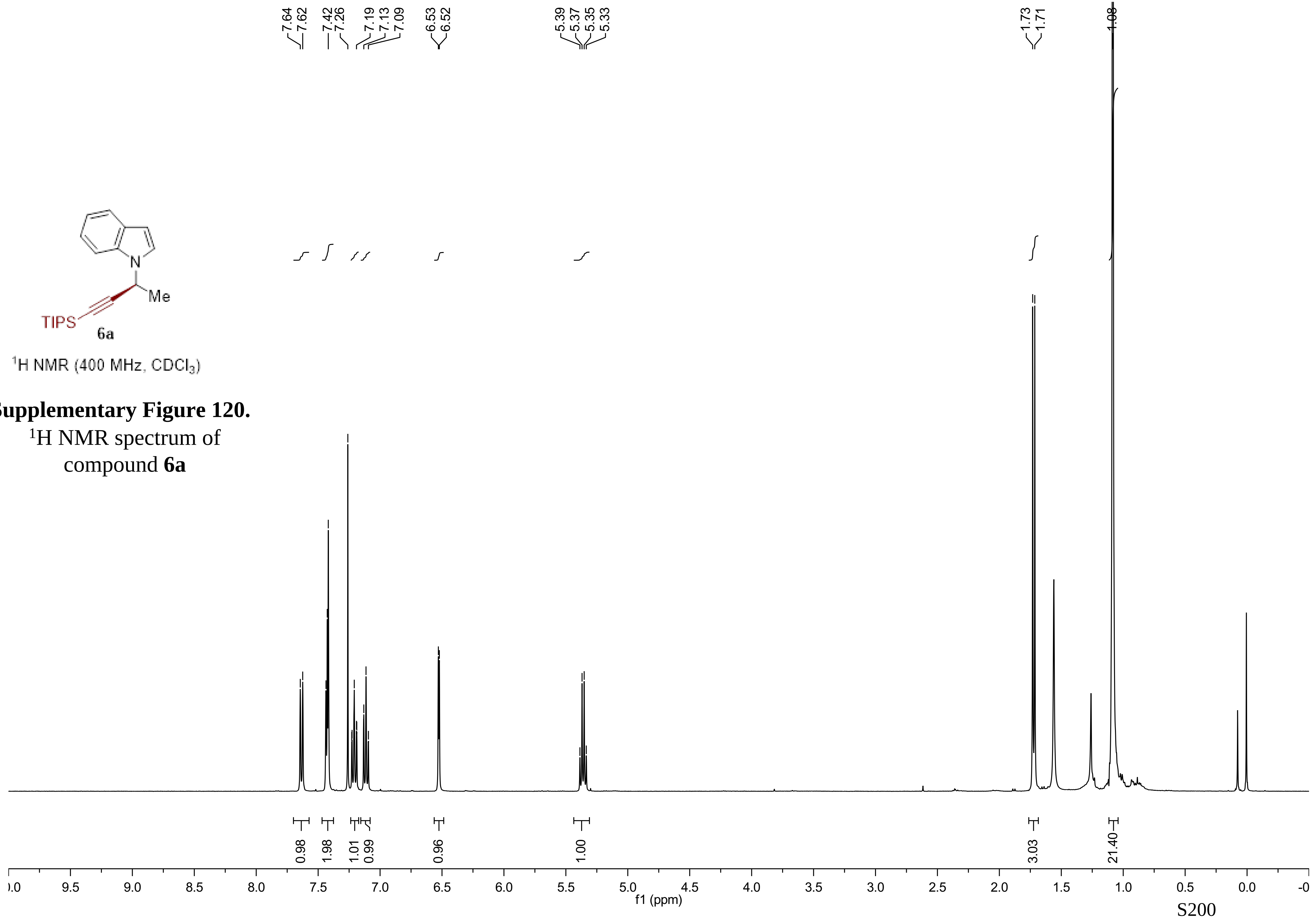

Supplementary Figure 120.
^{1}H NMR spectrum of compound **6a**

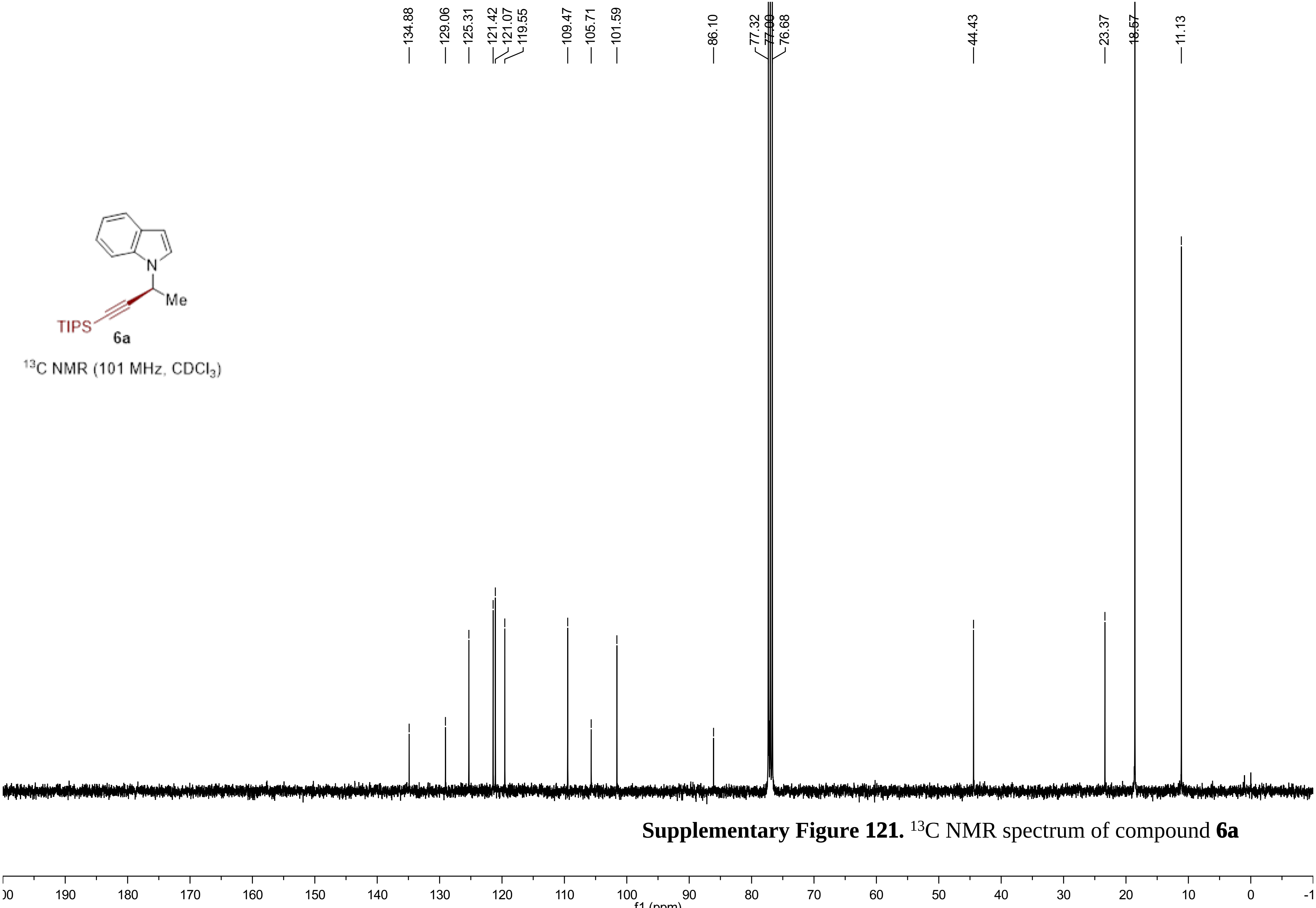

Supplementary Figure 121. ^{13}C NMR spectrum of compound **6a**

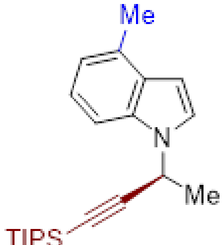

6b

^{1}H NMR (500 MHz, $CDCl_3$)

Supplementary Figure 122.
^{1}H NMR spectrum of compound **6b**

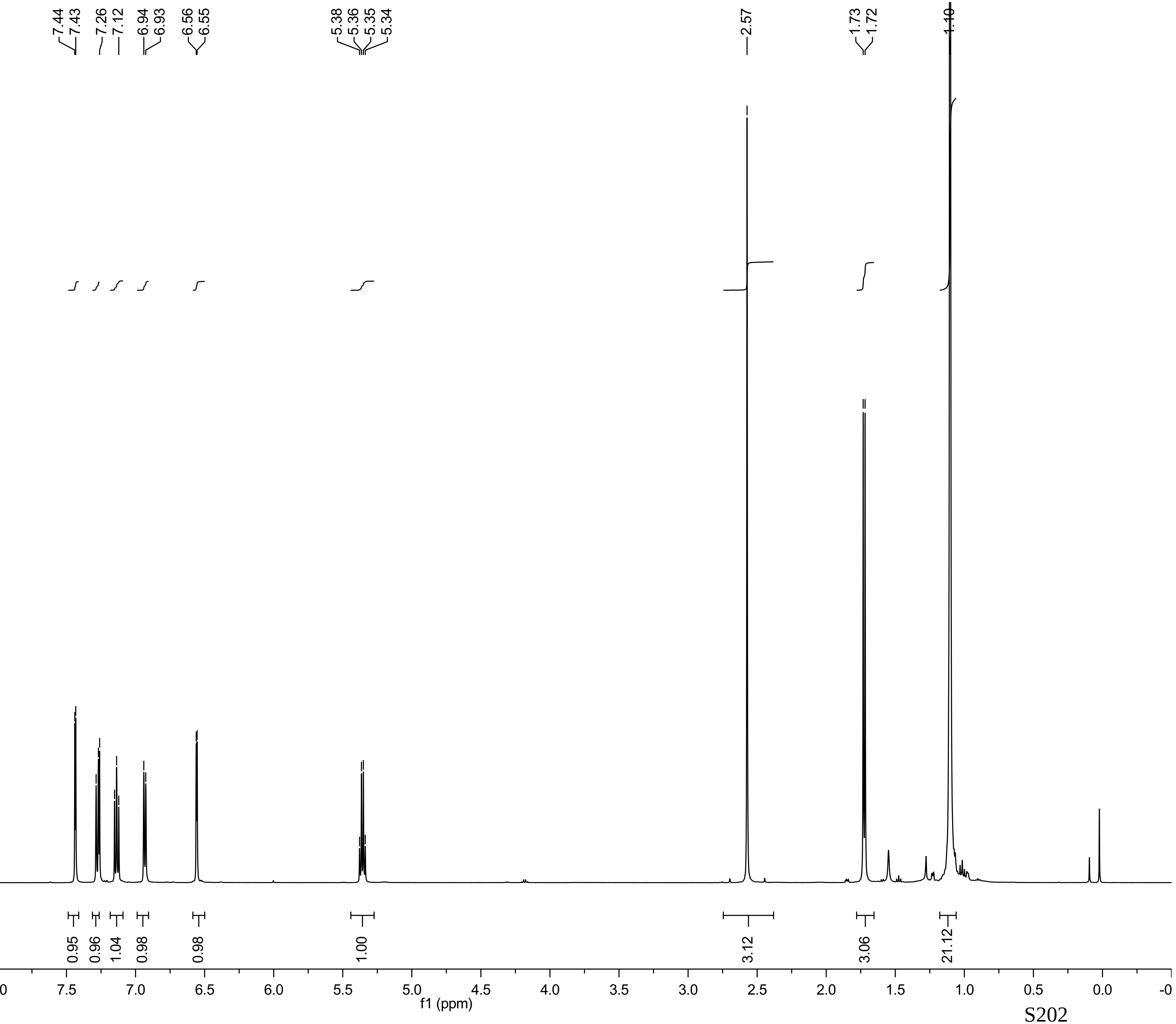

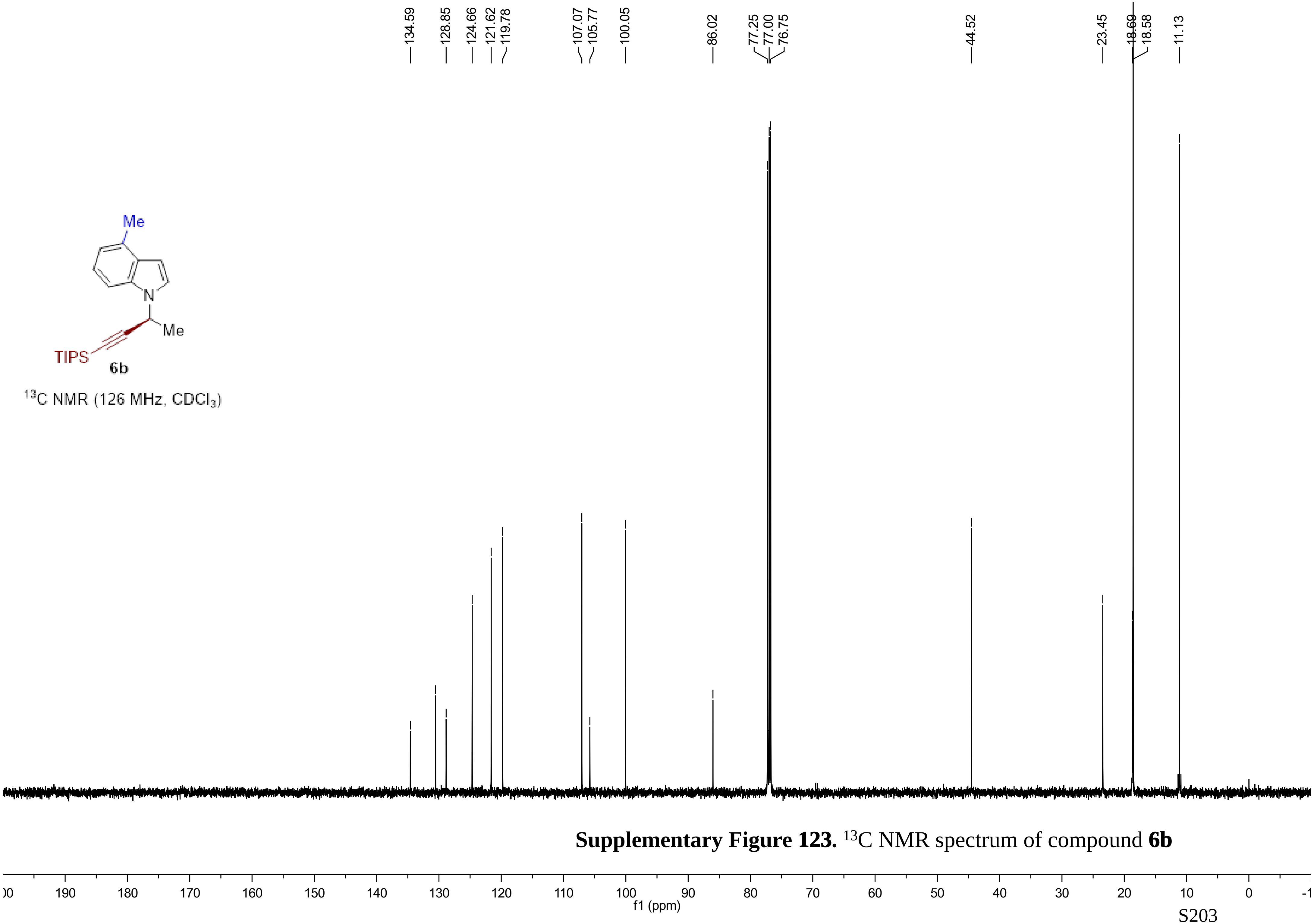

Supplementary Figure 123. ^{13}C NMR spectrum of compound **6b**

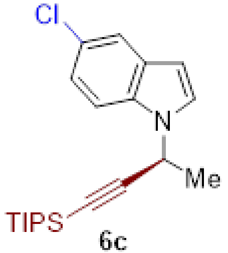

6c

^{1}H NMR (600 MHz, $CDCl_3$)

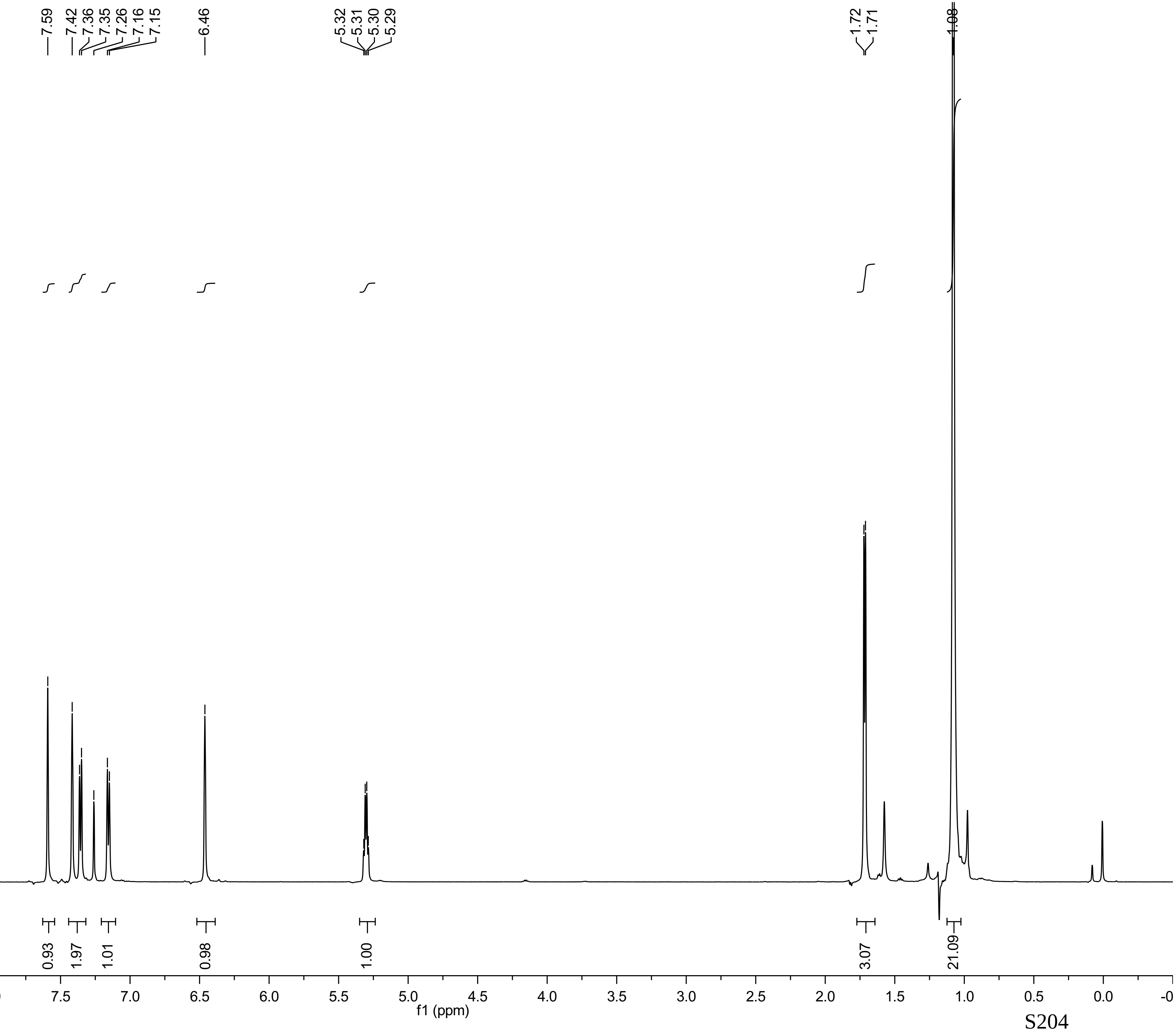

Supplementary Figure 124.
^{1}H NMR spectrum of compound **6c**

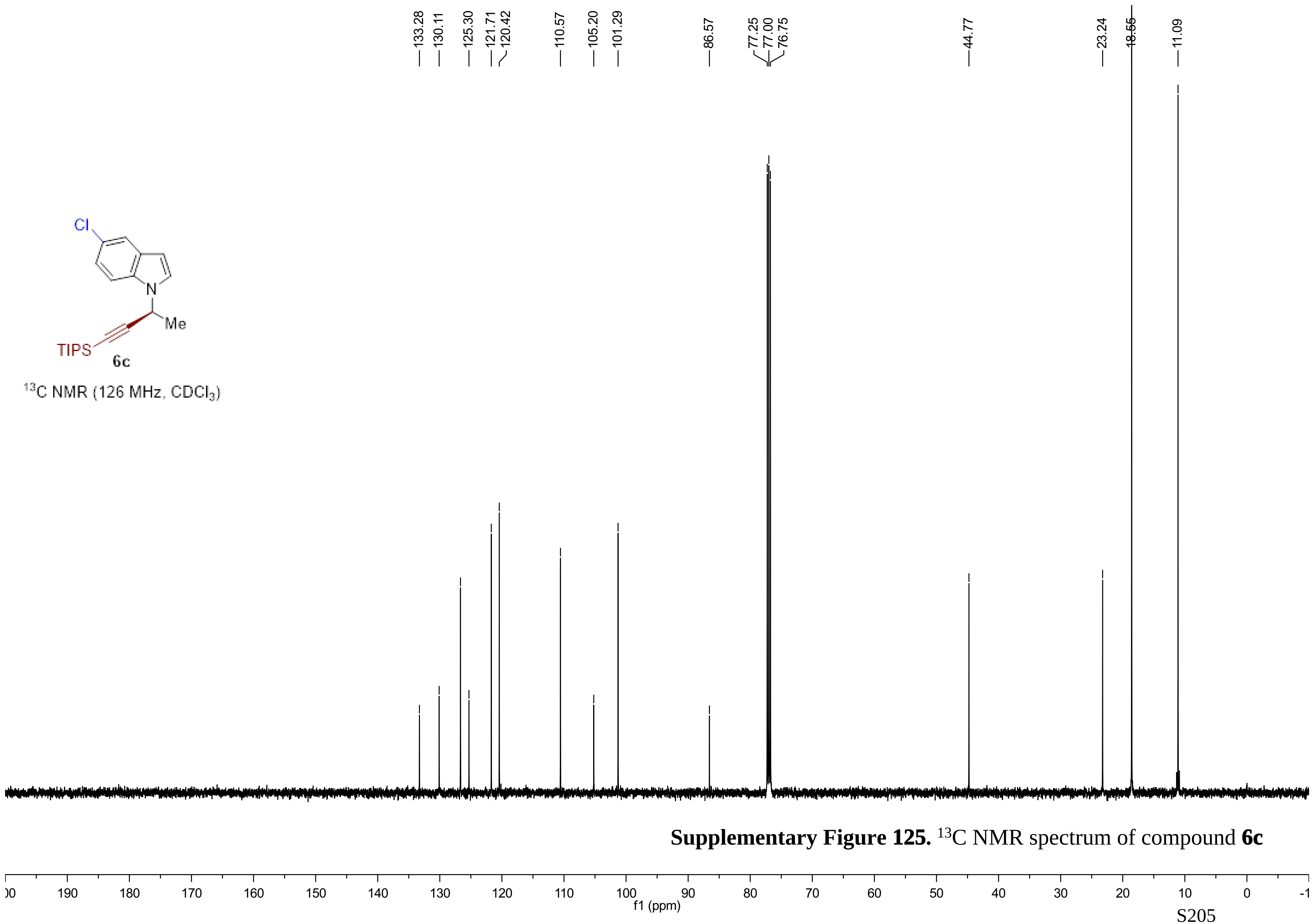

Supplementary Figure 125. ^{13}C NMR spectrum of compound **6c**

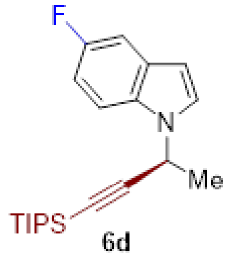

6d

^{1}H NMR (400 MHz, $CDCl_3$)

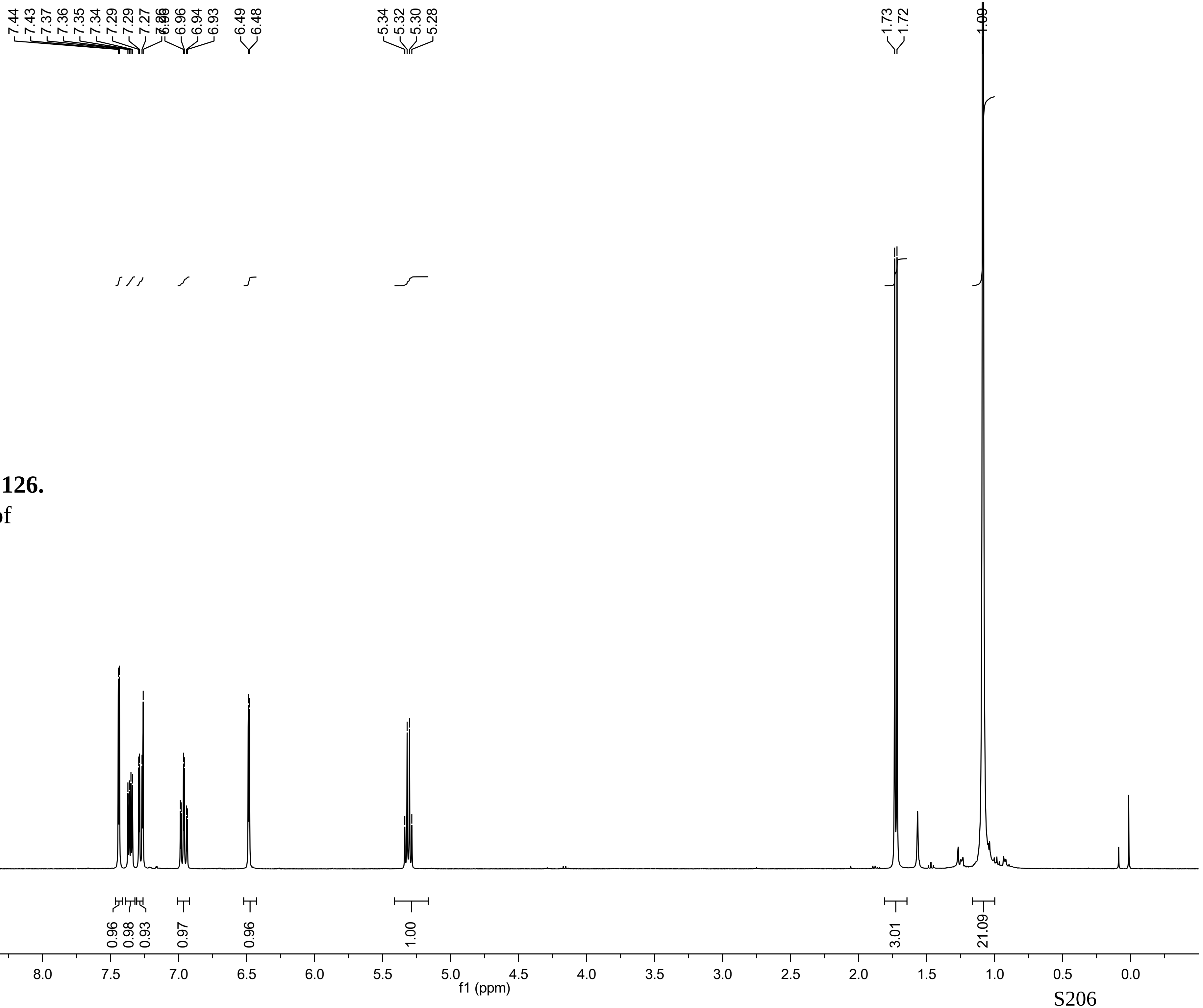

Supplementary Figure 126. ^{1}H NMR spectrum of compound **6d**

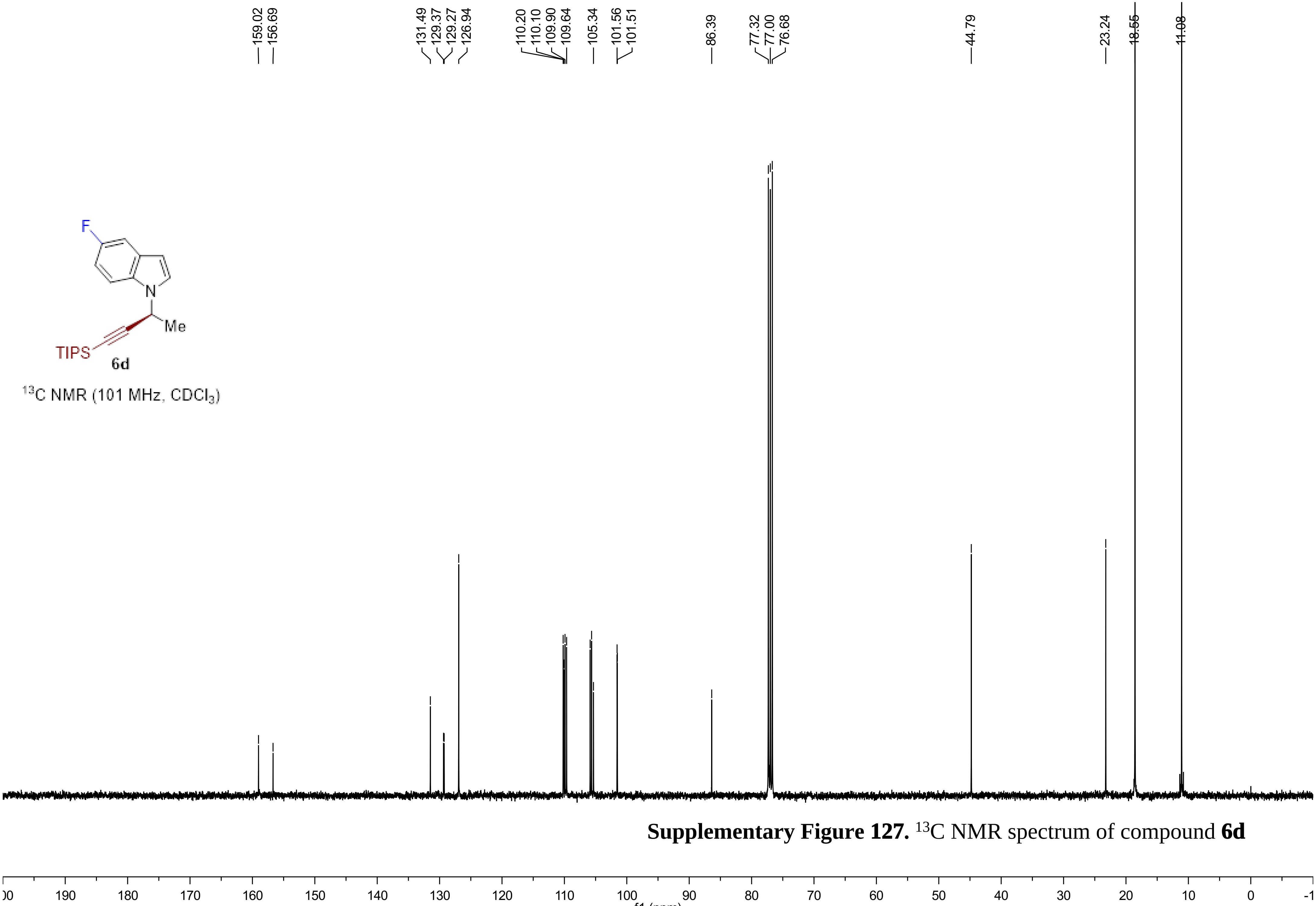

Supplementary Figure 127. ^{13}C NMR spectrum of compound **6d**

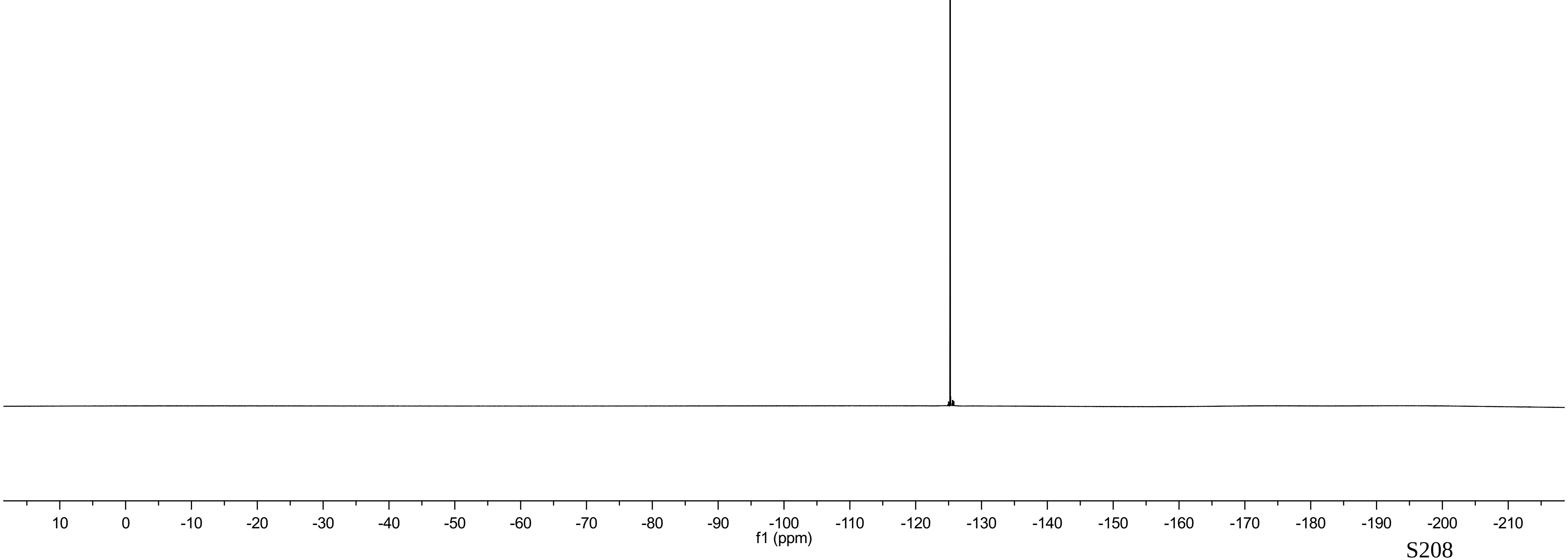

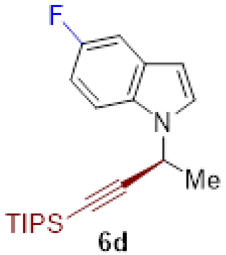

6d

^{19}F NMR (377 MHz, $CDCl_3$)

Supplementary Figure 128. ^{19}F NMR spectrum of compound **6d**

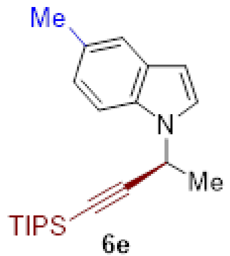

6e

^{1}H NMR (400 MHz, $CDCl_3$)

Supplementary Figure 129.
^{1}H NMR spectrum of compound **6e**

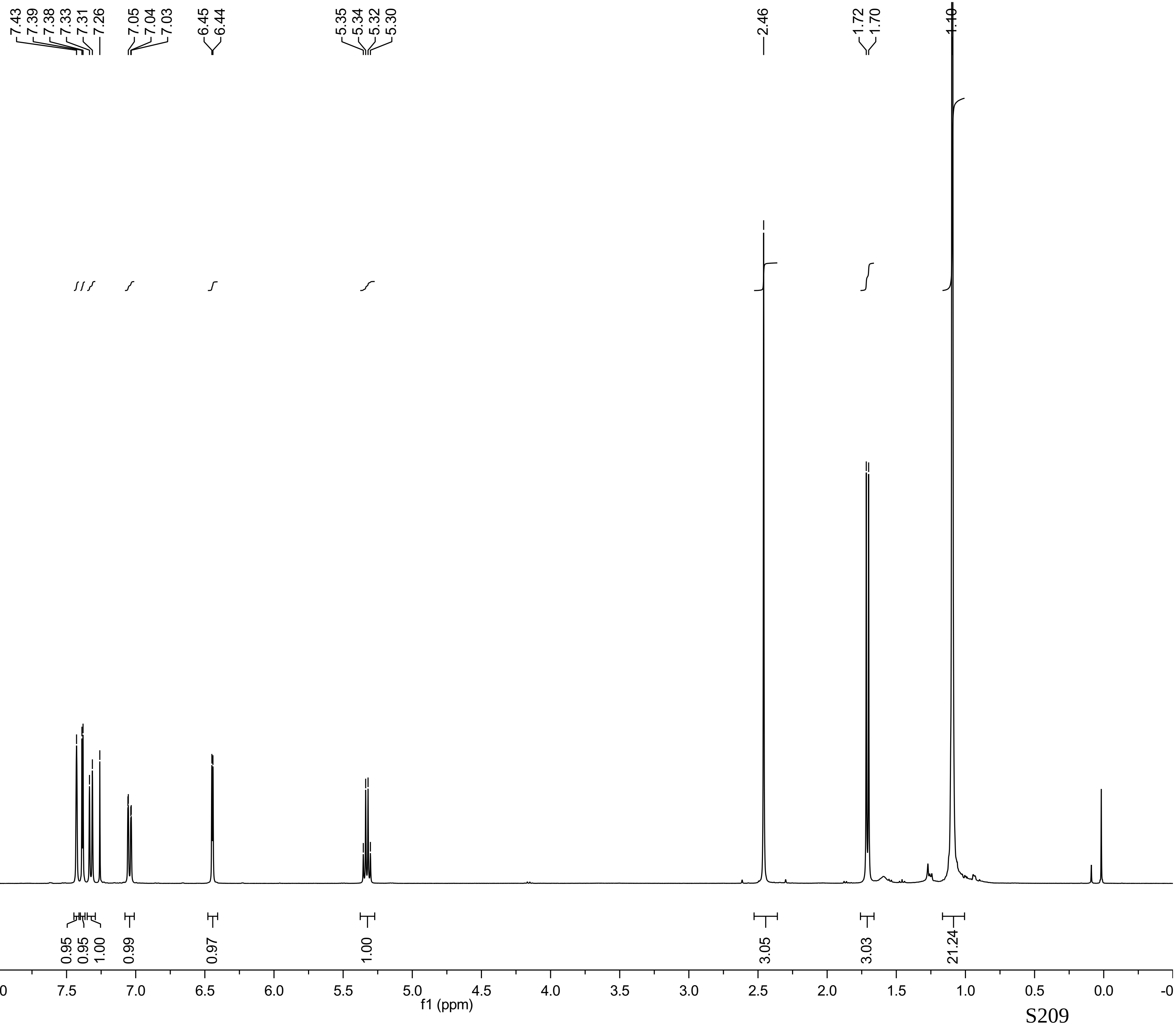

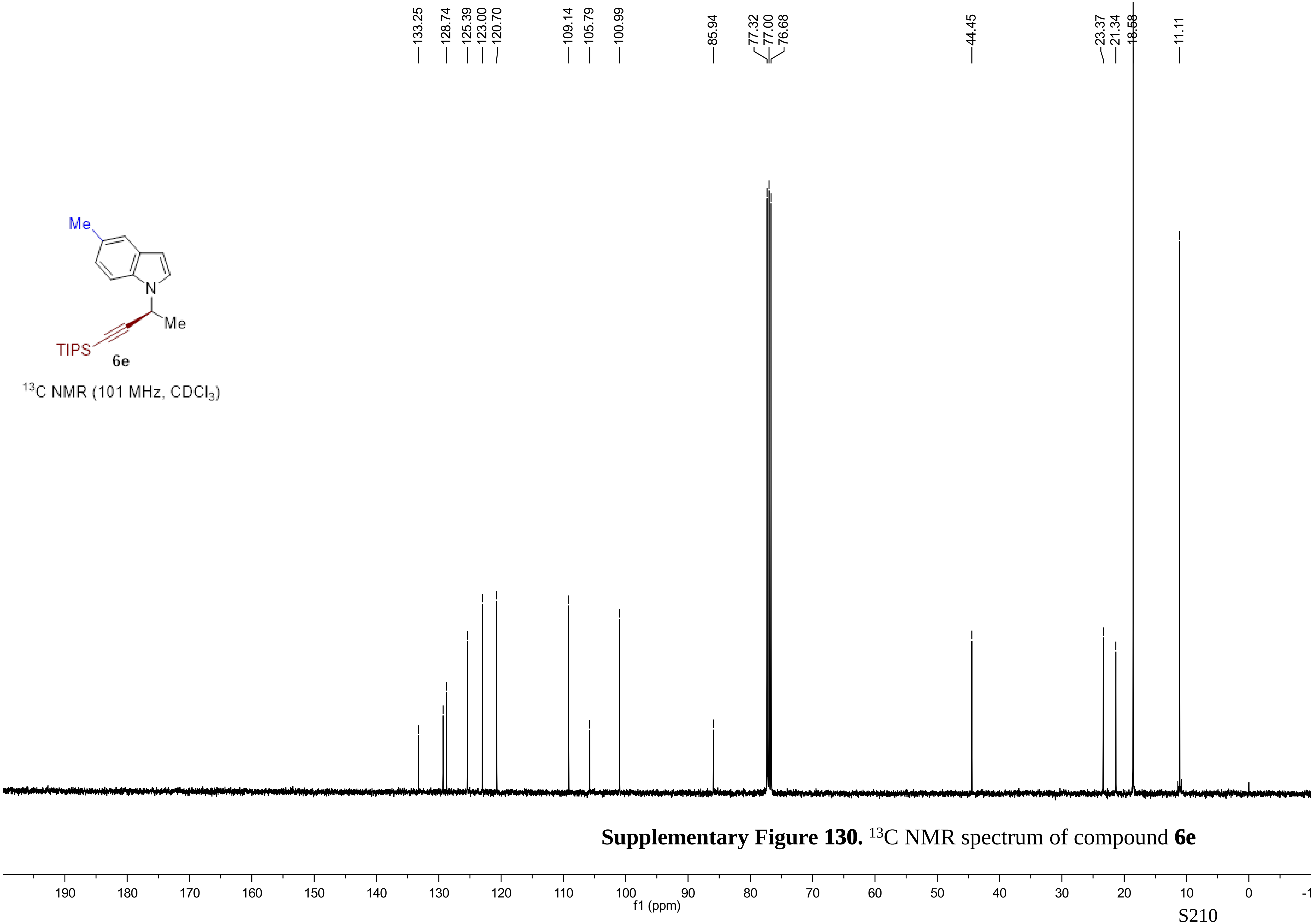

Supplementary Figure 130. ^{13}C NMR spectrum of compound **6e**

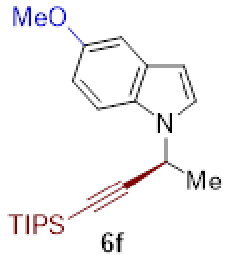

6f

^{1}H NMR (500 MHz, $CDCl_3$)

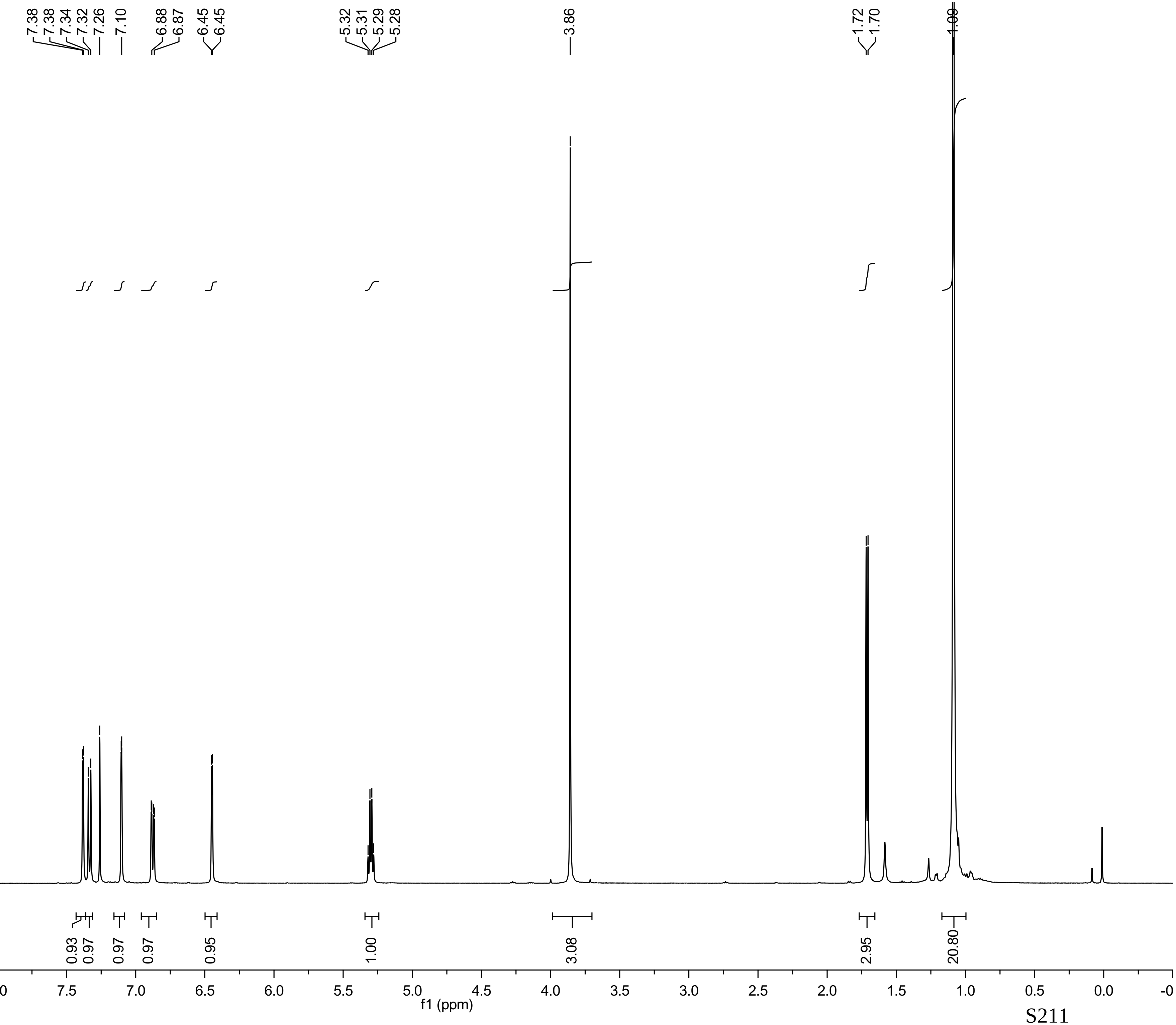

Supplementary Figure 131. ^{1}H NMR spectrum of compound **6f**

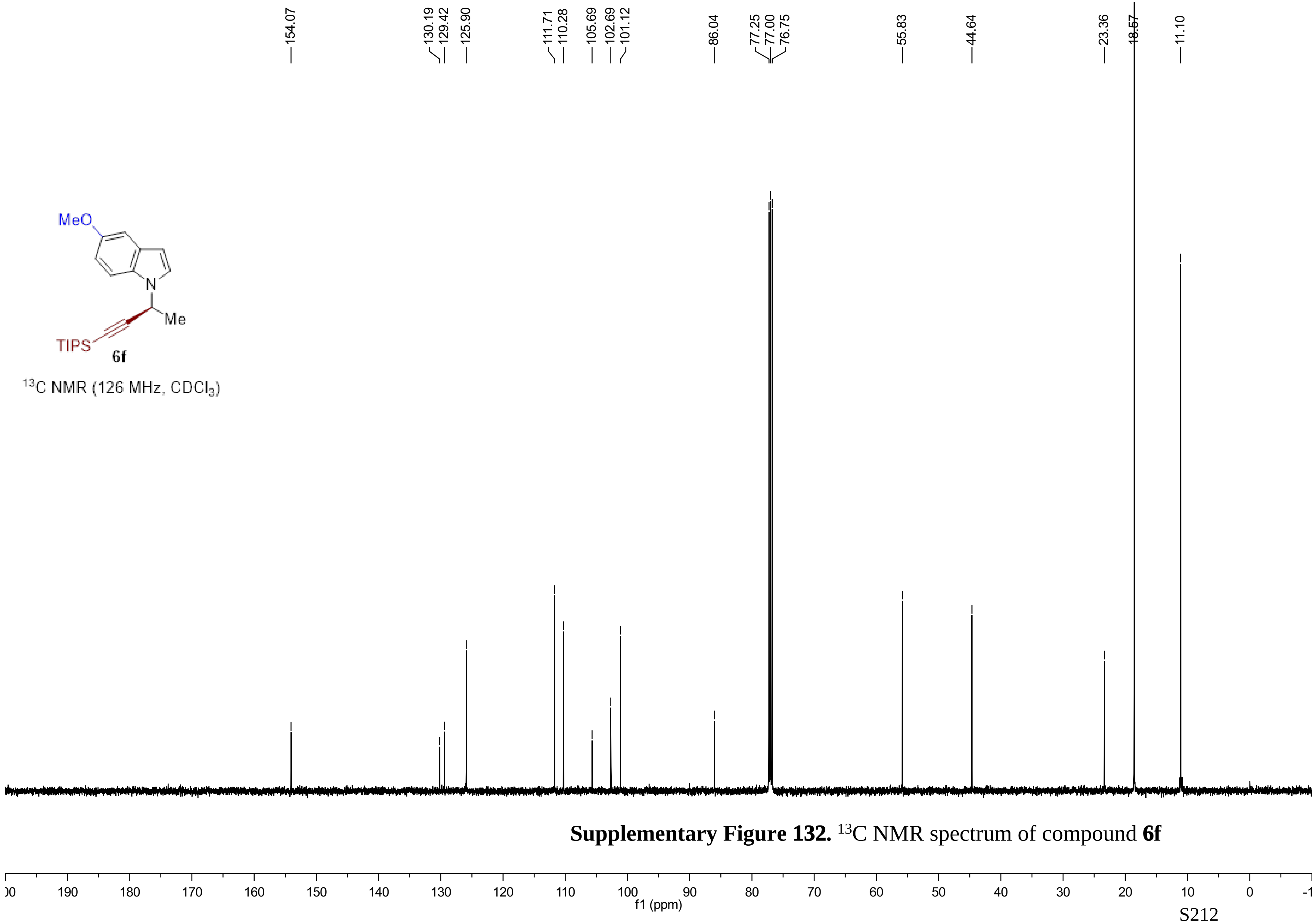

Supplementary Figure 132. ^{13}C NMR spectrum of compound **6f**

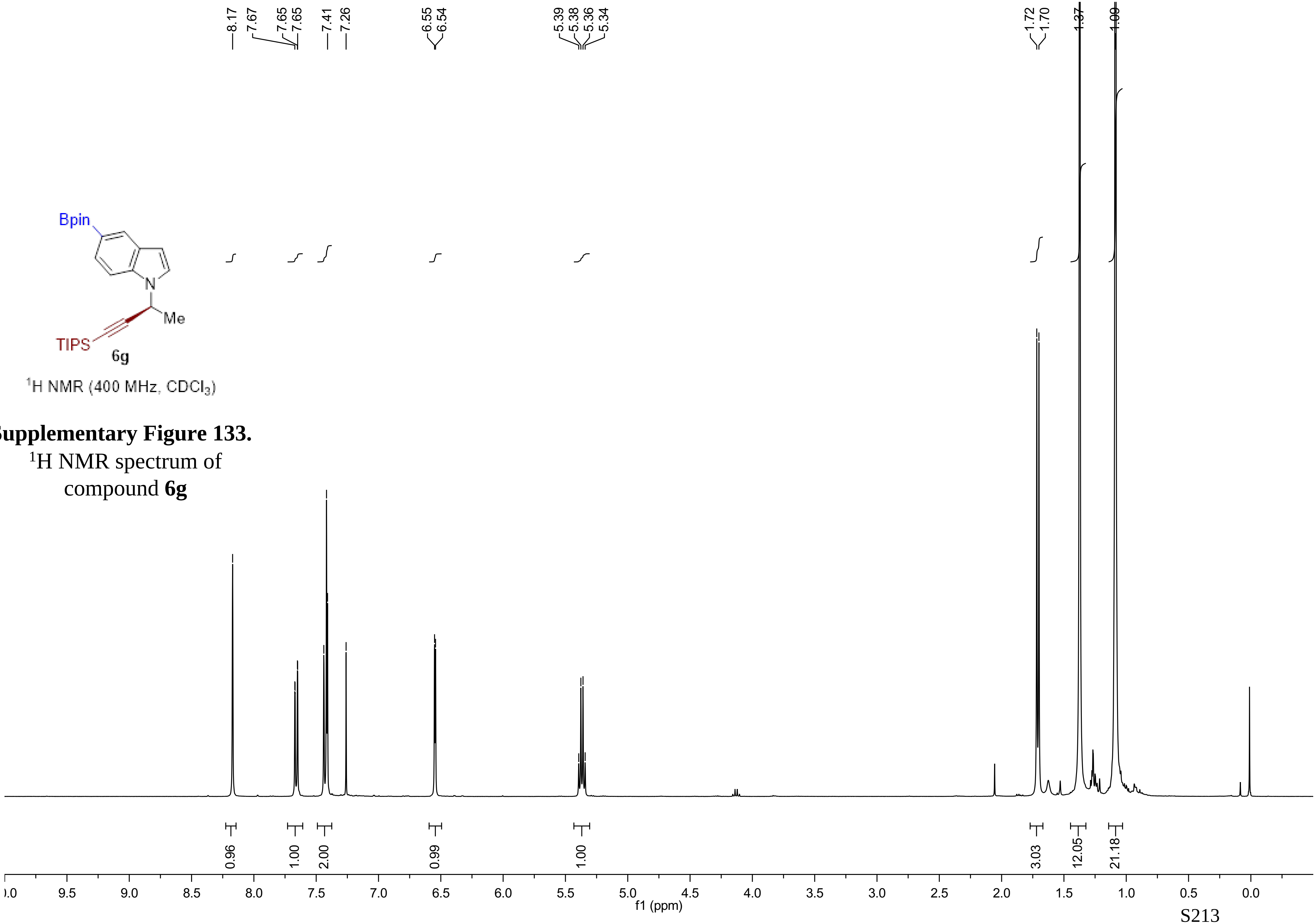

Supplementary Figure 133. ^{1}H NMR spectrum of compound **6g**

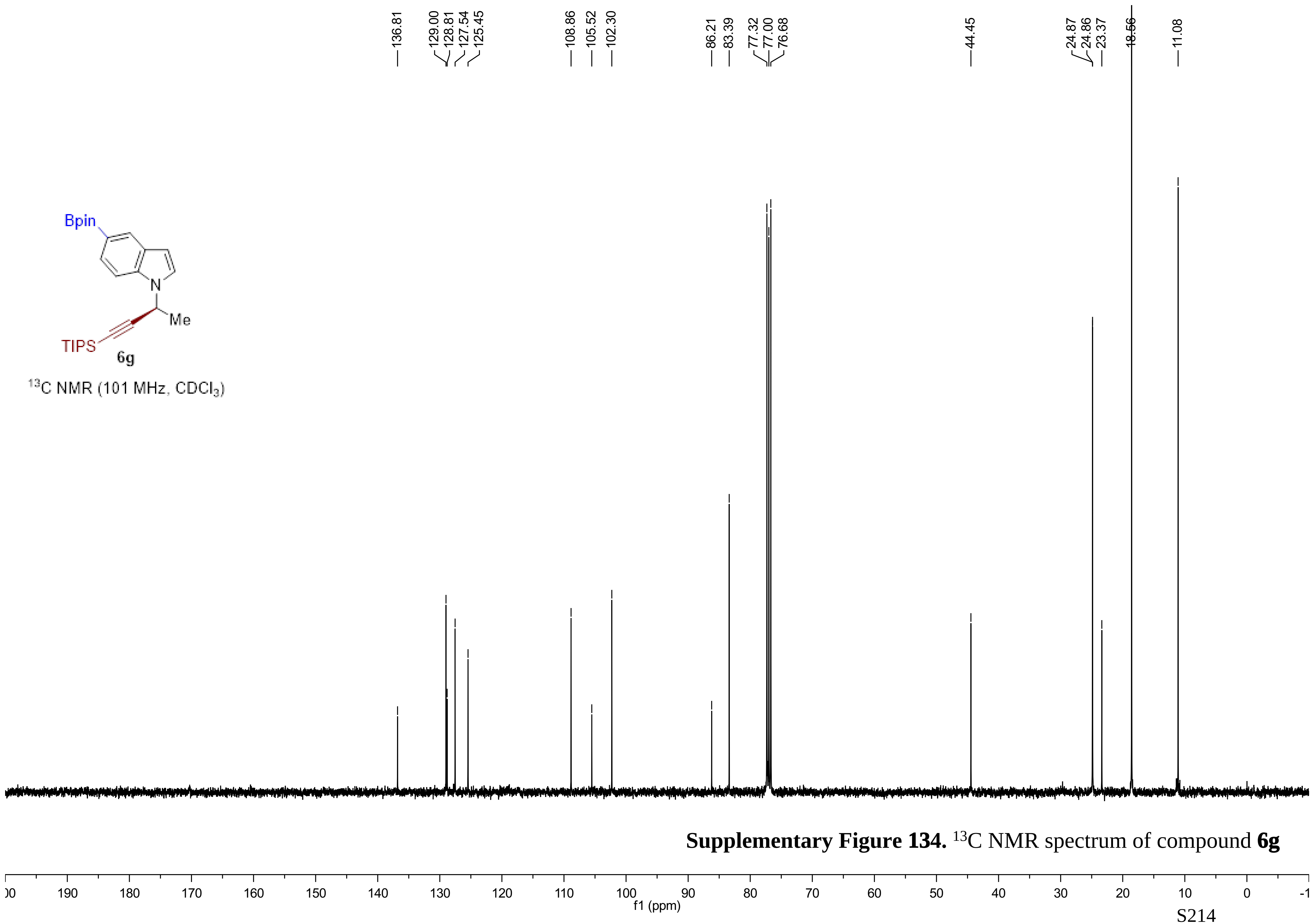

Supplementary Figure 134. ^{13}C NMR spectrum of compound **6g**

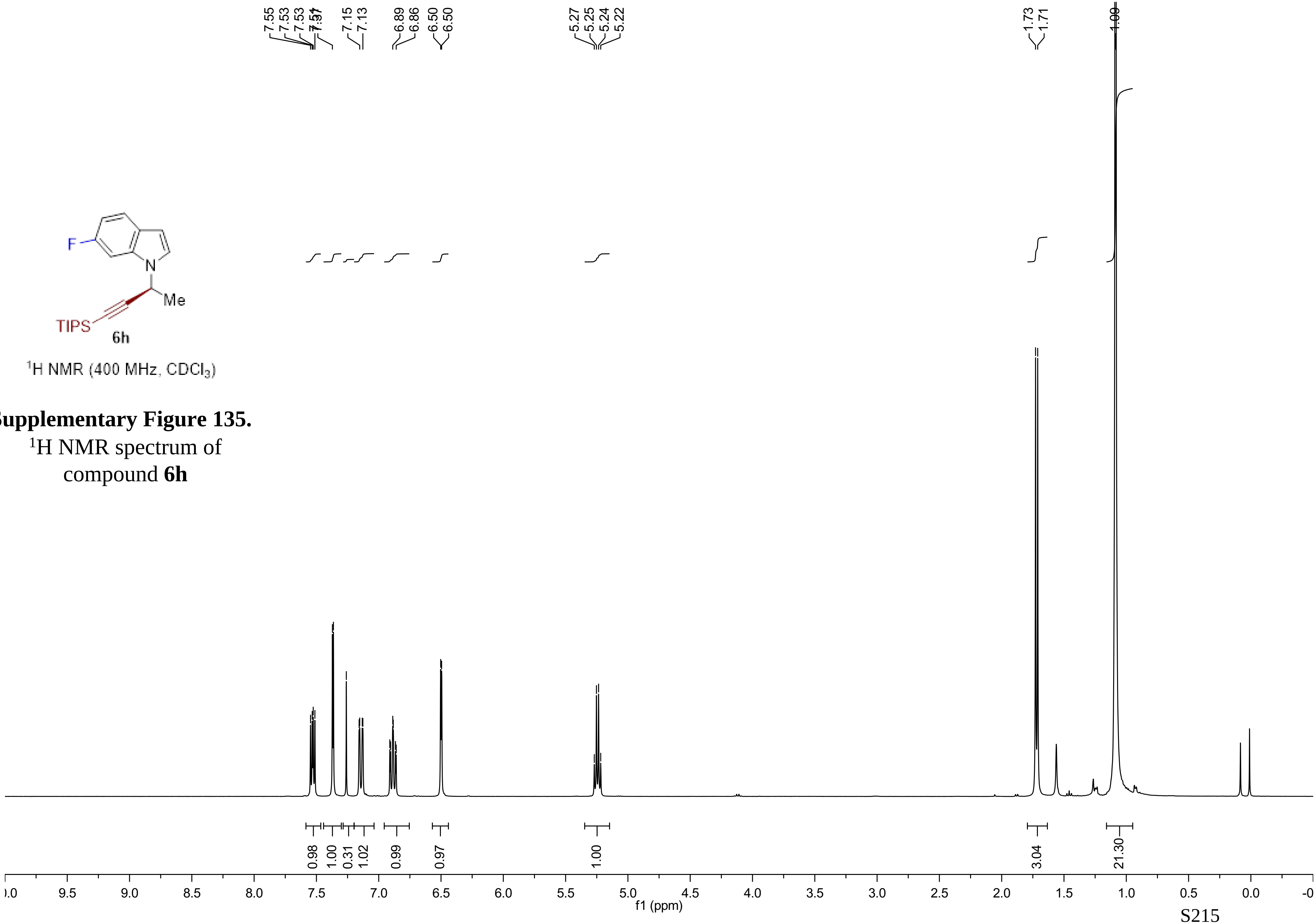

6h

^{1}H NMR (400 MHz, $CDCl_3$)

Supplementary Figure 135. ^{1}H NMR spectrum of compound **6h**

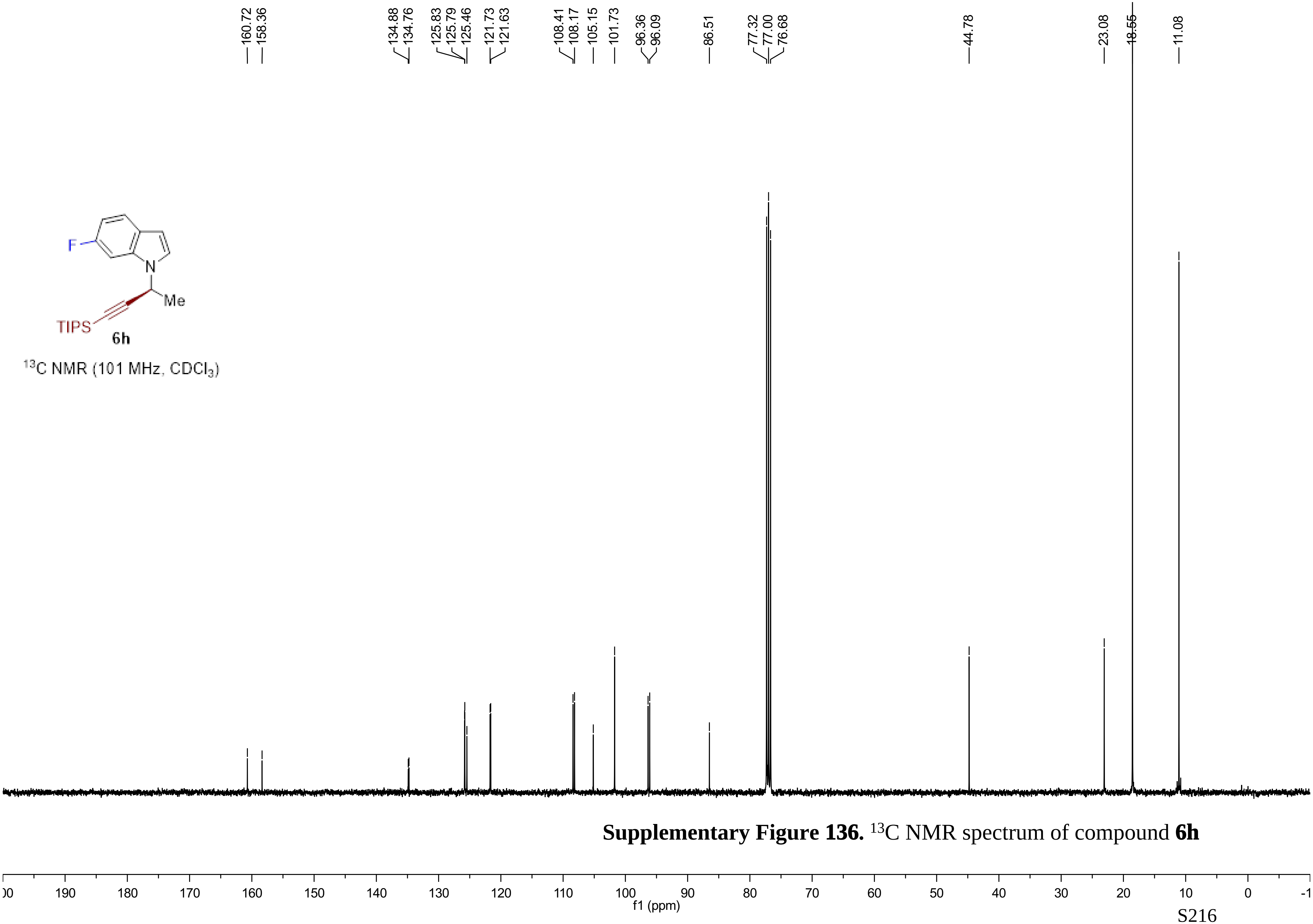

Supplementary Figure 136. ^{13}C NMR spectrum of compound **6h**

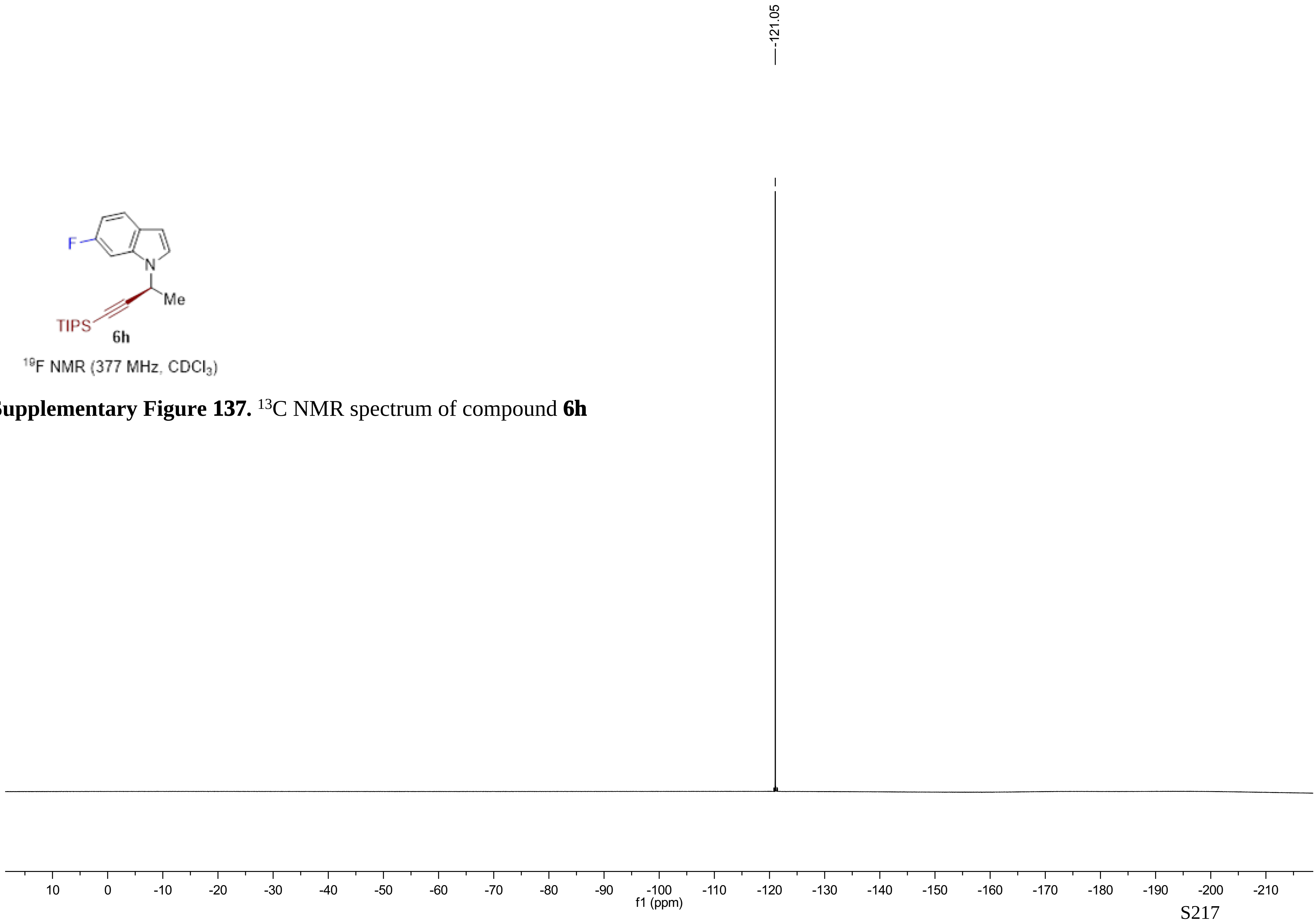

Supplementary Figure 137. ^{13}C NMR spectrum of compound **6h**

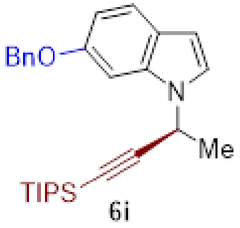

Supplementary Figure 138.
^{1}H NMR spectrum of compound **6i**

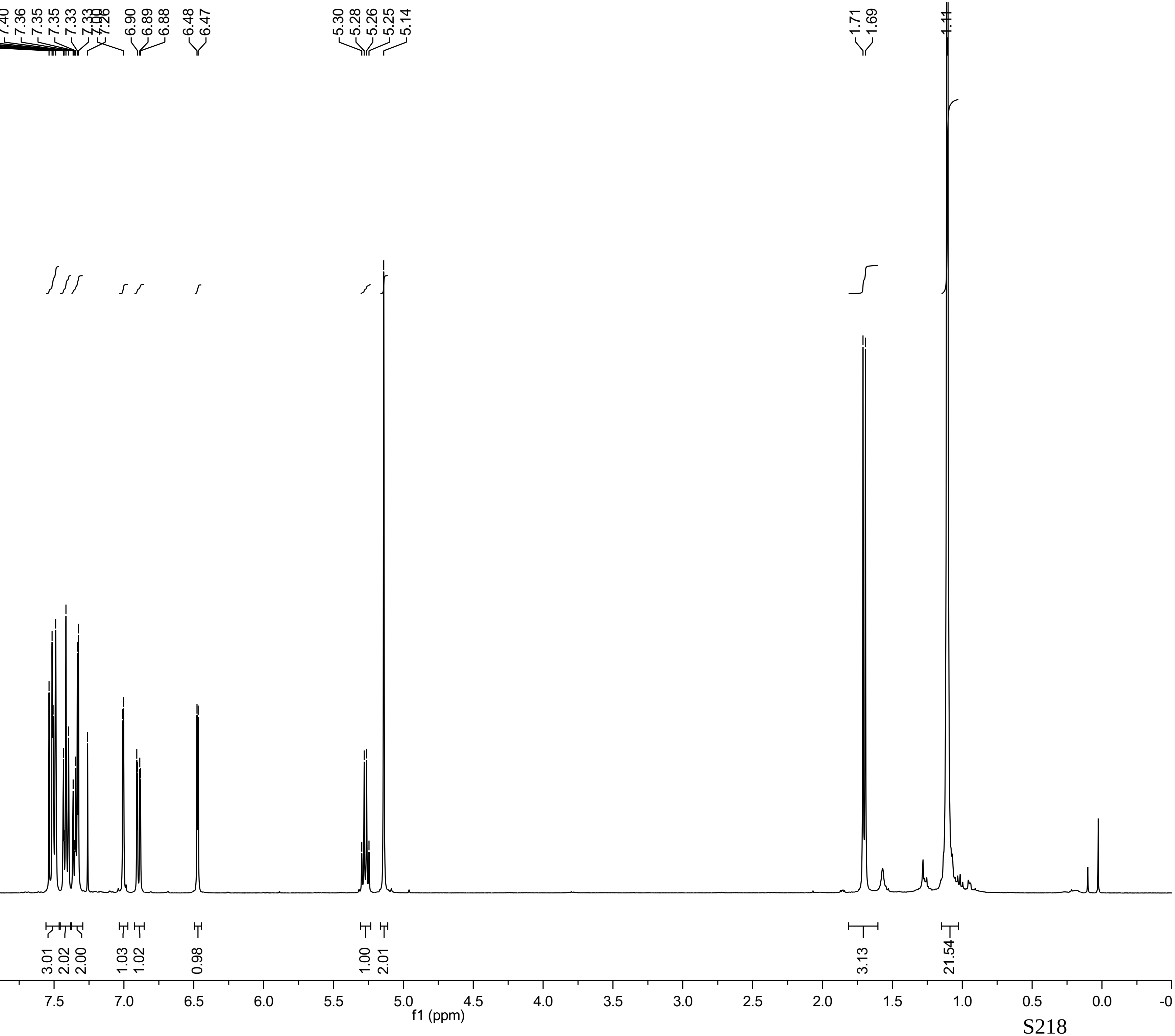

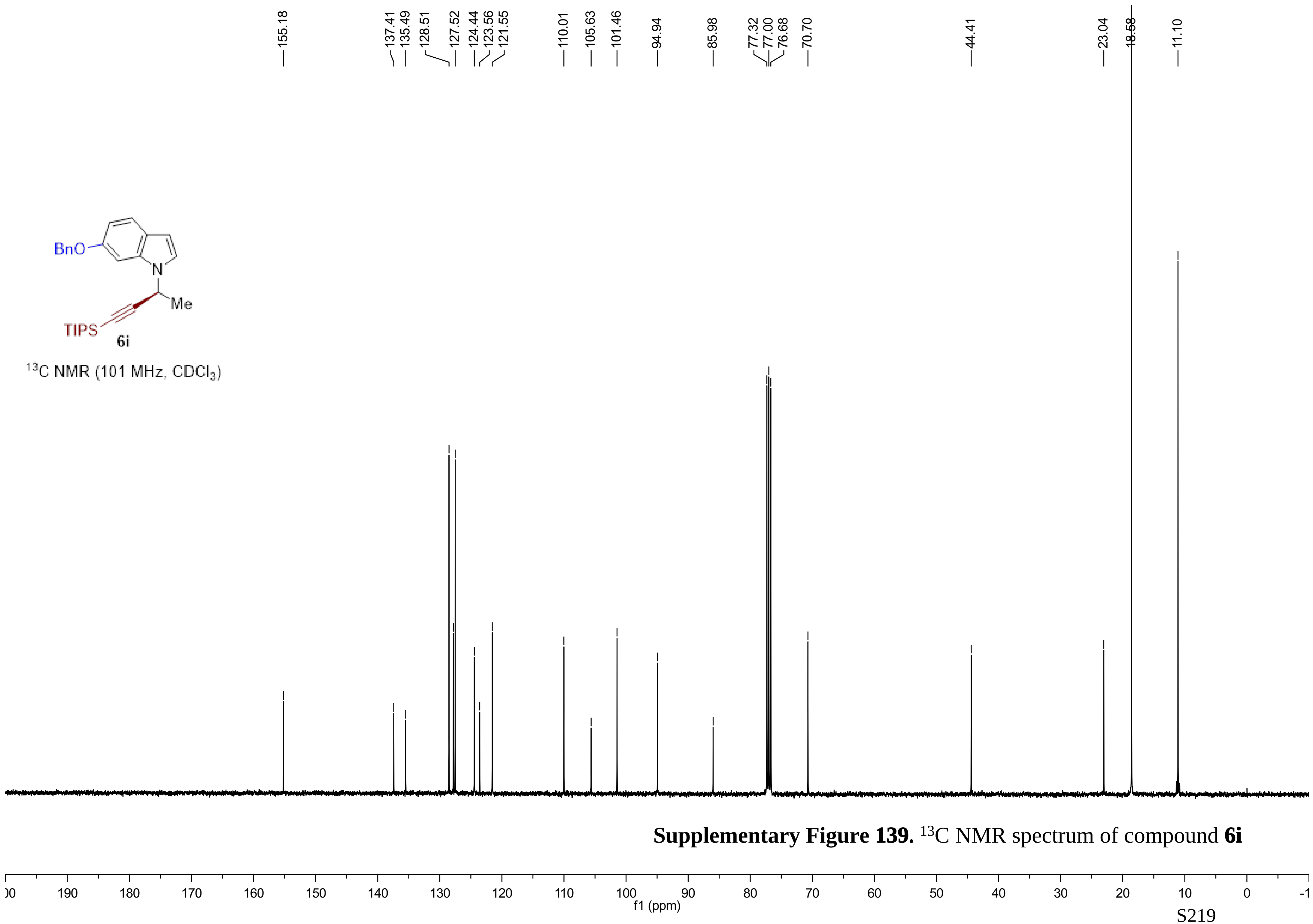

Supplementary Figure 139. ^{13}C NMR spectrum of compound **6i**

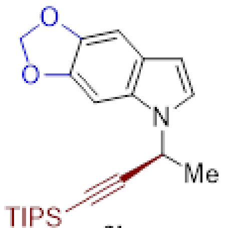

6j

^{1}H NMR (400 MHz, $CDCl_3$)

Supplementary Figure 140. ^{1}H NMR spectrum of compound **6j**

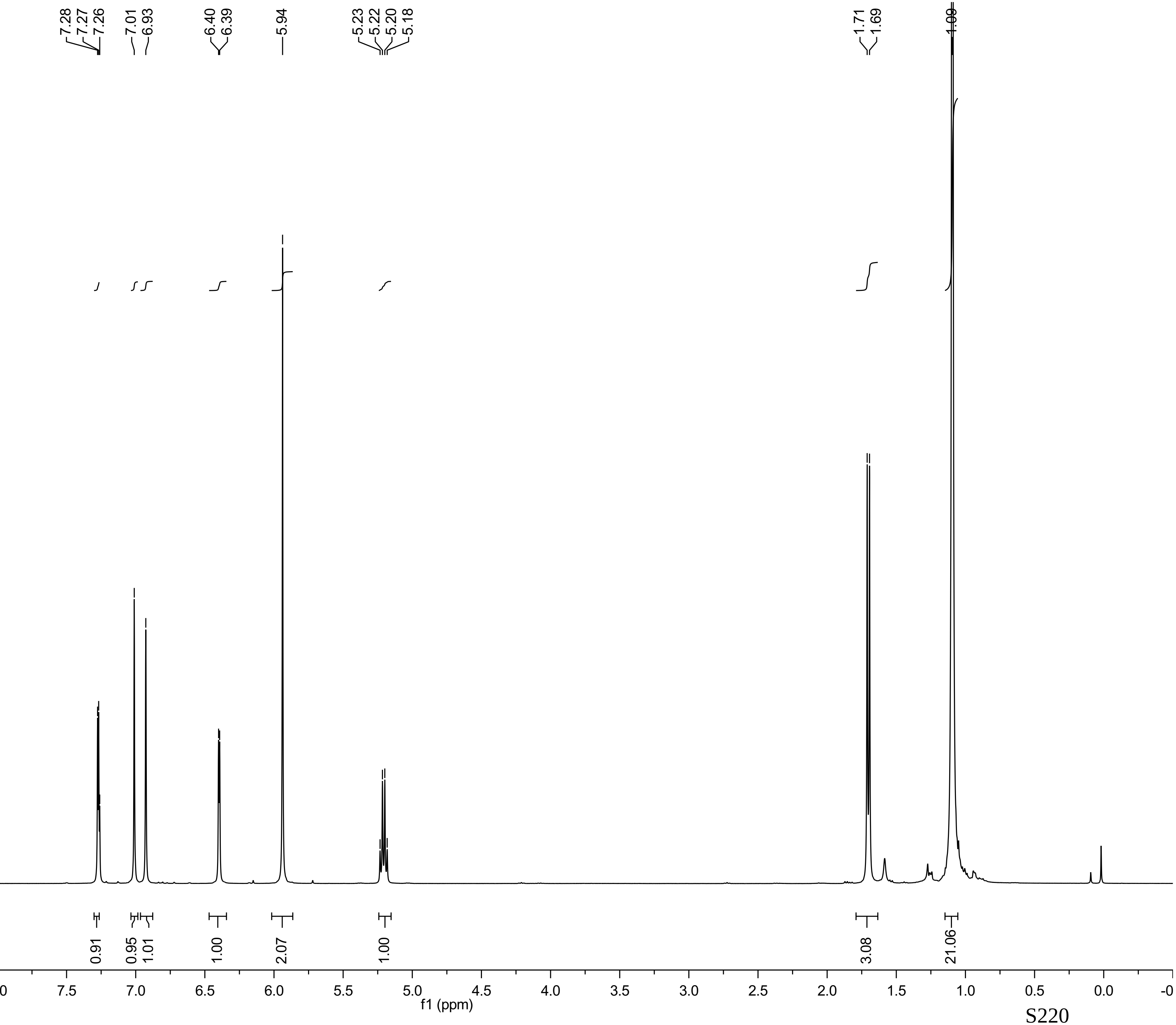

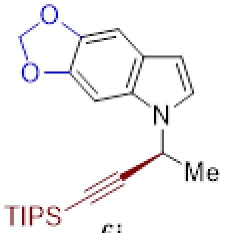

6j

^{13}C NMR (101 MHz, $CDCl_3$)

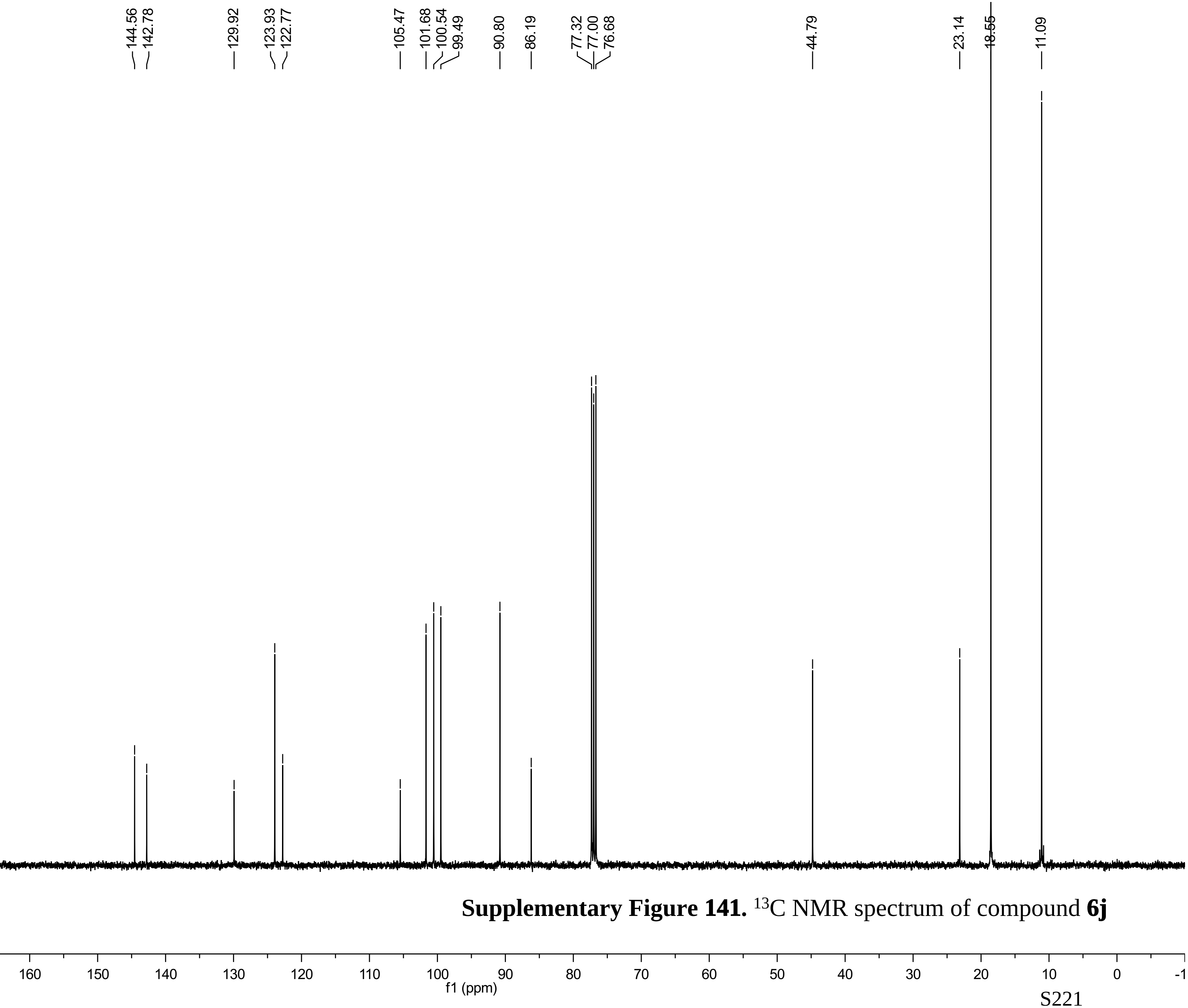

Supplementary Figure 141. ^{13}C NMR spectrum of compound **6j**

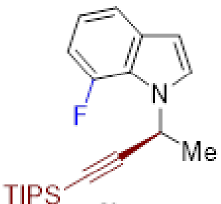

6k

^{1}H NMR (400 MHz, $CDCl_3$)

Supplementary Figure 142. ^{1}H NMR spectrum of compound **6k**

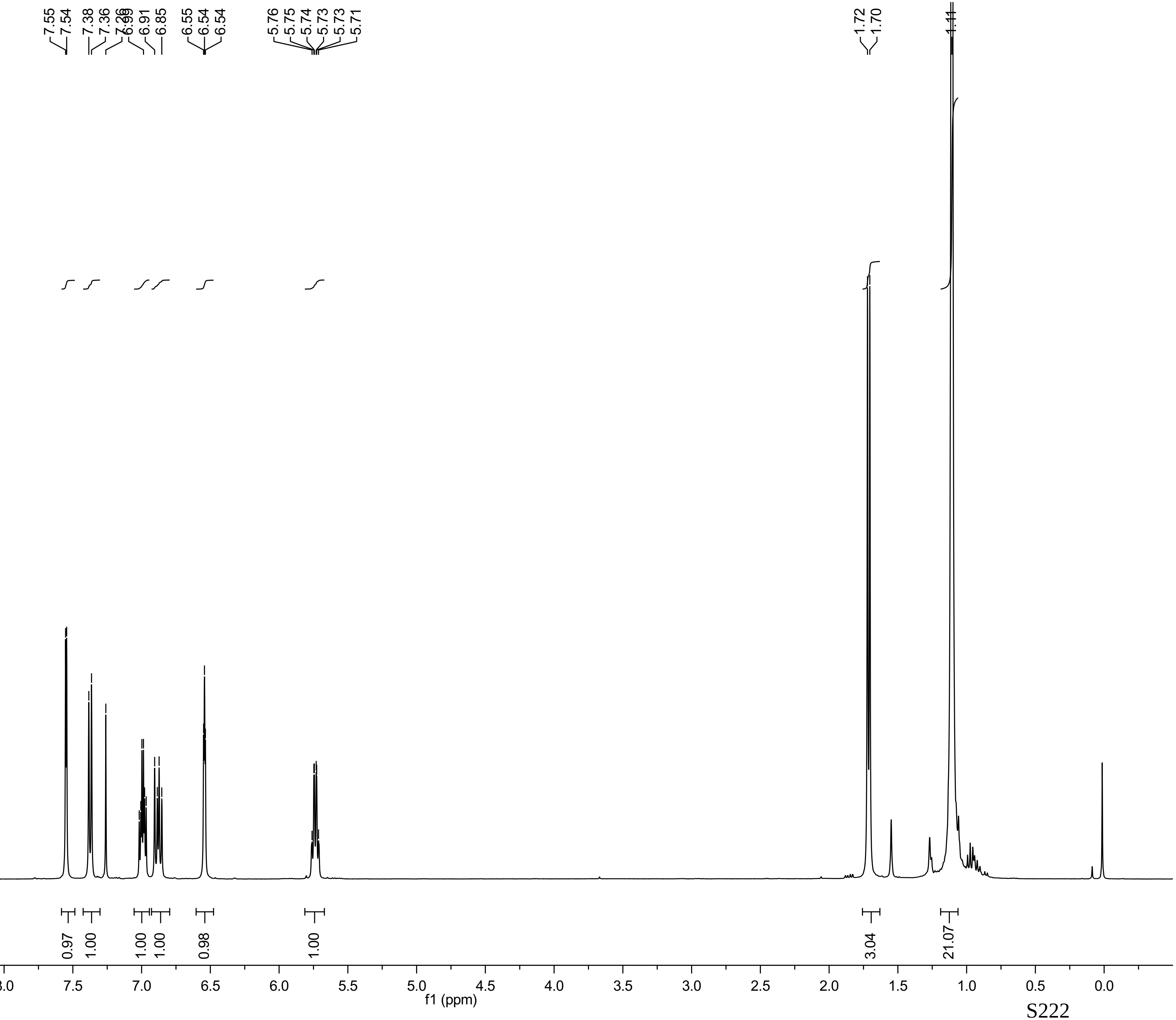

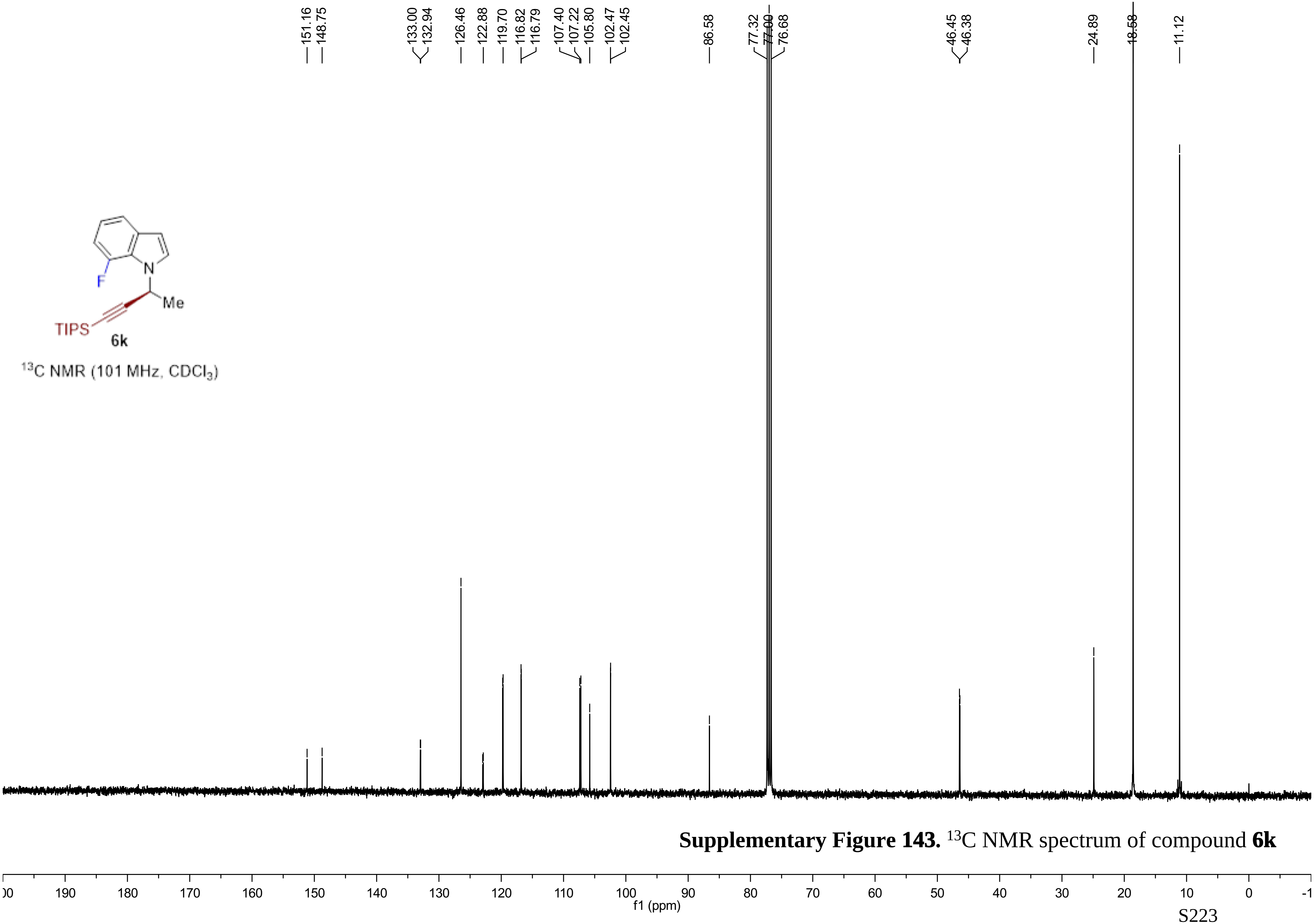

Supplementary Figure 143. ^{13}C NMR spectrum of compound **6k**

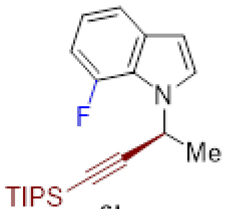

6k

^{19}F NMR (377 MHz, $CDCl_3$)

Supplementary Figure 144. ^{19}F NMR spectrum of compound **6k**

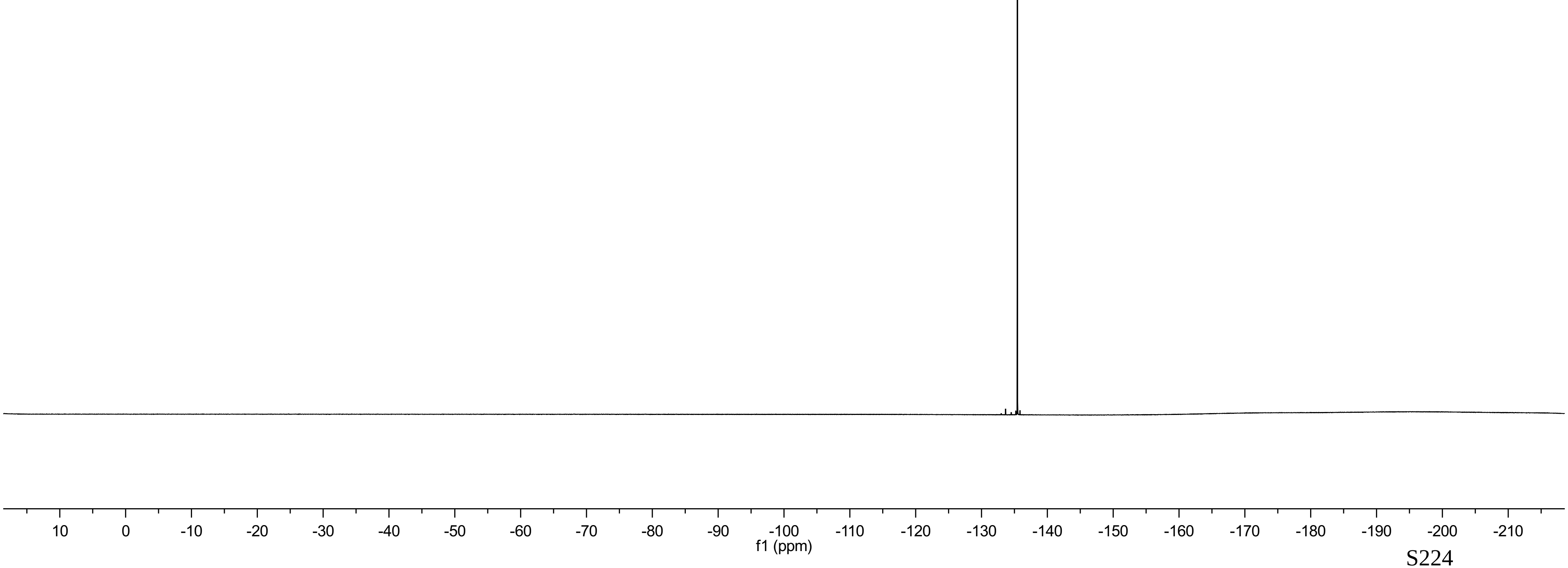

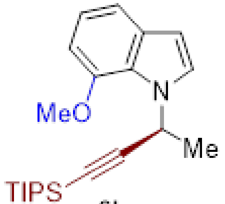

6l

^{1}H NMR (400 MHz, $CDCl_3$)

Supplementary Figure 145.
^{1}H NMR spectrum of compound **6l**

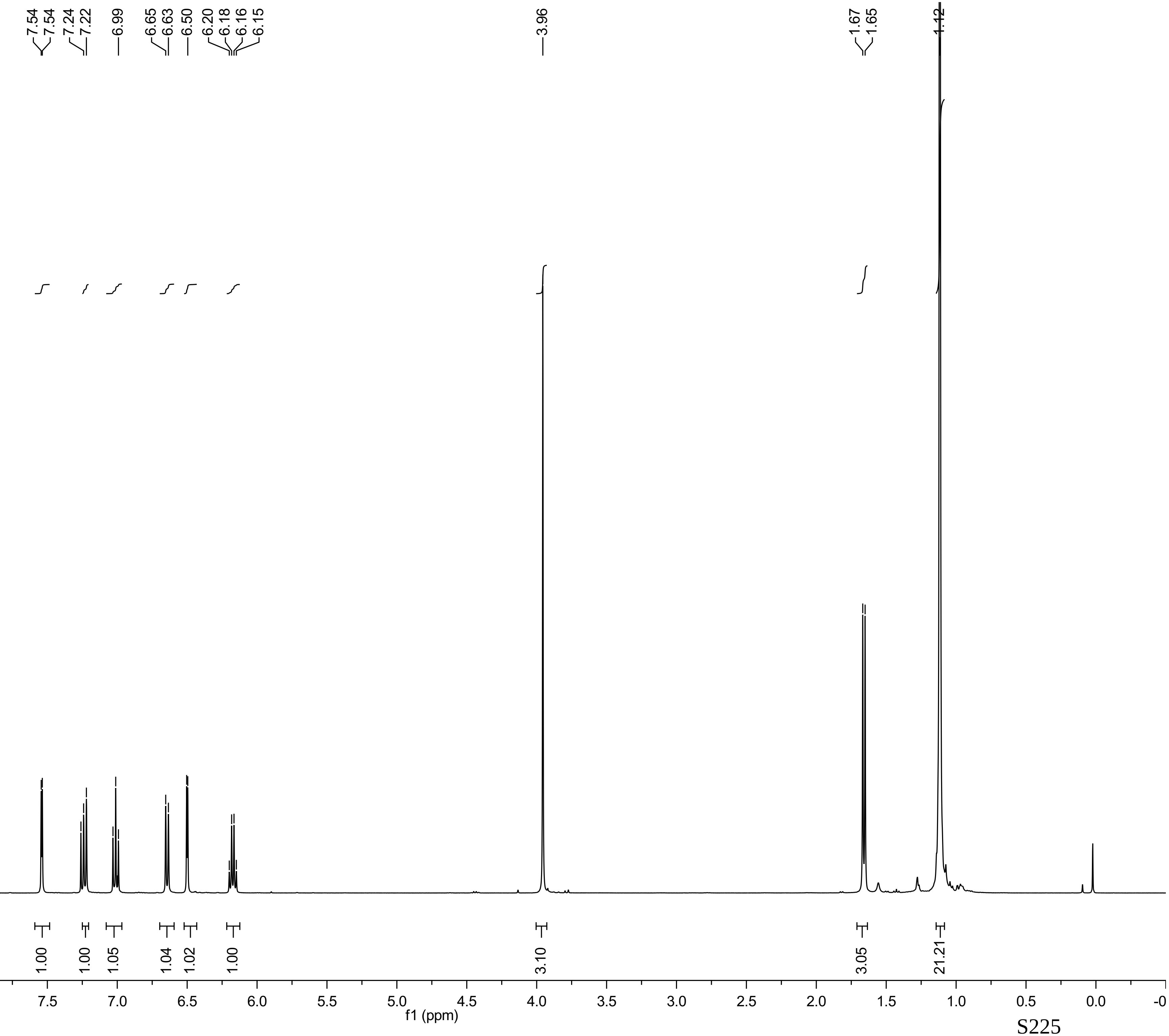

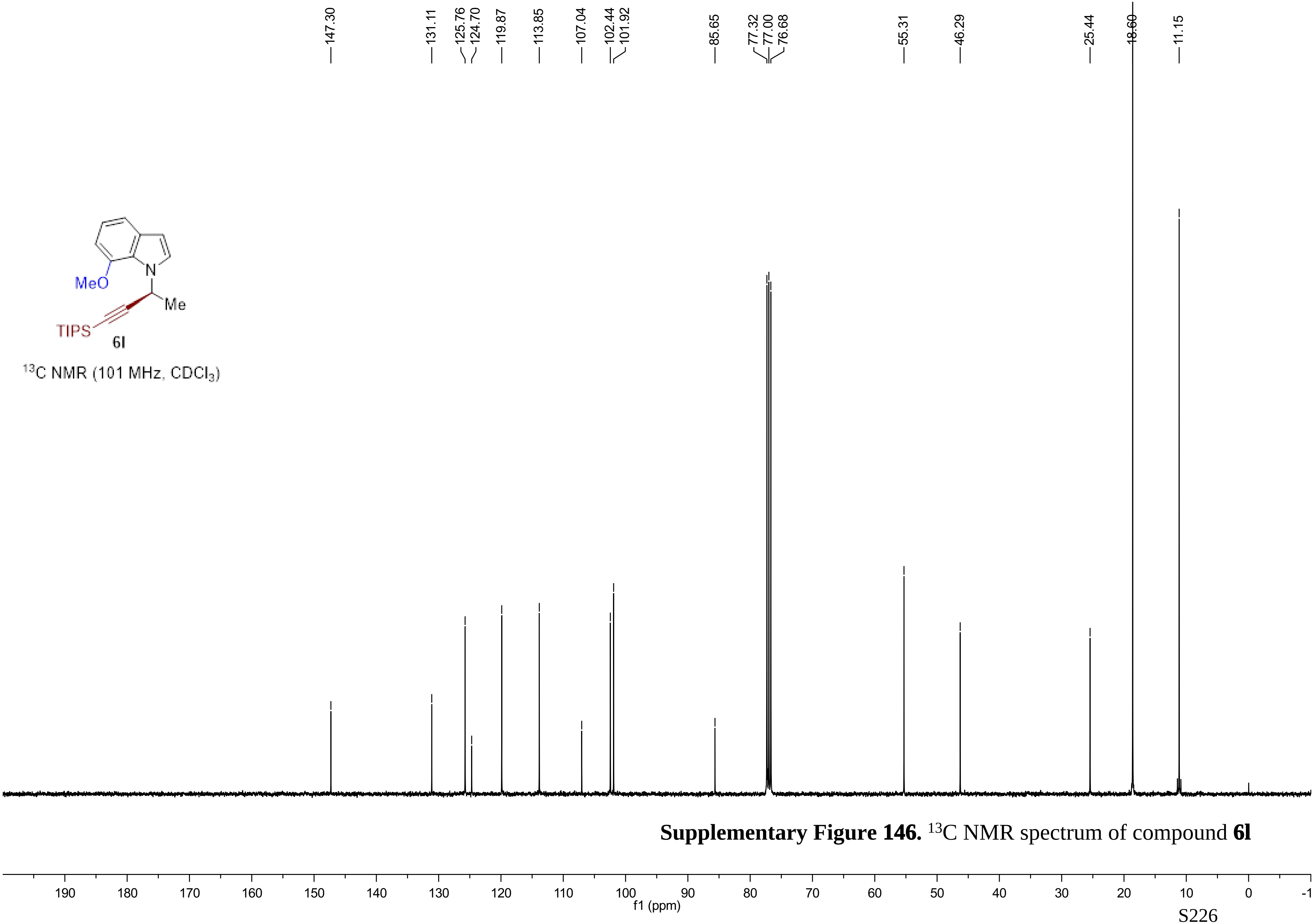

Supplementary Figure 146. ^{13}C NMR spectrum of compound **6l**

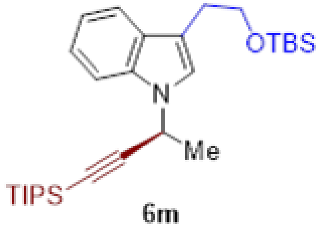

6m

^1H NMR (600 MHz, $CDCl_3$)

Supplementary Figure 147. ^1H NMR spectrum of compound **6m**

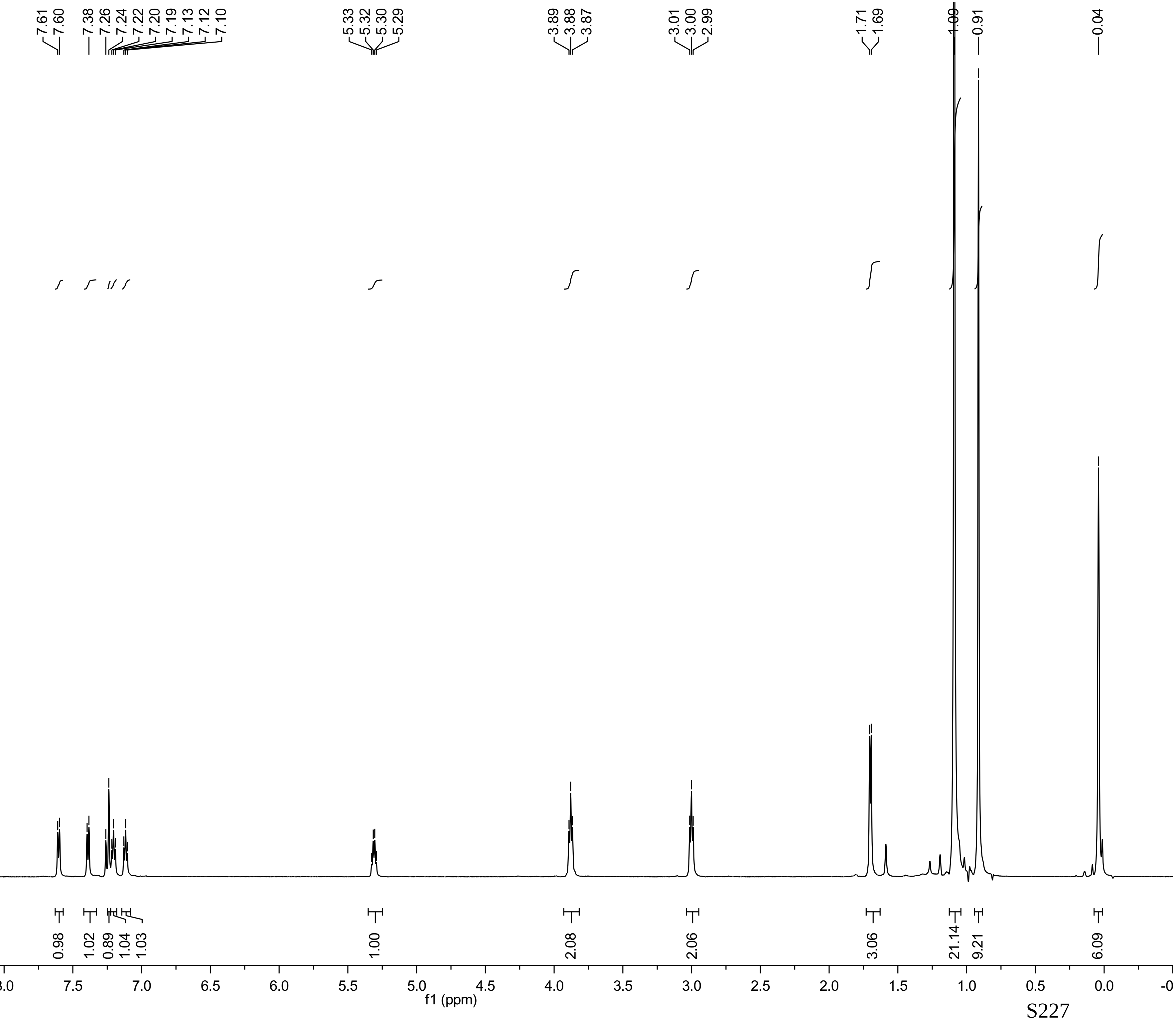

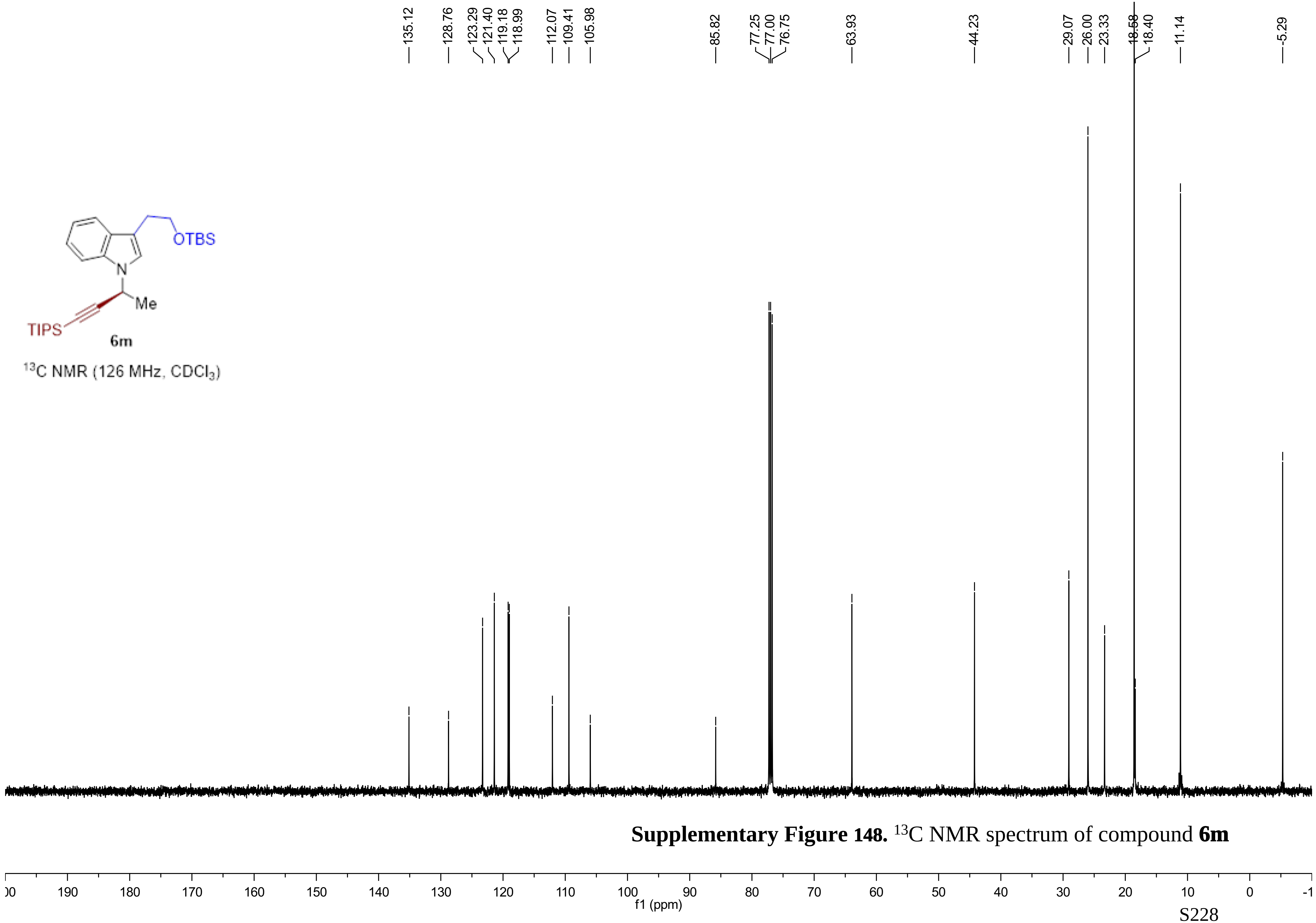

Supplementary Figure 148. ^{13}C NMR spectrum of compound **6m**

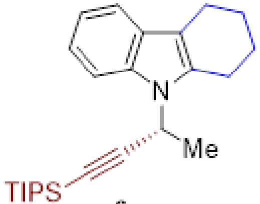

6n

^{1}H NMR (400 MHz, $CDCl_3$)

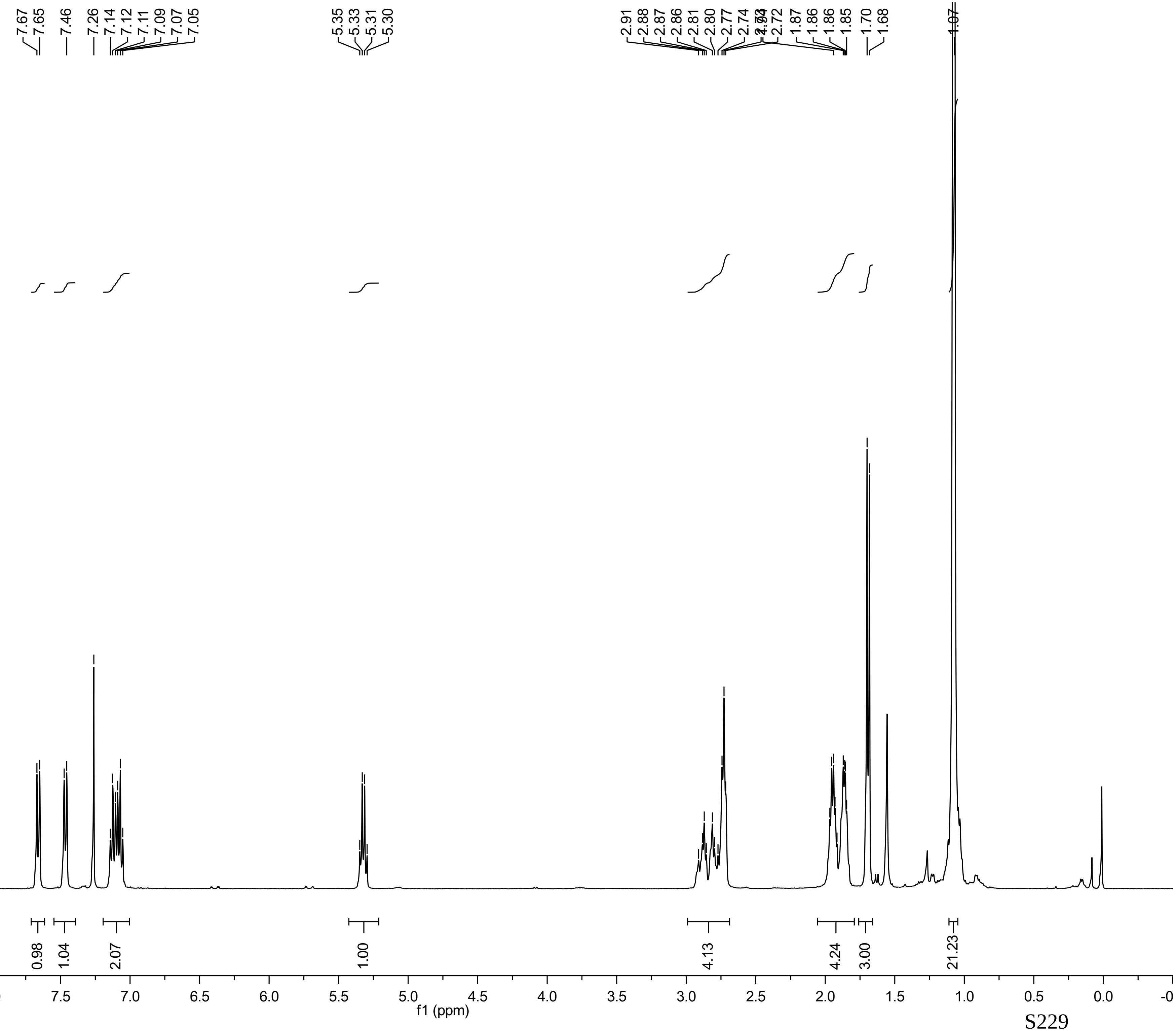

Supplementary Figure 149. ^{1}H NMR spectrum of compound **6n**

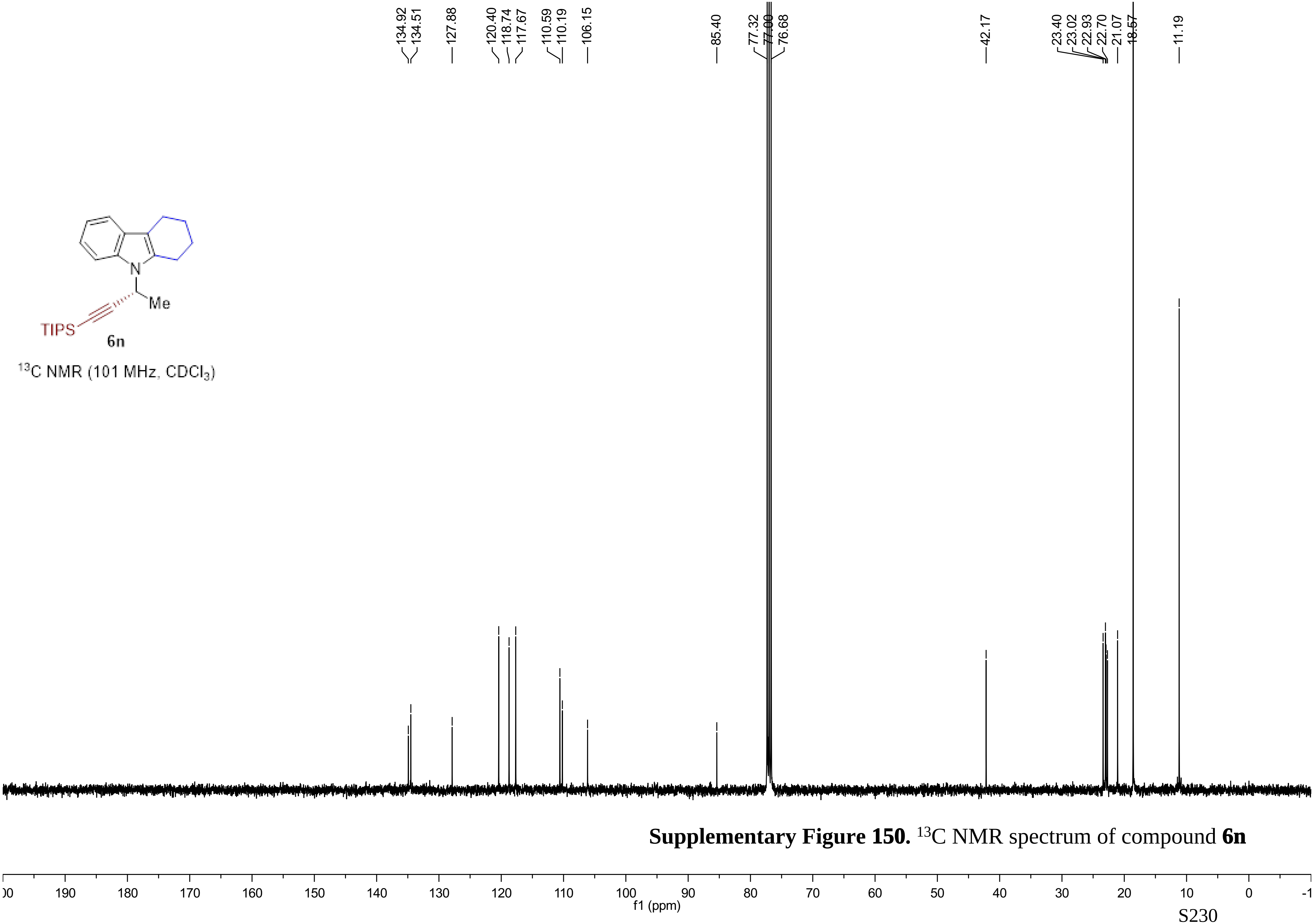

Supplementary Figure 150. ^{13}C NMR spectrum of compound **6n**

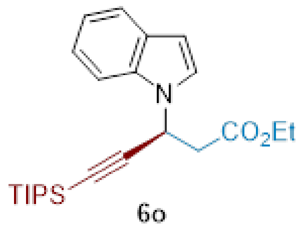

6o

^{1}H NMR (400 MHz, $CDCl_3$)

Supplementary Figure 151. ^{1}H NMR spectrum of compound **6o**

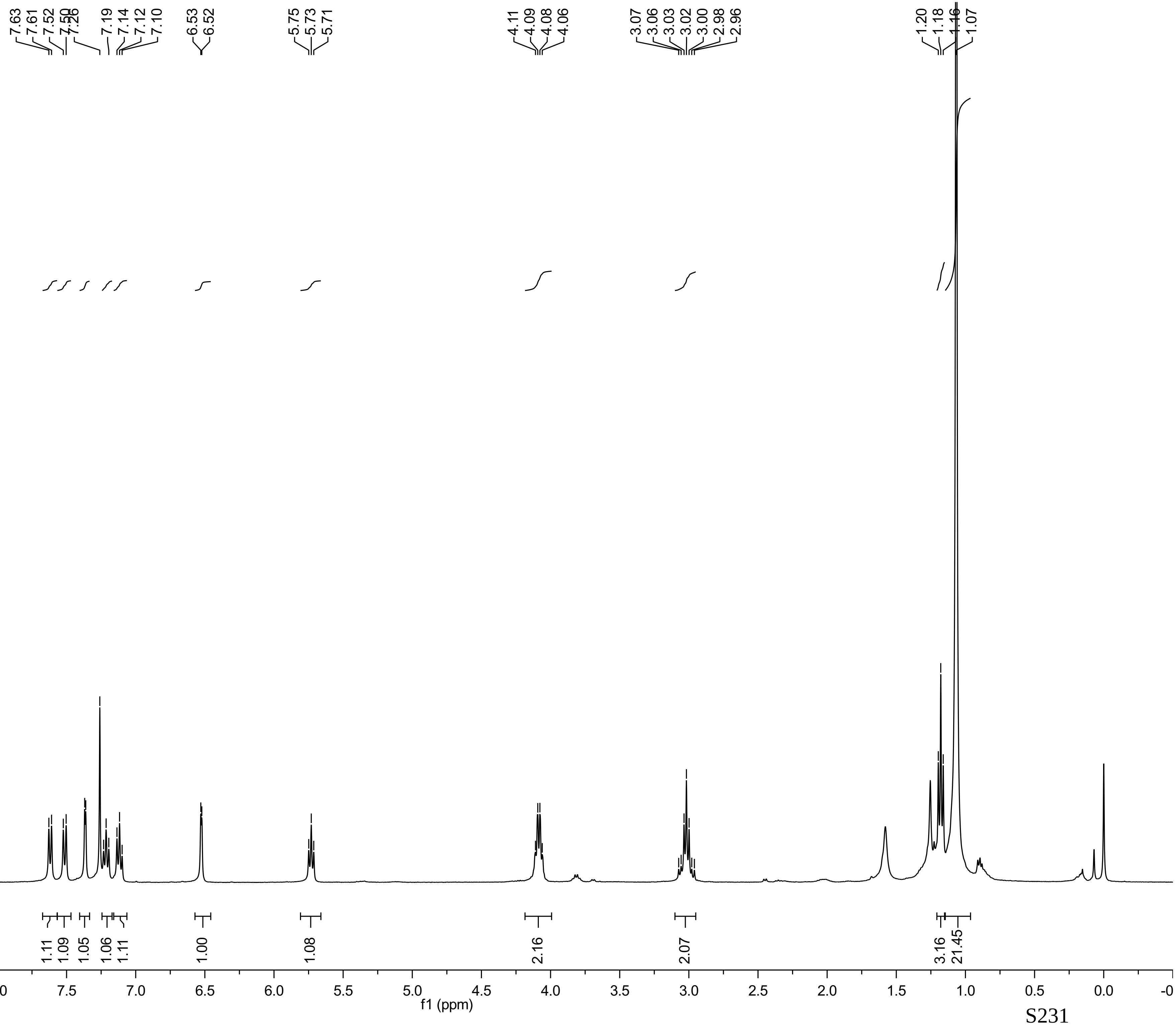

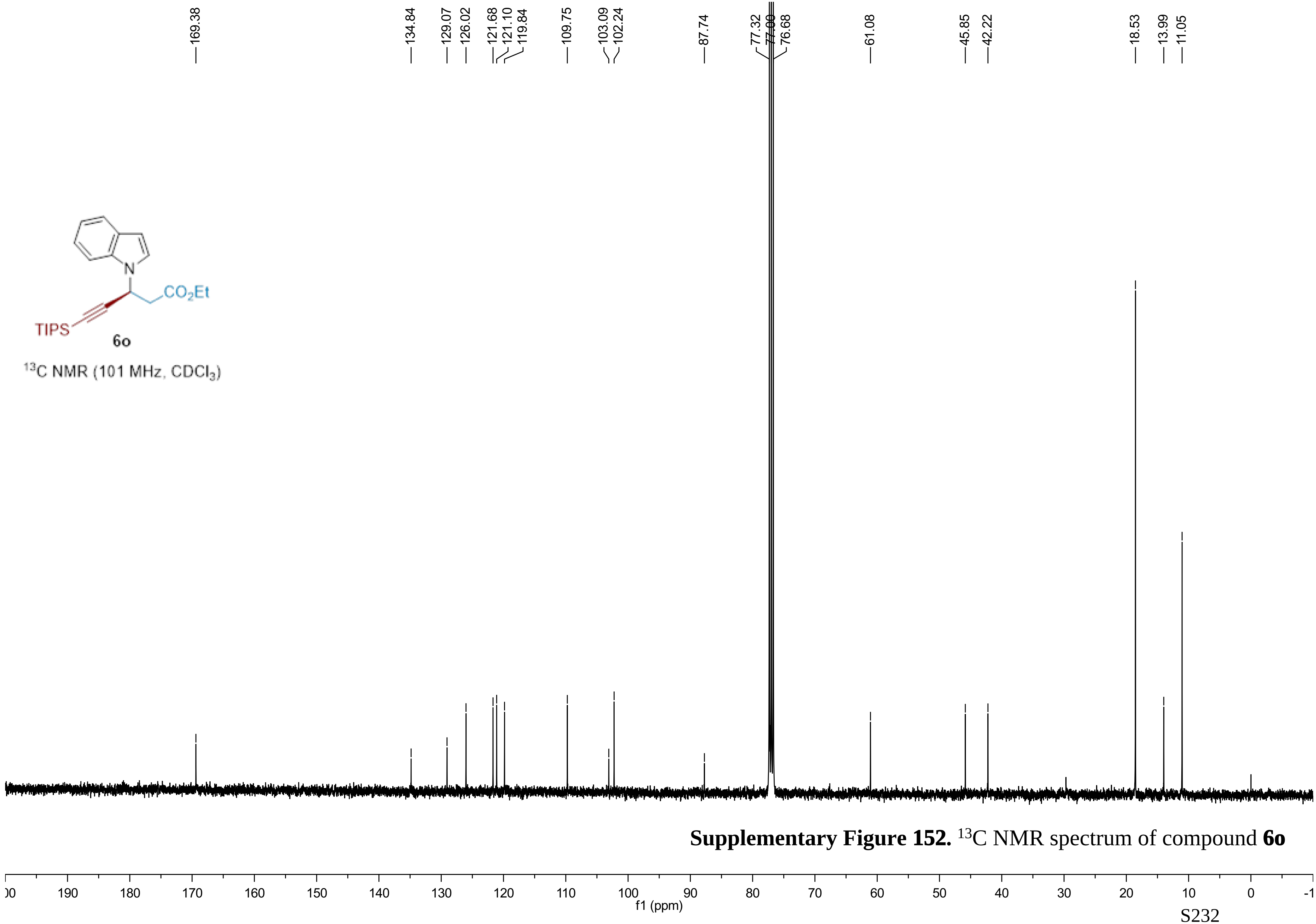

Supplementary Figure 152. ^{13}C NMR spectrum of compound **6o**

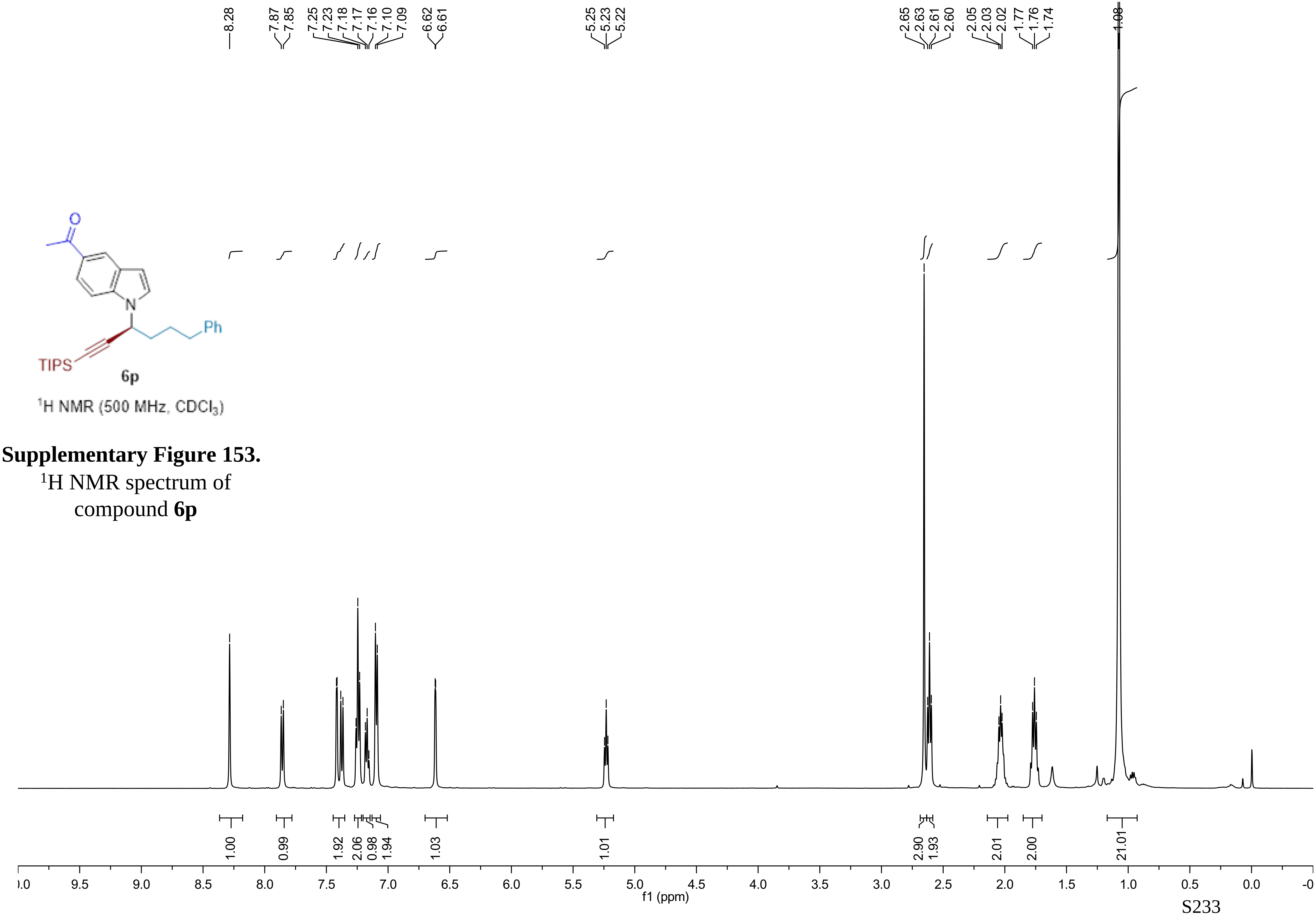

Supplementary Figure 153. ^{1}H NMR spectrum of compound **6p**

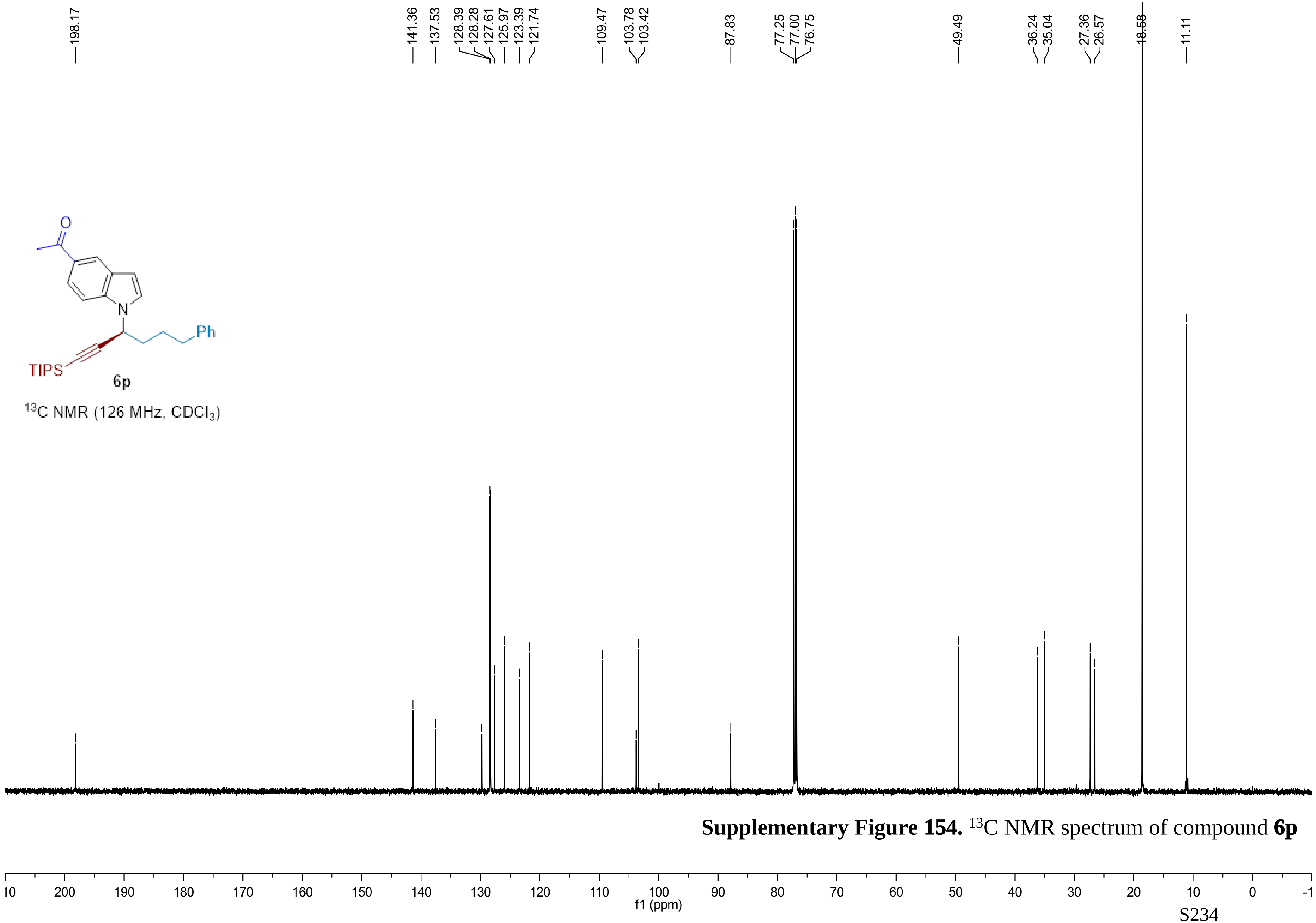

Supplementary Figure 154. ^{13}C NMR spectrum of compound **6p**

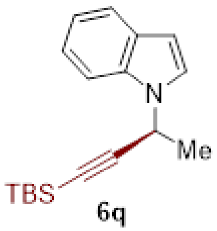

6q

^{1}H NMR (500 MHz, $CDCl_3$)

Supplementary Figure 155.
^{1}H NMR spectrum of compound **6q**

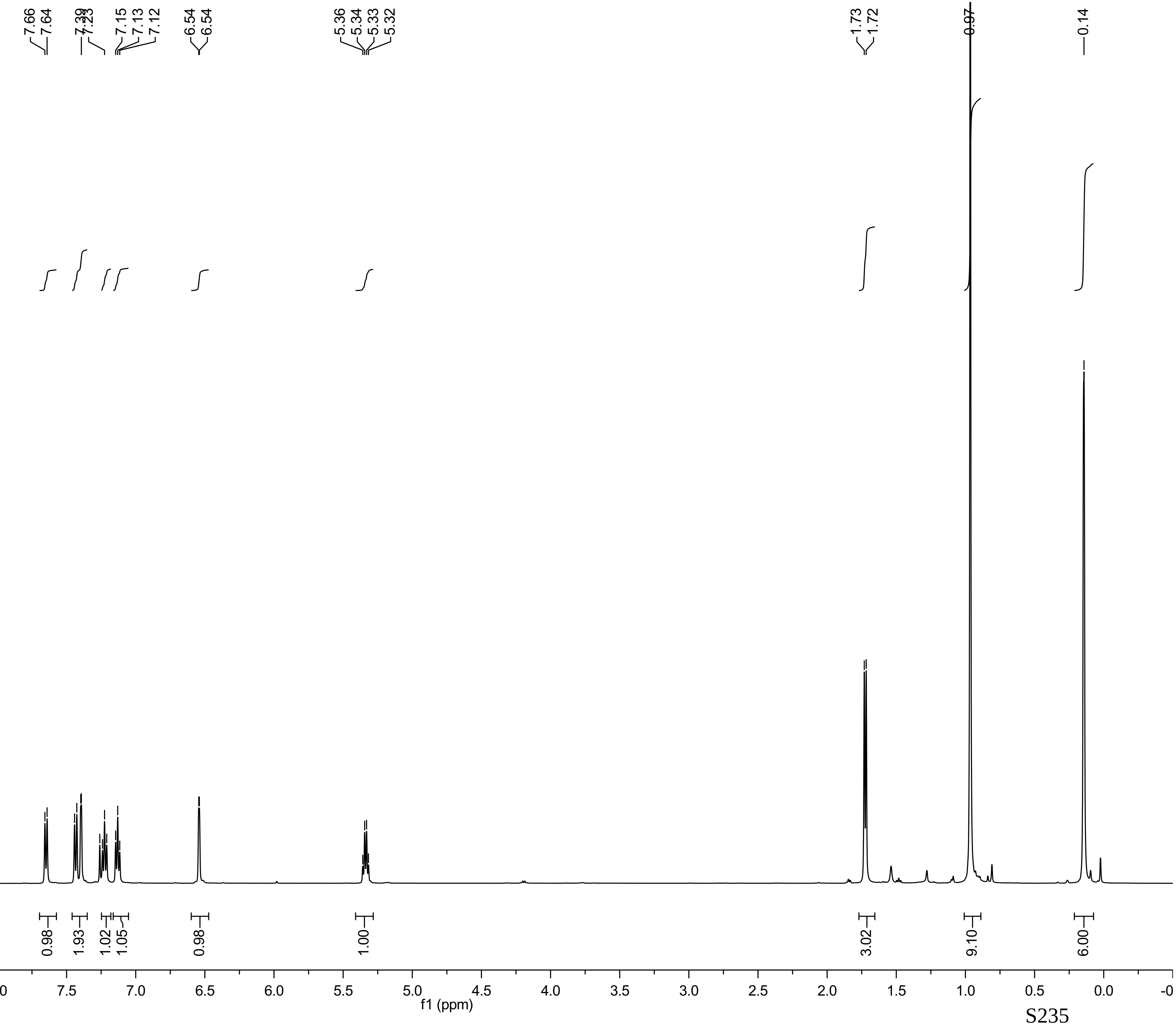

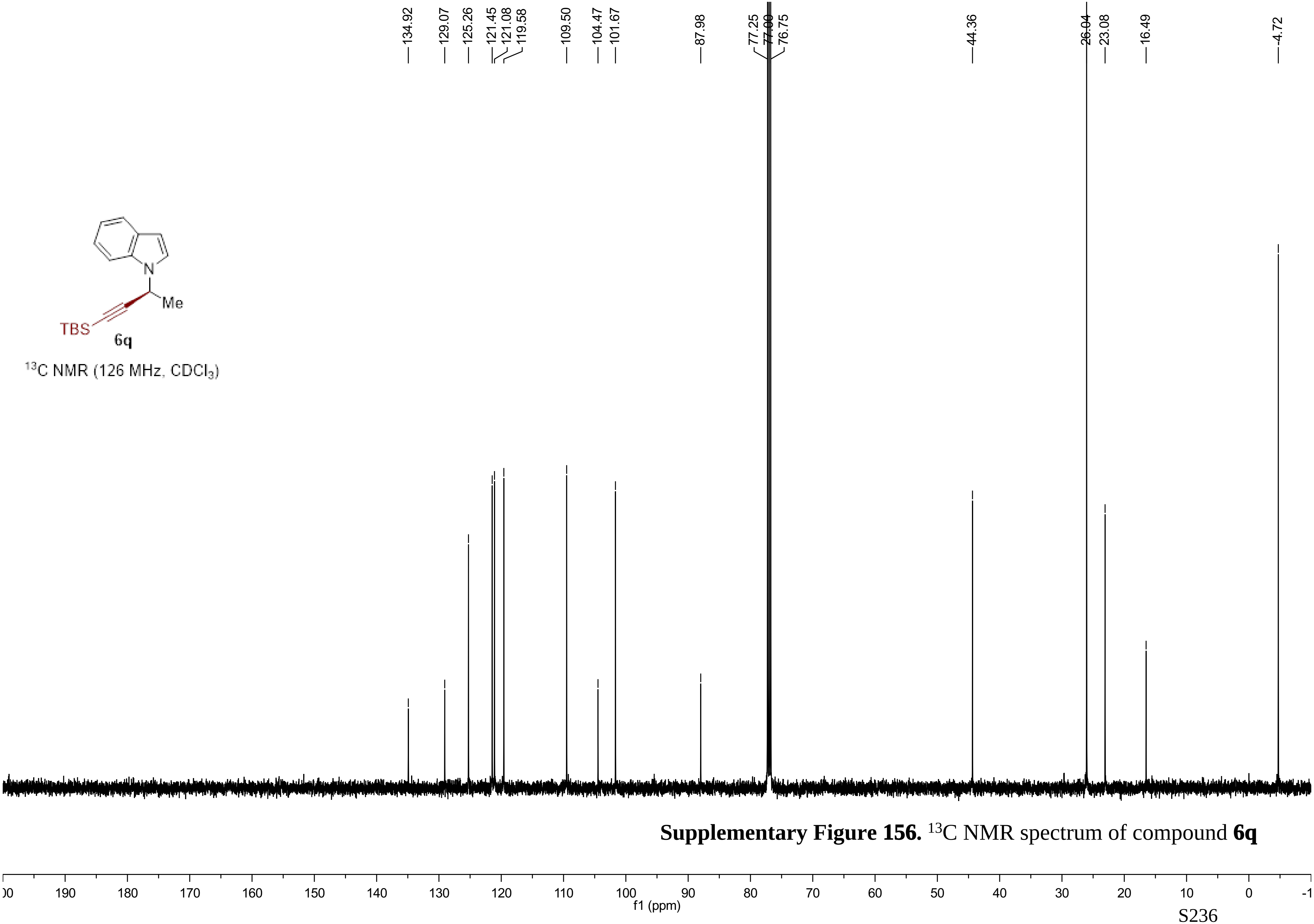

Supplementary Figure 156. ^{13}C NMR spectrum of compound **6q**

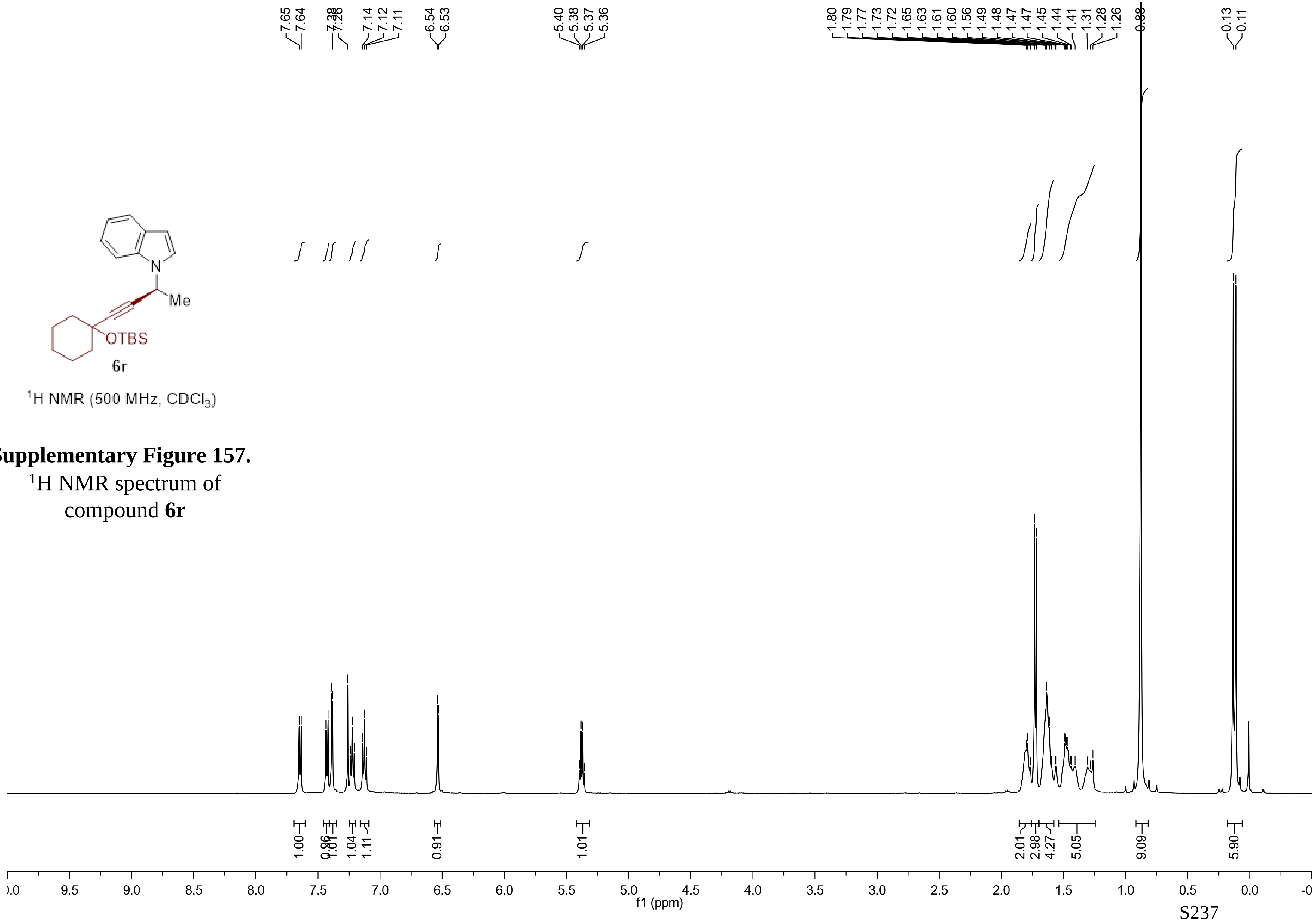

Supplementary Figure 157.
^{1}H NMR spectrum of compound **6r**

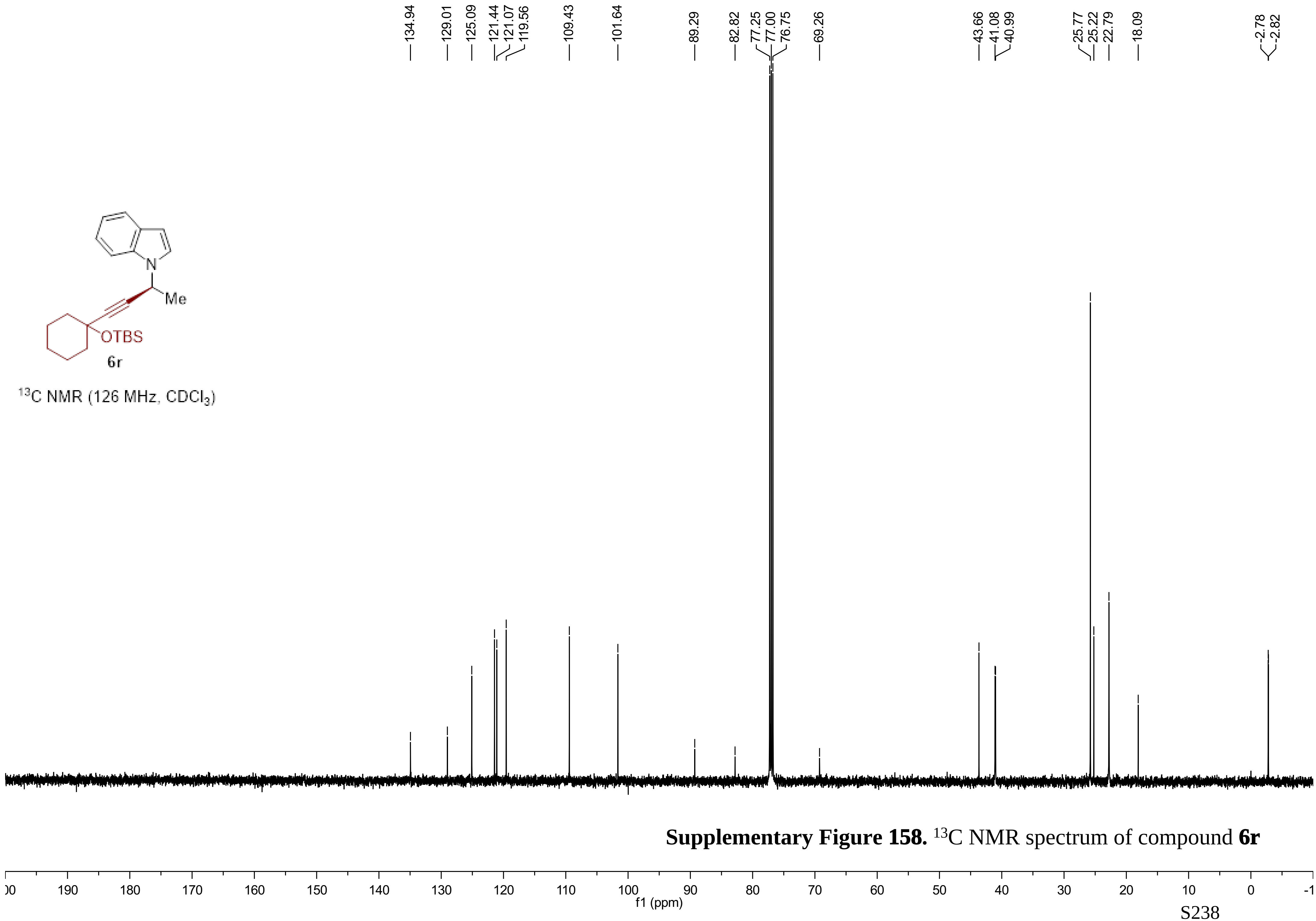

Supplementary Figure 158. ^{13}C NMR spectrum of compound **6r**

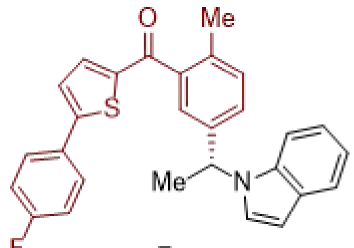

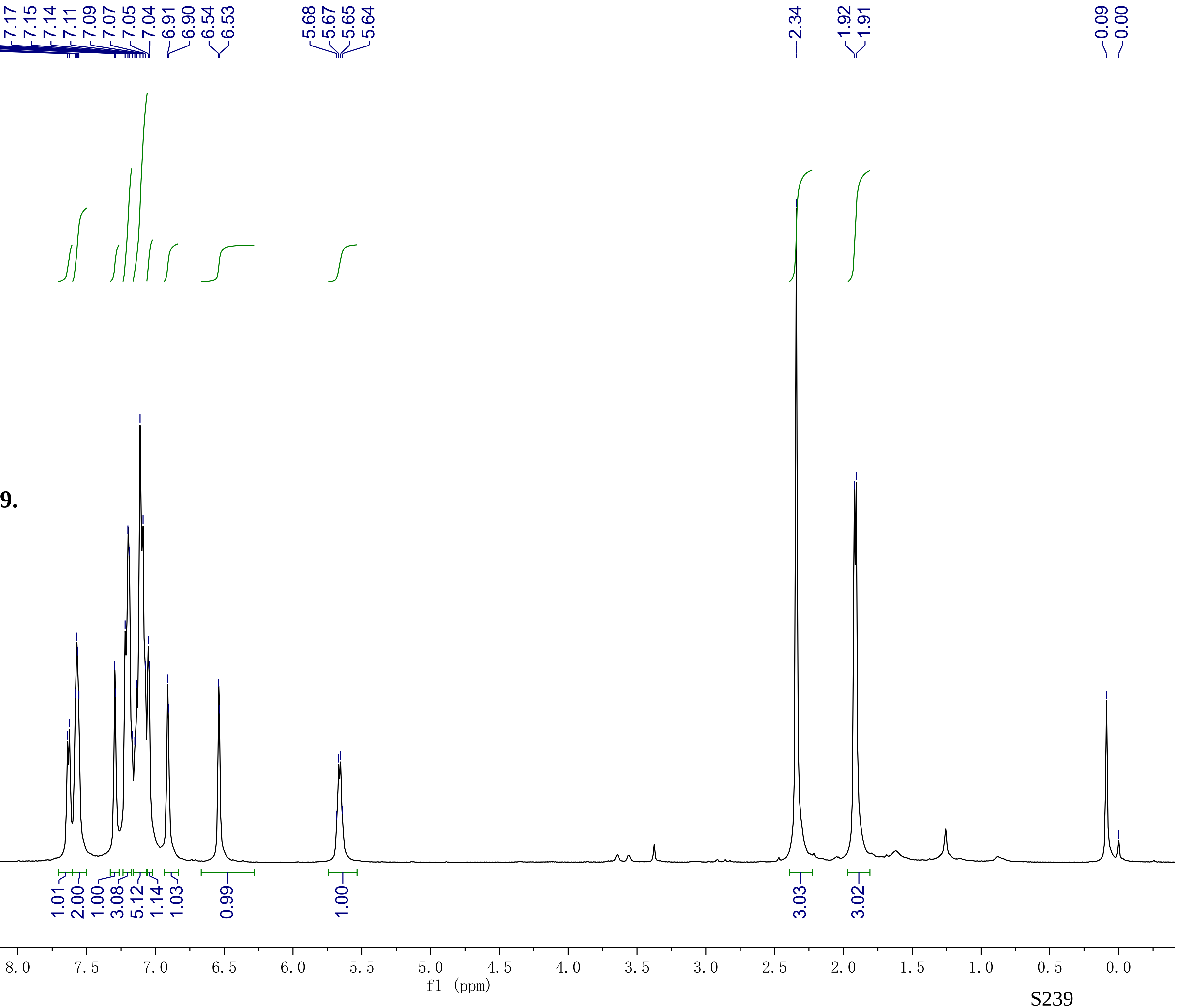

^{1}H NMR, 600 MHz, $CDCl_3$

Supplementary Figure 159. ^{1}H NMR spectrum of compound **7a**

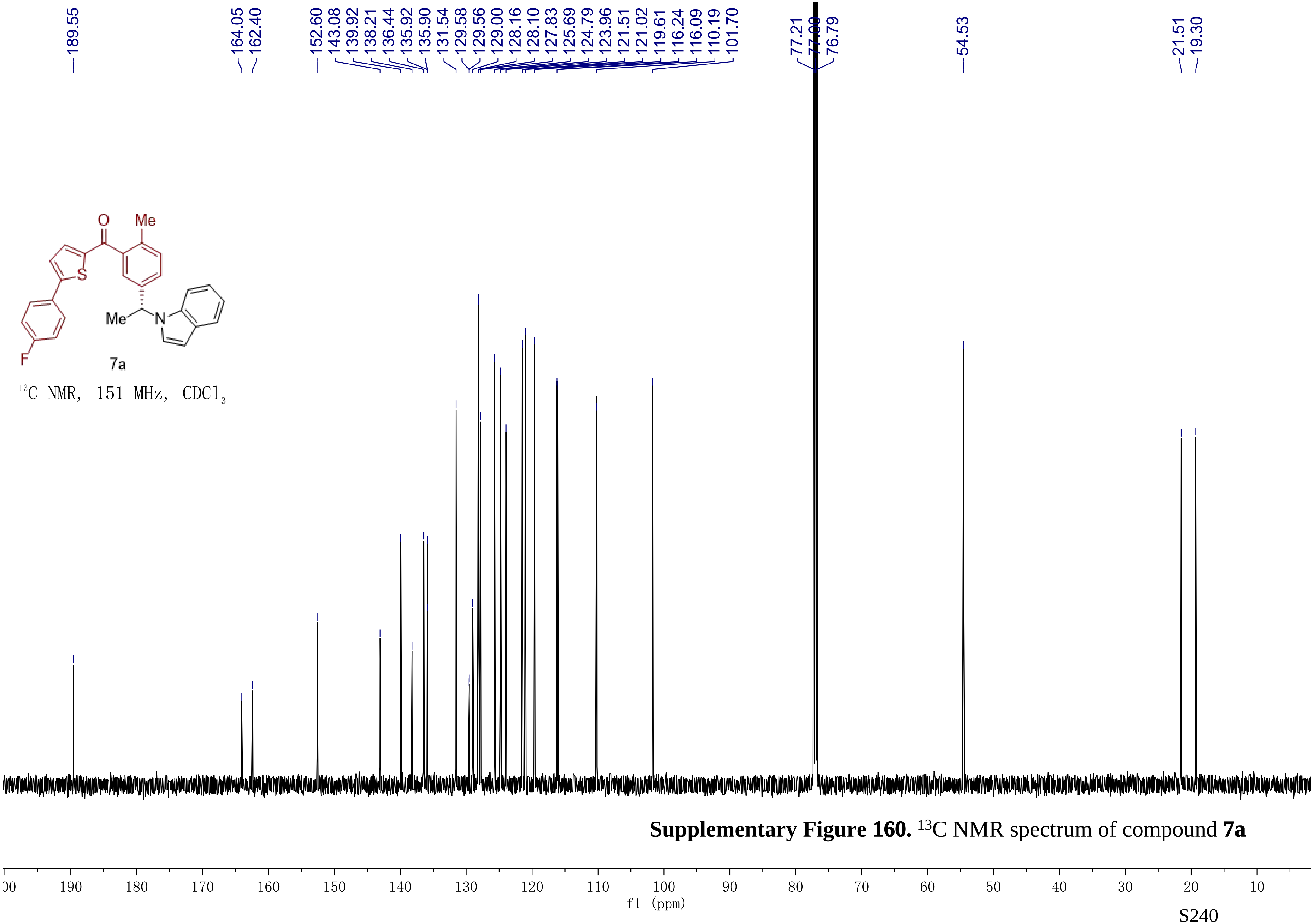

Supplementary Figure 160. ^{13}C NMR spectrum of compound **7a**

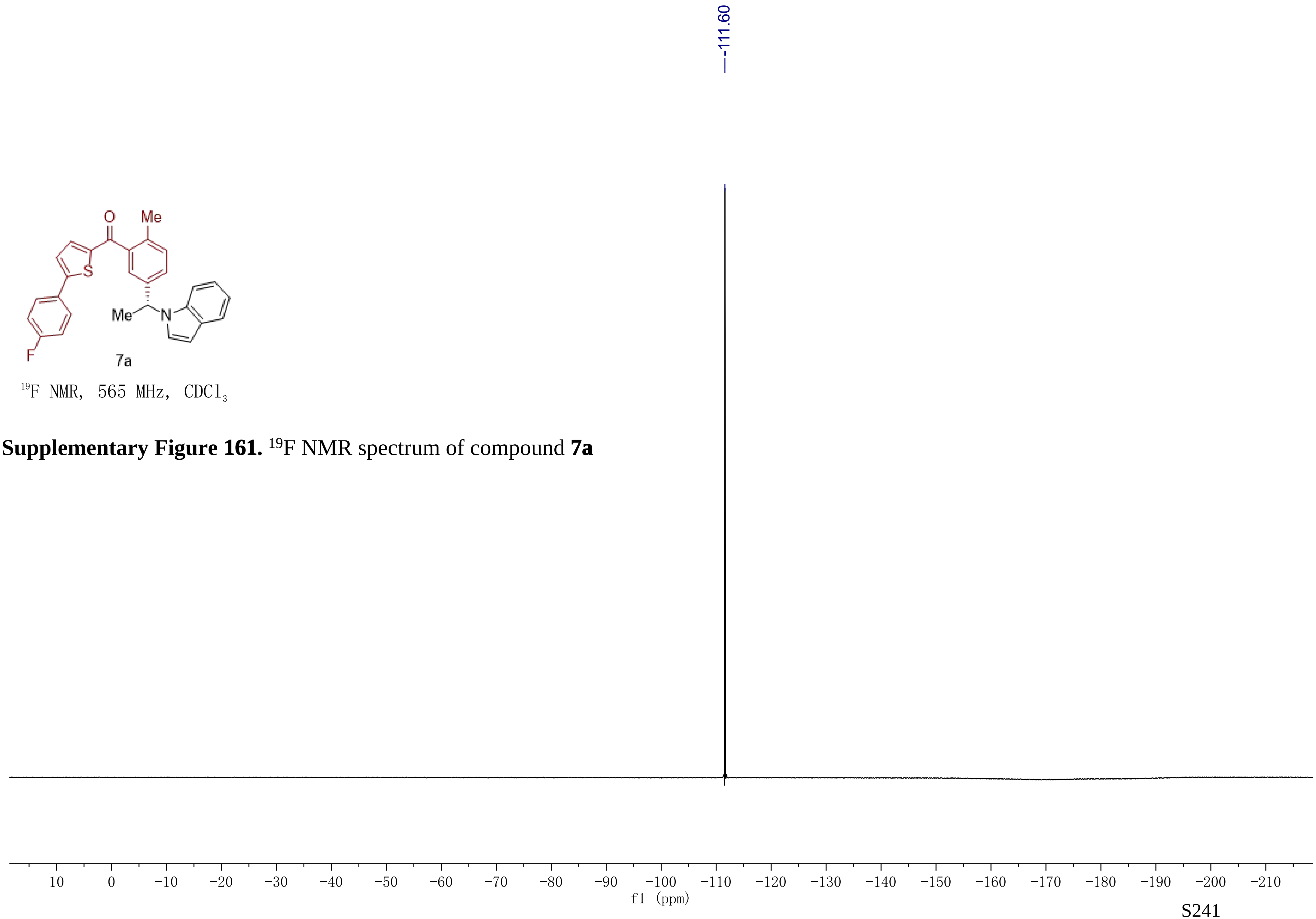

Supplementary Figure 161. ^{19}F NMR spectrum of compound **7a**

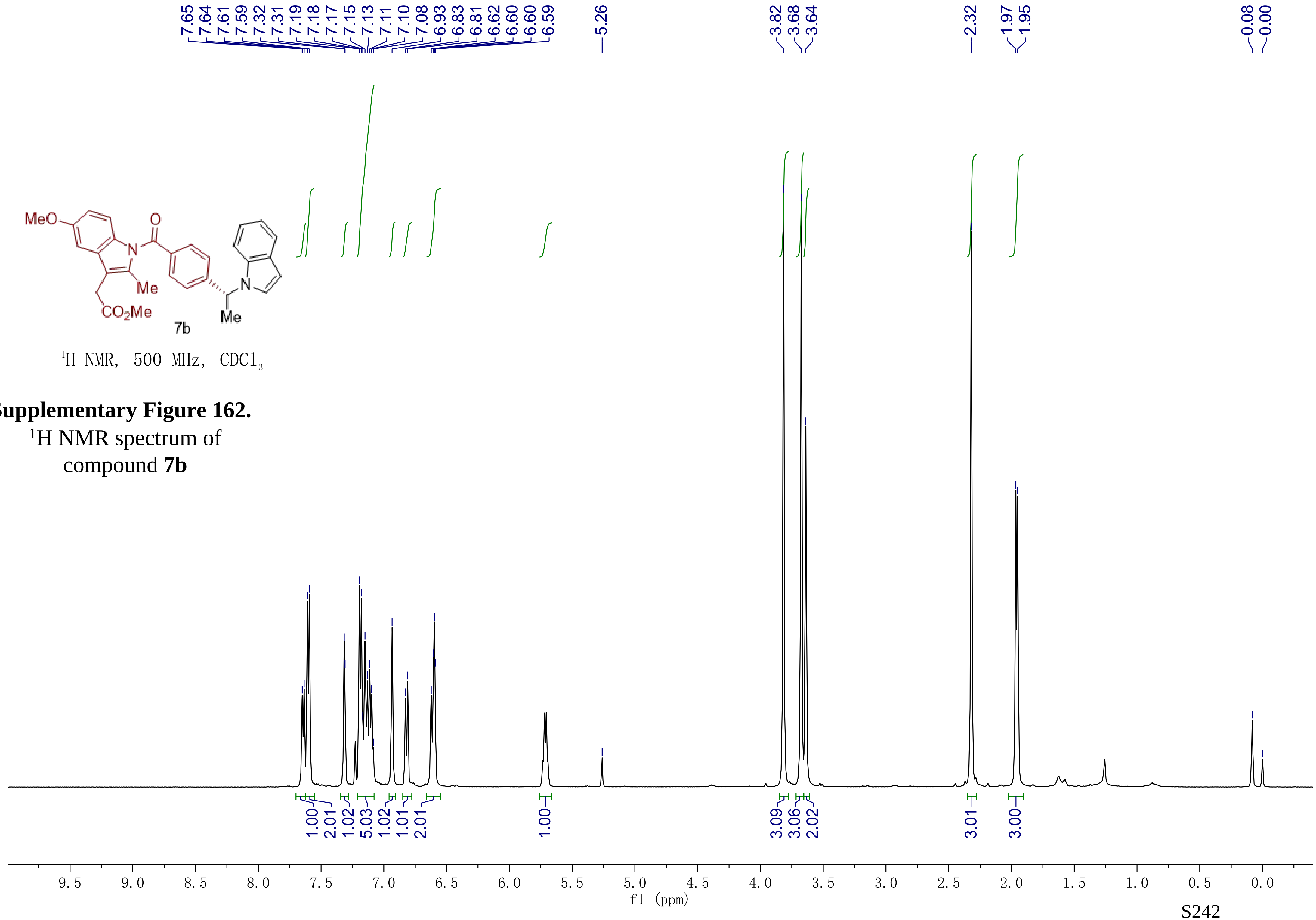

Supplementary Figure 162. ^{1}H NMR spectrum of compound **7b**

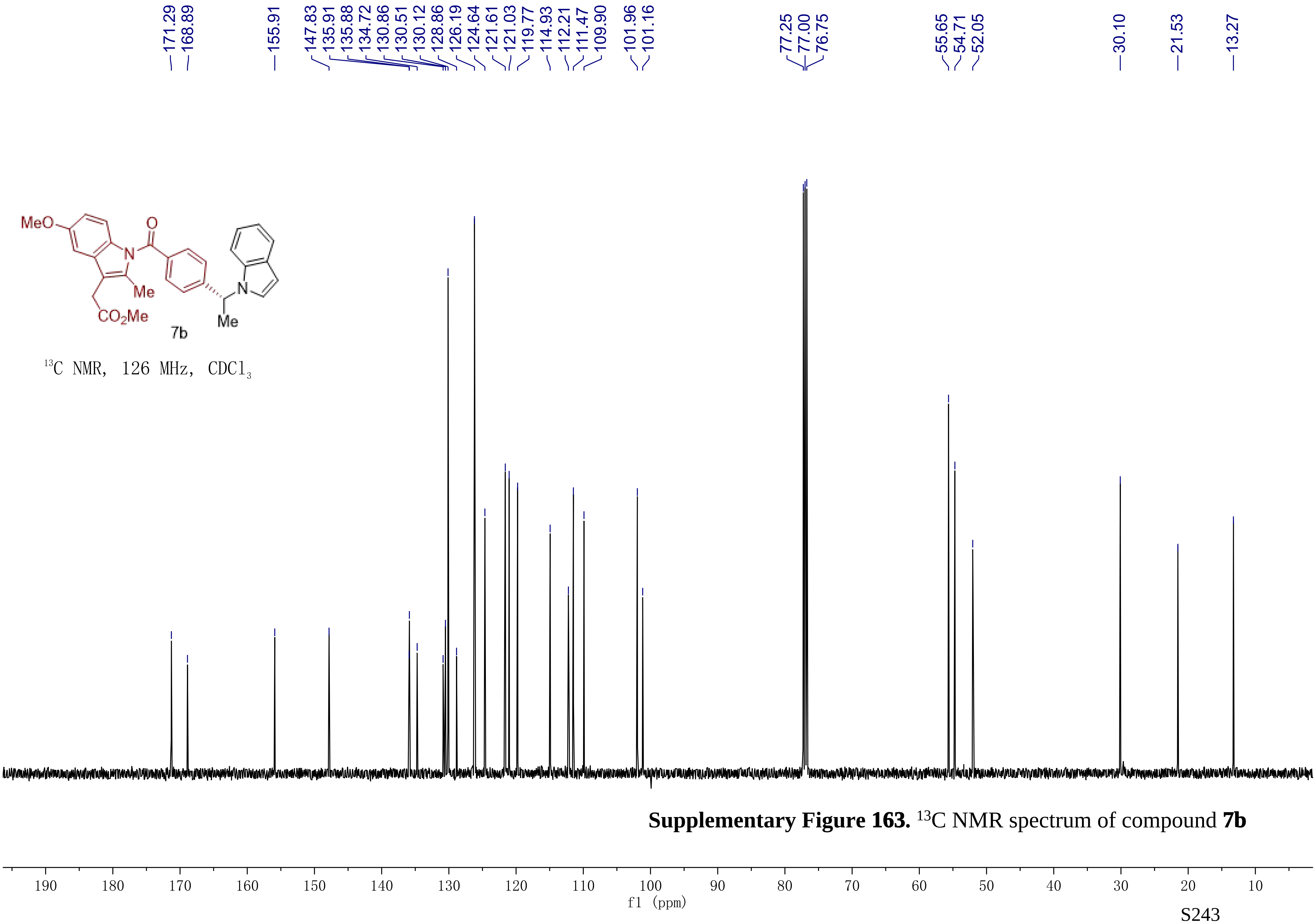

Supplementary Figure 163. ^{13}C NMR spectrum of compound **7b**

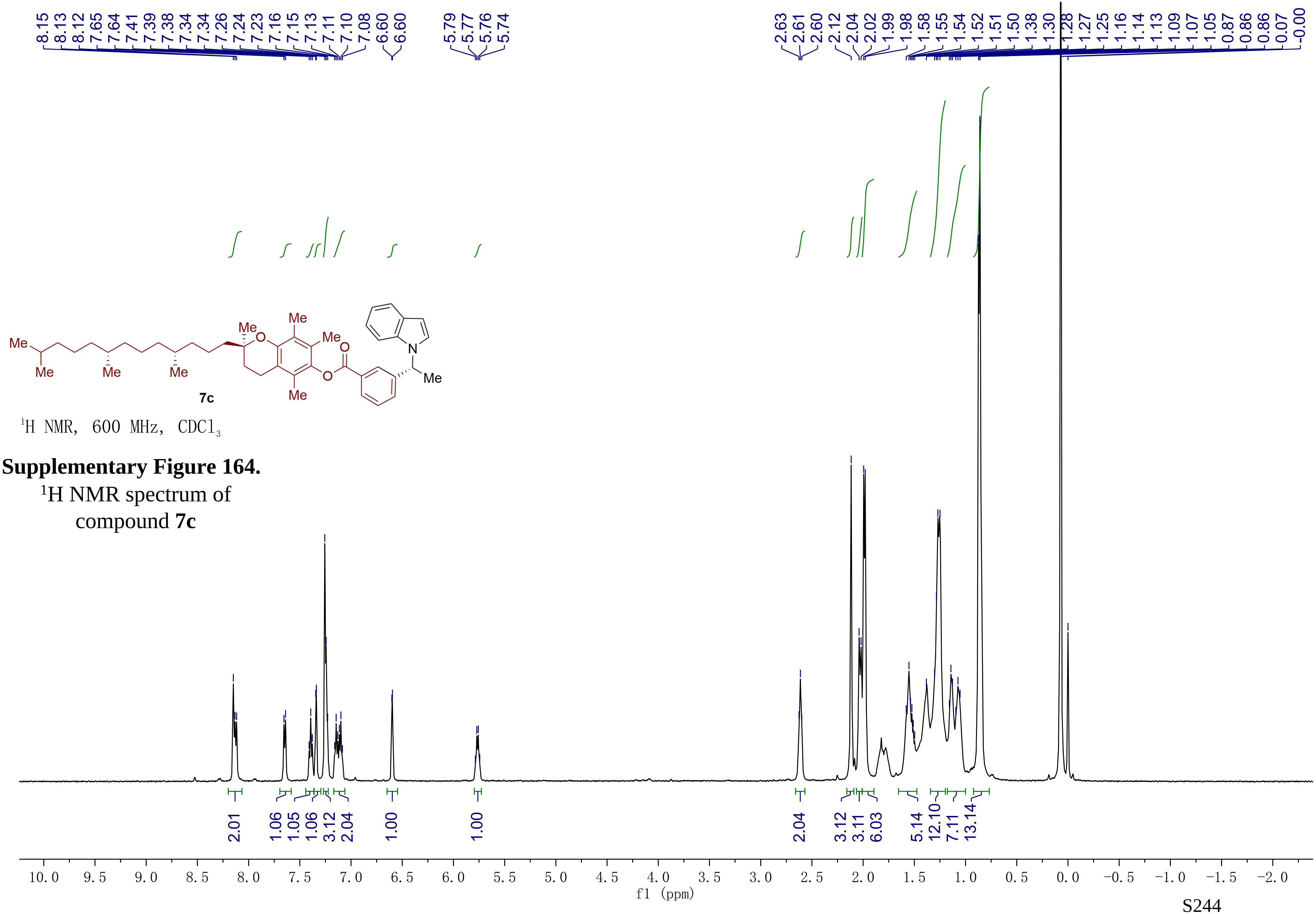

7c

^{1}H NMR, 600 MHz, $CDCl_3$

Supplementary Figure 164.
^{1}H NMR spectrum of compound **7c**

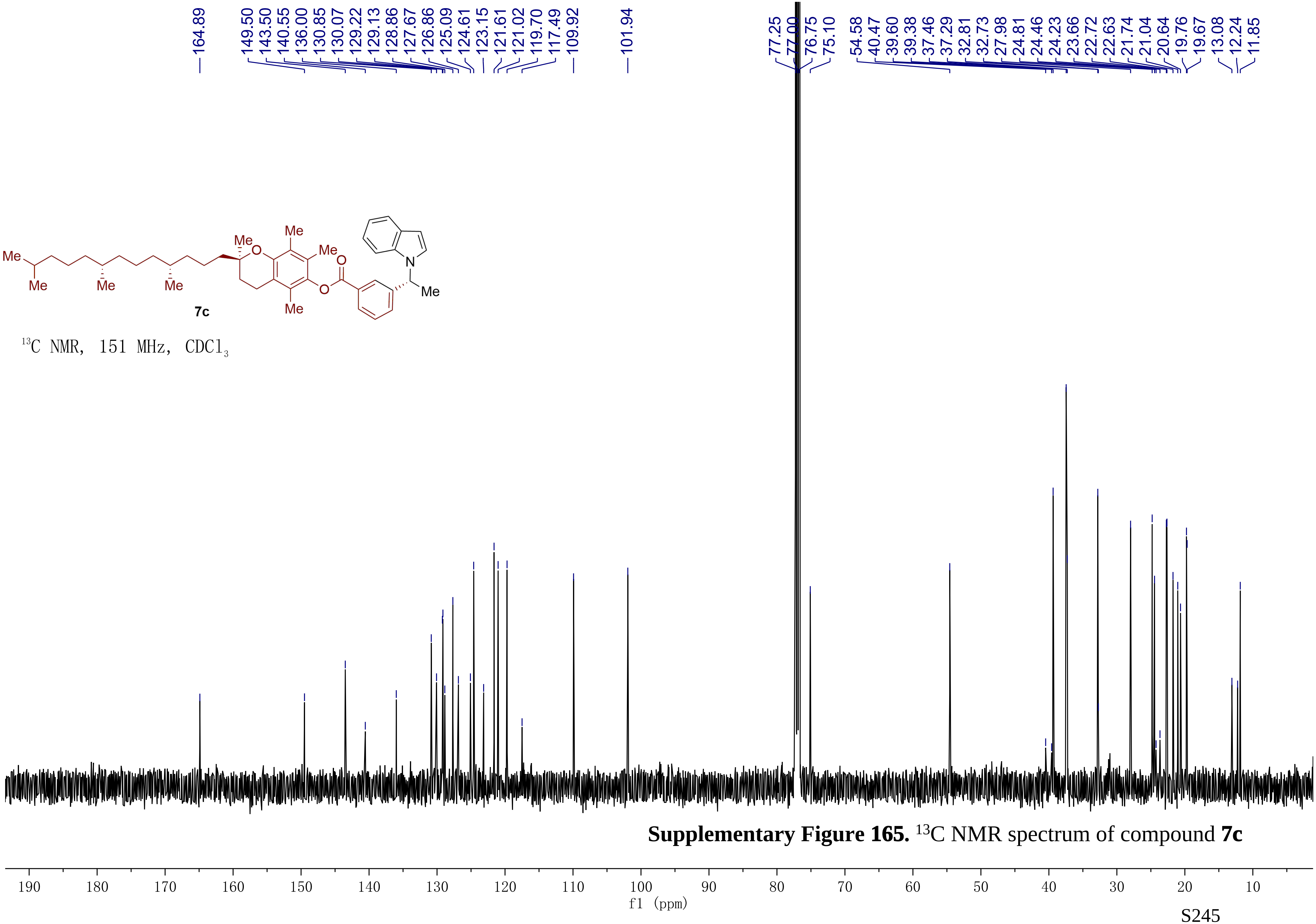

Supplementary Figure 165. ^{13}C NMR spectrum of compound **7c**

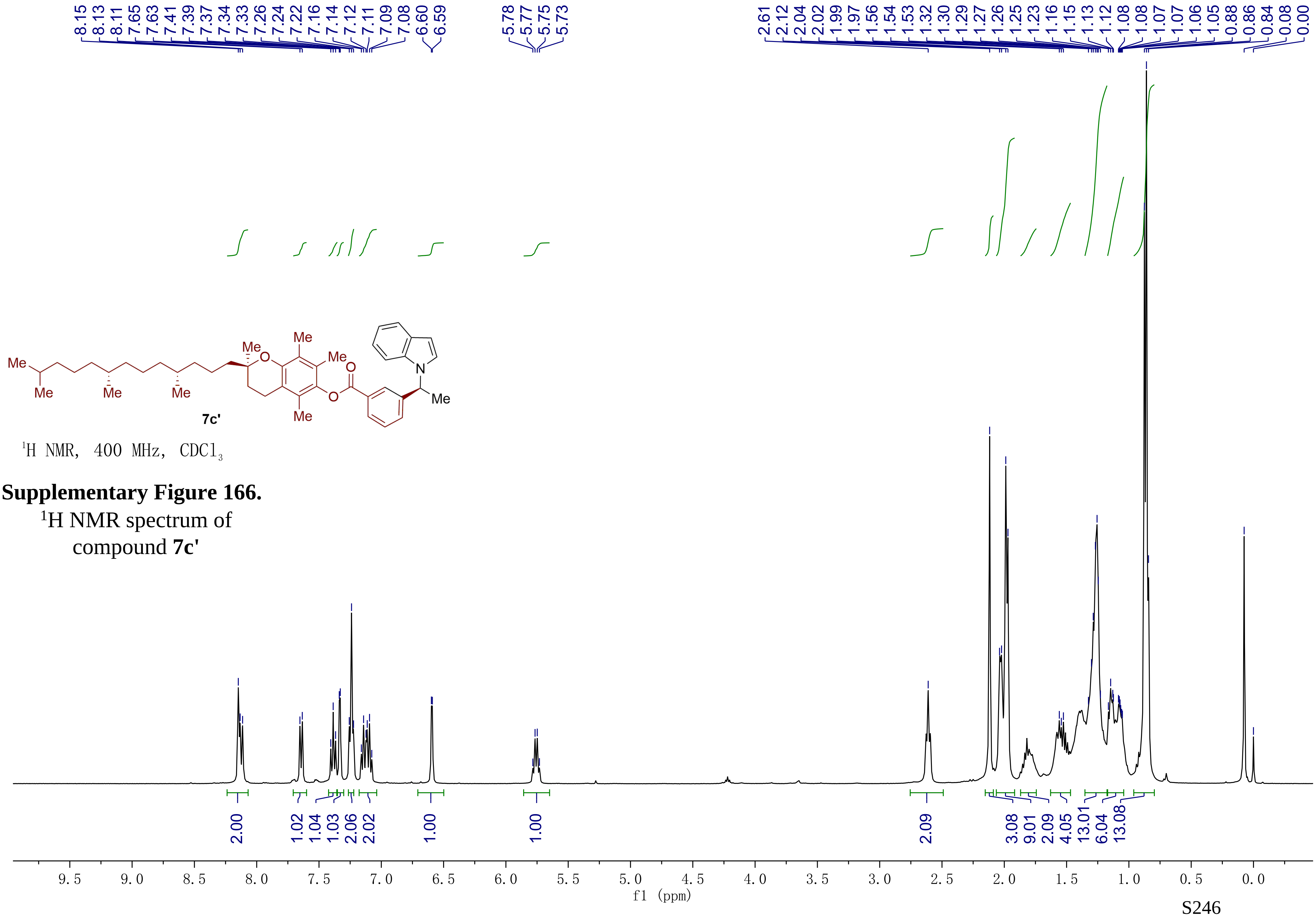

Supplementary Figure 166. ^{1}H NMR spectrum of compound **7c'**

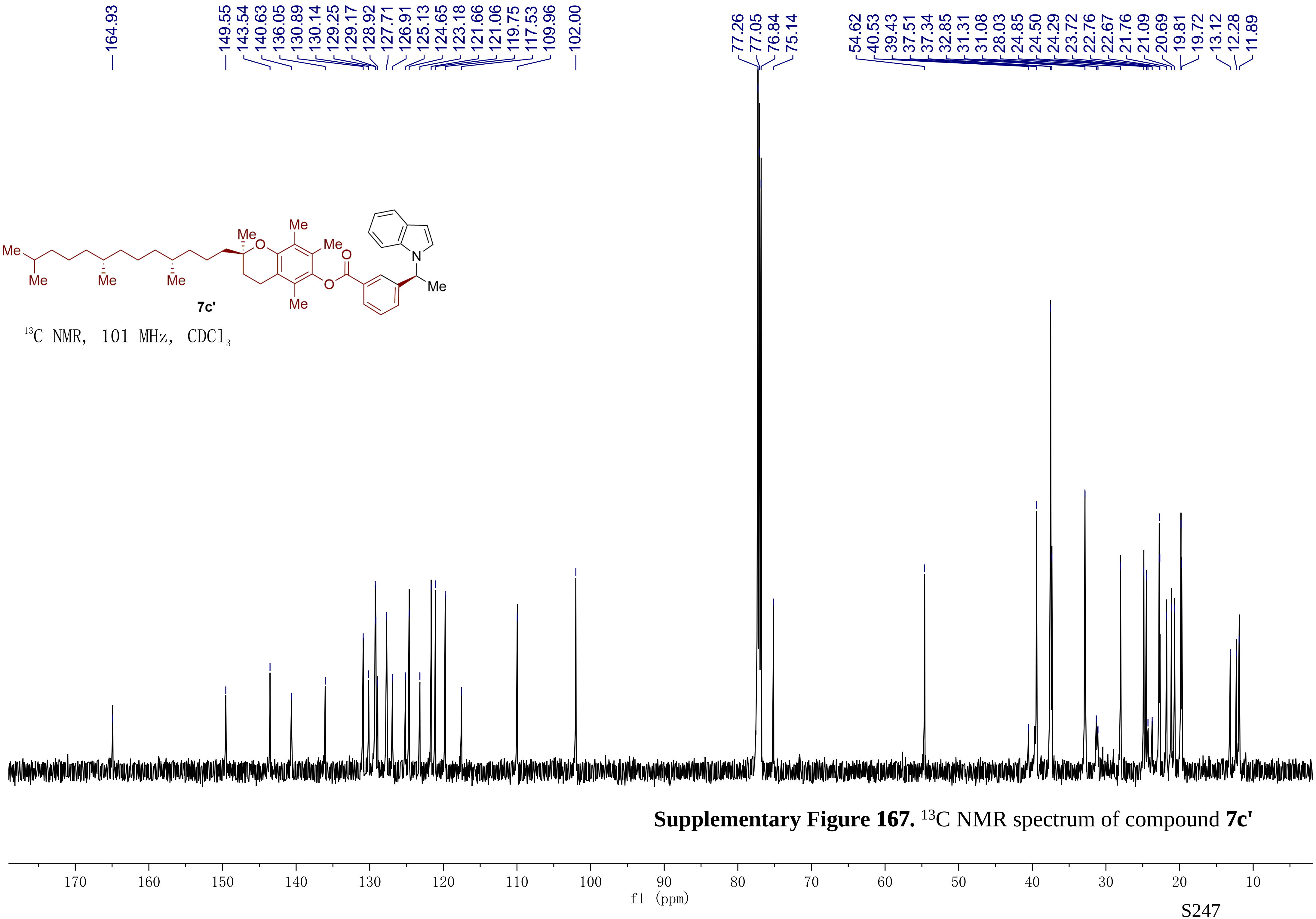

Supplementary Figure 167. ^{13}C NMR spectrum of compound **7c'**

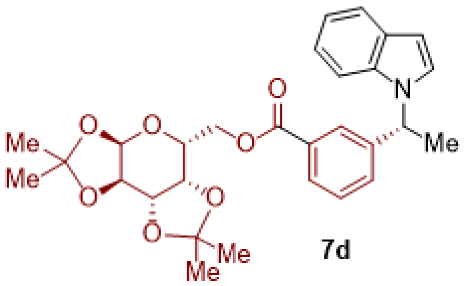

7d

¹H NMR, 500 MHz, CDCl$_3$

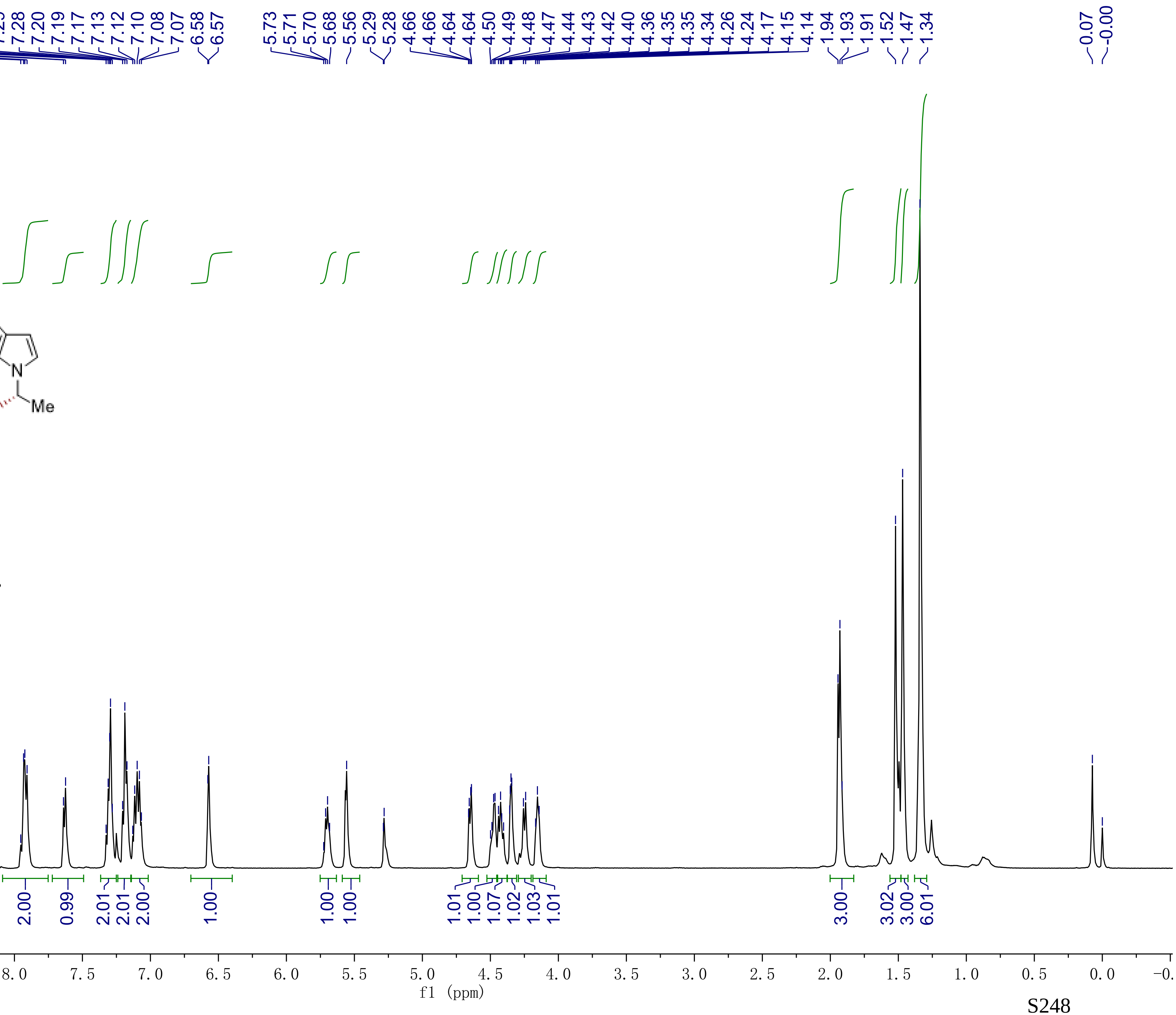

Supplementary Figure 168. ¹H NMR spectrum of compound **7d**

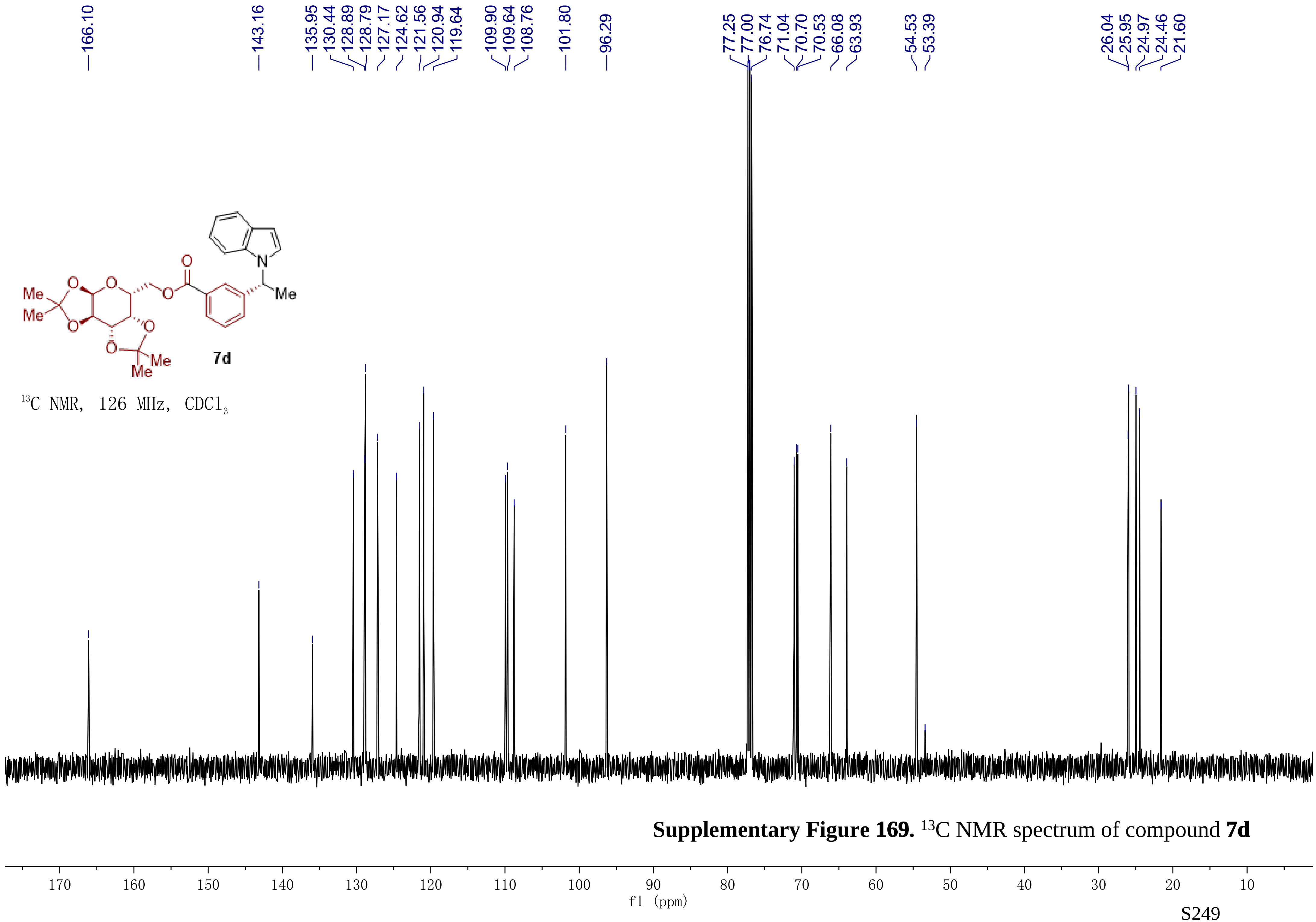

Supplementary Figure 169. ^{13}C NMR spectrum of compound **7d**

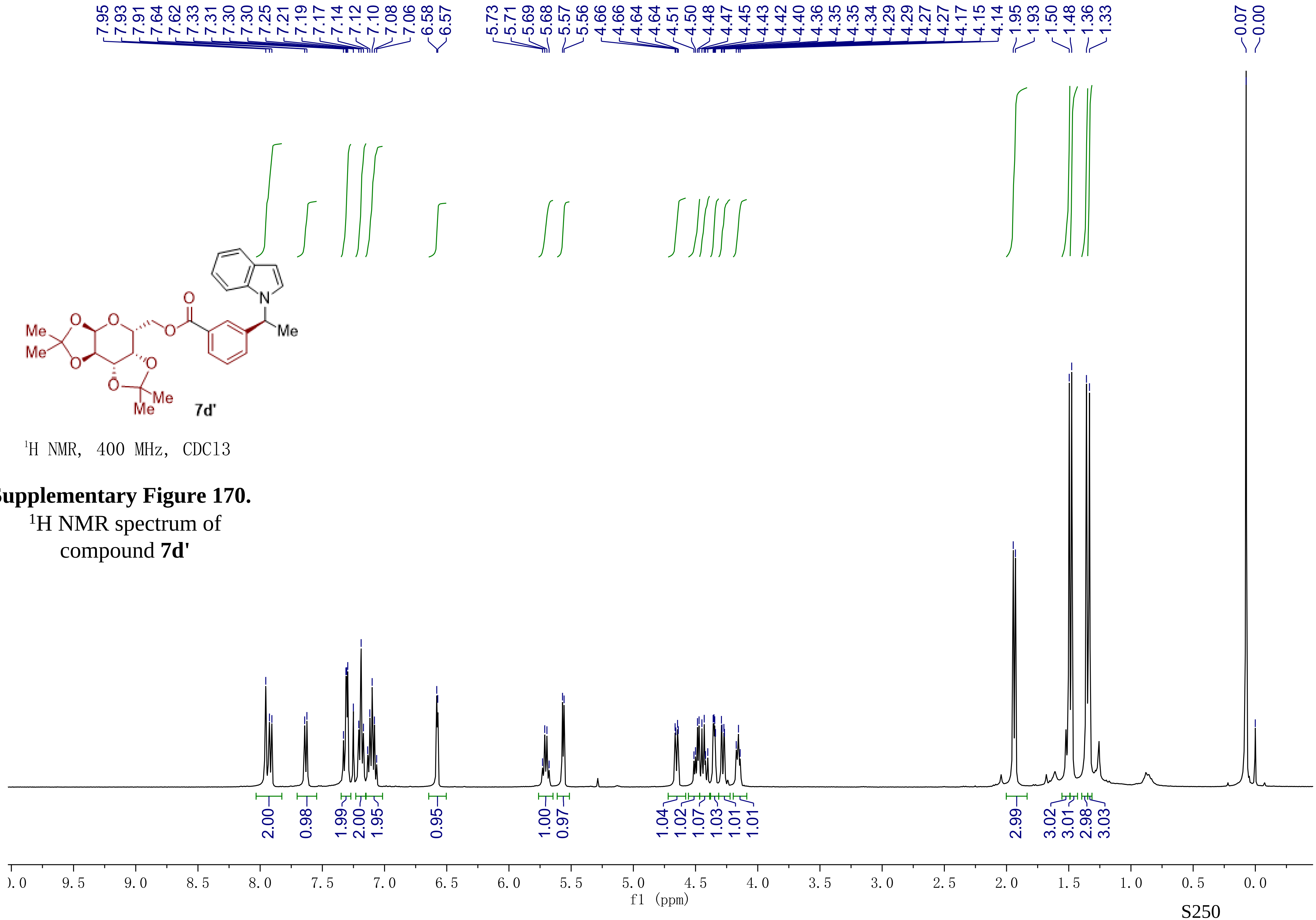

Supplementary Figure 170.
^{1}H NMR spectrum of compound **7d'**

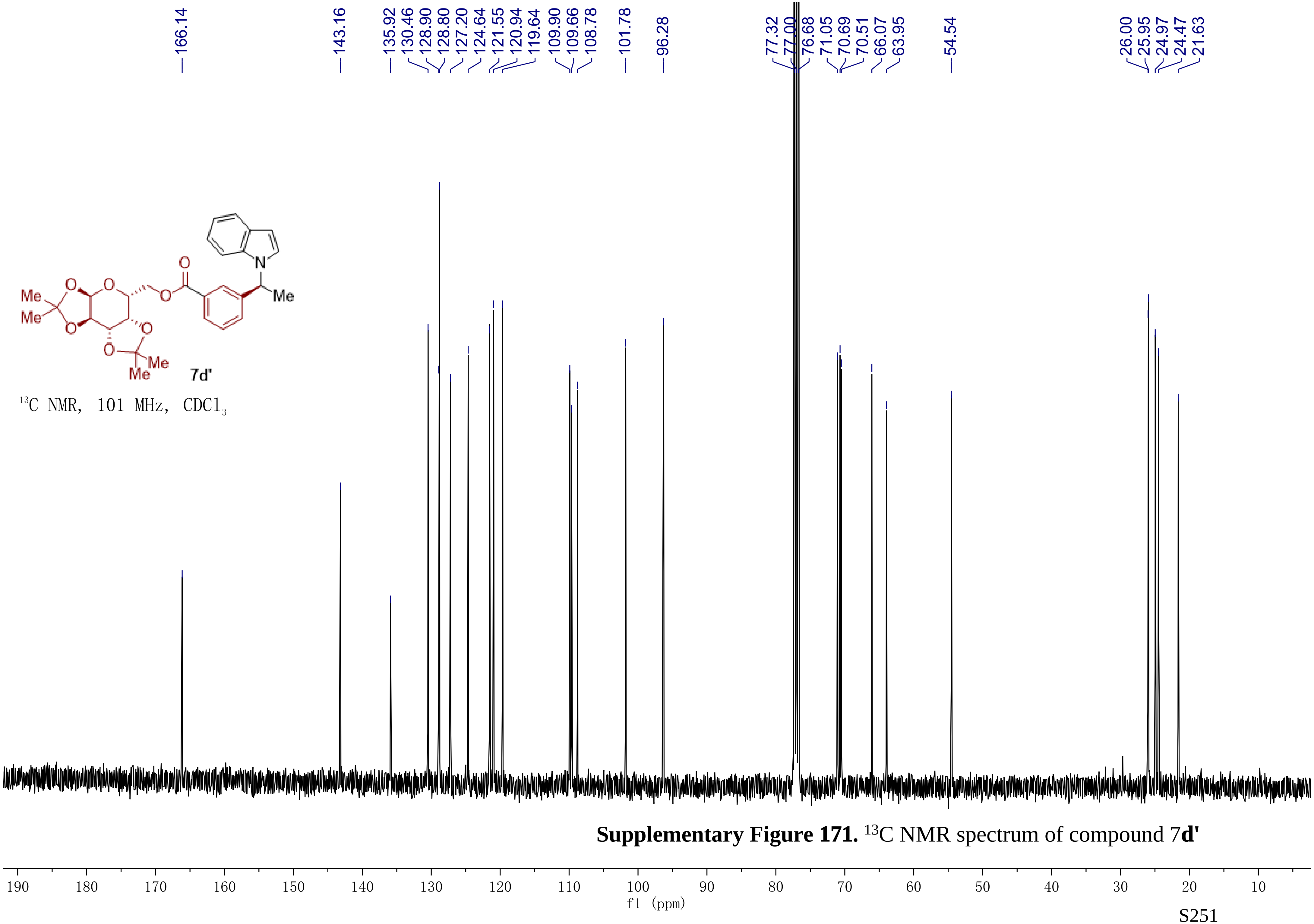

Supplementary Figure 171. ^{13}C NMR spectrum of compound 7**d'**

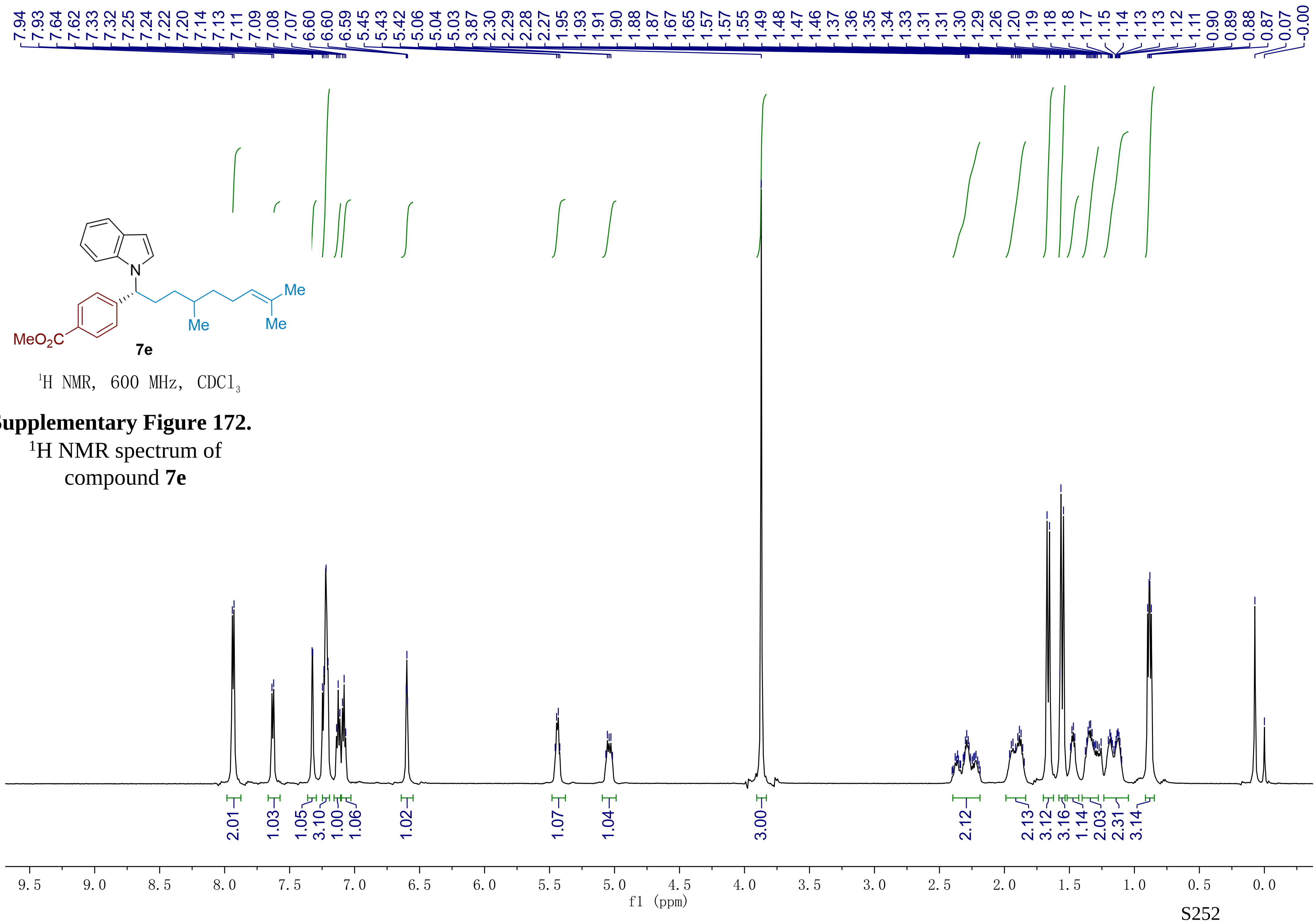

Supplementary Figure 172. ^{1}H NMR spectrum of compound **7e**

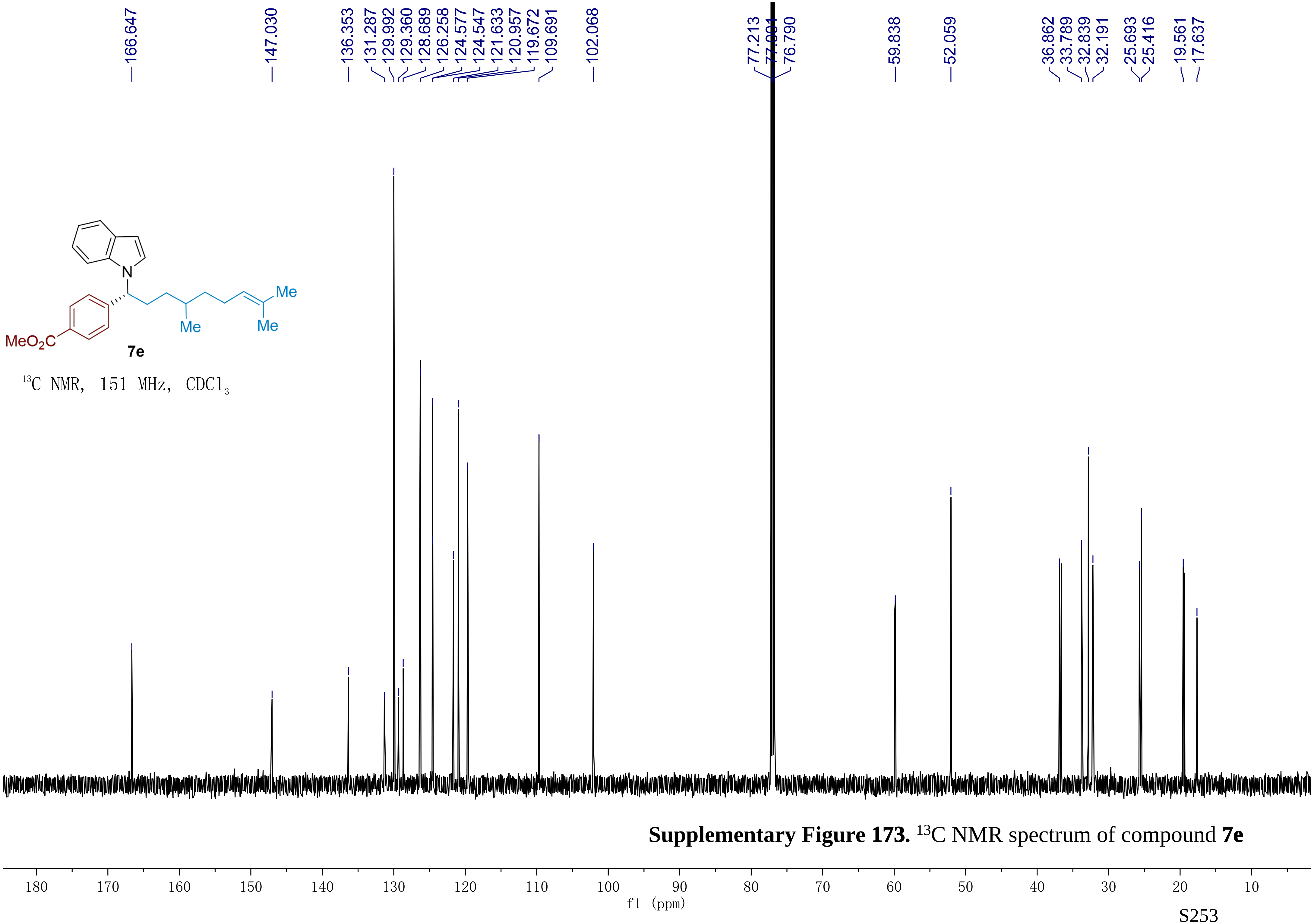

Supplementary Figure 173. ^{13}C NMR spectrum of compound **7e**

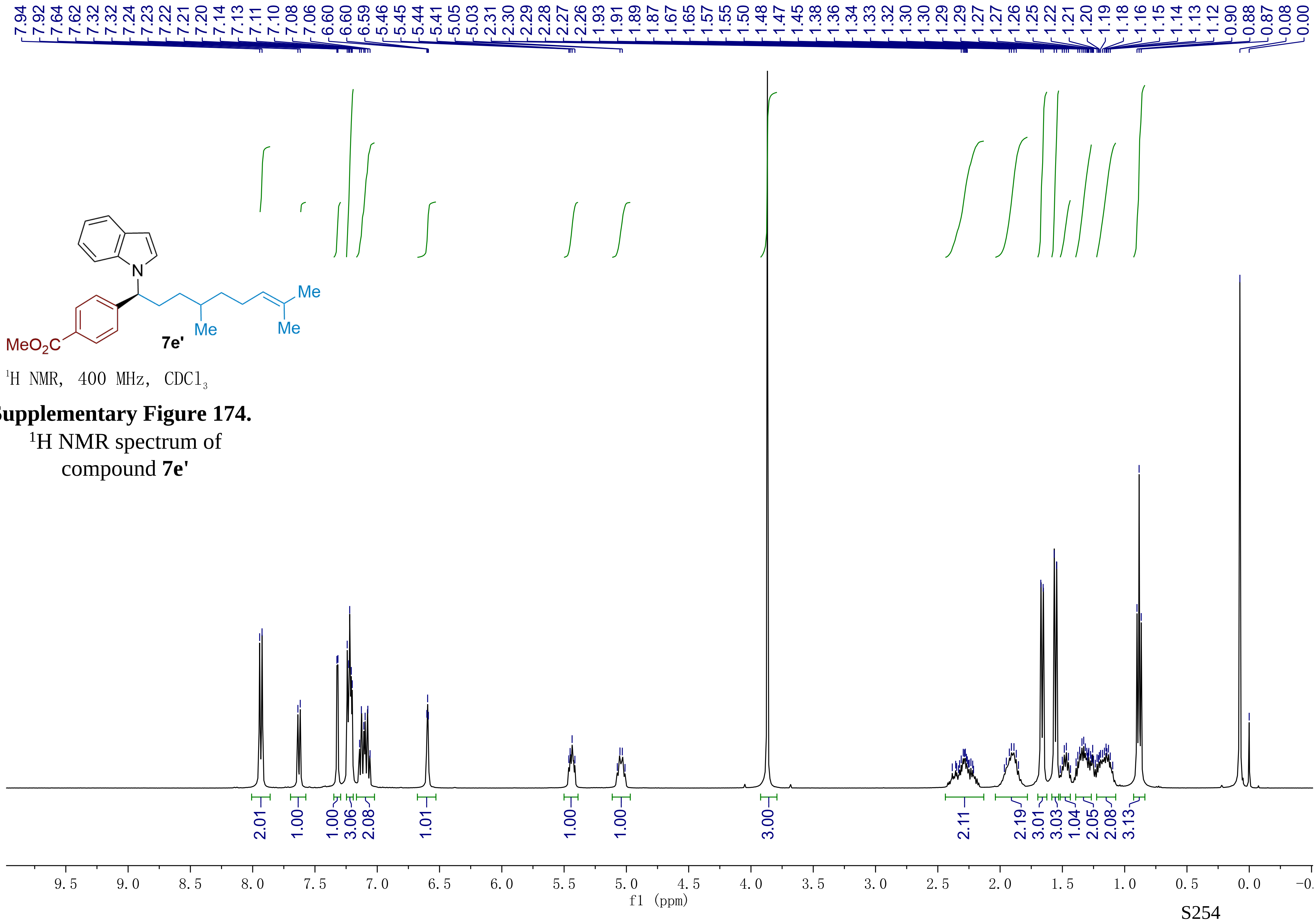

Supplementary Figure 174. ^1H NMR spectrum of compound **7e'**

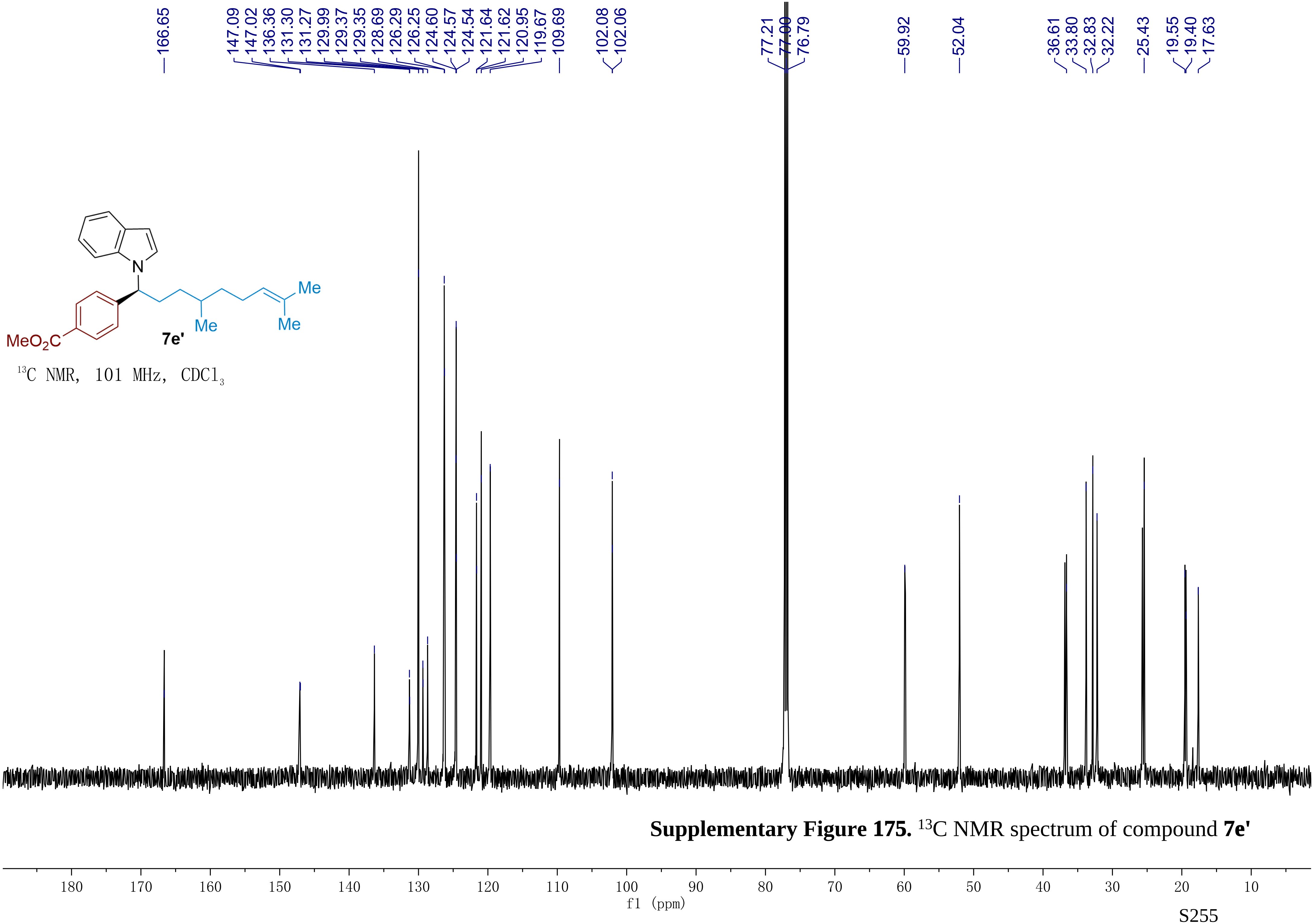

Supplementary Figure 175. ^{13}C NMR spectrum of compound **7e'**

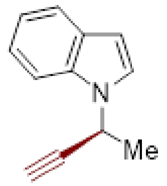

8

^{1}H NMR (400 MHz, $CDCl_3$)

Supplementary Figure 176. ^{1}H NMR spectrum of compound **8**

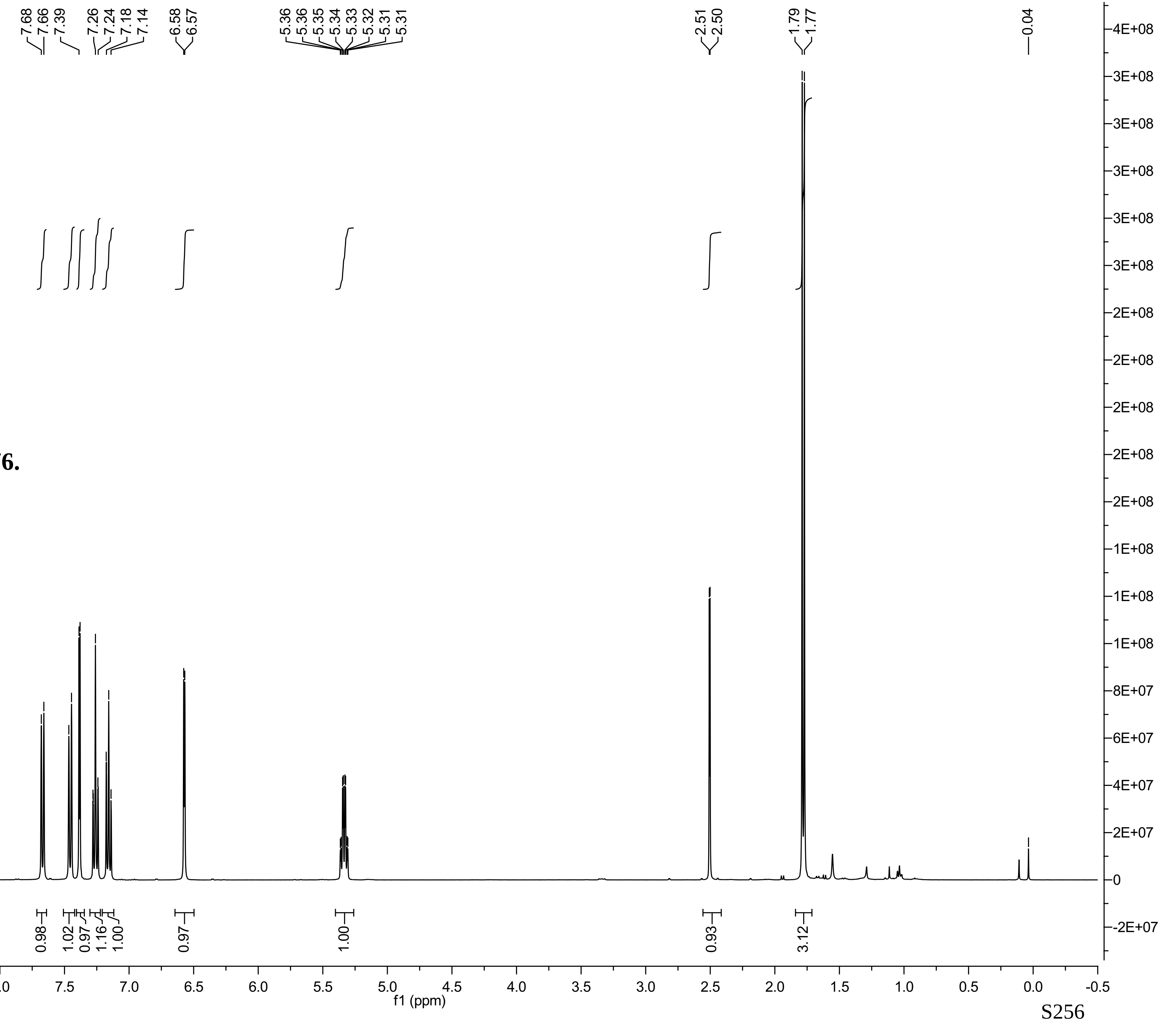

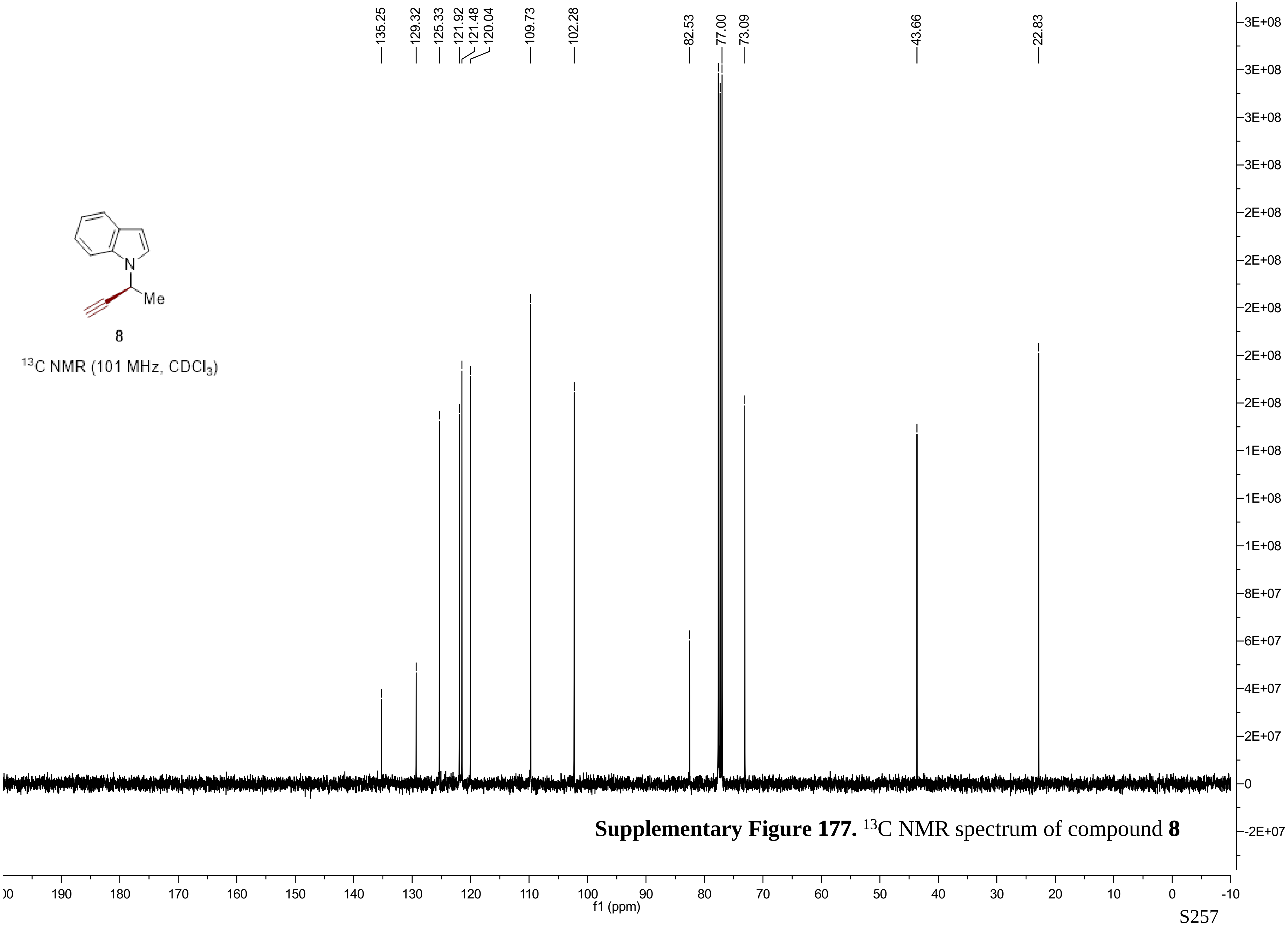

Supplementary Figure 177. ^{13}C NMR spectrum of compound **8**

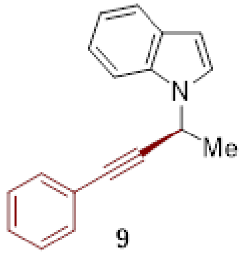

9

^{1}H NMR (500 MHz, $CDCl_3$)

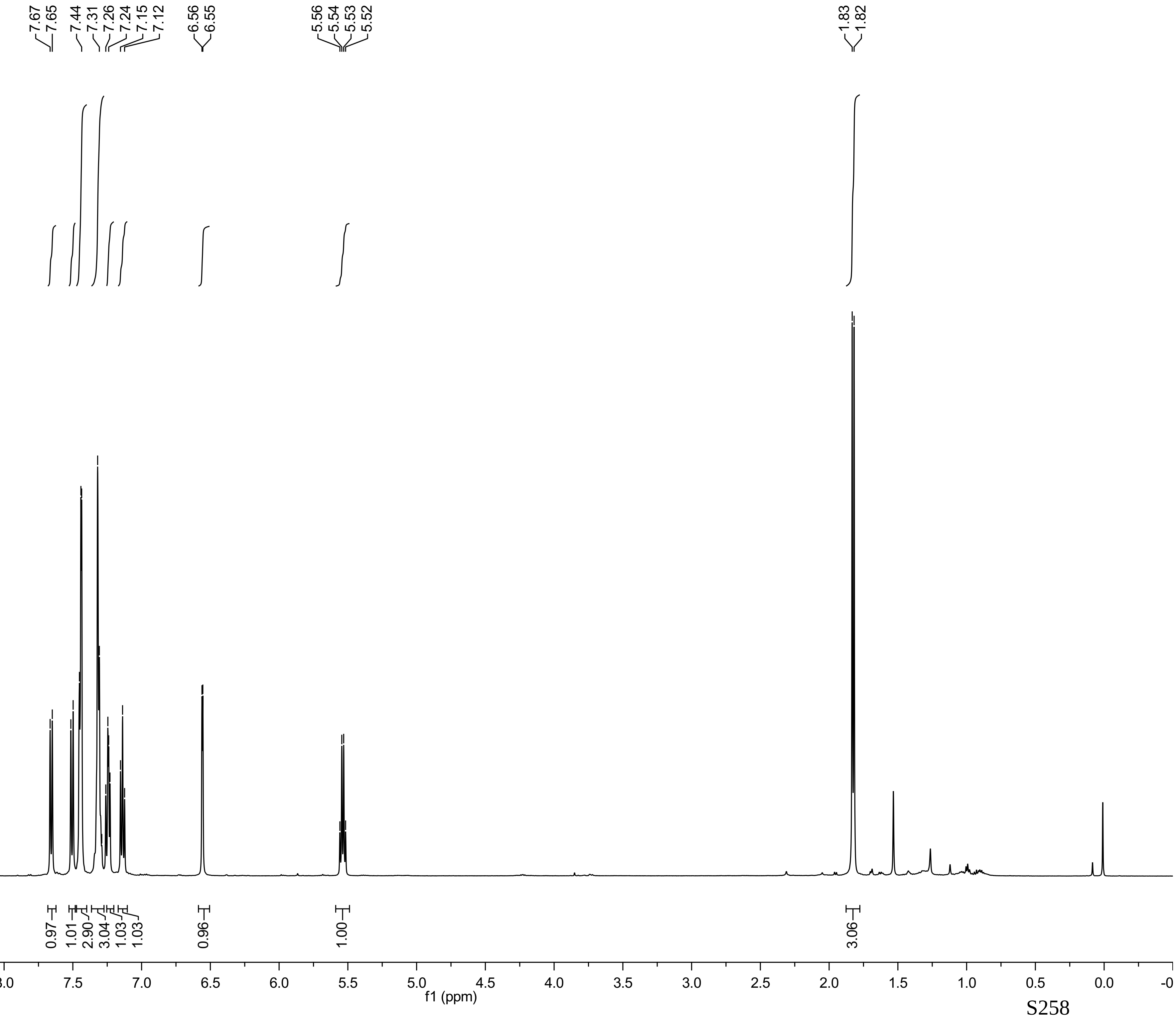

Supplementary Figure 178. ^{1}H NMR spectrum of compound **9**

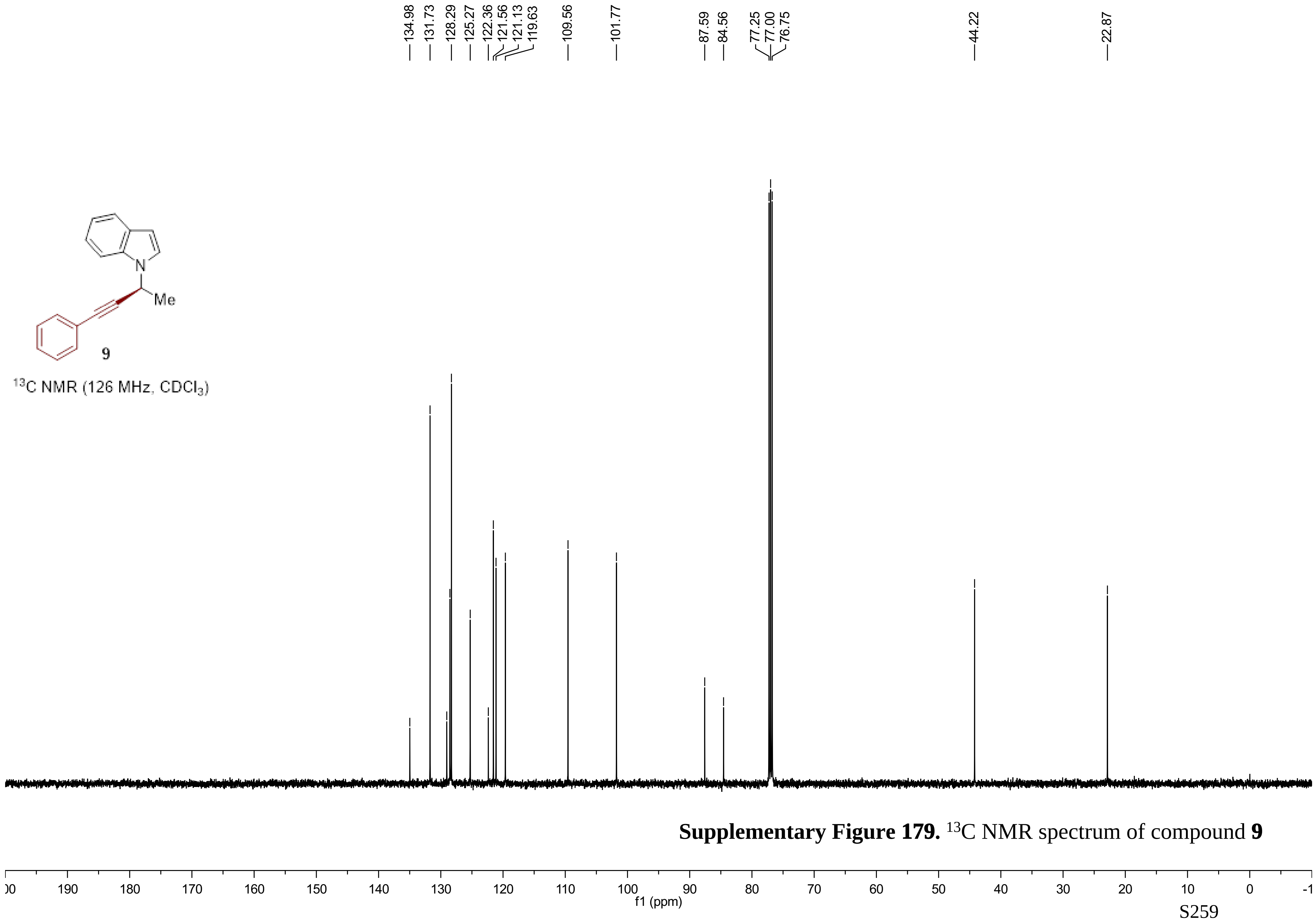

Supplementary Figure 179. ^{13}C NMR spectrum of compound **9**

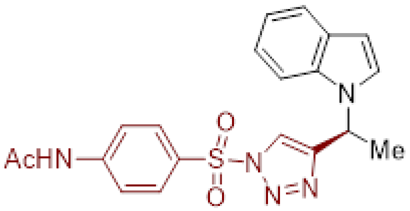

10

^{1}H NMR (400 MHz, $CDCl_3$)

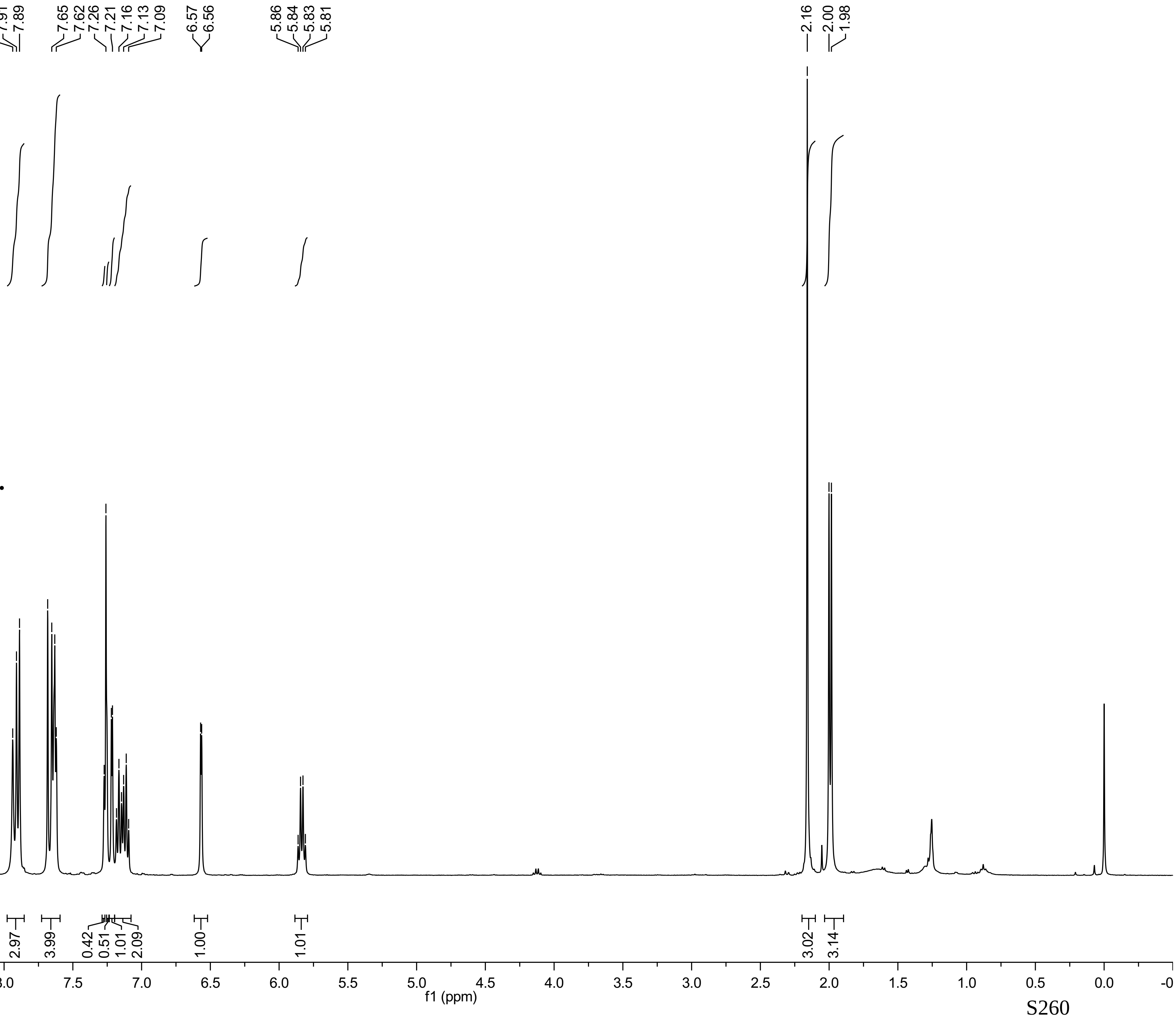

Supplementary Figure 180. ^{1}H NMR spectrum of compound **10**

10

^{13}C NMR (101 MHz, $CDCl_3$)

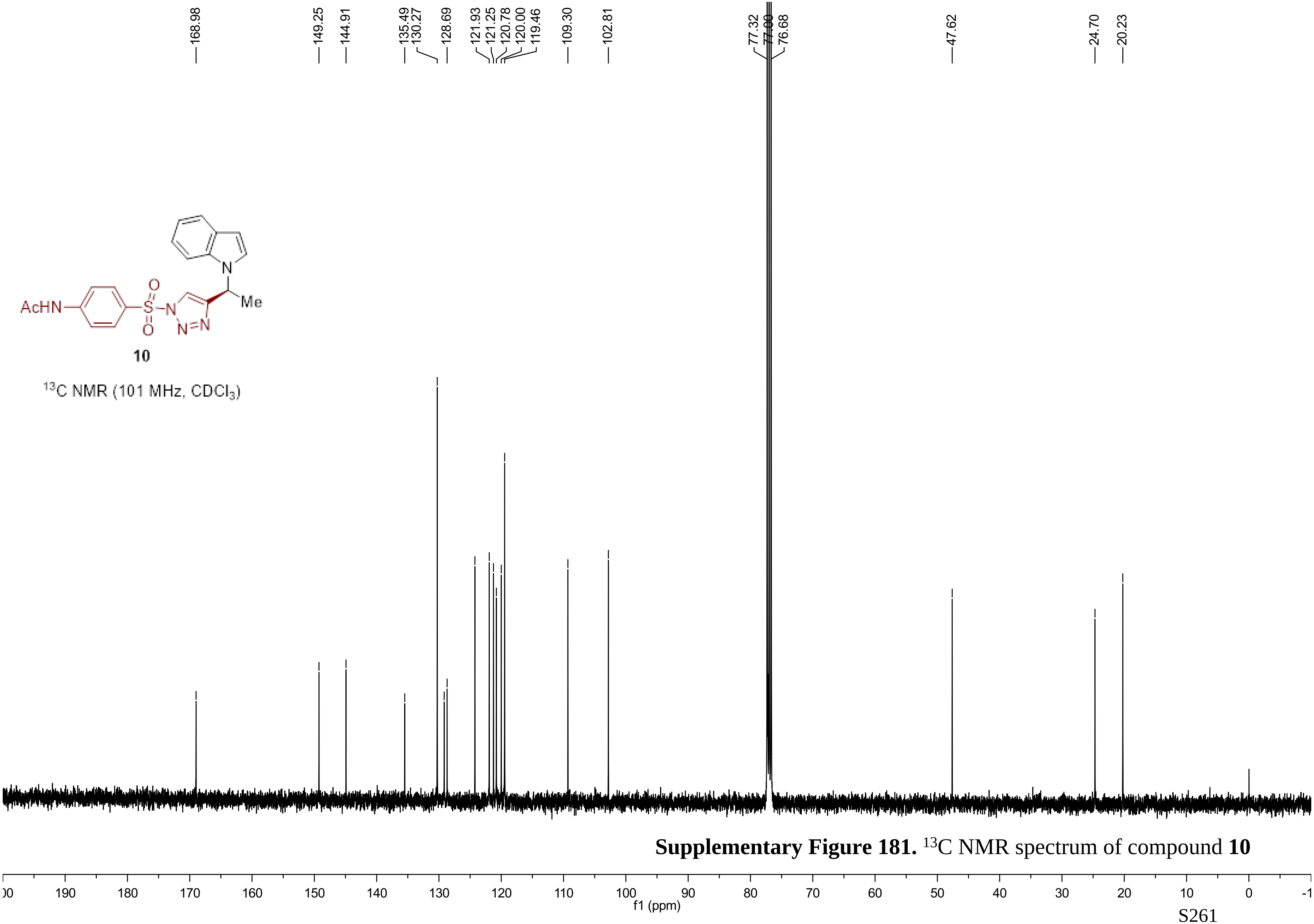

Supplementary Figure 181. ^{13}C NMR spectrum of compound **10**

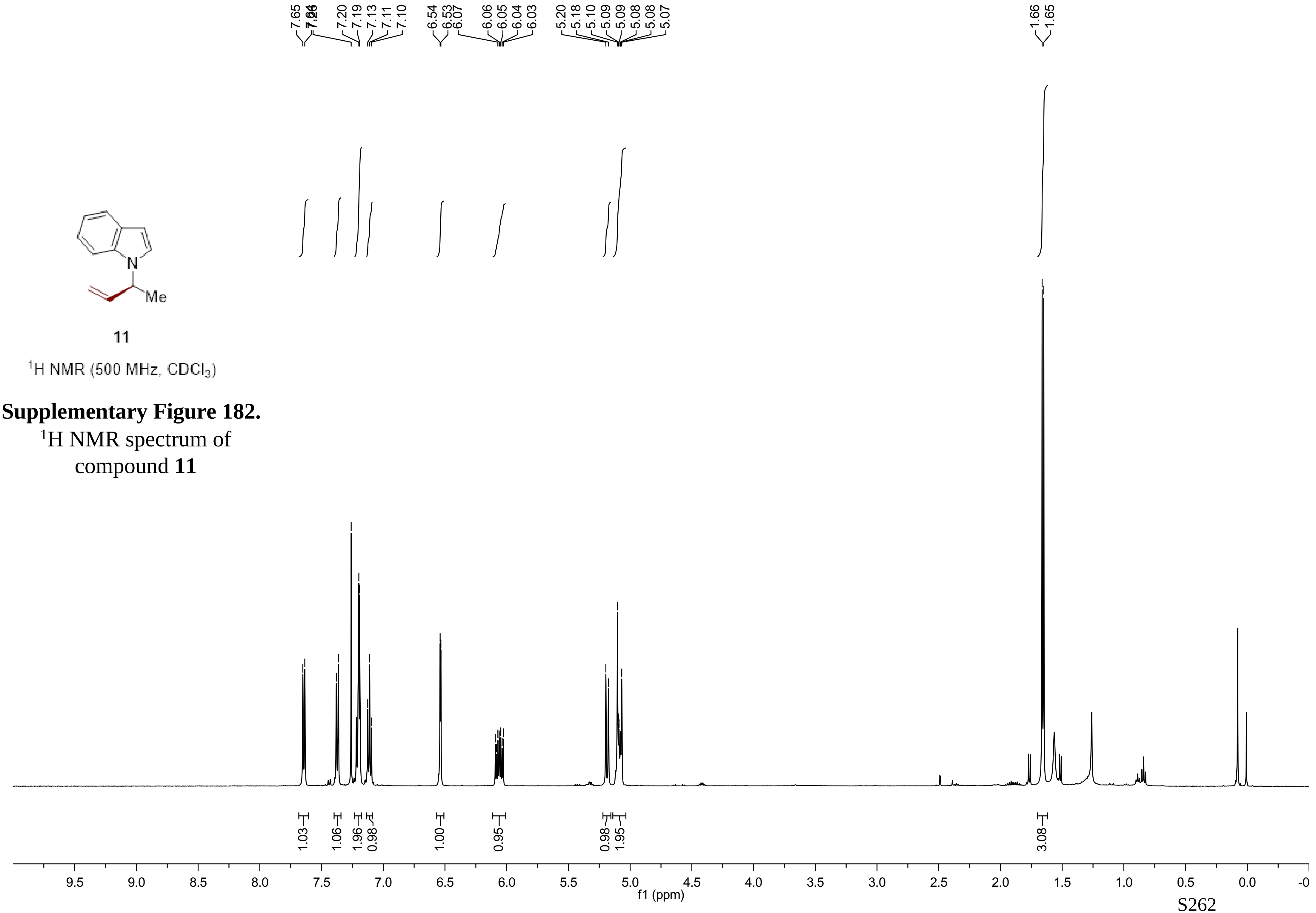

11

^{1}H NMR (500 MHz, $CDCl_3$)

Supplementary Figure 182. ^{1}H NMR spectrum of compound **11**

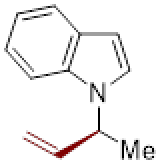

11

^{13}C NMR (126 MHz, $CDCl_3$)

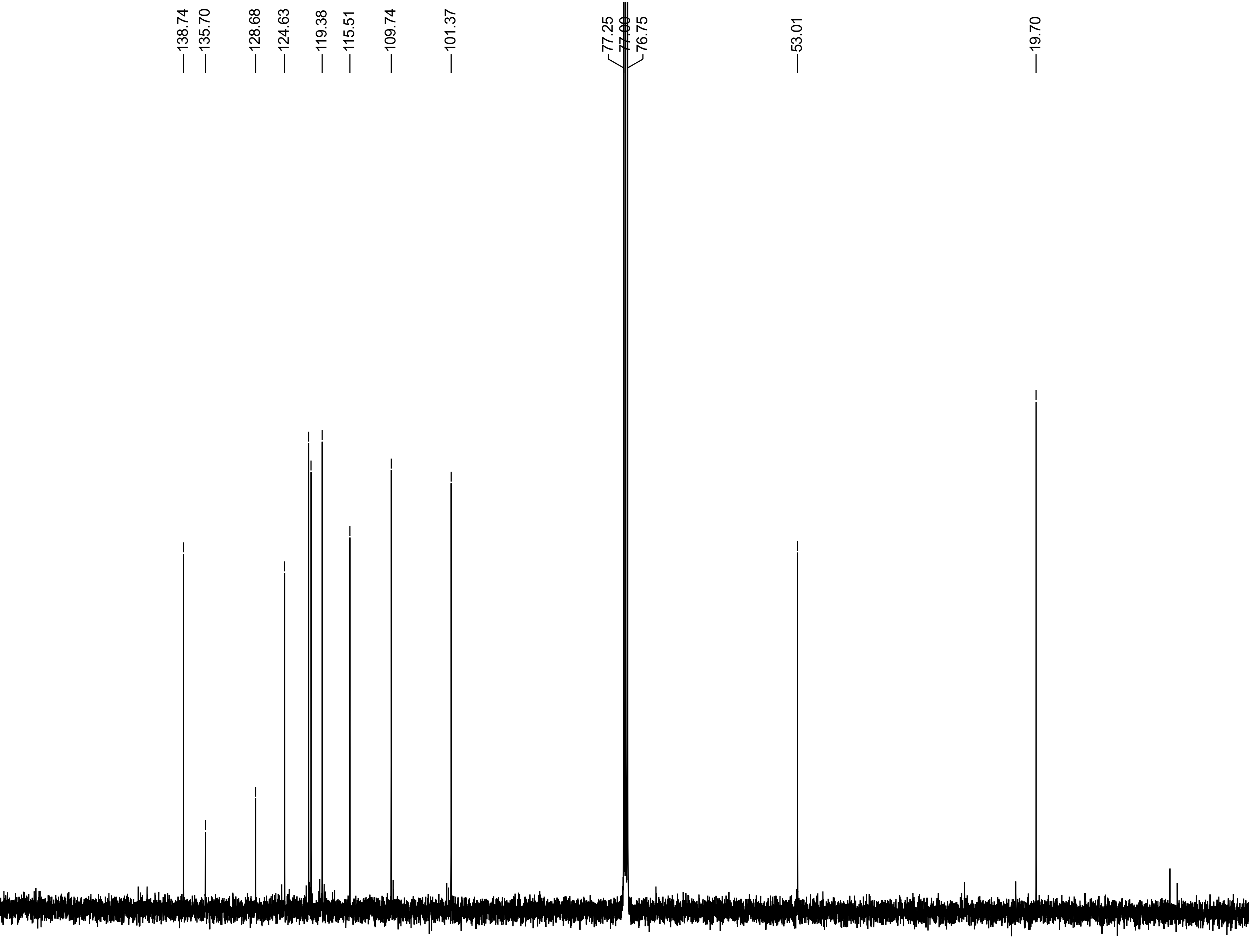

Supplementary Figure 183. ^{13}C NMR spectrum of compound **11**

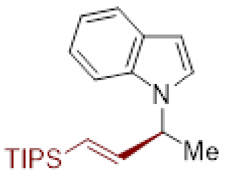

12

^{1}H NMR (400 MHz, $CDCl_3$)

Supplementary Figure 184.
^{1}H NMR spectrum of compound **12**

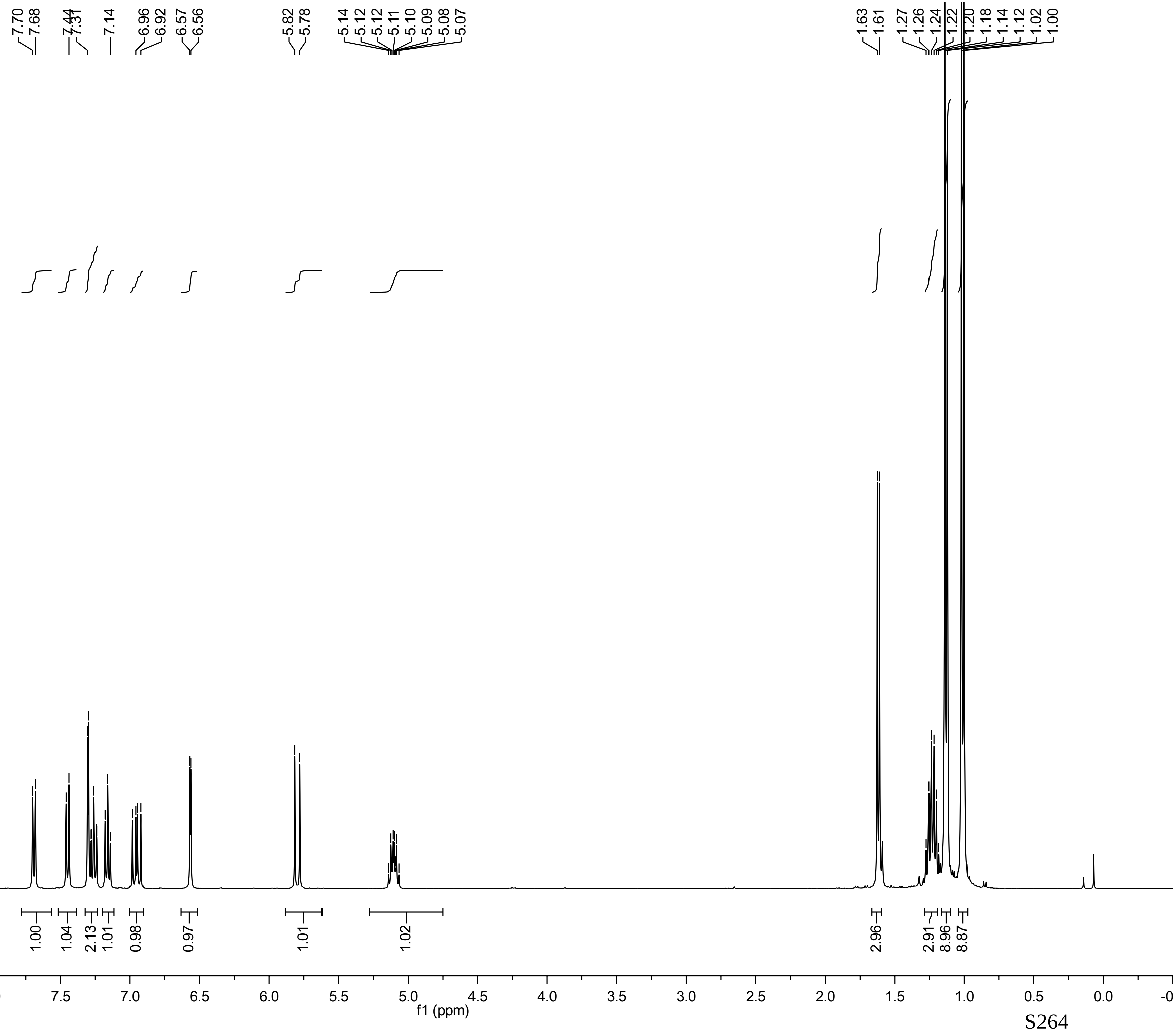

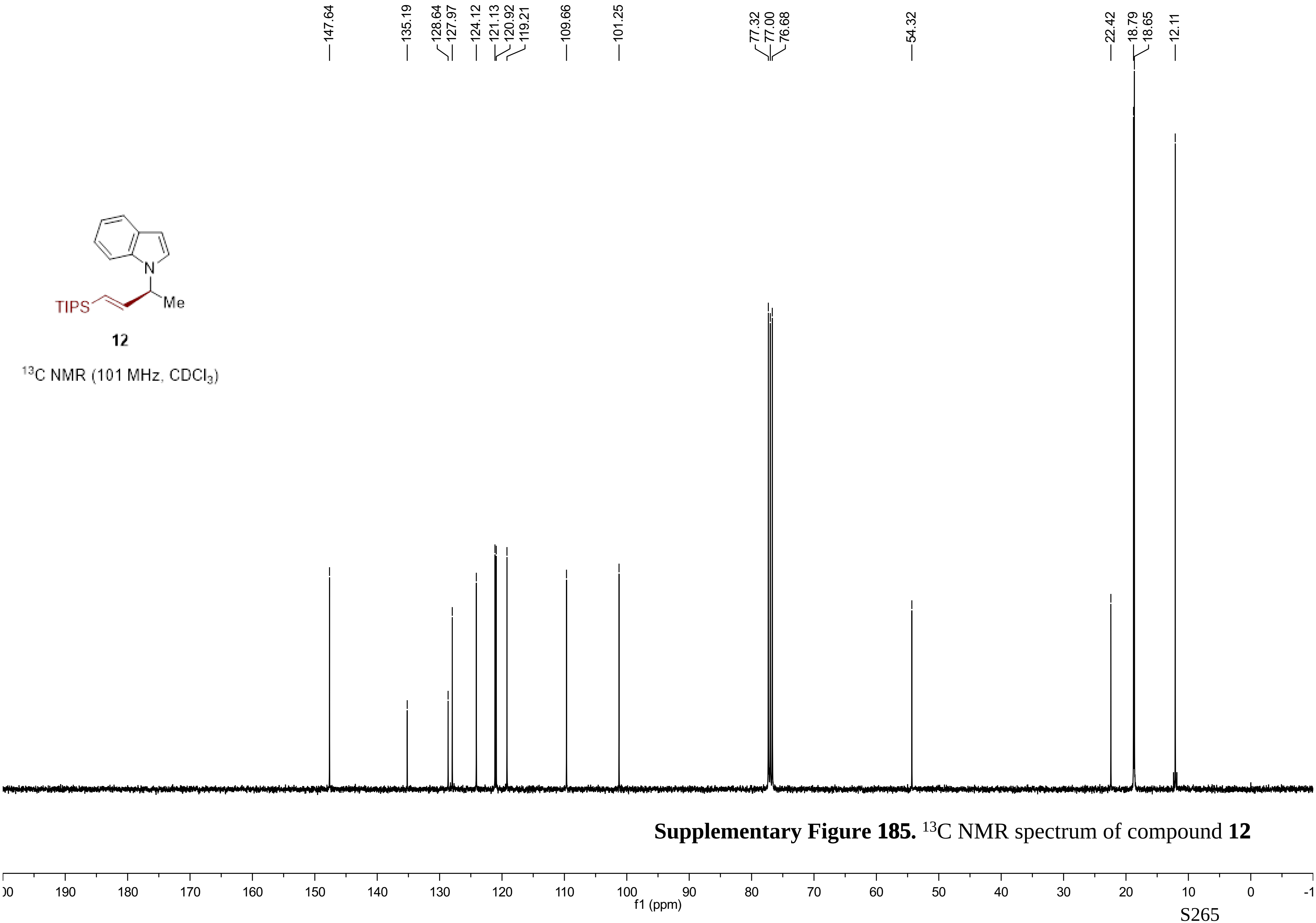

12

^{13}C NMR (101 MHz, $CDCl_3$)

Supplementary Figure 185. ^{13}C NMR spectrum of compound **12**

Data File E:\LCDATA\Data\lilun\2021-12-2111-53-57ll-1kiti-9-xiao.D
Sample Name: ll-1kiti-9-xiao

===

Acq. Operator : 系统
Sample Operator : 系统
Acq. Instrument : LC1260 Location : 21
Injection Date : 12/21/2021 11:54:59 AM
Inj Volume : 30.000 µl
Acq. Method : E:\LCDATA\Methods\LI-LUN.M
Last changed : 12/21/2021 11:53:24 AM by 系统
(modified after loading)
Analysis Method : E:\LCDATA\Methods\LI-LUN.M
Last changed : 3/14/2022 5:24:10 PM by 系统
(modified after loading)
Sample Info : Oj-H 80/1

Additional Info : Peak(s) manually integrated

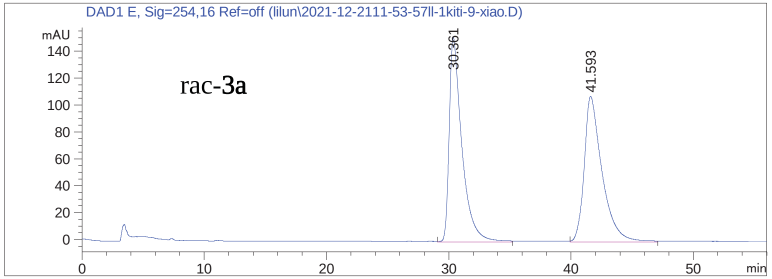

===

Area Percent Report

===

Sorted By : Signal
Multiplier : 1.0000
Dilution : 1.0000
Sample Amount: : 30.00000 [ng/ul] (not used in calc.)
Do not use Multiplier & Dilution Factor with ISTDs

Signal 1: DAD1 E, Sig=254,16 Ref=off

Peak #	RetTime [min]	Type	Width [min]	Area [mAU*s]	Height [mAU]	Area %
1	30.361	MM	1.2004	1.09468e4	151.99078	49.9140
2	41.593	MM	1.6906	1.09845e4	108.28822	50.0860

Totals : 2.19313e4 260.27900

===

*** End of Report ***

Data File E:\LCDATA\Data\lilun\2021-12-2110-31-51ll-1kiti-9-shou.D
Sample Name: ll-1kiti-9-shou

===

Acq. Operator : 系统
Sample Operator : 系统
Acq. Instrument : LC1260 Location : 21
Injection Date : 12/21/2021 10:32:44 AM
Inj Volume : 10.000 µl
Acq. Method : E:\LCDATA\Methods\LI-LUN.M
Last changed : 12/21/2021 9:29:33 AM by 系统
(modified after loading)
Analysis Method : E:\LCDATA\Methods\LI-LUN.M
Last changed : 3/14/2022 10:30:06 AM by 系统
(modified after loading)

d

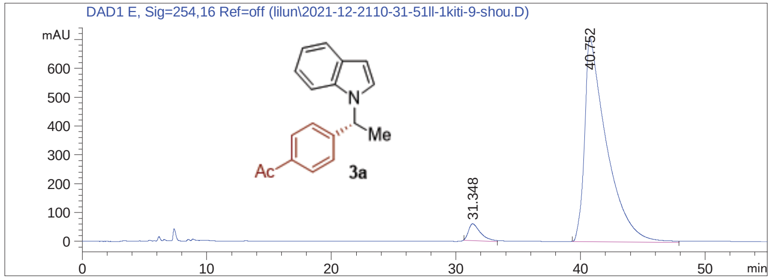

===

Area Percent Report

===

Sorted By : Signal
Multiplier : 1.0000
Dilution : 1.0000
Sample Amount: : 10.00000 [ng/ul] (not used in calc.)
Do not use Multiplier & Dilution Factor with ISTDs

Signal 1: DAD1 E, Sig=254,16 Ref=off

Peak #	RetTime [min]	Type	Width [min]	Area [mAU*s]	Height [mAU]	Area %
1	31.348	MM	1.1106	3899.10156	58.51097	4.2968
2	40.752	MM	2.0463	8.68446e4	707.34564	95.7032

Totals : 9.07437e4 765.85661

===

*** End of Report ***

Supplementary Figure 186. HPLC spectra of compound **3a**

Data File E:\LCDATA\Data\lilun\2021-11-0613-03-02ll-2-20-3L4.D
Sample Name: ll-2-20-3

===

Acq. Operator : 系统
Sample Operator : 系统
Acq. Instrument : LC1260 Location : 21
Injection Date : 11/6/2021 1:03:56 PM
Inj Volume : 10.000 µl
Acq. Method : E:\LCDATA\Methods\LI-LUN.M
Last changed : 11/6/2021 1:02:18 PM by 系统
(modified after loading)
Analysis Method : E:\LCDATA\Methods\LI-LUN.M
Last changed : 3/14/2022 2:00:15 PM by 系统
(modified after loading)
Sample Info : oj 80:10 1

Additional Info : Peak(s) manually integrated

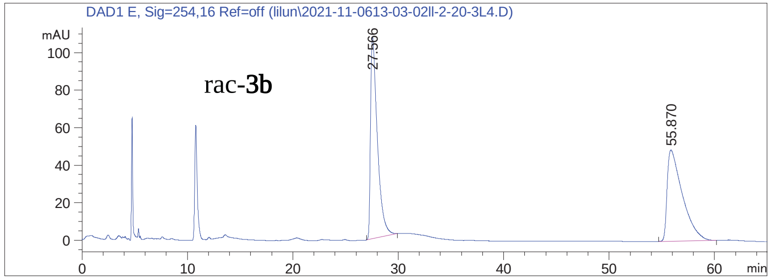

===

Area Percent Report

===

Sorted By : Signal
Multiplier : 1.0000
Dilution : 1.0000
Sample Amount: : 10.00000 [ng/ul] (not used in calc.)
Do not use Multiplier & Dilution Factor with ISTDs

Signal 1: DAD1 E, Sig=254,16 Ref=off

Peak #	RetTime [min]	Type	Width [min]	Area [mAU*s]	Height [mAU]	Area %
1	27.566	BB	0.6624	4839.65771	107.26121	49.8043
2	55.870	BB	1.3804	4877.69043	48.62081	50.1957

Totals : 9717.34814 155.88202

===

*** End of Report ***

Data File E:\LCDATA\Data\lilun\2022-09-1320-58-227.D
Sample Name: 7

===

Acq. Operator : 系统
Sample Operator : 系统
Acq. Instrument : LC1260 Location : 21
Injection Date : 9/13/2022 8:59:25 PM
Inj Volume : 30.000 µl
Acq. Method : E:\LCDATA\METHODS\LI-LUN.M
Last changed : 9/13/2022 8:53:52 PM by 系统
(modified after loading)
Analysis Method : E:\LCDATA\METHODS\LI-LUN.M
Last changed : 9/13/2022 10:09:16 PM by 系统
(modified after loading)
Sample Info : oj-H; n-hex:ipro=80:20; 1 ml/min,20 C

Additional Info : Peak(s) manually integrated

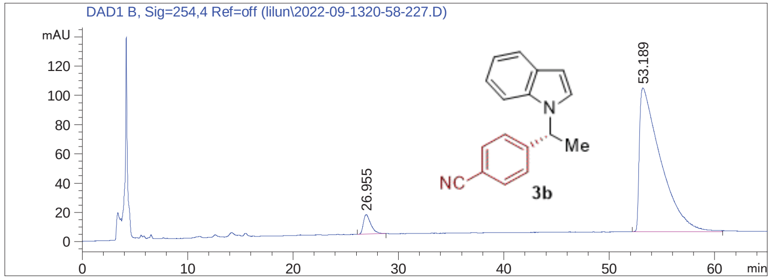

===

Area Percent Report

===

Sorted By : Signal
Multiplier : 1.0000
Dilution : 1.0000
Sample Amount: : 30.00000 [ng/ul] (not used in calc.)
Do not use Multiplier & Dilution Factor with ISTDs

Signal 1: DAD1 B, Sig=254,4 Ref=off

Peak #	RetTime [min]	Type	Width [min]	Area [mAU*s]	Height [mAU]	Area %
1	26.955	BB	0.6182	660.71234	13.28591	4.5466
2	53.189	MM	2.3521	1.38713e4	98.29184	95.4534

Totals : 1.45320e4 111.57775

===

*** End of Report ***

Supplementary Figure 187. HPLC spectra of compound **3b**

Data File E:\LCDATA\Data\lilun\2022-08-3010-48-10ll-1kiti-6-xiao.D
Sample Name: ll-1kiti-6-xiao

===

Acq. Operator : 系统
Sample Operator : 系统
Acq. Instrument : LC1260 Location : 21
Injection Date : 8/30/2022 10:49:10 AM
Inj Volume : 20.000 µl
Acq. Method : E:\LCDATA\METHODS\LI-LUN.M
Last changed : 8/30/2022 10:47:19 AM by 系统
(modified after loading)
Analysis Method : E:\LCDATA\METHODS\LI-LUN.M
Last changed : 8/30/2022 11:29:21 AM by 系统
(modified after loading)
Sample Info : oj-H; n-hex:ipro=90:10; 1.0ml/min,20 C

Additional Info : Peak(s) manually integrated

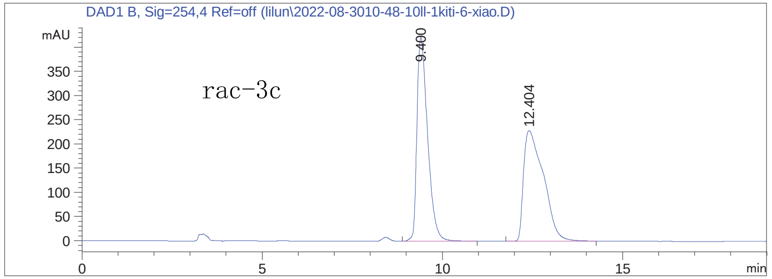

===

Area Percent Report

===

Sorted By : Signal
Multiplier : 1.0000
Dilution : 1.0000
Sample Amount: : 20.00000 [ng/ul] (not used in calc.)
Do not use Multiplier & Dilution Factor with ISTDs

Signal 1: DAD1 B, Sig=254,4 Ref=off

Peak #	RetTime [min]	Type	Width [min]	Area [mAU*s]	Height [mAU]	Area %
1	9.400	BB	0.3188	8692.75195	421.10175	49.8110
2	12.404	BBA	0.5466	8758.73047	228.81630	50.1890

Totals : 1.74515e4 649.91805

===

*** End of Report ***

Data File E:\LCDATA\Data\lilun\2022-08-3015-28-15ll-1kiti-6-shou.D
Sample Name: ll-1kiti-6-shou

===

Acq. Operator : 系统
Sample Operator : 系统
Acq. Instrument : LC1260 Location : 21
Injection Date : 8/30/2022 3:29:15 PM
Inj Volume : 20.000 µl
Acq. Method : E:\LCDATA\METHODS\LI-LUN.M
Last changed : 8/30/2022 3:27:21 PM by 系统
(modified after loading)
Analysis Method : E:\LCDATA\METHODS\LI-LUN.M
Last changed : 8/30/2022 2:25:19 PM by 系统
(modified after loading)
Sample Info : oj-H; n-hex:ipro=95;5; 1.0ml/min,20 C

Additional Info : Peak(s) manually integrated

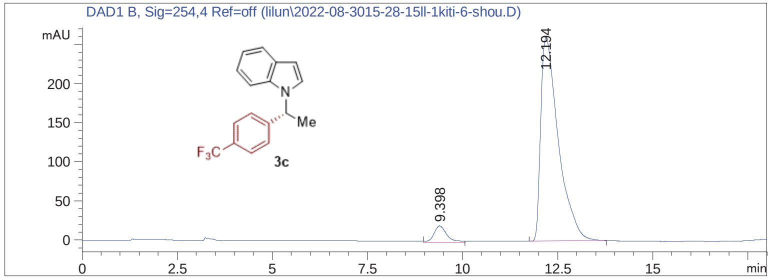

===

Area Percent Report

===

Sorted By : Signal
Multiplier : 1.0000
Dilution : 1.0000
Sample Amount: : 20.00000 [ng/ul] (not used in calc.)
Do not use Multiplier & Dilution Factor with ISTDs

Signal 1: DAD1 B, Sig=254,4 Ref=off

Peak #	RetTime [min]	Type	Width [min]	Area [mAU*s]	Height [mAU]	Area %
1	9.398	MM	0.3948	495.25275	20.90561	5.5957
2	12.194	MM	0.5354	8355.35742	260.11557	94.4043

Totals : 8850.61017 281.02118

===

*** End of Report ***

Supplementary Figure 188. HPLC spectra of compound **3c**

Data File E:\LCDATA\Data\lilun\2022-01-1311-30-01ll-1kiti-10-xiao.D
Sample Name: ll-1kiti-10-xiao

===

Acq. Operator : 系统
Sample Operator : 系统
Acq. Instrument : LC1260 Location : 21
Injection Date : 1/13/2022 11:30:57 AM
Inj Volume : 10.000 µl
Acq. Method : E:\LCDATA\Methods\LI-LUN.M
Last changed : 1/12/2022 11:23:46 AM by 系统
Analysis Method : E:\LCDATA\Methods\LI-LUN.M
Last changed : 3/18/2022 9:19:45 PM by 系统
(modified after loading)
Sample Info : oj 80 1 20

Additional Info : Peak(s) manually integrated

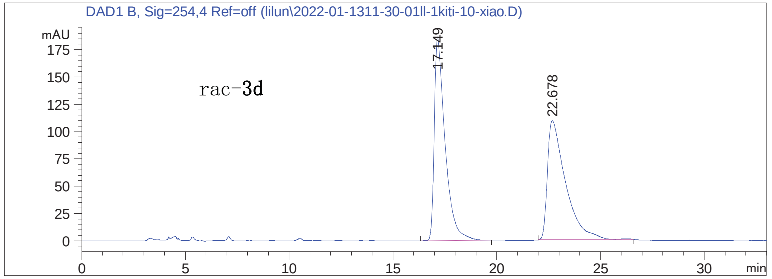

===
Area Percent Report
===

Sorted By : Signal
Multiplier : 1.0000
Dilution : 1.0000
Sample Amount : 10.00000 [ng/ul] (not used in calc.)
Do not use Multiplier & Dilution Factor with ISTDs

Signal 1: DAD1 B, Sig=254,4 Ref=off

Peak #	RetTime [min]	Type	Width [min]	Area [mAU*s]	Height [mAU]	Area %
1	17.149	BB	0.5218	6734.26563	186.26056	50.1865
2	22.678	MM	1.0201	6684.20264	109.20898	49.8135

Totals : 1.34185e4 295.46954

===
*** End of Report ***

Data File E:\LCDATA\Data\lilun\2022-01-1312-17-55ll-1kiti-10-shou.D
Sample Name: ll-1kiti-10-shou

===

Acq. Operator : 系统
Sample Operator : 系统
Acq. Instrument : LC1260 Location : 21
Injection Date : 1/13/2022 12:18:50 PM
Inj Volume : 10.000 µl
Acq. Method : E:\LCDATA\Methods\LI-LUN.M
Last changed : 1/12/2022 11:23:46 AM by 系统
Analysis Method : E:\LCDATA\Methods\LI-LUN.M
Last changed : 3/18/2022 9:19:45 PM by 系统
(modified after loading)
Sample Info : oj 80 1 20

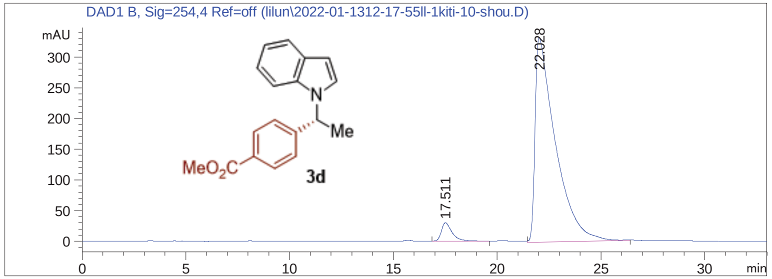

===
Area Percent Report
===

Sorted By : Signal
Multiplier : 1.0000
Dilution : 1.0000
Sample Amount : 10.00000 [ng/ul] (not used in calc.)
Do not use Multiplier & Dilution Factor with ISTDs

Signal 1: DAD1 B, Sig=254,4 Ref=off

Peak #	RetTime [min]	Type	Width [min]	Area [mAU*s]	Height [mAU]	Area %
1	17.511	BB	0.5243	1057.41858	29.77539	4.4637
2	22.028	MM	1.1329	2.26317e4	332.94354	95.5363

Totals : 2.36892e4 362.71893

===
*** End of Report ***

Supplementary Figure 189. HPLC spectra of compound **3d**

Data File E:\LCDATA\Data\lilun\2021-11-0512-03-09ll-2-19-1L7.D
Sample Name: ll-2-19-1

===
Acq. Operator   : 系统
Sample Operator : 系统
Acq. Instrument : LC1260   Location :   21
Injection Date : 11/5/2021 12:04:05 PM
                                                Inj Volume : 10.000 µl
Acq. Method   : E:\LCDATA\Methods\LI-LUN.M
Last changed   : 11/5/2021 9:05:24 AM by 系统
                  (modified after loading)
Analysis Method : E:\LCDATA\Methods\LI-LUN.M
Last changed   : 3/14/2022 2:15:15 PM by 系统
                  (modified after loading)
Sample Info   : od3 90:10 1

Additional Info : Peak(s) manually integrated

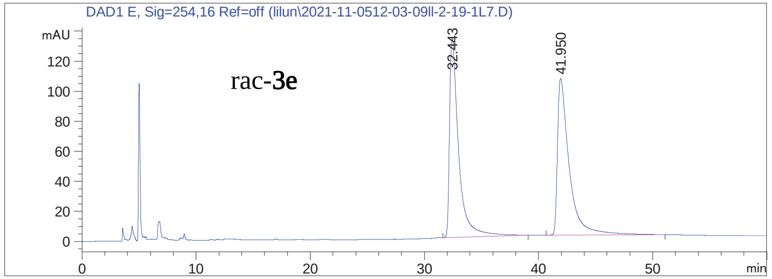

===
Area Percent Report
===

Sorted By   :   Signal
Multiplier   :   1.0000
Dilution   :   1.0000
Sample Amount: :   10.00000 [ng/ul]   (not used in calc.)
Do not use Multiplier & Dilution Factor with ISTDs

Signal 1: DAD1 E, Sig=254,16 Ref=off

Peak #	RetTime [min]	Type	Width [min]	Area [mAU*s]	Height [mAU]	Area %
1	32.443	BB	0.8061	7366.41943	133.02501	49.5798
2	41.950	BB	1.0314	7491.29541	104.37438	50.4202

Totals :   1.48577e4   237.39939

===
*** End of Report ***

Data File E:\LCDATA\Data\lilun\2021-11-0513-09-50ll-2-19-2L7.D
Sample Name: ll-2-19-2

===
Acq. Operator   : 系统
Sample Operator : 系统
Acq. Instrument : LC1260   Location :   21
Injection Date : 11/5/2021 1:10:46 PM
                                                Inj Volume : 10.000 µl
Acq. Method   : E:\LCDATA\Methods\LI-LUN.M
Last changed   : 11/5/2021 9:05:24 AM by 系统
                  (modified after loading)
Analysis Method : E:\LCDATA\Methods\LI-LUN.M
Last changed   : 3/14/2022 2:15:15 PM by 系统
                  (modified after loading)
Sample Info   : od3 90:10 1

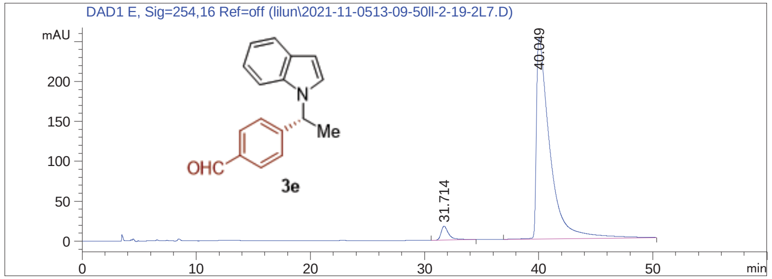

===
Area Percent Report
===

Sorted By   :   Signal
Multiplier   :   1.0000
Dilution   :   1.0000
Sample Amount: :   10.00000 [ng/ul]   (not used in calc.)
Do not use Multiplier & Dilution Factor with ISTDs

Signal 1: DAD1 E, Sig=254,16 Ref=off

Peak #	RetTime [min]	Type	Width [min]	Area [mAU*s]	Height [mAU]	Area %
1	31.714	BB	0.7088	838.62433	17.50891	3.8943
2	40.049	BBA	1.1515	2.06961e4	252.17807	96.1057

Totals :   2.15347e4   269.68698

===
*** End of Report ***

Supplementary Figure 190. HPLC spectra of compound **3e**

Data File E:\LCDATA\Data\lilun\2021-12-1111-16-36ll-2-58-3.D
Sample Name: ll-2-58-3

===

Acq. Operator : 系统
Sample Operator : 系统
Acq. Instrument : LC1260 Location : 21
Injection Date : 12/11/2021 11:17:42 AM
Inj Volume : 40.000 µl
Acq. Method : E:\LCDATA\Methods\ysl.M
Last changed : 12/11/2021 11:16:29 AM by 系统
(modified after loading)
Analysis Method : E:\LCDATA\Methods\LI-LUN.M
Last changed : 3/14/2022 2:25:05 PM by 系统
(modified after loading)
Sample Info : od 90;10 1

Additional Info : Peak(s) manually integrated

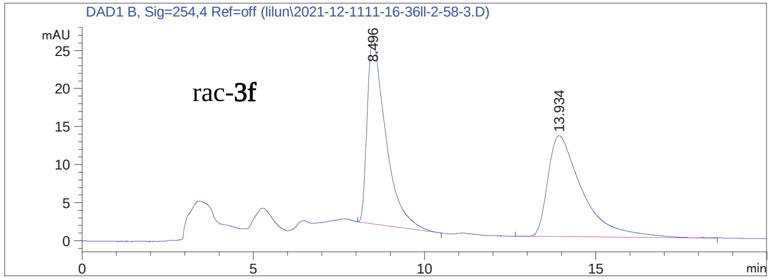

===

Area Percent Report

===

Sorted By : Signal
Multiplier : 1.0000
Dilution : 1.0000
Sample Amount: : 40.00000 [ng/ul] (not used in calc.)
Do not use Multiplier & Dilution Factor with ISTDs

Signal 1: DAD1 B, Sig=254,4 Ref=off

Peak #	RetTime [min]	Type	Width [min]	Area [mAU*s]	Height [mAU]	Area %
1	8.496	BB	0.5356	919.32318	24.50812	49.7732
2	13.934	MM	1.1659	927.70270	13.26208	50.2268

Totals : 1847.02588 37.77021

===

*** End of Report ***

Data File E:\LCDATA\Data\lilun\2021-12-1110-50-18ll-2-58-4.D
Sample Name: ll-2-58-4

===

Acq. Operator : 系统
Sample Operator : 系统
Acq. Instrument : LC1260 Location : 21
Injection Date : 12/11/2021 10:51:15 AM
Inj Volume : 20.000 µl
Acq. Method : E:\LCDATA\Methods\ysl.M
Last changed : 12/11/2021 10:49:52 AM by 系统
(modified after loading)
Analysis Method : E:\LCDATA\Methods\LI-LUN.M
Last changed : 3/14/2022 2:25:05 PM by 系统
(modified after loading)
Sample Info : od 90;10 1

Additional Info : Peak(s) manually integrated

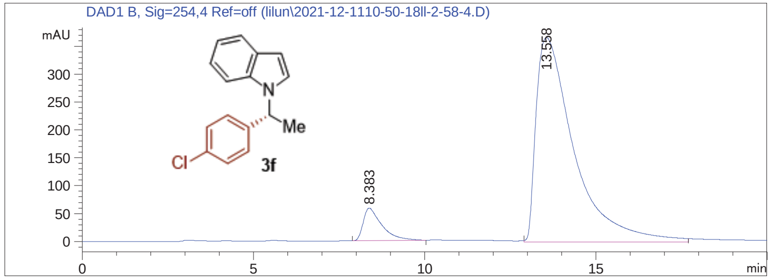

===

Area Percent Report

===

Sorted By : Signal
Multiplier : 1.0000
Dilution : 1.0000
Sample Amount: : 20.00000 [ng/ul] (not used in calc.)
Do not use Multiplier & Dilution Factor with ISTDs

Signal 1: DAD1 B, Sig=254,4 Ref=off

Peak #	RetTime [min]	Type	Width [min]	Area [mAU*s]	Height [mAU]	Area %
1	8.383	BB	0.5316	2195.26978	58.53066	7.1911
2	13.558	MM	1.2881	2.83322e4	366.58752	92.8089

Totals : 3.05275e4 425.11818

===

*** End of Report ***

Supplementary Figure 191. HPLC spectra of compound **3f**

Data File E:\LCDATA\Data\lilun\2022-01-0719-20-24ll-1kiti-39.D
Sample Name: ll-1kiti-39

===

Acq. Operator : 系统
Sample Operator : 系统
Acq. Instrument : LC1260 Location : 21
Injection Date : 1/7/2022 7:21:18 PM
Inj Volume : 10.000 µl
Acq. Method : E:\LCDATA\Methods\LI-LUN.M
Last changed : 1/7/2022 7:11:29 PM by 系统
(modified after loading)
Analysis Method : E:\LCDATA\Methods\LI-LUN.M
Last changed : 3/14/2022 2:25:05 PM by 系统
(modified after loading)
Sample Info : n-hex/i-proh=90:10 1ml/min,od

Additional Info : Peak(s) manually integrated

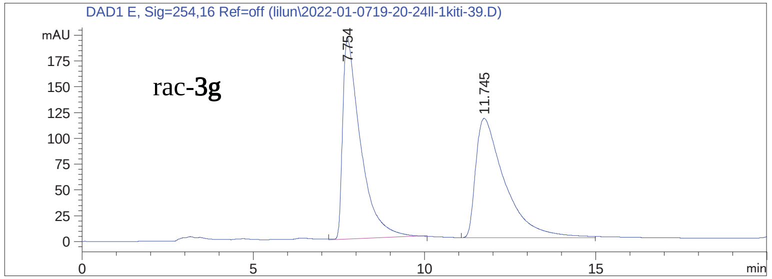

===
Area Percent Report
===

Sorted By : Signal
Multiplier : 1.0000
Dilution : 1.0000
Sample Amount: : 10.00000 [ng/ul] (not used in calc.)
Do not use Multiplier & Dilution Factor with ISTDs

Signal 1: DAD1 E, Sig=254,16 Ref=off

Peak #	RetTime [min]	Type	Width [min]	Area [mAU*s]	Height [mAU]	Area %
1	7.754	MM	0.6026	7066.51953	195.45941	50.6522
2	11.745	MM	0.9917	6884.54492	115.70226	49.3478

Totals : 1.39511e4 311.16167

===
*** End of Report ***

Data File E:\LCDATA\Data\lilun\2022-01-0719-51-45ll-1kiti-39-shou.D
Sample Name: ll-1kiti-39-shou

===

Acq. Operator : 系统
Sample Operator : 系统
Acq. Instrument : LC1260 Location : 21
Injection Date : 1/7/2022 7:52:38 PM
Inj Volume : 10.000 µl
Acq. Method : E:\LCDATA\Methods\LI-LUN.M
Last changed : 1/7/2022 7:11:29 PM by 系统
(modified after loading)
Analysis Method : E:\LCDATA\Methods\LI-LUN.M
Last changed : 3/14/2022 2:25:05 PM by 系统
(modified after loading)
Sample Info : n-hex/i-proh=90:10 1ml/min,od

Additional Info : Peak(s) manually integrated

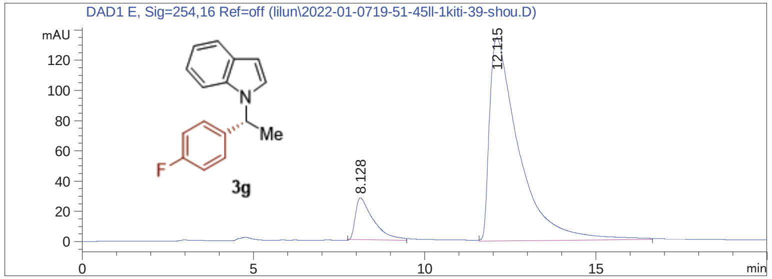

===
Area Percent Report
===

Sorted By : Signal
Multiplier : 1.0000
Dilution : 1.0000
Sample Amount: : 10.00000 [ng/ul] (not used in calc.)
Do not use Multiplier & Dilution Factor with ISTDs

Signal 1: DAD1 E, Sig=254,16 Ref=off

Peak #	RetTime [min]	Type	Width [min]	Area [mAU*s]	Height [mAU]	Area %
1	8.128	MM	0.6047	999.68433	27.55154	11.0942
2	12.115	MM	0.9910	8011.20313	134.73898	88.9058

Totals : 9010.88745 162.29052

===
*** End of Report ***

Supplementary Figure 192. HPLC spectra of compound **3g**

Data File E:\LCDATA\Data\lilun\2022-08-2914-49-52ll-1kiti-56-xiao.D
Sample Name: ll-1kiti-56-xiao

===
Acq. Operator   : 系统
Sample Operator : 系统
Acq. Instrument : LC1260   Location :   21
Injection Date : 8/29/2022 2:50:48 PM
                                                Inj Volume : 20.000 µl
Acq. Method   : E:\LCDATA\METHODS\LI-LUN.M
Last changed   : 8/29/2022 2:47:57 PM by 系统
                  (modified after loading)
Analysis Method : E:\LCDATA\METHODS\LI-LUN.M
Last changed   : 8/29/2022 3:20:52 PM by 系统
                  (modified after loading)
Sample Info   : od3-H; n-hex:ipro=98:2; 1.0ml/min,20 C

Additional Info : Peak(s) manually integrated

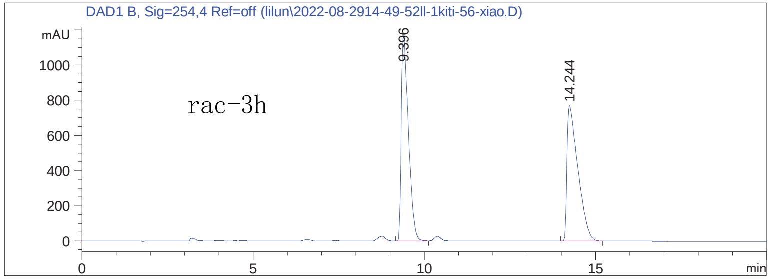

===
Area Percent Report
===

Sorted By   :   Signal
Multiplier   :   1.0000
Dilution   :   1.0000
Sample Amount: :   20.00000 [ng/ul]   (not used in calc.)
Do not use Multiplier & Dilution Factor with ISTDs

Signal 1: DAD1 B, Sig=254,4 Ref=off

Peak #	RetTime [min]	Type	Width [min]	Area [mAU*s]	Height [mAU]	Area %
1	9.396	BV	0.2252	1.70173e4	1159.54126	49.8980
2	14.244	MM	0.3698	1.70869e4	770.16302	50.1020

Totals :   3.41042e4 1929.70428

===
*** End of Report ***

Data File E:\LCDATA\Data\lilun\2022-08-2915-24-10ll-1kiti-56-shou.D
Sample Name: ll-1kiti-56-shou

===
Acq. Operator   : 系统
Sample Operator : 系统
Acq. Instrument : LC1260   Location :   21
Injection Date : 8/29/2022 3:25:11 PM
                                                Inj Volume : 20.000 µl
Acq. Method   : E:\LCDATA\METHODS\LI-LUN.M
Last changed   : 8/29/2022 2:47:57 PM by 系统
                  (modified after loading)
Analysis Method : E:\LCDATA\METHODS\LI-LUN.M
Last changed   : 8/29/2022 3:20:52 PM by 系统
                  (modified after loading)
Sample Info   : od3-H; n-hex:ipro=98:2; 1.0ml/min,20 C

Additional Info : Peak(s) manually integrated

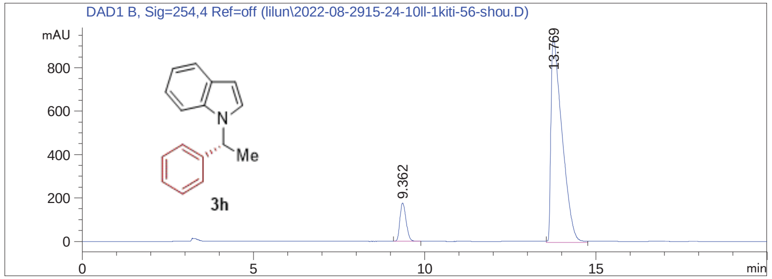

===
Area Percent Report
===

Sorted By   :   Signal
Multiplier   :   1.0000
Dilution   :   1.0000
Sample Amount: :   20.00000 [ng/ul]   (not used in calc.)
Do not use Multiplier & Dilution Factor with ISTDs

Signal 1: DAD1 B, Sig=254,4 Ref=off

Peak #	RetTime [min]	Type	Width [min]	Area [mAU*s]	Height [mAU]	Area %
1	9.362	BB	0.1932	2207.76099	177.34966	9.4177
2	13.769	MM	0.3750	2.12349e4	943.88458	90.5823

Totals :   2.34427e4 1121.23424

===
*** End of Report ***

Supplementary Figure 193. HPLC spectra of compound **3h**

Data File E:\LCDATA\Data\lilun\2022-08-3019-54-11ll-1kiti-27-xiao.D
Sample Name: ll-1kiti-27-xiao

===
Acq. Operator   : 系统
Sample Operator : 系统
Acq. Instrument : LC1260   Location :   21
Injection Date : 8/30/2022 7:55:14 PM
                                                Inj Volume : 20.000 µl
Acq. Method   : E:\LCDATA\METHODS\LI-LUN.M
Last changed   : 8/30/2022 7:18:30 PM by 系统
                  (modified after loading)
Analysis Method : E:\LCDATA\METHODS\LI-LUN.M
Last changed   : 8/30/2022 7:57:08 PM by 系统
                  (modified after loading)
Sample Info   : od-H; n-hex:ipro=90;10; 1.0ml/min,20 C

Additional Info : Peak(s) manually integrated

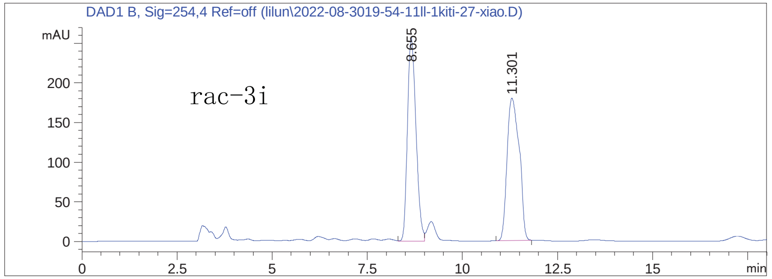

===
Area Percent Report
===

Sorted By   :   Signal
Multiplier   :   1.0000
Dilution   :   1.0000
Sample Amount: :   20.00000 [ng/ul]   (not used in calc.)
Do not use Multiplier & Dilution Factor with ISTDs

Signal 1: DAD1 B, Sig=254,4 Ref=off

Peak #	RetTime [min]	Type	Width [min]	Area [mAU*s]	Height [mAU]	Area %
1	8.655	MM	0.2617	4035.72852	257.00378	50.2597
2	11.301	BBA	0.3352	3994.01685	179.74971	49.7403

Totals :   8029.74536 436.75349

===
*** End of Report ***

Data File E:\LCDATA\Data\lilun\2022-08-3019-19-30ll-1kiti-27-shou.D
Sample Name: ll-1kiti-27-shou

===
Acq. Operator   : 系统
Sample Operator : 系统
Acq. Instrument : LC1260   Location :   21
Injection Date : 8/30/2022 7:20:30 PM
                                                Inj Volume : 20.000 µl
Acq. Method   : E:\LCDATA\METHODS\LI-LUN.M
Last changed   : 8/30/2022 7:18:30 PM by 系统
                  (modified after loading)
Analysis Method : E:\LCDATA\METHODS\LI-LUN.M
Last changed   : 8/30/2022 7:57:08 PM by 系统
                  (modified after loading)
Sample Info   : od-H; n-hex:ipro=90;10; 1.0ml/min,20 C

Additional Info : Peak(s) manually integrated

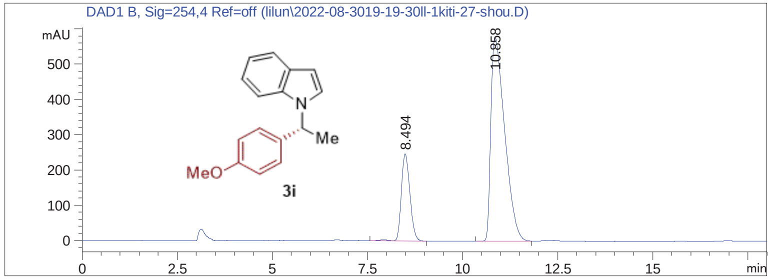

===
Area Percent Report
===

Sorted By   :   Signal
Multiplier   :   1.0000
Dilution   :   1.0000
Sample Amount: :   20.00000 [ng/ul]   (not used in calc.)
Do not use Multiplier & Dilution Factor with ISTDs

Signal 1: DAD1 B, Sig=254,4 Ref=off

Peak #	RetTime [min]	Type	Width [min]	Area [mAU*s]	Height [mAU]	Area %
1	8.494	VB R	0.2446	3909.16528	246.71608	21.1787
2	10.858	BBA	0.3898	1.45489e4	577.68311	78.8213

Totals :   1.84580e4   824.39919

===
*** End of Report ***

Supplementary Figure 194. HPLC spectra of compound **3i**

Data File E:\LCDATA\Data\lilun\2021-11-2912-54-37ll-2-46-3.D
Sample Name: ll-2-46-3

===
Acq. Operator   : 系统
Sample Operator : 系统
Acq. Instrument : LC1260   Location :   21
Injection Date : 11/29/2021 12:55:29 PM
                                                Inj Volume : 10.000 µl
Acq. Method   : E:\LCDATA\Methods\LI-LUN.M
Last changed   : 11/29/2021 12:52:20 PM by 系统
                  (modified after loading)
Analysis Method : E:\LCDATA\Methods\LI-LUN.M
Last changed   : 3/14/2022 2:54:35 PM by 系统
                  (modified after loading)
Sample Info   : oD 90:10 1

Additional Info : Peak(s) manually integrated

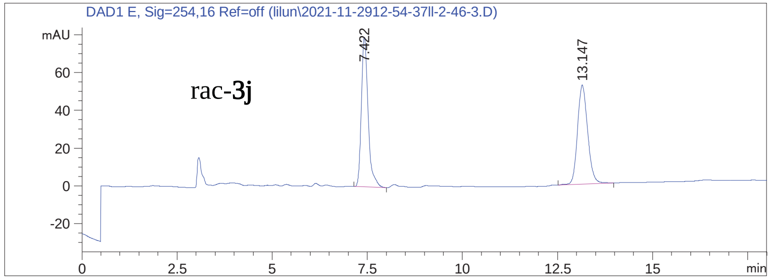

===
Area Percent Report
===

Sorted By   :   Signal
Multiplier   :   1.0000
Dilution   :   1.0000
Sample Amount: :   10.00000 [ng/ul]   (not used in calc.)
Do not use Multiplier & Dilution Factor with ISTDs

Signal 1: DAD1 E, Sig=254,16 Ref=off

Peak #	RetTime [min]	Type	Width [min]	Area [mAU*s]	Height [mAU]	Area %
1	7.422	BB	0.1881	976.84851	79.09657	50.0592
2	13.147	BB	0.2868	974.53625	52.45243	49.9408

Totals :   1951.38477 131.54900

===
*** End of Report ***

Data File E:\LCDATA\Data\lilun\2021-11-2913-18-09ll-ocf3-shou.D
Sample Name: ll-ocf3-shou

===
Acq. Operator   : 系统
Sample Operator : 系统
Acq. Instrument : LC1260   Location :   21
Injection Date : 11/29/2021 1:19:02 PM
                                                Inj Volume : 10.000 µl
Acq. Method   : E:\LCDATA\Methods\LI-LUN.M
Last changed   : 11/29/2021 12:52:20 PM by 系统
                  (modified after loading)
Analysis Method : E:\LCDATA\Methods\LI-LUN.M
Last changed   : 3/14/2022 2:54:35 PM by 系统
                  (modified after loading)
Sample Info   : oD 90:10 1

Additional Info : Peak(s) manually integrated

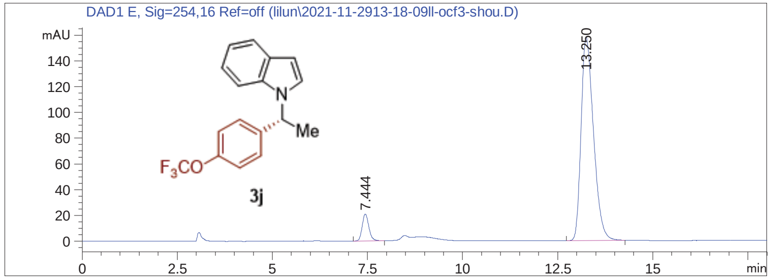

===
Area Percent Report
===

Sorted By   :   Signal
Multiplier   :   1.0000
Dilution   :   1.0000
Sample Amount: :   10.00000 [ng/ul]   (not used in calc.)
Do not use Multiplier & Dilution Factor with ISTDs

Signal 1: DAD1 E, Sig=254,16 Ref=off

Peak #	RetTime [min]	Type	Width [min]	Area [mAU*s]	Height [mAU]	Area %
1	7.444	BB	0.1873	252.55670	20.85031	6.5990
2	13.250	BBA	0.3486	3574.63745	157.63173	93.4010

Totals :   3827.19415 178.48204

===
*** End of Report ***

Supplementary Figure 195. HPLC spectra of compound **3j**

```
Data File E:\LCDATA\Data\lilun\2021-11-2119-49-31ll-2-40-3.D
Sample Name: ll-2-40-3

=====================================================================
Acq. Operator   : 系统
Sample Operator : 系统
Acq. Instrument : LC1260                          Location :   21
Injection Date  : 11/21/2021 7:50:25 PM
                                                Inj Volume : 10.000 µl
Acq. Method     : E:\LCDATA\Methods\LI-LUN.M
Last changed    : 11/21/2021 7:48:31 PM by 系统
                  (modified after loading)
Analysis Method : E:\LCDATA\Methods\LI-LUN.M
Last changed    : 3/14/2022 2:57:42 PM by 系统
                  (modified after loading)
Sample Info     : oj  90:10  1

Additional Info : Peak(s) manually integrated
```

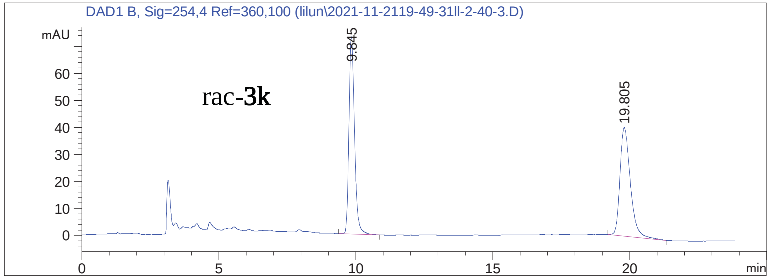


```
=====================================================================
                         Area Percent Report
=====================================================================

Sorted By             :      Signal
Multiplier            :      1.0000
Dilution              :      1.0000
Sample Amount:              :      10.00000  [ng/ul]   (not used in calc.)
Do not use Multiplier & Dilution Factor with ISTDs


Signal 1: DAD1 B, Sig=254,4 Ref=360,100
```

Peak #	RetTime [min]	Type	Width [min]	Area [mAU*s]	Height [mAU]	Area %
1	9.845	BB	0.2174	1033.89563	72.91469	49.5305
2	19.805	BB	0.3995	1053.49756	40.23045	50.4695

Totals : 2087.39319   113.14514

```
=====================================================================
                       *** End of Report ***
```

```
Data File E:\LCDATA\Data\lilun\2021-11-2120-21-20ll-2-39-4.D
Sample Name: ll-2-39-4

=====================================================================
Acq. Operator   : 系统
Sample Operator : 系统
Acq. Instrument : LC1260                          Location :   21
Injection Date  : 11/21/2021 8:22:17 PM
                                                Inj Volume : 10.000 µl
Acq. Method     : E:\LCDATA\Methods\LI-LUN.M
Last changed    : 11/21/2021 7:48:31 PM by 系统
                  (modified after loading)
Analysis Method : E:\LCDATA\Methods\LI-LUN.M
Last changed    : 3/14/2022 3:00:00 PM by 系统
                  (modified after loading)
Sample Info     : OD-3  90:10  1
```

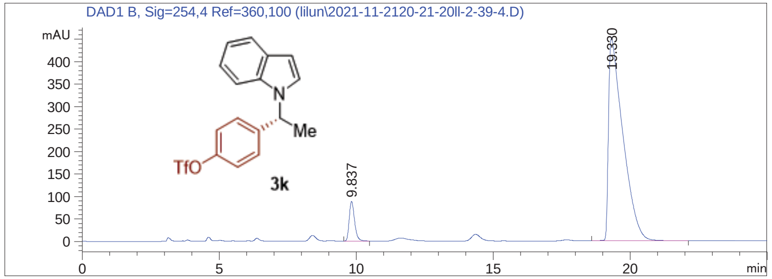


```
=====================================================================
                         Area Percent Report
=====================================================================

Sorted By             :      Signal
Multiplier            :      1.0000
Dilution              :      1.0000
Sample Amount:              :      10.00000  [ng/ul]   (not used in calc.)
Do not use Multiplier & Dilution Factor with ISTDs


Signal 1: DAD1 B, Sig=254,4 Ref=360,100
```

Peak #	RetTime [min]	Type	Width [min]	Area [mAU*s]	Height [mAU]	Area %
1	9.837	BB	0.2101	1197.47485	88.36588	6.7609
2	19.330	BB	0.5180	1.65144e4	452.30508	93.2391

Totals : 1.77118e4   540.67097

```
=====================================================================
                       *** End of Report ***
```

Supplementary Figure 196. HPLC spectra of compound **3k**

Data File E:\LCDATA\Data\lilun\2021-11-1818-40-22ll-2-36-311-17Z.D
Sample Name: ll-2-36-3

===

Acq. Operator : 系统
Sample Operator : 系统
Acq. Instrument : LC1260 Location : 21
Injection Date : 11/18/2021 6:41:15 PM
Inj Volume : 10.000 µl
Acq. Method : E:\LCDATA\Methods\LI-LUN.M
Last changed : 11/18/2021 6:39:10 PM by 系统
(modified after loading)
Analysis Method : E:\LCDATA\Methods\LI-LUN.M
Last changed : 3/14/2022 3:03:01 PM by 系统
(modified after loading)
Sample Info : oD 3 95:5 0.6

Additional Info : Peak(s) manually integrated

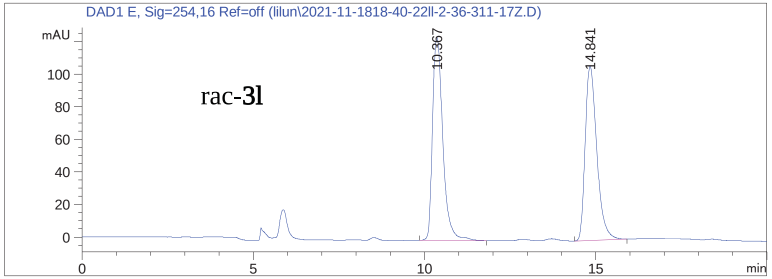

===

Area Percent Report

===

Sorted By : Signal
Multiplier : 1.0000
Dilution : 1.0000
Sample Amount: : 10.00000 [ng/ul] (not used in calc.)
Do not use Multiplier & Dilution Factor with ISTDs

Signal 1: DAD1 E, Sig=254,16 Ref=off

Peak #	RetTime [min]	Type	Width [min]	Area [mAU*s]	Height [mAU]	Area %
1	10.367	BBA	0.3273	2621.84619	124.67635	50.9564
2	14.841	BB	0.3629	2523.42871	107.10075	49.0436

Totals : 5145.27490 231.77709

===

*** End of Report ***

Data File E:\LCDATA\Data\lilun\2022-08-2913-42-42ll-1kiti-19-shou.D
Sample Name: ll-1kiti-19-shou

===

Acq. Operator : 系统
Sample Operator : 系统
Acq. Instrument : LC1260 Location : 21
Injection Date : 8/29/2022 1:43:42 PM
Inj Volume : 20.000 µl
Acq. Method : E:\LCDATA\METHODS\LI-LUN.M
Last changed : 8/29/2022 1:13:52 PM by 系统
(modified after loading)
Analysis Method : E:\LCDATA\METHODS\LI-LUN.M
Last changed : 8/29/2022 1:43:28 PM by 系统
(modified after loading)
Sample Info : od3-H; n-hex:ipro=90:10; 1.0ml/min,20 C

Additional Info : Peak(s) manually integrated

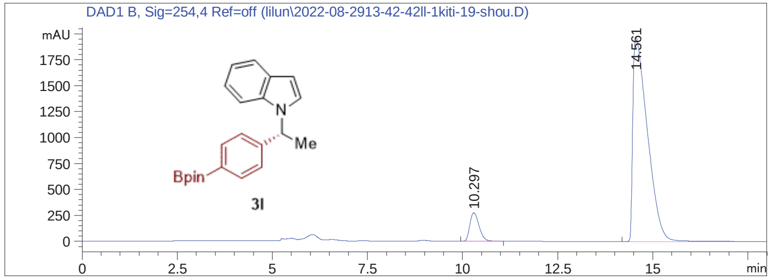

===

Area Percent Report

===

Sorted By : Signal
Multiplier : 1.0000
Dilution : 1.0000
Sample Amount: : 20.00000 [ng/ul] (not used in calc.)
Do not use Multiplier & Dilution Factor with ISTDs

Signal 1: DAD1 B, Sig=254,4 Ref=off

Peak #	RetTime [min]	Type	Width [min]	Area [mAU*s]	Height [mAU]	Area %
1	10.297	BB	0.2673	4645.99316	274.66949	8.1423
2	14.561	BB	0.4091	5.24140e4	1965.85925	91.8577

Totals : 5.70600e4 2240.52875

===

*** End of Report ***

Supplementary Figure 197. HPLC spectra of compound **3l**

Data File E:\LCDATA\Data\lilun\2022-01-1308-26-40ll-1kiti-8-xiao.D
Sample Name: ll-1kiti-8-xiao

===

Acq. Operator : 系统
Sample Operator : 系统
Acq. Instrument : LC1260 Location : 21
Injection Date : 1/13/2022 8:27:34 AM
Inj Volume : 10.000 µl
Acq. Method : E:\LCDATA\Methods\LI-LUN.M
Last changed : 1/12/2022 11:23:46 AM by 系统
Analysis Method : E:\LCDATA\Methods\LI-LUN.M
Last changed : 3/14/2022 3:07:59 PM by 系统
(modified after loading)
Sample Info : oj 80 1 20

Additional Info : Peak(s) manually integrated

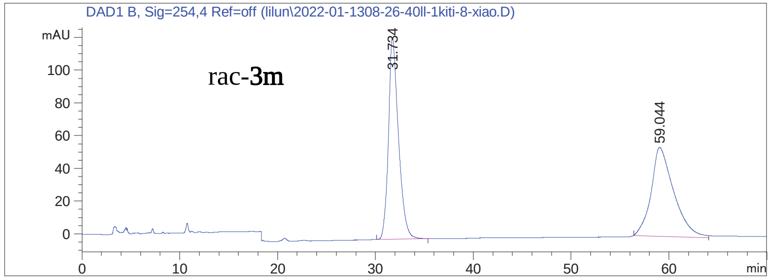

===
Area Percent Report
===

Sorted By : Signal
Multiplier : 1.0000
Dilution : 1.0000
Sample Amount: : 10.00000 [ng/ul] (not used in calc.)
Do not use Multiplier & Dilution Factor with ISTDs

Signal 1: DAD1 B, Sig=254,4 Ref=off

Peak #	RetTime [min]	Type	Width [min]	Area [mAU*s]	Height [mAU]	Area %
1	31.734	BB	1.0266	8724.88281	123.13860	49.9365
2	59.044	MM	2.6850	8747.07715	54.29598	50.0635
Totals :				1.74720e4	177.43458	

===
*** End of Report ***

Data File E:\LCDATA\Data\lilun\2022-01-1309-58-55ll-1kiti-8-shou.D
Sample Name: ll-1kiti-8-shou

===

Acq. Operator : 系统
Sample Operator : 系统
Acq. Instrument : LC1260 Location : 21
Injection Date : 1/13/2022 9:59:51 AM
Inj Volume : 10.000 µl
Acq. Method : E:\LCDATA\Methods\LI-LUN.M
Last changed : 1/12/2022 11:23:46 AM by 系统
Analysis Method : E:\LCDATA\Methods\LI-LUN.M
Last changed : 3/14/2022 3:07:59 PM by 系统
(modified after loading)
Sample Info : oj 80 1 20

Additional Info : Peak(s) manually integrated

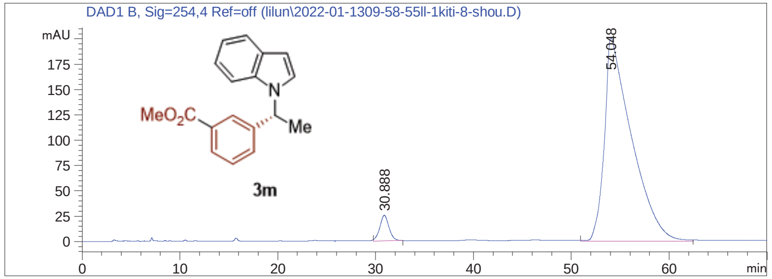

===
Area Percent Report
===

Sorted By : Signal
Multiplier : 1.0000
Dilution : 1.0000
Sample Amount: : 10.00000 [ng/ul] (not used in calc.)
Do not use Multiplier & Dilution Factor with ISTDs

Signal 1: DAD1 B, Sig=254,4 Ref=off

Peak #	RetTime [min]	Type	Width [min]	Area [mAU*s]	Height [mAU]	Area %
1	30.888	MM	1.0858	1657.96887	25.44977	4.0823
2	54.048	MM	3.2271	3.89556e4	201.19260	95.9177
Totals :				4.06136e4	226.64237	

===
*** End of Report ***

Supplementary Figure 198. HPLC spectra of compound **3m**

Data File E:\LCDATA\Data\lilun\2022-08-3009-54-53ll-1kiti-20-xiao.D
Sample Name: ll-1kiti-20-xiao

===
Acq. Operator   : 系统
Sample Operator : 系统
Acq. Instrument : LC1260   Location :   21
Injection Date : 8/30/2022 9:55:53 AM
                                                Inj Volume : 20.000 µl
Acq. Method   : E:\LCDATA\METHODS\LI-LUN.M
Last changed   : 8/30/2022 8:52:05 AM by 系统
                  (modified after loading)
Analysis Method : E:\LCDATA\METHODS\LI-LUN.M
Last changed   : 8/30/2022 10:20:36 AM by 系统
                  (modified after loading)
Sample Info   : oj-H; n-hex:ipro=90:10; 1.0ml/min,20 C

Additional Info : Peak(s) manually integrated

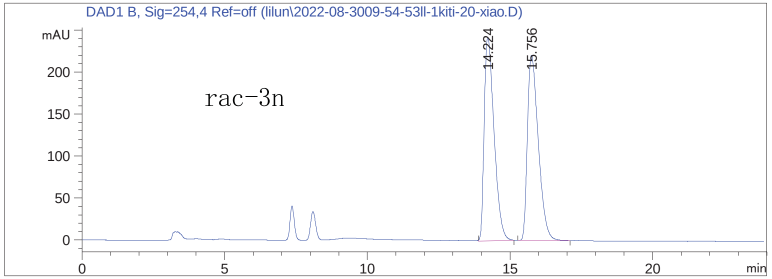

===
Area Percent Report
===

Sorted By   :   Signal
Multiplier   :   1.0000
Dilution   :   1.0000
Sample Amount: :   20.00000 [ng/ul]   (not used in calc.)
Do not use Multiplier & Dilution Factor with ISTDs

Signal 1: DAD1 B, Sig=254,4 Ref=off

Peak #	RetTime [min]	Type	Width [min]	Area [mAU*s]	Height [mAU]	Area %
1	14.224	MM	0.4002	5819.62451	242.39142	49.8391
2	15.756	BB	0.4117	5857.19873	220.65166	50.1609
Totals :				1.16768e4	463.04308	

===
*** End of Report ***

Data File E:\LCDATA\Data\lilun\2022-08-3010-20-15ll-1kiti-20-shou.D
Sample Name: ll-1kiti-20-shou

===
Acq. Operator   : 系统
Sample Operator : 系统
Acq. Instrument : LC1260   Location :   21
Injection Date : 8/30/2022 10:21:15 AM
                                                Inj Volume : 20.000 µl
Acq. Method   : E:\LCDATA\METHODS\LI-LUN.M
Last changed   : 8/30/2022 8:52:05 AM by 系统
                  (modified after loading)
Analysis Method : E:\LCDATA\METHODS\LI-LUN.M
Last changed   : 8/30/2022 10:20:36 AM by 系统
                  (modified after loading)
Sample Info   : oj-H; n-hex:ipro=90:10; 1.0ml/min,20 C

Additional Info : Peak(s) manually integrated

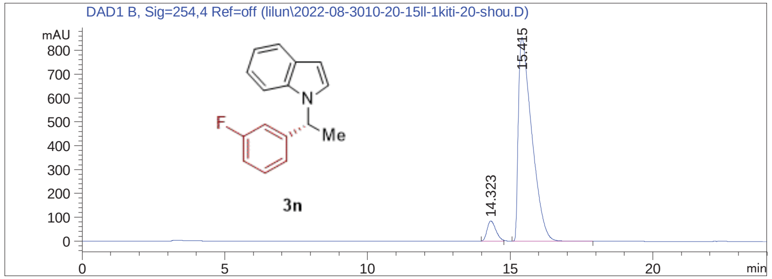

===
Area Percent Report
===

Sorted By   :   Signal
Multiplier   :   1.0000
Dilution   :   1.0000
Sample Amount: :   20.00000 [ng/ul]   (not used in calc.)
Do not use Multiplier & Dilution Factor with ISTDs

Signal 1: DAD1 B, Sig=254,4 Ref=off

Peak #	RetTime [min]	Type	Width [min]	Area [mAU*s]	Height [mAU]	Area %
1	14.323	MM	0.3591	1836.81543	85.25688	6.1625
2	15.415	BB	0.4859	2.79696e4	854.44574	93.8375
Totals :				2.98064e4	939.70262	

===
*** End of Report ***

Supplementary Figure 199. HPLC spectra of compound **3n**

Data File E:\LCDATA\Data\lilun\2022-09-1416-30-3330-5-xiao.D
Sample Name: 30-5-xiao

===

Acq. Operator : 系统
Sample Operator : 系统
Acq. Instrument : LC1260 Location : 21
Injection Date : 9/14/2022 4:31:40 PM
Inj Volume : 20.000 µl
Acq. Method : E:\LCDATA\METHODS\LI-LUN.M
Last changed : 9/14/2022 4:30:11 PM by 系统
(modified after loading)
Analysis Method : E:\LCDATA\METHODS\LI-LUN.M
Last changed : 9/14/2022 3:34:00 PM by 系统
(modified after loading)
Sample Info : od3-H; n-hex:ipro=90:10; 1 ml/min,20 C

Additional Info : Peak(s) manually integrated

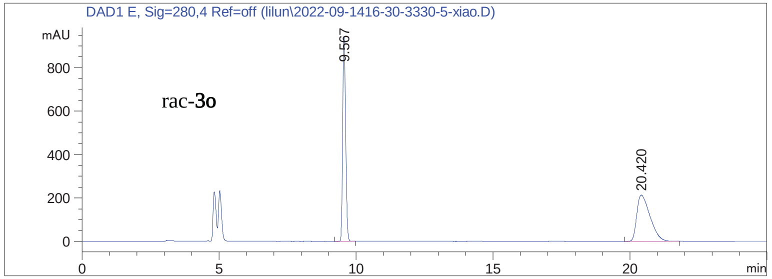

===

Area Percent Report

===

Sorted By : Signal
Multiplier : 1.0000
Dilution : 1.0000
Sample Amount: : 20.00000 [ng/ul] (not used in calc.)
Do not use Multiplier & Dilution Factor with ISTDs

Signal 1: DAD1 E, Sig=280,4 Ref=off

Peak #	RetTime [min]	Type	Width [min]	Area [mAU*s]	Height [mAU]	Area %
1	9.567	BB	0.1212	7176.23975	938.74084	50.0838
2	20.420	BB	0.5174	7152.23242	213.19594	49.9162

Totals : 1.43285e4 1151.93678

===

*** End of Report ***

Data File E:\LCDATA\Data\lilun\2022-09-1415-33-373o-5.D
Sample Name: 3o-5

===

Acq. Operator : 系统
Sample Operator : 系统
Acq. Instrument : LC1260 Location : 21
Injection Date : 9/14/2022 3:34:39 PM
Inj Volume : 20.000 µl
Acq. Method : E:\LCDATA\METHODS\LI-LUN.M
Last changed : 9/14/2022 3:02:31 PM by 系统
(modified after loading)
Analysis Method : E:\LCDATA\METHODS\LI-LUN.M
Last changed : 9/14/2022 3:34:00 PM by 系统
(modified after loading)
Sample Info : od3-H; n-hex:ipro=90:10; 1 ml/min,20 C

Additional Info : Peak(s) manually integrated

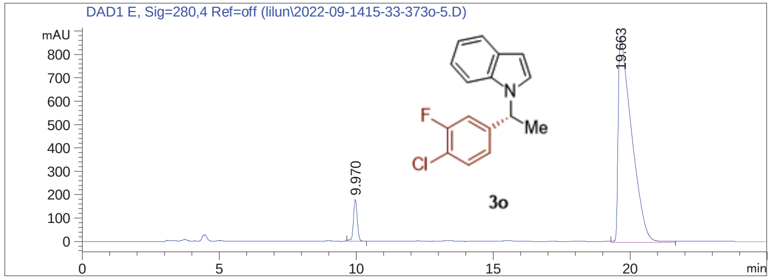

===

Area Percent Report

===

Sorted By : Signal
Multiplier : 1.0000
Dilution : 1.0000
Sample Amount: : 20.00000 [ng/ul] (not used in calc.)
Do not use Multiplier & Dilution Factor with ISTDs

Signal 1: DAD1 E, Sig=280,4 Ref=off

Peak #	RetTime [min]	Type	Width [min]	Area [mAU*s]	Height [mAU]	Area %
1	9.970	MM	0.1634	1754.22668	178.96788	5.5172
2	19.663	MM	0.5717	3.00414e4	875.83057	94.4828

Totals : 3.17957e4 1054.79845

===

*** End of Report ***

Supplementary Figure 200. HPLC spectra of compound **3o**

Data File E:\LCDATA\Data\lilun\2021-11-0509-06-47ll-2-18-1L7.D
Sample Name: ll-2-18-1

===

Acq. Operator : 系统
Sample Operator : 系统
Acq. Instrument : LC1260 Location : 21
Injection Date : 11/5/2021 9:07:42 AM
Inj Volume : 10.000 µl
Acq. Method : E:\LCDATA\Methods\LI-LUN.M
Last changed : 11/5/2021 9:05:24 AM by 系统
(modified after loading)
Analysis Method : E:\LCDATA\Methods\LI-LUN.M
Last changed : 3/14/2022 3:17:18 PM by 系统
(modified after loading)
Sample Info : oj 80:10 1

Additional Info : Peak(s) manually integrated

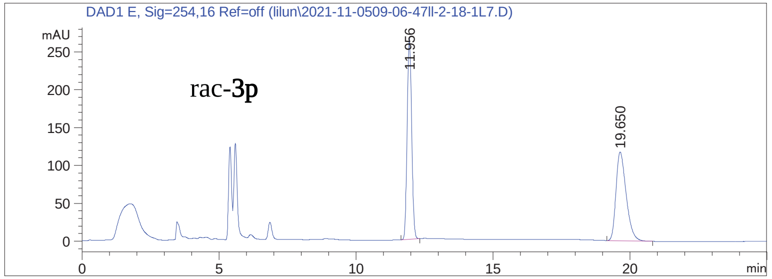

===

Area Percent Report

===

Sorted By : Signal
Multiplier : 1.0000
Dilution : 1.0000
Sample Amount: : 10.00000 [ng/ul] (not used in calc.)
Do not use Multiplier & Dilution Factor with ISTDs

Signal 1: DAD1 E, Sig=254,16 Ref=off

Peak #	RetTime [min]	Type	Width [min]	Area [mAU*s]	Height [mAU]	Area %
1	11.956	BB	0.1800	3059.75098	266.45447	50.1958
2	19.650	BB	0.3979	3035.88354	117.33187	49.8042

Totals : 6095.63452 383.78634

===

*** End of Report ***

Data File E:\LCDATA\Data\lilun\2022-09-1412-45-013p.D
Sample Name: 3p

===

Acq. Operator : 系统
Sample Operator : 系统
Acq. Instrument : LC1260 Location : 21
Injection Date : 9/14/2022 12:45:57 PM
Inj Volume : 10.000 µl
Acq. Method : E:\LCDATA\METHODS\LI-LUN.M
Last changed : 9/14/2022 12:44:53 PM by 系统
(modified after loading)
Analysis Method : E:\LCDATA\METHODS\LI-LUN.M
Last changed : 9/14/2022 11:02:40 AM by 系统
(modified after loading)
Sample Info : od-3H; n-hex:ipro=90:10; 1 ml/min,20 C

Additional Info : Peak(s) manually integrated

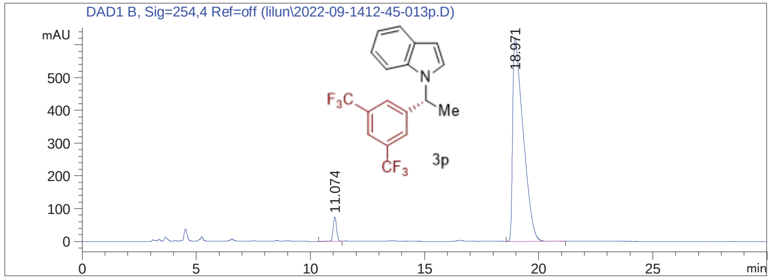

===

Area Percent Report

===

Sorted By : Signal
Multiplier : 1.0000
Dilution : 1.0000
Sample Amount: : 10.00000 [ng/ul] (not used in calc.)
Do not use Multiplier & Dilution Factor with ISTDs

Signal 1: DAD1 B, Sig=254,4 Ref=off

Peak #	RetTime [min]	Type	Width [min]	Area [mAU*s]	Height [mAU]	Area %
1	11.074	BB	0.1614	760.53326	74.31631	3.7036
2	18.971	BB	0.4589	1.97747e4	622.04706	96.2964

Totals : 2.05352e4 696.36337

===

*** End of Report ***

Supplementary Figure 201. HPLC spectra of compound **3p**

```
Data File E:\LCDATA\Data\lilun\2021-10-2922-32-30ll-2-9-1B11.D
Sample Name: ll-2-9-1

=====================================================================
Acq. Operator   : 系统
Sample Operator : 系统
Acq. Instrument : LC1260                          Location :   21
Injection Date  : 10/29/2021 10:33:23 PM
                                                Inj Volume : 5.000 µl
Acq. Method     : E:\LCDATA\Methods\LI-LUN.M
Last changed    : 10/29/2021 10:29:18 PM by 系统
                  (modified after loading)
Analysis Method : E:\LCDATA\Methods\LI-LUN.M
Last changed    : 3/13/2022 9:53:11 AM by 系统
                  (modified after loading)
Sample Info     : 90;10   1

Additional Info : Peak(s) manually integrated
```

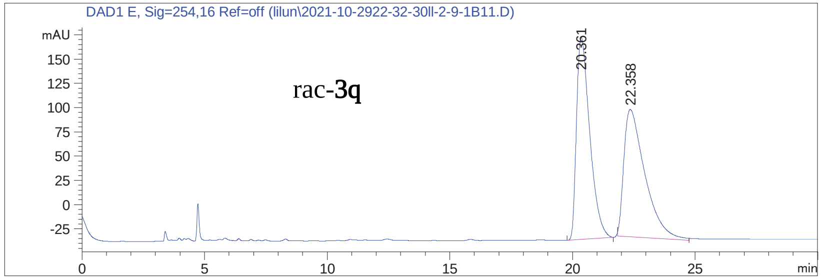


```
=====================================================================
                         Area Percent Report
=====================================================================

Sorted By             :      Signal
Multiplier            :      1.0000
Dilution              :      1.0000
Sample Amount:               :      10.00000  [ng/ul]   (not used in calc.)
Do not use Multiplier & Dilution Factor with ISTDs


Signal 1: DAD1 E, Sig=254,16 Ref=off
```

Peak #	RetTime [min]	Type	Width [min]	Area [mAU*s]	Height [mAU]	Area %
1	20.361	BB	0.5757	7848.25684	208.22025	50.0026
2	22.358	MM	0.9957	7847.44092	131.35771	49.9974

Totals : 1.56957e4 339.57796

```
=====================================================================
                         *** End of Report ***
```



```
Data File E:\LCDATA\Data\lilun\2021-10-2923-28-45ll-2-9-2B11.D
Sample Name: ll-2-9-2

=====================================================================
Acq. Operator   : 系统
Sample Operator : 系统
Acq. Instrument : LC1260                          Location :   21
Injection Date  : 10/29/2021 11:29:35 PM
                                                Inj Volume : 5.000 µl
Acq. Method     : E:\LCDATA\Methods\LI-LUN.M
Last changed    : 10/29/2021 10:29:18 PM by 系统
                  (modified after loading)
Analysis Method : E:\LCDATA\Methods\LI-LUN.M
Last changed    : 3/14/2022 10:18:30 AM by 系统
                  (modified after loading)
Sample Info     : 90;10   1

Additional Info : Peak(s) manually integrated
```

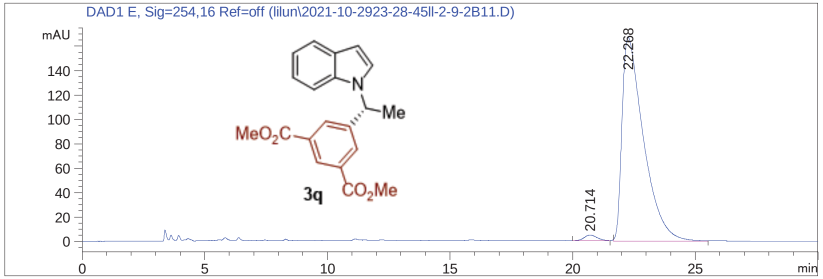


```
=====================================================================
                         Area Percent Report
=====================================================================

Sorted By             :      Signal
Multiplier            :      1.0000
Dilution              :      1.0000
Sample Amount:               :      5.00000  [ng/ul]   (not used in calc.)
Do not use Multiplier & Dilution Factor with ISTDs


Signal 1: DAD1 E, Sig=254,16 Ref=off
```

Peak #	RetTime [min]	Type	Width [min]	Area [mAU*s]	Height [mAU]	Area %
1	20.714	BB	0.5194	158.86668	4.50497	1.5334
2	22.268	MM	1.0183	1.02013e4	166.96851	98.4666

Totals : 1.03602e4 171.47347

```
=====================================================================
                         *** End of Report ***
```

Supplementary Figure 202. HPLC spectra of compound **3q**

Data File E:\LCDATA\Data\lilun\2022-08-3018-36-15ll-1kiti-18-xiao.D
Sample Name: ll-1kiti-18-xiao

===
Acq. Operator   : 系统
Sample Operator : 系统
Acq. Instrument : LC1260   Location :   21
Injection Date : 8/30/2022 6:37:15 PM
                                                Inj Volume : 20.000 µl
Acq. Method   : E:\LCDATA\METHODS\LI-LUN.M
Last changed   : 8/30/2022 5:45:33 PM by 系统
                  (modified after loading)
Analysis Method : E:\LCDATA\METHODS\LI-LUN.M
Last changed   : 8/30/2022 6:37:27 PM by 系统
                  (modified after loading)
Sample Info   : od3-H; n-hex:ipro=90;10; 1.0ml/min,20 C

Additional Info : Peak(s) manually integrated

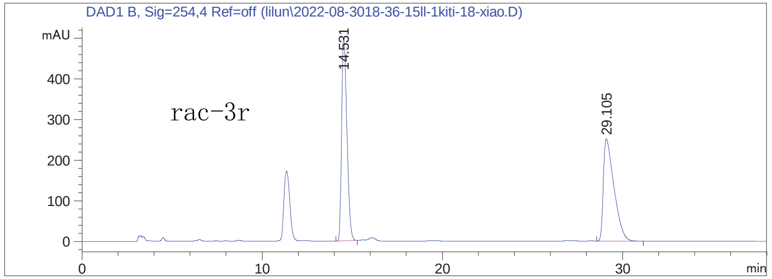

===
Area Percent Report
===

Sorted By   :   Signal
Multiplier   :   1.0000
Dilution   :   1.0000
Sample Amount: :   20.00000 [ng/ul]   (not used in calc.)
Do not use Multiplier & Dilution Factor with ISTDs

Signal 1: DAD1 B, Sig=254,4 Ref=off

Peak #	RetTime [min]	Type	Width [min]	Area [mAU*s]	Height [mAU]	Area %
1	14.531	BB	0.3179	1.02108e4	500.64944	49.8368
2	29.105	BB	0.6028	1.02777e4	251.50726	50.1632

Totals :   2.04885e4   752.15671

===
*** End of Report ***

Data File E:\LCDATA\Data\lilun\2022-08-3017-45-41ll-1kiti-18-shou.D
Sample Name: ll-1kiti-18-shou

===
Acq. Operator   : 系统
Sample Operator : 系统
Acq. Instrument : LC1260   Location :   21
Injection Date : 8/30/2022 5:46:42 PM
                                                Inj Volume : 20.000 µl
Acq. Method   : E:\LCDATA\METHODS\LI-LUN.M
Last changed   : 8/30/2022 5:45:33 PM by 系统
                  (modified after loading)
Analysis Method : E:\LCDATA\METHODS\LI-LUN.M
Last changed   : 8/30/2022 6:37:27 PM by 系统
                  (modified after loading)
Sample Info   : od3-H; n-hex:ipro=90;10; 1.0ml/min,20 C

Additional Info : Peak(s) manually integrated

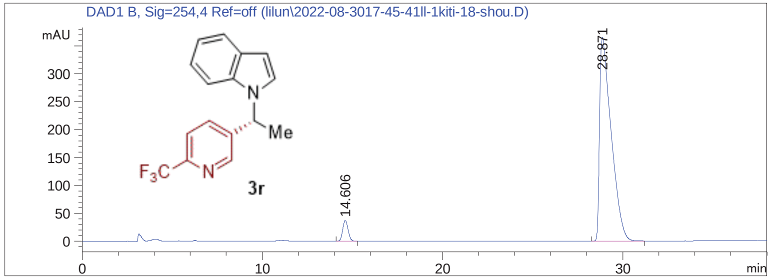

===
Area Percent Report
===

Sorted By   :   Signal
Multiplier   :   1.0000
Dilution   :   1.0000
Sample Amount: :   20.00000 [ng/ul]   (not used in calc.)
Do not use Multiplier & Dilution Factor with ISTDs

Signal 1: DAD1 B, Sig=254,4 Ref=off

Peak #	RetTime [min]	Type	Width [min]	Area [mAU*s]	Height [mAU]	Area %
1	14.606	BB	0.3253	768.96667	37.17370	4.5052
2	28.871	BB	0.6374	1.62996e4	364.68402	95.4948

Totals :   1.70686e4   401.85772

===
*** End of Report ***

Supplementary Figure 203. HPLC spectra of compound **3r**

Data File E:\LCDATA\Data\lilun\2021-11-1817-49-28ll-2-35-111-17Z.D
Sample Name: ll-2-35-1

===
Acq. Operator : 系统
Sample Operator : 系统
Acq. Instrument : LC1260 Location : 21
Injection Date : 11/18/2021 5:50:21 PM
Inj Volume : 10.000 µl
Acq. Method : E:\LCDATA\Methods\LI-LUN.M
Last changed : 11/18/2021 5:48:33 PM by 系统
(modified after loading)
Analysis Method : E:\LCDATA\Methods\LI-LUN.M
Last changed : 4/4/2022 8:31:17 AM by 系统
(modified after loading)
Sample Info : oD 3 90:10 1

Additional Info : Peak(s) manually integrated

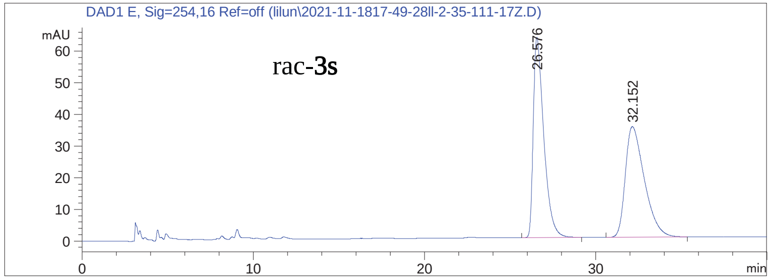

===
Area Percent Report
===

Sorted By : Signal
Multiplier : 1.0000
Dilution : 1.0000
Sample Amount: : 10.00000 [ng/ul] (not used in calc.)
Do not use Multiplier & Dilution Factor with ISTDs

Signal 1: DAD1 E, Sig=254,16 Ref=off

Peak #	RetTime [min]	Type	Width [min]	Area [mAU*s]	Height [mAU]	Area %
1	26.576	BB	0.6434	2705.90137	63.21589	50.0004
2	32.152	BB	1.1166	2705.85986	34.97614	49.9996

Totals : 5411.76123 98.19202

===
*** End of Report ***

Data File E:\LCDATA\Data\lilun\2021-11-1816-57-36ll-2-35-211-17Z.D
Sample Name: ll-2-35-2

===
Acq. Operator : 系统
Sample Operator : 系统
Acq. Instrument : LC1260 Location : 21
Injection Date : 11/18/2021 4:58:30 PM
Inj Volume : 10.000 µl
Acq. Method : E:\LCDATA\Methods\LI-LUN.M
Last changed : 11/18/2021 1:52:25 PM by 系统
(modified after loading)
Analysis Method : E:\LCDATA\Methods\LI-LUN.M
Last changed : 3/14/2022 5:30:42 PM by 系统
(modified after loading)
Sample Info : oD 3 90:10 1

Additional Info : Peak(s) manually integrated

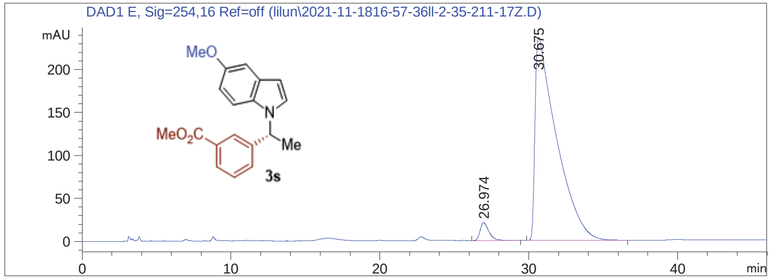

===
Area Percent Report
===

Sorted By : Signal
Multiplier : 1.0000
Dilution : 1.0000
Sample Amount: : 10.00000 [ng/ul] (not used in calc.)
Do not use Multiplier & Dilution Factor with ISTDs

Signal 1: DAD1 E, Sig=254,16 Ref=off

Peak #	RetTime [min]	Type	Width [min]	Area [mAU*s]	Height [mAU]	Area %
1	26.974	BB	0.6212	857.60364	20.79421	3.4969
2	30.675	BB	1.3481	2.36671e4	235.98643	96.5031

Totals : 2.45247e4 256.78064

===
*** End of Report ***

Supplementary Figure 204. HPLC spectra of compound **3s**

Data File E:\LCDATA\Data\lilun\2021-11-1614-36-07ll-2-33-3.D
Sample Name: ll-2-33-3

===

Acq. Operator : 系统
Sample Operator : 系统
Acq. Instrument : LC1260 Location : 21
Injection Date : 11/16/2021 2:37:04 PM
Inj Volume : 10.000 µl
Acq. Method : E:\LCDATA\Methods\LI-LUN.M
Last changed : 11/16/2021 2:22:14 PM by 系统
(modified after loading)
Analysis Method : E:\LCDATA\Methods\LI-LUN.M
Last changed : 3/14/2022 3:38:25 PM by 系统
(modified after loading)
Sample Info : oj 80:20 1

Additional Info : Peak(s) manually integrated

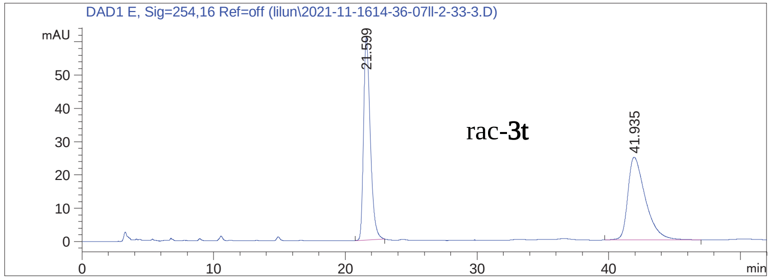

===

Area Percent Report

===

Sorted By : Signal
Multiplier : 1.0000
Dilution : 1.0000
Sample Amount: : 10.00000 [ng/ul] (not used in calc.)
Do not use Multiplier & Dilution Factor with ISTDs

Signal 1: DAD1 E, Sig=254,16 Ref=off

Peak #	RetTime [min]	Type	Width [min]	Area [mAU*s]	Height [mAU]	Area %
1	21.599	BBA	0.5382	2160.48413	60.86028	48.3550
2	41.935	BB	1.2969	2307.47583	24.90530	51.6450
Totals :				4467.95996	85.76558	

===

*** End of Report ***

Data File E:\LCDATA\Data\lilun\2021-11-1615-34-05ll-2-33-4-f.D
Sample Name: ll-2-33-4-f

===

Acq. Operator : 系统
Sample Operator : 系统
Acq. Instrument : LC1260 Location : 21
Injection Date : 11/16/2021 3:34:58 PM
Inj Volume : 10.000 µl
Acq. Method : E:\LCDATA\Methods\LI-LUN.M
Last changed : 11/16/2021 2:22:14 PM by 系统
(modified after loading)
Analysis Method : E:\LCDATA\Methods\LI-LUN.M
Last changed : 3/14/2022 3:38:25 PM by 系统
(modified after loading)
Sample Info : oj 80:20 1

Additional Info : Peak(s) manually integrated

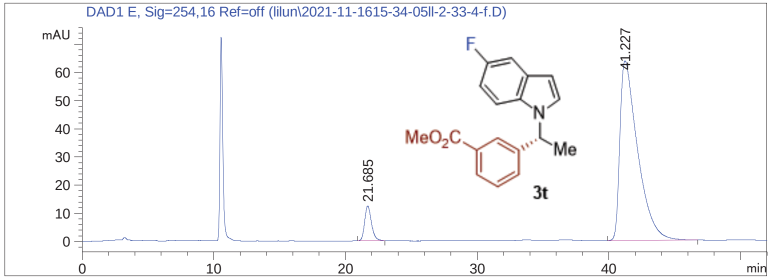

===

Area Percent Report

===

Sorted By : Signal
Multiplier : 1.0000
Dilution : 1.0000
Sample Amount: : 10.00000 [ng/ul] (not used in calc.)
Do not use Multiplier & Dilution Factor with ISTDs

Signal 1: DAD1 E, Sig=254,16 Ref=off

Peak #	RetTime [min]	Type	Width [min]	Area [mAU*s]	Height [mAU]	Area %
1	21.685	BBA	0.5448	441.80841	12.36792	6.8924
2	41.227	BB	1.3346	5968.30371	63.72802	93.1076
Totals :				6410.11212	76.09594	

===

*** End of Report ***

Supplementary Figure 205. HPLC spectra of compound **3t**

Data File E:\LCDATA\Data\lilun\2022-01-0411-24-40ll-1kiti-13-xiao.D
Sample Name: ll-1kiti-13-xiao

===

Acq. Operator : 系统
Sample Operator : 系统
Acq. Instrument : LC1260 Location : 21
Injection Date : 1/4/2022 11:25:37 AM
Inj Volume : 10.000 µl
Acq. Method : E:\LCDATA\Methods\LI-LUN.M
Last changed : 1/4/2022 11:16:22 AM by 系统
(modified after loading)
Analysis Method : E:\LCDATA\Methods\LI-LUN.M
Last changed : 3/14/2022 3:36:21 PM by 系统
(modified after loading)
Sample Info : OJ-H 80/ 1 (20)

Additional Info : Peak(s) manually integrated

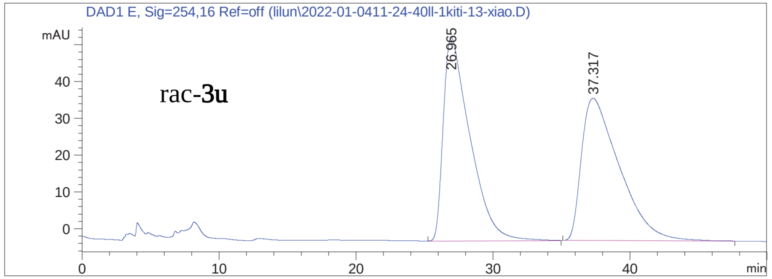

===

Area Percent Report

===

Sorted By : Signal
Multiplier : 1.0000
Dilution : 1.0000
Sample Amount: : 10.00000 [ng/ul] (not used in calc.)
Do not use Multiplier & Dilution Factor with ISTDs

Signal 1: DAD1 E, Sig=254,16 Ref=off

Peak #	RetTime [min]	Type	Width [min]	Area [mAU*s]	Height [mAU]	Area %
1	26.965	BB	1.8613	7473.67432	55.27862	50.0763
2	37.317	BB	2.3316	7450.90723	38.70988	49.9237
Totals :				1.49246e4	93.98850	

===

*** End of Report ***

Data File E:\LCDATA\Data\lilun\2022-01-0412-36-46ll-1kiti-13-shou.D
Sample Name: ll-1kiti-13-shou

===

Acq. Operator : 系统
Sample Operator : 系统
Acq. Instrument : LC1260 Location : 21
Inj Volume : 10.000 µl
Acq. Method : E:\LCDATA\Methods\LI-LUN.M
Last changed : 1/4/2022 11:16:22 AM by 系统
(modified after loading)
Analysis Method : E:\LCDATA\Methods\LI-LUN.M
Last changed : 3/14/2022 3:36:21 PM by 系统
(modified after loading)
Sample Info : OJ-H 80/ 1 (20)

Additional Info : Peak(s) manually integrated

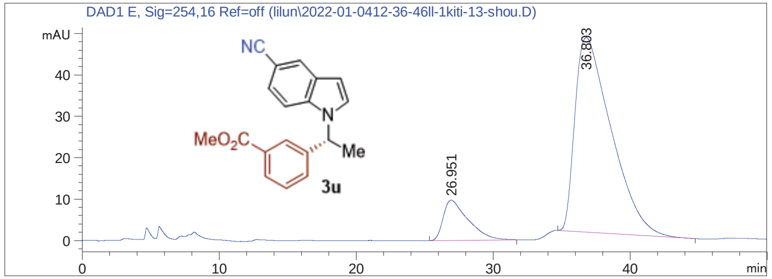

===

Area Percent Report

===

Sorted By : Signal
Multiplier : 1.0000
Dilution : 1.0000
Sample Amount: : 10.00000 [ng/ul] (not used in calc.)
Do not use Multiplier & Dilution Factor with ISTDs

Signal 1: DAD1 E, Sig=254,16 Ref=off

Peak #	RetTime [min]	Type	Width [min]	Area [mAU*s]	Height [mAU]	Area %
1	26.951	BB	1.4936	1217.96143	9.70878	12.3441
2	36.803	BB	2.4089	8648.81250	46.95610	87.6559
Totals :				9866.77393	56.66489	

===

*** End of Report ***

Supplementary Figure 206. HPLC spectra of compound **3u**

Data File E:\LCDATA\Data\lilun\2022-08-2816-40-41ll-1kiti-36-xiao.D
Sample Name: ll-1kiti-36-xiao

===

Acq. Operator : 系统
Sample Operator : 系统
Acq. Instrument : LC1260 Location : 21
Injection Date : 8/28/2022 4:41:41 PM
Inj Volume : 20.000 µl
Acq. Method : E:\LCDATA\METHODS\LI-LUN.M
Last changed : 8/28/2022 4:40:23 PM by 系统
(modified after loading)
Analysis Method : E:\LCDATA\METHODS\LI-LUN.M
Last changed : 8/28/2022 5:26:49 PM by 系统
(modified after loading)
Sample Info : od-H; n-hex:ipro=80:20; 1.0ml/min,20 C

Additional Info : Peak(s) manually integrated

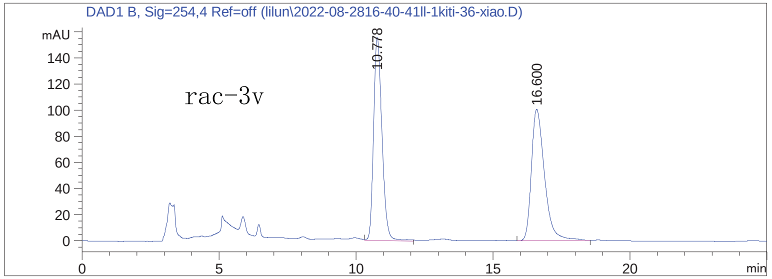

===

Area Percent Report

===

Sorted By : Signal
Multiplier : 1.0000
Dilution : 1.0000
Sample Amount: : 20.00000 [ng/ul] (not used in calc.)
Do not use Multiplier & Dilution Factor with ISTDs

Signal 1: DAD1 B, Sig=254,4 Ref=off

Peak #	RetTime [min]	Type	Width [min]	Area [mAU*s]	Height [mAU]	Area %
1	10.778	MM	0.3552	3311.98486	155.38899	49.7302
2	16.600	BB	0.5060	3347.91528	100.65687	50.2698

Totals : 6659.90015 256.04586

===

*** End of Report ***

Data File E:\LCDATA\Data\lilun\2022-08-2817-23-02ll-1kiti-36-shou.D
Sample Name: ll-1kiti-36-shou

===

Acq. Operator : 系统
Sample Operator : 系统
Acq. Instrument : LC1260 Location : 21
Injection Date : 8/28/2022 5:24:04 PM
Inj Volume : 20.000 µl
Acq. Method : E:\LCDATA\METHODS\LI-LUN.M
Last changed : 8/28/2022 4:40:23 PM by 系统
(modified after loading)
Analysis Method : E:\LCDATA\METHODS\LI-LUN.M
Last changed : 8/28/2022 5:51:45 PM by 系统
(modified after loading)
Sample Info : od-H; n-hex:ipro=80:20; 1.0ml/min,20 C

Additional Info : Peak(s) manually integrated

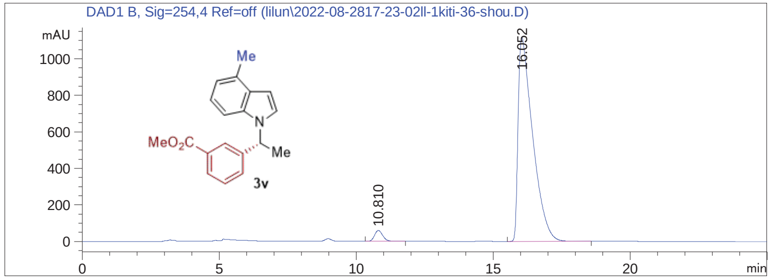

===

Area Percent Report

===

Sorted By : Signal
Multiplier : 1.0000
Dilution : 1.0000
Sample Amount: : 20.00000 [ng/ul] (not used in calc.)
Do not use Multiplier & Dilution Factor with ISTDs

Signal 1: DAD1 B, Sig=254,4 Ref=off

Peak #	RetTime [min]	Type	Width [min]	Area [mAU*s]	Height [mAU]	Area %
1	10.810	BBA	0.3210	1226.32117	59.37033	2.8790
2	16.052	BB	0.5461	4.13695e4	1116.98804	97.1210

Totals : 4.25958e4 1176.35837

===

*** End of Report ***

Supplementary Figure 207. HPLC spectra of compound **3v**

Data File E:\LCDATA\Data\lilun\2021-11-1719-52-25ll-2-35-3.D
Sample Name: ll-2-35-3

===

Acq. Operator : 系统
Sample Operator : 系统
Acq. Instrument : LC1260 Location : 21
Injection Date : 11/17/2021 7:53:19 PM
Inj Volume : 10.000 µl
Acq. Method : E:\LCDATA\Methods\LI-LUN.M
Last changed : 11/17/2021 7:50:32 PM by 系统
(modified after loading)
Analysis Method : E:\LCDATA\Methods\LI-LUN.M
Last changed : 3/18/2022 9:21:44 PM by 系统
(modified after loading)
Sample Info : ad 90:10 1

Additional Info : Peak(s) manually integrated

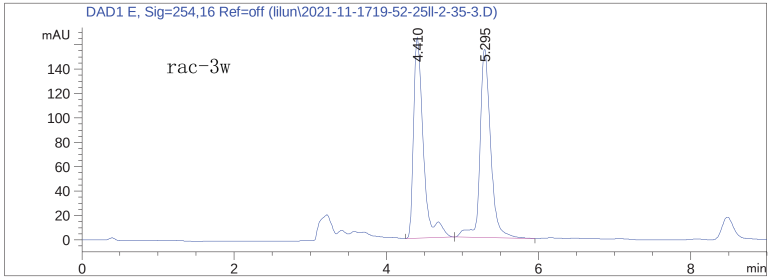

===

Area Percent Report

===

Sorted By : Signal
Multiplier : 1.0000
Dilution : 1.0000
Sample Amount: : 10.00000 [ng/ul] (not used in calc.)
Do not use Multiplier & Dilution Factor with ISTDs

Signal 1: DAD1 E, Sig=254,16 Ref=off

Peak #	RetTime [min]	Type	Width [min]	Area [mAU*s]	Height [mAU]	Area %
1	4.410	BV R	0.1296	1467.78601	164.52724	49.3307
2	5.295	VB R	0.1436	1507.61536	154.45509	50.6693
Totals :				2975.40137	318.98233	

===

*** End of Report ***

Data File E:\LCDATA\Data\lilun\2022-08-2910-51-07ll-1kiti-17-shou.D
Sample Name: ll-1kiti-17-shou

===

Acq. Operator : 系统
Sample Operator : 系统
Acq. Instrument : LC1260 Location : 21
Injection Date : 8/29/2022 10:52:08 AM
Inj Volume : 20.000 µl
Acq. Method : E:\LCDATA\METHODS\LI-LUN.M
Last changed : 8/29/2022 10:26:29 AM by 系统
(modified after loading)
Analysis Method : E:\LCDATA\METHODS\LI-LUN.M
Last changed : 8/29/2022 11:03:28 AM by 系统
(modified after loading)
Sample Info : od3-H; n-hex:ipro=90:10; 1.0ml/min,20 C

Additional Info : Peak(s) manually integrated

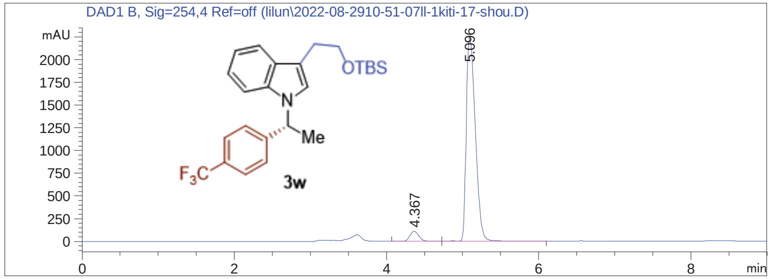

===

Area Percent Report

===

Sorted By : Signal
Multiplier : 1.0000
Dilution : 1.0000
Sample Amount: : 20.00000 [ng/ul] (not used in calc.)
Do not use Multiplier & Dilution Factor with ISTDs

Signal 1: DAD1 B, Sig=254,4 Ref=off

Peak #	RetTime [min]	Type	Width [min]	Area [mAU*s]	Height [mAU]	Area %
1	4.367	BB	0.1326	903.49713	109.10658	4.6278
2	5.096	VV R	0.1327	1.86198e4	2241.67944	95.3722
Totals :				1.95233e4	2350.78603	

===

*** End of Report ***

Supplementary Figure 208. HPLC spectra of compound **3w**

Data File E:\LCDATA\Data\HMQ\2022-01-0318-17-21wxm-2-0031.D
Sample Name: wxm-2-0031

===

Acq. Operator : 系统
Sample Operator : 系统
Acq. Instrument : LC1260 Location : 23
Injection Date : 1/3/2022 6:18:20 PM
Inj Volume : 10.000 µl
Acq. Method : E:\LCDATA\Methods\LI-LUN.M
Last changed : 1/3/2022 5:59:59 PM by 系统
(modified after loading)
Analysis Method : E:\LCDATA\Methods\LI-LUN.M
Last changed : 3/14/2022 4:00:13 PM by 系统
(modified after loading)
Sample Info : OJ-H 98/ 1 (20)xiao

Additional Info : Peak(s) manually integrated

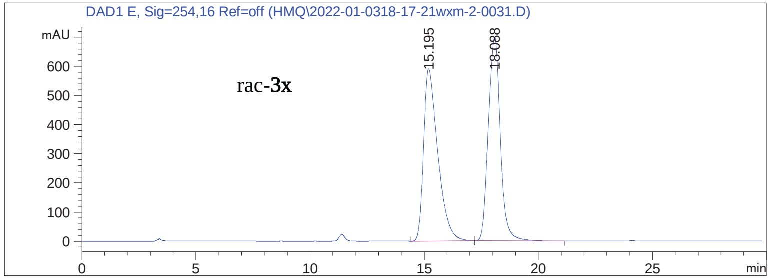

===

Area Percent Report

===

Sorted By : Signal
Multiplier : 1.0000
Dilution : 1.0000
Sample Amount: : 10.00000 [ng/ul] (not used in calc.)
Do not use Multiplier & Dilution Factor with ISTDs

Signal 1: DAD1 E, Sig=254,16 Ref=off

Peak #	RetTime [min]	Type	Width [min]	Area [mAU*s]	Height [mAU]	Area %
1	15.195	BB	0.6400	2.55563e4	591.54681	50.0089
2	18.088	BB	0.5846	2.55472e4	698.62543	49.9911
Totals :				5.11036e4	1290.17224	

===

*** End of Report ***

Data File E:\LCDATA\Data\HMQ\2022-01-0318-48-57wxm-2-011a.D
Sample Name: wxm-2-011a

===

Acq. Operator : 系统
Sample Operator : 系统
Acq. Instrument : LC1260 Location : 24
Injection Date : 1/3/2022 6:49:55 PM
Inj Volume : 10.000 µl
Acq. Method : E:\LCDATA\Methods\LI-LUN.M
Last changed : 1/3/2022 5:59:59 PM by 系统
(modified after loading)
Analysis Method : E:\LCDATA\Methods\LI-LUN.M
Last changed : 3/14/2022 4:00:13 PM by 系统
(modified after loading)
Sample Info : OJ-H 98/ 1 (20)shou

Additional Info : Peak(s) manually integrated

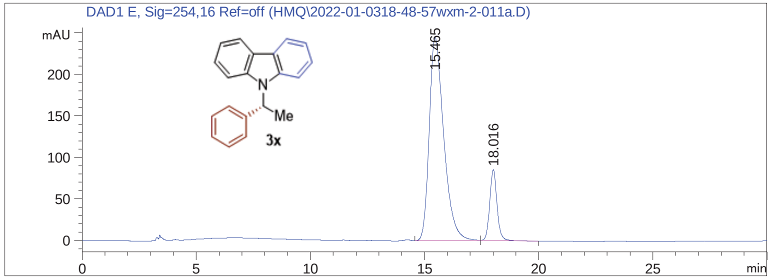

===

Area Percent Report

===

Sorted By : Signal
Multiplier : 1.0000
Dilution : 1.0000
Sample Amount: : 10.00000 [ng/ul] (not used in calc.)
Do not use Multiplier & Dilution Factor with ISTDs

Signal 1: DAD1 E, Sig=254,16 Ref=off

Peak #	RetTime [min]	Type	Width [min]	Area [mAU*s]	Height [mAU]	Area %
1	15.465	BB	0.6669	1.08013e4	244.75511	85.1235
2	18.016	BB	0.3404	1887.67590	85.22446	14.8765
Totals :				1.26890e4	329.97958	

===

*** End of Report ***

Supplementary Figure 209. HPLC spectra of compound **3x**

Data File E:\LCDATA\Data\lilun\2022-01-1709-54-09ll-1kiti-44-xiao.D
Sample Name: ll-1kiti-44-xiao

===

Acq. Operator : 系统
Sample Operator : 系统
Acq. Instrument : LC1260 Location : 21

Inj Volume : 10.000 µl

Acq. Method : E:\LCDATA\Methods\LI-LUN.M
Last changed : 1/12/2022 11:23:46 AM by 系统
Analysis Method : E:\LCDATA\Methods\LI-LUN.M
Last changed : 3/14/2022 4:05:27 PM by 系统
(modified after loading)
Sample Info : 90/1od-3;20C

Additional Info : Peak(s) manually integrated

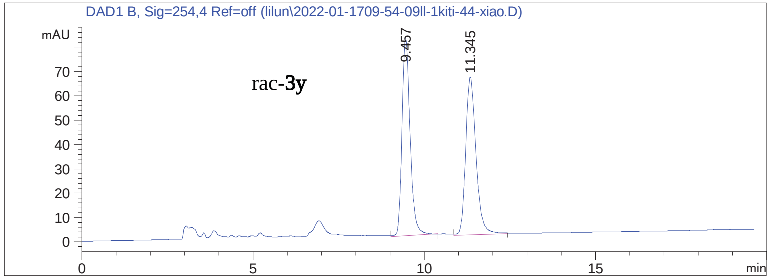

===

Area Percent Report

===

Sorted By : Signal
Multiplier : 1.0000
Dilution : 1.0000
Sample Amount: : 20.00000 [ng/ul] (not used in calc.)
Do not use Multiplier & Dilution Factor with ISTDs

Signal 1: DAD1 B, Sig=254,4 Ref=off

Peak #	RetTime [min]	Type	Width [min]	Area [mAU*s]	Height [mAU]	Area %
1	9.457	MM	0.2740	1341.65222	81.60913	49.6975
2	11.345	MM	0.3487	1357.98303	64.90281	50.3025

Totals : 2699.63525 146.51194

===

*** End of Report ***

Data File E:\LCDATA\Data\lilun\2022-01-1710-28-49ll-1kiti-44-shou.D
Sample Name: ll-1kiti-44-shou

===

Acq. Operator : 系统
Sample Operator : 系统
Acq. Instrument : LC1260 Location : 21
Injection Date : 1/17/2022 10:29:44 AM

Inj Volume : 10.000 µl

Acq. Method : E:\LCDATA\Methods\LI-LUN.M
Last changed : 1/12/2022 11:23:46 AM by 系统
Analysis Method : E:\LCDATA\Methods\LI-LUN.M
Last changed : 3/14/2022 4:05:27 PM by 系统
(modified after loading)
Sample Info : 90/1od-3;20C

Additional Info : Peak(s) manually integrated

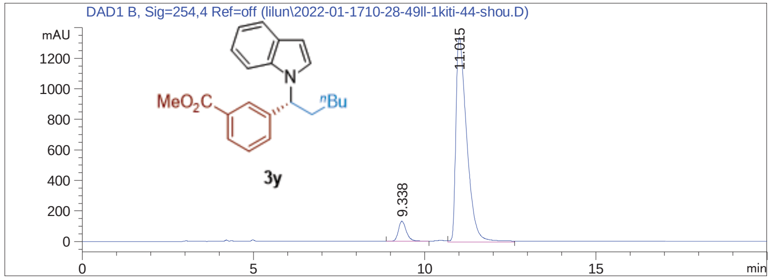

===

Area Percent Report

===

Sorted By : Signal
Multiplier : 1.0000
Dilution : 1.0000
Sample Amount: : 10.00000 [ng/ul] (not used in calc.)
Do not use Multiplier & Dilution Factor with ISTDs

Signal 1: DAD1 B, Sig=254,4 Ref=off

Peak #	RetTime [min]	Type	Width [min]	Area [mAU*s]	Height [mAU]	Area %
1	9.338	BB	0.2440	2092.97217	131.26680	6.5994
2	11.015	MM	0.3687	2.96216e4	1338.86169	93.4006

Totals : 3.17146e4 1470.12849

===

*** End of Report ***

Supplementary Figure 210. HPLC spectra of compound **3y**

Data File E:\LCDATA\Data\lilun\2022-01-2813-15-46ll-1kiti-48-xiao.D
Sample Name: ll-1kiti-48-xiao

===

Acq. Operator : 系统
Sample Operator : 系统
Acq. Instrument : LC1260 Location : 21
Injection Date : 1/28/2022 1:16:39 PM
Inj Volume : 10.000 µl
Acq. Method : E:\LCDATA\Methods\LI-LUN.M
Last changed : 1/28/2022 1:15:24 PM by 系统
(modified after loading)
Analysis Method : E:\LCDATA\Methods\LI-LUN.M
Last changed : 3/14/2022 4:07:55 PM by 系统
(modified after loading)
Sample Info : 90/1 ;oD-H;20C

Additional Info : Peak(s) manually integrated

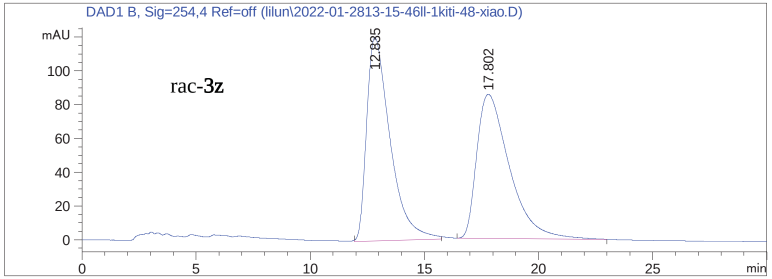

===

Area Percent Report

===

Sorted By : Signal
Multiplier : 1.0000
Dilution : 1.0000
Sample Amount: : 10.00000 [ng/ul] (not used in calc.)
Do not use Multiplier & Dilution Factor with ISTDs

Signal 1: DAD1 B, Sig=254,4 Ref=off

Peak #	RetTime [min]	Type	Width [min]	Area [mAU*s]	Height [mAU]	Area %
1	12.835	MM	1.1931	8628.98145	120.53976	50.4354
2	17.802	BBA	1.4630	8480.00098	85.36147	49.5646

Totals : 1.71090e4 205.90123

===

*** End of Report ***

Data File E:\LCDATA\Data\lilun\2022-01-2813-55-35ll-1kiti-48-shou.D
Sample Name: ll-1kiti-48-shou

===

Acq. Operator : 系统
Sample Operator : 系统
Acq. Instrument : LC1260 Location : 21
Injection Date : 1/28/2022 1:56:27 PM
Inj Volume : 10.000 µl
Acq. Method : E:\LCDATA\Methods\LI-LUN.M
Last changed : 1/28/2022 1:15:24 PM by 系统
(modified after loading)
Analysis Method : E:\LCDATA\Methods\LI-LUN.M
Last changed : 3/14/2022 4:07:55 PM by 系统
(modified after loading)
Sample Info : 80/1 ;oD-H;20C

Additional Info : Peak(s) manually integrated

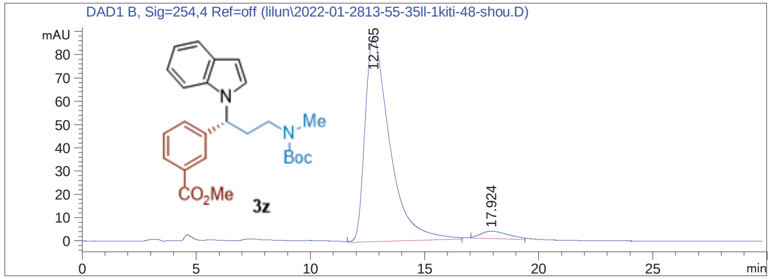

===

Area Percent Report

===

Sorted By : Signal
Multiplier : 1.0000
Dilution : 1.0000
Sample Amount: : 10.00000 [ng/ul] (not used in calc.)
Do not use Multiplier & Dilution Factor with ISTDs

Signal 1: DAD1 B, Sig=254,4 Ref=off

Peak #	RetTime [min]	Type	Width [min]	Area [mAU*s]	Height [mAU]	Area %
1	12.765	MM	1.2584	6617.48535	87.64330	96.1171
2	17.924	MM	1.4093	267.33115	3.16147	3.8829

Totals : 6884.81650 90.80477

===

*** End of Report ***

Supplementary Figure 211. HPLC spectra of compound **3z**

Data File E:\LCDATA\Data\lilun\2022-01-1914-59-07ll-1kiti-47-xiao.D
Sample Name: ll-1kiti-47-xiao

===

Acq. Operator : 系统
Sample Operator : 系统
Acq. Instrument : LC1260 Location : 21
Injection Date : 1/19/2022 3:00:04 PM
Inj Volume : 20.000 µl
Acq. Method : E:\LCDATA\Methods\LI-LUN.M
Last changed : 1/19/2022 2:58:56 PM by 系统
(modified after loading)
Analysis Method : E:\LCDATA\Methods\LI-LUN.M
Last changed : 3/14/2022 4:11:00 PM by 系统
(modified after loading)
Sample Info : 90/1 ad ;20C

Additional Info : Peak(s) manually integrated

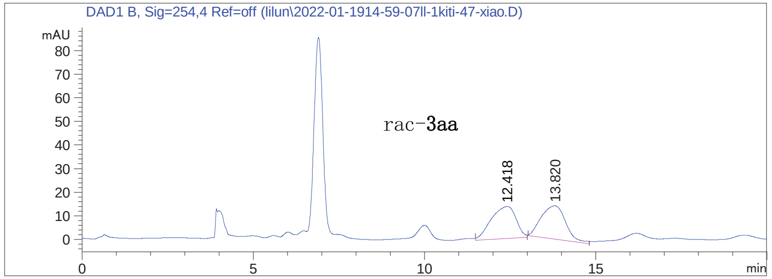

===

Area Percent Report

===

Sorted By : Signal
Multiplier : 1.0000
Dilution : 1.0000
Sample Amount: : 20.00000 [ng/ul] (not used in calc.)
Do not use Multiplier & Dilution Factor with ISTDs

Signal 1: DAD1 B, Sig=254,4 Ref=off

Peak #	RetTime [min]	Type	Width [min]	Area [mAU*s]	Height [mAU]	Area %
1	12.418	MM	0.8462	687.07147	13.53269	49.7847
2	13.820	MM	0.8198	693.01440	14.08932	50.2153

Totals : 1380.08588 27.62201

===

*** End of Report ***

Data File E:\LCDATA\Data\lilun\2022-01-1920-04-07ll-1kiti-47-shou.D
Sample Name: ll-1kiti-47-shou

===

Acq. Operator : 系统
Sample Operator : 系统
Acq. Instrument : LC1260 Location : 21
Injection Date : 1/19/2022 8:05:01 PM
Inj Volume : 10.000 µl
Acq. Method : E:\LCDATA\Methods\LI-LUN.M
Last changed : 1/19/2022 7:46:41 PM by 系统
(modified after loading)
Analysis Method : E:\LCDATA\Methods\LI-LUN.M
Last changed : 3/14/2022 4:11:00 PM by 系统
(modified after loading)
Sample Info : 90/1 oj ;20C

Additional Info : Peak(s) manually integrated

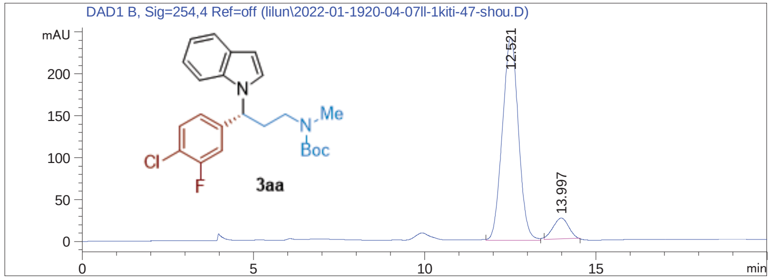

===

Area Percent Report

===

Sorted By : Signal
Multiplier : 1.0000
Dilution : 1.0000
Sample Amount: : 10.00000 [ng/ul] (not used in calc.)
Do not use Multiplier & Dilution Factor with ISTDs

Signal 1: DAD1 B, Sig=254,4 Ref=off

Peak #	RetTime [min]	Type	Width [min]	Area [mAU*s]	Height [mAU]	Area %
1	12.521	MM	0.5428	7877.72656	241.89648	91.0587
2	13.997	MM	0.5221	773.53949	24.69509	8.9413

Totals : 8651.26605 266.59158

===

*** End of Report ***

Supplementary Figure 212. HPLC spectra of compound **3aa**

Data File E:\LCDATA\Data\lilun\2022-01-2817-34-06ll-1kiti-49-shou.D
Sample Name: ll-1kiti-49-shou

===

Acq. Operator : 系统
Sample Operator : 系统
Acq. Instrument : LC1260 Location : 21
Injection Date : 1/28/2022 5:35:01 PM
Inj Volume : 10.000 µl
Acq. Method : E:\LCDATA\Methods\LI-LUN.M
Last changed : 1/28/2022 3:44:32 PM by 系统
(modified after loading)
Analysis Method : E:\LCDATA\Methods\LI-LUN.M
Last changed : 3/14/2022 4:17:21 PM by 系统
(modified after loading)
Sample Info : 90/1 ;oD-H;20C

Additional Info : Peak(s) manually integrated

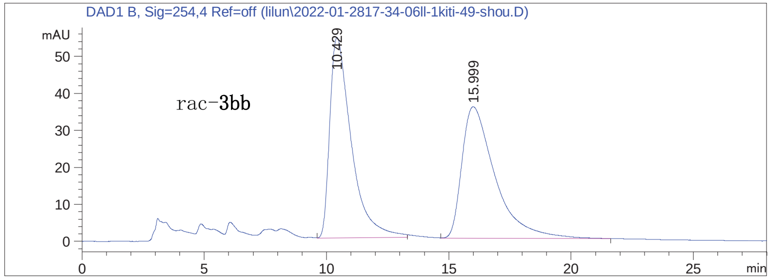

===

Area Percent Report

===

Sorted By : Signal
Multiplier : 1.0000
Dilution : 1.0000
Sample Amount: : 10.00000 [ng/ul] (not used in calc.)
Do not use Multiplier & Dilution Factor with ISTDs

Signal 1: DAD1 B, Sig=254,4 Ref=off

Peak #	RetTime [min]	Type	Width [min]	Area [mAU*s]	Height [mAU]	Area %
1	10.429	MM	1.0195	3313.83130	54.17661	49.7146
2	15.999	BB	1.3477	3351.87500	35.49584	50.2854
Totals :				6665.70630	89.67245	

===

*** End of Report ***

Data File E:\LCDATA\Data\lilun\2022-01-2816-27-01ll-1kiti-49-shou.D
Sample Name: ll-1kiti-49-shou

===

Acq. Operator : 系统
Sample Operator : 系统
Acq. Instrument : LC1260 Location : 21
Injection Date : 1/28/2022 4:27:52 PM
Inj Volume : 10.000 µl
Acq. Method : E:\LCDATA\Methods\LI-LUN.M
Last changed : 1/28/2022 3:44:32 PM by 系统
(modified after loading)
Analysis Method : E:\LCDATA\Methods\LI-LUN.M
Last changed : 3/14/2022 4:17:21 PM by 系统
(modified after loading)
Sample Info : 90/1 ;oD-H;20C

Additional Info : Peak(s) manually integrated

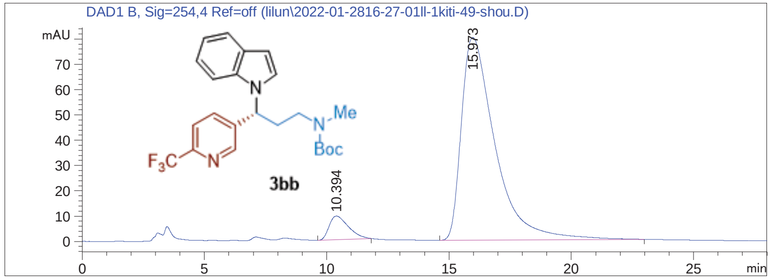

===

Area Percent Report

===

Sorted By : Signal
Multiplier : 1.0000
Dilution : 1.0000
Sample Amount: : 10.00000 [ng/ul] (not used in calc.)
Do not use Multiplier & Dilution Factor with ISTDs

Signal 1: DAD1 B, Sig=254,4 Ref=off

Peak #	RetTime [min]	Type	Width [min]	Area [mAU*s]	Height [mAU]	Area %
1	10.394	BBA	0.7731	509.35898	9.35638	6.2219
2	15.973	BBA	1.3825	7677.15674	80.07918	93.7781
Totals :				8186.51572	89.43556	

===

*** End of Report ***

Supplementary Figure 213. HPLC spectra of compound **3bb**

Data File E:\LCDATA\Data\lilun\2022-01-0915-37-28LL-38.D
Sample Name: LL-38

===

Acq. Operator : 系统
Sample Operator : 系统
Acq. Instrument : LC1260 Location : 21
Injection Date : 1/9/2022 3:38:38 PM
Inj Volume : 10.000 µl
Acq. Method : E:\LCDATA\Methods\LI-LUN.M
Last changed : 1/9/2022 3:37:25 PM by 系统
(modified after loading)
Analysis Method : E:\LCDATA\Methods\LI-LUN.M
Last changed : 3/14/2022 4:22:14 PM by 系统
(modified after loading)
Sample Info : n-hex/i-pro=90:10 1ml/min,AD-H

Additional Info : Peak(s) manually integrated

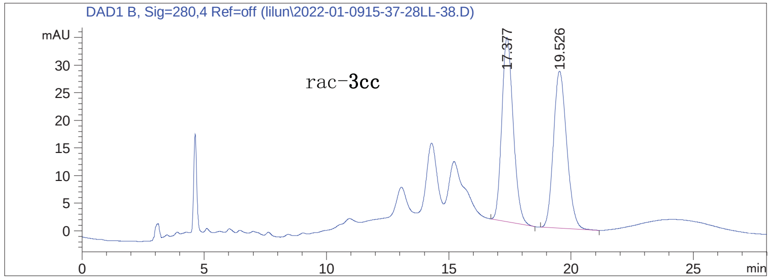

===
Area Percent Report
===

Sorted By : Signal
Multiplier : 1.0000
Dilution : 1.0000
Sample Amount: : 10.00000 [ng/ul] (not used in calc.)
Do not use Multiplier & Dilution Factor with ISTDs

Signal 1: DAD1 B, Sig=280,4 Ref=off

Peak #	RetTime [min]	Type	Width [min]	Area [mAU*s]	Height [mAU]	Area %
1	17.377	BB	0.5035	1084.70325	33.34973	50.1852
2	19.526	BB	0.5915	1076.69751	28.45259	49.8148

Totals : 2161.40076 61.80232

===
*** End of Report ***

Data File E:\LCDATA\Data\lilun\2022-01-0916-46-46LL-38-SHOU.D
Sample Name: LL-38-SHOU

===

Acq. Operator : 系统
Sample Operator : 系统
Acq. Instrument : LC1260 Location : 21
Injection Date : 1/9/2022 4:47:42 PM
Inj Volume : 10.000 µl
Acq. Method : E:\LCDATA\Methods\LI-LUN.M
Last changed : 1/9/2022 3:37:25 PM by 系统
(modified after loading)
Analysis Method : E:\LCDATA\Methods\LI-LUN.M
Last changed : 3/14/2022 4:22:14 PM by 系统
(modified after loading)
Sample Info : n-hex/i-pro=90:10 1ml/min,AD-H

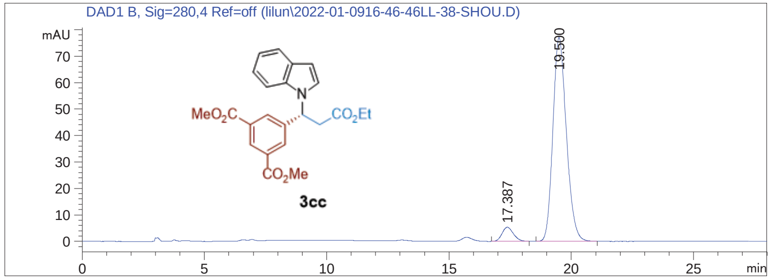

===
Area Percent Report
===

Sorted By : Signal
Multiplier : 1.0000
Dilution : 1.0000
Sample Amount: : 10.00000 [ng/ul] (not used in calc.)
Do not use Multiplier & Dilution Factor with ISTDs

Signal 1: DAD1 B, Sig=280,4 Ref=off

Peak #	RetTime [min]	Type	Width [min]	Area [mAU*s]	Height [mAU]	Area %
1	17.387	BB	0.4629	173.45509	5.36873	5.5489
2	19.500	BB	0.5912	2952.46973	77.02674	94.4511

Totals : 3125.92482 82.39547

===
*** End of Report ***

Supplementary Figure 214. HPLC spectra of compound **3cc**

Data File E:\LCDATA\Data\lilun\2021-12-1716-27-52.D
Sample Name: ll-2-76-13-shou

===

Acq. Operator : 系统
Sample Operator : 系统
Acq. Instrument : LC1260 Location : 21
Injection Date : 12/17/2021 4:28:49 PM
Inj Volume : 10.000 µl
Acq. Method : E:\LCDATA\Methods\LI-LUN.M
Last changed : 12/17/2021 4:27:46 PM by 系统
(modified after loading)
Analysis Method : E:\LCDATA\Methods\LI-LUN.M
Last changed : 3/14/2022 4:24:16 PM by 系统
(modified after loading)
Sample Info : AD-H 98/0.8

Additional Info : Peak(s) manually integrated

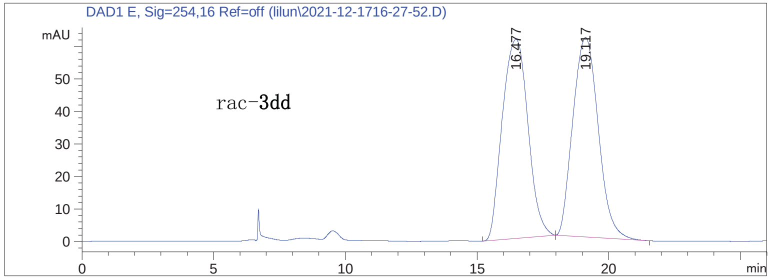

===

Area Percent Report

===

Sorted By : Signal
Multiplier : 1.0000
Dilution : 1.0000
Sample Amount: : 10.00000 [ng/ul] (not used in calc.)
Do not use Multiplier & Dilution Factor with ISTDs

Signal 1: DAD1 E, Sig=254,16 Ref=off

Peak #	RetTime [min]	Type	Width [min]	Area [mAU*s]	Height [mAU]	Area %
1	16.477	BB	1.1060	4144.53906	61.20870	50.1996
2	19.117	BB	1.0605	4111.57813	61.30453	49.8004
Totals :				8256.11719	122.51323	

===

*** End of Report ***

Data File E:\LCDATA\Data\lilun\2021-12-1717-00-31.D
Sample Name: ll-2-76-13-shou

===

Acq. Operator : 系统
Sample Operator : 系统
Acq. Instrument : LC1260 Location : 21
Injection Date : 12/17/2021 5:01:27 PM
Inj Volume : 10.000 µl
Acq. Method : E:\LCDATA\Methods\LI-LUN.M
Last changed : 12/17/2021 4:27:46 PM by 系统
(modified after loading)
Analysis Method : E:\LCDATA\Methods\LI-LUN.M
Last changed : 3/14/2022 4:24:16 PM by 系统
(modified after loading)
Sample Info : AD-H 98/0.5

Additional Info : Peak(s) manually integrated

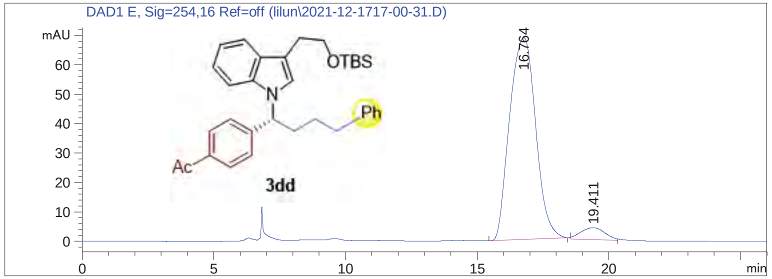

===

Area Percent Report

===

Sorted By : Signal
Multiplier : 1.0000
Dilution : 1.0000
Sample Amount: : 10.00000 [ng/ul] (not used in calc.)
Do not use Multiplier & Dilution Factor with ISTDs

Signal 1: DAD1 E, Sig=254,16 Ref=off

Peak #	RetTime [min]	Type	Width [min]	Area [mAU*s]	Height [mAU]	Area %
1	16.764	BB	1.1307	4710.31104	68.63148	94.7779
2	19.411	MM	1.0652	259.52893	4.06066	5.2221
Totals :				4969.83997	72.69215	

===

*** End of Report ***

Supplementary Figure 215. HPLC spectra of compound **3dd**

```
Data File E:\LCDATA\Data\HMQ\2022-01-0317-09-20ll-2-30-3.D
Sample Name: ll-2-30-3

    =====================================================================
    Acq. Operator   : 系统
    Sample Operator : 系统
    Acq. Instrument : LC1260                          Location :   21
    Injection Date  : 1/3/2022 5:10:14 PM
                                                    Inj Volume : 10.000 µl
    Acq. Method     : E:\LCDATA\Methods\LI-LUN.M
    Last changed    : 1/3/2022 4:26:20 PM by 系统
                      (modified after loading)
    Analysis Method : E:\LCDATA\Methods\LI-LUN.M
    Last changed    : 3/14/2022 4:29:39 PM by 系统
                      (modified after loading)
    Sample Info     : AD-H  95/ 1 (20)

    Additional Info : Peak(s) manually integrated
```

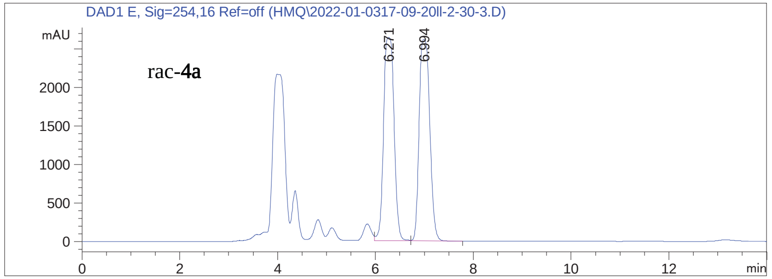


```
    =====================================================================
                         Area Percent Report
    =====================================================================

    Sorted By             :      Signal
    Multiplier            :      1.0000
    Dilution              :      1.0000
    Sample Amount:               :     10.00000  [ng/ul]   (not used in calc.)
    Do not use Multiplier & Dilution Factor with ISTDs


    Signal 1: DAD1 E, Sig=254,16 Ref=off

    Peak RetTime Type  Width     Area      Height     Area
      #   [min]        [min]   [mAU*s]     [mAU]        %
    ----|-------|----|-------|----------|----------|--------|
       1   6.271 FM    0.2474 3.92114e4  2641.86328  49.8640
       2   6.994 VB    0.2395 3.94253e4  2621.33008  50.1360

    Totals :                  7.86367e4  5263.19336


    =====================================================================
                          *** End of Report ***
```



```
Data File E:\LCDATA\Data\HMQ\2022-01-0218-39-12wxm-2-0115.D
Sample Name: wxm-2-0115

    =====================================================================
    Acq. Operator   : 系统
    Sample Operator : 系统
    Acq. Instrument : LC1260                          Location :   24
    Injection Date  : 1/2/2022 6:40:07 PM
                                                    Inj Volume : 10.000 µl
    Acq. Method     : E:\LCDATA\Methods\LI-LUN.M
    Last changed    : 1/2/2022 4:42:06 PM by 系统
                      (modified after loading)
    Analysis Method : E:\LCDATA\Methods\LI-LUN.M
    Last changed    : 3/14/2022 4:29:39 PM by 系统
                      (modified after loading)
    Sample Info     : AD-H  95/ 1 (18)

    Additional Info : Peak(s) manually integrated
```

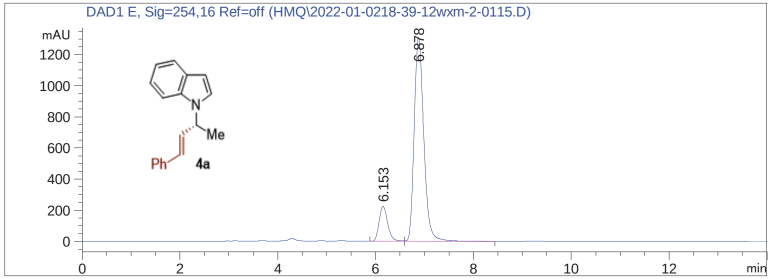


```
    =====================================================================
                         Area Percent Report
    =====================================================================

    Sorted By             :      Signal
    Multiplier            :      1.0000
    Dilution              :      1.0000
    Sample Amount:               :     10.00000  [ng/ul]   (not used in calc.)
    Do not use Multiplier & Dilution Factor with ISTDs


    Signal 1: DAD1 E, Sig=254,16 Ref=off

    Peak RetTime Type  Width     Area      Height     Area
      #   [min]        [min]   [mAU*s]     [mAU]        %
    ----|-------|----|-------|----------|----------|--------|
       1   6.153 BV    0.1903 2744.50171  225.00960  13.3294
       2   6.878 VB    0.2110 1.78454e4  1309.23010  86.6706

    Totals :                  2.05899e4  1534.23970


    =====================================================================
                          *** End of Report ***
```

Supplementary Figure 216. HPLC spectra of compound **4a**

Data File E:\LCDATA\Data\lilun\2022-01-2823-07-36ll-135-1-xiao.D
Sample Name: ll-135-1-xiao

===

Acq. Operator : 系统
Sample Operator : 系统
Acq. Instrument : LC1260 Location : 21
Injection Date : 1/28/2022 11:08:37 PM
Inj Volume : 20.000 µl
Acq. Method : E:\LCDATA\Methods\LI-LUN.M
Last changed : 1/28/2022 10:55:15 PM by 系统
(modified after loading)
Analysis Method : E:\LCDATA\Methods\LI-LUN.M
Last changed : 3/14/2022 4:36:33 PM by 系统
(modified after loading)
Sample Info : 90/1 ;OJ-H;20C

Additional Info : Peak(s) manually integrated

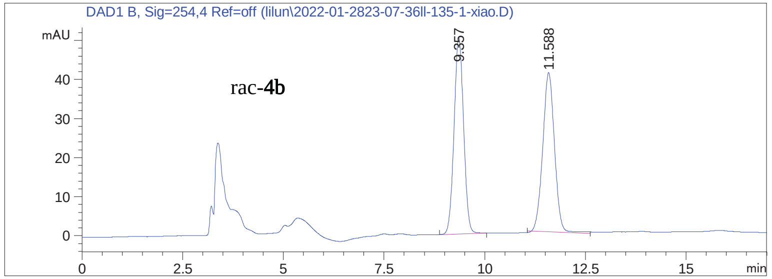

===

Area Percent Report

===

Sorted By : Signal
Multiplier : 1.0000
Dilution : 1.0000
Sample Amount: : 20.00000 [ng/ul] (not used in calc.)
Do not use Multiplier & Dilution Factor with ISTDs

Signal 1: DAD1 B, Sig=254,4 Ref=off

Peak #	RetTime [min]	Type	Width [min]	Area [mAU*s]	Height [mAU]	Area %
1	9.357	BB	0.2557	826.09973	50.26509	49.8063
2	11.588	MM	0.3399	832.52576	40.82389	50.1937

Totals : 1658.62549 91.08898

===

*** End of Report ***

Data File E:\LCDATA\Data\lilun\2022-01-2823-50-57ll-135-2-shou.D
Sample Name: ll-135-2-shou

===

Acq. Operator : 系统
Sample Operator : 系统
Acq. Instrument : LC1260 Location : 21
Injection Date : 1/28/2022 11:51:56 PM
Inj Volume : 20.000 µl
Acq. Method : E:\LCDATA\Methods\LI-LUN.M
Last changed : 1/28/2022 10:55:15 PM by 系统
(modified after loading)
Analysis Method : E:\LCDATA\Methods\LI-LUN.M
Last changed : 3/14/2022 4:36:33 PM by 系统
(modified after loading)
Sample Info : 98/1 ;OJ-H;20C

Additional Info : Peak(s) manually integrated

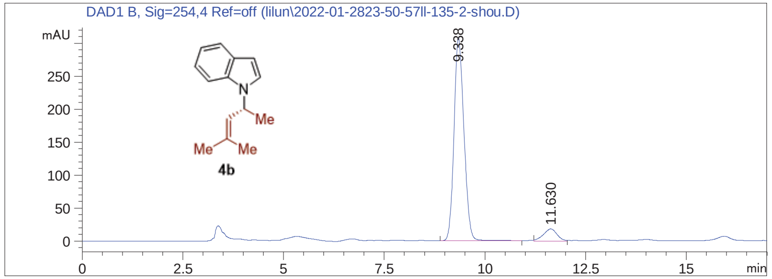

===

Area Percent Report

===

Sorted By : Signal
Multiplier : 1.0000
Dilution : 1.0000
Sample Amount: : 20.00000 [ng/ul] (not used in calc.)
Do not use Multiplier & Dilution Factor with ISTDs

Signal 1: DAD1 B, Sig=254,4 Ref=off

Peak #	RetTime [min]	Type	Width [min]	Area [mAU*s]	Height [mAU]	Area %
1	9.338	BB	0.2633	5266.34521	308.24094	92.0607
2	11.630	MM	0.4019	454.16724	18.83321	7.9393

Totals : 5720.51245 327.07415

===

*** End of Report ***

Supplementary Figure 217. HPLC spectra of compound **4b**

Data File E:\LCDATA\Data\lilun\2022-02-0421-36-36ll-52-x.D
Sample Name: ll-52-x

===
Acq. Operator : 系统
Sample Operator : 系统
Acq. Instrument : LC1260 Location : 21
Injection Date : 2/4/2022 9:37:34 PM
Inj Volume : 10.000 µl
Acq. Method : E:\LCDATA\Methods\LI-LUN.M
Last changed : 2/4/2022 9:34:46 PM by 系统
(modified after loading)
Analysis Method : E:\LCDATA\Methods\LI-LUN.M
Last changed : 3/14/2022 4:40:08 PM by 系统
(modified after loading)
Sample Info : 99/0.8 ;oj-H;20C

Additional Info : Peak(s) manually integrated

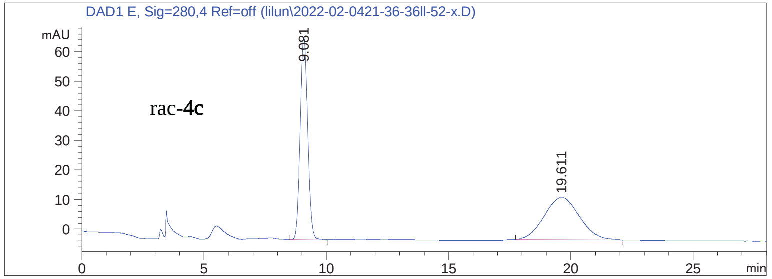

===
Area Percent Report
===

Sorted By : Signal
Multiplier : 1.0000
Dilution : 1.0000
Sample Amount: : 10.00000 [ng/ul] (not used in calc.)
Do not use Multiplier & Dilution Factor with ISTDs

Signal 1: DAD1 E, Sig=280,4 Ref=off

Peak #	RetTime [min]	Type	Width [min]	Area [mAU*s]	Height [mAU]	Area %
1	9.081	BB	0.3350	1462.09143	68.51716	50.4452
2	19.611	BB	1.2134	1436.28516	14.40262	49.5548
Totals :				2898.37659	82.91978	

===
*** End of Report ***

Data File E:\LCDATA\Data\lilun\2022-02-0422-30-09ll-52-s.D
Sample Name: ll-52-s

===
Acq. Operator : 系统
Sample Operator : 系统
Acq. Instrument : LC1260 Location : 21
Injection Date : 2/4/2022 10:31:01 PM
Inj Volume : 10.000 µl
Acq. Method : E:\LCDATA\Methods\LI-LUN.M
Last changed : 2/4/2022 9:34:46 PM by 系统
(modified after loading)
Analysis Method : E:\LCDATA\Methods\LI-LUN.M
Last changed : 3/14/2022 4:40:08 PM by 系统
(modified after loading)
Sample Info : 99/0.8 ;oj-H;20C

Additional Info : Peak(s) manually integrated

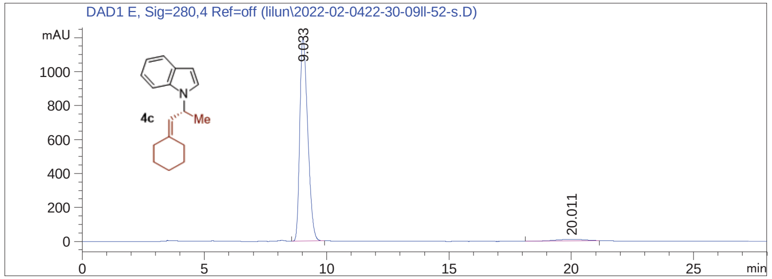

===
Area Percent Report
===

Sorted By : Signal
Multiplier : 1.0000
Dilution : 1.0000
Sample Amount: : 10.00000 [ng/ul] (not used in calc.)
Do not use Multiplier & Dilution Factor with ISTDs

Signal 1: DAD1 E, Sig=280,4 Ref=off

Peak #	RetTime [min]	Type	Width [min]	Area [mAU*s]	Height [mAU]	Area %
1	9.033	BB	0.3515	2.70889e4	1199.60669	97.3814
2	20.011	BB	0.9836	728.41205	8.91138	2.6186
Totals :				2.78173e4	1208.51807	

===
*** End of Report ***

Supplementary Figure 218. HPLC spectra of compound **4c**

Data File E:\LCDATA\Data\lilun\2022-02-0609-43-48ll-53-xiao.D
Sample Name: ll-53-xiao

===

```
Acq. Operator   : 系统
Sample Operator : 系统
Acq. Instrument : LC1260                          Location :   21
Injection Date  : 2/6/2022 9:44:45 AM
                                                Inj Volume : 20.000 µl
Acq. Method     : E:\LCDATA\Methods\LI-LUN.M
Last changed    : 2/6/2022 8:57:18 AM by 系统
                  (modified after loading)
Analysis Method : E:\LCDATA\Methods\LI-LUN.M
Last changed    : 3/14/2022 4:45:52 PM by 系统
                  (modified after loading)
Sample Info     : 98/0.8 ;od3-H;20C

Additional Info : Peak(s) manually integrated
```

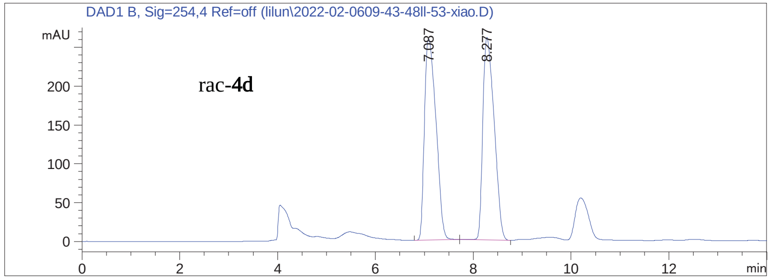

===

Area Percent Report

===

```
Sorted By             :      Signal
Multiplier            :      1.0000
Dilution              :      1.0000
Sample Amount:               :     20.00000  [ng/ul]   (not used in calc.)
Do not use Multiplier & Dilution Factor with ISTDs
```

Signal 1: DAD1 B, Sig=254,4 Ref=off

Peak #	RetTime [min]	Type	Width [min]	Area [mAU*s]	Height [mAU]	Area %
1	7.087	BB	0.2780	4222.65430	259.25104	50.0398
2	8.277	BB	0.2726	4215.94531	260.82770	49.9602

Totals : 8438.59961 520.07874

===

*** End of Report ***

Data File E:\LCDATA\Data\lilun\2022-02-0611-19-00ll-53-shou.D
Sample Name: ll-53-shou

===

```
Acq. Operator   : 系统
Sample Operator : 系统
Acq. Instrument : LC1260                          Location :   21
Injection Date  : 2/6/2022 11:20:02 AM
                                                Inj Volume : 20.000 µl
Acq. Method     : E:\LCDATA\Methods\LI-LUN.M
Last changed    : 2/6/2022 8:57:18 AM by 系统
                  (modified after loading)
Analysis Method : E:\LCDATA\Methods\LI-LUN.M
Last changed    : 3/14/2022 4:45:52 PM by 系统
                  (modified after loading)
Sample Info     : 98/0.8 ;od3-H;20C

Additional Info : Peak(s) manually integrated
```

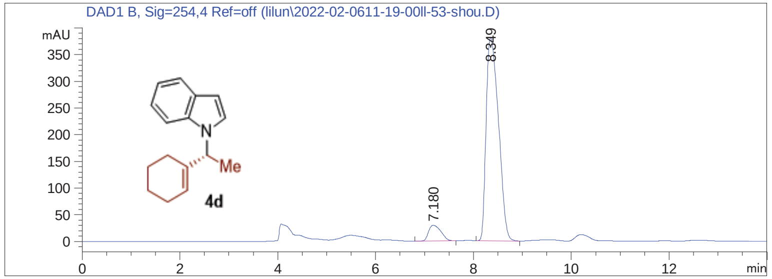

===

Area Percent Report

===

```
Sorted By             :      Signal
Multiplier            :      1.0000
Dilution              :      1.0000
Sample Amount:               :     20.00000  [ng/ul]   (not used in calc.)
Do not use Multiplier & Dilution Factor with ISTDs
```

Signal 1: DAD1 B, Sig=254,4 Ref=off

Peak #	RetTime [min]	Type	Width [min]	Area [mAU*s]	Height [mAU]	Area %
1	7.180	BB	0.2976	512.54205	29.36908	7.1546
2	8.349	BB	0.2958	6651.23145	380.73688	92.8454

Totals : 7163.77350 410.10596

===

*** End of Report ***

Supplementary Figure 219. HPLC spectra of compound **4d**

Data File E:\LCDATA\Data\lilun\2022-01-3011-16-33ll-1kiti-51-xiao.D
Sample Name: ll-1kiti-51-xiao

===

Acq. Operator : 系统
Sample Operator : 系统
Acq. Instrument : LC1260 Location : 21
Injection Date : 1/30/2022 11:17:31 AM
Inj Volume : 10.000 µl
Acq. Method : E:\LCDATA\Methods\LI-LUN.M
Last changed : 1/30/2022 11:40:48 AM by 系统
(modified after loading)
Analysis Method : E:\LCDATA\Methods\LI-LUN.M
Last changed : 3/14/2022 4:47:55 PM by 系统
(modified after loading)
Sample Info : 98/1 ;AD-H;20C

Additional Info : Peak(s) manually integrated

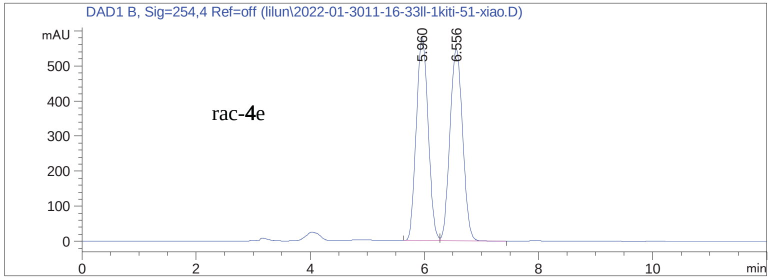

===

Area Percent Report

===

Sorted By : Signal
Multiplier : 1.0000
Dilution : 1.0000
Sample Amount: : 10.00000 [ng/ul] (not used in calc.)
Do not use Multiplier & Dilution Factor with ISTDs

Signal 1: DAD1 B, Sig=254,4 Ref=off

Peak #	RetTime [min]	Type	Width [min]	Area [mAU*s]	Height [mAU]	Area %
1	5.960	BV	0.2290	8384.58008	578.95612	49.7664
2	6.556	VB	0.2443	8463.30469	547.66638	50.2336

Totals : 1.68479e4 1126.62250

===

*** End of Report ***

Data File E:\LCDATA\Data\lilun\2022-01-3011-59-38ll-1kiti-51-xiao.D
Sample Name: ll-1kiti-51-xiao

===

Acq. Operator : 系统
Sample Operator : 系统
Acq. Instrument : LC1260 Location : 21
Injection Date : 1/30/2022 12:00:31 PM
Inj Volume : 10.000 µl
Acq. Method : E:\LCDATA\Methods\LI-LUN.M
Last changed : 1/30/2022 11:41:34 AM by 系统
(modified after loading)
Analysis Method : E:\LCDATA\Methods\LI-LUN.M
Last changed : 3/14/2022 4:47:55 PM by 系统
(modified after loading)
Sample Info : 98/1 ;AD-H;20C

Additional Info : Peak(s) manually integrated

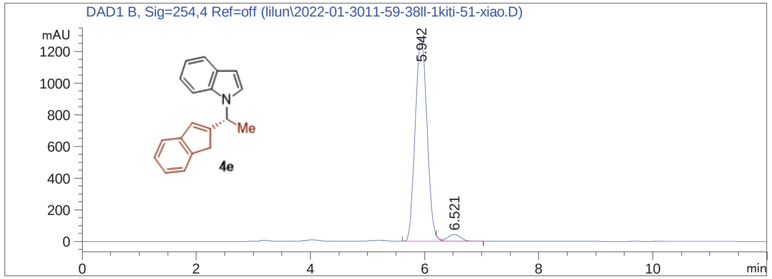

===

Area Percent Report

===

Sorted By : Signal
Multiplier : 1.0000
Dilution : 1.0000
Sample Amount: : 10.00000 [ng/ul] (not used in calc.)
Do not use Multiplier & Dilution Factor with ISTDs

Signal 1: DAD1 B, Sig=254,4 Ref=off

Peak #	RetTime [min]	Type	Width [min]	Area [mAU*s]	Height [mAU]	Area %
1	5.942	BV R	0.2278	1.85074e4	1286.98706	96.5163
2	6.521	VB E	0.2426	668.02069	42.67845	3.4837

Totals : 1.91754e4 1329.66551

===

*** End of Report ***

Supplementary Figure 220. HPLC spectra of compound **4c**

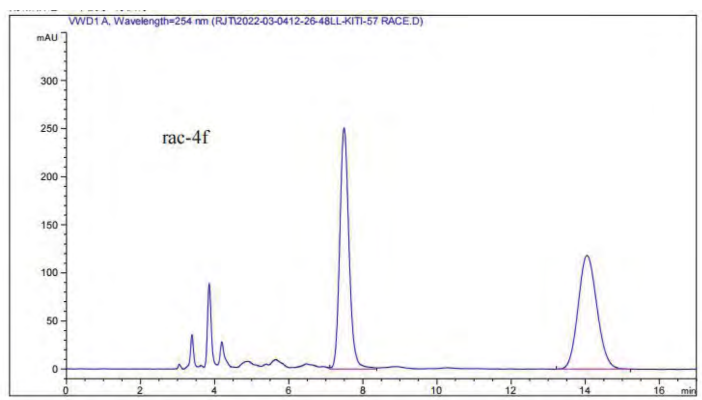

信号 1: VWD1 A, Wavelength=254 nm

峰 #	保留时间 [min]	类型	峰宽 [min]	峰面积 [mAU*s]	峰面积 %	名称
1	7.492	VV	0.2667	4337.73438	50.9569	?
2	14.046	BB	0.5519	4174.82080	49.0431	?

总量 : 8512.55518

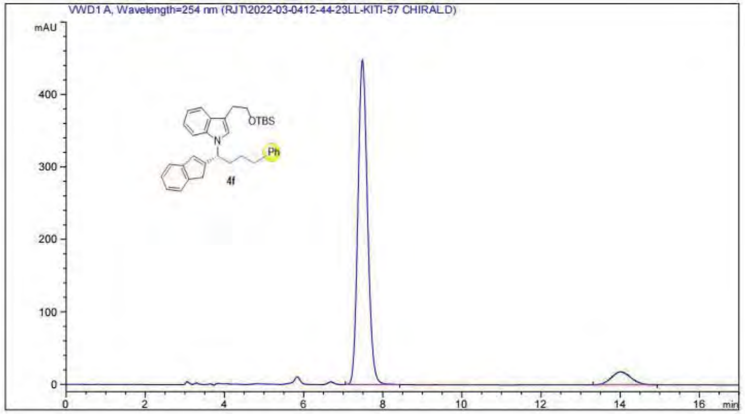

信号 1: VWD1 A, Wavelength=254 nm

峰 #	保留时间 [min]	类型	峰宽 [min]	峰面积 [mAU*s]	峰面积 %	名称
1	7.487	BB	0.2612	7532.28711	92.2718	?
2	14.010	BB	0.5448	630.86047	7.7282	?

总量 : 8163.14758

Supplementary Figure 221. HPLC spectra of compound **4f**

Data File E:\DATA\RJT\2022-02-1916-46-39YD.D
Sample Name: YD

===
Acq. Operator : 系统
Sample Operator : 系统
Acq. Instrument : LC1 Location : -
Injection Date : 2022/2/19 16:46:39
Inj Volume :
Acq. Method : C:\Chem32\1\Methods\ybm.M
Last changed : 2022/2/19 15:32:07 : 系统
(modified after loading)
Analysis Method : C:\CHEM32\1\METHODS\def_LC.M
Last changed : 2022/4/1 19:30:55 : 系统
(modified after loading)
Sample Info : IB-3, hex/ipr=100:0, 254 nm,1.0 ml/min,66.14 bar

Additional Info : Peak(s) manually integrated

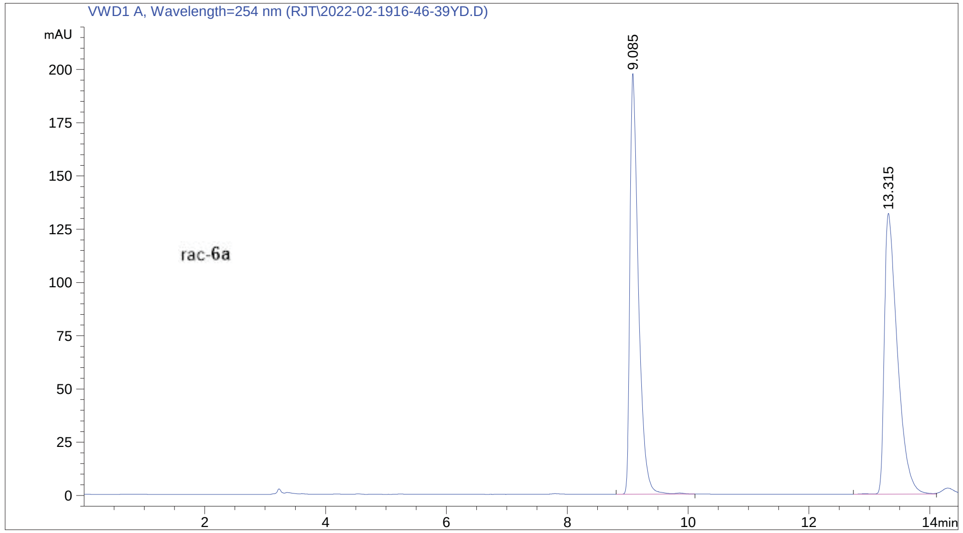

===
Area Percent Report
===

Sorted By : Signal
Multiplier : 1.0000
Dilution : 1.0000
Sample Amount: : 1.00000 [ng/ul] (not used in calc.)
Do not use Multiplier & Dilution Factor with ISTDs

Signal 1: VWD1 A, Wavelength=254 nm

Peak #	RetTime [min]	Type	Width [min]	Area [mAU*s]	Height [mAU]	Area %
1	9.085	BV R	0.1465	1938.27173	197.43634	49.5428
2	13.315	MF	0.2495	1974.04834	131.85512	50.4572

LC1 2022/4/1 19:30:59 系统

Data File E:\DATA\RJT\2022-01-2921-14-07YD CHIRAL.D
Sample Name: YD CHIRAL

===
Acq. Operator : 系统
Sample Operator : 系统
Acq. Instrument : LC1 Location : -
Injection Date : 2022/1/29 21:14:07
Inj Volume :
Acq. Method : C:\Chem32\1\Methods\ybm.M
Last changed : 2022/1/29 20:03:38 : 系统
(modified after loading)
Analysis Method : C:\CHEM32\1\METHODS\def_LC.M
Last changed : 2022/4/1 19:27:09 : 系统
(modified after loading)
Sample Info : IB-3, hex/ipr=100/0, 254 nm,1.0 ml/min, 68.29 bar

Additional Info : Peak(s) manually integrated

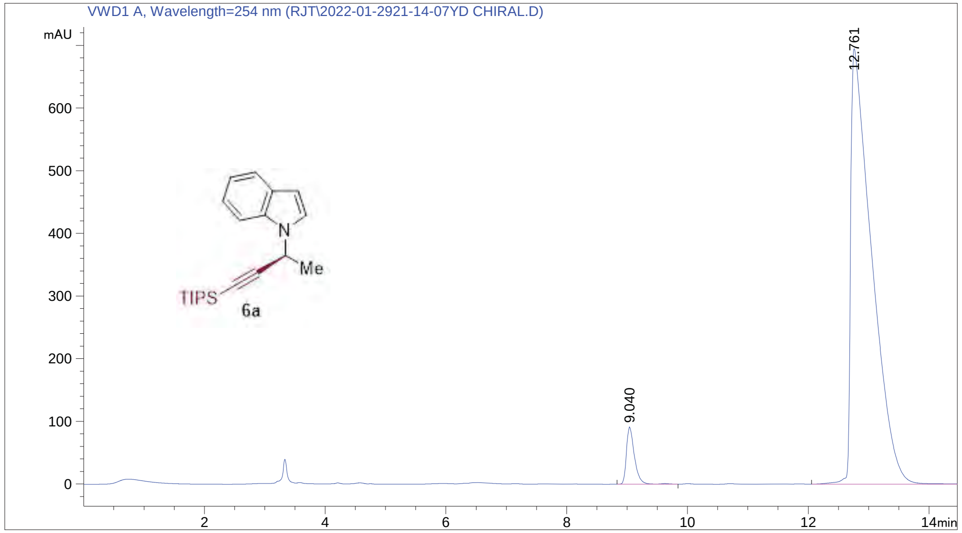

===
Area Percent Report
===

Sorted By : Signal
Multiplier : 1.0000
Dilution : 1.0000
Sample Amount: : 1.00000 [ng/ul] (not used in calc.)
Do not use Multiplier & Dilution Factor with ISTDs

Signal 1: VWD1 A, Wavelength=254 nm

Peak #	RetTime [min]	Type	Width [min]	Area [mAU*s]	Height [mAU]	Area %
1	9.040	BV R	0.1385	840.87646	91.24879	4.7720
2	12.761	BBA	0.3447	1.67802e4	694.95398	95.2280

Page 1/1

Supplementary Figure 222. HPLC spectra of compound **6a**

Data File E:\DATA\RJT\2022-02-1514-22-354-Me.D
Sample Name: 4-Me

===

Acq. Operator : 系统
Sample Operator : 系统
Acq. Instrument : LC1 Location : -
Injection Date : 2022/2/15 14:22:36
Inj Volume :
Acq. Method : C:\Chem32\1\Methods\ybm.M
Last changed : 2022/2/15 13:24:36 : 系统
(modified after loading)
Analysis Method : C:\Chem32\1\Methods\def_LC.M
Last changed : 2022/4/2 10:57:34 : 系统
(modified after loading)
Sample Info : IB-3, hex/ipr=100:0, 254 nm, 1.0 ml/min, 68.24 bar

Additional Info : Peak(s) manually integrated

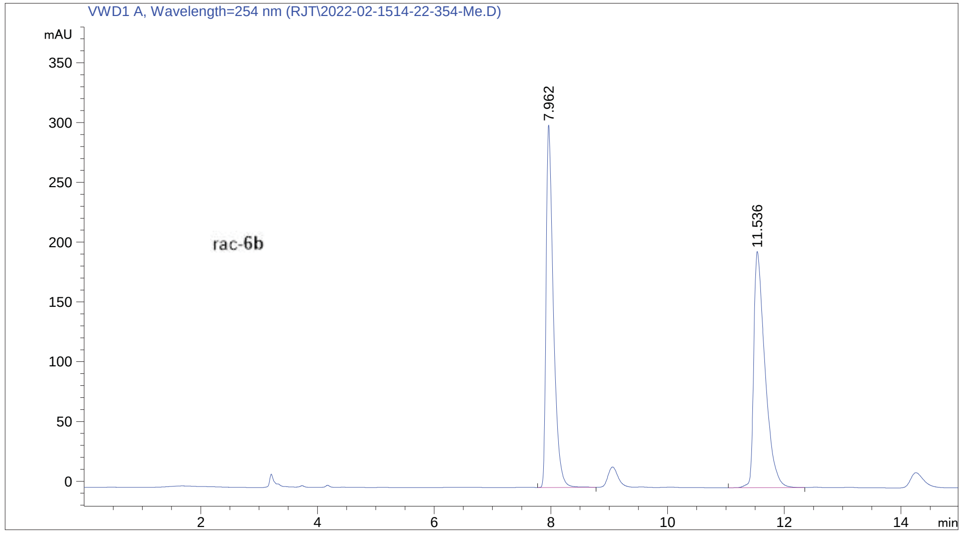

===
Area Percent Report
===

Sorted By : Signal
Multiplier : 1.0000
Dilution : 1.0000
Sample Amount: : 1.00000 [ng/ul] (not used in calc.)
Use Multiplier & Dilution Factor with ISTDs

Signal 1: VWD1 A, Wavelength=254 nm

Peak #	RetTime [min]	Type	Width [min]	Area [mAU*s]	Height [mAU]	Area %
1	7.962	BB	0.1291	2626.62231	303.06442	49.7092
2	11.536	BB	0.1990	2657.34985	197.42696	50.2908

LC1 2022/4/2 10:57:42 系统

Data File E:\DATA\RJT\2022-02-1514-07-144-Me.D
Sample Name: 4-Me

===

Acq. Operator : 系统
Sample Operator : 系统
Acq. Instrument : LC1 Location : -
Injection Date : 2022/2/15 14:07:14
Inj Volume :
Acq. Method : C:\Chem32\1\Methods\ybm.M
Last changed : 2022/2/15 13:24:36 : 系统
(modified after loading)
Analysis Method : C:\Chem32\1\Methods\def_LC.M
Last changed : 2022/4/2 10:56:23 : 系统
(modified after loading)
Sample Info : IB-3, hex/ipr=100:0, 254 nm, 1.0 ml/min, 68.24 bar

Additional Info : Peak(s) manually integrated

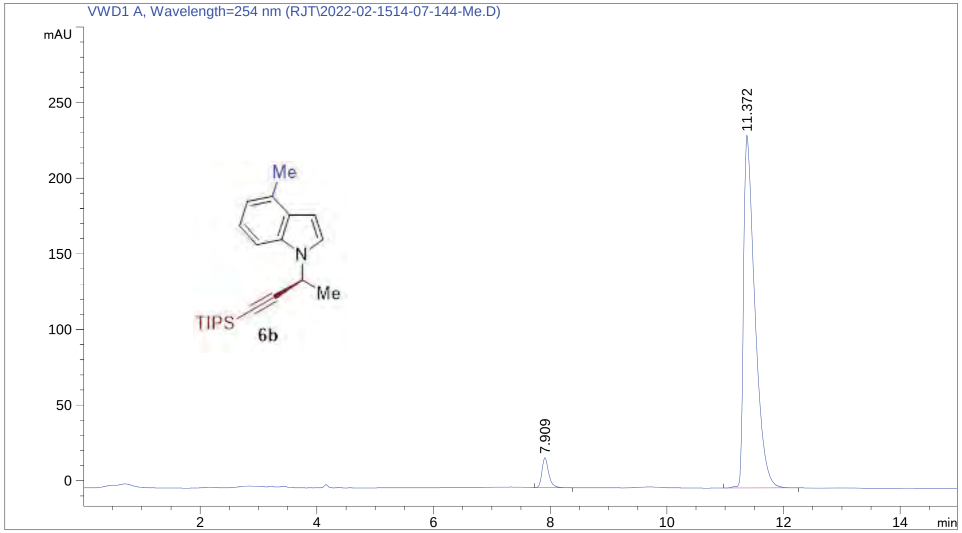

===
Area Percent Report
===

Sorted By : Signal
Multiplier : 1.0000
Dilution : 1.0000
Sample Amount: : 1.00000 [ng/ul] (not used in calc.)
Use Multiplier & Dilution Factor with ISTDs

Signal 1: VWD1 A, Wavelength=254 nm

Peak #	RetTime [min]	Type	Width [min]	Area [mAU*s]	Height [mAU]	Area %
1	7.909	MM	0.1330	156.99902	19.67753	4.7339
2	11.372	BB	0.2021	3159.50977	233.08203	95.2661

Page 1/1

Supplementary Figure 223. HPLC spectra of compound **6b**

Data File E:\DATA\RJT\2022-01-1716-46-485Cl-YD RACE.D
Sample Name: 5Cl-YD RACE

===

Acq. Operator : 系统
Sample Operator : 系统
Acq. Instrument : LC1 Location : -
Injection Date : 2022/1/17 16:46:48
Inj Volume :
Acq. Method : C:\Chem32\1\Methods\ybm.M
Last changed : 2022/1/17 15:18:26 : 系统
(modified after loading)
Analysis Method : C:\Chem32\1\Methods\def_LC.M
Last changed : 2022/4/2 11:00:38 : 系统
(modified after loading)
Sample Info : IB-3, hex/ipr=100/0, 254 nm, 1.0 ml/min, 68.60 bar

Additional Info : Peak(s) manually integrated

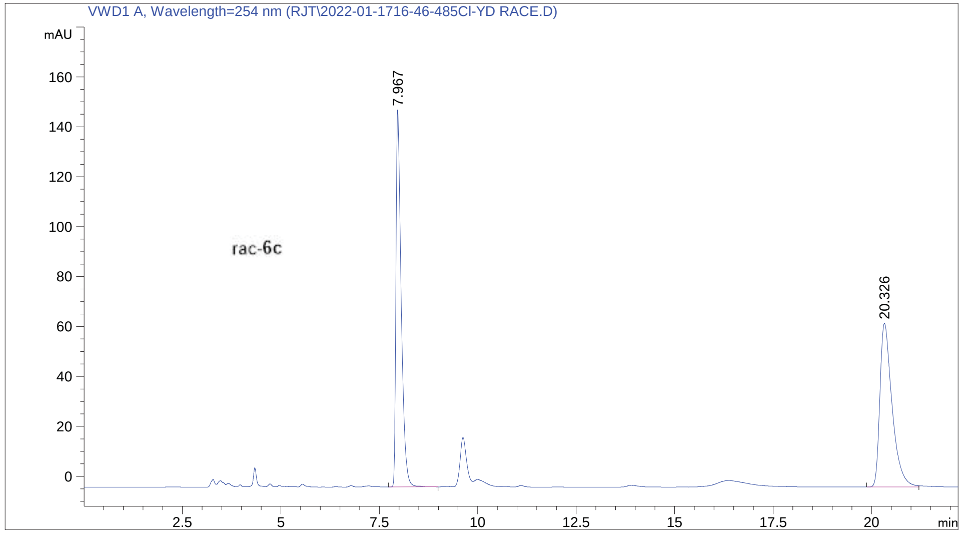

===
Area Percent Report
===

Sorted By : Signal
Multiplier : 1.0000
Dilution : 1.0000
Sample Amount: : 1.00000 [ng/ul] (not used in calc.)
Use Multiplier & Dilution Factor with ISTDs

Signal 1: VWD1 A, Wavelength=254 nm

Peak #	RetTime [min]	Type	Width [min]	Area [mAU*s]	Height [mAU]	Area %
1	7.967	BB	0.1380	1398.31897	151.09947	49.8277
2	20.326	MF	0.3578	1407.98853	65.57978	50.1723

Data File E:\DATA\RJT\2022-01-1717-10-175Cl-YD CHIRAL.D
Sample Name: 5Cl-YD CHIRAL

===

Acq. Operator : 系统
Sample Operator : 系统
Acq. Instrument : LC1 Location : -
Injection Date : 2022/1/17 17:10:17
Inj Volume :
Acq. Method : C:\Chem32\1\Methods\ybm.M
Last changed : 2022/1/17 15:18:26 : 系统
(modified after loading)
Analysis Method : C:\Chem32\1\Methods\def_LC.M
Last changed : 2022/4/2 11:00:03 : 系统
(modified after loading)
Sample Info : IB-3, hex/ipr=100/0, 254 nm, 1.0 ml/min, 68.60 bar

Additional Info : Peak(s) manually integrated

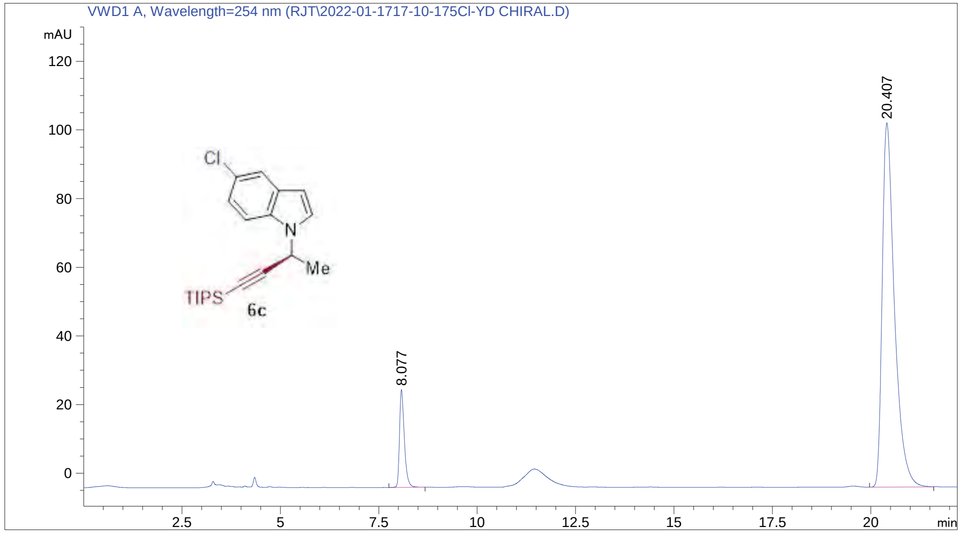

===
Area Percent Report
===

Sorted By : Signal
Multiplier : 1.0000
Dilution : 1.0000
Sample Amount: : 1.00000 [ng/ul] (not used in calc.)
Use Multiplier & Dilution Factor with ISTDs

Signal 1: VWD1 A, Wavelength=254 nm

Peak #	RetTime [min]	Type	Width [min]	Area [mAU*s]	Height [mAU]	Area %
1	8.077	BB	0.1288	243.52100	28.46641	9.5762
2	20.407	MF	0.3611	2299.44751	106.12550	90.4238

Supplementary Figure 224. HPLC spectra of compound **6c**

Data File E:\DATA\RJT\2022-01-1402-30-325F-YD RACE.D
Sample Name: 5F-YD RACE

===
Acq. Operator : 系统
Sample Operator : 系统
Acq. Instrument : LC1 Location : -
Injection Date : 2022/1/14 2:30:32
Inj Volume :
Acq. Method : C:\Chem32\1\Methods\ybm.M
Last changed : 2022/1/14 2:24:07 : 系统
(modified after loading)
Analysis Method : C:\Chem32\1\Methods\def_LC.M
Last changed : 2022/4/2 11:07:43 : 系统
(modified after loading)
Sample Info : IB-3, hex/ipr=100/0, 254 nm, 1.0 ml/min, 68.38 bar

Additional Info : Peak(s) manually integrated

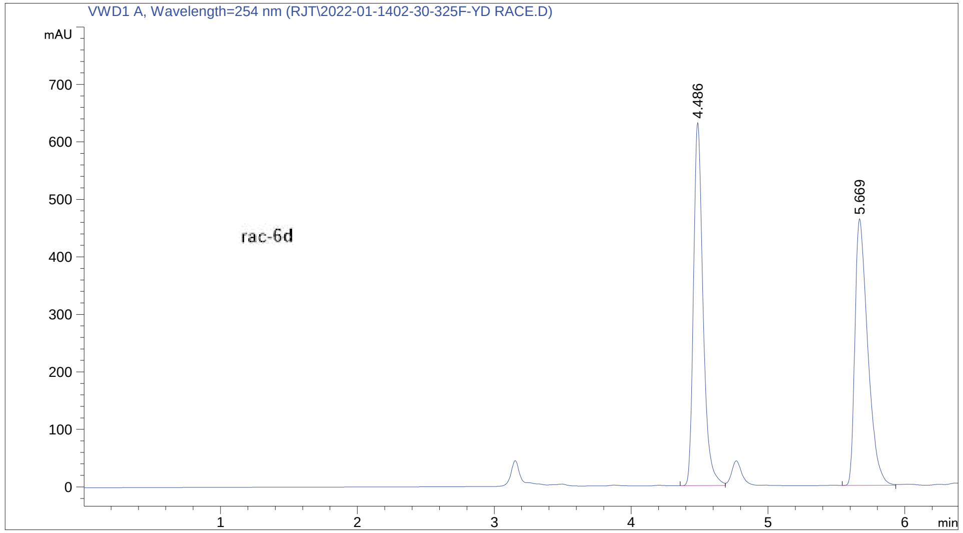

===
Area Percent Report
===

Sorted By : Signal
Multiplier : 1.0000
Dilution : 1.0000
Sample Amount: : 1.00000 [ng/ul] (not used in calc.)
Use Multiplier & Dilution Factor with ISTDs

Signal 1: VWD1 A, Wavelength=254 nm

Peak #	RetTime [min]	Type	Width [min]	Area [mAU*s]	Height [mAU]	Area %
1	4.486	MF	0.0808	3059.93970	631.48419	50.2241
2	5.669	MF	0.1090	3032.63208	463.88596	49.7759

Data File E:\DATA\RJT\2022-01-1403-36-185F-YD RACE.D
Sample Name: 5F-YD RACE

===
Acq. Operator : 系统
Sample Operator : 系统
Acq. Instrument : LC1 Location : -
Injection Date : 2022/1/14 3:36:18
Inj Volume :
Acq. Method : C:\Chem32\1\Methods\ybm.M
Last changed : 2022/1/14 2:24:07 : 系统
(modified after loading)
Analysis Method : C:\Chem32\1\Methods\def_LC.M
Last changed : 2022/4/2 11:07:12 : 系统
(modified after loading)
Sample Info : IB-3, hex/ipr=100/0, 254 nm, 1.0 ml/min, 68.38 bar

Additional Info : Peak(s) manually integrated

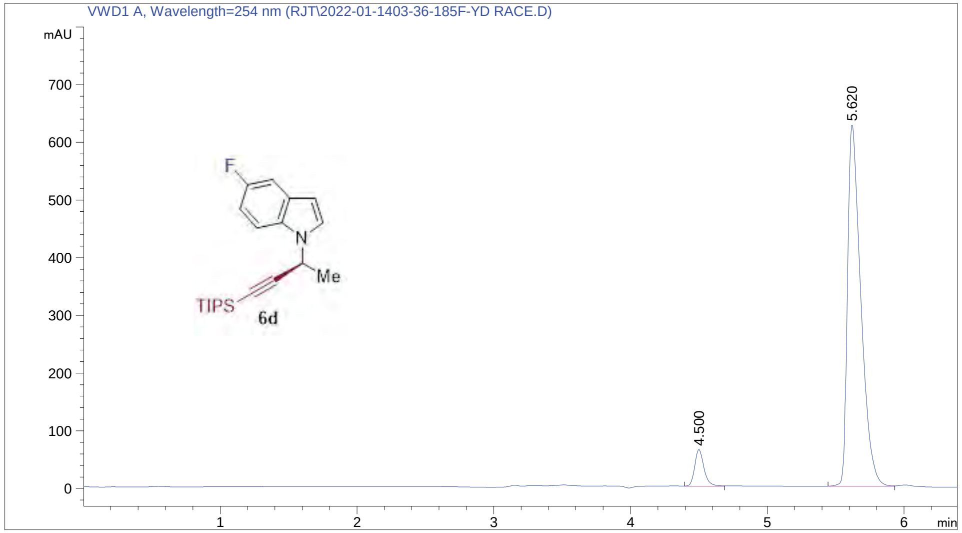

===
Area Percent Report
===

Sorted By : Signal
Multiplier : 1.0000
Dilution : 1.0000
Sample Amount: : 1.00000 [ng/ul] (not used in calc.)
Use Multiplier & Dilution Factor with ISTDs

Signal 1: VWD1 A, Wavelength=254 nm

Peak #	RetTime [min]	Type	Width [min]	Area [mAU*s]	Height [mAU]	Area %
1	4.500	MM	0.0789	300.38794	63.43184	6.6239
2	5.620	MM	0.1127	4234.49609	626.15586	93.3761

Supplementary Figure 225. HPLC spectra of compound **6d**

Data File E:\DATA\RJT\2022-02-1514-45-495-Me.D
Sample Name: 5-Me

===

Acq. Operator : 系统
Sample Operator : 系统
Acq. Instrument : LC1 Location : -
Injection Date : 2022/2/15 14:45:49
Inj Volume :
Acq. Method : C:\Chem32\1\Methods\ybm.M
Last changed : 2022/2/15 14:44:43 : 系统
(modified after loading)
Analysis Method : C:\Chem32\1\Methods\def_LC.M
Last changed : 2022/4/2 11:10:05 : 系统
(modified after loading)
Sample Info : IB-3, hex/ipr=100:0, 254 nm, 1.0 ml/min, 66.45 bar

Additional Info : Peak(s) manually integrated

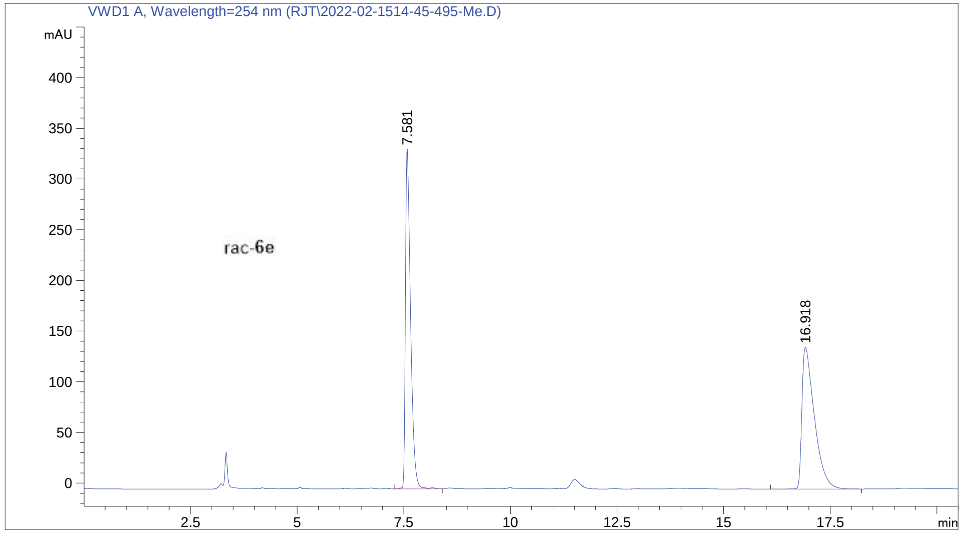

===
Area Percent Report
===

Sorted By : Signal
Multiplier : 1.0000
Dilution : 1.0000
Sample Amount: : 1.00000 [ng/ul] (not used in calc.)
Use Multiplier & Dilution Factor with ISTDs

Signal 1: VWD1 A, Wavelength=254 nm

Peak #	RetTime [min]	Type	Width [min]	Area [mAU*s]	Height [mAU]	Area %
1	7.581	BV R	0.1260	2815.90479	335.23083	49.8333
2	16.918	BB	0.2992	2834.74390	139.99883	50.1667

Data File E:\DATA\RJT\2022-02-1515-06-515-Me.D
Sample Name: 5-Me

===

Acq. Operator : 系统
Sample Operator : 系统
Acq. Instrument : LC1 Location : -
Injection Date : 2022/2/15 15:06:51
Inj Volume :
Acq. Method : C:\Chem32\1\Methods\ybm.M
Last changed : 2022/2/15 14:44:43 : 系统
(modified after loading)
Analysis Method : C:\Chem32\1\Methods\def_LC.M
Last changed : 2022/4/2 11:10:37 : 系统
(modified after loading)
Sample Info : IB-3, hex/ipr=100:0, 254 nm, 1.0 ml/min, 66.45 bar

Additional Info : Peak(s) manually integrated

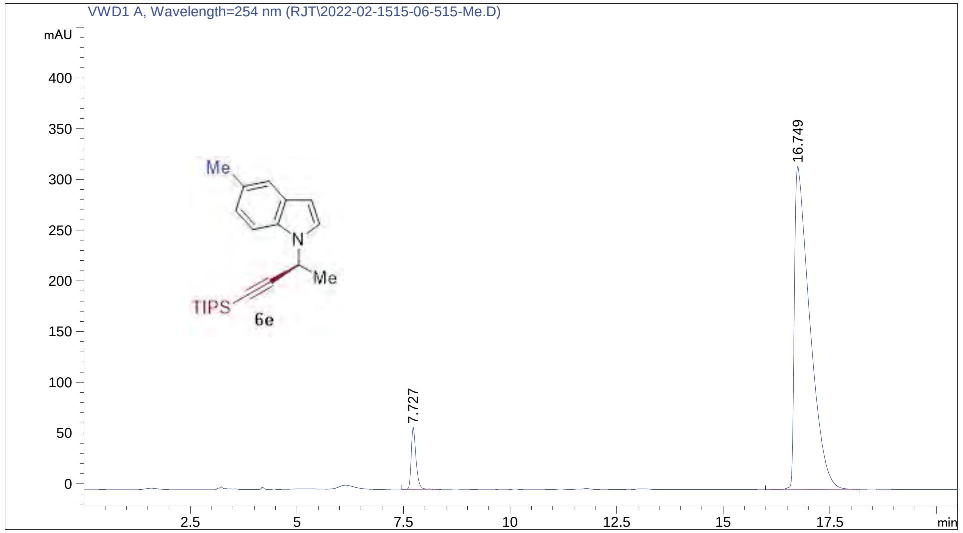

===
Area Percent Report
===

Sorted By : Signal
Multiplier : 1.0000
Dilution : 1.0000
Sample Amount: : 1.00000 [ng/ul] (not used in calc.)
Use Multiplier & Dilution Factor with ISTDs

Signal 1: VWD1 A, Wavelength=254 nm

Peak #	RetTime [min]	Type	Width [min]	Area [mAU*s]	Height [mAU]	Area %
1	7.727	FM	0.1318	482.88156	61.08179	5.6909
2	16.749	BB	0.3674	8002.29541	318.16971	94.3091

Supplementary Figure 226. HPLC spectra of compound **6e**

Data File E:\DATA\RJT\2022-02-1419-41-365-OMe.D
Sample Name: 5-OMe

===

Acq. Operator : 系统
Sample Operator : 系统
Acq. Instrument : LC1 Location : -
Injection Date : 2022/2/14 19:41:36
Inj Volume :
Acq. Method : C:\Chem32\1\Methods\ybm.M
Last changed : 2022/2/14 19:33:21 : 系统
(modified after loading)
Analysis Method : C:\Chem32\1\Methods\def_LC.M
Last changed : 2022/4/2 11:20:10 : 系统
(modified after loading)
Sample Info : IB-3, hex/ipr=99:1, 254 nm,1.0 ml/min,67.42 bar

Additional Info : Peak(s) manually integrated

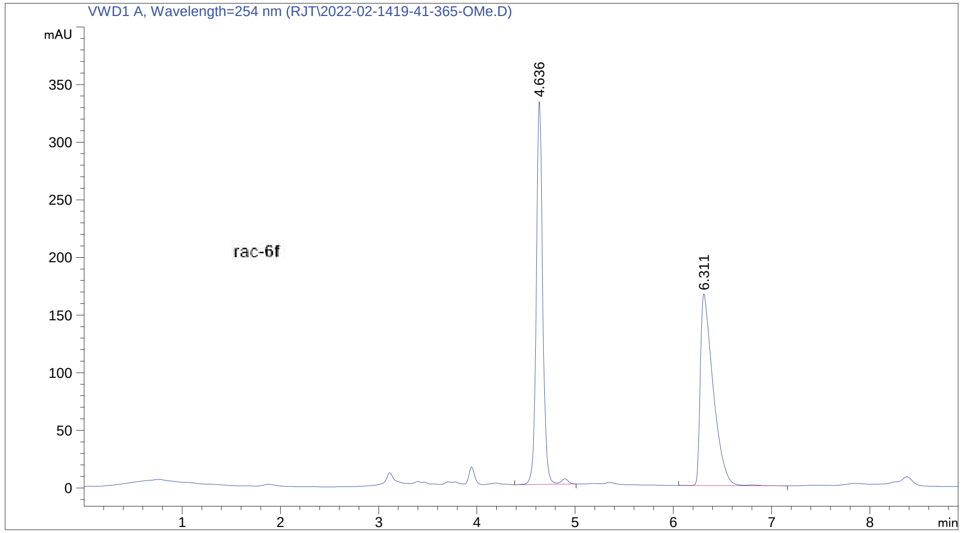

===
Area Percent Report
===

Sorted By : Signal
Multiplier : 1.0000
Dilution : 1.0000
Sample Amount: : 1.00000 [ng/ul] (not used in calc.)
Use Multiplier & Dilution Factor with ISTDs

Signal 1: VWD1 A, Wavelength=254 nm

Peak #	RetTime [min]	Type	Width [min]	Area [mAU*s]	Height [mAU]	Area %
1	4.636	BV R	0.0685	1520.12048	332.47052	50.0740
2	6.311	BV R	0.1344	1515.62476	166.24831	49.9260

LC1 2022/4/2 11:20:18 系统

Data File E:\DATA\RJT\2022-02-1419-16-035-OMe.D
Sample Name: 5-OMe

===

Acq. Operator : 系统
Sample Operator : 系统
Acq. Instrument : LC1 Location : -
Injection Date : 2022/2/14 19:16:03
Inj Volume :
Acq. Method : C:\Chem32\1\Methods\ybm.M
Last changed : 2022/2/14 17:41:01 : 系统
(modified after loading)
Analysis Method : C:\Chem32\1\Methods\def_LC.M
Last changed : 2022/4/2 11:19:35 : 系统
(modified after loading)
Sample Info : IB-3, hex/ipr=99:1, 254 nm,1.0 ml/min,67.42 bar

Additional Info : Peak(s) manually integrated

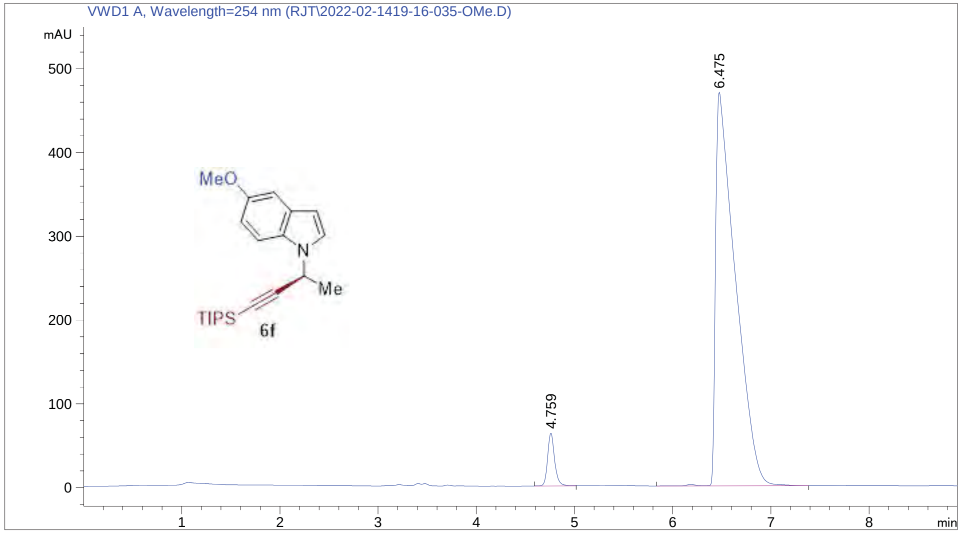

===
Area Percent Report
===

Sorted By : Signal
Multiplier : 1.0000
Dilution : 1.0000
Sample Amount: : 1.00000 [ng/ul] (not used in calc.)
Use Multiplier & Dilution Factor with ISTDs

Signal 1: VWD1 A, Wavelength=254 nm

Peak #	RetTime [min]	Type	Width [min]	Area [mAU*s]	Height [mAU]	Area %
1	4.759	MM	0.0830	314.37442	63.14611	4.5635
2	6.475	VB R	0.1940	6574.44092	469.70044	95.4365

Page 1/1

Supplementary Figure 227. HPLC spectra of compound **6f**

Data File E:\DATA\RJT\2022-02-0923-12-195Bpin YD.D
Sample Name: 5Bpin YD

===

Acq. Operator : 系统
Sample Operator : 系统
Acq. Instrument : LC1 Location : -
Injection Date : 2022/2/9 23:12:19
Inj Volume :
Acq. Method : C:\Chem32\1\Methods\ybm.M
Last changed : 2022/2/9 22:33:09 : 系统
(modified after loading)
Analysis Method : C:\Chem32\1\Methods\def_LC.M
Last changed : 2022/4/2 11:22:44 : 系统
(modified after loading)
Sample Info : IB-3, hex/ipr=99:1, 254 nm, 1.0 ml/min, 57.05 bar

Additional Info : Peak(s) manually integrated

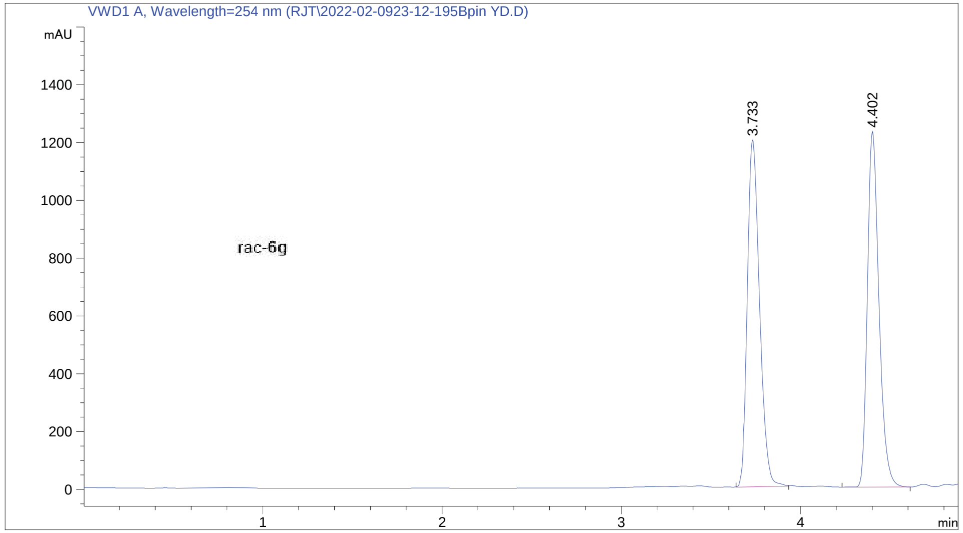

===
Area Percent Report
===

Sorted By : Signal
Multiplier : 1.0000
Dilution : 1.0000
Sample Amount: : 1.00000 [ng/ul] (not used in calc.)
Use Multiplier & Dilution Factor with ISTDs

Signal 1: VWD1 A, Wavelength=254 nm

Peak #	RetTime [min]	Type	Width [min]	Area [mAU*s]	Height [mAU]	Area %
1	3.733	MM	0.0788	5678.55078	1200.32898	50.7489
2	4.402	VB R	0.0583	5510.95117	1229.97412	49.2511

LC1 2022/4/2 11:22:47 系统

Data File E:\DATA\RJT\2022-02-0923-00-235Bpin YD.D
Sample Name: 5Bpin YD

===

Acq. Operator : 系统
Sample Operator : 系统
Acq. Instrument : LC1 Location : -
Injection Date : 2022/2/9 23:00:23
Inj Volume :
Acq. Method : C:\Chem32\1\Methods\ybm.M
Last changed : 2022/2/9 22:33:09 : 系统
(modified after loading)
Analysis Method : C:\Chem32\1\Methods\def_LC.M
Last changed : 2022/4/2 11:23:59 : 系统
(modified after loading)
Sample Info : IB-3, hex/ipr=99:1, 254 nm, 1.0 ml/min, 57.05 bar

Additional Info : Peak(s) manually integrated

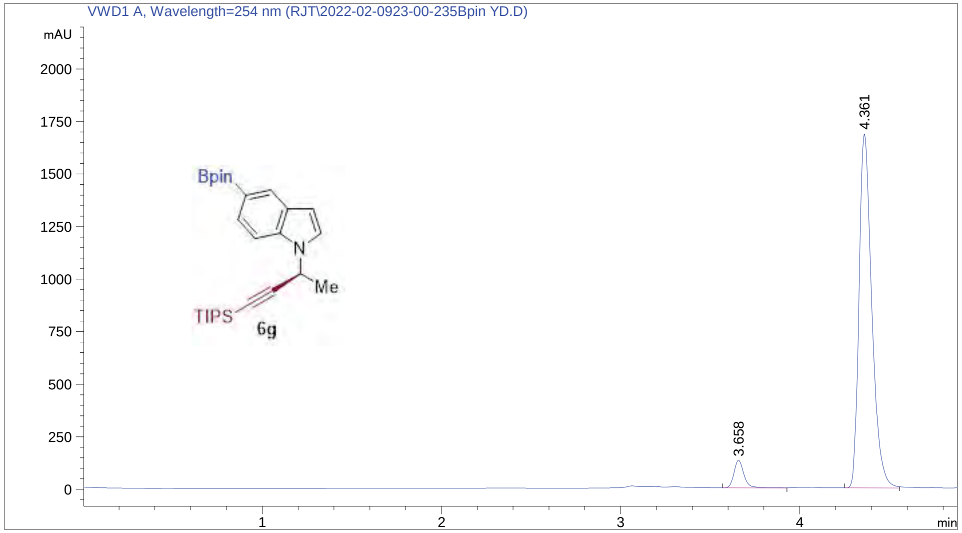

===
Area Percent Report
===

Sorted By : Signal
Multiplier : 1.0000
Dilution : 1.0000
Sample Amount: : 1.00000 [ng/ul] (not used in calc.)
Use Multiplier & Dilution Factor with ISTDs

Signal 1: VWD1 A, Wavelength=254 nm

Peak #	RetTime [min]	Type	Width [min]	Area [mAU*s]	Height [mAU]	Area %
1	3.658	MM	0.0555	518.34454	131.55570	5.8659
2	4.361	MF	0.0822	8318.21582	1685.04407	94.1341

Page 1/1

Supplementary Figure 228. HPLC spectra of compound **6g**

Data File E:\DATA\RJT\2022-01-3015-25-496F-YD.D
Sample Name: 6F-YD

===

Acq. Operator : 系统
Sample Operator : 系统
Acq. Instrument : LC1 Location : -
Injection Date : 2022/1/30 15:25:49
Inj Volume :
Acq. Method : C:\Chem32\1\Methods\ybm.M
Last changed : 2022/1/30 15:02:24 : 系统
(modified after loading)
Analysis Method : C:\Chem32\1\Methods\def_LC.M
Last changed : 2022/4/2 11:25:41 : 系统
(modified after loading)
Sample Info : IB-3, hex/ipr=100/0, 254 nm,1.0 ml/min, 69.75 bar

Additional Info : Peak(s) manually integrated

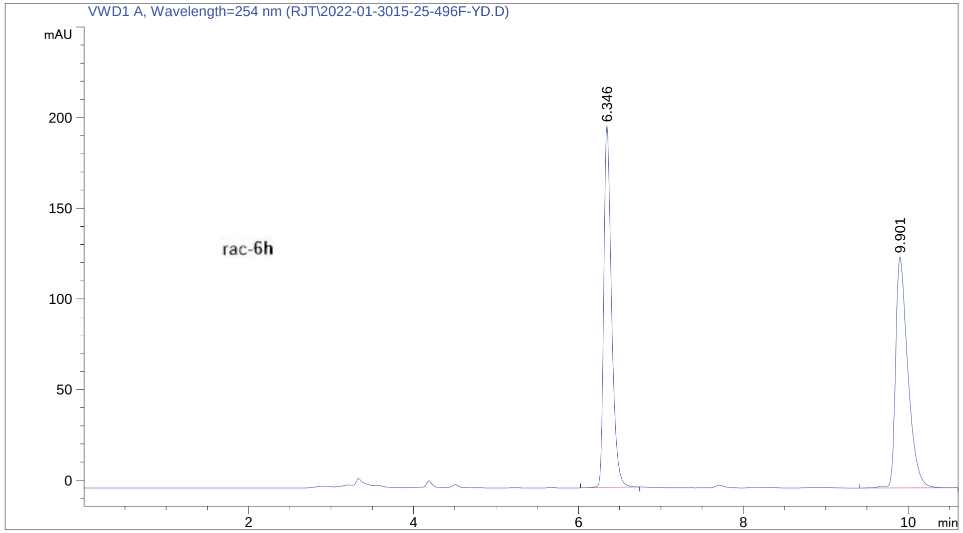

===
Area Percent Report
===

Sorted By : Signal
Multiplier : 1.0000
Dilution : 1.0000
Sample Amount: : 1.00000 [ng/ul] (not used in calc.)
Use Multiplier & Dilution Factor with ISTDs

Signal 1: VWD1 A, Wavelength=254 nm

Peak #	RetTime [min]	Type	Width [min]	Area [mAU*s]	Height [mAU]	Area %
1	6.346	BB	0.0996	1309.42371	199.52733	49.8846
2	9.901	VBAR	0.1545	1315.48022	127.35950	50.1154

LC1 2022/4/2 11:25:43 系统

Data File E:\DATA\RJT\2022-01-3015-36-566F-YD.D
Sample Name: 6F-YD

===

Acq. Operator : 系统
Sample Operator : 系统
Acq. Instrument : LC1 Location : -
Injection Date : 2022/1/30 15:36:56
Inj Volume :
Acq. Method : C:\Chem32\1\Methods\ybm.M
Last changed : 2022/1/30 15:02:24 : 系统
(modified after loading)
Analysis Method : C:\Chem32\1\Methods\def_LC.M
Last changed : 2022/4/2 11:26:17 : 系统
(modified after loading)
Sample Info : IB-3, hex/ipr=100/0, 254 nm,1.0 ml/min, 69.75 bar

Additional Info : Peak(s) manually integrated

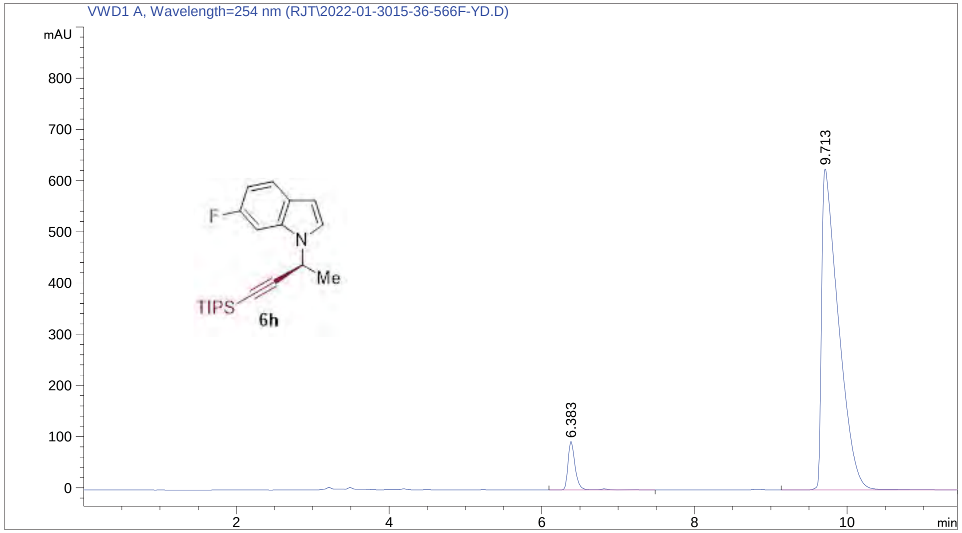

===
Area Percent Report
===

Sorted By : Signal
Multiplier : 1.0000
Dilution : 1.0000
Sample Amount: : 1.00000 [ng/ul] (not used in calc.)
Use Multiplier & Dilution Factor with ISTDs

Signal 1: VWD1 A, Wavelength=254 nm

Peak #	RetTime [min]	Type	Width [min]	Area [mAU*s]	Height [mAU]	Area %
1	6.383	BV R	0.0975	632.79803	94.57857	6.1541
2	9.713	VV R	0.2226	9649.67676	626.77777	93.8459

Page 1/1

Supplementary Figure 229. HPLC spectra of compound **6h**

Data File E:\DATA\RJT\2022-02-0917-35-296OBn-YD.D
Sample Name: 6OBn-YD

===

Acq. Operator : 系统
Sample Operator : 系统
Acq. Instrument : LC1 Location : -
Injection Date : 2022/2/9 17:35:29
Inj Volume :
Acq. Method : C:\Chem32\1\Methods\ybm.M
Last changed : 2022/2/9 17:21:38 : 系统
(modified after loading)
Analysis Method : C:\Chem32\1\Methods\def_LC.M
Last changed : 2022/4/2 11:28:24 : 系统
(modified after loading)
Sample Info : IB-3, hex/ipr=99:1, 254 nm,1.0 ml/min,68.82 bar

Additional Info : Peak(s) manually integrated

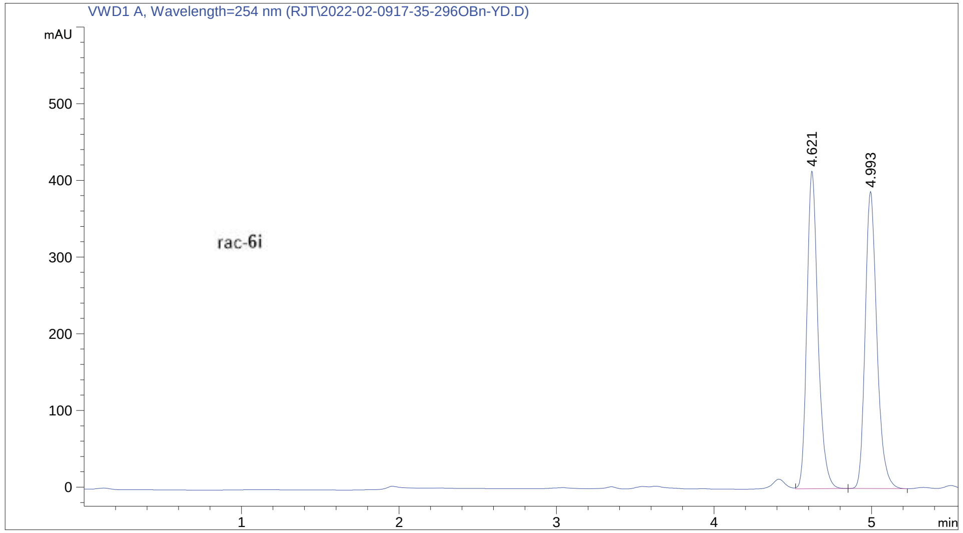

===
Area Percent Report
===

Sorted By : Signal
Multiplier : 1.0000
Dilution : 1.0000
Sample Amount: : 1.00000 [ng/ul] (not used in calc.)
Use Multiplier & Dilution Factor with ISTDs

Signal 1: VWD1 A, Wavelength=254 nm

Peak #	RetTime [min]	Type	Width [min]	Area [mAU*s]	Height [mAU]	Area %
1	4.621	FM	0.0814	2024.98474	414.59256	50.2290
2	4.993	BB	0.0795	2006.52209	387.23795	49.7710

LC1 2022/4/2 11:28:28 系统

Data File E:\DATA\RJT\2022-02-0917-29-206OBn-YD CHIRAL.D
Sample Name: 6OBn-YD CHIRAL

===

Acq. Operator : 系统
Sample Operator : 系统
Acq. Instrument : LC1 Location : -
Injection Date : 2022/2/9 17:29:20
Inj Volume :
Acq. Method : C:\Chem32\1\Methods\ybm.M
Last changed : 2022/2/9 17:21:38 : 系统
(modified after loading)
Analysis Method : C:\Chem32\1\Methods\def_LC.M
Last changed : 2022/4/2 11:28:52 : 系统
(modified after loading)
Sample Info : IB-3, hex/ipr=99:1, 254 nm,1.0 ml/min,68.82 bar

Additional Info : Peak(s) manually integrated

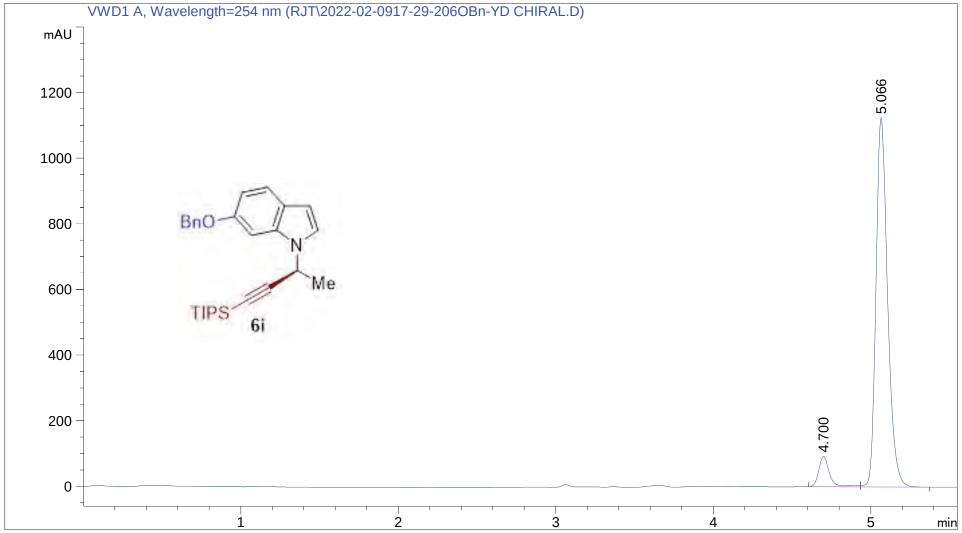

===
Area Percent Report
===

Sorted By : Signal
Multiplier : 1.0000
Dilution : 1.0000
Sample Amount: : 1.00000 [ng/ul] (not used in calc.)
Use Multiplier & Dilution Factor with ISTDs

Signal 1: VWD1 A, Wavelength=254 nm

Peak #	RetTime [min]	Type	Width [min]	Area [mAU*s]	Height [mAU]	Area %
1	4.700	BV R	0.0693	441.23593	92.18320	7.1109
2	5.066	VB	0.0788	5763.85059	1124.56250	92.8891

Supplementary Figure 230. HPLC spectra of compound **6i**

Data File E:\DATA\RJT\2022-01-3018-52-4656HJ-YD.D
Sample Name: 56HJ-YD

===

Acq. Operator : 系统
Sample Operator : 系统
Acq. Instrument : LC1 Location : -
Injection Date : 2022/1/30 18:52:46
Inj Volume :
Acq. Method : C:\Chem32\1\Methods\ybm.M
Last changed : 2022/1/30 18:10:17 : 系统
(modified after loading)
Analysis Method : C:\Chem32\1\Methods\def_LC.M
Last changed : 2022/4/2 11:33:59 : 系统
(modified after loading)
Sample Info : IB-3, hex/ipr=99/1, 254 nm, 1.0 ml/min, 69.75 bar

Additional Info : Peak(s) manually integrated

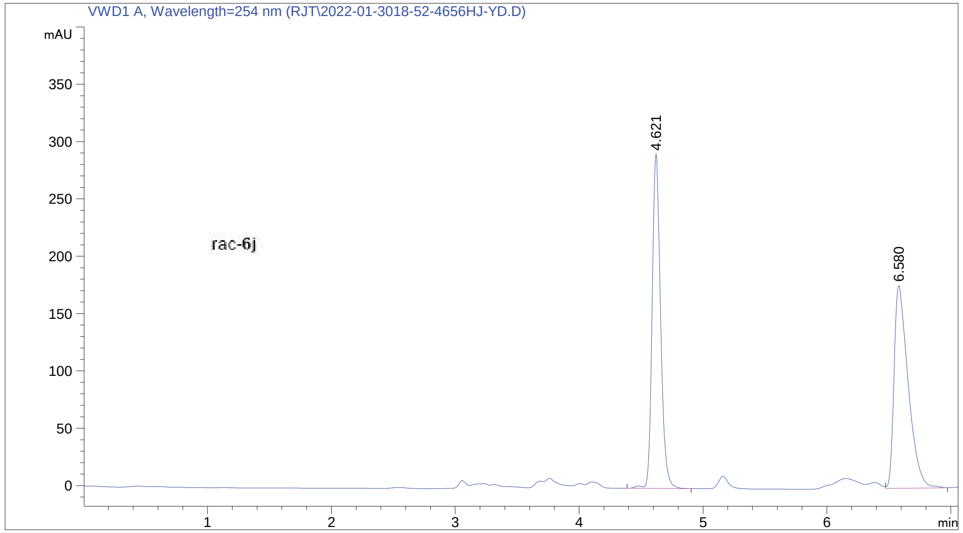

===
Area Percent Report
===

Sorted By : Signal
Multiplier : 1.0000
Dilution : 1.0000
Sample Amount: : 1.00000 [ng/ul] (not used in calc.)
Use Multiplier & Dilution Factor with ISTDs

Signal 1: VWD1 A, Wavelength=254 nm

Peak #	RetTime [min]	Type	Width [min]	Area [mAU*s]	Height [mAU]	Area %
1	4.621	VB R	0.0717	1376.39783	291.51300	49.5709
2	6.580	FM	0.1320	1400.22791	176.79411	50.4291

LC1 2022/4/2 11:34:02 系统

Data File E:\DATA\RJT\2022-01-3019-11-56597.D
Sample Name: 597

===

Acq. Operator : 系统
Sample Operator : 系统
Acq. Instrument : LC1 Location : -
Injection Date : 2022/1/30 19:11:56
Inj Volume :
Acq. Method : C:\Chem32\1\Methods\ybm.M
Last changed : 2022/1/30 18:10:17 : 系统
(modified after loading)
Analysis Method : C:\Chem32\1\Methods\def_LC.M
Last changed : 2022/4/2 11:32:57 : 系统
(modified after loading)
Sample Info : IB-3, hex/ipr=99/1, 254 nm, 1.0 ml/min, 69.75 bar

Additional Info : Peak(s) manually integrated

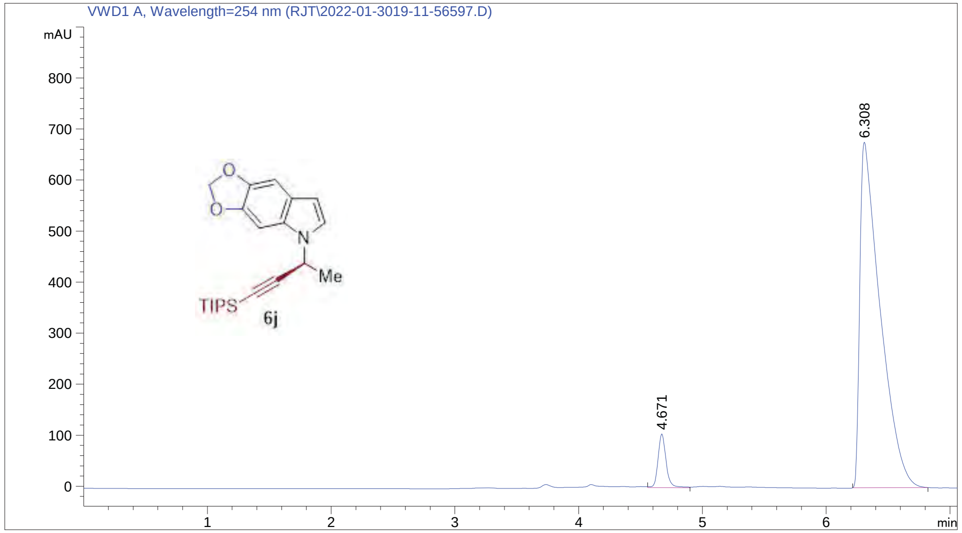

===
Area Percent Report
===

Sorted By : Signal
Multiplier : 1.0000
Dilution : 1.0000
Sample Amount: : 1.00000 [ng/ul] (not used in calc.)
Use Multiplier & Dilution Factor with ISTDs

Signal 1: VWD1 A, Wavelength=254 nm

Peak #	RetTime [min]	Type	Width [min]	Area [mAU*s]	Height [mAU]	Area %
1	4.671	MM	0.0752	475.19281	105.28246	5.7879
2	6.308	MM	0.1903	7734.94385	677.30414	94.2121

Page 1/1

Supplementary Figure 231. HPLC spectra of compound **6j**

Data File E:\DATA\RJT\2022-02-1823-10-407F.D
Sample Name: 7F

===

Acq. Operator : 系统
Sample Operator : 系统
Acq. Instrument : LC1 Location : -
Injection Date : 2022/2/18 23:10:40
Inj Volume :
Acq. Method : C:\Chem32\1\Methods\ybm.M
Last changed : 2022/2/18 23:07:38 : 系统
(modified after loading)
Analysis Method : C:\Chem32\1\Methods\def_LC.M
Last changed : 2022/4/2 12:23:39 : 系统
(modified after loading)
Sample Info : IB-3, hex/ipr=100:0, 254 nm, 1.0 ml/min, 67.40 bar

Additional Info : Peak(s) manually integrated

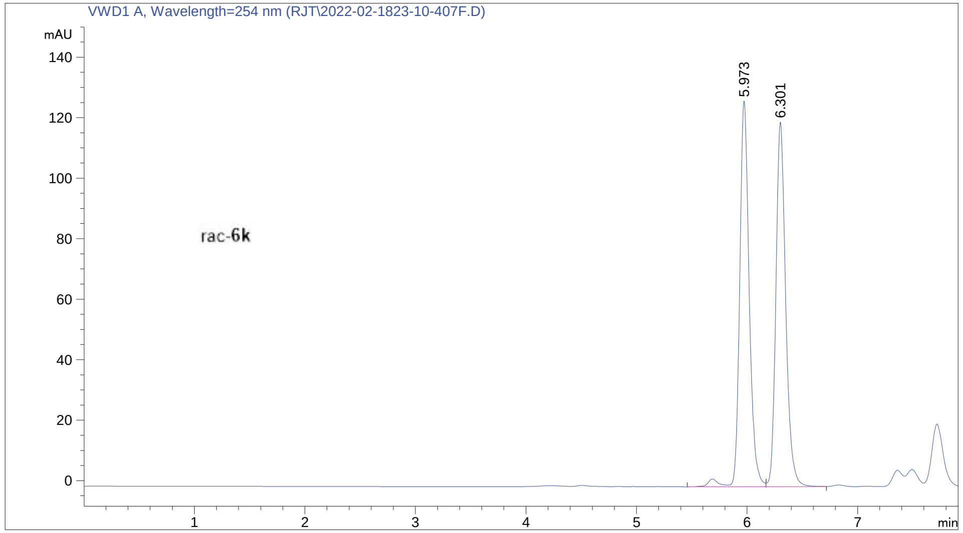

===
Area Percent Report
===

Sorted By : Signal
Multiplier : 1.0000
Dilution : 1.0000
Sample Amount: : 1.00000 [ng/ul] (not used in calc.)
Use Multiplier & Dilution Factor with ISTDs

Signal 1: VWD1 A, Wavelength=254 nm

Peak #	RetTime [min]	Type	Width [min]	Area [mAU*s]	Height [mAU]	Area %
1	5.973	VV R	0.0896	768.05377	127.51984	50.9414
2	6.301	VB	0.0936	739.66785	120.44535	49.0586

LC1 2022/4/2 12:24:05 系统

Data File E:\DATA\RJT\2022-02-1823-19-487F.D
Sample Name: 7F

===

Acq. Operator : 系统
Sample Operator : 系统
Acq. Instrument : LC1 Location : -
Injection Date : 2022/2/18 23:19:48
Inj Volume :
Acq. Method : C:\Chem32\1\Methods\ybm.M
Last changed : 2022/2/18 23:07:38 : 系统
(modified after loading)
Analysis Method : C:\Chem32\1\Methods\def_LC.M
Last changed : 2022/4/2 12:22:55 : 系统
(modified after loading)
Sample Info : IB-3, hex/ipr=100:0, 254 nm, 1.0 ml/min, 67.40 bar

Additional Info : Peak(s) manually integrated

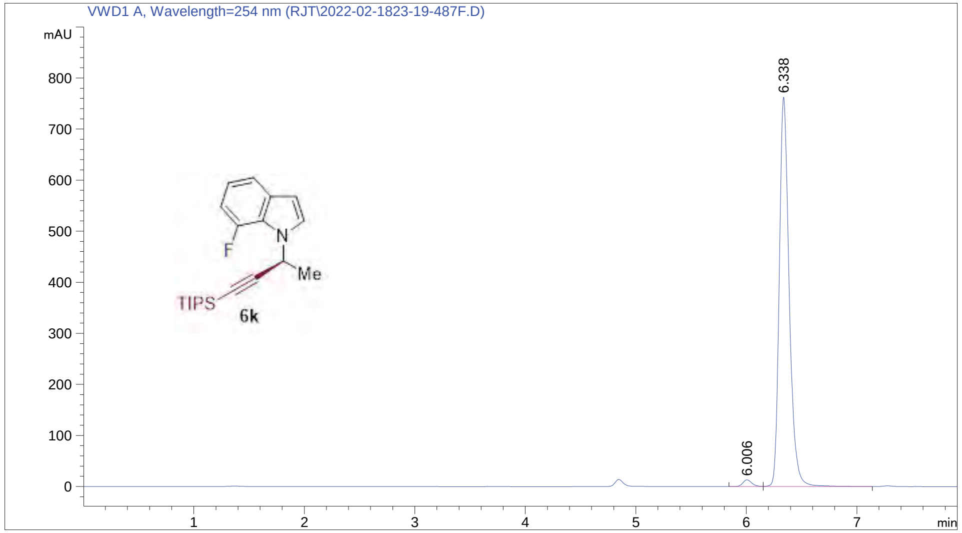

===
Area Percent Report
===

Sorted By : Signal
Multiplier : 1.0000
Dilution : 1.0000
Sample Amount: : 1.00000 [ng/ul] (not used in calc.)
Use Multiplier & Dilution Factor with ISTDs

Signal 1: VWD1 A, Wavelength=254 nm

Peak #	RetTime [min]	Type	Width [min]	Area [mAU*s]	Height [mAU]	Area %
1	6.006	BV	0.0889	76.90068	13.21250	1.5920
2	6.338	VV	0.0947	4753.67383	762.74133	98.4080

Page 1/1

Supplementary Figure 232. HPLC spectra of compound **6k**

Data File E:\DATA\RJT\2022-02-1521-38-477-OMe.D
Sample Name: 7-OMe

===

Acq. Operator : 系统
Sample Operator : 系统
Acq. Instrument : LC1 Location : -
Injection Date : 2022/2/15 21:38:47
Inj Volume :
Acq. Method : C:\Chem32\1\Methods\ybm.M
Last changed : 2022/2/15 21:18:22 : 系统
(modified after loading)
Analysis Method : C:\Chem32\1\Methods\def_LC.M
Last changed : 2022/4/4 10:21:20 : 系统
(modified after loading)
Sample Info : IB-3, hex/ipr=100:0, 254 nm,0.7 ml/min,46.15 bar

Additional Info : Peak(s) manually integrated

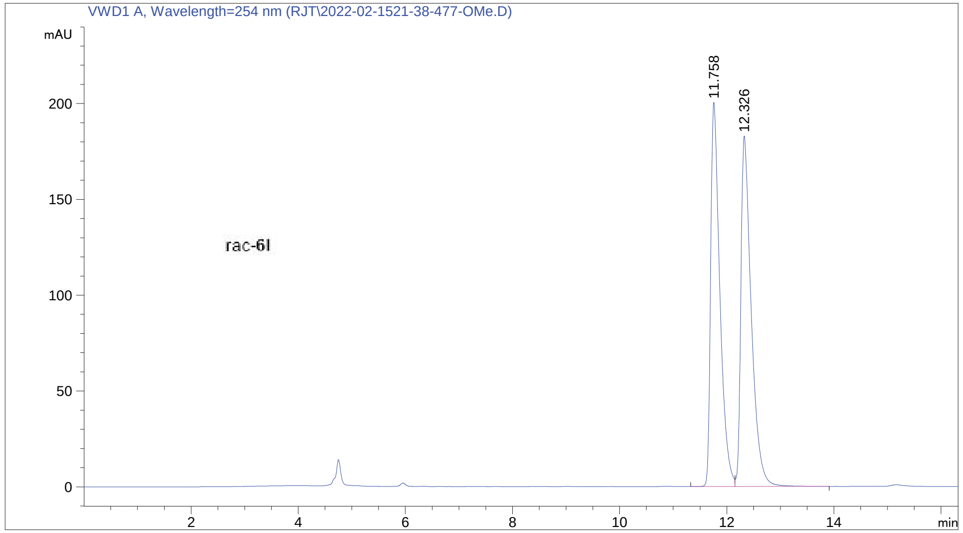

===
Area Percent Report
===

Sorted By : Signal
Multiplier : 1.0000
Dilution : 1.0000
Sample Amount: : 1.00000 [ng/ul] (not used in calc.)
Use Multiplier & Dilution Factor with ISTDs

Signal 1: VWD1 A, Wavelength=254 nm

Peak #	RetTime [min]	Type	Width [min]	Area [mAU*s]	Height [mAU]	Area %
1	11.758	BV	0.1796	2398.61670	200.38821	49.3007
2	12.326	VB	0.2003	2466.66602	182.93533	50.6993

LC1 2022/4/4 10:21:22 系统

Data File E:\DATA\RJT\2022-02-1521-55-387-OMe.D
Sample Name: 7-OMe

===

Acq. Operator : 系统
Sample Operator : 系统
Acq. Instrument : LC1 Location : -
Injection Date : 2022/2/15 21:55:38
Inj Volume :
Acq. Method : C:\Chem32\1\Methods\ybm.M
Last changed : 2022/2/15 21:18:22 : 系统
(modified after loading)
Analysis Method : C:\Chem32\1\Methods\def_LC.M
Last changed : 2022/4/2 12:25:09 : 系统
(modified after loading)
Sample Info : IB-3, hex/ipr=100:0, 254 nm,0.7 ml/min,46.15 bar

Additional Info : Peak(s) manually integrated

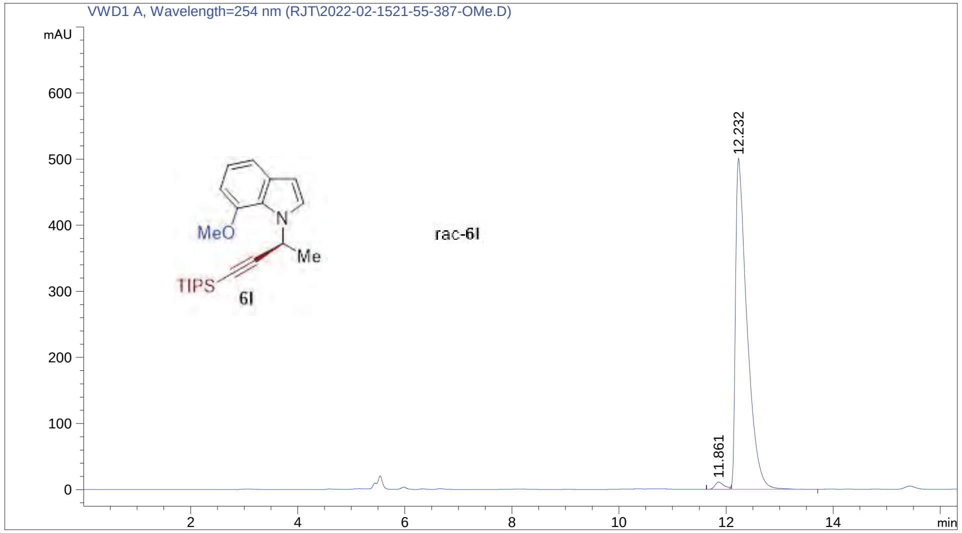

===
Area Percent Report
===

Sorted By : Signal
Multiplier : 1.0000
Dilution : 1.0000
Sample Amount: : 1.00000 [ng/ul] (not used in calc.)
Use Multiplier & Dilution Factor with ISTDs

Signal 1: VWD1 A, Wavelength=254 nm

Peak #	RetTime [min]	Type	Width [min]	Area [mAU*s]	Height [mAU]	Area %
1	11.861	BV E	0.1730	128.35988	10.93449	1.6836
2	12.232	VB R	0.2188	7495.72998	500.30328	98.3164

Page 1/1

Supplementary Figure 233. HPLC spectra of compound **6l**

Data File E:\DATA\RJT\2022-02-1422-43-013-OTBS.D
Sample Name: 3-OTBS

===

Acq. Operator : 系统
Sample Operator : 系统
Acq. Instrument : LC1 Location : -
Injection Date : 2022/2/14 22:43:01
Inj Volume :
Acq. Method : C:\Chem32\1\Methods\ybm.M
Last changed : 2022/2/14 19:58:44 : 系统
(modified after loading)
Analysis Method : C:\Chem32\1\Methods\def_LC.M
Last changed : 2022/4/2 12:29:10 : 系统
(modified after loading)
Sample Info : IB-3, hex/ipr=100:0, 254 nm, 1.0 ml/min, 66.90 bar

Additional Info : Peak(s) manually integrated

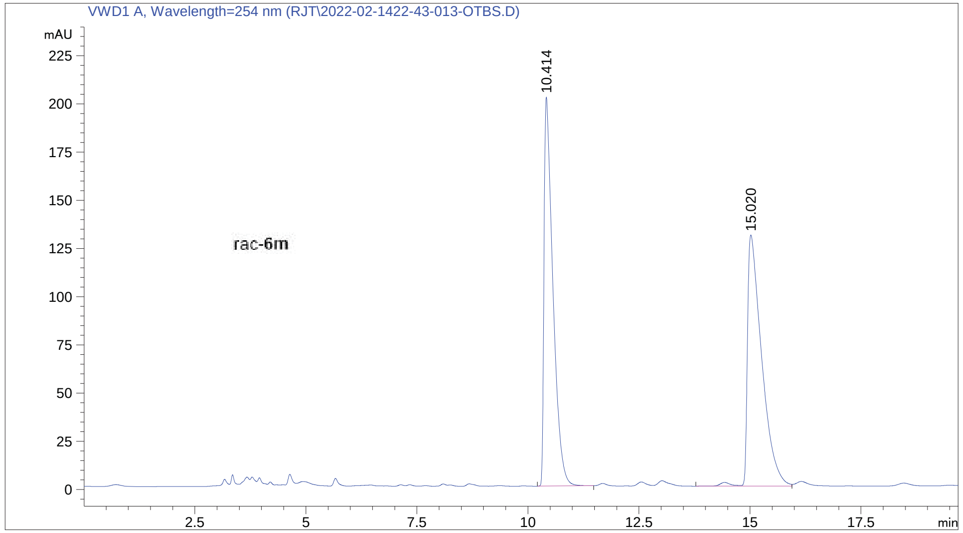

===
Area Percent Report
===

Sorted By : Signal
Multiplier : 1.0000
Dilution : 1.0000
Sample Amount: : 1.00000 [ng/ul] (not used in calc.)
Use Multiplier & Dilution Factor with ISTDs

Signal 1: VWD1 A, Wavelength=254 nm

Peak #	RetTime [min]	Type	Width [min]	Area [mAU*s]	Height [mAU]	Area %
1	10.414	BB	0.2059	2816.75073	201.75871	49.1251
2	15.020	MF	0.3731	2917.08423	130.32414	50.8749

Data File E:\DATA\RJT\2022-02-1421-07-433-OTBS.D
Sample Name: 3-OTBS

===

Acq. Operator : 系统
Sample Operator : 系统
Acq. Instrument : LC1 Location : -
Injection Date : 2022/2/14 21:07:43
Inj Volume :
Acq. Method : C:\Chem32\1\Methods\ybm.M
Last changed : 2022/2/14 19:58:44 : 系统
(modified after loading)
Analysis Method : C:\Chem32\1\Methods\def_LC.M
Last changed : 2022/4/2 12:28:12 : 系统
(modified after loading)
Sample Info : IB-3, hex/ipr=100:0, 254 nm, 1.0 ml/min, 66.90 bar

Additional Info : Peak(s) manually integrated

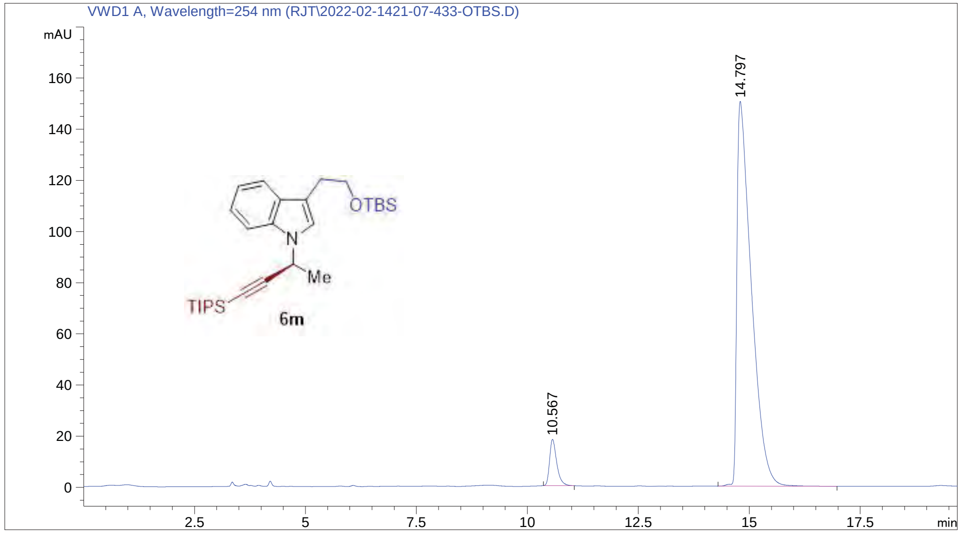

===
Area Percent Report
===

Sorted By : Signal
Multiplier : 1.0000
Dilution : 1.0000
Sample Amount: : 1.00000 [ng/ul] (not used in calc.)
Use Multiplier & Dilution Factor with ISTDs

Signal 1: VWD1 A, Wavelength=254 nm

Peak #	RetTime [min]	Type	Width [min]	Area [mAU*s]	Height [mAU]	Area %
1	10.567	MM	0.1857	201.89435	18.12244	5.6013
2	14.797	VB R	0.3339	3402.52686	150.44760	94.3987

LC1 2022/4/2 12:29:12 系统

Page 1/1

Supplementary Figure 234. HPLC spectra of compound **6m**

Data File E:\DATA\RJT\2022-03-1323-13-03SHKZ-R.D
Sample Name: SHKZ-R

===

Acq. Operator : 系统
Sample Operator : 系统
Acq. Instrument : LC1 Location : -
Injection Date : 2022/3/13 23:13:03
Inj Volume :
Acq. Method : C:\Chem32\1\Methods\ybm.M
Last changed : 2022/3/13 22:17:57 : 系统
(modified after loading)
Analysis Method : C:\Chem32\1\Methods\def_LC.M
Last changed : 2022/4/2 12:37:22 : 系统
(modified after loading)
Sample Info : IB-3, hex/ipr=100/0, 254 nm, 1.0 ml/min, 69.00 bar

Additional Info : Peak(s) manually integrated

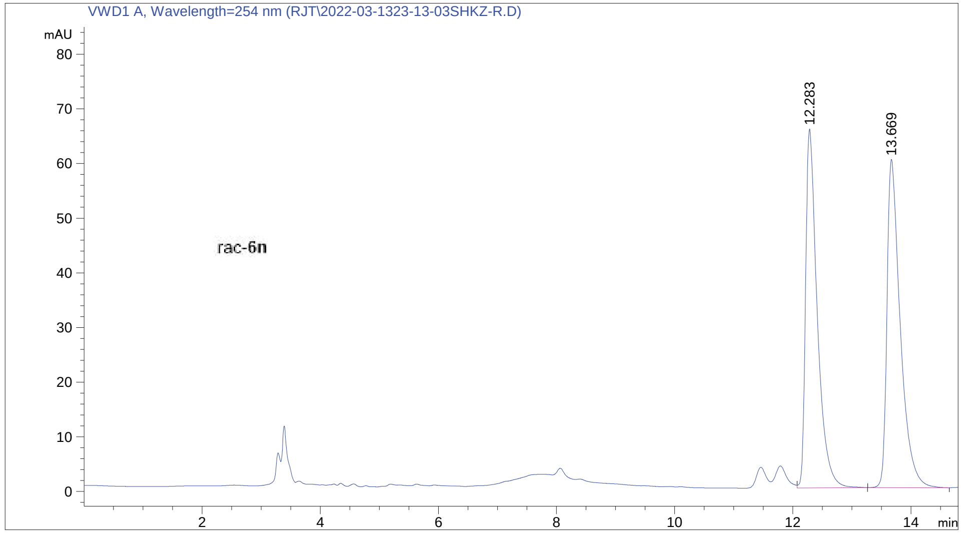

===
Area Percent Report
===

Sorted By : Signal
Multiplier : 1.0000
Dilution : 1.0000
Sample Amount: : 1.00000 [ng/ul] (not used in calc.)
Use Multiplier & Dilution Factor with ISTDs

Signal 1: VWD1 A, Wavelength=254 nm

Peak #	RetTime [min]	Type	Width [min]	Area [mAU*s]	Height [mAU]	Area %
1	12.283	FM	0.2285	900.27808	65.66745	48.9483
2	13.669	BB	0.2304	938.96472	60.06259	51.0517

Data File E:\DATA\RJT\2022-03-1322-37-54SHKZ-C.D
Sample Name: SHKZ-C

===

Acq. Operator : 系统
Sample Operator : 系统
Acq. Instrument : LC1 Location : -
Injection Date : 2022/3/13 22:37:54
Inj Volume :
Acq. Method : C:\Chem32\1\Methods\ybm.M
Last changed : 2022/3/13 22:17:57 : 系统
(modified after loading)
Analysis Method : C:\Chem32\1\Methods\def_LC.M
Last changed : 2022/4/2 12:36:21 : 系统
(modified after loading)
Sample Info : IB-3, hex/ipr=100/0, 254 nm, 1.0 ml/min, 69.00 bar

Additional Info : Peak(s) manually integrated

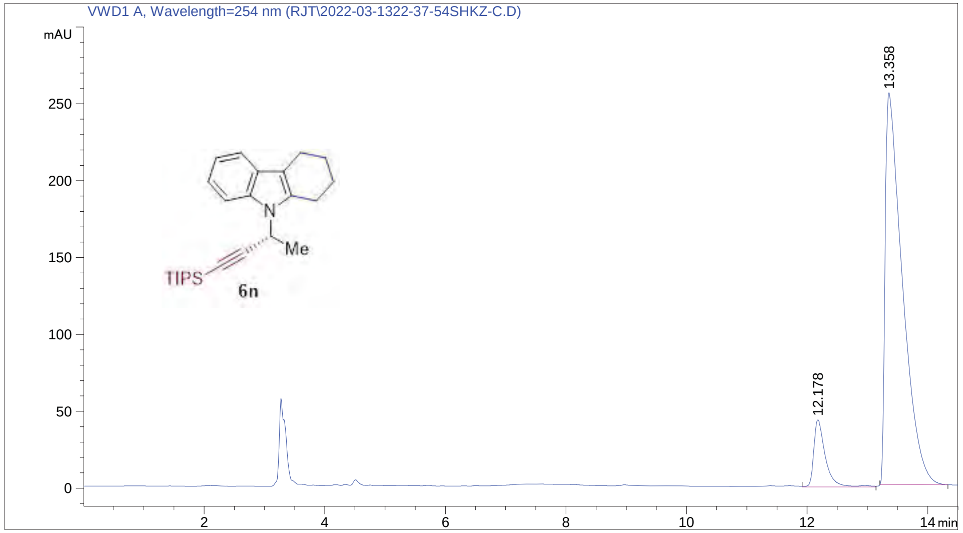

===
Area Percent Report
===

Sorted By : Signal
Multiplier : 1.0000
Dilution : 1.0000
Sample Amount: : 1.00000 [ng/ul] (not used in calc.)
Use Multiplier & Dilution Factor with ISTDs

Signal 1: VWD1 A, Wavelength=254 nm

Peak #	RetTime [min]	Type	Width [min]	Area [mAU*s]	Height [mAU]	Area %
1	12.178	MM	0.2232	584.58887	43.64915	10.3052
2	13.358	MM	0.3328	5088.18457	254.82747	89.6948

Supplementary Figure 235. HPLC spectra of compound **6n**

Data File E:\DATA\RJT\2022-03-0419-11-32RJT-4-749 R.D
Sample Name: RJT-4-749 R

===

Acq. Operator : 系统
Sample Operator : 系统
Acq. Instrument : LC1 Location : -
Injection Date : 2022/3/4 19:11:32
Inj Volume :
Acq. Method : C:\Chem32\1\Methods\ybm.M
Last changed : 2022/3/4 19:11:05 : 系统
(modified after loading)
Analysis Method : C:\Chem32\1\Methods\def_LC.M
Last changed : 2022/4/2 12:41:21 : 系统
(modified after loading)
Sample Info : IB-3, hex/ipr=100/0, 254 nm, 1.0 ml/min, 58.51 bar

Additional Info : Peak(s) manually integrated

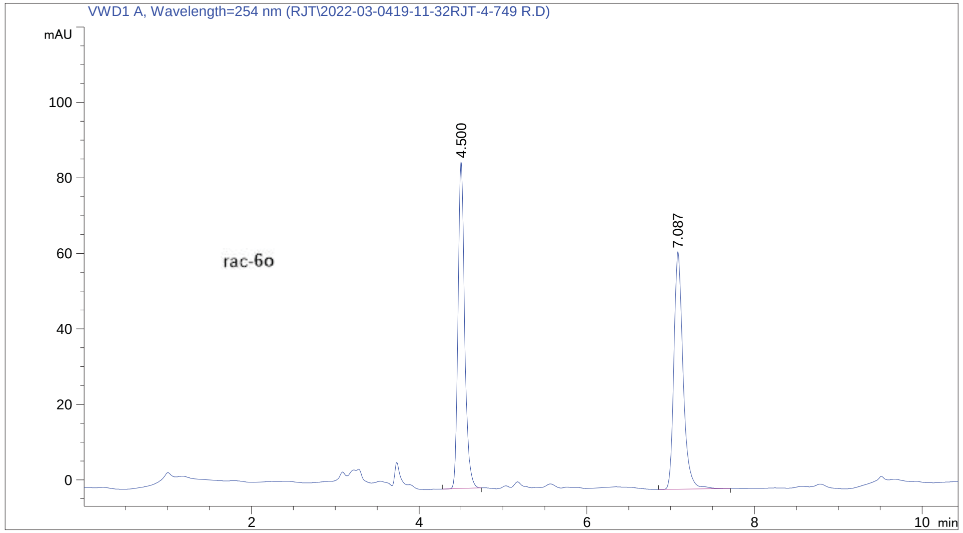

===
Area Percent Report
===

Sorted By : Signal
Multiplier : 1.0000
Dilution : 1.0000
Sample Amount: : 1.00000 [ng/ul] (not used in calc.)
Use Multiplier & Dilution Factor with ISTDs

Signal 1: VWD1 A, Wavelength=254 nm

Peak #	RetTime [min]	Type	Width [min]	Area [mAU*s]	Height [mAU]	Area %
1	4.500	BB	0.0829	473.82555	85.48895	49.7033
2	7.087	BB	0.1159	479.48305	52.89309	50.2967

Data File E:\DATA\RJT\2022-03-0418-25-07RJT-4-749 C.D
Sample Name: RJT-4-749 C

===

Acq. Operator : 系统
Sample Operator : 系统
Acq. Instrument : LC1 Location : -
Injection Date : 2022/3/4 18:25:07
Inj Volume :
Acq. Method : C:\Chem32\1\Methods\ybm.M
Last changed : 2022/3/4 18:04:14 : 系统
(modified after loading)
Analysis Method : C:\Chem32\1\Methods\def_LC.M
Last changed : 2022/4/2 12:39:05 : 系统
(modified after loading)
Sample Info : IB-3, hex/ipr=100/0, 254 nm, 1.0 ml/min, 58.51 bar

Additional Info : Peak(s) manually integrated

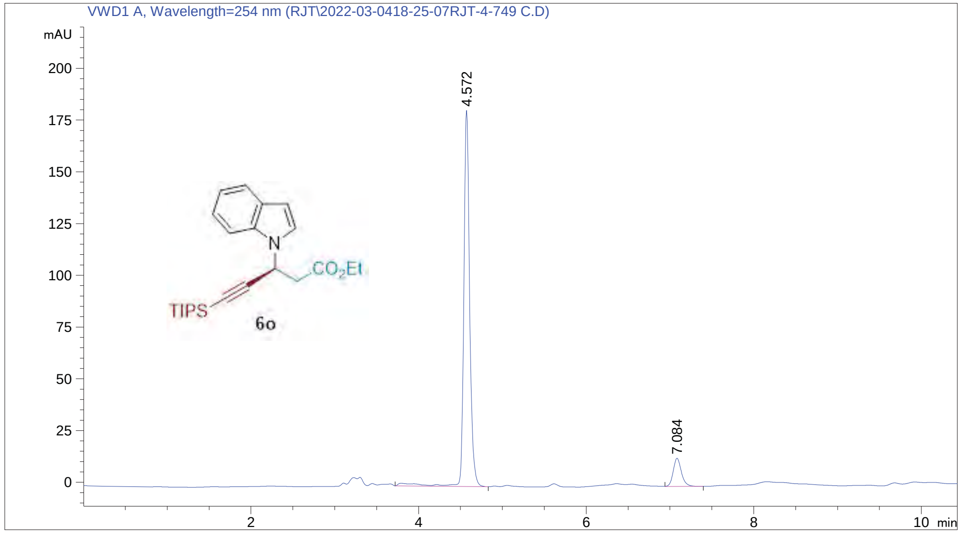

===
Area Percent Report
===

Sorted By : Signal
Multiplier : 1.0000
Dilution : 1.0000
Sample Amount: : 1.00000 [ng/ul] (not used in calc.)
Use Multiplier & Dilution Factor with ISTDs

Signal 1: VWD1 A, Wavelength=254 nm

Peak #	RetTime [min]	Type	Width [min]	Area [mAU*s]	Height [mAU]	Area %
1	4.572	VB R	0.0742	890.49408	181.77304	90.4842
2	7.084	FM	0.1142	93.64950	13.67328	9.5158

LC1 2022/4/2 12:41:23 系统

Page 1/1

Supplementary Figure 236. HPLC spectra of compound **6o**

Data File E:\DATA\RJT\2022-03-2014-08-11TJNX- RACE.D
Sample Name: TJNX- RACE

===

Acq. Operator : 系统
Sample Operator : 系统
Acq. Instrument : LC1 Location : -
Injection Date : 2022/3/20 14:08:11
Inj Volume :
Acq. Method : C:\Chem32\1\Methods\def_LC.M
Last changed : 2022/3/20 14:06:14 : 系统
(modified after loading)
Analysis Method : C:\Chem32\1\Methods\def_LC.M
Last changed : 2022/4/2 12:42:49 : 系统
(modified after loading)
Sample Info : OD-H, hex/ipr=90/10, 254nm, 1.0 ml/min, 53.73 bar

Additional Info : Peak(s) manually integrated

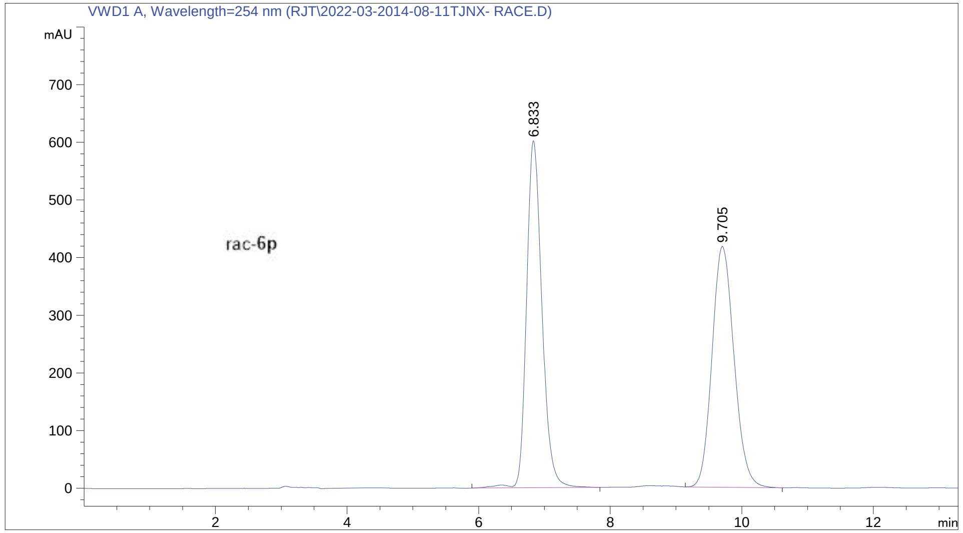

===
Area Percent Report
===

Sorted By : Signal
Multiplier : 1.0000
Dilution : 1.0000
Sample Amount: : 1.00000 [ng/ul] (not used in calc.)
Use Multiplier & Dilution Factor with ISTDs

Signal 1: VWD1 A, Wavelength=254 nm

Peak #	RetTime [min]	Type	Width [min]	Area [mAU*s]	Height [mAU]	Area %
1	6.833	VB R	0.2492	9795.94922	601.71332	50.2045
2	9.705	BB	0.3635	9716.16309	417.49167	49.7955

LC1 2022/4/2 12:42:51 系统

Data File E:\DATA\RJT\2022-03-2014-22-13TJNX- CHIRAL.D
Sample Name: TJNX- CHIRAL

===

Acq. Operator : 系统
Sample Operator : 系统
Acq. Instrument : LC1 Location : -
Injection Date : 2022/3/20 14:22:13
Inj Volume :
Acq. Method : C:\Chem32\1\Methods\def_LC.M
Last changed : 2022/3/20 14:06:14 : 系统
(modified after loading)
Analysis Method : C:\Chem32\1\Methods\def_LC.M
Last changed : 2022/4/2 12:42:24 : 系统
(modified after loading)
Sample Info : OD-H, hex/ipr=90/10, 254nm, 1.0 ml/min, 52.58 bar

Additional Info : Peak(s) manually integrated

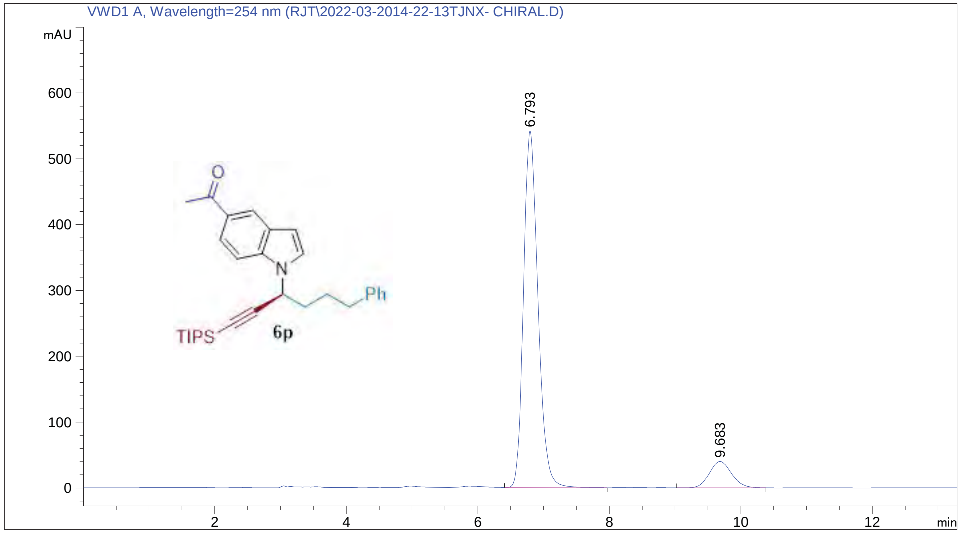

===
Area Percent Report
===

Sorted By : Signal
Multiplier : 1.0000
Dilution : 1.0000
Sample Amount: : 1.00000 [ng/ul] (not used in calc.)
Use Multiplier & Dilution Factor with ISTDs

Signal 1: VWD1 A, Wavelength=254 nm

Peak #	RetTime [min]	Type	Width [min]	Area [mAU*s]	Height [mAU]	Area %
1	6.793	VB	0.2350	8264.26953	541.63770	89.7806
2	9.683	BB	0.3642	940.69177	40.02388	10.2194

Page 1/1

Supplementary Figure 237. HPLC spectra of compound **6p**

```
Data File E:\DATA\RJT\2022-02-1920-18-04TBS.D
Sample Name: TBS

=====================================================================
Acq. Operator   : 系统
Sample Operator : 系统
Acq. Instrument : LC1                            Location :     -
Injection Date  : 2022/2/19 20:18:04
                                               Inj Volume :
Acq. Method     : C:\Chem32\1\Methods\ybm.M
Last changed    : 2022/2/19 20:17:31 :  系统
                  (modified after loading)
Analysis Method : C:\Chem32\1\Methods\def_LC.M
Last changed    : 2022/4/2 12:46:41 :  系统
                  (modified after loading)
Sample Info     : IB-3, hex/ipr=100:0, 254 nm,1.0 ml/min,66.14 bar

Additional Info : Peak(s) manually integrated
```

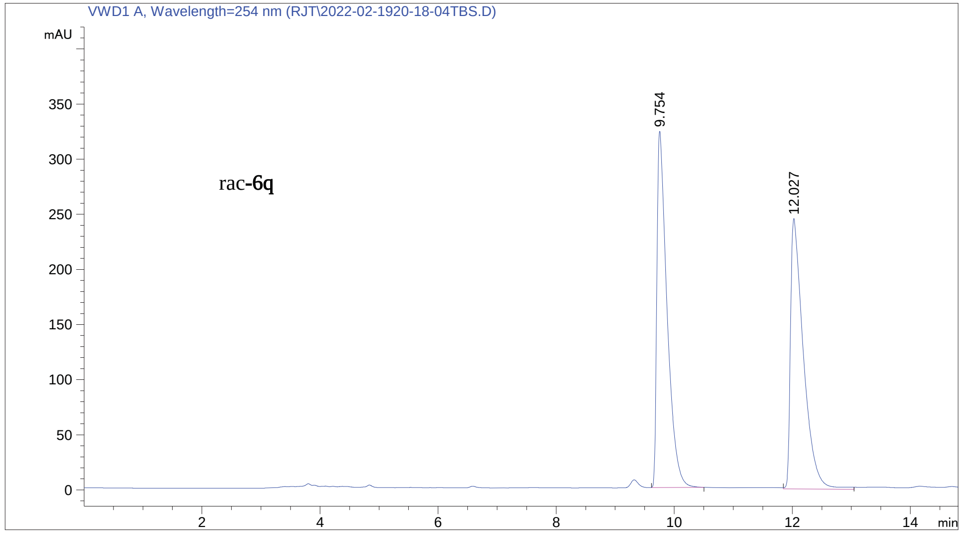


```
=====================================================================
                         Area Percent Report
=====================================================================

Sorted By             :      Signal
Multiplier                   :      1.0000
Dilution                     :      1.0000
Sample Amount:                          :      1.00000  [ng/ul]    (not used in calc.)
Use Multiplier & Dilution Factor with ISTDs


Signal 1: VWD1 A, Wavelength=254 nm
```

Peak #	RetTime [min]	Type	Width [min]	Area [mAU*s]	Height [mAU]	Area %
1	9.754	MM	0.1996	3871.56396	323.22079	50.9003
2	12.027	MM	0.2538	3734.60864	246.28700	49.0997

```
Data File E:\DATA\RJT\2022-02-1920-48-05TBS.D
Sample Name: TBS

=====================================================================
Acq. Operator   : 系统
Sample Operator : 系统
Acq. Instrument : LC1                            Location :     -
Injection Date  : 2022/2/19 20:48:05
                                               Inj Volume :
Acq. Method     : C:\Chem32\1\Methods\ybm.M
Last changed    : 2022/2/19 20:17:31 :  系统
                  (modified after loading)
Analysis Method : C:\Chem32\1\Methods\def_LC.M
Last changed    : 2022/4/2 12:46:08 :  系统
                  (modified after loading)
Sample Info     : IB-3, hex/ipr=100:0, 254 nm,1.0 ml/min,66.14 bar

Additional Info : Peak(s) manually integrated
```

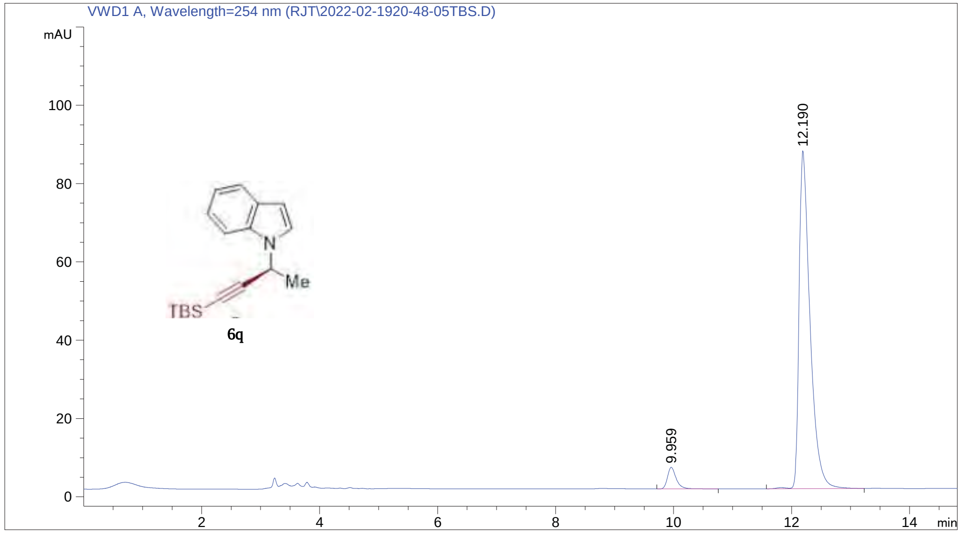


```
=====================================================================
                         Area Percent Report
=====================================================================

Sorted By             :      Signal
Multiplier                   :      1.0000
Dilution                     :      1.0000
Sample Amount:                          :      1.00000  [ng/ul]    (not used in calc.)
Use Multiplier & Dilution Factor with ISTDs


Signal 1: VWD1 A, Wavelength=254 nm
```

Peak #	RetTime [min]	Type	Width [min]	Area [mAU*s]	Height [mAU]	Area %
1	9.959	BB	0.1545	56.63719	5.52905	4.8459
2	12.190	VB R	0.1924	1112.13696	86.28687	95.1541

Supplementary Figure 238. HPLC spectra of compound **6q**

Data File E:\DATA\RJT\2022-03-0219-47-10737.D
Sample Name: 737

===

Acq. Operator : 系统
Sample Operator : 系统
Acq. Instrument : LC1 Location : -
Injection Date : 2022/3/2 19:47:10
Inj Volume :
Acq. Method : C:\Chem32\1\Methods\ybm.M
Last changed : 2022/3/2 15:19:42 : 系统
(modified after loading)
Analysis Method : C:\Chem32\1\Methods\def_LC.M
Last changed : 2022/4/2 12:44:20 : 系统
(modified after loading)
Sample Info : IB-3, hex/ipr=100/0, 254 nm, 1.0 ml/min, 68.00 bar

Additional Info : Peak(s) manually integrated

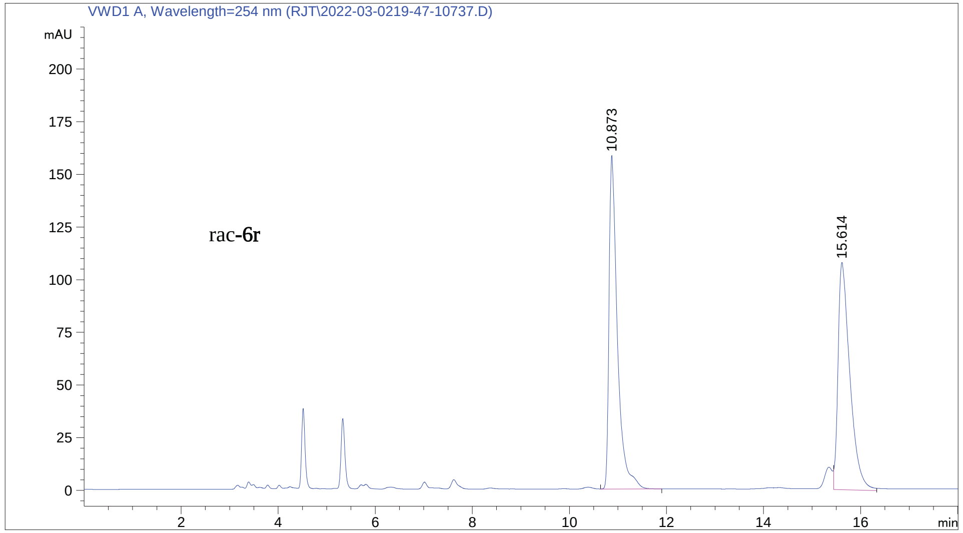

===
Area Percent Report
===

Sorted By : Signal
Multiplier : 1.0000
Dilution : 1.0000
Sample Amount: : 1.00000 [ng/ul] (not used in calc.)
Use Multiplier & Dilution Factor with ISTDs

Signal 1: VWD1 A, Wavelength=254 nm

Peak #	RetTime [min]	Type	Width [min]	Area [mAU*s]	Height [mAU]	Area %
1	10.873	VB	0.1772	1901.79602	158.29018	50.9520
2	15.614	FM	0.2826	1830.72681	107.95129	49.0480

Data File E:\DATA\RJT\2022-03-0219-27-29738.D
Sample Name: 738

===

Acq. Operator : 系统
Sample Operator : 系统
Acq. Instrument : LC1 Location : -
Injection Date : 2022/3/2 19:27:29
Inj Volume :
Acq. Method : C:\Chem32\1\Methods\ybm.M
Last changed : 2022/3/2 15:19:42 : 系统
(modified after loading)
Analysis Method : C:\Chem32\1\Methods\def_LC.M
Last changed : 2022/4/2 12:44:49 : 系统
(modified after loading)
Sample Info : IB-3, hex/ipr=100/0, 254 nm, 1.0 ml/min, 68.00 bar

Additional Info : Peak(s) manually integrated

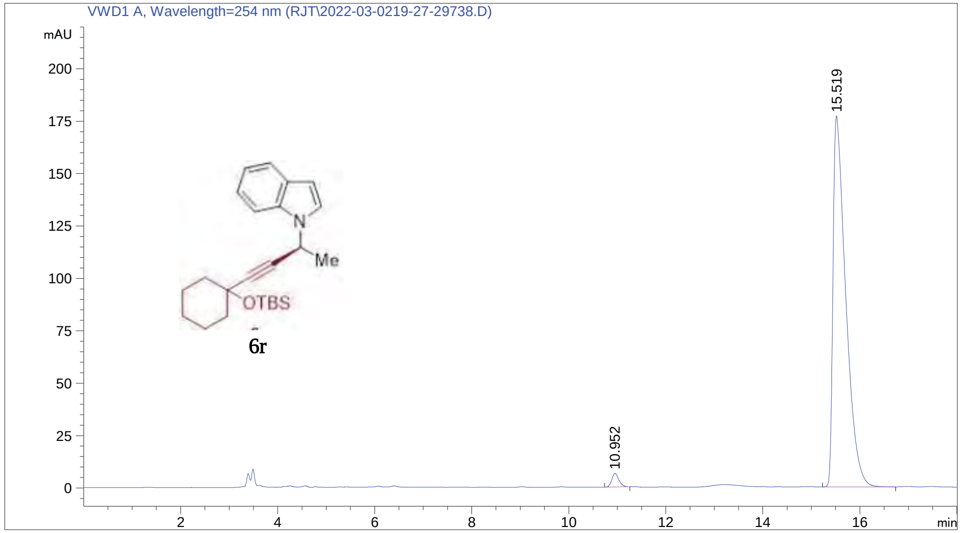

===
Area Percent Report
===

Sorted By : Signal
Multiplier : 1.0000
Dilution : 1.0000
Sample Amount: : 1.00000 [ng/ul] (not used in calc.)
Use Multiplier & Dilution Factor with ISTDs

Signal 1: VWD1 A, Wavelength=254 nm

Peak #	RetTime [min]	Type	Width [min]	Area [mAU*s]	Height [mAU]	Area %
1	10.952	BB	0.1558	66.67954	6.54969	2.0250
2	15.519	BB	0.2707	3226.13110	176.99182	97.9750

Supplementary Figure 239. HPLC spectra of compound **6r**

Data File D:\Chem32\1\Data\RenJiangTao\BC-Ar-R 2022-09-07 18-54-52.D
Sample Name: BC-Ar-R

===

Acq. Operator : SYSTEM
Sample Operator : SYSTEM
Acq. Instrument : 1260 Location : 1
Injection Date : 9/7/2022 6:54:52 PM
Inj Volume : No inj
Acq. Method : D:\Chem32\1\Methods\DEF_LC.M
Last changed : 9/7/2022 6:04:25 PM by SYSTEM
(modified after loading)
Analysis Method : D:\Chem32\1\Methods\DEF_LC.M
Last changed : 9/14/2022 9:37:03 AM by SYSTEM
(modified after loading)
Sample Info : OJ-H, Hex:Ipr=80/20, 1.0 ml/min, 254 nm. 49.62 bar

Additional Info : Peak(s) manually integrated

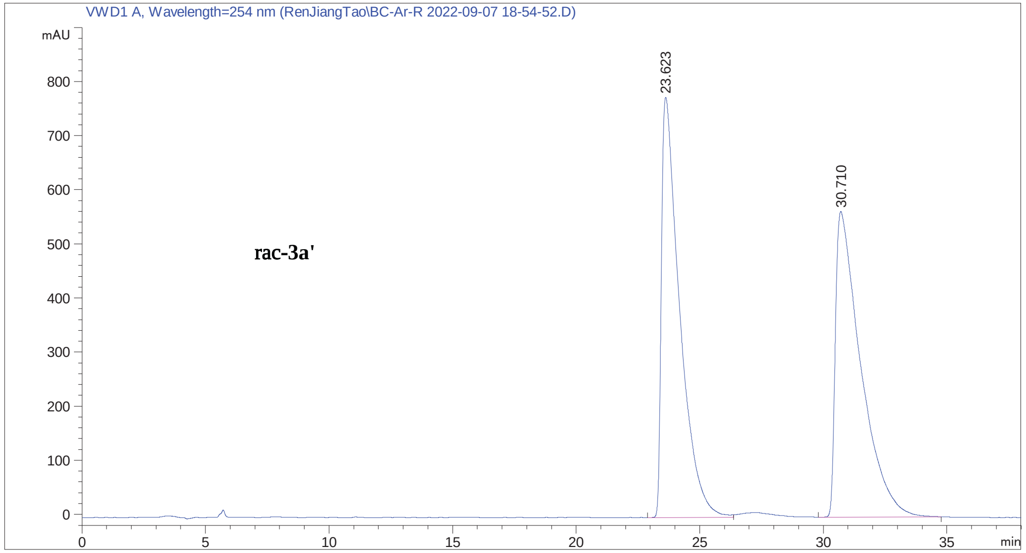

===
Area Percent Report
===

Sorted By : Signal
Multiplier : 1.0000
Dilution : 1.0000
Use Multiplier & Dilution Factor with ISTDs

Signal 1: VWD1 A, Wavelength=254 nm

Peak #	RetTime [min]	Type	Width [min]	Area [mAU*s]	Height [mAU]	Area %
1	23.623	MF	0.8557	3.98770e4	776.73767	49.8076
2	30.710	BB	1.0028	4.01851e4	565.01849	50.1924

Data File D:\Chem32\1\Data\RenJiangTao\BC-Ar-C 2022-09-07 18-15-28.D
Sample Name: BC-Ar-C

===

Acq. Operator : SYSTEM
Sample Operator : SYSTEM
Acq. Instrument : 1260 Location : 1
Injection Date : 9/7/2022 6:15:28 PM
Inj Volume : No inj
Acq. Method : D:\Chem32\1\Methods\DEF_LC.M
Last changed : 9/7/2022 6:04:25 PM by SYSTEM
(modified after loading)
Analysis Method : D:\Chem32\1\Methods\DEF_LC.M
Last changed : 9/14/2022 9:38:24 AM by SYSTEM
(modified after loading)
Sample Info : OJ-H, Hex:Ipr=80/20, 1.0 ml/min, 254 nm. 49.62 bar

Additional Info : Peak(s) manually integrated

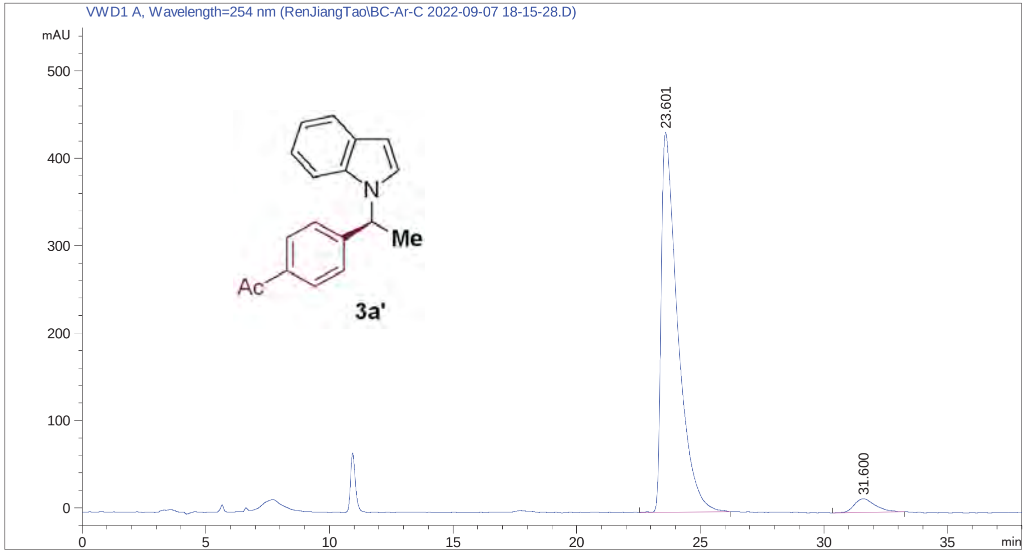

===
Area Percent Report
===

Sorted By : Signal
Multiplier : 1.0000
Dilution : 1.0000
Use Multiplier & Dilution Factor with ISTDs

Signal 1: VWD1 A, Wavelength=254 nm

Peak #	RetTime [min]	Type	Width [min]	Area [mAU*s]	Height [mAU]	Area %
1	23.601	VB R	0.6587	2.02219e4	434.75357	95.5959
2	31.600	BB	0.8748	931.62177	15.62119	4.4041

Supplementary Figure 240. HPLC spectra of compound **3a'**

Data File D:\Chem32\1\Data\RenJiangTao\RJT-BC-XR 2022-09-04 18-15-03.D
Sample Name: RJT-BC-XR

===

Acq. Operator : SYSTEM
Sample Operator : SYSTEM
Acq. Instrument : 1260 Location : 1
Injection Date : 9/4/2022 6:15:03 PM
Inj Volume : No inj
Acq. Method : D:\Chem32\1\Methods\DEF_LC.M
Last changed : 9/4/2022 5:57:18 PM by SYSTEM
(modified after loading)
Analysis Method : D:\Chem32\1\Methods\DEF_LC.M
Last changed : 9/4/2022 6:40:57 PM by SYSTEM
(modified after loading)
Sample Info : AD-H, Hex:Ipr=98/2, 1.0 ml/min, 254 nm.44.49 bar

Additional Info : Peak(s) manually integrated

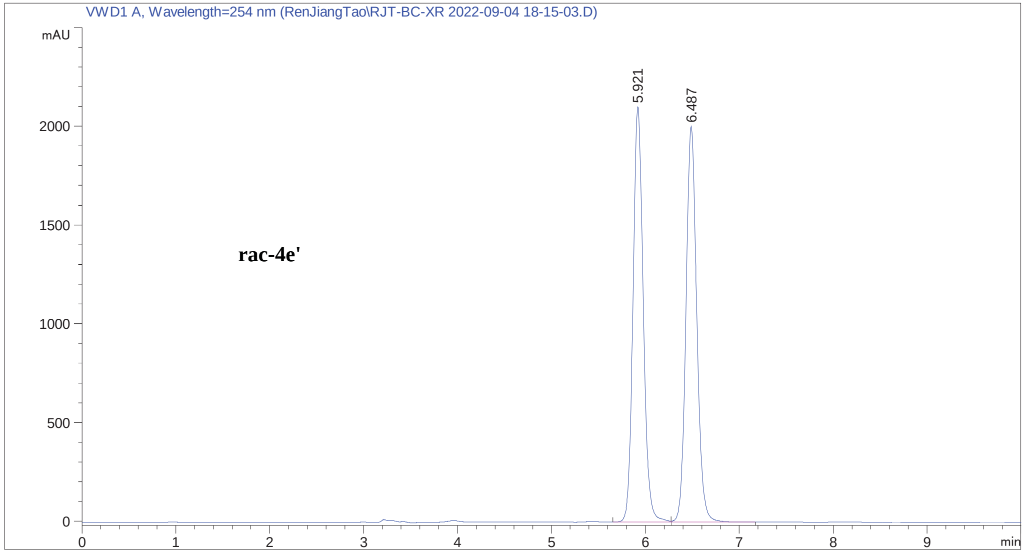

===

Area Percent Report

===

Sorted By : Signal
Multiplier : 1.0000
Dilution : 1.0000
Use Multiplier & Dilution Factor with ISTDs

Signal 1: VWD1 A, Wavelength=254 nm

Peak #	RetTime [min]	Type	Width [min]	Area [mAU*s]	Height [mAU]	Area %
1	5.921	BV	0.1166	1.59606e4	2101.80859	49.9026
2	6.487	VB	0.1233	1.60229e4	2002.58752	50.0974

Data File D:\Chem32\1\Data\RenJiangTao\BC-X-C 2022-09-07 16-19-05.D
Sample Name: BC-X-C

===

Acq. Operator : SYSTEM
Sample Operator : SYSTEM
Acq. Instrument : 1260 Location : 1
Injection Date : 9/7/2022 4:19:05 PM
Inj Volume : No inj
Acq. Method : D:\Chem32\1\Methods\DEF_LC.M
Last changed : 9/7/2022 3:30:04 PM by SYSTEM
(modified after loading)
Analysis Method : D:\Chem32\1\Methods\DEF_LC.M
Last changed : 9/14/2022 9:42:41 AM by SYSTEM
(modified after loading)
Sample Info : AD-H, Hex:Ipr=98/2, 1.0 ml/min, 254 nm. 44.82 bar

Additional Info : Peak(s) manually integrated

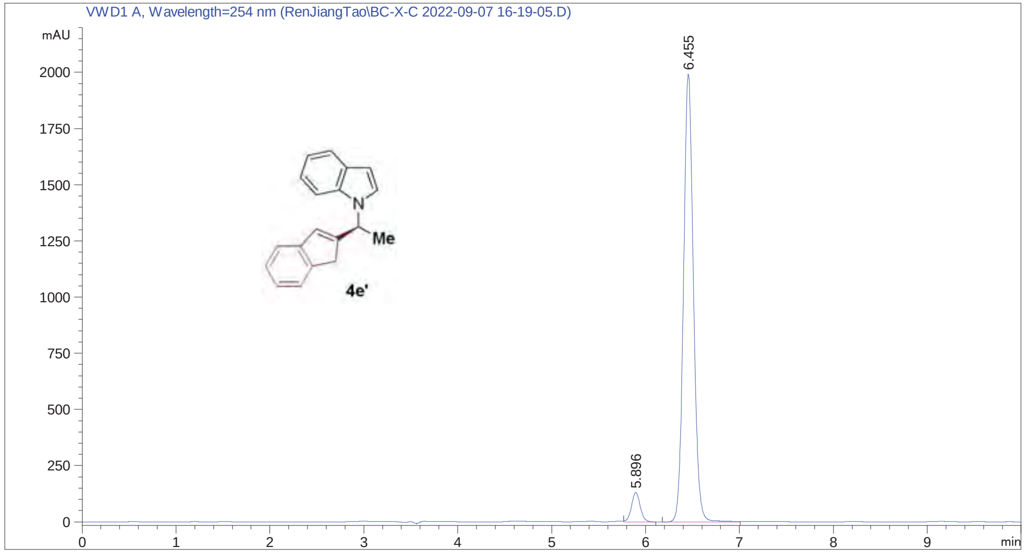

===

Area Percent Report

===

Sorted By : Signal
Multiplier : 1.0000
Dilution : 1.0000
Use Multiplier & Dilution Factor with ISTDs

Signal 1: VWD1 A, Wavelength=254 nm

Peak #	RetTime [min]	Type	Width [min]	Area [mAU*s]	Height [mAU]	Area %
1	5.896	FM	0.1076	849.45386	131.57437	5.4995
2	6.455	MF	0.1220	1.45966e4	1994.02246	94.5005

Supplementary Figure 241. HPLC spectra of compound **4e'**

Data File E:\LCDATA\Data\lilun\2021-11-2111-35-25ll-11-xiao.D
Sample Name: ll-11-xiao

===

Acq. Operator : 系统
Sample Operator : 系统
Acq. Instrument : LC1260 Location : 21
Injection Date : 11/21/2021 11:36:19 AM
Inj Volume : 10.000 µl
Acq. Method : E:\LCDATA\Methods\LI-LUN.M
Last changed : 11/21/2021 10:43:27 AM by 系统
(modified after loading)
Analysis Method : E:\LCDATA\Methods\LI-LUN.M
Last changed : 3/14/2022 4:52:34 PM by 系统
(modified after loading)
Sample Info : oD 90:10 1

Additional Info : Peak(s) manually integrated

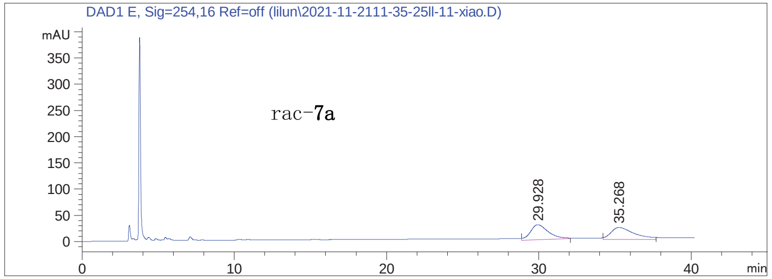

===

Area Percent Report

===

Sorted By : Signal
Multiplier : 1.0000
Dilution : 1.0000
Sample Amount: : 10.00000 [ng/ul] (not used in calc.)
Do not use Multiplier & Dilution Factor with ISTDs

Signal 1: DAD1 E, Sig=254,16 Ref=off

Peak #	RetTime [min]	Type	Width [min]	Area [mAU*s]	Height [mAU]	Area %
1	29.928	MM	1.3856	2392.66748	28.77951	49.9257
2	35.268	MM	1.7785	2399.78638	22.48858	50.0743

Totals : 4792.45386 51.26810

===

*** End of Report ***

Data File E:\LCDATA\Data\lilun\2021-11-2112-17-26ll-11-shou.D
Sample Name: ll-11-shou

===

Acq. Operator : 系统
Sample Operator : 系统
Acq. Instrument : LC1260 Location : 21
Injection Date : 11/21/2021 12:18:22 PM
Inj Volume : 10.000 µl
Acq. Method : E:\LCDATA\Methods\LI-LUN.M
Last changed : 11/21/2021 10:43:27 AM by 系统
(modified after loading)
Analysis Method : E:\LCDATA\Methods\LI-LUN.M
Last changed : 3/14/2022 4:52:34 PM by 系统
(modified after loading)
Sample Info : oD 90:10 1

Additional Info : Peak(s) manually integrated

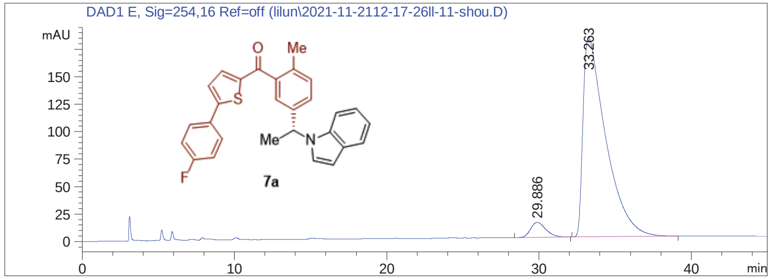

===

Area Percent Report

===

Sorted By : Signal
Multiplier : 1.0000
Dilution : 1.0000
Sample Amount: : 10.00000 [ng/ul] (not used in calc.)
Do not use Multiplier & Dilution Factor with ISTDs

Signal 1: DAD1 E, Sig=254,16 Ref=off

Peak #	RetTime [min]	Type	Width [min]	Area [mAU*s]	Height [mAU]	Area %
1	29.886	BB	1.0073	955.47418	13.67330	4.8349
2	33.263	BB	1.4885	1.88064e4	181.55702	95.1651

Totals : 1.97619e4 195.23032

===

*** End of Report ***

Supplementary Figure 242. HPLC spectra of compound **7a**

Data File E:\LCDATA\Data\lilun\2021-11-2320-39-00ll-2-40-1.D
Sample Name: ll-2-40-1

===

Acq. Operator : 系统
Sample Operator : 系统
Acq. Instrument : LC1260 Location : 21
Injection Date : 11/23/2021 8:39:54 PM
Inj Volume : 10.000 µl
Acq. Method : E:\LCDATA\Methods\LI-LUN.M
Last changed : 11/23/2021 8:32:51 PM by 系统
(modified after loading)
Analysis Method : E:\LCDATA\Methods\LI-LUN.M
Last changed : 3/14/2022 4:54:40 PM by 系统
(modified after loading)
Sample Info : oD 65:35 1

Additional Info : Peak(s) manually integrated

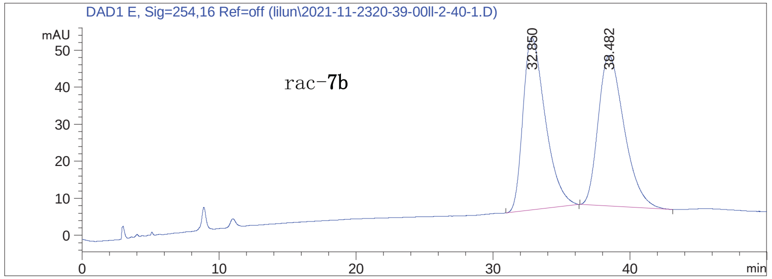

===

Area Percent Report

===

Sorted By : Signal
Multiplier : 1.0000
Dilution : 1.0000
Sample Amount: : 10.00000 [ng/ul] (not used in calc.)
Do not use Multiplier & Dilution Factor with ISTDs

Signal 1: DAD1 E, Sig=254,16 Ref=off

Peak #	RetTime [min]	Type	Width [min]	Area [mAU*s]	Height [mAU]	Area %
1	32.850	BB	1.6087	5276.97607	46.54453	49.6588
2	38.482	BB	1.7737	5349.50146	40.63887	50.3412

Totals : 1.06265e4 87.18339

===

*** End of Report ***

Data File E:\LCDATA\Data\lilun\2021-11-2321-37-40ll-2-40-2.D
Sample Name: ll-2-40-2

===

Acq. Operator : 系统
Sample Operator : 系统
Acq. Instrument : LC1260 Location : 21
Injection Date : 11/23/2021 9:38:37 PM
Inj Volume : 10.000 µl
Acq. Method : E:\LCDATA\Methods\LI-LUN.M
Last changed : 11/23/2021 8:32:51 PM by 系统
(modified after loading)
Analysis Method : E:\LCDATA\Methods\LI-LUN.M
Last changed : 3/14/2022 4:54:40 PM by 系统
(modified after loading)
Sample Info : oD 65:35 1

Additional Info : Peak(s) manually integrated

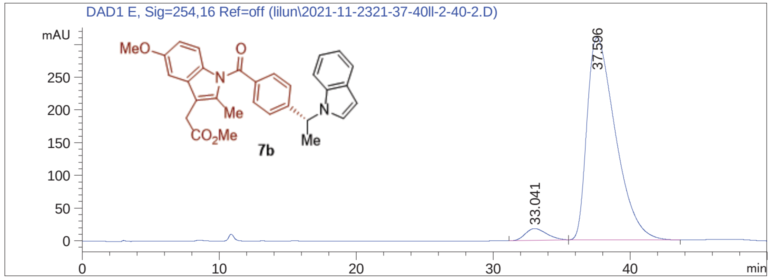

===

Area Percent Report

===

Sorted By : Signal
Multiplier : 1.0000
Dilution : 1.0000
Sample Amount: : 10.00000 [ng/ul] (not used in calc.)
Do not use Multiplier & Dilution Factor with ISTDs

Signal 1: DAD1 E, Sig=254,16 Ref=off

Peak #	RetTime [min]	Type	Width [min]	Area [mAU*s]	Height [mAU]	Area %
1	33.041	BB	1.3415	1947.47705	18.09626	4.1804
2	37.596	BB	2.1684	4.46386e4	309.17697	95.8196

Totals : 4.65861e4 327.27323

===

*** End of Report ***

Supplementary Figure 243. HPLC spectra of compound **7b**

Data File E:\LCDATA\Data\lilun\2021-12-2409-42-50ll-1kiti-35-RR.D
Sample Name: ll-1kiti-35-RR

===

Acq. Operator   : 系统
Sample Operator : 系统
Acq. Instrument : LC1260   Location :   21
Injection Date : 12/24/2021 9:43:46 AM
                                                Inj Volume : 10.000 µl
Acq. Method   : E:\LCDATA\Methods\LI-LUN.M
Last changed   : 12/24/2021 8:43:57 AM by 系统
                  (modified after loading)
Analysis Method : E:\LCDATA\Methods\LI-LUN.M
Last changed   : 3/14/2022 4:57:10 PM by 系统
                  (modified after loading)
Sample Info   : OJ-H 90/1 30du

Additional Info : Peak(s) manually integrated

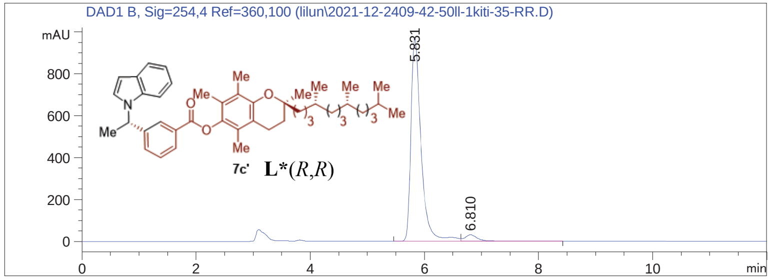

===

Area Percent Report

===

Sorted By   :   Signal
Multiplier   :   1.0000
Dilution   :   1.0000
Sample Amount: :   10.00000 [ng/ul]   (not used in calc.)
Do not use Multiplier & Dilution Factor with ISTDs

Signal 1: DAD1 B, Sig=254,4 Ref=360,100

Peak #	RetTime [min]	Type	Width [min]	Area [mAU*s]	Height [mAU]	Area %
1	5.831	BV R	0.1821	1.20015e4	972.22937	95.8791
2	6.810	VB E	0.2418	515.82813	31.04740	4.1209

Totals :   1.25173e4 1003.27677

===

*** End of Report ***

Data File E:\LCDATA\Data\lilun\2021-12-2409-23-18ll-1kiti-35-ss.D
Sample Name: ll-1kiti-35-ss

===

Acq. Operator   : 系统
Sample Operator : 系统
Acq. Instrument : LC1260   Location :   21
Injection Date : 12/24/2021 9:24:11 AM
                                                Inj Volume : 10.000 µl
Acq. Method   : E:\LCDATA\Methods\LI-LUN.M
Last changed   : 12/24/2021 8:43:57 AM by 系统
                  (modified after loading)
Analysis Method : E:\LCDATA\Methods\LI-LUN.M
Last changed   : 3/14/2022 4:57:10 PM by 系统
                  (modified after loading)
Sample Info   : AD-H   80:20 1

Additional Info : Peak(s) manually integrated

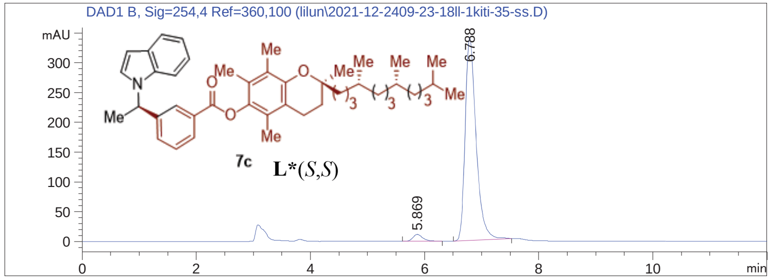

===

Area Percent Report

===

Sorted By   :   Signal
Multiplier   :   1.0000
Dilution   :   1.0000
Sample Amount: :   10.00000 [ng/ul]   (not used in calc.)
Do not use Multiplier & Dilution Factor with ISTDs

Signal 1: DAD1 B, Sig=254,4 Ref=360,100

Peak #	RetTime [min]	Type	Width [min]	Area [mAU*s]	Height [mAU]	Area %
1	5.869	BB	0.1829	135.46745	11.37699	2.9056
2	6.788	BB	0.2010	4526.82959	340.62744	97.0944

Totals :   4662.29704 352.00443

===

*** End of Report ***

Supplementary Figure 244. HPLC spectra of compound **7c & 7c'**

Data File E:\LCDATA\Data\lilun\2021-12-2011-35-55ll-1kiti-24-RR.D
Sample Name: ll-1kiti-24-RR

===
Acq. Operator   : 系统
Sample Operator : 系统
Acq. Instrument : LC1260   Location :   21
Injection Date : 12/20/2021 11:36:48 AM
Inj Volume : 10.000 µl
Acq. Method   : E:\LCDATA\Methods\LI-LUN.M
Last changed   : 12/20/2021 10:23:33 AM by 系统
(modified after loading)
Analysis Method : E:\LCDATA\Methods\LI-LUN.M
Last changed   : 3/14/2022 5:01:51 PM by 系统
(modified after loading)
Sample Info   : Od-H 90/1

Additional Info : Peak(s) manually integrated

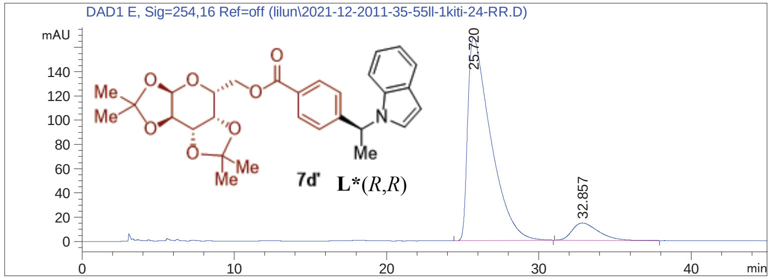

===
Area Percent Report
===

Sorted By   :   Signal
Multiplier   :   1.0000
Dilution   :   1.0000
Sample Amount: :   10.00000 [ng/ul]   (not used in calc.)
Do not use Multiplier & Dilution Factor with ISTDs

Signal 1: DAD1 E, Sig=254,16 Ref=off

Peak #	RetTime [min]	Type	Width [min]	Area [mAU*s]	Height [mAU]	Area %
1	25.720	BB	1.4917	1.74882e4	167.55869	91.1813
2	32.857	BB	1.5597	1691.38599	14.24063	8.8187

Totals :   1.91796e4   181.79932

===
*** End of Report ***

Data File E:\LCDATA\Data\lilun\2021-12-2010-23-43ll-1kiti-24-SS.D
Sample Name: ll-1kiti-24-SS

===
Acq. Operator   : 系统
Sample Operator : 系统
Acq. Instrument : LC1260   Location :   21
Injection Date : 12/20/2021 10:24:41 AM
Inj Volume : 10.000 µl
Acq. Method   : E:\LCDATA\Methods\LI-LUN.M
Last changed   : 12/20/2021 10:23:33 AM by 系统
(modified after loading)
Analysis Method : E:\LCDATA\Methods\LI-LUN.M
Last changed   : 3/14/2022 5:01:51 PM by 系统
(modified after loading)
Sample Info   : Od-H 90/1

Additional Info : Peak(s) manually integrated

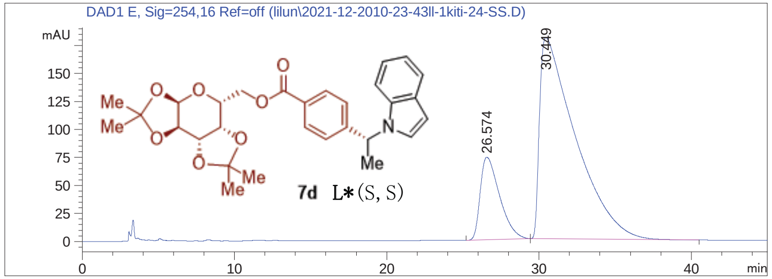

===
Area Percent Report
===

Sorted By   :   Signal
Multiplier   :   1.0000
Dilution   :   1.0000
Sample Amount: :   10.00000 [ng/ul]   (not used in calc.)
Do not use Multiplier & Dilution Factor with ISTDs

Signal 1: DAD1 E, Sig=254,16 Ref=off

Peak #	RetTime [min]	Type	Width [min]	Area [mAU*s]	Height [mAU]	Area %
1	26.574	BB	1.3271	6531.16406	73.89905	18.1548
2	30.449	BB	2.1811	2.94437e4	179.74051	81.8452

Totals :   3.59748e4   253.63956

===
*** End of Report ***

Supplementary Figure 245. HPLC spectra of compound **7d & 7d'**

Data File E:\LCDATA\Data\lilun\2022-01-1720-08-00ll-1kiti-45-rr.D
Sample Name: ll-1kiti-45-rr

===

Acq. Instrument : LC1260 Location : 21

Inj Volume : 10.000 µl

Acq. Method : E:\LCDATA\Methods\LI-LUN.M
Last changed : 1/17/2022 6:13:10 PM by 系统
(modified after loading)
Analysis Method : E:\LCDATA\Methods\LI-LUN.M
Last changed : 3/14/2022 5:05:45 PM by 系统
(modified after loading)
Sample Info : 90/1 ic ;20C

Additional Info : Peak(s) manually integrated

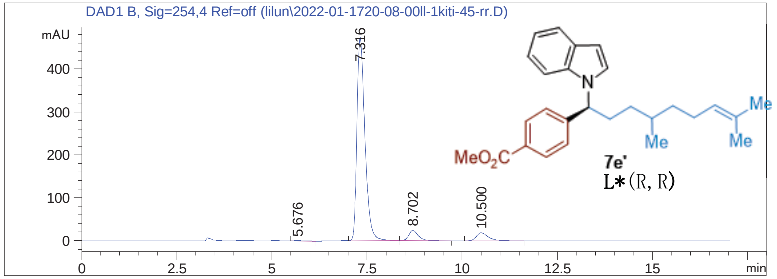

===
Area Percent Report
===

Sorted By : Signal
Multiplier : 1.0000
Dilution : 1.0000
Sample Amount: : 10.00000 [ng/ul] (not used in calc.)
Do not use Multiplier & Dilution Factor with ISTDs

Signal 1: DAD1 B, Sig=254,4 Ref=off

Peak #	RetTime [min]	Type	Width [min]	Area [mAU*s]	Height [mAU]	Area %
1	5.676	BB	0.1577	17.26036	1.65456	0.2227
2	7.316	BB	0.2243	6914.55811	473.62097	89.2124
3	8.702	BB	0.2633	409.51080	23.73208	5.2836
4	10.500	BB	0.3319	409.34238	18.66511	5.2814

Totals : 7750.67165 517.67272

===
*** End of Report ***

Data File E:\LCDATA\Data\lilun\2022-01-1719-23-59ll-1kiti-45-ss.D
Sample Name: ll-1kiti-45-ss

===
Acq. Operator : 系统
Sample Operator : 系统
Acq. Instrument : LC1260 Location : 21
Injection Date : 1/17/2022 7:24:56 PM
Inj Volume : 10.000 µl
Acq. Method : E:\LCDATA\Methods\LI-LUN.M
Last changed : 1/17/2022 6:13:10 PM by 系统
(modified after loading)
Analysis Method : E:\LCDATA\Methods\LI-LUN.M
Last changed : 3/14/2022 5:05:45 PM by 系统
(modified after loading)
Sample Info : 90/1 ic ;20C

Additional Info : Peak(s) manually integrated

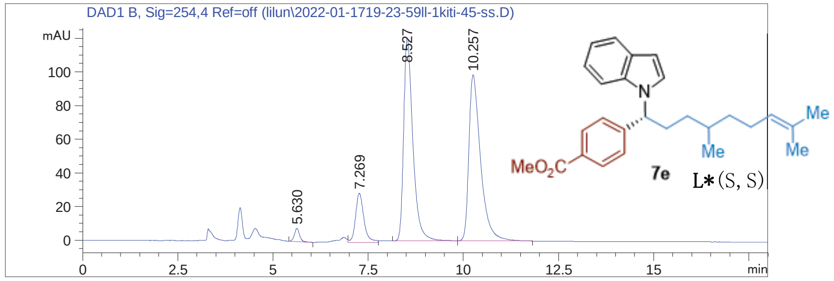

===
Area Percent Report
===

Sorted By : Signal
Multiplier : 1.0000
Dilution : 1.0000
Sample Amount: : 10.00000 [ng/ul] (not used in calc.)
Do not use Multiplier & Dilution Factor with ISTDs

Signal 1: DAD1 B, Sig=254,4 Ref=off

Peak #	RetTime [min]	Type	Width [min]	Area [mAU*s]	Height [mAU]	Area %
1	5.630	BB	0.1439	72.83354	7.72917	1.5652
2	7.269	MM	0.2574	452.59250	29.30056	9.7265
3	8.527	BB	0.2545	2028.57922	120.45522	43.5954
4	10.257	BBA	0.3185	2099.19531	98.54141	45.1129

Totals : 4653.20058 256.02636

===
*** End of Report ***

Supplementary Figure 246. HPLC spectra of compound **7e & 7e'**

Data File E:\DATA\RJT\2022-01-1323-59-56RJT-ZH-1 RACE.D
Sample Name: RJT-ZH-1 RACE

===

Acq. Operator : 系统
Sample Operator : 系统
Acq. Instrument : LC1 Location : -
Injection Date : 2022/1/13 23:59:56
Inj Volume :
Acq. Method : C:\Chem32\1\Methods\ybm.M
Last changed : 2022/1/13 23:35:59 : 系统
(modified after loading)
Analysis Method : C:\Chem32\1\Methods\def_LC.M
Last changed : 2022/4/2 12:48:54 : 系统
(modified after loading)
Sample Info : IC-3, hex/ipr=99/1, 254 nm, 1.0 ml/min, 45.97 bar

Additional Info : Peak(s) manually integrated

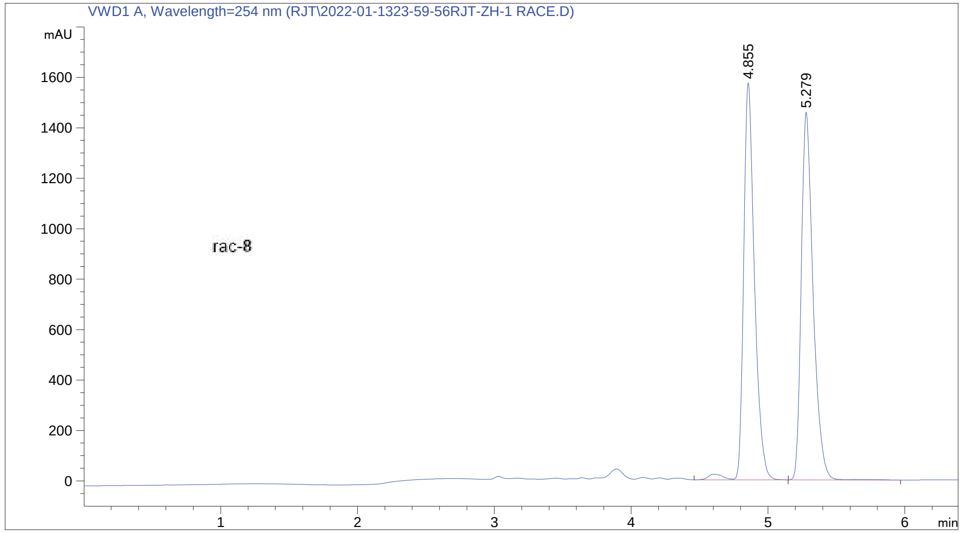

===
Area Percent Report
===

Sorted By : Signal
Multiplier : 1.0000
Dilution : 1.0000
Sample Amount: : 1.00000 [ng/ul] (not used in calc.)
Use Multiplier & Dilution Factor with ISTDs

Signal 1: VWD1 A, Wavelength=254 nm

Peak #	RetTime [min]	Type	Width [min]	Area [mAU*s]	Height [mAU]	Area %
1	4.855	VB R	0.0826	8743.64551	1572.62476	49.8193
2	5.279	BV R	0.0912	8807.07520	1457.97900	50.1807

Data File E:\DATA\RJT\2022-01-1400-43-16RJT-ZH-1 CHIRAL.D
Sample Name: RJT-ZH-1 CHIRAL

===

Acq. Operator : 系统
Sample Operator : 系统
Acq. Instrument : LC1 Location : -
Injection Date : 2022/1/14 0:43:16
Inj Volume :
Acq. Method : C:\Chem32\1\Methods\ybm.M
Last changed : 2022/1/13 23:35:59 : 系统
(modified after loading)
Analysis Method : C:\Chem32\1\Methods\def_LC.M
Last changed : 2022/4/2 12:48:06 : 系统
(modified after loading)
Sample Info : IC-3, hex/ipr=99/1, 254 nm, 1.0 ml/min, 74.17 bar

Additional Info : Peak(s) manually integrated

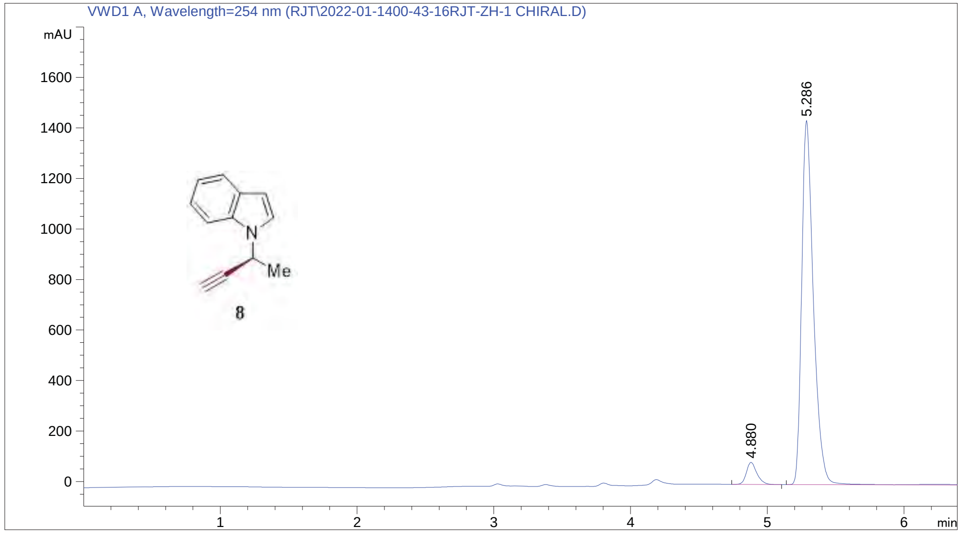

===
Area Percent Report
===

Sorted By : Signal
Multiplier : 1.0000
Dilution : 1.0000
Sample Amount: : 1.00000 [ng/ul] (not used in calc.)
Use Multiplier & Dilution Factor with ISTDs

Signal 1: VWD1 A, Wavelength=254 nm

Peak #	RetTime [min]	Type	Width [min]	Area [mAU*s]	Height [mAU]	Area %
1	4.880	BB	0.0802	467.34250	87.68377	5.1012
2	5.286	BV R	0.0899	8694.15234	1441.27039	94.8988

LC1 2022/4/2 12:48:55 系统

Supplementary Figure 247. HPLC spectra of compound **8**

Page 1/1

Data File E:\DATA\RJT\2022-01-2813-01-29RJT-ZH-2.D
Sample Name: RJT-ZH-2

===

Acq. Operator : 系统
Sample Operator : 系统
Acq. Instrument : LC1 Location : -
Injection Date : 2022/1/28 13:01:29
Inj Volume :
Acq. Method : C:\Chem32\1\Methods\ybm.M
Last changed : 2022/1/28 12:13:49 : 系统
(modified after loading)
Analysis Method : C:\Chem32\1\Methods\def_LC.M
Last changed : 2022/4/2 12:49:53 : 系统
(modified after loading)
Sample Info : IB-3, hex/ipr=99/1, 254 nm, 1.0 ml/min, 73.75 bar

Additional Info : Peak(s) manually integrated

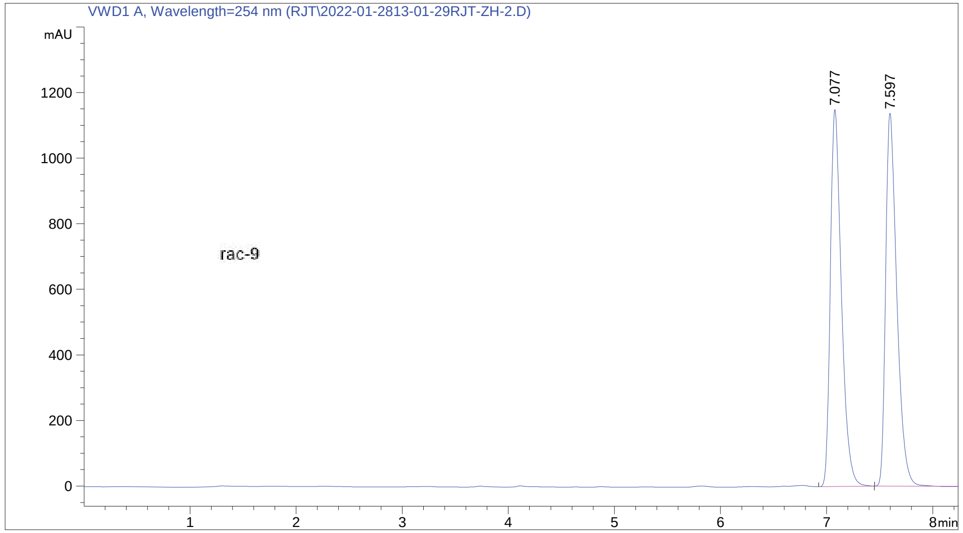

===
Area Percent Report
===

Sorted By : Signal
Multiplier : 1.0000
Dilution : 1.0000
Sample Amount: : 1.00000 [ng/ul] (not used in calc.)
Use Multiplier & Dilution Factor with ISTDs

Signal 1: VWD1 A, Wavelength=254 nm

Peak #	RetTime [min]	Type	Width [min]	Area [mAU*s]	Height [mAU]	Area %
1	7.077	BB	0.1104	8313.10645	1149.23730	49.9901
2	7.597	BB	0.1113	8316.38574	1137.29529	50.0099

LC1 2022/4/2 12:49:56 系统

Data File E:\DATA\RJT\2022-01-2811-29-06RJT-ZH-2.D
Sample Name: RJT-ZH-2

===

Acq. Operator : 系统
Sample Operator : 系统
Acq. Instrument : LC1 Location : -
Injection Date : 2022/1/28 11:29:06
Inj Volume :
Acq. Method : C:\Chem32\1\Methods\ybm.M
Last changed : 2022/1/28 11:28:11 : 系统
(modified after loading)
Analysis Method : C:\Chem32\1\Methods\def_LC.M
Last changed : 2022/4/2 12:50:23 : 系统
(modified after loading)
Sample Info : IB-3, hex/ipr=99/1, 254 nm, 1.0 ml/min, 73.75 bar

Additional Info : Peak(s) manually integrated

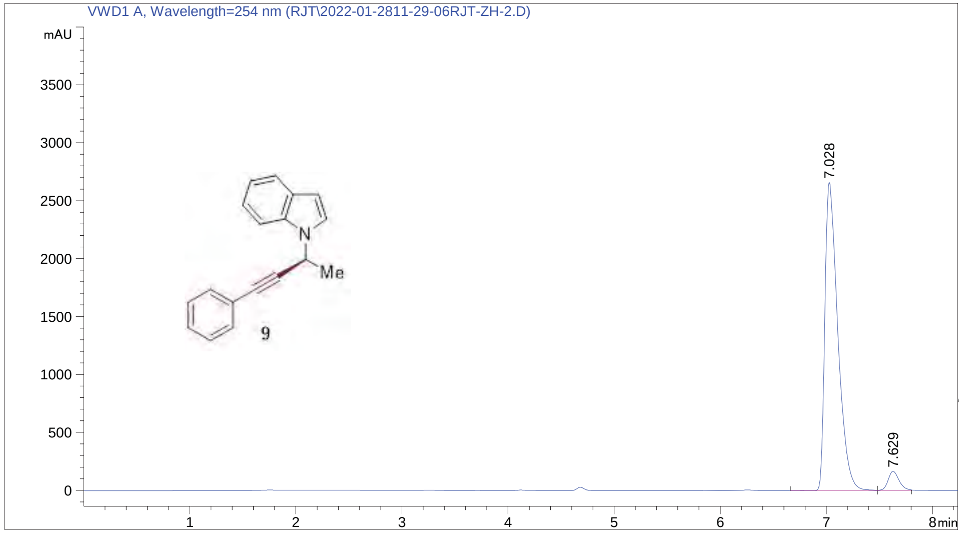

===
Area Percent Report
===

Sorted By : Signal
Multiplier : 1.0000
Dilution : 1.0000
Sample Amount: : 1.00000 [ng/ul] (not used in calc.)
Use Multiplier & Dilution Factor with ISTDs

Signal 1: VWD1 A, Wavelength=254 nm

Peak #	RetTime [min]	Type	Width [min]	Area [mAU*s]	Height [mAU]	Area %
1	7.028	VV R	0.1289	2.25578e4	2650.33252	94.9126
2	7.629	MF	0.1210	1209.11279	166.61293	5.0874

Page 1/1

Supplementary Figure 248. HPLC spectra of compound **9**

Data File E:\DATA\RJT\2022-02-1617-39-04N3.D
Sample Name: N3

===

Acq. Operator : 系统
Sample Operator : 系统
Acq. Instrument : LC1 Location : -
Injection Date : 2022/2/16 17:39:04
Inj Volume :
Acq. Method : C:\Chem32\1\Methods\ybm.M
Last changed : 2022/2/16 17:33:44 : 系统
(modified after loading)
Analysis Method : C:\Chem32\1\Methods\def_LC.M
Last changed : 2022/4/2 12:55:01 : 系统
(modified after loading)
Sample Info : IC, hex/ipr=80:20, 254 nm, 1.0 ml/min, 41.06 bar

Additional Info : Peak(s) manually integrated

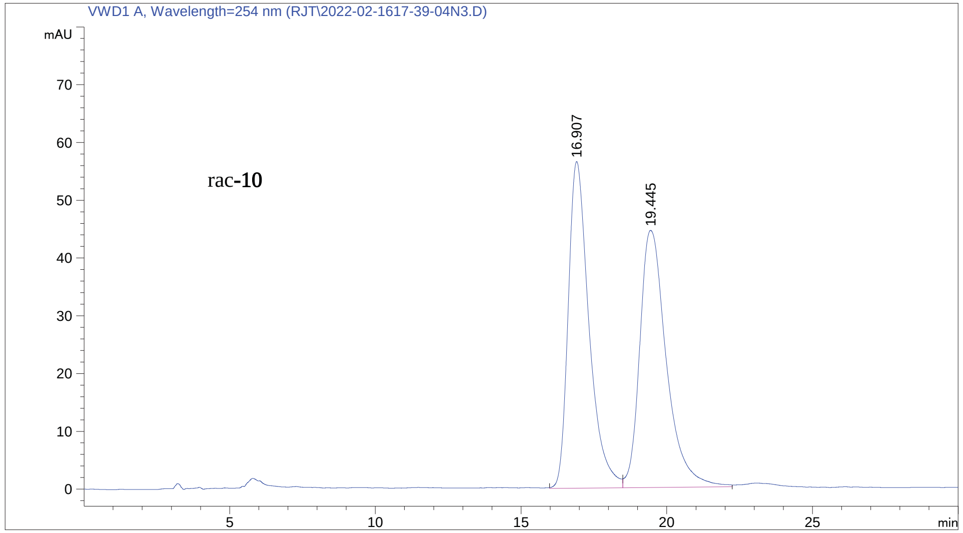

===
Area Percent Report
===

Sorted By : Signal
Multiplier : 1.0000
Dilution : 1.0000
Sample Amount: : 1.00000 [ng/ul] (not used in calc.)
Use Multiplier & Dilution Factor with ISTDs

Signal 1: VWD1 A, Wavelength=254 nm

Peak #	RetTime [min]	Type	Width [min]	Area [mAU*s]	Height [mAU]	Area %
1	16.907	MF	0.8268	2804.38892	56.53105	49.7666
2	19.445	FM	1.0603	2830.69116	44.49511	50.2334

LC1 2022/4/2 12:55:03 系统

Data File E:\DATA\RJT\2022-02-1618-12-02N3.D
Sample Name: N3

===

Acq. Operator : 系统
Sample Operator : 系统
Acq. Instrument : LC1 Location : -
Injection Date : 2022/2/16 18:12:02
Inj Volume :
Acq. Method : C:\Chem32\1\Methods\ybm.M
Last changed : 2022/2/16 17:33:44 : 系统
(modified after loading)
Analysis Method : C:\Chem32\1\Methods\def_LC.M
Last changed : 2022/4/2 12:54:02 : 系统
(modified after loading)
Sample Info : IC, hex/ipr=80:20, 254 nm, 1.0 ml/min, 41.06 bar

Additional Info : Peak(s) manually integrated

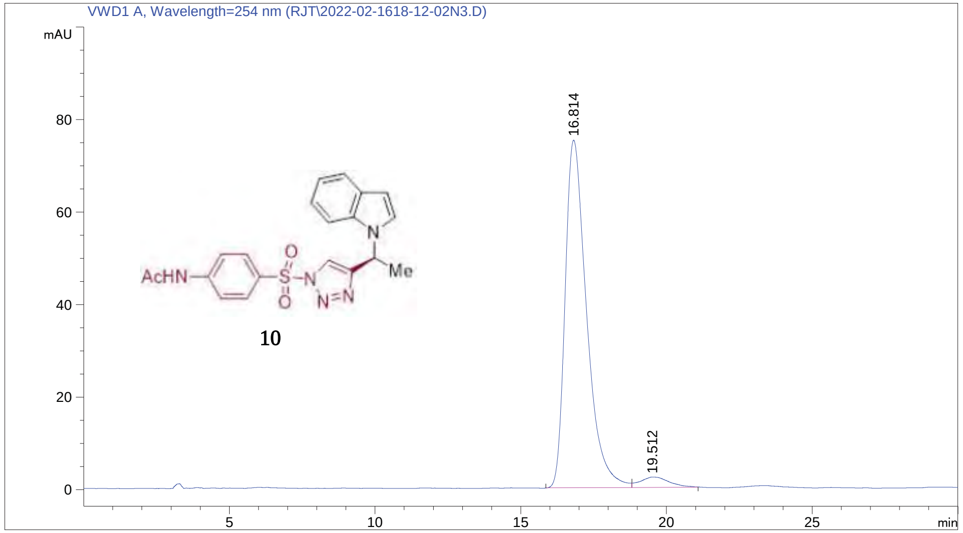

===
Area Percent Report
===

Sorted By : Signal
Multiplier : 1.0000
Dilution : 1.0000
Sample Amount: : 1.00000 [ng/ul] (not used in calc.)
Use Multiplier & Dilution Factor with ISTDs

Signal 1: VWD1 A, Wavelength=254 nm

Peak #	RetTime [min]	Type	Width [min]	Area [mAU*s]	Height [mAU]	Area %
1	16.814	MF	0.8527	3842.66406	75.11092	96.1186
2	19.512	FM	1.1524	155.17097	2.24412	3.8814

Page 1/1

Supplementary Figure 247. HPLC spectra of compound **10**

Data File D:\Chem32\1\Data\RenJiangTao\R-11-R 2022-09-10 21-04-40.D
Sample Name: R-11-R

===
Acq. Operator : SYSTEM
Sample Operator : SYSTEM
Acq. Instrument : 1260 Location : 1
Injection Date : 9/10/2022 9:04:40 PM
Inj Volume : No inj
Acq. Method : D:\Chem32\1\Methods\DEF_LC.M
Last changed : 9/10/2022 8:24:15 PM by SYSTEM
(modified after loading)
Analysis Method : D:\Chem32\1\Methods\DEF_LC.M
Last changed : 9/13/2022 9:39:34 AM by SYSTEM
(modified after loading)
Sample Info : OD-H, Hex:Ipr=99/1, 1.0 ml/min, 254 nm,44.05 bar

Additional Info : Peak(s) manually integrated

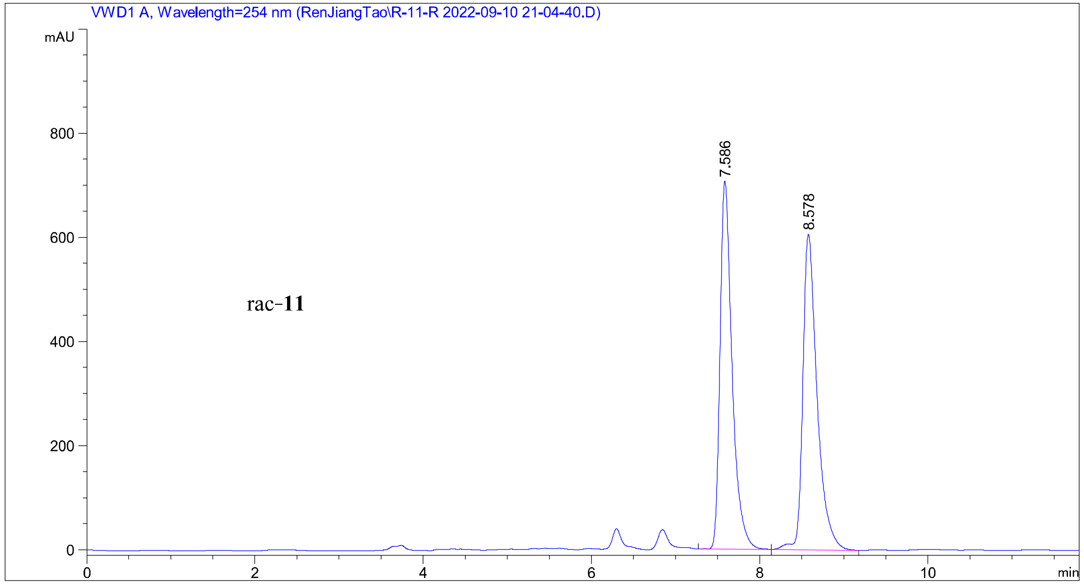

===
Area Percent Report
===

Sorted By : Signal
Multiplier : 1.0000
Dilution : 1.0000
Use Multiplier & Dilution Factor with ISTDs

Signal 1: VWD1 A, Wavelength=254 nm

Peak #	RetTime [min]	Type	Width [min]	Area [mAU*s]	Height [mAU]	Area %
1	7.586	VV R	0.1494	7044.81201	706.15625	49.5752
2	8.578	VB R	0.1742	7165.53760	606.04309	50.4248

Data File D:\Chem32\1\Data\RenJiangTao\RJT-11-C 2022-09-13 21-26-53.D
Sample Name: RJT-11-C

===
Acq. Operator : SYSTEM
Sample Operator : SYSTEM
Acq. Instrument : 1260 Location : 1
Injection Date : 9/13/2022 9:26:53 PM
Inj Volume : No inj
Acq. Method : D:\Chem32\1\Methods\DEF_LC.M
Last changed : 9/13/2022 8:52:01 PM by SYSTEM
(modified after loading)
Analysis Method : D:\Chem32\1\Methods\DEF_LC.M
Last changed : 9/14/2022 9:19:54 AM by SYSTEM
(modified after loading)
Sample Info : OD-H, Hex:Ipr=99/1, 1.0 ml/min, 254 nm,43.60 bar

Additional Info : Peak(s) manually integrated

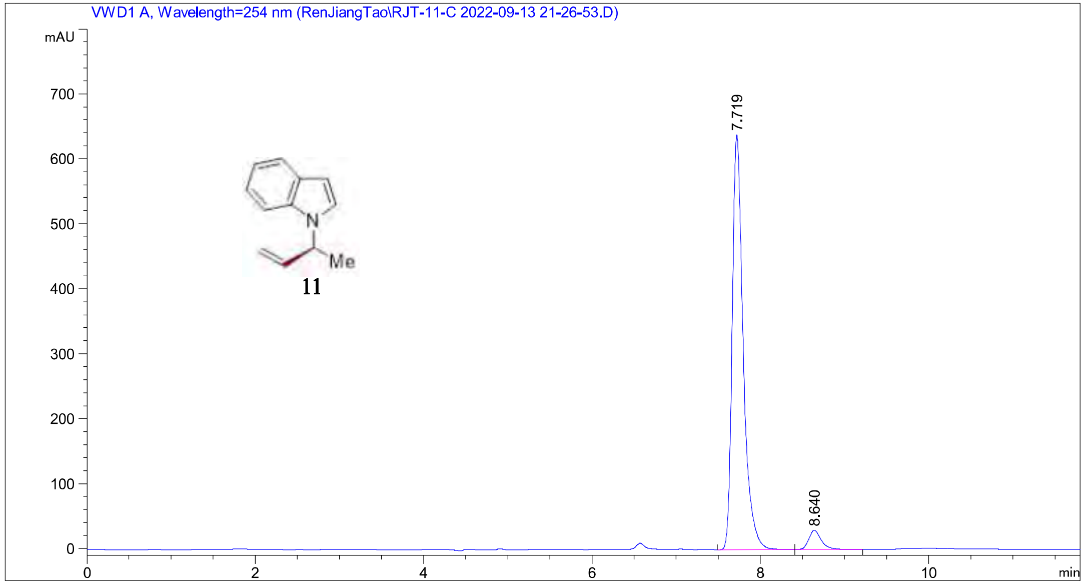

===
Area Percent Report
===

Sorted By : Signal
Multiplier : 1.0000
Dilution : 1.0000
Use Multiplier & Dilution Factor with ISTDs

Signal 1: VWD1 A, Wavelength=254 nm

Peak #	RetTime [min]	Type	Width [min]	Area [mAU*s]	Height [mAU]	Area %
1	7.719	BV	0.1370	5801.58496	638.45111	94.8900
2	8.640	VB	0.1594	312.42300	29.77879	5.1100

Supplementary Figure 250. HPLC spectra of compound **11**

Data File E:\DATA\RJT\2022-02-1522-51-24DHHY.D
Sample Name: DHHY

===

Acq. Operator : 系统
Sample Operator : 系统
Acq. Instrument : LC1 Location : -
Injection Date : 2022/2/15 22:51:24
Inj Volume :
Acq. Method : C:\Chem32\1\Methods\ybm.M
Last changed : 2022/2/15 22:12:38 : 系统
(modified after loading)
Analysis Method : C:\Chem32\1\Methods\def_LC.M
Last changed : 2022/4/2 12:52:47 : 系统
(modified after loading)
Sample Info : IB-3, hex/ipr=100:0, 254 nm, 1.0 ml/min, 66.41 bar

Additional Info : Peak(s) manually integrated

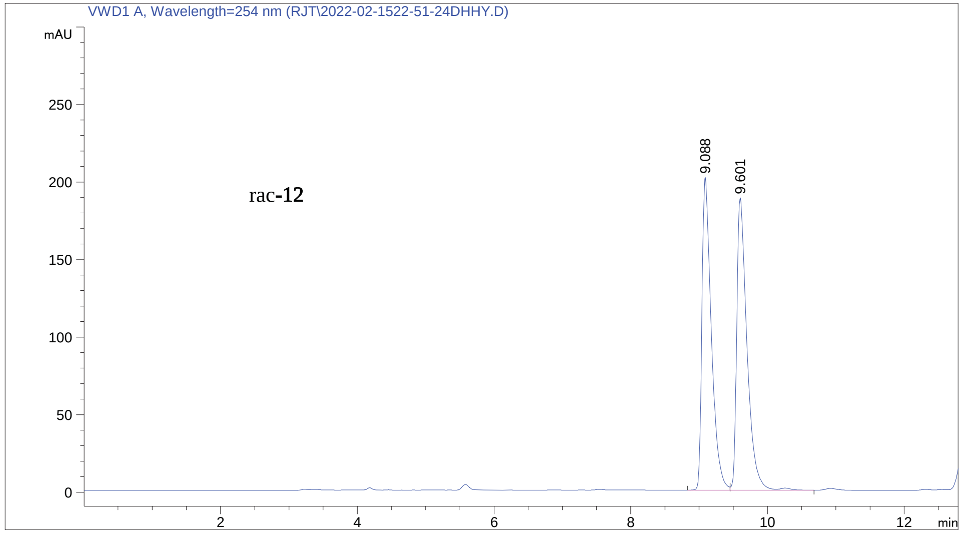

===
Area Percent Report
===

Sorted By : Signal
Multiplier : 1.0000
Dilution : 1.0000
Sample Amount: : 1.00000 [ng/ul] (not used in calc.)
Use Multiplier & Dilution Factor with ISTDs

Signal 1: VWD1 A, Wavelength=254 nm

Peak #	RetTime [min]	Type	Width [min]	Area [mAU*s]	Height [mAU]	Area %
1	9.088	BV	0.1424	1926.16858	201.65405	49.4822
2	9.601	VV R	0.1548	1966.48145	188.38391	50.5178

Data File E:\DATA\RJT\2022-02-1523-16-28DHHY.D
Sample Name: DHHY

===

Acq. Operator : 系统
Sample Operator : 系统
Acq. Instrument : LC1 Location : -
Injection Date : 2022/2/15 23:16:28
Inj Volume :
Acq. Method : C:\Chem32\1\Methods\ybm.M
Last changed : 2022/2/15 22:12:38 : 系统
(modified after loading)
Analysis Method : C:\Chem32\1\Methods\def_LC.M
Last changed : 2022/4/2 12:52:21 : 系统
(modified after loading)
Sample Info : IB-3, hex/ipr=100:0, 254 nm, 1.0 ml/min, 66.41 bar

Additional Info : Peak(s) manually integrated

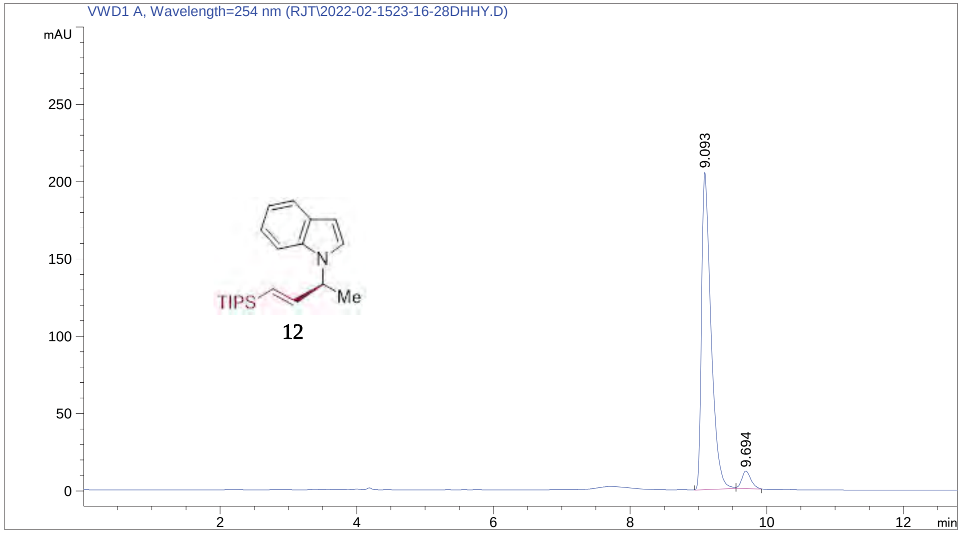

===
Area Percent Report
===

Sorted By : Signal
Multiplier : 1.0000
Dilution : 1.0000
Sample Amount: : 1.00000 [ng/ul] (not used in calc.)
Use Multiplier & Dilution Factor with ISTDs

Signal 1: VWD1 A, Wavelength=254 nm

Peak #	RetTime [min]	Type	Width [min]	Area [mAU*s]	Height [mAU]	Area %
1	9.093	MM	0.1602	1972.45972	205.14821	94.8928
2	9.694	MM	0.1555	106.15915	11.38127	5.1072

LC1 2022/4/2 12:52:49 系统

Supplementary Figure 251. HPLC spectra of compound **12**

Page 1/1

2. Supplementary References

1. Jiang, B. & Shi, M. Rhodium(ii)-catalyzed intermolecular [3+2] annulation of *N*-vinyl indoles with N-tosyl-1,2,3-triazoles via an aza-vinyl Rh carbene. *Org. Chem. Front.* **4**, 2459-2464 (2017).
2. Rattanangkool, E., Vilaivan, T., Sukwattanasinitt, M. & Wacharasindhu, S. An Atom-Economic Approach for Vinylation of Indoles and Phenols Using Calcium Carbide as Acetylene Surrogate. *Eur. J. Org. Chem.* **2016**, 4347-4353 (2016).
3. Bai, J.-F. *et al.* Organocatalytic Formal (3+2) Cycloaddition toward Chiral Pyrrolo[1,2-a]indoles via Dynamic Kinetic Resolution of Allene Intermediates. *Org. Lett.* **22**, 5439-5445 (2020).
4. Liao, Q., Wang, Y., Zhang, L. & Xi, C. A General Copper-Catalyzed Coupling of Azoles with Vinyl Bromides. *J. Org. Chem.* **74**, 6371-6373 (2009).
5. Amos, S. G. E., Nicolai, S. & Waser, J. Photocatalytic Umpolung of *N*- and *O*-substituted alkenes for the synthesis of 1,2-amino alcohols and diols. *Chem. Sci.* **11**, 11274-11279 (2020).
6. Shen, J.-J. *et al.* Cu-NHC-Catalyzed Enantioselective Conjugate Silyl addition to Indol-1-ylacrylate Derivatives. *ChemistrySelect* **4**, 11358-11361 (2019).
7. Bacauanu, V. *et al.* Metallaphotoredox Difluoromethylation of Aryl Bromides. *Angew. Chem. Int. Ed.* **57**, 12543-12548 (2018).
8. Bera, S. & Hu, X. Nickel-Catalyzed Regioselective Hydroalkylation and Hydroarylation of Alkenyl Boronic Esters. *Angew. Chem. Int. Ed.* **58**, 13854-13859 (2019).
9. Pan, R. *et al.* Nickel-Catalyzed Reductive 1,2-Dialkynylation of Alkenes Bearing an 8-Aminoquinoline Directing Group. *Org. Lett.* **21**, 8915-8920 (2019).
10. Li, Y. *et al.* Nickel-Catalyzed Chemodivergent 1,1-Difunctionalization of Unactivated α-Olefins with Alkynyl Electrophiles and B_2pin_2. *ACS Catal.* **10**, 4888-4894 (2020).
11. Pezzetta, C., Bonifazi, D. & Davidson, R. W. M. Enantioselective Synthesis of *N*-Benzylic Heterocycles: A Nickel and Photoredox Dual Catalysis Approach. *Org. Lett.* **21**, 8957-8961 (2019).
12. Ye, Y., Kim, S.-T., Jeong, J., Baik, M.-H. & Buchwald, S. L. CuH-Catalyzed Enantioselective Alkylation of Indole Derivatives with Ligand-Controlled Regiodivergence. *J. Am. Chem. Soc.* **141**, 3901-3909 (2019).
13. Liu, W.-B., Zhang, X., Dai, L.-X. & You, S.-L. Asymmetric *N*-Allylation of Indoles Through the Iridium-Catalyzed Allylic Alkylation/Oxidation of Indolines. *Angew. Chem. Int. Ed.* **51**, 5183-5187 (2012).
